# Phylogenetic analysis of a new morphological dataset elucidates the evolutionary history of Crocodylia and resolves the long-standing gharial problem

**DOI:** 10.7717/peerj.12094

**Published:** 2021-09-06

**Authors:** Jonathan P. Rio, Philip D. Mannion

**Affiliations:** 1 Department of Earth Science and Engineering, Imperial College London, London, United Kingdom; 2 Department of Earth Sciences, University College London, London, United Kingdom

**Keywords:** Crocodylia, Phylogeny, Gharial problem, Allgatoroidea, Gavialoidea, Crocodyloidea, Extended implied weighting, Continuous characters

## Abstract

First appearing in the latest Cretaceous, Crocodylia is a clade of semi-aquatic, predatory reptiles, defined by the last common ancestor of extant alligators, caimans, crocodiles, and gharials. Despite large strides in resolving crocodylian interrelationships over the last three decades, several outstanding problems persist in crocodylian systematics. Most notably, there has been persistent discordance between morphological and molecular datasets surrounding the affinities of the extant gharials, *Gavialis gangeticus* and *Tomistoma schlegelii*. Whereas molecular data consistently support a sister taxon relationship, in which they are more closely related to crocodylids than to alligatorids, morphological data indicate that *Gavialis* is the sister taxon to all other extant crocodylians. Here we present a new morphological dataset for Crocodylia based on a critical reappraisal of published crocodylian character data matrices and extensive firsthand observations of a global sample of crocodylians. This comprises the most taxonomically comprehensive crocodylian dataset to date (144 OTUs scored for 330 characters) and includes a new, illustrated character list with modifications to the construction and scoring of characters, and 46 novel characters. Under a maximum parsimony framework, our analyses robustly recover *Gavialis* as more closely related to *Tomistoma* than to other extant crocodylians for the first time based on morphology alone. This result is recovered regardless of the weighting strategy and treatment of quantitative characters. However, analyses using continuous characters and extended implied weighting (with high *k*-values) produced the most resolved, well-supported, and stratigraphically congruent topologies overall. Resolution of the gharial problem reveals that: (1) several gavialoids lack plesiomorphic features that formerly drew them towards the stem of Crocodylia; and (2) more widespread similarities occur between species traditionally divided into tomistomines and gavialoids, with these interpreted here as homology rather than homoplasy. There remains significant temporal incongruence regarding the inferred divergence timing of the extant gharials, indicating that several putative gavialids (‘thoracosaurs’) are incorrectly placed and require future re-appraisal. New alligatoroid interrelationships include: (1) support for a North American origin of Caimaninae in the latest Cretaceous; (2) the recovery of the early Paleogene South American taxon *Eocaiman* as a ‘basal’ alligatoroid; and (3) the paraphyly of the Cenozoic European taxon *Diplocynodon*. Among crocodyloids, notable results include modifications to the taxonomic content of Mekosuchinae, including biogeographic affinities of this clade with latest Cretaceous–early Paleogene Asian crocodyloids. In light of our new results, we provide a comprehensive review of the evolutionary and biogeographic history of Crocodylia, which included multiple instances of transoceanic and continental dispersal.

## Introduction

Extant crocodylians are semi-aquatic ambush predators and piscivores that are globally distributed across the tropics and subtropics, inhabiting freshwater and estuarine environments (Grigg & Kirshner, 2015). They currently number 25 species, comprising alligators, caimans, crocodiles, and gharials; however, this number is most likely an underestimate given that several established species continue to be recognised as cryptic species complexes (*e.g*. Hekkala et al., 2011; Eaton et al., 2009; Shirley et al., 2018; Bittencourt et al., 2019; Brochu & Sumrall, 2020; Roberto et al., 2020). The last common ancestor of living crocodylians defines the crown group Crocodylia (Benton & Clark, 1988; Brochu et al., 2009). This group currently comprises approximately 140 recognised species, the earliest unambiguous members of which appear in the Campanian (latest Cretaceous), ~80 million years ago (Ma) (Brochu, 2003).

Crocodylians have been described as ‘living fossils’ partly because of their apparently conservative body plan (*e.g*. Langston, 1973; Meyer, 1984), and, over the course of the evolutionary history of their ancestors and extinct relatives (Crocodyliformes), they have repeatedly converged on similar skull shapes (*e.g*. Brochu, 2001; Sadleir & Makovicky, 2008; Wilberg, 2017; Ballell et al., 2019; Morris et al., 2019; Groh et al., 2020). Nevertheless, crocodylians have a rich evolutionary history (Brochu, 2003; Mannion et al., 2015). They exhibited dramatic differences in body size (Godoy et al., 2019; Gearty & Payne, 2020; Stockdale & Benton, 2021), ranging from dwarf forms such as *Osteolaemus* to the giant *Purussaurus*, the latter breaking the axial constraints exhibited in extant crocodylians (Scheyer et al., 2019). The fossil record of crocodylians also reveals a greater disparity in skull morphology than in extant taxa (Stubbs et al., 2013, 2021; Godoy, 2020), including ‘surfboard’-snouted forms such as *Mourasuchus* (Price, 1964) and the longirostrine, ‘saw’-like, narrow-snouted *Euthecodon* (Ginsburg & Buffetaut, 1978). It also provides evidence of a broader ecological diversity than their extant representatives. They transitioned from fully aquatic to terrestrial habitats at least twice (Wilberg, Turner & Brochu, 2019), and evolved feeding strategies beyond the carnivorous and piscivorous habits of extant taxa (Ősi, 2014; Gignac et al., 2019; Melstrom & Irmis, 2019; Drumheller & Wilberg, 2020). This includes durophagy (Salas-Gismondi et al., 2015) and possibly filter- or ‘gulp’-feeding (Langston, 1965; Cidade et al., 2017; Cidade, Riff & Hsiou, 2019). Furthermore, these diverse forms sometimes occupied the same habitat, greatly exceeding the number of sympatric occurrences in today’s crocodylian diversity hotspots (Scheyer et al., 2013; Salas-Gismondi et al., 2015).

Crocodylians also achieved a global distribution, including dispersals into high palaeolatitudes (*e.g*. Estes & Hutchison, 1980; Willis & Stilwell, 2000; Eberle et al., 2014) and across large oceanic barriers (Vélez-Juarbe, Brochu & Santos, 2007; Meredith et al., 2011; Oaks, 2011; Nicolaï & Matzke, 2019). The clade underwent a series of radiations and extinctions throughout its evolutionary history, including the survival of several lineages across the Cretaceous/Paleogene (K/Pg) mass extinction, 66 Ma (Markwick, 1998; Brochu, 2003; Bronzati, Montefeltro & Langer, 2015; Mannion et al., 2015; De Celis, Narváez & Ortega, 2020). Although likely constrained by a combination of abiotic and biotic factors (Solórzano et al., 2020; Stubbs et al., 2021), crocodylian diversification dynamics appear to show close ties to environmental and climatic fluctuations (Hutchison, 1982; Markwick, 1998; Brochu, 2003; Bronzati, Montefeltro & Langer, 2015; Mannion et al., 2015; De Celis, Narváez & Ortega, 2020; Pan et al., 2021).

Attempts at determining the evolutionary interrelationships of Crocodylia have a long history of study, dating back to the work of the earliest comparative anatomists on specimens of extant species (*e.g*. Duméril, 1806; Cuvier, 1807; Duméril & Bibron, 1835). However, the largest strides in resolving crocodylian phylogeny have occurred in the last four decades, from new morphological (*e.g*. Brochu, 1999a) and molecular data (*e.g*. Densmore & Owen, 1989; Green et al., 2014), as well as methodological advances in data analysis (*e.g*. Yang & Rannala, 2012). Nevertheless, there are still substantial gaps in our knowledge, as well as discrepancies between datasets, that stand in the way of a robust phylogeny of Crocodylia. Most notably, this includes the conflicting phylogenetic affinities of the extant gharial, *Gavialis gangeticus*, based on molecular and morphological datasets, but also includes a plethora of additional systematic problems. As well as hindering our understanding of the group’s evolutionary and biogeographic history, these problems also limit our ability to use phylogenetic trees to evaluate extinction risk and determine conservation priorities in extant species (*e.g*. Isaac et al., 2007; Gumbs et al., 2018, 2020; Colston et al., 2020).

### Previous studies of crocodylian interrelationships

**Figure 1:**
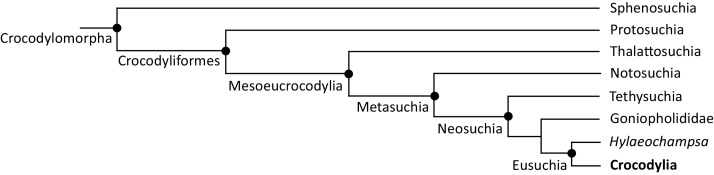
Simplified cladogram of Crocodylomorpha, after Wilberg, Turner & Brochu (2019).

The definition of Crocodylia as the crown-group of extant crocodylians is relatively recent (Benton & Clark, 1988). Prior to this, ‘Crocodilia’ comprised a far more inclusive and imprecisely defined group, including taxa from the Triassic, ~200 Ma (*e.g*. Hay, 1930; Mook, 1934; Sill, 1968). Indeed, the taxonomic content of ‘Crocodilia’ outlined by Mook (1934) approximately corresponds to Crocodyliformes in today’s phylogenetic nomenclature, *i.e*. Protosuchia + Mesoeucrocodylia (Benton & Clark, 1988; Sereno et al., 2001; Martin & Benton, 2008) (Fig. 1). Nevertheless, extant crocodylians have consistently been placed within Eusuchia, which was originally defined by Huxley (1875) as an apomorphy-based group for taxa with pterygoid-bound choanae and procoelous vertebrae. Eusuchia is now phylogenetically defined, and more exclusive, comprising the last common ancestor of *Hylaeochampsa vectiana* and Crocodylia, and all of its descendants (Brochu, 1999a). In turn, Eusuchia is a clade within a larger grouping, Neosuchia (Fig. 1), that is defined as all crocodyliforms more closely related to *Crocodylus niloticus* than to *Notosuchus terrestris* (Sereno et al., 2001).

**Figure 2:**
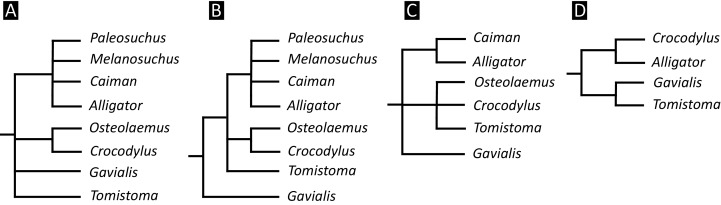
Cladograms outlining the interrelationships of extant crocodylians implied from the taxonomic classification schemes of earlier studies. (A) After Romer (1956) and Steel (1973); (B) after Kälin (1955) and Sill (1970); (C) after Mook (1934); and (D) after Hay (1930).

Over the last century, multiple pre-cladistic classification schemes of extant crocodylians have emerged based on traditional comparative anatomical data (Fig. 2). These classification schemes agree in several respects, for example in considering *Crocodylus* to be closely related to *Osteolaemus*, and recognising *Alligator* to group with caimanines (*i.e. Caiman*, *Melanosuchus*, and *Paleosuchus*) (*e.g*. Mook, 1934; Kälin, 1955; Romer, 1956; Sill, 1968; Steel, 1973). However, there has historically been disagreement over the affinities of the extant gharials (*Gavialis* and *Tomistoma*) with regards to other living crocodylians. Whereas Romer (1956) and Steel (1973) placed all living crocodylians within the same family (‘Crocodylidae’), Mook (1934), Kälin (1955), and Sill (1968) placed *Gavialis* within a separate family, indicating that it is more distantly related to all other extant crocodylians. Although some pre-cladistic classifications suggested a closer relationship between *Gavialis* and *Tomistoma* (*e.g*. Hay, 1930), the prevailing morphological hypothesis has been that *Gavialis* is distantly related to all other extant crocodylians.

**Figure 3:**
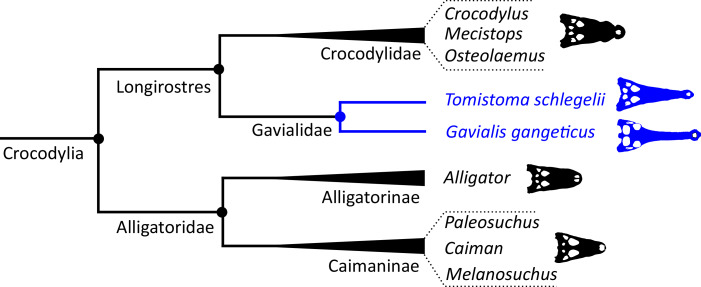
Simplified interrelationships of Crocodylia based on the phenogram generated by Densmore (1983).

Crocodylian systematics received a surge of interest in the 1980s. This was stimulated by the introduction of phylogenetic systematics (Hennig, 1965), technological advancements such as amino acid and DNA sequencing, new morphological character datasets, as well as computational advances in analysing morphological and molecular datasets. Another important driver of investigations into crocodylian interrelationships was continued debate over the affinities of the two extant gharials. Disagreement between traditional morphological hypotheses and new, molecular hypotheses would propel and shape the course of investigations. The earliest crocodylian cladogram based on biomolecular data was generated by Densmore (1983), who conducted a series of phenetic analyses based on blood proteins. By contrast to the morphological hypothesis, these analyses suggested a sister relationship between *Tomistoma schlegelii* and *Gavialis gangeticus*, with this clade (Gavialidae) more closely related to crocodylids (*Crocodylus* and *Osteolaemus*) than to alligatorids (Fig. 3). The crocodylian clade excluding Alligatoridae was later phylogenetic defined as Longirostres (Harshman et al., 2003). These protein distance data also supported a relatively recent divergence of *Crocodylus* in the Miocene (Brochu, 2000); in earlier studies, *Crocodylus* had often been considered an ancient taxon with representatives extending back to the Cretaceous (*e.g*. Marsh, 1872; Cope, 1882; Lydekker, 1886; Etheridge, 1917).

The emergence of a new hypothesis for crocodylian interrelationships prompted some authors to attempt to reconcile the morphological and molecular data. Buffetaut (1985b) outlined morphological similarities between *Gavialis*, *Crocodylus*, and *Tomistoma*. Furthermore, he argued that the fossil record was compatible with the molecular hypothesis, and that suitable candidates for a common ancestor of *Tomistoma* and *Gavialis* could be found in the Eocene of North Africa, such as ‘*Tomistoma*’ (=*Eogavialis*) *africanum*. By contrast, traditional comparative anatomical studies continued to support the early divergence of *Gavialis* from all other extant crocodylians, with evidence from osteoderm arrangement, braincase and appendicular anatomy, as well as differences in tail musculature (*e.g*. Ross & Mayer, 1983; Tarsitano, 1985; Tarsitano, Frey & Riess, 1989; Frey, Riess & Tarsitano, 1989). Furthermore, in parallel, new morphological character datasets were published that would further support the early divergence of *Gavialis* (*e.g*. Norell, 1988, 1989; Norell & Clark, 1990; Willis, 1993; Clark, 1994; Salisbury & Willis, 1996). Based on the morphology of outgroups such as the Early Cretaceous neosuchian *Bernissartia fagesii*, Norell (1989) argued that many of the proposed anatomical similarities between *Gavialis*, *Crocodylus*, and *Tomistoma* were plesiomorphic for Crocodylia, and simply lost in Alligatoridae.

Not all comparative anatomical evidence distinguishes *Gavialis* from other extant crocodylians. All extant *Crocodylus* species, *Osteolaemus*, *Tomistoma*, and *Gavialis* share adaptations for tolerating saltwater, to the exclusion of alligatorids. These include a keritanised buccal cavity and lingual osmoregulatory pores on the tongue (Taplin, Grigg & Beard, 1985; Taplin & Grigg, 1989). Although the latter are greatly reduced in *Gavialis* (which can be interpreted as a secondary adaptation to inhabiting freshwater environments), the presence of these pores suggests a closer affinity of *Gavialis* with *Tomistoma* and crocodylids, than to alligatorids (Taplin & Grigg, 1989). Reconsiderations of the biomolecular evidence, using more refined techniques, continued to support the sister relationship of *Gavialis* and *Tomistoma* (Hass et al., 1992). Furthermore, in the late 1980s to 1990s, new support for Longirostres emerged from the analyses of DNA sequences and restriction fragment length polymorphism matrices (Densmore & Owen, 1989; Densmore & White, 1991; Gatesy & Amato, 1992; Gatesy, DeSalle & Wheeler, 1993; Aggarwal et al., 1994). As such, morphological and molecular datasets continued to support contrasting hypotheses for crocodylian interrelationships.

**Figure 4:**
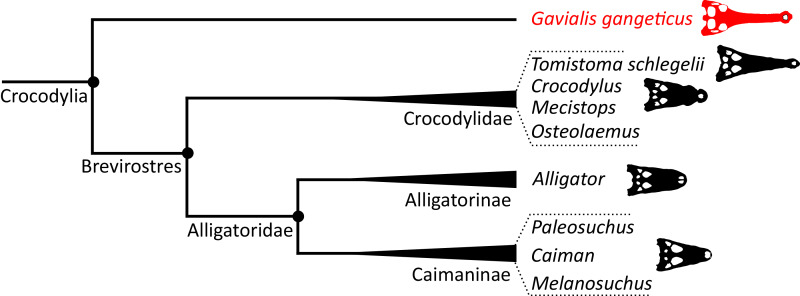
Simplified cladogram, illustrating the traditional morphological hypothesis of extant crocodylian interrelationships.

Poe (1997) presented a combined phylogenetic analysis of extant crocodylians, incorporating 64 morphological characters, restriction fragment characters, and mtDNA sequences. The resulting strict consensus tree was congruent with the typical molecular topology. Brochu (1997b) conducted a similar combined analysis. Using the same biomolecular data as Poe (1997), Brochu (1997b) included 164 morphological characters, which constituted the largest morphological character dataset applied in crocodylian systematics at the time. Two-thirds of these characters were new, with the remainder drawn from a synthesis of earlier studies (Benton & Clark, 1988; Norell, 1988, 1989; Norell & Clark, 1990; Buscalioni, Sanz & Casanovas, 1992; Willis, 1993; Clark, 1994). Brochu (1997b) conducted a series of analyses that used different combinations of extant and fossil taxa, as well as morphological and molecular data. *Gavialis* was consistently recovered as the sister taxon to all other extant crocodylians, in agreement with the traditional morphological hypothesis (Fig. 4); however, the position of *Tomistoma* depended on whether fossil ingroup taxa were included or excluded (Brochu, 1997b). The Brochu (1997b) dataset has formed the basis of essentially all morphological phylogenetic analyses of Crocodylia over the last two decades. Numerous studies have augmented it with newly described taxa and novel characters, resulting in revised phylogenetic hypotheses (*e.g*. Brochu, 1999a, 2004a, 2004b; Hua & Jouve, 2004; Salisbury et al., 2006; Brochu, 2010, 2011; Brochu & Storrs, 2012; Jouve et al., 2015; Salas-Gismondi et al., 2015; Jouve, 2016; Narváez et al., 2016; Salas-Gismondi et al., 2016; Cidade et al., 2017; Lee & Yates, 2018; Salas-Gismondi et al., 2019; Iijima & Kobayashi, 2019; Massonne et al., 2019; Groh et al., 2020; Nicholl et al., 2020; Rio et al., 2020; Ristevski et al., 2020, 2021; Blanco, 2021). Similarly, there has been a synchronous burst of molecular studies of Crocodylia, with phylogenetic analyses of several mitochondrial and nuclear genes (*e.g*. Harshman et al., 2003; Janke et al., 2005; McAliley et al., 2006; Ji et al., 2006; Roos, Aggarwal & Janke, 2007; Willis et al., 2007; Gatesy & Amato, 2008; Meganathan et al., 2010; Yan et al., 2010; Man et al., 2011; Meredith et al., 2011; Oaks, 2011; Bittencourt et al., 2019; Milián-García et al., 2020; Hekkala et al., 2021; Pan et al., 2021), as well as a whole genome analysis (Green et al., 2014). However, despite these developments, the *Gavialis*-*Tomistoma* morphology *versus* molecular dichotomy remains largely unresolved.

### Problems in crocodylian systematics

#### The gharial problem

**Figure 5:**
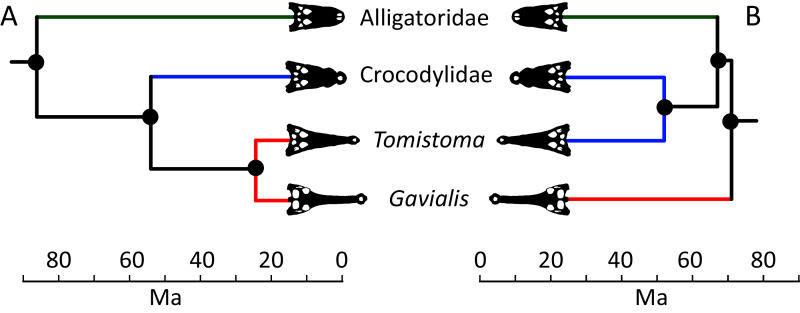
Contrasting topologies of molecular (A) and morphological (B) phylogenies illustrating differences in the divergence of principal crocodylian clades. Molecular divergences based on Oaks (2011) and morphological divergences based on the stratigraphically oldest fossils assigned to each clade prior to the current study.

**Figure 6:**
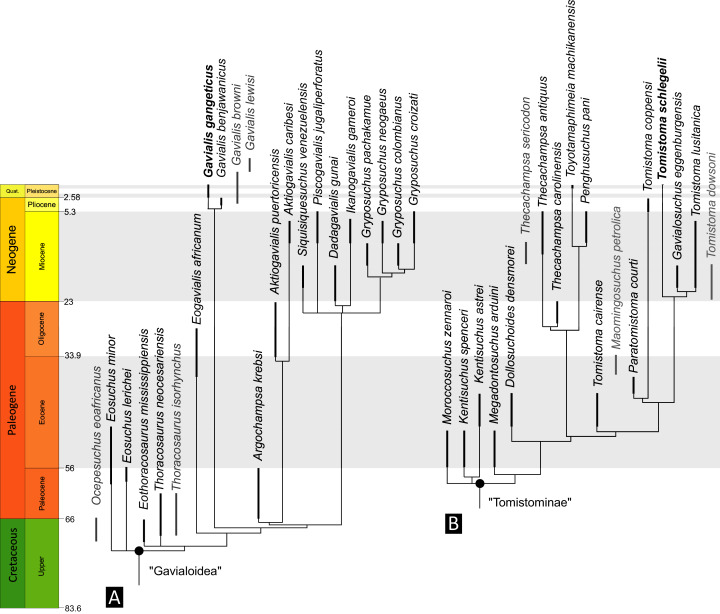
Time-calibrated phylogenies illustrating the stratigraphic distribution of (A) Gavialoidea after Salas-Gismondi et al. (2019), and (B) Tomistominae after Jouve (2016). Taxa in grey not included in aforementioned studies.

As outlined above, the phylogenetic position of *Gavialis gangeticus* in relation to other crocodylians is one of the most persistent problems in crocodylian systematics. It is also arguably the most significant problem, given that the contrasting hypotheses indicate substantial rearrangements of the crocodylian tree. Thus far, our discussion of the gharial problem has centred on topological differences; however, the incongruence has implications for the estimated divergence time of the two extant gharials too (Fig. 5). Molecular data indicate that *Tomistoma schlegelii* and *Gavialis gangeticus* last shared a common ancestor 31–18 Ma (Oaks, 2011; Pan et al., 2021). This estimate is at odds with fossil data, which indicate that taxa (Gavialoidea) more closely related to *Gavialis* than to *Tomistoma* first appeared by the latest Cretaceous (~80 Ma) (Brochu, 2004a). Harshman et al. (2003) commented that if some of these early appearing gavialoids are incorrectly assigned to this clade, then the temporal incongruence would become narrower. In particular, those authors referred to the early diverging taxon, *Thoracosaurus*, remains of which are present in the latest Cretaceous–early Paleogene of Europe and North America (Brochu, 2004a). However, in the subsequent years, several new gavialoid taxa have been described, such that it is not only one or two taxa that result in this incongruence. Indeed, there is now a relatively continuous fossil record of morphologically intermediate forms, bridging the gap between the earliest appearing fossil gavialoids and extant *Gavialis* (Fig. 6A), including *Eothoracosaurus* from the latest Cretaceous of North America (Brochu, 2004a), *Dolichochampsa* from the latest Cretaceous of South America (Jouve et al., 2021), *Ocepesuchus* from the latest Cretaceous of north Africa (Jouve et al., 2008), *Eosuchus* from the early Paleogene of Europe and North America (Delfino, Piras & Smith, 2005; Brochu, 2006), *Aktiogavialis* from the late Paleogene to early Neogene of the Caribbean (Vélez-Juarbe, Brochu & Santos, 2007; Salas-Gismondi et al., 2019), and *Eogavialis* from the late Paleogene to Neogene of Africa (Andrews, 1906; Hecht & Malone, 1972; Storrs, 2003). Similarly, there is an extensive, near-global fossil record of taxa referred to Tomistominae, spanning the early Eocene (~54 Ma) to the Pleistocene (Kobayashi et al., 2006; Brochu, 2007b; Piras et al., 2007; Jouve et al., 2015; Jouve, 2016; Nicholl et al., 2020; Ristevski et al., 2021) (Fig. 6B). As such, the morphological hypothesis for an early diverging Gavialoidea is highly congruent stratigraphically. Although this could of course be incorrect, it would require a rearrangement of evolutionary relationships among fossil crocodylians, which have been largely stable over the last two decades (*e.g*. Brochu, 2004a; Brochu et al., 2012; Jouve et al., 2015; Narváez et al., 2016; Salas-Gismondi et al., 2019).

With the wealth of new morphological character data (Brochu, 1997b), early investigations of the gharial problem explored whether there might be hidden support for the molecular hypothesis in the morphological data, *i.e*. a ‘secondary signal’ that supports a grouping of *Tomistoma* + *Gavialis* (Trueman, 1998; Gatesy et al., 2003; Harshman et al., 2003). Trueman (1998) used reverse successive weighting of morphological characters, *i.e*. down-weighting non-homoplastic characters, in order to allow the secondary signal (if present) to influence the topology (though see Brochu, 1999b). As a result, Trueman (1998) recovered the molecular topology and identified 12 morphological characters that supported this grouping. Using a simpler approach, Harshman et al. (2003) optimised Brochu’s (1997a) morphological characters on both the molecular and morphological trees. Harshman et al. (2003) characterised the secondary signal as those characters that had fewer steps on the molecular than the morphological tree, resulting in 17 characters that supported the molecular topology. Furthermore, Harshman et al. (2003) recognised that the construction of several morphological characters precluded tomistomines and gavialoids from sharing the same character states. Using alternative criteria and based on a slightly different set of characters, Gatesy et al. (2003) also recovered a secondary signal. As such, it has been demonstrated that there is support for the molecular hypothesis ‘hidden’ in the morphological data.

Several studies have also turned to combined morphological and molecular analyses to resolve the gharial problem, and these typically recover the molecular topology (Poe, 1997; Gatesy et al., 2003; Gold, Brochu & Norell, 2014; Lee & Yates, 2018; Iijima & Kobayashi, 2019; Hekkala et al., 2021). However, the results of combined analyses depend strongly on the proportions of different data types (*e.g*. Poe, 1997; Brochu, 1997b), and might simply reflect the data with the strongest signal (Brochu, 2003). Furthermore, combined analyses including fossil taxa continue to face the issue of temporal incongruence, given that the earliest appearing, latest Cretaceous gavialoids are still recovered within the crown gharial clade (Gatesy et al., 2003; Gold, Brochu & Norell, 2014; Iijima & Kobayashi, 2019). Lee & Yates (2018) introduced stratigraphic data (Bayesian tip-dating) into combined analyses, which resolved both the topological and temporal incongruence. Unlike previous combined analyses, all pre-Neogene gavialoids in their analysis were recovered outside of Crocodylia. Furthermore, several taxa usually considered as tomistomines were recovered in the stem of the crown gharial group instead, resulting in a topology largely consistent with the molecular divergence time of Gavialidae presented by Oaks (2011). Although the use of stratigraphic data in phylogenetic analyses is controversial (*e.g*. Smith, 2000; Alroy, 2002; Fisher, 2008), Lee & Yates (2018) study demonstrates that the fossil record can be stratigraphically congruent with the molecular hypothesis.

There are several arguments that support the acceptance of the molecular over the morphological topology. The molecular topology has withstood numerous independent analyses, including the use of multiple gene loci in mitochondrial and nuclear DNA (*e.g*. Harshman et al., 2003; Man et al., 2011; Oaks, 2011). By contrast, although morphological character datasets have been augmented with new characters, morphological characters are typically reused in subsequent iterations of a dataset, meaning that there is little real independence between analyses. It might also be argued that DNA sequence data have an advantage over morphological character data, since the delimitation between the four nucleotide bases is unequivocal. Although binary presence/absence characters might be simple to delimit in morphological datasets, the appropriate approach for complex, multistate characters is the subject of much debate (*e.g*. Wilkinson, 1995; Sereno, 2007; Brazeau, 2011).

Nevertheless, it is prudent to consider ways in which the molecular data could be misleading. A criticism of early biomolecular studies (*e.g*. Densmore, 1983) was that these were based on phenetic analyses and they lacked outgroup rooting (Norell, 1989). Additionally, in molecular phylogenetic analyses, the issue emerges that the closest living relatives of Crocodylia, Aves, is separated by extremely long branches of approximately 250 million years (Harshman et al., 2003). Using outgroups that are so distantly separated could result in spurious relationships (*e.g*. Wilberg, 2015). A common argument against the morphological datasets has been that they are strongly affected by convergence (Hass et al., 1992; Brochu, 1997b). However, molecular data can be misled by long-branch attraction, *i.e*. distantly related taxa with many convergently acquired genetic features can be incorrectly grouped together. It is possible then that *Tomistoma* and *Gavialis* truly belong to distantly related lineages as suggested by morphological data, but that their evolutionary (genetic) ancestry has essentially been erased by the accumulation of apomorphies. However, these criticisms have been addressed through: (1) the construction of molecular phylogenies rather than phenetics; and (2) the use of less convergence-prone, non-coding gene loci to ameliorate issues of long-branch attraction (Harshman et al., 2003).

As such, it appears difficult to reject the molecular topology, and thus it is the morphological data that appears to be problematic in terms of inferring the phylogenetic relationships of crocodylians. Indeed, several authors have suggested that the scrutiny of morphological characters might be critical in resolving the incongruence (*e.g*. Hass et al., 1992; Brochu, 1997b; Harshman et al., 2003). Accordingly, a few studies have begun to reassess the morphological character data. Sookias (2020) conducted a review of morphological characters applied in crocodylian systematics. Based on a sample of extant crocodylians, he found that the removal or revision of characters lacking a ‘robust’ construction resulted in a topology that is more concordant with the molecular hypothesis. Also, recent studies have demonstrated that a review of certain ‘important’ taxa could also be formative in the debate. Iijima & Kobayashi (2019) re-evaluated the anatomy of two species referred to Tomistominae from East Asia. They recognised several gavialoid atavisms in these taxa, as well as new characters that begin to bridge the morphological gap between the extant gharials. Most recently, Ristevski et al. (2020) recovered weak support for the molecular hypothesis in some of their trees based on analyses of morphological data. As such, it appears that the gharial problem might be tractable through increased character and taxon sampling, as well as improved character construction.

#### Other taxonomic problems

**Figure 7:**
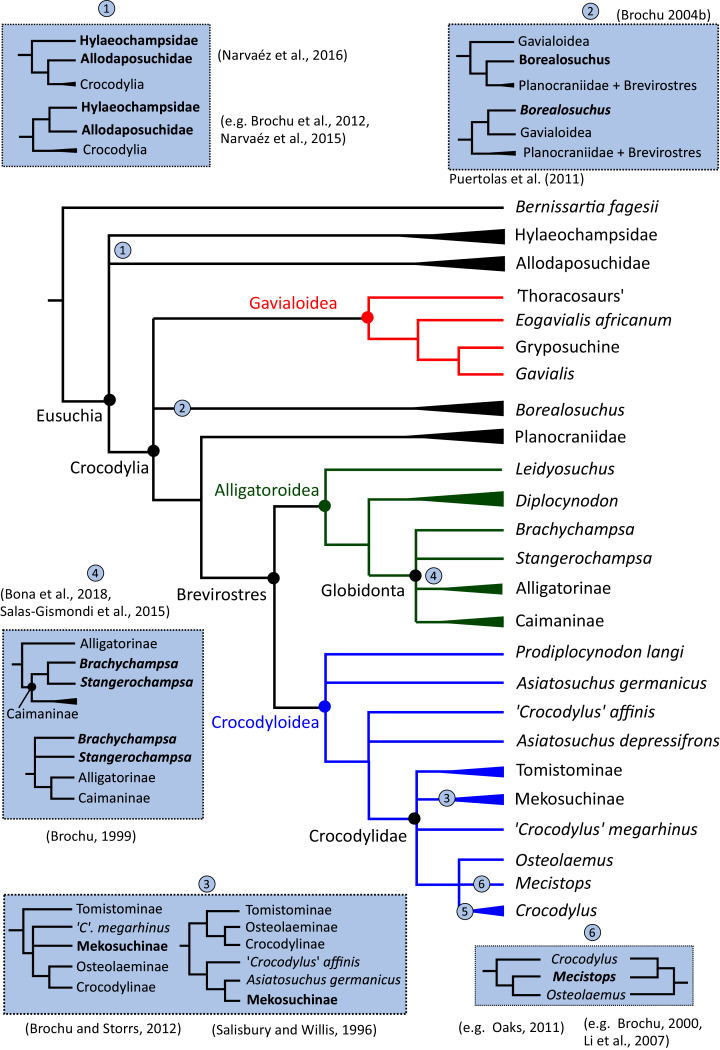
Simplified morphological phylogeny of Crocodylia based on Brochu et al. (2012), summarising some of the taxonomic problems discussed.

In addition to the gharial problem, there are a host of other unresolved issues in crocodylian systematics (Fig. 7), including: (1) the affinities of Allodaposuchidae, which has been recovered as an early diverging clade within Crocodylia (*e.g*. Blanco, 2021), the sister clade to Crocodylia (*e.g*. Narváez et al., 2016), or forming a grouping with Hylaeochampsidae (*e.g*. Brochu et al., 2012; Narváez et al., 2015); (2) the taxonomic content and biogeographic origin of Caimaninae, with several recent studies (*e.g*. Salas-Gismondi et al., 2015; Bona et al., 2018) recovering latest Cretaceous North American taxa as the earliest members of what is typically considered a South American clade (*e.g*. Brochu, 1999a; Bona, 2007); (3) the biogeographic origin and phylogenetic affinities of the endemic Australasian clade Mekosuchinae (*e.g*. Salisbury & Willis, 1996; Brochu & Storrs, 2012; Yates & Pledge, 2017; Lee & Yates, 2018; Ristevski et al., 2020); (4) whether *Mecistops* is more closely related to *Crocodylus* (*e.g*. Brochu, 2000, 2007a; Brochu et al., 2010; Poe, 1997; McAliley et al., 2006; Li et al., 2007) or *Osteolaemus* (*e.g*. Gatesy et al., 2003; Schmitz et al., 2003; Willis, 2009; Oaks, 2011; Man et al., 2011; Lee & Yates, 2018; Pan et al., 2021; Hekkala et al., 2021); and (5) the species interrelationships of the crown genus *Crocodylus*, as well as the resulting biogeographic implications (*e.g*. Meganathan et al., 2010; Meredith et al., 2011; Oaks, 2011; Nicolaï & Matzke, 2019; Delfino et al., 2020, 2021).

As noted above, nearly all morphological analyses of crocodylian interrelationships have been based on the data matrix of Brochu (1997b), which was originally designed to evaluate the relationships of approximately 60 crocodylian species. Although iterations of this dataset have been augmented with new characters and taxa, most studies have made only small modifications in an attempt to improve resolution in specific parts of the tree, without a critical re-examination of the whole dataset. Additionally, the coding and treatment of morphological characters has been conservative in crocodylian systematics. For example, only in recent studies has the ordering of multistate characters been implemented (Lee & Yates, 2018; Iijima & Kobayashi, 2019; Groh et al., 2020; Nicholl et al., 2020; Rio et al., 2020; Ristevski et al., 2020, 2021). Fewer studies still have explored the impact of different styles of character construction (*e.g*. reductive coding) and the use of different character weighting strategies (Groh et al., 2020; Nicholl et al., 2020; Rio et al., 2020; Ristevski et al., 2020, 2021; see also Johnson, Young & Brusatte, 2020). Furthermore, there have been relatively few attempts to introduce novel sources of data into phylogenetic analyses of Crocodylia, such as morphometric data (Gold, Brochu & Norell, 2014; Bona et al., 2018) and continuous characters (Groh et al., 2020).

In this study, we present a new morphological character list and dataset for Crocodylia, based on an extensive review of the literature and first-hand examination of specimens. We provide a discussion of modifications made to existing morphological characters, as well as comprehensive illustrations of character states to improve repeatability in future analyses. We analyse this phylogenetic dataset within a parsimony framework, testing the effects of different treatments of quantitative data and weighting strategies. We present new topologies for crocodylian interrelationships, test several competing hypotheses pertaining to problematic taxa, and provide a revised view of the evolutionary and biogeographic history of Crocodylia.

## Methods

### Taxon sampling

Our new dataset consists of 144 operational taxonomic units (OTUs), 119 of which were studied first-hand by the lead author. Character scoring for the remaining 25 OTUs was based on descriptions and figures in the literature, supplemented by photographs from colleagues. The choice of outgroup can have a significant effect on topology, as demonstrated previously for crocodyliforms (Wilberg, 2015; Sookias, 2020). As such, 12 of the OTUs consist of taxa that lie outside of the crocodylian radiation, comprising: *Bernissartia fagesii* (the designated outgroup taxon), *Isisfordia duncani*, *Theriosuchus pusillus*, three paralligatorids (the ‘Glen Rose Form’, *Wannchampsus kirpachi*, and *Shamosuchus djadochtaensis*), three hylaeochampsids (*Hylaeochampsa vectiana*, *Iharkutosuchus makadii*, and *Acynodon iberoccitanus*), and three allodaposuchids (*Allodaposuchus precedens*, *Agaresuchus fontisensis*, and *Lohuecosuchus megadontos* [note that these have all recently been referred to *Allodaposuchus* by Blanco (2021)). A full list of OTUs, including which specimens and publications were used for scoring, is provided in Appendix 1. A table of crocodylian clade names along with their definitions is provided in Table 1.

**Table 1:** Definitions of clade names, ordered systematically.

Taxon name	Definition	Taxon definition
Eusuchia (Huxley, 1875)	The last common ancestor of *Hylaeochampsa vectiana*, *Alligator mississippiensis*, *Crocodylus niloticus*, *Gavialis gangeticus* and all of its descendants.	Brochu (1999a)
Hylaeochampsidae (Andrews, 1913)	The last common ancestor of *Hylaeochampsa vectiana*, *Iharkutosuchus makadii*, *Pachycheilosuchus trinquei* and *Pietraroiasuchus ormezzanoi* and all of its descendants.	Buscalioni et al. (2011)
Allodaposuchidae (Narváez et al., 2015)	*Allodaposuchus precedens* and all crocodyliforms more closely related to it than to *Hylaeochampsa vectiana*, *Shamosuchus djadochtaensis*, *Borealosuchus sternbergii*, *Planocrania datangensis*, *Alligator mississippiensis*, *Crocodylus niloticus*, or *Gavialis gangeticus*.	Narváez et al. (2015)
Crocodylia (Gmelin, 1789)	The last common ancestor of *Alligator mississippiensis*, *Crocodylus niloticus* and *Gavialis gangeticus*, and all of its descendants.	Benton & Clark (1988)
Alligatoroidea (Gray, 1844)	*Alligator mississippiensis* and all crocodylians more closely related to it than to *Crocodylus niloticus* and *Gavialis gangeticus*	Norell, Clark & Hutchison (1994)
Diplocynodontinae (Brochu, 1999a)	*Diplocynodon ratelii* and all crocodylians more closely related to it than to *Alligator mississippiensis*	Brochu (1999a)
Globidonta (Brochu, 1999a)	*Alligator mississippiensis* and all crocodylians more closely related to it than to *Diplocynodon ratelii*	Brochu (1999a)
Alligatoridae (Cuvier, 1807)	The last common ancestor of *Alligator mississippiensis* and *Caiman crocodilus* and all of its descendants	Norell, Clark & Hutchison (1994)
Alligatorinae (Kälin, 1939)	*Alligator mississippiensis* and all crocodylians more closely related to it than to *Caiman crocodilus*	Brochu (1999a)
Caimaninae (Norell, 1988)	*Caiman crocodilus* and all crocodylians more closely related to it than to *Alligator mississippiensis*	Brochu (1999a)
Jacarea (Norell, 1988)	The last common ancestor of *Caiman latirostris*, *Caiman crocodilus*, *Caiman yacare*, *Melanosuchus niger*, and all its descendants	Brochu (1999a)
Planocraniidae (Li, 1976)	The last common ancestor of *Planocrania hengdongensis*, *Planocrania datangensis*, *Boverisuchus vorax*, *Boverisuchus magnifrons*, and all of its descendants	Brochu (2012)
Longirostres (Cuvier, 1807)	The last common ancestor of *Crocodylus niloticus*, *Gavialis gangeticus*, and all of its descendants	Harshman et al. (2003)
Crocodyloidea (Fitzinger, 1826)	*Crocodylus niloticus*, and all crocodylians more closely related to it than to *Alligator mississippiensis* or *Gavialis gangeticus*	Norell, Clark & Hutchison (1994)
Crocodylidae (Cuvier, 1807)	The last common ancestor of *Crocodylus niloticus*, *Osteolaemus tetraspis* and all of its descendants	Norell, Clark & Hutchison (1994); Brochu (2003)
Osteolaeminae (Brochu, 2003)	*Osteolaemus tetraspis* and all crocodylians more closely related to it than to *Crocodylus niloticus*	Brochu (2003)
Crocodylinae (Cuvier, 1807)	*Crocodylus niloticus*, and all crocodylians more closely related to it than to *Osteolaemus tetraspis*	Brochu (1999a)
Mekosuchinae (Balouet & Buffetaut, 1987)	The last common ancestor of *Kambara implexidens* and *Mekosuchus inexpectatus*, and all of its descendants	Salisbury & Willis (1996) and Brochu (2003)
Gavialoidea (Hay, 1930)	*Gavialis gangeticus*, and all crocodylians more closely related to it than to *Alligator mississippiensis* and *Crocodylus niloticus*	Norell, Clark & Hutchison (1994)
Gavialidae (Hay, 1930)	The last common ancestor of *Gavialis gangeticus*, *Tomistoma schlegelii*, and all of its descendants	Norell, Clark & Hutchison (1994)
Tomistominae (Kälin, 1955)	*Tomistoma schlegelii* and all crocodylians more closely related to it than to *Gavialis gangeticus*	Brochu (2003)
Gavialinae (Nopcsa, 1923)	*Gavialis gangeticus* and all crocodylians more closely related to it than to *Tomistoma schlegelii*	Brochu (2003)
Gryposuchinae (Vélez-Juarbe, Brochu & Santos, 2007)	*Gryposuchus jessei* and all crocodylians more closely related to it than to *Gavialis gangeticus* and *Tomistoma schlegelii*.	Vélez-Juarbe, Brochu & Santos (2007)
Brevirostres (Zittel, 1890)	*The last common ancestor of Alligatoroidea and Crocodyloidea* and all of its descendants	Brochu (1999a)

### Character list construction

An initial character list was constructed by assimilating all existing morphological characters from previously published studies of crocodylian systematics. The largest single source of characters was the dataset of Brochu (1997b), which contains a substantial number of novel characters, as well as characters modified from earlier studies (Benton & Clark, 1988; Norell, 1988; Norell & Clark, 1990; Clark, 1994). Significant contributions of new characters were later introduced by Hua & Jouve (2004), Jouve et al. (2015), Salas-Gismondi et al. (2015, 2016, 2019), Lee & Yates (2018), and Iijima & Kobayashi (2019). A large number of additional studies also introduced crocodylian characters in fewer numbers; the origin of these and all other characters is described in detail in the character list.

All characters were sorted anatomically and checked for redundancy. When overlapping characters were found, the original contribution was retained and accepted modifications were cited. All attempts were made to understand existing characters. This included scoring characters alongside specimens in museum collections, and checking which taxa were scored for particular character states in a large sample of published character taxon matrices (Brochu, 1999a; Buscalioni et al., 2001; Salisbury et al., 2006; Brochu, 2007a; Ősi, Clark & Weishampel, 2007; Brochu, 2011; Brochu et al., 2012; Brochu & Storrs, 2012; Salas-Gismondi et al., 2016; Cidade et al., 2017; Cossette & Brochu, 2018; Lee & Yates, 2018; Souza-Filho et al., 2018; Iijima & Kobayashi, 2019; Salas-Gismondi et al., 2019). Similar to the approach of Sookias (2020), these methods provided a means of assessing the ‘robustness’ of characters. Where inconsistencies emerged between character formulations and personal observations of specimens, characters were revised by the addition, removal, and/or modification of character states, as well as the introduction of new characters when necessary. All such changes are described in detail in the character list.

In total, 162 characters from previous studies of crocodylian systematics were omitted from the dataset for one of several reasons: (1) they were autapomorphies, and therefore phylogenetically uninformative; (2) they were accidental duplicates, being redundant with another morphological character; (3) the morphological variation that was described was ambiguous, and/or not visible in specimens studied first hand; (4) the character varied intraspecifically in most or all taxa studied. All discarded characters, their origin, and reason for removal, are listed in Table S1. A total of 45 characters are new to this study: 26 of these are based on personal observations in museum collections, and the remainder are based on a survey of the literature. The complete dataset comprises 330 morphological characters. 95% of characters are illustrated to create the most complete atlas of morphological characters published for crocodylian systematics.

### Character construction

#### Discrete morphological characters

All new characters and modifications to existing characters were constructed following the protocols of Sereno (2007) and Brazeau (2011) and as recently used for crocodyliforms by Tennant, Mannion & Upchurch (2016) and Groh et al. (2020). The most important aspects of character construction are detailed below:

1. Multistate characters that combine an ‘absent’ state plus two or more ‘present but variable’ states were reductively coded into two or more characters. For example, Brochu’s (1997b) character 152 is: “Internal choana not septate (0) or with septum that remains recessed within choana (1) or with septum that projects out of choana (2)”. Here, this character was reductively coded into two characters: (a) “Choanae, septum: present (0); absent (1)” (C193 in our study) and (b) “Choanae, external projection of the septum: absent, septum remains recessed within choanae (0); present, septum approaches external margin of choanae (1)” (C194 in our study). Reductively coding characters such as this has the benefit of capturing the grouping information in the presence or absence of a feature (Brazeau, 2011). The disadvantage is that parsimony algorithms treat inapplicable data in the same way as missing data. As such, the parsimony algorithm will optimise character (b) in taxa that lack a choanal septum, influencing the parsimony scores of trees and possibly leading to spurious groupings of taxa (Strong & Lipscomb, 1999; Brazeau, 2011). The latter can be alleviated if zero-length branches are set to collapse (as is the default in TNT). Furthermore, in the following discussion, the phylogenetic results are explored in detail, including the optimisation of several reductively coded characters, allowing the assessment of characters supporting particular nodes.
2. Compound characters, which describe variation in two or more non-homologous morphological features, were separated into two or more characters. For example, Jouve et al. (2015)’s character 43 is: “Splenial participates in mandibular symphysis and splenial symphysis adjacent to no more than one dentary alveolus (0); splenial excluded from mandibular symphysis and anterior tip of splenial passes ventral to Meckelian groove (1); splenial excluded from mandibular symphysis and anterior tip of splenial passes dorsal to Meckelian groove (2); participates in the mandibular symphysis over the length of two to five teeth (3); deep splenial symphysis, participates in the mandibular symphysis over the length of five to seven teeth, and forms wide ‘V’ within symphysis (4); or deep splenial symphysis participates in the mandibular symphysis over the length of five to seven teeth, and splenial constricted within symphysis and forms narrow ‘V’ (5); or deep splenial symphysis, longer than seven dentary alveoli (6)”. Here, this character is split into four separate morphological characters: (i) one that describes the presence or absence of contact of the splenial in the dentary symphysis (C222); (ii) one that describes the position of the anterior splenial tip relative to the Meckelian groove (C223); (iii) one that describes the length of participation of the splenial in the symphysis (C224); and (iv) one that describes the shape of the splenial within the symphysis as either wide or narrow (C225).

#### Continuous morphological characters

Morphological characters used in phylogenetic analyses often describe variation that is quantitative, for example describing the relative sizes of processes, lengths of sutural contacts, or counts of teeth and vertebrae (Rae, 1998; Wiens, 2001). Commonly, such quantitative features are discretely delimited using terminology such as ‘large’, ‘small’, and ‘poorly developed’. This kind of terminology is very common in crocodylian systematics. For example, the character list of Brochu (1997b (characters 83, 110, and 111, respectively]; italics added by authors of present study) includes: “Quadratojugal sends *long* anterior process along lower temporal bar (0) or sends *modest* process, or none at all, along lower temporal bar (1)”, “Palatine process extends (0) or does not extend (1) *significantly* beyond the anterior end of the suborbital fenestra”, and “Maxillary foramen for palatine ramus of CN-V *small* or not present (0) or *very large* (1).”

Such terminology is problematic, given the subjective nature of determining whether a feature is ‘large’ or ‘small’, etc. Although the original author/s usually have a clear idea of how the states are divided from one another, this has ramifications for repeatability and consistency, particularly when other authors add taxa to a matrix, who might have a very different concept of what connotes a ‘large’ or ‘small’ feature. A partial remedy to this problem is through the delimitation of quantitative character states by threshold values. In theory, these thresholds should represent discontinuities in measured values of all the taxa included in an analysis; however, since such data are seldom presented, it is not always clear how previous character states are delimited. Examples in crocodylian systematics include Jouve et al. (2015)’s character 237 (“Pterygoid at least 50% wider than its minimal length (0) or nearly as wide as its minimal length (1)”) and Lee & Yates (2018) character 217 (“Elongation of the retroarticular process: length at least 1.5 times the maximum width (0), or less than 1.5 times the maximum width (1)”).

A number of methods have been developed to delimit quantitative variation into discrete character states, such as gap-coding (Mickevich & Johnson, 1976), gap weighting (Thiele, 1993), and step-matrix gap weighting (Wiens, 2001). These methods use different statistical criteria for delimiting continuous variation; however, a common concern is that taxa with significantly different values may be assigned to the same state, whereas taxa with non-significant differences can be assigned to different states (Farris, 1990; Goloboff, Mattoni & Quinteros, 2006). Additionally, Garcia-Cruz & Sosa (2006) demonstrated that alternative methods of character discretisation applied to the same dataset can result in significant differences in phylogenetic results.

Goloboff, Mattoni & Quinteros (2006) introduced a procedure that enables continuous data to be included directly in a phylogenetic analysis, eliminating the need for prior discretisation. Nevertheless, the use of continuous characters is considered controversial by some authors. Arguments against the use of continuous characters include the potential for greater homoplasy, the artificial grouping of taxa based on phenetic data, the arbitrary choices of measurements, and character redundancy (*e.g*. Cox & Urbatsch, 1990; Stevens, 1991; Brocklehurst, Romano & Fröbisch, 2016). Despite these criticisms, many of which are also applicable to discrete morphological characters and can be mitigated, continuous characters have been found to contain useful phylogenetic information in numerous studies across a broad suite of taxonomic groups (*e.g*. Goloboff, Mattoni & Quinteros, 2006; Hornung-Leoni & Sosa, 2008; Mannion et al., 2013; Parins-Fukuchi, 2017; Randle & Sansom, 2017; Jones & Butler, 2018; Groh et al., 2020). An important consideration when using continuous characters is how extensively they should be applied. For example, Wiens (2001) implied that all morphological characters are best treated continuously. However, as commented upon by Goloboff, Mattoni & Quinteros (2006), this is not practical, and characters showing well-defined, discrete variation should be coded as such.

When characters are treated continuously in software such as TNT (Goloboff, Farris & Nixon, 2008), the absolute difference between one character value and another is used to calculate the cost of character state transformations, with up to 3 decimal places considered (Brocklehurst, Romano & Fröbisch, 2016). As such, the cost of a transformation between a condition in one species to the condition in another is proportional to the magnitude of that difference. Difficulty arises, however, when trying to determine the relative cost of transformations between characters that vary on different orders of magnitude (Goloboff, Mattoni & Quinteros, 2006; Koch, Soto & Ramírez, 2015). This occurs in continuous character datasets because it is possible to combine meristic characters, ratios of measurements, and characters measured using different units. This raises the need to scale continuous characters, *i.e*. to adjust the cost of transformations between characters (Goloboff, Mattoni & Quinteros, 2006).

This problem can be illustrated by considering the following two continuous characters implemented in this study: (a) character 12, incisive foramen size, ratio of maximum mediolateral width of incisive foramen to the mediolateral width of the rostrum at the premaxilla-maxilla suture (after Brochu, 1999a (C124); Jouve et al., 2008 (C124); Groh et al., 2020 (C5)); and (b) character 21: scapular blade, anteroposterior flare of dorsal end at maturity: angle between anterior and posterior margins (after Benton & Clark, 1988; Brochu, 1997a (C22)). Whereas character 12 is a ratio of two linear measurements with a total range of 0.4, character 21 is an angular measurement with a range of 65, *i.e*. two orders of magnitude larger. Left unscaled, characters expressed in larger orders of magnitude (such as character 21) will exert a greater influence in determining the optimal topology than other characters (Mannion et al., 2013; Koch, Soto & Ramírez, 2015). In this case, the weight of character 21 is approximately 150 times greater than that of character 12. It therefore follows that characters varying on larger orders of magnitude need to be scaled down relative to characters that vary on smaller orders of magnitude.

Implied weighting (see below) was proposed as a way to decrease the problem of scaling. Measures of homoplasy will be greater in characters that vary on a larger scale, and those characters would be down-weighted when implied weighting is implemented (Goloboff, Mattoni & Quinteros, 2006). Other authors have re-scaled continuous characters to unity, *i.e*. making the total range of a continuous character equal to one step of a discretely coded character (Escapa & Catalano, 2013; Koch, Soto & Ramírez, 2015; Groh et al., 2020). Empirical evidence demonstrates that while both methods diminish the issue of scaling, implied weights only does so partially, and higher values of group support result from first re-scaling continuous characters (Koch, Soto & Ramírez, 2015). In this study, continuous characters were re-scaled by taking the reciprocal of the total range of the continuous character (*x*). Since character weights cannot be non-integers in TNT, this value was multiplied by 100:

$$(1/x) \times 100$$

A ‘side-effect’ of multiplying character weights by 100 is that tree lengths in all analyses implementing continuous characters are two orders of magnitude higher than all other analyses.

#### Re-discretised morphological characters

Some of the continuous characters used in this analysis are derived from discrete morphological characters. In order to test the impact of scoring these continuously, quantitative characters were also scored discretely. This was achieved by re-discretising continuous characters based on the measured values. The threshold values used to delimit the re-discretised character states were based on the original character description, to allow comparisons between this and earlier studies. If no threshold was given, or if the character was new, the state boundaries were determined by plotting the continuous character values from smallest to highest and seeking clear discontinuities in the data (Figs. S1, S2). Histograms for all continuous characters were also plotted to identify distribution patterns that might guide the delimitation of the continuous data (Figs. S3, S4). The Shapiro-Wilk test for normality was also implemented in R version 3.5.1 (R Core Team, 2018) for each set of measurements. For example, if the continuous values were found to contain multiple modes (*e.g*. if they were bimodal), then these modes might serve as the thresholds for delimiting character states. Difficulty arises when no discontinuity exists in the data. In such cases, the boundary was drawn using an anatomically ‘sensible’ and phylogenetically informative value. For example, there was no existing cut-off value for delimiting variation in the expansion of the ischial blade relative to the ischial length (Character 26), nor is there a discontinuity in the data. As such, the boundary between character states was drawn at 0.5, *i.e*. half the ischium length. This divided the measured values equally, whilst remaining intuitive when scoring the character.

### Extended implied weighting

It has been argued that characters that are highly homoplastic are less useful in determining phylogenetic relationships than characters exhibiting little homoplasy (Farris, 1969; Goloboff, 1993). Indeed, down-weighting homoplastic characters has been shown to increase phylogenetic accuracy in both simulations and with morphological and molecular datasets (Chippindale & Wiens, 1994; Goloboff et al., 2008; Goloboff, 2014; Goloboff, Torres & Arias, 2018; Groh et al., 2020). Goloboff (1993) introduced a novel approach to weighting homoplastic characters, ‘implied weights’, which weights characters during a tree search. A weight is calculated for each character depending on its fit to a given tree using the following formula:

$${\rm weight} = k / (h + k)$$

where ‘*k*’ is a constant defined in advance that controls the severity of the weighting function (with lower *k*-values resulting in more severe down-weighting), and ‘*h*’ is a measure of a character’s homoplasy. The sum of weighted character scores is calculated for each tree recovered during the tree search, with searches attempting to find trees that maximise these scores (Goloboff, 2014). Missing data can negatively influence traditional implied weighting. This occurs because homoplastic characters that are only scored in a few taxa are not down-weighted when convergent taxa are grouped together. As such, a modification to the algorithm was introduced by Goloboff (2014)—‘extended implied weighting’ (EIW)—which is better able to cope with missing data, and this approach is applied here. Since the choice of *k*-value has a strong impact on results, multiple *k*-values should be tested, with higher values especially appropriate for larger datasets (Goloboff et al., 2008; Goloboff, Torres & Arias, 2018; O’Reilly et al., 2016; Groh et al., 2020; Tschopp & Upchurch, 2019). Here, *k*-values of 3 and 12 are utilised.

### Phylogenetic analyses

Three sets of analyses were performed, each with a different treatment of quantitative data (Table 2): (1) quantitative characters treated continuously; (2) quantitative characters treated discretely; and (3) quantitative characters omitted. Within each set, Parsimony analyses were conducted under: (i) equal weighting; (ii) EIW with a *k*-value of 3 (EIW3), and (iii) EIW with a *k*-value of 12 (EIW12).

**Table 2:** Summary of phylogenetic analyses.

Analysis	Characters	Weighting	No. characters
1.1	Continuous and discrete	Equal	330
1.2	Continuous and discrete	EIW, *k* = 3	330
1.3	Continuous and discrete	EIW, *k* = 12	330
2.1	Re-discretised	Equal	330
2.2	Re-discretised	EIW, *k* = 3	330
2.3	Re-discretised	EIW, *k* = 12	330
3.1	Quantitative characters excluded	Equal	304
3.2	Quantitative characters excluded	EIW, *k* = 3	304
3.3	Quantitative characters excluded	EIW, *k* = 12	304

All analyses were performed using the New Technology Search in TNT (Goloboff, Farris & Nixon, 2008; Goloboff & Catalano, 2016), with all algorithms enabled and the consensus tree stabilized five times with a factor of 75. Trees recovered from the first iteration were used as starting trees for a traditional search using tree bisection and reconnection. With the exception of some recent studies (Lee & Yates, 2018; Iijima & Kobayashi, 2019; Groh et al., 2020; Nicholl et al., 2020; Rio et al., 2020; Ristevski et al., 2020, 2021), most analyses of crocodylian systematics have not ordered multistate characters. Although there has been much debate on the use of ordered characters, simulations demonstrate that ordered characters increase resolution (*e.g*. Grand et al., 2013). Here, 36 multistate characters were treated as ordered (characters 17, 37, 47, 48, 58, 65, 72, 75, 78, 81, 87, 88, 102, 109, 110, 137, 142, 151, 162, 175, 181, 188, 210, 214, 220, 221, 222, 224, 235, 243, 284, 293, 297, 308, 323, and 324), given that they represent a clear transformational series (Brazeau, 2011). The continuous + discrete and discrete-only datasets are presented in Supplemental Files 1 and 2 respectively.

### Measures of phylogenetic support

The consensus of optimal trees from analyses using continuous characters and extended implied weighting tends to be more resolved than trees obtained from discrete characters and equal weighted analyses. This is because taxa seldom share identical scores for continuous characters (values of which can have up to three decimal places in our study), resulting in fewer ties in tree lengths. Similarly, fewer ties between most parsimonious trees (MPTs) are recovered under EIW because the differential weighting of characters based on their homoplasy results in tree lengths with non-integer values. Consequently, very well resolved, but poorly supported, clades can be encountered, such that measures of internal accuracy become even more important. A total of nine phylogenetic analyses were conducted in this study, each producing a series of MPTs. After each analysis, support was assessed by resampling using the Jackknife and Bootstrap scripts provided in TNT, each with 100 replicates to reduce computational time across the nine analyses. Support was also assessed using the Bremer decay index. Differences in the relative step-length of characters after rescaling and extended implied weighting can obscure Bremer support values. These become non-integers, and their order of magnitude changes between analyses using a different *k*-value. This makes the practice of collapsing nodes with a Bremer support value of less than one step difficult. To account for this, the average step length of a character after weighting was calculated for each analysis, similar to the approach of Jones & Butler (2018). This was achieved by dividing tree length by the sum of all character transformations. Nodes with a Bremer support below the weighting-adjusted average step length were collapsed, and the remaining nodes were counted for each analysis. The consistency index (CI) and retention index (RI) were also calculated using the Stats.run script provided in TNT. Individual character CI and RI values were calculated using the Charstats.run script made available by Martin Ramírez (https://sites.google.com/site/teosiste/tp/archivos).

### Stratigraphic congruence

Bremer support, Jackknife, and Bootstrap each provide a measure of a tree’s internal accuracy, which does not necessarily mean the topology is correct. As such, stratigraphic congruence was used as an independent measure of validity following previous studies (*e.g*. Jones & Butler, 2018; Groh et al., 2020). Stratigraphic congruence was calculated for each set of MPTs in R version 3.5.1 (R Core Team, 2018) using the ‘StratPhyloCongruence’ command in the strap package (Bell & Lloyd, 2015). Taxon age ranges were extracted from Mannion et al. (2019) and updated following a review of the literature (Table S2). The strap package implements four measures of stratigraphic congruence. Firstly, the Stratigraphic Consistency Index (SCI) is the ratio of the stratigraphically ‘consistent’ internal nodes to the total number of nodes (Huelsenbeck, 1994). To be considered ‘consistent’, the first appearance datum of a node’s descendants must be equal to or younger than its sister node. Secondly, the Relative Completeness Index (RCI) is defined by the ratio of the minimum implied ghost ranges of a time calibrated tree, to the sum of the stratigraphic ranges of all taxa (Benton & Storrs, 1994). Thus, it effectively describes the proportion of time in a phylogenetic tree that is occupied by known taxon ranges. Thirdly, the ‘modified’ Manhattan Stratigraphic Measure (MSM*) works by optimising the differences in first appearance ages of taxa as a Sankoff character in a target tree, and calculating its total length (Siddall, 1998; Pol & Norell, 2001). The ratio of target tree length is taken to the minimum possible tree length (a tree in which the youngest appearing taxa are deeply nested, and older taxa are successively nested towards the root). Fourthly, the Gap Excess Ratio (GER) operates by defining optimal and suboptimal hypothetical trees based on the maximum and minimum possible sum of ghost ranges, respectively (Wills, 1999). Stratigraphic congruence is then based on the proportion of ghost ranges in a target tree compared to the suboptimal and optimal trees. The significance of each congruence measure was tested using the ‘StratPhyloCongruence’ command, using 1,000 permutations each for randomly generated and resampled trees.

### Constrained searches

Constrained searches were performed using the ‘Force’ command in TNT to compare the topologies of each analysis with existing hypotheses, *e.g*. the alternative positions of *Gavialis* in topologies resulting from analyses of morphological *versus* molecular data. The significance of tree length increase was assessed using the Templeton test with the TNT script ‘templetontest.run’ provided by Alexander Schimdt-Lebuhn (http://phylo.wikidot.com/tntwiki). A full list of constraints applied in our analyses can be found in Table S3 along with commands for implementation in TNT in Supplemental File 3.

### Categorisation of synapomorphies

**Figure 8:**
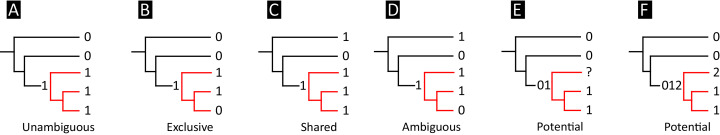
A summary of terms used to categorise synapomorphies. Red branches highlight the ingroup.

Where synapomorphies are discussed, they are categorised following the protocol in Tschopp, Mateus & Benson (2015). Unambiguous synapomorphies are present in all ingroup taxa, but no taxa (in this dataset) outside of the ingroup (Fig. 8A). Exclusive synapomorphies occur in some but not all ingroup taxa, but not in any taxon outside of the ingroup (Fig. 8B). Shared synapomorphies are present in all ingroup taxa and occur in some taxa outside of the ingroup (Fig. 8C). Ambiguous synapomorphies occur in some but not all ingroup taxa, and also occur outside of the ingroup (Fig. 8D). ‘Potential synapomorphies’ is a new category added here, describing character states which could be diagnostic of the ingroup. When optimising synapomorphies in TNT, these potential synapomorphies are not listed because of uncertainty in the condition of taxa immediately in the stem of the ingroup (Fig. 8E), or because closely related taxa and immediate outgroups exhibit different conditions (Fig. 8F).

## Results

### Overall topological results

**Figure 9:**
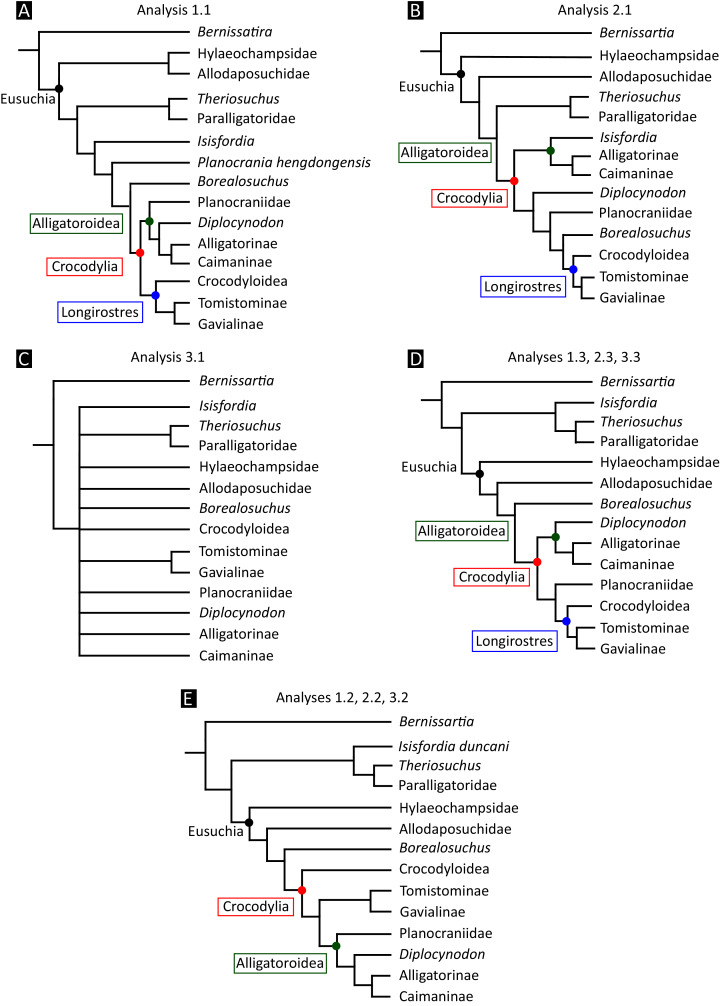
Summary of phylogenetic results from all nine analyses.

All analyses produced informative results, with the exception of Analysis 3.1 (quantitative characters excluded, equal weights), which resulted in >400,000 MPTs, recovering a large polytomy between most in-group taxa (Fig. 9C; see also Fig. S1). Nevertheless, in all analyses (including Analysis 3.1), *Gavialis gangeticus* is recovered as the closest living relative of *Tomistoma schlegelii*, together defining the crown gharial clade Gavialidae. Results of the eight informative analyses can be divided into two topological categories that tend to correlate with weighting strategy. The first set comprises all equal weighted analyses (1.1 and 2.1, but not the unresolved 3.1) (Figs. 9A, 9B) and all analyses using EIW12 (analyses 1.3, 2.3, and 3.3) (Fig. 9D). In the resultant topologies from these analyses, Crocodylia comprises three lineages: (Alligatoroidea + (Crocodyloidea + Gavialoidea)). Crocodyloidea is shorn of morphological tomistomines that are now recovered within Gavialoidea. Together, Gavialoidea and Crocodyloidea comprise Longirostres, which is the sister clade to Alligatoroidea (Harshman et al., 2003). The second category comprises all analyses under EIW3 (1.2, 2.2, and 3.2). These analyses recover an unconventional relationship, in which Crocodylia comprises: (Crocodyloidea + (Alligatoroidea + Gavialoidea)) (Fig. 9E).

The compositions of Alligatoroidea and Crocodyloidea are very similar between all analyses and identical in Gavialoidea. Principle differences in topology mostly pertain to three labile taxa: *Borealosuchus*, *Diplocynodon*, and Planocraniidae (Fig. 9). *Borealosuchus* is recovered as a stem crocodylian in all analyses except 2.1 (in which it is recovered as a stem longirostrine) (Fig. 9B) and 3.1 (unresolved). *Diplocynodon* is recovered as a ‘basal’ alligatoroid in all analyses except 2.1 (in which it is recovered as a stem longirostrine) and 3.1 (unresolved). Whereas Planocraniidae is recovered as a ‘basal’ alligatoroid clade in analyses 1.2, 2.2, and 3.2 (EIW3), it occurs in the stem to Longirostres in analyses 1.3, 2.1, 2.3, and 3.3 (EIW12). In Analysis 1.1, Planocraniidae is polyphyletic; whereas *Planocrania hengdongensis* is recovered in the stem to Crocodylia, the clade (*Planocrania datangensis* + (*Boverisuchus vorax* + *Boverisuchus magnifrons*)) is recovered as a ‘basal’ member of Alligatoroidea. Otherwise, the taxonomic content of the principal crocodylian clades is relatively consistent between analyses.

### Phylogenetic support

**Figure 10:**
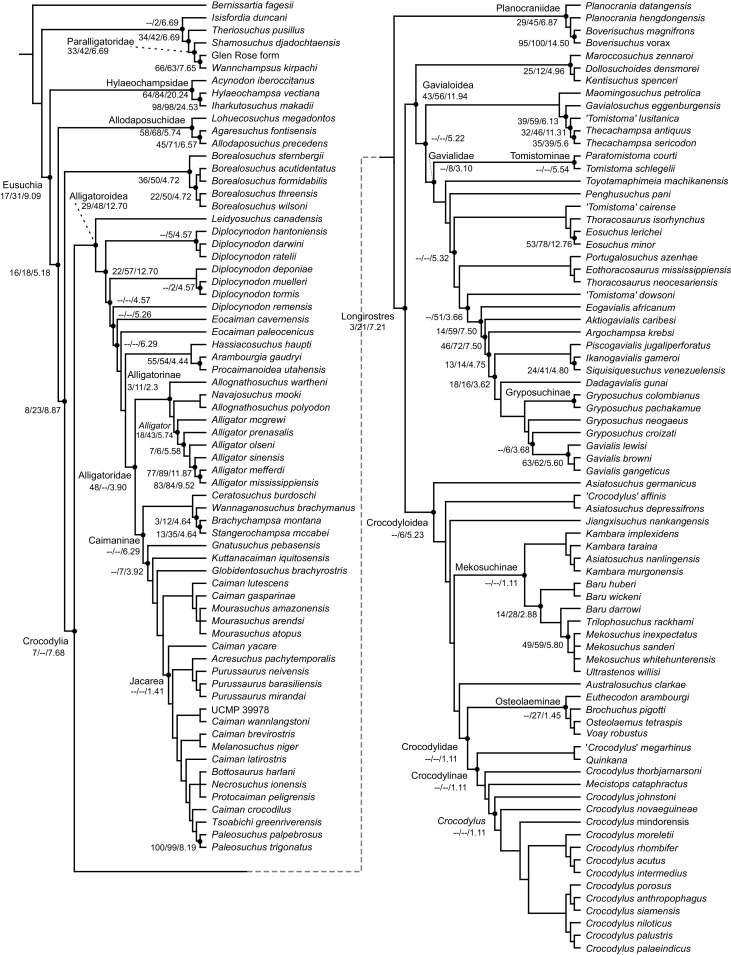
The strict consensus of the three most parsimonious trees obtained from Analysis 1.3. Values adjacent to nodes indicate values of internal consistency (Bootstrap/Jackknife/Bremer) and are shown only for nodes with Bremer support over 1 average step length.

Table 3 summarises three measures of internal phylogenetic support for each analysis: Bremer support (as number of nodes retained after collapsing branches with a support value less than one average step length), average Jackknife, and average Bootstrap. Furthermore, these values are mapped on to the topology resulting from Analysis 1.3 (Fig. 10). Overall, these measures are low across the trees of all analyses, which is most likely the result of the high degree of taxon sampling in this study, which includes a large number of incompletely preserved specimens. In Analysis 1 (quantitative characters treated continuously), the highest Bremer support, Jackknife, and Bootstrap values are consistently recovered under EIW12 (Analysis 1.3). The lowest support values are consistently obtained under EIW3 (Analysis 1.2). In the re-discretised dataset, average Bootstrap and Jackknife values are highest in the analysis employing EIW12, but lowest in the equal weighted analysis (Analysis 2.1). However, Analysis 2.1 retained the greatest number of nodes (84) above one step length and Analysis 2.2 retains the fewest (30). Among the analyses that excluded quantitative characters, Analysis 3.2 has the lowest Bremer support and Bootstrap values on average, whereas Analysis 3.3 had the highest Bremer support and Jackknife values on average.

**Table 3:** Summary of measures of internal tree support.

Analysis	MPTs	Tree length	CI	RI	Step length	Nodes retained	Average bootstrap	Average Jackknife
1.1	6	225,142.3	0.182	0.665	101.600	52	16.7	20.6
1.2	9	15,852.4	0.175	0.649	6.880	20	15.5	14.1
1.3	3	8,181.9	0.178	0.657	3.620	54	18.0	23.8
2.1	>400,000	2,279	0.182	0.644	1.000	84	7.5	7.9
2.2	3	158.0	0.170	0.645	0.067	30	13.9	19.2
2.3	3	83.2	0.174	0.654	0.037	49	17.5	22.3
3.1	>400,000	2,042	0.191	0.661	1.000	46	20.5	16.6
3.2	69	144.6	0.178	0.663	0.068	25	15.7	19.5
3.3	3	75.3	0.182	0.672	0.036	48	18.0	22.9

### Stratigraphic congruence

The topologies recovered under all analyses are more stratigraphically congruent than would be expected of random data. Three of the four indices (RCI, GER, and MSM*) show overall consistent trends. These indicate that Analysis 1.3 (continuous and discrete data with EIW12) recovered the most stratigraphically congruent topology, and Analysis 2.1 recovered the most incongruent topology (equal weighting and quantitative characters re-discretised) (Table 4). However, the SCI values do not follow this trend, instead indicating that the most stratigraphically congruent topology was recovered under Analysis 3.1, followed by analyses 2.1, 1.1, and 1.3, respectively. It is noteworthy that analyses 3.1 and 2.1 produced the largest number of MPTs. As such, the ranges of values of stratigraphic congruence between the best and worst trees in these analyses are the largest of all other analyses. Indeed, although analyses 3.1 and 2.1 recover the highest SCI values, Analysis 3.1 also recovers the lowest, and the worst tree in Analysis 2.1 is less stratigraphically congruent than the worst tree recovered in analyses 1.1, 1.3, and 2.3. Across all analyses, values for RCI, GER, and MSM* are consistently higher in analyses under EIW12 and usually lowest under EIW3 (Table 4).

**Table 4:** Summary of indices of stratigraphic congruence.

Analysis	SCI		RCI		GER		MSM*	
	Best tree	Worst tree	Best tree	Worst tree	Best tree	Worst tree	Best tree	Worst tree
1.1	0.493	0.479	−219.177	−219.703	0.837	0.836	0.054	0.054
1.2	0.451	0.430	−236.654	−247.555	0.828	0.822	0.052	0.050
1.3	0.472	0.456	−204.232	−206.205	0.845	0.844	0.057	0.057
2.1	0.507	0.437	−252.765	−314.393	0.819	0.786	0.050	0.042
2.2	0.437	0.415	−248.362	−254.279	0.821	0.818	0.050	0.049
2.3	0.458	0.444	−218.074	−219.269	0.838	0.837	0.055	0.055
3.1	0.542	0.415	−238.322	−307.601	0.827	0.789	0.051	0.043
3.2	0.458	0.430	−252.106	−277.419	0.819	0.806	0.049	0.046
3.3	0.451	0.437	−217.368	−220.034	0.838	0.837	0.055	0.054

**Note:**

The *p*-values for all stratigraphic congruence values (not shown) are < 0.0001.

### Detailed topological results

In order to facilitate the presentation of topological results in more detail, the results of one analysis are chosen to avoid lengthy and unwieldy comparisons between multiple alternative topologies. The results of analyses 1.2, 2.2, and 3.2 are considered the least probable, because the sister relationship between Alligatoroidea and Gavialoidea contradicts the consensus of all morphological and molecular analyses to date. Furthermore, the trees produced from these analyses are among the least stratigraphically congruent and have the lowest Bremer support values of all analyses (Table 3). These results likely reflect the severe weighting factor (*k* = 3) implemented in these analyses, which has been advised against in large datasets (Goloboff, Torres & Arias, 2018). Analyses 3.1 and 2.1 recovered large numbers of MPTs (>400,000 in each case), such that the resultant strict consensus trees are the least informative of all other analyses, and they are also characterised by low values of stratigraphic congruence. Analyses 1.1, 1.3, 2.3, and 3.3 recover similar topologies (Fig. 9), with Analysis 1.3 producing the most stratigraphically congruent result. Consequently, further discussion is centred on Analysis 1.3, although comparisons are made with analyses 1.1, 2.3, and 3.3, where relevant.

#### Overall topology

The stem of Crocodylia comprises a series of successively nested, early diverging non-crocodylian eusuchians (Fig. 10). A division occurs at the crown node Crocodylia, which separates Alligatoroidea from Planocraniidae + Longirostres. Within Longirostres, a further branching occurs between Gavialoidea and Crocodyloidea.

#### Non-crocodylian eusuchians

*Isisfordia duncani* + (*Theriosuchus pusillus* + Paralligatoriidae) are recovered as the earliest diverging non-crocodylian eusuchians in this dataset. The clade comprising *Theriosuchus pusillus* + (*Shamosuchus djadochtaensis* + (*Wannchampsus kirpachi* + the ‘Glen Rose Form’)), is similar to previous studies (*e.g*. Turner, 2015; Tennant, Mannion & Upchurch, 2016). Hylaeochampsidae (*Acynodon iberoccitanus* + (*Iharkutosuchus makadii* + *Hylaeochampsa vectiana*)) and Allodaposuchidae (*Lohuecosuchus megadontos* + (*Allodaposuchus precedens* + *Agaresuchus fontisensis*) are recovered as successively branching lineages. This is consistent with Narváez et al. (2016), but differs from many earlier studies, which support a sister relationship between Hylaeochampsidae and Allodaposuchidae (*e.g*. Brochu et al., 2012; Narváez et al., 2015; Turner, 2015). Moving crownward, the multispecific *Borealosuchus* is recovered as the sister clade to Crocodylia. The monophyly of *Borealosuchus* is well supported (recovered in 50% of Jacknife replicates, Bremer support 4.7). Relationships within *Borealosuchus* are weakly supported, but *B*. *sternbergii* is recovered as the earliest diverging species of the genus, outside of two pairs of nested sister taxa: (*B*. *wilsoni* + *B*. *threeensis*) + (*B*. *formidabilis* + *B*. *acutidentatus*).

#### Alligatoroidea

Similar to most previous studies, *Leidyosuchus canadensis* and *Diplocynodon* are recovered as the earliest diverging members of Alligatoroidea (*e.g*. Brochu, 1999a; Brochu et al., 2012). However, by contrast to nearly all previous analyses (see Rio et al., 2020), the multispecific genus *Diplocynodon* is paraphyletic. This paraphyly results in the replacement of a conventional divergence between ‘Diplocynodontinae’ and ‘Globidonta’ (*i.e*. all other alligatoroids) (Brochu, 1999a) with successive branching lineages in the stem leading to the crown group Alligatoridae. Whereas *D*. *hantoniensis* is most closely related to *D*. *ratelii* + *D*. *darwini*, the clade comprising *D*. *deponiae* + (*D*. *tormis* + *D*. *muelleri*) is recovered further crownward. *D*. *remensis* is recovered most crownward of all putative *Diplocynodon* species.

Crown group Alligatoridae is divided into Alligatorinae and Caimaninae; however, the taxonomic content and arrangement of both subclades is very different to previous studies. Firstly, the putative early caimanines, *Eocaiman cavernensis* and *Eocaiman palaeocenicus*, are placed outside of Alligatoridae, as is also the case for a series of taxa usually recovered as alligatorines (*Hassiacosuchus haupti*, *Procaimanoidea utahensis*, and *Arambourgia gaudryi*). Alligatorinae comprises the early diverging *Allognathosuchus wartheni*, which lies outside of the clade formed by (*Allognathosuchus polyodon* + *Navajosuchus mooki*) + *Alligator*. Except for the recovery of *Alligator mcgrewi* as the earliest diverging member of *Alligator*, the topology within *Alligator* is congruent with most previous studies (*e.g*. Brochu, 1999a). *A*. *mississippiensis* and *A*. *mefferdi* are recovered as deeply nested sister taxa, with *A*. *mcgrewi*, *A*. *prenasalis*, *A*. *olseni*, and *A*. *sinensis*, as successively branching stemward taxa.

The composition and topology of Caimaninae also differs from most previous findings. Firstly, two taxa traditionally recovered as alligatorines, *Ceratosuchus burdoshi* and *Wannaganosuchus brachymanus*, and two traditionally ‘basal globidontans’, *Brachychampsa montana* and *Stangerochampsa mccabei*, form an early diverging stem caimanine clade. The latter two species were also recovered in a similar position in the analyses of Salas-Gismondi et al. (2015) and Bona et al. (2018). In line with other recent studies, *Gnatusuchus pebasensis*, *Kuttanacaiman iquitosensis*, and *Globidentosuchus brachyrostris* are also recovered among the earliest diverging caimanines (*e.g*. Salas-Gismondi et al., 2015, 2019; Cidade, Fortier & Hsiou, 2020).

At this point, a divergence occurs between Jacarea and the clade comprising: *Caiman lutescens* + (*Caiman gasparinae* + *Mourasuchus*) (Fig. 10). The composition of Jacarea is considerably expanded from its traditional meaning, but support for internal nodes is very low. *Caiman yacare* is the earliest diverging member of Jacarea, ‘basal’ to a clade comprising (*Acresuchus pachytemporalis* + (*Purussaurus neivensis* + (*Purussaurus mirandai* + *Purussaurus brasiliensis*))) and a complex series of successively branching clades (Fig. 10). Notably, the clade consisting of *Caiman crocodilus* + (*Tsoabichi greenriverensis* + (*Paleosuchus trigonatus* + *Paleosuchus palpebrosus*)) is deeply nested within Jacarea, and sister to a polytomous clade of stratigraphically early caimanines, comprising *Protocaiman peligrensis*, *Necrosuchus ionensis*, and *Bottosaurus harlani*. The placement of this latter trio of taxa within Caimaninae is consistent with recent analyses, including evidence for a deeply nested position for both *Bottosaurus* and *Necrouchus* (Brochu, 2011; Bona et al., 2018; Cossette & Brochu, 2018; Cidade, Fortier & Hsiou, 2020; Cossette, 2021; Godoy et al., 2020).

#### Crocodyloidea

Crocodyloidea is recovered as the sister lineage to Gavialoidea, forming the clade Longirostres. The earliest diverging crocodyloids are *Asiatosuchus germanicus*, (‘*Crocodylus*’ *affinis* + *Asiatosuchus depressifrons*), and *Jiangxisuchus nankangensis*. These taxa form the stem of a large clade marked by a basal division broadly separating Mekosuchinae and the crown group Crocodylidae. The composition of Mekosuchinae is slightly different to previous analyses (*e.g*. Lee & Yates, 2018). Most notably, *Asiatosuchus nanlingensis* is deeply nested in Mekosuchinae, as the sister taxon to *Kambara murgonensis*. Furthermore, two putative mekosuchines are recovered outside of Mekosuchinae, with *Australosuchus clarkae* recovered in the stem to Crocodylidae, and *Quinkana* recovered as the sister taxon of ‘*Crocodylus*’ *megarhinus*, within Crocodylinae. Mekosuchinae is composed of two sister lineages, which separate (*Kambara implexidens* + (*Kambara taraina* + (*Kambara murgonensis* + *Asiatosuchus nanlingensis*))) from (*Baru wickeni* + *Baru huberi* + (*Baru darrowi* + (*Trilophosuchus rackhami* + (*Ultrastenos willisi* + *Mekosuchus* ssp.)))).

Crocodylidae is divided into two clades: Osteolaeminae and Crocodylinae. Within Osteolaeminae, *Euthecodon arambourgi* and *Brochuchus pigotti* are successively nested taxa to a clade consisting of *Voay robustus* + *Osteolaemus tetraspis*. The clade ‘*Crocodylus*’ *megarhinus* + *Quinkana* is recovered in the stem of Crocodylinae, outside of the crown genus *Crocodylus*. *Crocodylus thorbjarnarsoni* and the extant African slender snouted crocodile, *Mecistops cataphractus*, are similarly recovered as stem crocodylines. The relationships within the crown genus *Crocodylus* show broad similarities to the results of molecular and existing morphological phylogenies (*e.g*. Brochu, 2000; Oaks, 2011). Three extant Indo-Pacific *Crocodylus* species (*C*. *johnstoni*, *C*. *novaeguineae*, and *C*. *mindorensis*) are the earliest diverging members of the genus (Fig. 10). These species are successively nested in the stem of the crown genus *Crocodylus*, which broadly separates extant Neotropical *Crocodylus* species from the remaining Indo-Pacific and African species. Within the Neotropical clade, *C*. *moreletii* and *C*. *rhombifer* are successively nested species stemward to *C*. *intermedius* + *C*. *acutus*. On the opposite branch of the clade, (*C*. *niloticus* + (*C*. *palaeindicus* + *C*. *palustris*)) and (*C*. *porosus* + (*C*. *siamensis* + *C*. *anthropophagus*)) are sister clades.

#### Gavialoidea

By contrast to the results of nearly all morphological phylogenies, we do not recover Tomistominae and Gavialoidea as separate lineages. In line with molecular analyses, as well as many combined morphological and molecular analyses, we recover *Tomistoma schlegelii* as the closest living relative of *Gavialis gangeticus*, defining the crown clade Gavialidae. Here, Tomistominae in the traditional sense (*e.g*. Brochu, 2007b; Piras et al., 2007; Jouve, 2016) is paraphyletic, recovered as a series of successively nested, early diverging lineages in Gavialoidea. This topology is most similar to that recovered in combined morphological and molecular analyses (Lee & Yates, 2018; Iijima & Kobayashi, 2019).

The earliest diverging gavialoids recovered here are: (*Maroccosuchus zennaroi* + (*Kentisuchus spenceri* + *Dollosuchoides densmorei*)). Support is found for a second early diverging clade of traditional tomistomines at the base of Gavialoidea, composed of (*Maomingosuchus petrolica* + (*Gavialosuchus eggenburgensis* + (‘*Tomistoma*’ *lusitanica* + (*Thecachampsa sericodon* + *Thecachampsa antiquus*)))) (Fig. 10). This lineage is the sister group of the crown clade Gavialidae. The earliest diverging gavialids are: (*Tomistoma schlegelii* + *Paratomistoma courti*), *Toyotamaphimeia machikanensis*, and *Penghusuchus pani*.

Taxa commonly referred to as ‘thoracosaurs’, such as *Thoracosaurus* ssp., *Eosuchus* ssp., and *Eothoracosaurus mississippiensis*, are nested within Gavialidae, but comprise a polyphyletic assemblage. Whereas the clade consisting of *Thoracosaurus isorhynchus* + (*Eosuchus minor* + *Eosuchus lerichei*) is recovered as the sister clade to ‘*Tomistoma*’ *cairense*, *Thoracosaurus neocesariensis* and *Eothoracosaurus mississippiensis* form a separate clade with *Portugalosuchus azenhae*, from the early Late Cretaceous of Portugal (Mateus, Puértolas-Pascual & Callapez, 2019).

Moving crownward, ‘*Tomistoma*’ *dowsoni*, *Eogavialis africanum*, *Aktiogavialis caribesi*, and *Argochampsa krebsi* are successively nested taxa, ‘basal’ to a clade of Neotropical and Indian gavialids. This clade divides (*Piscogavialis jugaliperforatus* + (*Siquisiquesuchus venezuelensis* + *Ikanogavialis gameroi*)) from *Dadagavialis gunai* and all species of *Gryposuchus* and *Gavialis*. *Gryposuchus* is paraphyletic, with *Gryposuchus neogaeus* and *Gryposuchus croizati* more closely related to *Gavialis* than to the remaining two species usually included within *Gryposuchus* (*Gryposuchus pachakamue* + *Gryposuchus colombianus*). Consequently, Gryposuchinae *sensu* Vélez-Juarbe, Brochu & Santos (2007) is not recovered. *Gavialis* is monophyletic, with a sister relationship between *Gavialis gangeticus* and *Gavialis browni*, to the exclusion of *Gavialis lewisi*.

## Discussion

### The impact of quantitative character treatment

The impact of different treatments of quantitative characters is most apparent between equal weighted analyses. The strict consensus trees of analyses 2.1 and 3.1 are increasingly less well-resolved than Analysis 1.1. This result was anticipated, given that equal step lengths resulting from the use of discrete-only characters in these analyses allows for numerous ties in optimal tree length. Furthermore, Analysis 3.1 was also expected to have the poorest resolution given that fewer characters were utilised (304) than in the other two analyses (330). The treatment of quantitative characters appears to be relatively insignificant under EIW. There are fewer topological differences between the three analyses using a *k*-value of 3 and those applying a value of 12, compared to the three equal weighted analyses. This is illustrated by the number of taxa retained in the agreement subtrees of analyses under different weighting strategies (Table 5). Furthermore, the differences in topology are restricted to the interrelationships of nested taxa, rather than the overall topology of each tree. This is exemplified in the strict consensus of the nine trees resulting from analyses 1.3, 2.3, and 3.3 (Fig. S1). By contrast, there are considerably fewer taxa retained in the agreement subtrees of analyses that treat quantitative data the same way, but use different weighting strategies (Table 5). Furthermore, the differences in topology are widespread. For example, the strict consensus of analyses 1.1, 1.2, and 1.3 results in a large polytomy between the principal crocodylian lineages, with little to no resolution in any major clade (Fig. S2). This indicates that weighting strategy has more control on tree topology than how quantitative data is treated, a result similar to that found in studies of sauropod dinosaurs (Mannion et al., 2013) and neosuchian phylogeny (Groh et al., 2020).

**Table 5:** Maximum number of taxa retained in agreement subtrees from pairwise comparisons of each analysis.

Analysis	1.1	1.2	1.3	2.1	2.2	2.3	3.1	3.2	3.3
**1.1**	–	53	83	69	53	87	65	54	89
**1.2**	–	–	72	34	119	74	22	117	71
**1.3**	–	–	–	53	68	114	20	74	126
**2.1**	–	–	–	–	34	66	41	48	68
**2.2**	–	–	–	–	–	74	23	117	74
**2.3**	–	–	–	–	–	–	23	72	126
**3.1**	–	–	–	–	–	–	–	23	23
**3.2**	–	–	–	–	–	–	–	–	73
**3.3**	–	–	–	–	–	–	–	–	–

Similar to Groh et al. (2020), during the re-discretisation of continuous characters, it was clear that the existing character state boundaries for some characters were arbitrarily drawn. Furthermore, in characters where new character state boundaries had to be erected, discontinuities in the data marking obvious threshold values were sometimes absent or challenging to interpret (Document S2). 50% of the continuous characters have normally distributed values and the remainder exhibit skewed distributions. Altogether, these results suggest that it is more appropriate to treat quantitative data continuously, rather than imposing arbitrary values to delimit discrete character states.

### The use of extended implied weighting

Application of extended implied weighting with *k* = 3 results in trees that consistently score more poorly for measures of phylogenetic accuracy, internal consistency, and stratigraphic congruence, than those produced with a *k*-value of 12, and (in most cases) those under equal weights (Table 3). All analyses using EIW3 recover a topology in which Gavialoidea is more closely related to Alligatoroidea than to Crocodyloidea, a hypothesis that contrasts with all previous results based on morphological, molecular, and combined analyses. Fewer internal nodes are supported in Jackknife and Bootstrap replicates, and those retained have lower support values compared to analyses under EIW12. Furthermore, analyses implementing EIW3 consistently achieve lower values for stratigraphic congruence than analyses using EIW12, and they are often lower than equal weighted analyses. Following Goloboff, Torres & Arias (2018), these results argue in favour of the use of higher *k*-values (see also Tschopp & Upchurch, 2019). This contrasts with the neosuchian-focused dataset of Groh et al. (2020), which consistently found higher values of stratigraphic congruence with *k* = 3. Several factors might have contributed to this, including differences in character sampling, numbers of continuous characters, ingroup composition, and number of OTUs, but it is beyond the scope of the present study to evaluate the causes of these differences.

### Anatomical support and implications for the systematics of non-crocodylian eusuchians

#### Isisfordia

**Figure 11:**
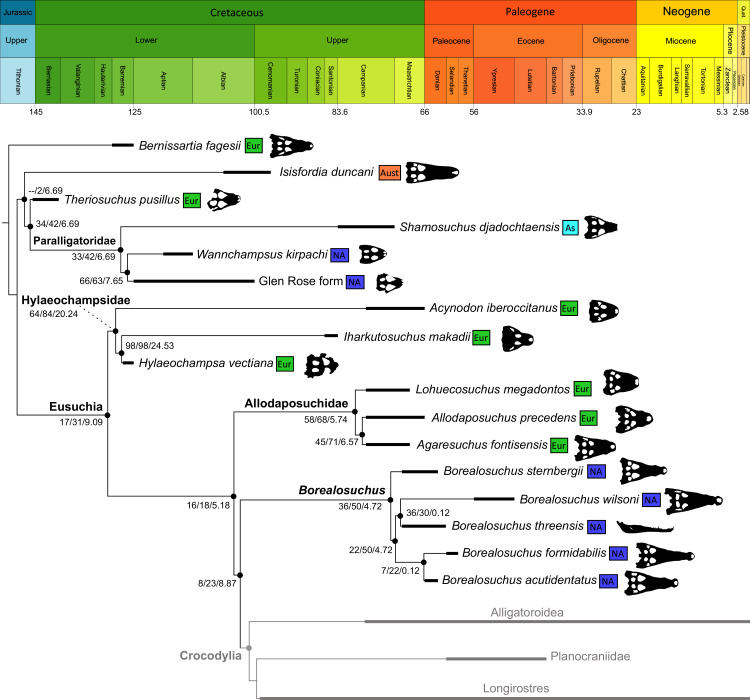
Time-calibrated phylogeny of stem crocodylian taxa from Analysis 1.3. Ages = Ma. Support values: Bootstrap/Jackknife/Bremer. Abbreviations: As, Asia; Aust, Australasia; Eur, Europe; NA, North America.

There are conflicting hypotheses for the phylogenetic affinities of *Isisfordia duncani*, from the mid-Cretaceous of Australia (Salisbury et al., 2006; Turner, 2015; Turner & Pritchard, 2015; Groh et al., 2020; Martin et al., 2020). Originally *Isisfordia* was recovered as the earliest diverging member of Eusuchia, sister to the clade formed by *Allodaposuchus precedens* + Crocodylia (Salisbury et al., 2006). This placement was based on the combination of plesiomorphic and derived eusuchian character states, such as the presence of incipiently procoelous vertebrae and the apparently pterygoid-bound choanae. However, Turner & Pritchard (2015) reassessed this morphology, and demonstrated the participation of the palatine in the choanae. In an analysis incorporating this re-interpretation, Turner & Pritchard (2015) recovered *Isisfordia* as an early diverging neosuchian, ‘basal’ to the outgroup in our analysis, *Bernissartia fagesii*. By contrast, a recent study of neosuchian relationships recovered *Isisfordia* within the crown group Crocodylia (Groh et al., 2020). However, that analysis produced a relatively unconventional topology, in which Hylaeochampsidae was also recovered in the crown group, closely related to *Isisfordia*. This might reflect the absence of members of Allodaposuchidae from that dataset. *Isisfordia* is here recovered outside of Eusuchia, as the sister taxon to Paralligatoridae (Fig. 11).

Given that *Isisfordia* might represent an early diverging neosuchian, its relationships should be more thoroughly tested in a larger sample of non-eusuchian neosuchians, one that also incorporates recently identified additional species of this genus (Hart et al., 2019; Hart, 2020). Re-running Analysis 1.3 with *Isisfordia* excluded *a priori* results only in the movement of *Theriosuchus* from the stem of Paralligatoridae to the earliest diverging ingroup taxon.

#### Hylaeochampsidae and Allodaposuchidae

Hylaeochampsidae is relatively well supported (Jackknife = 84, Bootstrap = 64), in particular the sister relationship between (*Hylaeochampsa vectiana* + *Iharkutosuchus makadii*) and *Acynodon iberoccitanus* (Jackknife = 98, Bootstrap = 98), which is commonly recovered (*e.g*. Ősi, Clark & Weishampel, 2007; Brochu et al., 2012; Narváez et al., 2015, 2016; Mateus, Puértolas-Pascual & Callapez, 2019). Hylaeochampsidae is supported by ten synapomorphies: (1) premaxilla-maxilla suture at the same level or anterior to the posterior margin of the nares (C49-1, shared); (2) lacrimal-nasal contact absent (C53-1, shared); (3) spina quadratojugalis greatly reduced or absent (C103-1, shared); (4) premaxilla extends posteriorly on the palate to the level of 3 maxillary alveoli (C142-3, ambiguous); (5) premaxilla-maxilla suture bowed with one rounded apex (C146-1, shared); (6) maxillary alveoli gradually increase in diameter posteriorly toward penultimate alveolus (C147-6, unambiguous); (7) diastema between maxillary alveoli 5 and 6 (C152-1, shared); (8) posterior dentary and maxillary teeth molariform and multicusped (C156-2, unambiguous); (9) anterior margin of the suborbital fenestra at the same level or posterior to the anterior margin of the orbit (C166-1, shared); and (10) ectopterygoid extends to the level of or anterior to two thirds the suborbital fenestra length (C174-1, shared).

There is lower support for Allodaposuchidae (Jackknife = 68, Bootstrap = 58) but, in common with the majority of recent analyses, this clade is more closely related to Crocodylia than to Hylaeochampsidae (*e.g*. Narváez et al., 2016; Mateus, Puértolas-Pascual & Callapez, 2019). Allodaposuchidae is supported by seven synapomorphies: (1) ratio of interorbital distance to width across the anterior cranial table margin (C6: 0.3–0.4); (2) ratio of incisive foramen width to rostrum width at premaxilla-maxilla suture (C12: 0.17–0.19); (3) presence of a transverse interorbital bridge (C31-1, shared); (4) largest maxillary alveolus is number 4 (C147-2, shared); (5) dentary symphysis adjacent to 9–12 alveoli (C222-2, shared); (6) anterior perforation of splenial for cranial nerve V absent (C229-1, shared); and (7) surangular-articular suture bowed laterally in glenoid fossa (C247-1, shared).

#### Borealosuchus

The phylogenetic affinities of *Borealosuchus* contrast between studies. Whereas most previous analyses have recovered *Borealosuchus* in the stem of Brevirostres (Alligatoroidea + Crocodyloidea) (Brochu, 1997a, 2004a; Hua & Jouve, 2004; Brochu, 2006; Jouve et al., 2015; Narváez et al., 2015, 2016; Salas-Gismondi et al., 2016; Groh et al., 2020), others recover *Borealosuchus* as either the sister taxon to Gavialoidea (Salisbury et al., 2006; Pol, Turner & Norell, 2009; Martin, 2010a; Puértolas-Pascual, Canudo & Cruzado-Caballero, 2011), or in a polytomy with Gavialoidea and Brevirostres (Brochu, 2012; Brochu et al., 2012; Hastings, Reisser & Scheyer, 2016). In Analysis 1.3 (and indeed all analyses except 2.1 and 3.1), *Borealosuchus* is the sister taxon to Crocodylia, similar to the results of Gatesy et al. (2003) and Gold, Brochu & Norell (2014). This result is weakly supported in Jackknife (50) and Bootstrap (36) replicates, with Bremer support of 4.7.

*Borealosuchus* + Crocodylia is supported by three continuous and three discrete synapomorphies: (1) ratio of snout length to skull length (C1: 0.58–0.60); (2) cranial table length to width (C8: 0.69); (3) number of maxillary alveoli (C17: 17); (4) absence of fossa on anteromedial corner of supratemporal fenestra (C84-1, shared); (5) indentation of the mediolateral margin of quadrate condyle (C118-1. ambiguous); and (6) presence of an external mandibular fenestra (C234-1, shared). Outside of this clade, the absence of an anteromedial fossa of the supratemporal fenestra is known only in *Isisfordia duncani* and *Shamosuchus djadochtaensis*. Similarly, where preserved, the only non-crocodylian eusuchian to exhibit an external mandibular fenestra is *Isisfordia duncani*. The morphology of the quadrate condyle (C118-1) provides additional ambiguous support for *Borealosuchus* + Crocodylia, given that the condition is shared by the outgroup. Furthermore, the morphology of the condyle changes multiple times in crownward nodes of Crocodylia. For example, whereas most alligatoroids share the condition with *Borealosuchus*, it is lost in longirsotrines (C118-0) and re-acquired in Mekosuchinae (C118-1).

### Emended diagnosis of Crocodylia

Our results enable us to provide an emended diagnosis for Crocodylia, which is supported by one continuous and nine discrete synapomorphies: (1) width to length ratio of the external naris (C3: 1.0–1.2); (2) lacrimal anteroposteriorly longer than the prefrontal (C58-0, ambiguous); (3) absence of a protuberance on the dorsolateral margin of the postorbital bar (C90-1, ambiguous); (4) presence of a surangular spur lingual to posteriormost dentary alveoli (C242-0, ambiguous); (5) first postaxial vertebra with a prominent hypapophysis (C279-0, ambiguous); (6) scapulocoracoid facet broad immediately anterior to glenoid fossa, and tapering anteriorly (C301-1, ambiguous); (7) olecranon process of ulna wide and rounded (C305-1, shared); (8) proximal diaphysis of ulna straight (C306-1, shared); (9) six dorsal osteoderms per transverse row at maturity (C324-2, ambiguous); and (10) absence of an anterolateral process on the dorsal midline osteoderms (C327-1, ambiguous).

The diagnostic utility of most of these synapomorphies is weak given that many of them also appear in some non-crocodylian eusuchians and are not present in all crocodylians (*e.g*. 58-0, 90-1, 242-0, 324-2, 327-1). The presence of a hypopophysis on the first postaxial vertebra (279-0), as well as a tapering scapulocoracoid facet (301-1), do not characterise any non-crocodylian eusuchians included in our analysis, where preserved; however, not all crocodylians share these features. Additionally, there is a considerable amount of missing data for these two characters. Two morphological features of the ulna are shared synapomorphies of Crocodylia: (1) a rounded olecranon process (305-1); and (2) a straight (rather than bowed) proximal end of the diaphysis (306-1). Both of these features appear independently in some paralligatorids. However, as with other postcranial synapomorphies discussed, there is a large proportion of missing data for these characters.

### Anatomical support and implications for the systematics of Alligatoroidea

**Figure 12:**
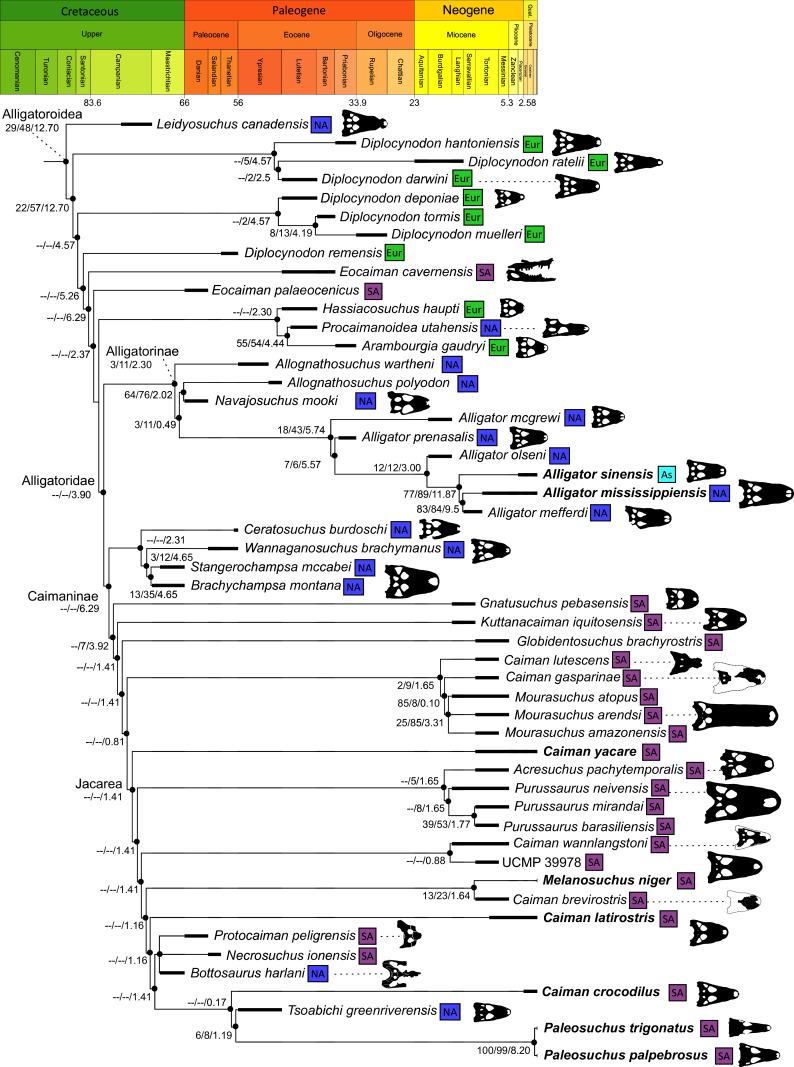
Time-calibrated phylogeny of Alligatoroidea from Analysis 1.3. Ages = Ma. Support values: Bootstrap/Jackknife/Bremer. Abbreviations: As, Asia; Eur, Europe; NA, North America; SA, South America.

Alligatoroidea has been one of the most stable clades in Crocodylia in terms of taxonomic content and internal phylogenetic relationships, with strong concordance between molecular and morphological topologies (*e.g*. Brochu, 1999a; Oaks, 2011; Jouve et al., 2015). This owes to extensive study of this group. Here we recover a consistent taxonomic content of Alligatoroidea, but with several new interrelationships and support for internal clades (Fig. 12).

#### Modifications to the diagnosis of Alligatoroidea

Alligatoroidea is supported by two continuous and eight discrete synapomorphies: (1) ratio of antorbital rostrum width to cranial table width = 1.7 (C2); (2) supratemporal fenestra width to length = 0.63–0.67 (C11); (3) transverse ridge between orbits (C31-1, ambiguous); (4) quadratojugal spine positioned high between the posterior and dorsal angles of the infratemporal fenestra (C104-1, shared); (5) posterior extent of premaxillae on palate not reaching one maxillary alveolus (C142-0, ambiguous); (6) posterior and anterolateral margins of choanae elevated (C195-2, ambiguous); (7) dorsal surangular processes subequal (C241-1, ambiguous); (8) surangular does not extend to posterior tip of retroarticular process (C245-1, ambiguous); (9) foramen aerum inset from medial margin of retroarticular process (C248-1, shared); and (10) dorsal extent of the retroarticular process at the same level or ventral to articular fossa (C251-0, ambiguous).

This differs to earlier diagnoses of Alligatoroidea (Norell, Clark & Hutchison, 1994; Brochu, 1999a) in several respects. Not only are there differences in the characters considered diagnostic of Alligatoroidea, but many more synapomorphies appear to be shared and ambiguous, and therefore seem ‘weaker’. The latter is usually because of the stricter definition of synapomorphies used here (see Methods). A dorsally positioned foramen aerum on the quadrate condyle, and a broad separation of the ectopterygoid from the maxillary toothrow, have long since been regarded as unambiguous synapomorphies of Alligatoroidea (Norell & Clark, 1990; Norell, Clark & Hutchison, 1994; Brochu, 1999a). These characters are still employed in this study, but no longer diagnose the clade. The foramen aerum is dorsally positioned in all alligatoroids where preserved (C117-1), but we consider the same condition to occur in some non-alligatoroid taxa, such as *Borealosuchus sternbergii* (USNM 6533, Appendix 2: fig. 45) and *Borealosuchus formidabilis* (Erickson, 1976, fig. 6). A dorsally positioned foramen aerum has also previously been recognised in Allodaposuchidae, *e.g. Allodaposuchus precedens* (MMS/VBN-12-10A) and *Agaresuchus fontisensis* (Narváez et al., 2016, fig. 3B), and recently in the hylaeochampsid *Iharkutosuchus makadii* (Mateus, Puértolas-Pascual & Callapez, 2019, fig.S14B). As such, a dorsally positioned foramen aerum is recovered as a synapomorphy of Eusuchia, which is lost at the node comprising Planocraniidae + Longirostres. Similarly, a broad separation of the ectopterygoid and maxilla (C175-0) is recognised far more widely within Neosuchia. This character has received significant score changes, with the ‘alligatoroid condition’ newly recognised in *Bernissartia fagesii* (Appendix 2: fig. 45A), several *Borealosuchus* species (Appendix 2: figs. 45B–45C), and most gavialoids (Appendix 2: figs. 45E, 45G). Following Brochu (1999a), subequal surangular processes (C241-1) and a medially inset foramen aerum on the retroarticular process (C248-1) are still recovered as synapomorphies of Alligatoroidea; however, these are no longer unambiguous. For example, both *Borealosuchus formidabilis* (Erickson, 1976), and the paralligatorid *Shamosuchus djadochtaensis* (Pol, Turner & Norell, 2009) are also characterised by the presence of subequal surangular processes, whereas they are unequal in the alligatoroid, *Procaimanoidea utahensis* (USNM 15996). Similarly, a medially inset foramen aerum is also present in the paralligatorid, *Wannchampsus kirpachi* (Adams, 2014), as well as the crocodyloid, *Mekosuchus inexpectatus* (MNHN NCP 06).

Five synapomorphies of Alligatoroidea are newly recognised in this study, all of which are ambiguous. A transverse interorbital bridge (C31-1) is present in the earliest diverging alligatoroids, but seemingly lost and reacquired independently in some alligatorines and most caimanines. Outside of Alligatoroidea, an interorbital bridge is present in *Acynodon iberoccitanus*, Allodaposuchidae, and the ‘basal’ crocodyloid *Jiangxisuchus nankangensis*. Within Alligatoroidea, a short posterior extension of the premaxillae on the palate (C142-0) only occurs in *Leidyosuchus canadensis*, *Diplocynodon*, and *Paleosuchus trigonatus*. All other alligatoroids exhibit longer posterior extensions of the premaxillae. The highly shortened condition also occurs multiple times independently in other eusuchians. Everted anterolateral margins of the choanae (C195-2) is a newly recognised condition, exhibited by all species of *Diplocynodon* (where preserved) and *Leidyosuchus canadensis*. This condition is absent in all alligatoroids crownward of *Diplocynodon remensis*; although they still possess everted lateral margins, these are morphologically distinct. A surangular that is truncated before reaching the posterior tip of the retroarticular process (C245-1) was not listed as a synapomorphy of Alligatoroidea by Brochu (1999a), but it was optimised as such in the dataset of Narváez et al. (2016). This synapomorphy is ambiguous because this condition is lost in most caimanines and is also shared by many gavialoids. Variation in the dorsoventral height of the retroarticular process was not discretised by Brochu (1999a). In the current study, a ventrally positioned retroarticular process (C251-0) characterises most alligatoroids, but is also present in several non-crocodylian eusuchians, including Paralligatoridae.

#### The phylogenetic relationships and monophyly of Diplocynodon

The phylogenetic relationships of *Diplocynodon* have been tested by a number of authors (Brochu, 1997a, 1999a; Martin, 2010a; Martin & Gross, 2011; Brochu et al., 2012; Delfino & Smith, 2012; Martin et al., 2014; Groh et al., 2020; Rio et al., 2020). *Diplocynodon* has consistently been recovered as a monophyletic group, almost always as an early diverging alligatoroid genus (but see Groh et al., 2020); however, there is surprisingly little consensus over the interrelationships of *Diplocynodon* species (Fig. 5). Adding to this lack of previous consensus, the preferred topology of this study recovers a paraphyletic *Diplocynodon*, comprising a series of successively diverging clades (Fig. 12).

Despite its stratigraphically earlier appearance than all other *Diplocynodon* species (Martin et al., 2014), *D*. *remensis* is recovered in the most crownward position of all *Diplocynodon* species. Three characters are optimised as synapomorphies of *D*. *remensis* and all crownward nodes: (1) a tall interorbital bridge (C32-1, ambiguous); (2) a frontoparietal suture that incipiently contacts the supratemporal fenestrae (C75-1, ambiguous); and (3) a quadrate excluded from the ventral margin of the orbitotemporal canal (C87-2, ambiguous). A tall interorbital bridge is an ambiguous synapomorphy. Not only is it absent in several alligatoroids more crownward than *D*. *remensis* (including all alligatorines), but, as a result of reductive coding, it is also inapplicable to several members of this clade (*e.g. Hassiacosuchus*, *Procaimanoidea*, *Arambourgia*). A frontoparietal suture which incipiently contacts the supratemporal fenestra (C75-1) is absent in all other species of *Diplocynodon* (C75-0), and almost all more crownward taxa (C75-2). However, as this character is ordered, this topology allows the more parsimonious, stepwise optimisation of this character. In all *Diplocynodon* species except *D*. *remensis* and *D*. *muelleri*, the quadrate contributes to a small portion of the ventral margin of the orbitotemporal canal (C87-1). This is considered intermediate between the plesiomorphic eusuchian condition, in which there is a large quadrate participation (C87-0), and a derived condition found in almost all alligatorids, where there is no quadrate participation (C87-2) (Appendix 2: fig. 32). *D*. *remensis* exhibits the ‘derived’ condition, supporting its crownward position. Otherwise, only *D*. *muelleri* exhibits this condition among *Diplocynodon* species.

A further five characters suggest that the clade comprising (*D*. *deponiae* + (*D*. *tormis* + *D*. *muelleri*)) is more closely related to *D*. *remensis* and all crownward nodes than to that consisting of *D*. *hantoniensis* + (*D*. *ratelii* + *D*. *darwini*): (1) the ratio of snout length to skull length = 0.48–0.51 (C1); (2) the pterygoid width to length = 3.2 (C15); (3) flush orbital margins (C72-0, ambiguous), (4) anterior splenial tip passing dorsal to meckelian fossa (C223-1, ambiguous); and (5) a tail completely encased in osteoderms (C329-1). Although not calculated in *D*. *remensis* due to deformation, the snout length is proportionally shorter in this clade compared to (*D*. *hantoniensis* + (*D*. *ratelii* + *D*. *darwini*)). The proportions of the pterygoid appear to also support this clade; however, they could not be calculated for *D*. *remensis*, *D*. *tormis*, or *D*. *muelleri*. Character 223 is reductively coded, such that the character is inapplicable to any taxon with a splenial symphysis, including *D*. *remensis*. Flush orbital margins (C72-0) is an ambiguous synapomorphy as it characterises both *D*. *darwini* and *D*. *deponiae*, whereas *D*. *muelleri* has upturned orbital margins (72-1). Although the presence of a tail encased in osteoderms also supports this clade, this character can only be assessed in *D*. *darwini* and *D*. *deponiae* among *Diplocynodon* species.

In all equal weighted analyses, *Diplocynodon* is recovered as monophyletic. Furthermore, *Diplocynodon* forms the sister taxon to *Leidyosuchus canadensis* in analyses 1.1 and 2.1. The monophyly of both *Diplocynodon* and the clade *Leidysosuchus* + *Diplocynodon* is relatively well supported in those analyses (Bremer support = 2). The only other analysis recovering a monophyletic *Diplocynodon* is Analysis 3.1; however, this clade lacked any internal resolution and is itself part of a large polytomy with all other eusuchians. Constraining Analysis 1.3 to recover a monophyletic *Diplocynodon* results in an insignificant tree length increase of 8.7 steps (templeton test: *p* > 0.05). The topology within *Diplocynodon* differs to analyses 1.1 and 2.1, except that in all cases *D*. *remensis* is now the earliest diverging species, and a sister relationship between *D*. *darwini* and *D*. *deponiae* is consistently recovered. Constraining a monophyletic *Diplocynodon* also modifies the topology in non-crocodylian eusuchians. Principally, *Borealosuchus* is no longer immediately in the stem of Crocodylia, but is instead outside of Eusuchia (Fig. S3).

Nine synapomorphies diagnose *Diplocynodon* in the constrained and equal weighted analyses: (1) dorsally facing external nares (C41-0, ambiguous); (2) jugal anterior extent posterior to anterior frontal process (C63-1, ambiguous); (3) second premaxillary alveolus separated from 1st and close to 3rd (C145-1, ambiguous); (4) partial interlocking occlusion of dentary and maxillary teeth (C151-1, shared); (5) choanae shape sub-triangular, tapering posteriorly (C190-1, ambiguous); (6) splenial anterior perforation for cranial nerve V absent (C229-1, shared); (7) lingual foramen perforates surangular-articular suture (C253-1, shared); (8) anterolateral process present on dorsal midline osteoderms (C327-0, ambiguous); and (9) ventral osteoderms paired (C328-2, shared).

Furthermore, the sister relationship between *Leidyosuchus canadensis* and *Diplocynodon* is supported by eight synapomorphies in these analyses: (1) absence of transverse ridge between the orbits (C31-1, ambiguous); (2) external contact between the nasals and naris absent (C46-1, shared); (3) lateral margin of the orbit is at the same level or medial to the lateral margin of the maxilla at the level of alveoli 3–6 (C74-1, ambiguous); (4) frontoparietal suture linear (C76-1, ambiguous); (5) absence of a medial contact between the postorbital and quadratojugal at the dorsal corner of the infratemporal fenestra (C105-0, shared); (6) posterior extent of premaxillae on palate not reaching the level of 1 maxillary alveolus (C142-0, shared); (7) dentary alveoli 3 and 4 confluent (C217-0, shared); and (8) dentary symphysis adjacent to fewer than 6 full dentary alveoli (C221-0, shared).

Two of the eight synapomorphies of *Diplocynodon* recovered by Brochu (1999a) are also found here. The first, the presence of paired ventral osteoderms (C328-2), is present in all *Diplocynodon* species in which the relevant region is preserved, but it is also shared by Caimaninae and *Borealosuchus*. The second, perforation of the surangular-articular suture by the lingual foramen (C253-1), is also present (where preserved) in all *Diplocynodon* species but shared by most species of *Alligator* and most crocodyloids. The remaining six synapomorphies of Brochu (1999a) are either plesiomorphic for Crocodylia (characters 277 and 309), diagnostic of *Leidyosuchus* + *Diplocynodon* (characters 46 and 76), or potential synapomorphies of *Leidyosuchus* + *Diplocynodon* (character 215 and 264). These characters are briefly discussed below.

A deep and rounded iliac blade has long been considered an unambiguous synapomorphy of *Diplocynodon* (Brochu, 1999a; Martin et al., 2014). Because this character combines two independent anatomical features, it was here split into two, with one quantitative character defining the ‘depth’ of the postacetabular process (C309), and the other the degree of indentation on the dorsal margin of the iliac blade (C308). These modifications result in a much wider distribution of a deep iliac blade (C309-0), and the absence of a notch on the dorsal outline (C308-0), such that both are plesiomorphic for Crocodylia. A centrally positioned axial hypapophysis (C277-0) was similarly recovered as an unambiguous synapomorphy of *Diplocynodon* by Brochu (1999a). By contrast with previous studies, this condition is now recognised more widely in Alligatoroidea, for example including *Caiman yacare*, and several species of *Alligator* (*A*. *mcgrewi*, *A*. *prenasalis*, and *A*. *sinensis*). As a result, and because of the high proportion of missing data for this character across the tree, this feature is now recovered as the plesiomorphic condition in Crocodylia. Similar to Brochu (1999a), a prominent anterior process of the proatlas (C264-0) might diagnose *Diplocynodon*, but this cannot yet be determined because of missing data in several ‘basal’ *Diplocynodon* species and *Leidyosuchus canadensis*. Following Brochu (1999a) and subsequent studies, all species of *Diplocynodon* are scored as having a straight pterygoid-quadrate suture between the foramen ovale and basisphenoid exposure where preserved (215-1). As with the morphology of the proatlas, this is similarly recovered as a potential synapomorphy of *Leidyosuchus* + *Diplocynodon* because of missing data in the former taxon. A linear frontoparietal suture (76-1) unambiguously diagnosed *Diplocynodon* according to Brochu (1999a). This condition can be observed in most *Diplocynodon* species, with the exception of *D*. *darwini* and *D*. *ratelii*, in which it is absent. The condition is also present in *Leidyosuchus canadensis*, and thus it is recovered as an ambiguous synapomorphy of *Leidyosuchus* + *Diplocynodon* in Analysis 1.1 and in the constrained version of Analysis 1.3. Nasals that are excluded dorsally from the external naris (46-1) was recovered as an ambiguous synapomorphy of *Diplocynodon* by Brochu (1999a). This character has received significant modification here; most importantly it has been converted into a binary character describing the presence or absence of an external nasal-narial contact. Whereas under the previous character construction *Leidyosuchus* and *Diplocynodon* had different states, now they have the same condition, and this is an ambiguous synapomorphy uniting these two genera.

Seven new synapomorphies of *Diplocynodon* are recovered here; however, five are ambiguous, occurring multiple times within Alligatoroidea, or non-crocodylian eusuchians, and are not present in all members of *Diplocynodon* (C41-0, C63-1, C145-1, C190-1, C327-0). The remaining two are shared synapomorphies. The first of these shared synapomorphies, the presence of partial interlocking dentary and maxillary teeth (C151-1), is relatively uncommon in Crocodylia. This otherwise occurs in some ‘basal’ crocodyloids, *Borealosuchus sternbergii*, *Allodaposuchus precedens*, and one alligatoroid—*Caiman crocodilus*. The second, absence of an anterior perforation on the splenial (C229-1), occurs in most caimanines, where preserved, and almost all crocodyloids.

Two of the synapomorphies supporting *Leidyosuchus* + *Diplocynodon* have already been discussed (C46-1, C76-1). This clade was supported by a further six synapomorphies in analyses 1.1 and 2.1 and in the constrained version of Analysis 1.3. The postorbital and quadratojugal can be seen in contact medially at the dorsal corner of the infratemporal fenestra in all alligatoroids where preserved, except *Leidyosuchus* and *Diplocynodon*. Absence of a medial contact otherwise occurs only in Longirostres (C105-0). Confluence of dentary alveoli 3 and 4 (C217-0) also supports this clade, otherwise known only in *Borealosuchus* and *Eothoracosaurus mississippiensis*. In the unconstrained version of Analysis 1.3, the position of *Borealosuchus* means that this condition is instead recovered as the plesiomorphic condition in Crocodylia. *Leidyosuchus* and *Diplocynodon* share a short dentary symphysis adjacent to fewer than 6 alveoli (221-0). However, this condition also occurs in alligatorines, caimanines, crocodyloids, and non-crocodylian eusuchians. The short posterior extension of the premaxillae on the palate that characterises *Leidyosuchus* and *Diplocynodon* (142-0) is uncommon in Alligatoroidea, otherwise only occurring in *Paleosuchus trigonatus*. In general, this condition is uncommon in other crocodylians too, occurring only in *Toyotamaphimeia machikanensis*, *Asiatosuchus germanicus*, and some mekosuchines. The lateral margin of the orbit is at the same level or medial to maxillary alveoli 3–6 in *Leidyosuchus* and some *Diplocynodon* species (74-1). This character is relatively labile in Crocodylia; for example, it is optimised as independently evolving four times within Alligatoroidea under constrained Analysis 1.3. A transverse interorbital bridge (31-1) also ambiguously diagnoses *Diplocynodon* + *Leidyosuchus*. Once again, the character is labile within Alligatoroidea, occurring independently in caimanines and alligatorines. Furthermore, the bridge is absent in both *D*. *deponiae* and *D*. *darwini*. One additional feature, which is optimised as a potential synapomorphy of *Leidyosuchus* and *Diplocynodon*, is the presence of raised lateral and anterolateral walls of the choanae (195-2). This condition is exclusively found in this clade; however, since all other alligatoroids exhibit a different condition (195-1), which itself is different to the closest outgroups of Alligatoroidea (195-0), the ancestral condition for Alligatoroidea is uncertain.

In summary, the paraphyly of *Diplocynodon* is less a result of character support that teases the clade apart, and more a result of decreased support in previously proposed synapomorphies. As shown above, the synapomorphies that group *D*. *remensis* and the clade (*D*. *deponiae* + (*D*. *tormis* + *D*. *muelleri*)) with crownward taxa are few and ambiguous. However, only two of the eight characters previously recovered as diagnostic of *Diplocynodon* are recovered in unweighted and constrained analyses. The remainder are found more widely in Crocodylia or are recovered as ambiguous or potential synapomorphies of *Leidyosuchus* + *Diplocynodon*. Despite the number of new synapomorphies that support the monophyly of *Diplocynodon*, as well as *Leidyosuchus* + *Diplocynodon*, in unweighted and constrained analyses, these relationships are not supported under EIW. This suggests that characters capable of supporting a monophyletic *Diplocynodon* are down-weighted as a result of missing data or homoplasy. Indeed, characters supporting both the monophyly of *Diplocynodon* and a sister relationship with *Leidyosuchus* in equal weighted and constrained analyses are among the most homoplastic of all characters (Supplemental File 4). For example, characters 41, 63, 145, 229 and 253 all fall within the 20 most homoplastic characters in our data matrix. Furthermore, more than 50% of all taxa in our dataset cannot be scored for characters 229, 253, 327 and 328. Although characters supporting *Leidyosuchus* + *Diplocynodon* are scored for a greater number of taxa, they are also highly homoplastic.

#### The taxonomic content of Caimaninae

Seven synapomorphies diagnose Caimaninae in our study: (1) ratio of distal ischial blade expansion to ischium length = 0.54 (C26); (2) anterior frontal process at the same level or posterior to anterior orbit margin (C62-1, ambiguous); (3) width across dorsal supraoccipital exposure equal to or greater than half posterior parietal width (C78-1, ambiguous); (4) medial wall of the parietal bears foramina (C85-1, exclusive); (5) dentary alveoli 1–4 at the same level or higher than alveoli 11–12 (C218-0, shared); (6) anterior extent of the angular does not exceed the anteroposterior mid-length of the foramen intermandibularis caudalis (C257-1, ambiguous); and (7) eight nuchal osteoderms (C323-2, ambiguous).

Monophyly of the North American clade (*Brachychampsa*, *Ceratosuchus*, *Stangerochampsa*, *Wannaganosuchus*) is supported by five synapomorphies: (1) ratio of interorbital distance to anterior cranial table width = 0.27 (C6); (2) ratio of supratemporal fenestra length to cranial table length = 0.6 (C10); (3) supratemporal fenestra lateromedial width to anteroposterior length = 0.7–0.8 (C11); (4) anterior margin of the suborbital fenestra at the same level or posterior to the anterior orbit margin (C166-1, ambiguous); and (5) anterior extent of the ectopterygoid greater than or equal to two thirds of suborbital fenestra length (C174-1, shared). The clade (*Wannaganosuchus* + (*Stangerochampsa* + *Brachychampsa*)) is supported by two synapomorphies: (1) the presence a fossa on the lateral margin of the naris (C45-1, unambiguous); and (2) a sutural contact between the lacrimal and nasal (C53-0, shared). Finally, the sister relationship between *Stangerochampsa* and *Brachychampsa* is supported by four synapomorphies: (1) ratio of antorbital rostrum width to anterior cranial table width = 1.8 (C2); (2) anterior frontal process anterior to anterior orbital margin (C62-0, shared); (3) premaxilla-maxilla suture intersects lateral margin of incisive foramen (C137-2, shared); and (4) dentary profile shape between alveoli 4 and 10 curved (C220-1, shared).

Caimaninae is poorly recovered in Bootstrap and Jackknife replicates, but rather well supported by the Bremer decay index. The most compelling morphological support for the inclusion of a latest Cretaceous–Paleocene North American clade in Caimaninae is the presence of foramina on the medial parietal wall in *Brachychampsa montana* (C85-1) (Appendix 2: fig. 30H). Medial parietal perforations have hitherto supported the monophyly of South American caimanines (albeit ambiguously, due to non-preservation in some taxa) (Norell, 1988; Brochu, 1999a). Although the presence of these perforations can only be determined in *Brachychampsa* amongst these North American taxa, they are otherwise exclusively found in Caimaninae.

The angular does not exceed the anteroposterior mid-length of the foramen intermandibularis caudalis in *Stangerochampsa mccabei* and all South American caimanines, where preserved (C257-1), with the exception of *Mourasuchus arendsi*. This condition was also recovered as a synapomorphy of these North American taxa in Caimaninae by Bona et al. (2018). Outside of Caimaniane, this condition is otherwise only present in *Alligator mcgrewi* and *Mecistops cataphractus*; however, there is a considerable amount of missing data across the tree (64% of taxa), including all remaining members of this putative North American caimanine clade.

A short anterior frontal process, that does not exceed the anterior margin of the orbits (C62-1), is almost exclusively known in Caimaninae, including two North American taxa, *Ceratosuchus burdoshi* and *Wannaganosuchus brachymanus*. Otherwise, this condition only occurs in *Theriosuchus pusillus*, *Hylaeochampsa vectiana*, *Hassiacosuchus haupti*, and *Mekosuchus inexpectatus*. This character was introduced by Jouve (2004), and it has otherwise only been utilised in subsequent iterations of this data matrix (*e.g*. Jouve et al., 2008, 2015; Jouve, 2016). Jouve (2016) also scored this character state as characterising *Brachychampsa montana* and *Stangerochampsa mccabei*, which would add further support for this inclusion of the North American clade in Caimaninae; however, in both taxa the frontal is actually positioned slightly further anterior than the orbits (Wu, Brinkman & Russell, 1996, fig. 1A).

Unlike all other crocodylians, most caimanines are characterised by a large supraoccipital exposure, which excludes the parietal from the posterior margin of the cranial table (C78-2). By contrast, most other alligatoroids have a small supraoccipital exposure (C78-0). As previously recognised, *Brachychampsa montana* and *Stangerochampsa* lie between these extremes, with a large supraoccipital exposure that does not exclude the parietal from the posterior cranial table margin (C78-1) (Brochu, 2010). Ordering of this character such that the condition in *Brachychampsa* and *Stangerochampsa* is intermediate (C78-1) supports a close relationship between these taxa and Caimaninae.

An elevated dentary toothrow at the level of alveoli 1–4 that exceeds the height of the posteriormost dentary alveoli (C218-0) occurs in all members of Caimaninae where preserved, including all members of the North American clade. However, this condition is not unique to Caimaninae, occurring in almost all alligatoroids except for some ‘basal’ alligatorids and alligatorines, as well as all other crocodylians.

Knowledge of the number of nuchal osteoderms (C323) is restricted to extant and a few exceptionally preserved fossil taxa, and thus this character is only scored in 26 species. Nevertheless, all extant caimanines have eight nuchal osteoderms (C323-2), a feature shared by *Brachychampsa montana* (UCMP 133901) and convergently acquired in Gavialoidea (*e.g. Tomistoma schlegelii*, Appendix 2: fig. 132F). The condition in all other members of the North American clade is unknown. Extant alligators have six nuchal osteoderms (Appendix 2: fig. 132B–132C), which appears to be the plesiomorphic condition in Alligatoroidea, as exhibited by *Diplocynodon darwini* (*e.g*. HLMD-Me-10262).

A relatively wide distal expansion of the ischium (C26: 0.54) provides weak support for this clade. Although this character cannot be scored in most taxa, measurements in this dataset reveal that caimanines, including *Wanaganosuchus brachymanus*, have a relatively wider distal expansion than all other alligatoroids where preserved.

Two additional characters should be considered that supported the caimanine affinities of a similar North American clade in the analysis of Bona et al. (2018): (1) a dorsally facing naris (C41-1); and (2) absence of a prominent anterior process of the proatlas (C264-1). Here, the ancestral morphology of the naris in Caimaninae is ambiguous, given that it could be dorsally or anterodorsally facing. The absence of a prominent anterior process of the proatlas is similarly not recovered here as a synapomorphy of Caimaninae. As a result of modifications to character scores, the absence of an anterior process of the proatlas is plesiomorphic in Alligatoroidea, although appearing in some ‘basal’ alligatoroids such as *Diplocynodon*. Furthermore, the only taxon preserving the proatlas among the North American clade, *Brachychampsa montana*, exhibits a prominent process (C264-0). Thus, this character actually differentiates the North American clade from most members of the South American caimanines.

In summary, character support for Caimaninae including an early diverging North American clade is rather weak. Only one of the synapomorphies uniting a North American clade with South American caimanines is exclusive (C85-1), and it can only be scored in one of the North American taxa. Other synapomorphies follow a similar pattern. Although they are almost exclusively known in the South American caimanines, they are typically scored in two or fewer North American taxa (C62, C78, C257). Other characters are significantly more ambiguous (C218) or exhibit a significant amount of missing data (C26, C323), undermining the support for this clade.

#### The phylogenetic affinities of Eocaiman

*Eocaiman* is an alligatoroid genus based on fragmentary remains from the Paleocene–Eocene of South America. Three species have been described: *E*. *cavernensis* (Simpson, 1933) and *E*. *palaeocenicus* (Bona, 2007) are included here, whereas *E*. *itaboraiensis* was not included, as the holotype and referred material comprise phylogenetically uninformative fragments (Pinheiro et al., 2013). By strong contrast to its typical placement as one of the earliest diverging members of traditional (*i.e*. South American) caimanines (Brochu, 1999a; Bona, 2007; Brochu, 2010, 2011; Brochu et al., 2012; Fortier et al., 2014; Salas-Gismondi et al., 2015; Bona et al., 2018; Cossette & Brochu, 2018; Souza-Filho et al., 2018; Cidade, Fortier & Hsiou, 2020; Godoy et al., 2020), our study recovers *Eocaiman* in the stem of Alligatoridae instead. This placement is surprising, as the spatiotemporal distribution of *Eocaiman* fits well with its usual placement as a ‘basal’ member of Caimaninae. Nevertheless, the reasons for the exclusion of *Eocaiman* from Caimaninae can be recognised by comparing character support between this and previous studies.

Analyses based on the datasets of Brochu (1999a) and Brochu et al. (2012) tend only to include the type species of *Eocaiman* (*E*. *cavernensis*). These studies recover many of the following traditional synapomorphies of Caimaninae in *E*. *cavernensis* (character numbers from Brochu et al. (2012)): (1) a splenial excluded from the dentary symphysis with the anterior tip passing dorsal to the Meckelian fossa (C54-2); (2) surangular-angular suture passing broadly along the ventral margin of the external mandibular fenestra (C60-1); (3) a large supraoccipital exposure blocking the parietal from the posterior margin of the skull table (C158-3); and (4) exoccipitals with slender ventral processes that contact the basioccipital tubera (C174-2). By contrast, most of these are not recovered as synapomorphies supporting the placement of *Eocaiman* in Caimaninae in our study, as discussed below.

*Eocaiman cavernensis* is known only from the holotype (AMNH 3158), which preserves portions of both mandibular rami, as well as a fragmentary skull missing the cranial table (Godoy et al., 2020). The supraoccipital is not preserved, therefore any description of its size in *E*. *cavernensis* must instead be based on an undescribed specimen (AMNH 19170) catalogued as *Eocaiman* sp. This specimen was recovered from an unspecified late Eocene locality in Mendoza Province, Argentina, some 500 km from the type locality of *E*. *cavernensis*.

A surangular-angular suture that passes broadly along the ventral margin of the external mandibular fenestra was recovered as an unambiguous synapomorphy of South American Caimaninae, including *E*. *cavernensis*, by previous authors (Brochu, 1999a; Brochu et al., 2012; Narváez et al., 2016). Here, this condition is no longer recovered in many caimanines, including *Eocaiman*, which instead share a posterodorsal intersection of the suture that is recovered as plesiomorphic in Crocodylia.

Slender, descending processes of the exoccipitals that reach the basioccipital tubera have been recovered as an unambiguous synapomorphy of South American Caimaninae (Brochu, 1999a). According to Brochu (1999a), these processes do not actually contact the tubera in Caimaninae, but they extend slightly further ventrally than most other crocodylians. The distinction between this condition, and that exhibited by most crocodylians lacking contact between the exoccipitals and basioccipital tubera, was considered too vague in this study. Instead, the character has been simplified to describe absence or presence of contact between the exoccipitals and basioccipital tubera (C127). However, irrespective of whether this distinction is valid, neither the holotype nor AMNH 19170 appear to preserve this portion of the occiput.

We concur with previous studies that *E*. *palaeocenicus*, *E*. *cavernensis*, and South American members of Caimaninae share a rostral tip of the splenial that is positioned dorsal to the meckelian fossa. In other studies, this condition united *Eocaiman* with other caimanines. Here, the original character has been reductively coded, and resultantly this character no longer diagnoses the clade. Furthermore, the same condition is present in all species of *Alligator* that lack a splenial symphysis, and so this condition does not unambiguously diagnose South American Caimaninae either.

An examination of the character scores in the recently published dataset of Cidade, Fortier & Hsiou (2020) supports all of the aforementioned observations. The only exception is character 54 of Brochu et al. (2012), which was not reductively coded, in contrast with our study. Despite these changes, Cidade, Fortier & Hsiou (2020) still recovered *Eocaiman* as an early diverging member of South American Caimaninae. This rests on two synapomorphies in their study. The first, as expected, pertains to the rostral tip of the splenial, which was delimited as originally described by Brochu (1999a). The second is the intersection of the surangular-angular suture dorsal to the ventral tip of the articular, which was scored as present in *Eocaiman palaeocenicus*. This condition is recovered almost exclusively in South American caimanines here, constituting a robust synapomorphy of the clade; however, this region of the mandible is too poorly preserved in *E*. *palaeocenicus* to determine its morphology (MPEF 1933a, and all referred material (Appendix 1)).

In this study, only two of the characters comprising synapomorphies of Caimaninae could be scored in *Eocaiman*. In both species of *Eocaiman* included, the height of the dentary at the level of alveoli 1–4 is dorsoventrally lower than alveoli 11–12 (C218-1, Appendix 2: fig. 93). Where preserved, all species of Caimaninae exhibit the opposite condition, in which the anterior dentary alveoli are approximately at the same level as the posterior alveoli (C218-0). This character has been included in relatively few phylogenetic analyses, within which this feature is always recovered as an autapomorphy of *Eocaiman* within South American Caimaninae (Bona, 2007; Pinheiro et al., 2013; Cidade et al., 2017; Cidade, Fortier & Hsiou, 2020; Souza-Filho et al., 2018). By contrast to these studies, the derived condition is here also found in most alligatorines, contributing to the exclusion of *Eocaiman* from Caimaninae.

South American caimanines are diagnosed as having 13 maxillary alveoli, but *E*. *cavernensis* has 14. Since the number of maxillary alveoli can vary intraspecifically within crocodylians by 1–2 alveoli (Iordansky, 1973), and several South American caimanines have 14 maxillary alveoli, this character does not strongly support the exclusion of *Eocaiman* from the South American caimanine clade.

The morphology of the posterolateral surface of the mandible is newly characterised in this study (C240), and it provides stronger support for the exclusion of *Eocaiman* from South American caimanines. In *E*. *palaeocencius*, there is a broad, unornamented exposure of the angular ventral to the retroarticular process for attachment of M. pterygoideus ventralis (C240-0) (Appendix 2: fig. 106G). This condition is shared by all alligatorines, several ‘basal’ alligatoroids, and the North American ‘basal’ caimanine clade. By contrast, all South American caimanines lack this unornamented exposed surface (C240-1) where preserved.

Two morphological features of *Eocaiman cavernensis* support a position within Caimaninae. These features are shared almost exclusively by caimanines, but they are most parsimoniously optimised as being convergent in our ‘main’ topology. Firstly, a feature previously unrecognised in *E*. *cavernensis* is a prominent spectacle (C32-1). Although the presence of a low spectacle was recovered as an ambiguous synapomorphy of Alligatoroidea here, a tall spectacle is almost exclusively known in South American caimanines. Despite the inclusion of this anatomical feature as a character in several studies (Barrios, 2011; Cidade et al., 2017; Cidade, Fortier & Hsiou, 2020; Souza-Filho et al., 2018), these did not recognise its presence in *E*. *cavernensis*. Secondly, the dorsal jugal margin has a characteristic sharp, orthogonal step (C94-2) (Appendix 2: fig. 36C) in those South American caimanines in which the relevant region is preserved, which is otherwise only known in *E*. *cavernensis*.

Two constrained analyses were performed to test the newly recovered position of *Eocaiman* (Table S3). In the first, both species of *Eocaiman* were forcibly recovered in Caimaninae, as defined in Analysis 1.3. In the second, they were forcibly recovered in the South American caimanine clade. Both constrained searches resulted in small and insignificant tree length increases. Forcing *Eocaiman* into Caimaninae results in a polytomy in which this genus is the earliest diverging caimanine lineage (Fig. S4). By contrast, forcing *Eocaiman* into the South American clade results in major topological differences (Fig. S5). Most notably, the North American caimanines are instead recovered in the stem of Alligatoridae. A more traditional Caimaninae is recovered, in which the stem consists of a paraphyletic *Eocaiman*, as well as two taxa otherwise recovered in the stem of Alligatoridae: *Procaimanoidea utahensis* and *Arambourgia gaudryi*.

In summary, *E*. *cavernensis* and *E*. *palaeocencius* are most parsimoniously recovered outside of Caimaninae here. This is surprising given that all alligatoroid taxa described from South America to date have been phylogenetically recovered within Caimaninae (Cidade, Fortier & Hsiou, 2019). This novel position is partly the result of modifications to character scores that until now supported its position in Caimaninae. Furthermore, there are a number of morphological features that indicate that *Eocaiman* lies outside of Caimaninae (*i.e*. dentary height, and morphology of the angular). On the other hand, all three species of *Eocaiman* are known from extremely limited material, such that most of the character states diagnostic of Caimaninae and less inclusive clades cannot be scored. Some of the character states that appear to draw *Eocaiman* outside of the South American caimanine clade (*e.g*. exposure of the fossa for M. pterygoideus ventralis on the angular) are plesiomorphic for Alligatoridae, also occurring in the North American caimanines. As such, these might be expected to occur in the earliest South American caimanines too. Constrained searches that force *Eocaiman* into the South American caimanine clade result in statistically insignificant tree length increases, as a result of the shared presence of some morphological features that are otherwise almost exclusively known in South American caimanines. The position of *Eocaiman* outside of Caimaninae is therefore only tentatively accepted, and this hypothesis will need to be re-tested once more complete material becomes available.

#### The phylogenetic affinities of Protocaiman peligrensis

*Protocaiman peligrensis* was recently described from the early Paleocene of Argentina (Bona et al., 2018). Its age and provenance makes it another good candidate for one of the most ‘basal’ South American caimanines. Indeed, the sole previous phylogenetic analysis to include this taxon recovered it as the earliest diverging member of the (primarily) South American caimanine clade (Bona et al., 2018). By contrast, we recover *Protocaiman* in a more nested position within this caimanine clade, in a polytomy with the contemporaneous species *Necrosuchus ionensis* (Brochu, 2011), as well as *Bottosaurus harlani* from the latest Cretaceous–early Paleocene of the USA (Cossette & Brochu, 2018; Cossette, 2021).

As in Bona et al. (2018), the inclusion of *Protocaiman* in the South American caimanine clade is here supported by: (1) supratemporal fenestrae with overhanging rims (C81-1); and (2) the presence of medial parietal foramina (C85-1) (although the latter is recovered as diagnostic of all caimanines here). In Bona et al. (2018), a sister relationship between *Protocaiman* and all other South American caimanines was supported by the presence of a small supraoccipital exposure (C78-0), contrasting with the large exposure (blocking the parietal from the posterior margin of the cranial table) that characterises most members of the South American clade (C78-2). By contrast, the same character supports the least inclusive clade comprising *Protocaiman* and *Paleosuchus*. A large supraoccipital exposure that does not block the parietal (C78-1) is recovered as a synapomorphy of this clade, that is secondarily lost in *Protocaiman* (C78-0). This alternative optimisation appears to be the result of character ordering, with the earliest diverging South American caimanine recovered here, *Gnatusuchus pebasensis*, characterised by a very large supraoccipital exposure (C78-2). Placing *Protocaiman* ‘basal’ to this taxon, as in Bona et al. (2018), would increase the number of transformations for this character by two steps.

Another important difference in our study to that of Bona et al. (2018) was the use of geometric morphometric characters describing the dorsal skull table morphology in the latter. Those authors reported that the optimisation of these morphometric characters revealed similarities between *Protocaiman* and North American caimanines, drawing *Protocaiman* to the ancestral node of Caimaninae. Although linear morphometric features of the skull table are incorporated as continuous characters here, they do not capture the same details of the skull table as landmark data. Two additional characters support the inclusion of *Protocaiman* in a crownward position among caimanines. *Protocaiman* exhibits very slightly upturned orbital margins (72-1), which are present in all members of the least inclusive clade comprising *Caiman lutescens* and *Paleosuchus palpebrosus*. By contrast, the North American caimanines, as well as *Gnatusuchus*, *Kuttanacaiman* and *Globidentosuchus* (all recovered at the ‘base’ of this derived clade), exhibit flush orbital margins (C72-0). A concavo-convex frontoparietal suture (C76-0) also supports the position of *Protocaiman* in this ‘derived’ clade, although this synapomorphy is ambiguous given the absence of this condition in several members (*Paleosuchus*, *Tsoabichi*, *Bottosaurus*). Furthermore, this character state is highly homoplastic, occurring in the early branching North American caimanines, as well as some ‘basal’ South American caimanines.

#### The phylogenetic affinities of Protocaiman, Eocaiman, Necrosuchus and Bottosaurus

*Protocaiman peligrensis*, *Necrosuchus ionensis*, and *Eocaiman palaeocenicus* were all recovered from the early Paleocene Salamanca Formation of Chubut Province, Argentina. By contrast, *Eocaiman cavernensis* was recovered some 80 km away, in the middle to late Eocene of the Sarmiento Formation (Bona & Barrios, 2015). The close proximity and scarcity of overlapping material between some of these specimens has raised the possibility of their synonymy (Brochu, 2011; Bona et al., 2018). *Necrosuchus* and the *Eocaiman* species are known from anatomically overlapping mandibular remains. In addition to differences in their gross morphology (Brochu, 2011), the distinction between these taxa is supported by phylogenetic analyses. Both the phylogenetic analysis of Bona et al. (2018) and our own also supports the distinction between *Protocaiman* and *Eocaiman*; however, the lack of overlapping material between these taxa adds uncertainty.

According to Bona et al. (2018), *Necrosuchus* and *Protocaiman* also lack overlapping remains, but the recovery of these taxa in different parts of their tree was used to support their distinctness. However, Bona et al. (2018) appear to have overlooked the shared preservation of the quadrate condyles in these two taxa (Brochu, 2011, fig. 1; Bona et al., 2018, fig. 1), from which additional features are recognised in this study. The polytomy in our analyses, comprising *Necrosuchus ionensis*, *Protocaiman peligrensis*, and *Bottosaurus harlani*, is supported by one ambiguous synapomorphy: a large notch on the dorsomedial edge of the quadrate condyle (C118-1). This condition is not recovered in any other member of the South American caimanine clade, which almost exclusively exhibit a restricted, laterally inset notch (C118-2). The condition in *Protocaiman* and *Necrosuchus* is recovered as plesiomorphic for Crocodylia, retained in all ‘basal’ alligatoroids, most alligatorines, and the North American caimanines. Although one might expect this to support a ‘basal’ position of *Necrosuchus* and *Protocaiman* within Caimaninae, it is here more parsimoniously recovered as an atavism within the crown group of caimanines. This is the only similarity between the overlapping remains of *Necrosuchus* and *Protocaiman*, and a second feature of the quadrate condyle actually distinguishes them. Whereas the dorsal and ventral margins of the quadrate condyle taper medially in *Protocaiman* (as they do in most caimanines) (C119-1) (Appendix 2: fig. 45), they are sub-parallel in *Necrosuchus* (119-0) (Brochu, 2011, fig. 1E), which is the plesiomorphic condition in Alligatoroidea. The third member of this polytomy is *Bottosaurus harlani*, but the only synapomorphy uniting this clade (C118-1) cannot be scored in this North American taxon. Previous studies have recovered *Bottosaurus* as a close relative of *Paleosuchus* and the early Eocene North American species, *Tsoabichi greenriverensis* (Cossette & Brochu, 2018; Cidade, Fortier & Hsiou, 2020; Cossette, 2021).

#### The taxonomic content of Jacarea

Jacarea was first applied by Norell (1988) to include the three extant species of *Caiman* (*C*. *latirostris*, *C*. *crocodilus*, and *C*. *yacare*) and *Melanosuchus niger*, as distinguished from the two species of the dwarf caiman genus *Paleosuchus*. Subsequently, Brochu (1999a) formally defined this taxon as the last common ancestor of *Caiman* and *Melanosuchus*, and all of their descendants. Among fossil taxa, Jacarea has typically included South American caimanines with a general caiman-like morphology, such as *Caiman brevirostris* and *Caiman wannlangstoni* (Brochu, 2011; Salas-Gismondi et al., 2015; Bona et al., 2018; Cossette & Brochu, 2018; Souza-Filho et al., 2018). Although poorly supported, the traditional composition of Jacarea is lost in this study as a result of the deeply nested position of *Paleosuchus*, the polyphyly of the genus *Caiman*, and the general pattern of successive branching found across the South American caimanine clade (Fig. 12). Cidade, Fortier & Hsiou (2020) also recovered a topology in which *Necrosuchus* was deeply nested within Caimaninae, with possible affinities to Jacarea, although they were unable to resolve whether it belonged in the latter clade.

Analysis 1.3 was constrained to recover Jacarea as traditionally defined, allowing several OTUs that have been recovered in the group in previous analyses to float (*Caiman brevirostris*, *Caiman wannlangstoni*, UCMP 39978, *Purussaurus*, *Acresuchus*, and *Necrosuchus*). The resultant three MPTs were insignificantly longer by 19.3 steps, and their consensus reveals major topological differences to the unconstrained tree (Fig. S6). All floating taxa are recovered in Jacarea, except *Necrosuchus*, which along with *Protocaiman* forms a polytomy outside of the crown group of caimanines. Considering only extant taxa, *Paleosuchus* is the sister taxon to Jacarea, consistent with the topology from molecular analyses. Also different to the unconstrained analysis is the exclusion of the North American clade from Caimaninae, which instead is nested in Alligatorinae. *Eocaiman* remains outside of Caimaninae, but it is paraphyletic along the stem of Alligatorinae.

#### The phylogenetic affinities of Caiman lutescens (MACN PV 13551)

**Figure 13:**
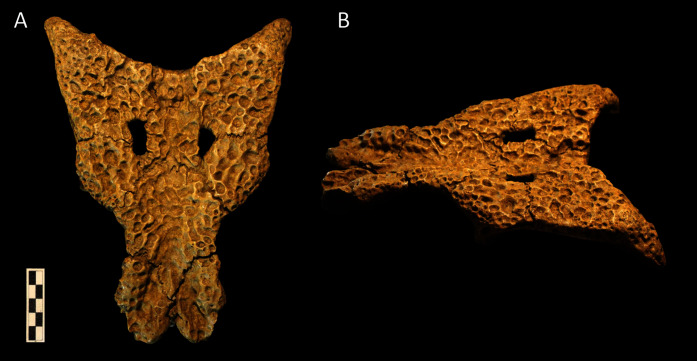
*Caiman lutescens* (MACN PV 13551) skull in (A) dorsal view, and (B) dorsolateral view.

*Caiman lutescens* was erected based on fragmentary remains from late Miocene deposits in northeastern Argentina (Rovereto, 1912; Bona & Barrios, 2015). The type material, which consists of a skull table (MACN PV 13551), a large fragment of the rostrum (MACN PV 5416), and numerous vertebrae and dentary fragments, is no longer considered to represent a single individual or even a single species (Langston, 1965; Brochu, 1999a; Bona, Riff & Gasparini, 2012; Bona & Barrios, 2015). The rostral fragment (MACN PV 5416) is now referred to *Caiman latirostris* (Bona, Riff & Gasparini, 2012), whereas the mandibular and postcranial elements belong to an indeterminate crocodylian. As such, *Caiman lutescens* is represented only by a skull table (MACN PV 13551) (Fig. 13). Nevertheless, despite its incomplete nature, MACN PV 13551 preserves numerous features diagnostic of South American caimanines: (1) a large supraoccipital exposure, blocking the parietal from the posterior margin of the cranial table (C78-2); (2) overhanging rims of the supratemporal fenestrae (C81-1); (3) a reduced anterior extent of the frontal process (C62-1); (4) interconnected prefrontals (C59-1); and (5) a tall interorbital spectacle (C32-1).

**Figure 14:**
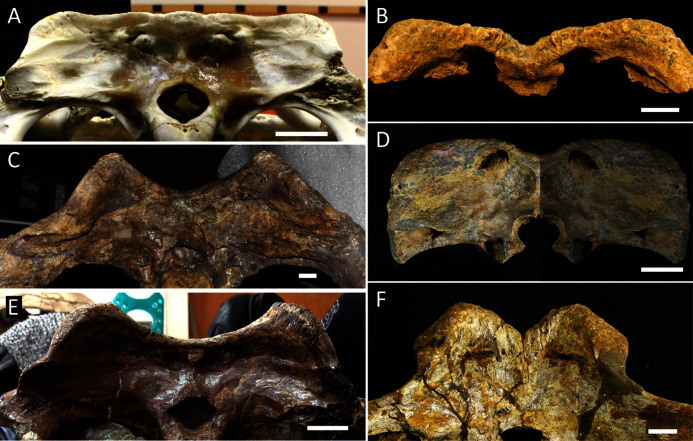
Occipital view of the cranium showing variation in morphology of the posterior cranial table margin. (A) *Caiman yacare* (AMNH 97300); (B) *Caiman lutescens* (MACN PV 13551); (C) *Purussaurus neivensis* (UCMP 39704); (D) *Caiman gasparinae* (right hand side mirrored for clarity) (MLP-IV-15-1); (E) *Purussaurus neivensis* juvenile (IGM DHL-45); (F) *Mourasuchus arendsi* (UFAC 1431). All scale bars = 20 mm.

**Figure 15:**
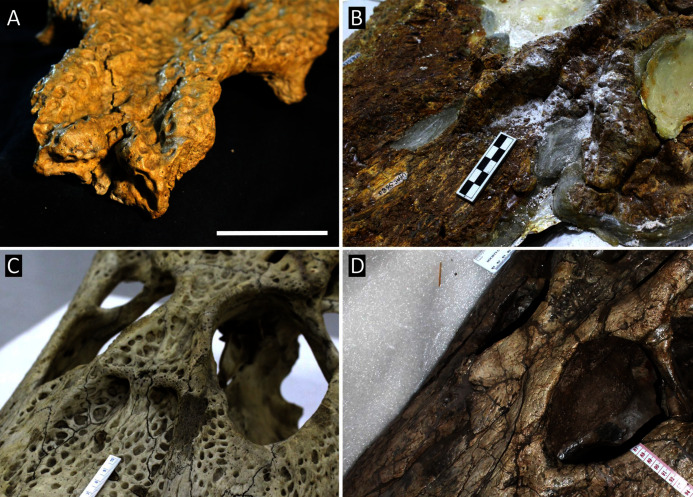
Anterolateral view of the rostrum showing morphology of the interorbital bridge (spectacle). (A) *Caiman lutescens* (MACN PV 13551); (B) *Mourasuchus arendsi* (UFAC 5883); (C) *Melanosuchus niger* (NHMUK 45.8.25.125); (D) *Purussaurus neivensis* (UCMP 39704). Scale bar in A = 50 mm.

In our analysis, *Caiman lutescens* is recovered as the sister taxon to *Caiman gasparinae* + *Mourasuchus*. This relationship is weakly supported by two synapomorphies: (1) ratio of the interorbital distance to anterior cranial table width = 0.4–0.5 (C6); and (2) presence of an acute dorsal indentation on the supraoccipital (C82-1, shared). The sister relationship between *Caiman gasparinae* and *Mourasuchus* is supported only by the ratio of anteroposterior supratemporal fenestra length to cranial table length = 0.3 (C10). Brochu (1999a) suggested close affinities between *Caiman lutescens* and *Purussaurus* or *Mourasuchus*, which are among the largest and most unusual appearing crocodylians known (Price, 1964; Langston, 1966; Gasparini, 1985). The skull table of *Caiman lutescens* indeed belonged to a large animal: its dimensions are greater than all extant *Caiman* species examined here. However, they are approximately equivalent to *Acresuchus* (UFAC 2507), *Mourasuchus amazonensis* (UFAC 1424), adult *Melanosuchus niger* (*e.g*. NHMUK 45.8.25.125), and *Caiman gasparinae* (MLP IV-15-1), and smaller than the largest specimens of *Purussaurus* (*e.g*. UFAC 2507, UCMP 39704) (Table 6), although we cannot be sure of the ontogenetic stage of *Caiman lutescens*. Further similarities between *Caiman lutescens*, *Purussaurus*, and *Mourasuchus* include a wide interorbital distance, and a thickening of the medial orbital margin. The interorbital width is continuously scored in this study, and it is recovered as a synapomorphy supporting the clade (*Caiman lutescens* + (*Caiman gasparinae* + *Mourasuchus*)), but *Purussaurus* exhibits a nearly equivalent interorbital distance. Most alligatoroids exhibit upturned orbital margins; however, in *Caiman lutescens* the upturned margins are thick and rugose, again similar to *Purussaurus* and *Mourasuchus*. Several authors have recognised that the posterior cranial table margin of *Caiman lutescens* is broadly ‘U’ shaped across its length (Langston, 1965; Brochu, 1999a; Bona, Riff & Gasparini, 2012) (Fig. 13), a condition otherwise known only in *Purussaurus* and *Acresuchus*. Bona, Riff & Gasparini (2012) also considered the skull table to be deeply concave about the sagittal line in *Caiman lutescens* (Fig. 14B), another feature known only in *Purussaurus* and *Acresuchus*. By contrast with Bona, Riff & Gasparini (2012), we regard the morphology of the skull table of *Caiman lutescens* as distinct from that of *Purussaurus* (Fig. 14C). *Caiman lutescens* exhibits a deep indentation on the skull table that is restricted to the sagittal line (82-1) (Figs. 13B, 14B). By contrast, in both *Purussaurus* and *Acresuchus*, the remainder of the skull table is relatively flat. This indentation is also found in *Caiman gasparinae* (Fig. 14D), *Mourasuchus* (Fig. 14F), and *Caiman latirostris*, although it does not extend as far anteriorly in these taxa as it does in *Caiman lutescens*. Strongly overhanging rims of the supratemporal fenestrae, as well as a tall spectacle with a deep posterior fossa (Fig. 15), also distinguish *Caiman lutescens* (Fig. 15A) from *Acresuchus* and *Purussaurus* (Fig. 15B), but they unite it with *Mourasuchus* and *Caiman gasparinae*.

**Table 6:** Comparisons of cranial measurements in selected caimanine taxa.

Taxon	Specimen	Skull length	ST anterior width	ST posterior width	ST anteroposterior length	IO width
*Caiman lutescens*	MACN-PV 13551	–	132	158	79	61
*Acresuchus pachytemporalis*	UFAC 2507	550	104	122	83	35
*Caiman gasparinae*	MLP 73-IV-15-1	–	114	73	88	51
*Caiman latirostris*	MACN 1420	320	82	99	54	28
*Caiman yacare*	MACN unnumbered	321	80	91	53	26
*Melanosuchus niger*	NHMUK 45.8.25.125	630	122	156	100	39
*Mourasuchus arendsi*	MLP 73-IV-15-8	–	114	125	65	55
*Mourasuchus* cf. *amazonensis*	UFAC 1424	~1,120	–	–	90	–
*Purussaurus brasiliensis*	UFAC 1403	1,420	315	330	165	170
*Purussaurus neivensis*	UCMP 39704	990	206	188	156	83

**Note:**

ST, Skull table; IO, Inter-orbital. All measurements in millimetres.

Despite these similarities, several features of the cranial table clearly distinguish *Caiman lutescens* from *Mourasuchus*. Most notably, *Caiman lutescens* lacks the squamosal horns characteristic of all *Mourasuchus* species, where preserved (Cidade et al., 2019b). *Mourasuchus arendsi* and *Mourasuchus amazonensis* also exhibit subparallel lateral cranial table margins, unlike the posteriorly divergent margins of *Caiman lutescens*. The anterior corners of the cranial table formed by the postorbitals are sharp in *Mourasuchus arendsi* and *Mourasuchus amazonensis*, but they are smoothly curved and crescentic in *Caiman lutescens*. Finally, *Mourasuchus arendsi* and *Mourasuchus amazonensis* exhibit a sagittal ridge on the parietal, which is absent in *Caiman lutescens*.

In summary, *Caiman lutescens* shares features in common with *Mourasuchus* and *Purussaurus*, but it lacks apomorphic features of both taxa. Morphological characters supporting a sister relationship between *Caiman lutescens* and (*Caiman gasparinae* + *Mourasuchus*) are few, but diagnostic. Among alligatoroids, the indentation of the supraoccipital (C82-1) is otherwise only known in *Caiman latirostris*; however, the anterior extent of the indentation is unique to *Caiman lutescens*. *Caiman lutescens* represents a distinct species, but the discovery of more complete material is needed to further test its phylogenetic affinities with Caimaninae.

#### The phylogenetic affinities of UCMP 39978

**Figure 16:**
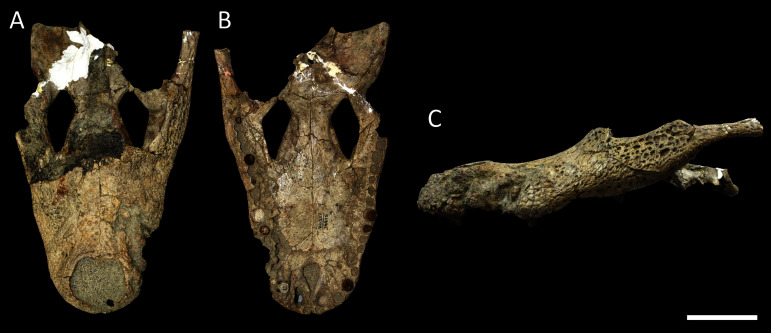
UCMP 39978 (‘*Caiman* cf. *lutescens*’) skull in (A) dorsal; (B) ventral; and (C) left lateral views. Scale bar = 50 mm.

UCMP 39978 is a partial skull missing the cranial table, from the middle Miocene Honda Group of Colombia (Fig. 16). Langston (1965) identified this specimen as *Caiman* cf. *lutescens* based on similarities with MACN PV 5416, which was assigned to *Caiman lutescens* at the time (see above). Now only one overlapping bone (the supraoccipital) exists between UCMP 39978 and *Caiman lutescens*, and by itself it does not preserve any diagnostic features. Nevertheless, these taxa are most likely not synonymous. In addition to their geographic and temporal separation, *Caiman lutescens* exhibits highly divergent features reminiscent of *Mourasuchus* or *Purussaurus*, but UCMP 39978 exhibits a generalised *Caiman* morphology (Fig. 16). Despite referring the material to *Caiman* cf. *lutescens*, Langston (1965) recognised similarities between UCMP 39978 and the extant species *Caiman latirostris*, suggesting the latter might be descended from UCMP 39978, with only a few evolutionary changes. Among the similarities he noted were the presence of rostral ridges, the overall proportions of the skull, and the large palatine exposure.

Brochu (1999a) was the first to test the affinities of UCMP 39978 phylogenetically and recovered it in a polytomy with *Caiman latirostris* and *Melanosuchus*. More recently, Bona, Riff & Gasparini (2012) recovered the specimen in a similar polytomy, also including *Caiman crocodilus*, *Caiman yacare*, and *Caiman gasparinae*. Nevertheless, they referred UCMP 39978 to *Caiman latirostris* based on several of the features listed by Langston (1965), in addition to the wide nasals and maxillae, and a large opening of the external naris. After the discovery of several new caimanines from the middle Miocene of Peru, Salas-Gismondi et al. (2015) noted similarities between UCMP 39978 and *Caiman wannlangstoni*, and Scheyer & Delfino (2016) listed UCMP 39978 as aff. *Caiman wannlangstoni*. Nevertheless, the most recent phylogenetic analyses of Caimaninae have continued to recover UCMP 39978 in a polytomy with most species of *Caiman* and *Melanosuchus* (Cidade, Fortier & Hsiou, 2020; Souza-Filho et al., 2018).

In our analysis, a weakly supported sister relationship is recovered between UCMP 39978 and *Caiman wannlangstoni*, based on one shared synapomorphy: the lateral margin of the orbit is situated further laterally than that of the maxilla at the level of alveoli 3–6 (C74-0). UCMP 39978 is recovered more distantly related to *Caiman latirostris*, and several morphological features argue against its referral to that taxon. For example, by contrast with *Caiman latirostris*, UCMP 39978 lacks an anterior invagination of the palatine process as well as a nasal-lacrimal contact, and it exhibits a highly enlarged external naris.

Salas-Gismondi et al. (2015) diagnosed *Caiman wannlangstoni* based on a combination of 11 characters. All but one (sinuosity of maxilla lateral margin) were discretised as morphological characters in our analysis. UCMP 39978 exhibits four of these diagnostic features: (1) sinuous lateral margins of the maxilla; (2) prominent rostral ridges (C27-1); (3) a fossa on the anterior margin of the choanae (C197-1); and (4) a broad maxillary shelf at the anterolateral margin of the suborbital fenestra (C168-1). However, these features provide only weak support for the referral of UCMP 39978 to *Caiman wannlangstoni*, given that they are found in other South American caimanines too. The ‘strongly sinuous’ lateral rostral margins of *Caiman wannlangstoni* do not appear significantly more sinuous than most South American caimanines. Furthermore, prominent rostral ridges are also present in *Caiman brevirostris*, *Caiman latirostris*, *Melanosuchus niger*, and *Purussaurus neivensis*. Although UCMP 39978 and *Caiman wannlangstoni* do share a deep fossa excavating the anterior margin of the choanae, this appears to be of limited diagnostic use, given that such a fossa appears in several large, osteologically mature crocodylians. It commonly occurs in mature *Crocodylus* species, but the condition has also been observed in large individuals of *Caiman latirostris* (MACN V 1420) and *Melanosuchus niger* (NHMUK 45.8.25.125). Furthermore, a broad maxillary shelf on the anterolateral margin of the suborbital fenestra is present in all alligatorids where preserved. Although this condition is accentuated in *Caiman wannlangstoni* and UCMP 39978, it is not discernible from *Caiman latirostris* or *Melanosuchus niger*.

Several anatomical features directly refute the referral of UCMP 39978 to *Caiman wannlangstoni* (Salas-Gismondi et al., 2015). Unlike *Caiman wannlangstoni*, UCMP 39978 lacks globular posterior dentition (otherwise only known in *Caiman brevirostris*) and anterodorsally facing nares (absent in all other caimanines). Furthermore, whereas the rostrum is vaulted in *Caiman wannlangstoni*, in UCMP 39978 the rostrum has a height to width ratio comparable to most other caimanines. An additional feature that distinguishes UCMP 39978 from *Caiman wannlangstoni* is the absence of a nasal-lacrimal contact and the absence of a maxillary process in the lacrimal in UCMP 39978. Finally, although the size of the external naris was not discretised, it is much larger in proportion to the premaxillae in UCMP 39978 than all other South American caimanines (except *Purussaurus brasiliensis* (UFAC 1403)).

In summary, we support previous rejections of the referral of UCMP 39978 to *Caiman lutescens*. Whereas UCMP 39978 exhibits a generalised caimanine morphology, the skull table of *Caiman lutescens* indicates that it was a close relative of *Purussaurus* or *Mourasuchus*. Furthermore, the referral to *Caiman latirostris* is rejected based on the presence of several autapomorphies in UCMP 39978, pertaining to the absence of a nasal-lacrimal contact and invagination of the palatine process, as well as the extreme enlargement of the naris. Finally, although UCMP 39978 might be closely related to *Caiman wannlangstoni* (Scheyer & Delfino, 2016), we do not consider them to be conspecific given the absence of several autapomorphies, namely the globular posterior dentition and anterodorsally facing nares that characterise *Caiman wannlangstoni* (Salas-Gismondi et al., 2015). As such, UCMP 39978 likely warrants a new species name, although that is beyond the scope of this study.

### Anatomical support and implications for the systematics of Planocraniidae

Like *Borealosuchus*, Planocraniidae (comprising *Boverisuchus* and *Planocrania*) is typically recovered as a stem brevirostrine, commonly as the sister clade of Alligatoroidea + Crocodyloidea (*e.g*. Puértolas-Pascual, Canudo & Cruzado-Caballero, 2011; Brochu et al., 2012; Brochu, 2012; Salas-Gismondi et al., 2016; Mateus, Puértolas-Pascual & Callapez, 2019). However, this clade has also been recovered in a ‘basal’ crocodyloid position (Jouve et al., 2015), as well as in a polytomy with Alligatoroidea and Crocodyloidea (Jouve, 2016). Furthermore, Groh et al. (2020) recovered Planocraniidae as a polyphyletic group, with *Boverisuchus vorax* recovered as a stem brevirostrine and *Planocrania* as a crocodyloid. By contrast, some combined analyses suggest that Planocraniidae is the sister clade to Crocodylia (*e.g*. Gatesy et al., 2003; Lee & Yates, 2018). The sister relationship between Planocraniidae and Longirostres recovered here is weakly supported (Jackknife = 14, Bootstrap = 4, Bremer = 3.4), but is similar to the results of the combined analysis of Iijima & Kobayashi (2019). Only three synapomorphies support Planocraniidae + Longirostres: (1) degree of flare in the scapula blade = 23–31° (C21); (2) foramen aerum on the dorsomedial corner of the quadrate condyle (C117-0, shared); and (3) ectopterygoid-maxilla suture parallel and adjacent to posterior maxillary alveoli (C175-1, ambiguous).

There is little consensus over the position of Planocraniidae between our analyses, with variation principally between those using extended implied weighting with different weighting factors. Whereas all analyses using EIW12 recover Planocraniidae in the stem of Longirostres (Fig. 9), those using the more severe weighting factor (*k* = 3) position Planocraniidae in the stem of Alligatoroidea (Fig. 9). The position of Planocraniidae is also different in Analysis 1.1, in which it is polyphyletic, with *Planocrania hengdongensis* recovered as a stem crocodylian, and the remaining planocraniids forming a ‘basal’ alligatoroid clade (Fig. 9).

### Anatomical support and implications for the systematics of Gavialoidea

**Figure 17:**
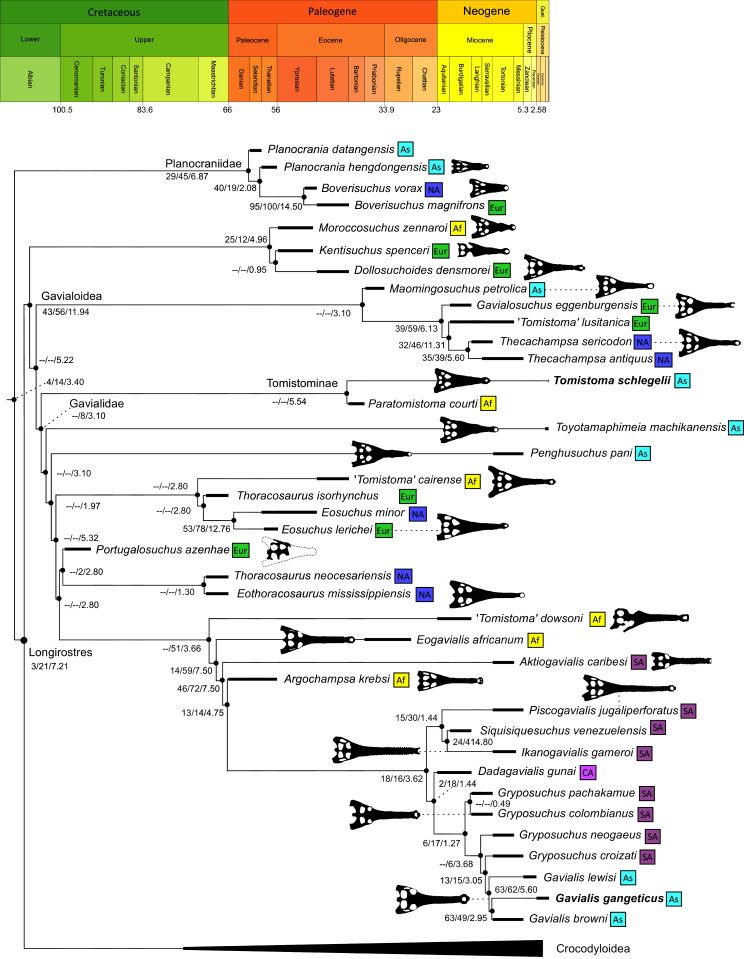
Time-calibrated phylogeny of Gavialoidea. Ages = Ma. Support values: Bootstrap/Jackknife/Bremer. Abbreviations: Af, Africa; As, Asia; CA, Central America; Eur, Europe; NA, North America.

Our modifications to the taxonomic content of Gavialoidea constitute the most substantial changes to the crocodylian tree reported in this study. Perhaps most notably, and in contrast to all previous morphology-only analyses, *Gavialis gangeticus* and *Tomistoma schlegelii* are found to be closely related, defining the crown gharial clade Gavialidae (Fig. 17). Furthermore, Gavialoidea (Bremer support = 11.9) is the sister clade of Crocodyloidea, together defining Longirostres (Fig. 17). The latter is moderately well-supported, with Bremer support = 7.2, but poorly recovered in Bootstrap and Jackknife replicates (Fig. 17).

We constrained Analysis 1.3 to recover the traditional morphological topology, *i.e*. Alligatoroidea + Crocodyloidea (=Brevirostres). Under this constraint, Gavialoidea is the sister taxon to all other crocodylians, and Crocodyloidea comprises Tomistominae and Crocodylinae (Fig. S7). Forcing this topology results in a significantly less parsimonious tree than the unconstrained analysis (72.3 extra steps, Templeton test *p* < 0.25). The results of our study are based on a new dataset that includes taxa that have not previously been incorporated, new and revised morphological characters and scores, and the use of continuous characters. To determine why the traditional morphological topology is not recovered, we provide a detailed assessment of all these factors.

#### Is the recovery of Gavialidae due to convergence in long-snouted taxa?

The gharial problem is unique in the usual narrative of morphology *versus* molecules in phylogenetics, *i.e*. groups that are well supported by morphological data are often recovered as polyphyletic in molecular analyses, which indicate that the morphology is the result of convergence. The opposite is the case for Crocodylia. Whereas morphological data indicate that the elongate snouts of *Tomistoma schlegelii* and *Gavialis gangeticus* were convergently acquired, it is explained as true homology by molecular data. The results from our analysis support the molecular hypothesis for the first time based on morphology alone; however, the recovery of Gavialidae raises the question of whether this result is due to an overwhelming number of new characters pertaining to longirostry (in this case, meaning long and narrow). Indeed, this analysis has introduced new morphological characters that explicitly factor in snout length and associated characters in phylogenetic reconstruction, *e.g*. continuous characters 1, 5, 10, 11 and 17. In addition, previous studies of neosuchian phylogeny have identified multiple morphological characters associated with longirostry (*e.g*. Jouve, 2009; Groh et al., 2020), several of which are included in this study. The role of longirostrine characters in driving our result was tested by repeating analyses 1.1 and 1.3, with the exclusion of 22 characters that have previously been identified as correlates of longirostry (Jouve, 2009; Groh et al., 2020) (Table 7). The resultant topology is almost identical to analyses implementing the full dataset (Fig. S8). Both Gavialidae and Longirostres are still recovered, but now the crown clade Gavialidae is expanded to include *Maroccosuchus zennaroi*, *Kentisuchus spenceri*, and *Dollosuchoides densmorei*. Further evidence that the result is not a product of convergence is demonstrated by character support for Gavialoidea and Gavialidae that is unrelated to longirostry (see below). Finally, several unequivocally non-gavialoid, longirostrine taxa, such as *Crocodylus johnstoni*, *Crocodylus intermedius*, *Euthecodon arambourgi*, and *Mecistops cataphractus*, do not cluster together, nor with Gavialoidea, suggesting that longirostrine characters have not ‘overwhelmed’ our analysis.

**Table 7:** Characters employed in this study identified as correlated with longirostry.

Character	State	Character description
1	N/A	Snout length in dorsal view, ratio of anteroposterior snout length to total skull length
5	N/A	Rostral depth, ratio of maximum dorsoventral height of the maxilla to mediolateral width of the maxilla at the 5th maxillary alveolus
10	N/A	Supratemporal fenestra size, ratio of anteroposterior supratemporal fenestra length to maximum anteroposterior cranial table length
11	N/A	Supratemporal fenestra shape, ratio of lateromedial width to anteroposterior length
17	N/A	Number of maxillary alveoli
30	0	Rostral ornamentation, anteroposteriorly orientated preorbital ridges extending from the anterior corner of the orbit: absent or very modest
37	0	Skull table morphology: posterolateral edges directed ventrolaterally from the sagittal axis
46	1	Nasals, external contact with naris: absent
47	2	Nasals, excluded internally from posterior narial margin
48	2	Nasals, contact with premaxillae: absent
76	70	Frontoparietal suture, shape between supratemporal fenestrae: concavo-convex
90	0	Postorbital, protuberance on the dorsolateral margin of the postorbital bar at maturity: present
107	1	Squamosal, anterior divergence of dorsal and ventral rims of lateral groove: present
146	2	Premaxilla-maxilla suture, shape on palate in ventral view: posteriorly bowed, with 1 acute apex
147	7	Maxillary alveoli homodont
158	1	Maxilla, position of alveoli relative to maxillary palate separating toothrows: or dorsal
160	1	Palatines, anterior process shape: wedge shaped
219	3	Dentary alveoli: no differentiation posterior to 4th alveolus
220	0	Dentary, shape of dorsal profile in lateral view between 4th and 10th alveoli: linear
221	3, 4	Dentary symphysis, adjacent to: 13–20 (3) or >20 (4)
224	2, 3	Splenial, anterior extent in dentary symphysis: adjacent to: 4–7 alveoli (2); >7 alveoli (3)
278	1	Axis, hypapophysis shape: forked

#### What are the reasons for the recovery of Gavialidae?

**Figure 18:**
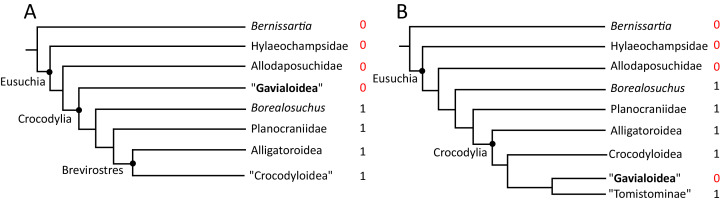
Simplified cladograms illustrating alternative optimisations of a hypothetical character on a morphological (A) and molecular (B) tree.

In essentially all previous morphological phylogenies, Gavialoidea is recovered as the earliest branching crocodylian clade, sister to all other crocodylians. The reason for this can be uncovered by optimising synapomorphies in traditional morphological datasets. Gavialoids appear to possess a suite of plesiomorphic morphological features (*i.e*. typically shared with some non-crocodylian neosuchians) that are mostly absent in Brevirostres (Fig. 18A). As such, if the molecular topology is to be supported, then these features must be explained as reversals to ‘primitive’ states (atavisms), which is less parsimonious (Gatesy et al., 2003; Harshman et al., 2003; Iijima & Kobayashi, 2019) (Fig. 18B). Modifications to the construction and coding of these atavistic characters might have played a decisive role in the recovery of Gavialidae here. Indeed, Iijima & Kobayashi (2019) recognised several gavialine atavistic character states in the East Asian taxa, *Penghusuchus pani* and *Toyotamaphimeia machikanensis*, which are usually recovered as tomistomines. Although a phylogenetic analysis implementing the new character state distributions still recovered the traditional morphological topology in that study, searches constrained to recover the molecular topology were non-significantly longer by only five steps (Iijima & Kobayashi, 2019). This demonstrates the importance of these atavistic gavialine characters, as discussed below. The second question to answer is which synapomorphies unite Gavialoidea, and other less inclusive clades that group traditional tomistomines and gavialoids together, in this study and does the novel topology reflect changes to character scores, character delimitation, and/or taxon sampling.

#### Atavistic characters

**Figure 19:**
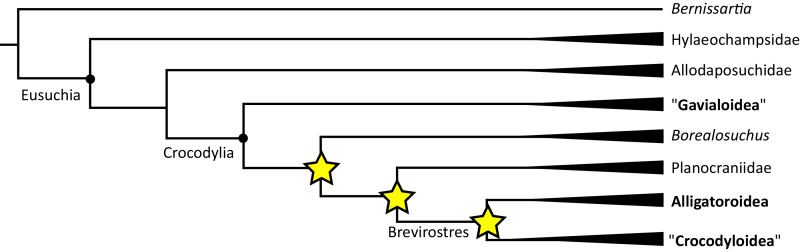
Simplified phylogeny illustrating the topology recovered by Narváez et al. (2016). Stars indicate the nodes for which synapomorphies were optimised.

Characters supporting a close relationship between Alligatoroidea and Crocodyloidea, to the exclusion of Gavialoidea (and usually most non-crocodylian taxa), were identified by optimising synapomorphies in the stem to Brevirostres in the supplementary dataset of Narváez et al. (2016) (Fig. 19). That dataset is based on the character matrix of Brochu & Storrs (2012), supplemented with a number of non-crocodylian taxa (mostly allodaposuchids) that are also present in our analysis. Narváez et al. (2016) recovered the traditional morphological topology and this is used here as a proxy for most previous morphological phylogenies. Despite differences in taxon sampling between that and our own, all major crocodylian clades are sampled in both.

Sixteen morphological characters in the dataset of Narváez et al. (2016) were identified that support the exclusion of Gavialoidea from the clade comprising (*Borealosuchus* + (Planocraniidae + Brevirostres)): 9-0, 12-0, 14-1, 18-1, 27-0, 28-0, 33-0, 34-0, 38-0, 40-0, 43-0, 108-0, 134-0, 135-0, 149-1, 181-0 (character numbers and states from Narváez et al. (2016)). All of these characters are used in the present study, albeit with varying degrees of modification. The optimisation pattern of these characters was compared between this study and the dataset of Narváez et al. (2016). Modifications to most of these characters appears to be formative in the recovery of Gavialidae herein. The characters, score changes, and changes to character delimitations discussed below and summarised in Table 8.

**Table 8:** Changes to characters that supported the traditional morphological topology.

Narváez et al. (2016)	This study	Character states supporting the traditional morphological topology	Modification to character	Difference in optimisation pattern
135-0	92-0	Postorbital bar, flush with dorsolateral margin of the jugal	Unmodified	Gavialoid characters reversed
181-0	118, 119	118: Quadrate condyle, notch on the dorsal articular border: absent or small. 119: quadrate condyle shape, dorsal and ventral margins subparallel across length	Reductively coded, new states added	Gavialoid characters reversed
12-0	274-0	Axis neural spine, posterior half of spine dorsally inflected to form crest	Unmodified	Change in plesiomorphic condition for Eusuchia and Crocodylia
28-0	304-0	Humerus, scarring on proximodorsal surface: two muscle scars discernible (for *M. teres* major and *M. dorsalis* scapulae)	Unmodified	Change in plesiomorphic condition for Eusuchia and Crocodylia
38-0	325-0	Dorsal osteoderms, longitudinal midline keel: absent	Unmodified	Change in plesiomorphic condition for Eusuchia and Crocodylia
108-0	66-0	Prefrontal pillar morphology: dorsal half of pillar narrow, less than twice minimum anteroposterior length	Unmodified	Change in plesiomorphic condition for Eusuchia and Crocodylia
149-1	115-0	External auditory meatus, posterior margin shape: straight	Reductively coded	Plesiomorphic condition ambiguous
14-1	276-1	Axis, lateral process (diapophysis) on neural arch lateral margin: present	Unmodified	Gavialoid condition found in traditional tomistomines
27-0	302-0	Humerus, proximal edge of deltopectoral crest: straight, emerging smoothly from proximal end of the humerus	Unmodified	Gavialoid condition found in traditional tomistomines
33-0	307-0	Ilium, preacetabular process shape: acute, pointed anteriorly	Unmodified	Gavialoid condition found in traditional tomistomines
34-0	309-0	Ilium, dorsal outline of the postacetabular process: convex, no dorsal indentation	Reductively coded, ordered	Gavialoid condition found in traditional tomistomines
43-0	327-0	Dorsal midline osteoderms, anterolateral process: present	Unmodified	Gavialoid condition found in traditional tomistomines
9-0	271-0	Axial rib, tuberculum shape: broad, equal in size to capitulum	Unmodified	Gavialoid condition found in crocodyloids
18-1	279-1	First postaxial vertebra, prominent hypapophysis: absent	Unmodified	Atavism
40-0	324-1	Dorsal osteoderms, number in each transverse row at maturity: four	New state added, ordered	Atavism
134-0	90-0	Postorbital, protuberance on the dorsolateral margin of the postorbital bar at maturity: present	Unmodified	Atavism

##### Postorbital bar flush with the dorsal margin of the jugal

In Narváez et al. (2016 (C135)), all gavialoids were scored for the plesiomorphic condition, in which the postorbital is flush against the jugal (C135-0). By contrast, *Borealosuchus*, Planocraniidae, and Brevirostres were scored with the alternative condition, in which the postorbital is separated from the jugal arch by a sulcus (C135-1). Consequently, this character supported the sister relationship of Gavialoidea to all other crocodylians. In the current study (C92), all gavialoids, except *Gavialis*, are shown to share the same condition as all other crocodylians (C92-1), thus weakening the case to separate Gavialoidea from *Borealosuchus*, Planocraniidae, and Brevirostres. The flush condition in *Gavialis* is now recovered as a local autapomorphy in Gavialidae (C92-0).

##### Medial hemicondyle of the quadrate, small and ventrally reflected

In the dataset of Narváez et al. (2016 (C181)), essentially all gavialoids were scored as possessing a ventrally reflected medial hemicondyle (C181-0), which was shared with all non-crocodylian taxa and *Borealosuchus*. By contrast, Planocraniidae, Crocodyloidea, and Alligatoroidea each exhibited a different condition. This character has received significant modification here, including separation into two characters, which describe differences in the morphology of a notch on the quadrate condyle (C118) and the overall shape of the quadrate condyle (C119) (Appendix 2: fig. 45). The result is that the presence of a small notch on the dorsomedial corner of the quadrate condyle (C118-0) is recovered as a synapomorphy of Longirostres. The second character (C119) has a more complicated pattern when optimised. Whereas some gavialoids are still shown to share a ventrally reflected medial hemicondyle in common with non-crocodylian taxa (C119-3), other gavialoids exhibit a rectangular quadrate condyle (C119-0), which is different to non-crocodylian taxa, but also unlike most members of Longirostres.

##### Axis neural spine crested

According to the dataset of Narváez et al. (2016 (C12)), a posteriorly crested axial neural spine is the plesiomorphic condition for Eusuchia and Crocodylia (C12-0). The presence of a crested neural spine in Gavialoidea (C12-1) appears to draw this clade stemwards. Furthermore, according to their dataset, the crested neural spine is lost at the node comprising ((*Borealosuchus* + (Planocraniidae + Brevirostres)), but then regained in Crocodyloidea. There have been some important changes to character scores and taxon sampling here (C274 in our study). For example, the small preserved portion of the axis of *Shamosuchus* is considered uncrested (Pol, Turner & Norell, 2009, fig. 21D) (previously ‘?’). Additionally, *Isisfordia* has been included here, which also has an uncrested axial neural spine (Salisbury et al., 2006, fig. 3A). The result of these changes is that an uncrested axial neural spine is the plesiomorphic condition at the nodes of both Eusuchia and Crocodylia (C274-1). This polarity change has been facilitated by the large amount of missing data in non-crocodylian taxa (*e.g*. all hylaeochampsids and allodaposuchids). The presence of a crested axial neural spine is now recovered as a potential synapomorphy of Gavialidae (C274-0).

##### Two scars for M. teres major and M. dorsalis scapulae dorsal to deltopectoral crest

In the dataset of Narváez et al. (2016 (C28)), where preserved, all non-crocodylian taxa and Gavialoidea are scored as having two muscle insertion scars on the humerus. In their study, this condition is optimised as the plesiomorphic condition in Eusuchia and Crocodylia, lost at the node comprising (*Borealosuchus* + (Planocraniidae + Brevirostres)). In our study (C304), *Bernissartia* is the only non-crocodylian taxon to exhibit the double muscle insertion (C304-0). The plesiomorphic condition for Eusuchia and Crocodylia has thus changed to a single muscle insertion (C304-1), and the double muscle insertion of several gavialoids is now an atavism. The change in character optimisation appears to be a result of minor differences in taxon sampling, a few character-score changes in non-crocodylian taxa, coupled with missing data. For example, whereas Narváez et al. (2016) included the hylaeochampsid *Pietraroiasuchus ormezzanoi* (which appears to have the condition of a double insertion scar), it was not incorporated here. Additionally, *Isisfordia* is included in this study (which lacks the condition of a double insertion scar), but it was not included in the dataset of Narváez et al. (2016). Furthermore, the condition in *Shamosuchus* has been changed from missing data to a ‘1’ (Pol, Turner & Norell, 2009, fig. 25). Coupled with the lack of data in all other non-crocodylian taxa, these minor differences facilitate the change in character optimisation.

##### Absence of longitudinal crest on dorsal midline osteoderms

In the dataset of Narváez et al. (2016 (C38)), the absence of a midline keel on dorsal osteoderms is recovered as the plesiomorphic condition in Eusuchia (38-0). The shared absence of keeled osteoderms in Gavialoidea supports its placement as an early branching crocodylian clade, along with *Borealosuchus*. Unkeeled osteoderms are also present in some taxa usually included in Tomistominae, but this is explained by convergence under the traditional topology. In the dataset presented here (C325), the plesiomorphic condition in Eusuchia has changed to the possession of keeled osteoderms (C325-1)). This change in polarity is due in part to the recognition of keeled osteoderms in *Theriosuchus pusillus*, the inclusion of *Isisfordia* (which has keeled osteoderms), and the high proportion of missing data in non-crocodylian taxa such as Allodaposuchidae and Hylaeochampsidae, which are now optimised as having keeled osteoderms. Furthermore, the presence of unkeeled osteoderms is recovered as a synapomorphy of the least inclusive clade comprising *Toyotamaphimeia* and *Gavialis gangeticus*.

##### Dorsal half of the prefrontal pillar narrow

Based on the dataset of Narváez et al. (2016 (C108)), the dorsal half of the prefrontal pillar is ‘primitively’ narrow in Eusuchia, which is shared by gavialoids (C108-0), supporting their early divergence in Crocodylia. By contrast, the node comprising (*Borealosuchus* + (Planocraniidae + Brevirostres)) is characterised by having a dorsally broad prefrontal pillar. As with the preceding characters, the optimisation of the narrow pillar morphology as being plesiomorphic is based on very few taxa that preserve this portion of the anatomy. In our study (C66), changes to taxon sampling, coupled with minor changes to character scores and missing data, have resulted in a reversal of the plesiomorphic eusuchian condition to dorsally broad prefrontal pillars (C66-1). Furthermore, several gavialoids are found to have a wide pillar (*e.g. Eogavialis*, *Piscogavialis*, *Eosuchus lerichei*), in common with all other crocodylians, whereas others do not preserve this portion of the anatomy (*e.g. Thoracosaurus*, *Tomistoma lusitanica*).

##### Posterior margin of the otic aperture linear

According to the dataset of Narváez et al. (2016 (C149)), the posterior margin of the otic aperture is plesiomorphically linear in Eusuchia (C149-1). This condition is shared by Gavialoidea, *Borealosuchus*, and Planocraniidae, but absent in Brevirostres, which exhibit an invaginated posterior margin of the otic aperture. In the present study (C112), it is agreed that most alligatoroids, crocodyloids, and traditional tomistomines exhibit the invaginated condition, and that traditional gavialoids exhibit the linear condition. However, by contrast to Narváez et al. (2016), the condition in all non-crocodylian eusuchians, except *Borealosuchus* and *Shamosuchus*, is considered unknown. Although the condition in allodaposuchids and hylaeochampsids is scored as a ‘?’, this actually represents inapplicable data. These taxa exhibit an entirely different morphology, in which the squamosal and quadrate do not contact each other posterior to the otic aperture (C112-1). This ambiguity in the stem to Crocodylia reduces the stemward pull on Gavialoidea, and the linear otic aperture is recovered as a synapomorphy of the least inclusive clade containing *Eothoracosaurus mississippiensis* and *Gavialis gangeticus*.

##### Presence of a diapophysis on the axial neural arch

The pattern of character optimisation for the presence of an axial diapophysis is essentially the same in this study as that in Narváez et al. (2016 (C14)). Among non-crocodylian taxa preserving the axis, a diapophysis is only scored as present in *Bernissartia* in our dataset (C276-1). As in Narváez et al. (2016), the axial diapophysis is lost in subsequent nodes but independently evolved in some gavialoids. An important difference to their study, and most previous morphological analyses, is the recognition of an axial diapophysis in *Toyotamaphimeia* and *Penghusuchus* (Iijima & Kobayashi, 2019). Incorporating this information means that this condition is now recovered as a synapomorphy of the least inclusive clade comprising *Toyotamaphimeia machikanensis* and *Gavialis gangeticus*.

##### Proximal edge of the deltopectoral crest emerges smoothly from proximal end of humerus

There are no changes to character scores of non-crocodylian taxa from the dataset of Narváez et al. (2016 (C27)), and only minor changes to scores in some gavialoids, for this feature. As in previous analyses, the presence of a low, smoothly emerging deltopectoral crest is optimised as the plesiomorphic condition in Eusuchia in our dataset (C302-0). Where preserved, this condition is present in *Bernissartia*, *Theriosuchus*, and all *Borealosuchus* species. However, unlike the dataset of Narváez et al. (2016), *Penghusuchus* is included here, which has recently been shown to exhibit the plesiomorphic condition (Iijima & Kobayashi, 2019). The presence of a low deltopectoral crest is now recovered as an atavistic synapomorphy of the least inclusive clade comprising *Penghusuchus pani* and *Gavialis gangeticus*.

##### Prominent preacetabular process on the ilium

As in previous analyses, the presence of a prominent preacetabular process of the ilium is recovered as the plesiomorphic condition in Eusuchia and Crocodylia, present in *Bernissartia*, *Theriosuchus*, and *Allodaposuchus precedens* (C307-0), but unknown in most other non-crocodylian taxa. In the dataset of Narváez et al. (2016 (C33)), the presence of a prominent preacetabular process in Gavialoidea (C33-0) was optimised as providing support for the early divergence of this group. Although the condition is still recovered here in most gavialoids, it is more parsimoniously optimised as a re-acquisition of the primitive condition. Score changes to traditional gavialoid taxa have probably made little contribution to this result, *e.g. Thoracosaurus isorhynchus* and *Thoracosaurus neocesariensis* are both scored with a ‘?’ for this character, but these taxa are still optimised as possessing a prominent preacetabular process. A prominent preacetabular process has also been recognised in *Toyotamaphimeia* and *Penghusuchus* (Iijima & Kobayashi, 2019). As a result, this feature is optimised as a synapomorphy of the least inclusive clade comprising *Toyotamaphimeia machikanensis* and *Gavialis gangeticus*.

##### Dorsal margin of the iliac blade rounded with a smooth border

In previous studies, the plesiomorphic condition for Eusuchia is for the dorsal outline of the postacetabular process of the ilium to lack an indentation. This condition was scored for Gavialoidea and some ‘basal’ alligatorines, but not in crocodylines, traditional tomistomines, or most other alligatoroids in Narváez et al. (2016 (C34)). This equivocally supports the early divergence of Gavialoidea from all other crocodylians in traditional morphological datasets. In the present study, the plesiomorphic condition remains unchanged; however, we have made multiple character score changes. Furthermore, the inclusion of *Toyotamaphimeia* and *Penghusuchus* in this dataset, which, unlike other traditional tomistomines, retain the plesiomorphic condition, allies them with gavialoids. The high proportion of missing data in traditional tomistomines, which are usually optimised as sharing the same condition as crocodylines, facilitates a change in the optimised character state to the plesiomorphic condition. This character was listed as a potential atavism by Iijima & Kobayashi (2019), but it is not recovered as such here because the plesiomorphic condition is retained at the base of Longirostres.

##### Anterolateral process on dorsal midline osteoderms

As in previous analyses (*e.g*. Narváez et al., 2016 (C43)), the presence of an anterolateral process on the dorsal midline osteoderms is plesiomorphic for Eusuchia and remains so in the immediate stem to Crocodylia (C327-0). No significant changes are made to scores of non-crocodylian eusuchians, except the presence of the plesiomorphic condition in *Shamosuchus* (previously missing data), but this further supports the ‘primitive’ nature of this feature. In addition to *Shamosuchus*, the plesiomorphic condition is present in *Bernissartia*, *Theriosuchus* and *Borealosuchus* (although *Isisfordia* shows the derived condition (C327-1)). Where preserved, the plesiomorphic condition is also present in most traditional gavialoids, *e.g. Gavialis gangeticus*, *Eogavialis africanum*, *Eosuchus minor* and *Eothoracosaurus*. However, by contrast to traditional topologies, the anterolateral process is optimised as being an atavism in gavialoids, rather than retention of the plesiomorphic condition. Within Gavialoidea, notable changes to character scores include the recognition of the plesiomorphic condition in *Penghusuchus* and *Toyotamaphimeia* (Iijima & Kobayashi, 2019). Indeed, the presence of an anterolateral process on the dorsal midline osteoderms is recovered as a synapomorphy of the least inclusive clade comprising *Toyotamaphimeia* and *Gavialis gangeticus*. The plesiomorphic condition is also newly recognised in several other crocodylian taxa, such as some *Diplocynodon* species, but these changes appear to be inconsequential to the placement of Gavialoidea recovered here.

##### Broad axial rib tuberculum equal in size to tuberculum

In previous studies (*e.g*. Narváez et al., 2016 (C9)), the presence of a broad axial rib tuberculum was recovered as the plesiomorphic condition in Eusuchia and Crocodylia. Where preserved, the same condition is recovered in most gavialoids, as well as *Borealosuchus*. By contrast, a narrow axial rib tuberculum was recovered as a synapomorphy of Brevirostres. That gavialoids possess a broad axial rib tuberculum (C271-0) is agreed upon here; however, major character score changes have been made to Crocodyloidea, *Tomistoma schlegelii*, and some taxa traditionally included within Tomistominae (*e.g. Maomingosuchus*), such that they are also recognised as having a broad axial rib tuberculum. After these modifications, a broad axial rib tuberculum is optimised as a retained plesiomorphic feature in Longirostres that is only lost in Alligatoridae, members of which exhibit a narrow axial rib tuberculum (C271-1).

##### First postaxial vertebra lacking a prominent hypapophysis

As in previous analyses (*e.g*. Narváez et al., 2016 (C18)), the absence of a hypapophysis on the third cervical vertebra is recovered as the plesiomorphic condition in Eusuchia and remains so in the immediate stem to Crocodylia in our dataset (C279-1). The absence of a hypapophysis in Gavialoidea was optimised as the retention of the plesiomorphic condition in previous studies, whereas here it is identified as convergently acquired instead. A number of character score changes have been made to traditional gavialoid taxa, but these do not appear to have significantly altered the topology. For example, both species of *Thoracosaurus* are now scored as ‘?’, but these are still optimised as possessing the feature. Furthermore, *Eosuchus lerichei* is found to share the ‘gavialoid condition’, strengthening its position within Gavialoidea. *Toyotamaphimeia* is scored for the derived condition, but this is recovered as an autapomorphy of the species.

##### Four contiguous dorsal osteoderms per row at maturity

As in Narváez et al. (2016 (C40)), Gavialoidea is here found to have the same number of dorsal osteoderms (four) as occurs plesiomorphically in Eusuchia (C324-1), and unlike all other crocodylians which have between six (C324-2) to eight (C324-3) dorsal osteoderms. Although all non-crocodylian taxa are optimised as having the same condition as Gavialoidea in our analysis, the plesiomorphic condition is somewhat ambiguous. Whereas *Bernissartia* has four dorsal osteoderms, *Theriosuchus* only has two, for which a new character state (C324-0) has been added. *Isisfordia* differs again in having six dorsal osteoderms. Determining the plesiomorphic condition for Crocodylia is further complicated by the lack of data for all allodaposuchid and hylaeochampsid species, which are optimised as having four dorsal osteoderms. Furthermore, the condition in most gavialoids is unknown: although 22 taxa are optimised as having four osteoderms, in this study the condition is definitively known only in *Gavialis gangeticus* and *Eosuchus minor*. The condition is also unknown in taxa traditionally included within Tomistominae, with the exception of the extant species, *Tomistoma schlegelii*, which has six dorsal osteoderms.

##### Process on the dorsolateral surface of the postorbital bar

A spine on the dorsolateral surface of the postorbital bar is present in *Bernissartia* and several non-crocodylian eusuchians (C90-0), and it has long been recognised as a ‘primitive’ feature in Eusuchia (*e.g*. Norell, 1989). In most morphological phylogenies (*e.g*. Narváez et al., 2016 (C134)), the shared presence of this spine in Gavialoidea, and its absence in essentially all brevirostrines, supports the position of Gavialoidea as an early diverging crocodylian clade. Here, the presence of a postorbital spine is still recovered as the plesiomorphic condition in Eusuchia and Crocodylia, but the condition in gavialoids is homoplastic. The plesiomorphic condition is newly recognised in *Kentisuchus spenceri* and *Maroccosuchus zennaroi*; however, this is recovered as another instance of convergence.

**Figure 20:**
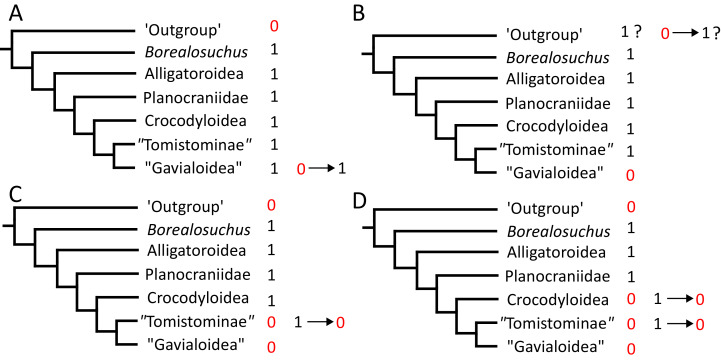
Simplified cladograms illustrating the principal changes in character optimisation of synapomorphies of Brevirostres and stemwards nodes recovered in Narváez et al. (2016). For simplicity, ’outgroup’ refers to all taxa stemward of *Borealosuchus* recovered here. See text for further explanation.

In summary, two morphological characters received significant character score changes in gavialoid taxa, such that they now share the condition present in alligatoroids and crocodyloids (Fig. 20A). Five characters received modifications to scores, particularly in non-crocodylian taxa, such that they no longer share the same condition as gavialoids. This results either in a reversal in character polarity at the nodes of Eusuchia and Crocodylia, or the condition becomes ambiguous at these nodes (Fig. 20B). In six characters, the plesiomorphic condition in Eusuchia and Crocodylia is unchanged, as are scores within Gavialoidea; however, the ‘gavialoid condition’ is now also recognised in traditional tomistomines (Fig. 20C) or in all crocodyloids (Fig. 20D), supporting an expanded Gavialoidea and Longirostres, respectively. A further three characters received essentially no character score changes in gavialoids and non-crocodylians, but they are now more parsimoniously optimised as atavisms within Gavialoidea (Fig. 18B).

**Figure 21:**
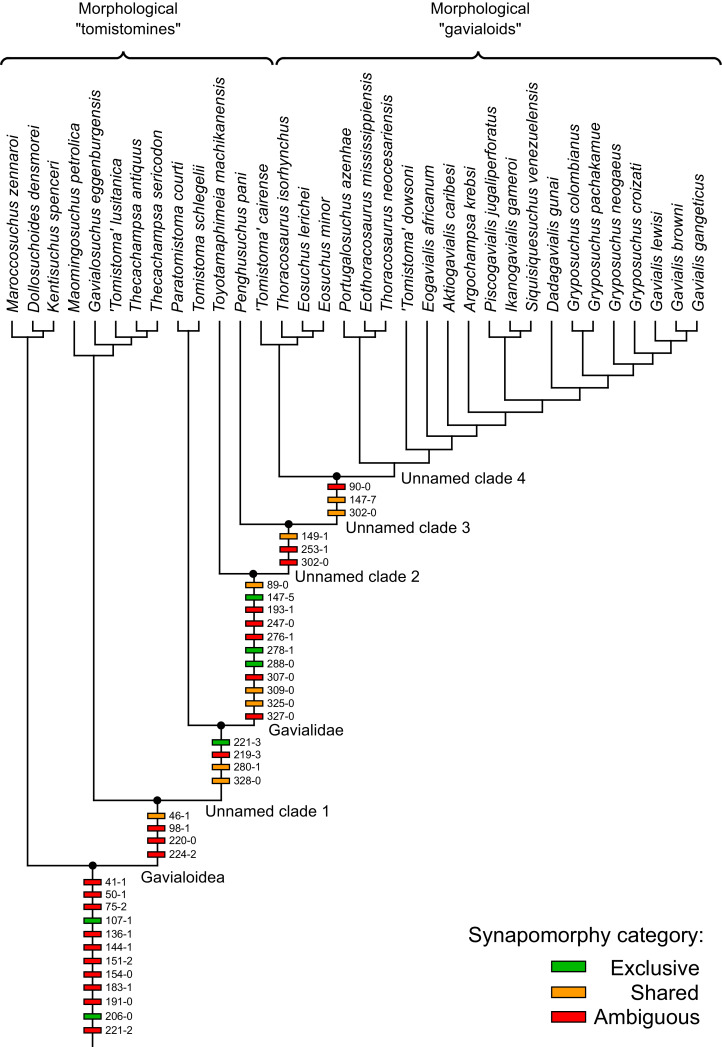
Synapomorphies of Gavialoidea and less inclusive clades that unite morphological (=traditional) tomistomines and gavialoids.

In addition to modifications to some characters that previously pulled gavialoids towards the stem of Crocodylia, there are also a number of additional characters supporting clades containing traditional tomistomines and gavialoids (Fig. 21). Synapomorphies of all nodes containing these taxa are discussed below and summarised in Table 9.

**Table 9:** Summary of synapomorphies of Gavialoidea and less inclusive clades uniting morphological gavialoids and tomistomines.

Character and state	Node/Least inclusive clade	Synapomorphy type	Previously found in gavialoids + tomistomines	Character modifications	Score changes
41-1	Gavialoidea	Ambiguous	Yes	None	Minor
50-1	Gavialoidea	Ambiguous	Yes	None	Minor
75-2	Gavialoidea	Ambiguous	No	Ordered	Minor
107-1	Gavialoidea	Exclusive	Yes	None	Major
136-1	Gavialoidea	Ambiguous	No	State removed	Major
144-1	Gavialoidea	Ambiguous	Yes	None	Minor
151-2	Gavialoidea	Ambiguous	Yes	Ordered	Minor
154-0	Gavialoidea	Ambiguous	New character	N/A	N/A
183-1	Gavialoidea	Ambiguous	No	None	Minor
191-0	Gavialoidea	Ambiguous	New character	N/A	N/A
206-0	Gavialoidea	Exclusive	Yes	None	Minor
221-2	Gavialoidea	Ambiguous	Yes	State added, ordered	N/A
46-1	*Maomingosuchus* + *Gavialis*	Shared	Yes	Reductively coded	N/A
98-1	*Maomingosuchus* + *Gavialis*	Ambiguous	No	None	Major
220-0	*Maomingosuchus* + *Gavialis*	Ambiguous	Yes	None	Minor
224-2	*Maomingosuchus* + *Gavialis*	Ambiguous	No	Reductively coded	N/A
219-3	Gavialidae	Ambiguous	No	None	Major
221-3	Gavialidae	Exclusive	Yes	State added, ordered	N/A
280-1	Gavialidae	Shared	Yes	None	Minor
328-0	Gavialidae	Shared	Yes	None	Minor
89-0	*Toyotamaphimeia* + *Gavialis*	Shared	Yes	None	Major
193-1	*Toyotamaphimeia* + *Gavialis*	Ambiguous	No	Reductively coded	Minor
247-0	*Toyotamaphimeia* + *Gavialis*	Ambiguous	Yes	None	Minor
278-1	*Toyotamaphimeia* + *Gavialis*	Exclusive	Yes	None	Minor
288-0	*Toyotamaphimeia* + *Gavialis*	Exclusive	Yes	None	Minor
149-1	*Penghusuchus* + *Gavialis*	Shared	New character	N/A	N/A
253-1	*Penghusuchus* + *Gavialis*	Shared	Yes	None	Major
147-7	‘*Tomistoma*’ *cairense* + *Gavialis*	Shared	Yes	None	N/A

#### Character support for Gavialoidea

Gavialoidea is supported by 16 synapomorphies: (1) ratio of rostrum length to skull length = 0.67 (C1); (2) ratio of rostral depth to rostrum width = 0.43 (C5); (3) ratio of supratemporal fenestra width to length = 0.98 (C11); (4) inclination of dorsal margin of external mandibular fenestra = 22° (C19); (5) dorsally projecting naris (C41-1, ambiguous); (6) posterodorsal processes of the premaxillae extending beyond the level of the 3rd maxillary alveolus (C50-1, ambiguous); (7) frontoparietal suture does not intersect the supratemporal fenestrae (C75-2, ambiguous); (8) lateral grooves of the squamosal flare anteriorly (C107-1, exclusive); (9) rounded posterior pterygoid processes (C136-1, ambiguous); (10) last premaxillary alveolus positioned medially to the penultimate alveolus (C144-1, ambiguous); (11) inline occlusion of the dentary and maxillary teeth (C151-2, ambiguous); (12) absence of a diastema between maxillary alveoli 6 and 7 (C154-0, ambiguous); (13) absence of anterior process of the quadratojugal on medial jugal surface (C183-1, ambiguous); (14) anterior margin of the choanae not invaginated (C191-0, ambiguous); (15) capitate process of the laterosphenoid orientated laterally (C206-0, exclusive); and (16) dentary symphysis adjacent to the level of 9–12 alveoli (C221-2, ambiguous).

Most traditional tomistomines and gavialoids have previously been scored as sharing an anteriorly flaring lateral squamosal groove (C107-1), which has been interpreted as homoplasy (Brochu, 1997b; Harshman et al., 2003; Brochu et al., 2012; Jouve, 2016; Narváez et al., 2016). Potentially significant character score changes have been made here, resulting in the more widespread occurrence of the derived condition in taxa usually referred to Tomistominae. This derived condition is newly recognised in *Kentisuchus spenceri* (formerly absent in all other studies) and *Maroccosuchus zennaroi* (absent according to Jouve, 2016). This feature is recognised as an exclusive synapomorphy of Gavialoidea here, given that it is absent in *Tomistoma schlegelii*, *Maomingosuchus petrolica*, ‘*Tomistoma*’ *cairense*, *Eosuchus lerichei*, and *Portugalosuchus azenhae*.

Traditionally, a laterally directed capitate process of the laterosphenoid (C206-0) has been recovered in all gavialoids, where preserved. This condition has previously been considered present in some, but not all tomistomines, and has been interpreted as another example of convergence. For example, based on existing datasets and corroborated here, *Maroccosuchus*, *Paratomistoma*, and *Thecachampsa* each exhibit a laterally directed process. By contrast, *Tomistoma schlegelii* and *Penghusuchus* exhibit an anterolaterally directed capitate process (C206-1), as in all other crocodylians. Character scores for several other traditional tomistomine taxa have been changed from the derived condition (as scored by Jouve, 2016) to missing data (*Kentisuchus* and *Gavialosuchus eggenburgensis*) (see also Narváez et al., 2016). Additionally, the plesiomorphic condition is recognised in ‘*Tomistoma*’ *dowsoni*, which has rarely been included in phylogenetic analyses.

A dorsally facing external naris (C41-1) is recovered as a synapomorphy of Gavialoidea here; however, this condition evolved independently multiple times within Crocodylia, including in Caimaninae, some alligatorines, crocodylines, and mekosuchines. As in previous analyses, the plesiomorphic condition in Crocodylia, and indeed in Eusuchia, is for the naris to face anterodorsally. Whereas in the traditional topology the dorsally facing condition was considered to have evolved independently in Gavialoidea and Crocodyloidea, here it is recovered as a shared derived feature of traditional tomistomines and gavialoids that is convergent in crocodylines. There have been no significant changes to character scores of gavialoid taxa that have influenced this result.

Posterodorsal processes of the premaxillae which extend posteriorly beyond the level of the 3^rd^ maxillary alveolus (C50-1) have been recognised in traditional tomistomines and gavialoids in previous studies (Brochu et al., 2012; Jouve, 2016; Narváez et al., 2016; Salas-Gismondi et al., 2019). Whereas this was formerly explained as convergence, it is recovered as a shared derived feature here. There have been no significant changes to gavialoid character scores, except the recognition of the derived condition in *Maomingosuchus petrolica* (formerly unknown).

A frontoparietal suture that sits entirely on the skull table (C75-2) is recovered as a shared synapomorphy of Gavialoidea. This condition evolved independently within Alligatoroidea and Crocodyloidea, and it is also ambiguous because the condition varies within Gavialoidea. Whereas traditional tomistomine taxa forming the stem of Gavialoidea exhibit a frontoparietal suture entirely exposed on the skull table (C75-2), the suture incipiently contacts the supratemporal fenestrae in crownward taxa (C75-1), which mostly correspond to traditional gavialoids. Furthermore, several taxa (mostly ‘thoracosaurs’) exhibit a deep intersection of the frontopareital suture with the supratemporal fenestrae (C75-0). Several score changes have been made to gavialoid taxa and the character is newly ordered, but these modifications do not appear to be the reason for uniting traditional tomistomines and gavialoids.

The last premaxillary alveolus is medially inset relative to the penultimate alveolus (C144-1) in all traditional tomistomines and most gavialoids, where preserved. This character was first introduced in the analysis of Jouve et al. (2015), in which this feature was recovered as convergently acquired by Tomistominae and Gavialoidea. By contrast, it is recovered as a shared synapomorphy of Gavialoidea here, also occurring in *Mecistops cataphractus*. This character is slightly ambiguous within Gavialoidea, as the last alveolus is posterior or posterolateral to the penultimate alveolus in *Thecachampsa antiquus*, *Eosuchus*, and *Eothoracosaurus*.

A dentary symphysis adjacent to 9–12 alveoli (C221-2) is a shared synapomorphy of Gavialoidea, which evolved convergently in *Borealosuchus formidabilis*, some paralligatorids, and a small number of alligatoroids. This synapomorphy is not shared by all gavialoids, as the dentary symphysis elongates stepwise (>20 alveoli, (C221-4)) in crownward nodes, especially in the clade comprising (*Piscogavialis* + (*Siquisiquesuchus* + *Ikanogavialis*)), as well as independently in *Gavialis*. Nevertheless, no non-gavialoid taxon exhibits a dentary symphysis exceeding 12 alveoli. Comparisons of character score changes with previous studies are challenging, as the delimitation of this character differs between datasets (*e.g*. Brochu et al., 2012; Narváez et al., 2016; Salas-Gismondi et al., 2016). Most datasets delimited this character with only three character states (Brochu et al., 2012; Jouve, 2016; Lee & Yates, 2018; Iijima & Kobayashi, 2019), in which traditional tomistomines and gavialoids have the same score, to the exclusion of all other crocodylians; however, this has always previously been interpreted as homoplasy. Salas-Gismondi et al. (2016, 2019) added a fourth character state to capture the even longer dentary symphysis that distinguishes traditional gavialoids from tomistomines. Whereas the modification to the character by Salas-Gismondi et al. (2016) is accepted here, where it is further augmented by an additional character state describing a symphysis adjacent to >20 alveoli, the character is ordered in this study, contrasting with previous analyses. Consequently, although traditional tomistomines and gavialoids are still scored as having broadly different symphysis lengths, they are recognised as being more similar to each other than to most other crocodylians.

In previous studies, the presence of interlocking maxillary and dentary teeth (C151-2) was recovered as a shared derived feature of tomistomines and crocodylines, which independently evolved in Gavialoidea. Here, there are few significant changes to character scores in any of these clades, but the interlocking dentition of traditional gavialoids and tomistomines is now a shared derived feature that convergently evolved within Crocodyloidea. Although the interlocking condition is present in many crocodyloids, it is not recovered as a synapomorphy of Longirostres, as some ‘basal’ crocodyloids (*e.g. Asiatosuchus germanicus*) exhibit an overbite (C151-0), and many have an intermediate interlocking condition (C151-1), including several mekosuchines, ‘*Crocodylus*’ *affinis* and ‘*Asiatosuchus*’ *depressifrons*.

Here, as in previous analyses (Brochu et al., 2012; Narváez et al., 2016; Jouve, 2016), the presence of a long anterior quadratojugal process on the medial surface of the lower temporal bar is recovered as the plesiomorphic condition in Eusuchia and in Crocodylia (C183-0). Whereas the absence of an anterior process (C183-1) has previously been recovered as an ambiguous synapomorphy of Crocodylidae (Narváez et al., 2016), it is here recovered as an ambiguous synapomorphy of Gavialoidea, which convergently evolved in crocodylines, osteolaemines, and some ‘basal’ crocodyloids. Since traditional gavialoids are scored with the plesiomorphic condition (C183-0), this indicates that a reversal occurred within crownward nodes of Gavialoidea.

The presence of a linear or curved anterior choanal margin (C191-0) provides new support for Gavialoidea. This condition is plesiomorphic in Eusuchia, with the ‘derived’ invaginated condition (C191-1) evolving in Allodaposuchidae, *Borealosuchus*, and most alligatoroids. The condition in Gavialoidea is recovered as an atavism, which convergently evolved within Crocodylidae (present in all crocodylines, some osteolaemines, and mekosuchines).

The absence of a diastema between maxillary alveoli 6 and 7 also provides new, but ambiguous, support for Gavialoidea (C154-0). This is a shared synapomorphy that is highly labile in Crocodylia. The diastema is plesiomorphically absent in Eusuchia, but it is gained at the node uniting *Borealosuchus* + Crocodylia. Some alligatoroids secondarily lost the diastema, as also occurred in Gavialoidea. The diastema is ‘primitively’ present in Crocodyloidea, but independently lost again in several taxa.

The presence of short posterior pterygoid processes that are not dorsoventrally expanded is a shared synapomorphy of Gavialoidea. Formerly, this character had three states, which described: tall posterior processes (0), optimised as the plesiomorphic condition in Eusuchia and Crocodylia; small processes that are directed posteroventrally (1), optimised as an ambiguous synapomorphy of Crocodylidae; and small processes that are directed posteriorly (2), optimised as a synapomorphy of derived gavialoids. Despite the presence of small posterior pterygoid processes in some gavialoids and in most traditional tomistomines, these taxa must be scored for different character states. In the revised format, the direction of the pterygoid processes is disregarded, and character 135 distinguishes between dorsoventrally tall (C135-0) or short (C135-1) processes. The presence of short posterior processes in traditional gavialoids and tomistomines is recovered as a shared derived feature, convergently evolved in crocodylines. Small posterior processes are not present in all gavialoids. As recognised in previous studies, ‘thoracosaurs’, such as *Thoracosaurus*, *Eosuchus*, and *Eothoracosaurus*, possess dorsoventrally expanded posterior pterygoid processes.

#### Character support for unnamed gavialoid clade 1

Unnamed gavialoid clade 1 comprises *Maomingosuchus petrolica* + *Gavialosuchus eggenburgensis* + ‘*Tomistoma*’ *lusitanica* + *Thecachampsa sericodon* + *Thecachampsa antiquus* + Gavialidae. It is diagnosed by six synapomorphies: (1) ratio of rostrum length to skull length = 0.67–0.68 (C1); (2) ratio of rostral depth to rostral width = 0.46–0.58 (C5); (3) absence of external contact between the nasals and naris (C46-1, shared); (4) dorsal margin of the infratemporal fenestra oval shaped (C98-1, ambiguous); (5) dorsal profile of the dentary linear (C220-0, ambiguous); and (6) splenial symphysis adjacent to 4–7 alveoli (C224-2, ambiguous).

This clade is supported by a splenial symphysis whose anterior extent is adjacent to 4–7 teeth (C224-2). This synapomorphy is not shared by any other crocodylian taxon, but it occurs independently in *Theriosuchus pusillus* and some paralligatorids (*Wannchampsus kirpachi* and the Glen Rose Form). As with the dentary symphysis, the splenial symphysis in Gavialoidea lengthens even further in crownward nodes to more than seven alveoli (C224-3). The reductive coding of this character has clearly factored into the recovery of an expanded Gavialoidea. As delimited in most studies, this character describes separate conditions for traditional tomistomines and gavialoids. Although both share a long splenial symphysis, in traditional tomistomines it is described as ‘constricted’, but in gavialoids it is described as ‘wide’. These character states are optimised as unambiguous synapomorphies of the traditional Tomistominae and Gavialoidea clades, respectively, in all previous morphological phylogenies. By contingently coding this character, the difference in morphology of the splenial symphysis is still recognised (C225), but the elongated symphyses of traditional tomistomines and gavialoids are recovered as homologous.

Most members of this unnamed clade share a linear dorsal profile of the dentary, where preserved (C220-0), except for *Eosuchus minor* and *Penghusuchus pani*. Within Crocodylia, this condition otherwise only occurs in *Mourasuchus*. Previous studies recognised the presence of a linear dorsal profile of the dentary in several traditional members of Tomistominae and Gavialoidea, but this was recovered as convergence. Minor character score changes are made here, but this feature is now optimised as a shared derived feature in these taxa.

A broadly rounded dorsal margin of the infratemporal fenestra (98-1) supports this clade, but this morphology is absent in some gavialoids (*Eosuchus lerichei*), and independently evolved in some crocodyloids (*Crocodylus johnstoni*, *Brochuchus pigotti*, and ‘*Asiatosuchus*’ *depressifrons*). This feature has been recognised in both traditional tomistomines and gavialoids in previous studies (Salas-Gismondi et al., 2019), and only minor character score changes have been made here.

Finally, this unnamed clade is supported by the absence of an external contact between the nasals and naris (46-1). The absence of a nasal-narial contact convergently evolved in *Borealosuchus*, several ‘basal’ alligatoroids (most *Diplocynodon* species and *Leidyosuchus*), and rarely in Crocodyloidea (*Mecistops cataphractus*, *Crocodylus johnstoni*, and *Mekosuchus inexpectatus*). Previous studies discretised the presence or absence of a nasal-narial contact using an unordered multistate character that also described the degree of contact with the premaxilla. Although, as in this study, most traditional tomistomines and gavialoids were scored as lacking nasal-narial contact, this was previously optimised as convergence.

#### Character support for Gavialidae

Gavialidae is diagnosed by seven synapomorphies: (1) infratemporal fenestra size relative to cranial table length = 0.49–0.55 (C7); (2) number of maxillary alveoli = 16 (C17); (3) scapular blade flare = 40° (C21); (4) dentary symphysis adjacent to 13–20 teeth (C221-3, exclusive); (5) no differentiation in dentary alveolus size posterior to 4th tooth (C219-3, ambiguous); (6) neural spine of first postaxial vertebra less than half centrum length (C280-1, shared); and (7) ventral osteoderms absent or poorly developed (C328-0, shared).

A dentary symphysis adjacent to 13–20 alveoli (221-3) is an exclusive synapomorphy of Gavialidae, lengthening further in crownward nodes. As discussed under the synapomorphies for Gavialoidea above, few studies have increased the number of character states to delimit dentary symphysis length (Salas-Gismondi et al., 2015, 2016, 2019). Fewer still have ordered this character (Iijima & Kobayashi, 2019). Character scores in previous studies implied that only traditional gavialoids possess symphyses elongated beyond the 12th dentary alveolus (Salas-Gismondi et al., 2019). In the present study, the elongated condition is also recognised in *Tomistoma schegelii* and *Penghusuchus pani*.

The absence of differentiation in size of dentary alveoli posterior to the 4th dentary tooth (219-3) also characterises Gavialidae, but this occurs convergently in *Mourasuchus* and the non-crocodylian *Isisfordia duncani*. This feature tends to be correlated with lengthening of the rostrum, as seen in all gavialids, *Mourasuchus*, and to a degree in *Isisfordia*; however, it is not present in all longirostrines. For example, several of the earliest diverging gavialoids recovered here, such as *Maroccosuchus*, *Dollosuchoides*, *Thecachampsa*, and ’*Tomistoma*’ *lusitanica*, exhibit a modest enlargement around the 10th, 11th, or 12th alveolus. Whereas the delimitation of this character is essentially identical to previous studies that employ it (Brochu et al., 2012; Jouve, 2016; Narváez et al., 2016), the character scores of a number of taxa have been modified. Notably, a number of longirostrine taxa were scored as missing data in previous studies (Brochu et al., 2012; Narváez et al., 2016), including taxa for which the anatomy is definitely preserved, such as the extant *Tomistoma schlegelii*, as well as numerous fossil taxa (*e.g. Eosuchus lerichei*, *Eosuchus minor*, *Eothoracosaurus mississippiensis*, *Piscogavialis jugaliperforatus*, and *Thecachampsa antiquus*). Although the large amount of missing data in previous analyses does not support the exclusion of traditional tomistomines from Gavialoidea, it results in the optimisation of all these taxa sharing the condition of all other crocodyloids (219-2), whereas several traditional tomistomines actually have undifferentiated posterior dentary alveoli, similar to *Gavialis*.

The absence or extremely poor development of ventral osteoderms (C328-0) is recovered as a shared synapomorphy of Gavialidae. Ventral osteoderms are otherwise only absent in Crocodylinae (all species except *Crocodylus johnstoni*). The preservation of ventral osteoderms is rare, and within Gavialidae the condition is only known in the extant members *Tomistoma schlegelii* and *Gavialis gangeticus*. The delimitation of this character is the same as expressed in previous studies, and character scores are almost identical; however, whereas the absence of ventral osteoderms was previously recovered as an ambiguous synapomorphy of Crocodyloidea, and convergent in Gavialoidea, it is here optimised as a shared derived feature of Gavialidae.

An anteroposteriorly short first postaxial neural spine (C280-1) is recovered as a synapomorphy of Gavialidae. This synapomorphy is ambiguous, as a result of the large amount of missing data within Gavialidae: only four gavialids can be scored for this feature, whereas the remaining taxa are optimised as having the condition. The distribution of character scores is the same as previous studies. In previous analyses, the lengthened neural spine in traditional gavialoids and tomistomines was most parsimoniously optimised as two independent acquisitions of the character state. Here the condition is optimised as a truly homologous feature of Gavialidae.

#### Character support for unnamed gavialoid clade 2

Unnamed gavialoid clade 2 is the least inclusive clade containing *Toyotamaphimeia machikanensis* and *Gavialis gangeticus*. It is diagnosed by fifteen synapomorphies: (1) rostrum length to skull length = 0.68–0.70 (C1); (2) cranial table length to width ratio = 0.64–0.66 (C8); (3) inclination of dorsal margin of external mandibular fenestra = 23° (C19); (4) ratio of ulna to humeral length = 0.61 (C24); (5) postorbital bar anteroposteriorly expanded, elliptical cross section (C89-0, shared); (6) largest maxillary alveolus is number 7 (C147-5, exclusive); (7) absence of a choanal septum (C193-1, ambiguous); (8) surangular-articular suture straight in glenoid fossa (C247-0, ambiguous); (9) presence of diapophysis on axial neural arch (C276-1, ambiguous); (10) axial hypapophysis forked (C278-1, exclusive); (11) width across prezygapophyses constant throughout presacral vertebrae (C288-0, exclusive); (12) presence of preacetabular process of the ilium (C307-0, ambiguous); (13) dorsal outline of iliac blade lacks indentation (309-0, shared); (14) dorsal midline osteoderms unkeeled (C325-0, shared); and (15) dorsal midline osteoderms with anterolateral process (C327-0, ambiguous).

In previous studies, a forked axial hypapophysis (C278-1) was recovered as an ambiguous synapomorphy of Gavialoidea (Narváez et al., 2016) that was not present in any traditional tomistomines or any other crocodylian. Recently, the forked condition has been identified in *Penghusuchus pani* and *Toyotamaphimeia machikanensis* (Iijima & Kobayashi, 2019), supporting a closer relationship between traditional tomistomines and gavialoids than previously recognised.

An anteroposteriorly expanded postorbital bar (C89-0) is a shared synapomorphy of this unnamed clade, present in several non-crocodylians, including *Bernissartia* and *Hylaeochampsa*. Previously, the shared presence of an expanded postorbital bar was optimised as being a reacquired ‘primitive’ feature in both traditional gavialoids and tomistomines. Here the shared condition unites some of these taxa instead. The presence of an expanded postorbital bar in ‘*Tomistoma*’ *lusitanica* and *Thecachampsa* is recovered as an independent reacquisition of the condition.

The largest maxillary alveolus is the seventh in *Toyotamaphimeia* and *Penghusuchus* (C147-5). This is recovered as a synapomorphy of this unnamed clade; however, all other crownward members of this clade have homodont dentition (see unnamed clade 4 below).

This clade is also supported by the absence of a choanal septum (C193-1). This character is new, derived by contingently coding character 152 of Brochu (1997b), but comparisons between character scores are still possible. There have been minor changes to character scores but, as recovered in previous studies, most traditional tomistomines have a choanal septum and most gavialoids do not. Previously, this supported the exclusion of Gavialoidea from the clade (*Borealosuchus* + (Planocraniidae + Brevirostres)), members of which have choanal septa. Some traditional tomistomines are herein recognised as lacking a choanal septum, *e.g. Maroccosuchus*, *Toyotamaphimeia*, and *Penghusuchus*. Whereas the absence of a choanal septum is recovered as an autapomorphy of *Maroccosuchus*, its absence in *Toyotamaphimeia* and *Penghusuchus* supports a closer relationship of these taxa with traditional gavialoids than to traditional tomistomines.

The presence of a bowed surangular-articular suture in the mandibular glenoid fossa (C247-1) is typically recovered as a synapomorphy of Crocodyloidea, supporting the traditional distinction between tomistomines and gavialoids. Here it is recovered as a synapomorphy of Longirostres, but reverses to the plesiomorphic, straight condition (C247-0) at the node comprising *Toyotamaphimeia* and all taxa more closely related to *Gavialis gangeticus*. Changes to character scores here have increased the uncertainty of the condition in several traditional tomistomines, and one gavialoid exhibits the bowed condition (*Argochampsa krebsi*, NHMUK R 36872).

This unnamed clade is also supported by a constant width across the prezygapophyses in presacral vertebrae (C288-0), a condition not shared by any other taxa in this study. Nevertheless, this synapomorphy is ambiguous due to the high proportion of missing data. As such, the plesiomorphic condition is only scored in *Gavialis gangeticus* and *Toyotamaphimeia machikanensis* (Iijima & Kobayashi, 2019) amongst gavialoids. Characters 276, 307, 309, 325 and 327 are discussed above under atavistic characters.

#### Character support for unnamed gavialoid clade 3

Unnamed gavialoid clade 3 is the least inclusive clade containing *Penghusuchus pani* and *Gavialis gangeticus*. It is diagnosed by five synapomorphies: (1) ratio of supratemporal fenestra length to cranial table length = 0.54 (C10); (2) ratio of basioccipital tubera width to occipital condyle width = 1.52–1.60 (C15); (3) maxillary toothrow linear between alveoli 1–4 (C149-1, shared); (4) lingual foramen perforates surangular-articular suture (C253-1, ambiguous); and (5) deltopectoral crest emerges smoothly from proximal end of humerus (C302-0, ambiguous).

In *Penghusuchus pani* and all taxa more closely related to *Gavialis gangeticus*, the maxillary toothrow between alveoli 1–4 is anteroposteriorly orientated (149-1). This contrasts with the laterally flaring condition of essentially all other eusuchians. This derived feature independently evolved in the clade comprising *Gavialosuchus eggenburgensis*, ‘*Tomistoma*’ *lusitanica* and *Thecachampsa*. Otherwise, all other gavialoids share the plesiomorphic condition. This character is new and therefore score comparisons with previous studies are not possible.

This unnamed clade is further supported by the position of the lingual foramen, perforating the surangular-articular suture. Previously, this character was recovered as a shared synapomorphy within Crocodyloidea, present in Crocodylinae, Osteolaeminae, Mekosuchinae, and some traditional tomistomines (not in *Tomistoma schlegelii*, *Toyotamaphimeia*, and *Paratomistoma*). There have been some significant changes to character scores in traditional tomistomine taxa here. *Maroccosuchus* and *Kentisuchus* exhibit a perforation on the surangular entirely, and the condition in *Thecachampsa antiquus* is considered unknown. As a result, this character has a complicated optimisation pattern. The plesiomorphic condition in Gavialoidea is for the lingual foramen to perforate the surangular only (C253-0). The character state changes at the node comprising *Penghusuchus* and all taxa more closely related to *Gavialis gangeticus* (C253-1), and then reverses to the plesiomorphic condition in crownward nodes. Character 302 is discussed above under atavistic characters.

#### Character support for unnamed gavialoid clade 4

Unnamed gavialoid clade 4 is the least inclusive clade containing ‘*Tomistoma*’ *cairense* and *Gavialis gangeticus*. It is diagnosed by five synapomorphies: (1) ratio of rostrum width to cranial table width = 1.1–1.2 (C2); (2) number of maxillary alveoli = 17–20 (C17); (3) protuberance on the dorsolateral margin of the postorbital bar (C90-0, ambiguous); (4) maxillary alveoli homodont (C147-7, shared); and (5) two scars discernible on proximodorsal surface of humerus for *M. teres major* and *M. dorsalis scapulae* (C304-0, shared).

This clade is unambiguously supported by the presence of homodont maxillary dentition (147-7), with the condition unknown only in *Thoracosaurus neocesariensis* and *Portugalosuchus azenhae*. Previous studies have recovered homodont dentition as a synapomorphy of Gavialoidea, with homodonty convergently evolving in isolated cases in Crocodylia, *e.g*. in *Mourasuchus*, *Euthecodon*, and ‘*Tomistoma*’ *cairense*. Previous studies have recovered the latter species as nested within Tomistominae, but here this taxon is more closely related to traditional gavialoids than to any traditional tomistomine. Characters 90 and 304 are discussed above under atavistic characters.

#### Summary of character support for Gavialoidea and Gavialidae

Table 9 summarises the synapomorphies of Gavialoidea and less inclusive clades that support the grouping of traditional tomistomines and gavialoids. Character support uniting these groups has been identified in previous morphological character datasets (Trueman, 1998; Gatesy et al., 2003; Harshman et al., 2003). Whereas these similarities were most parsimoniously optimised as being convergently acquired, here they diagnose Gavialoidea, or less inclusive clades. Four of these characters are exclusively found in traditional tomistomines and gavialoids: C107-1 and C206-0, which are synapomorphies of Gavialoidea, and C278-1 and C288-0, which are synapomorphies of the least inclusive clade comprising *Toyotamaphimeia* and *Gavialis gangeticus*. The remainder are variably present in other crocodylian lineages and therefore provide more ambiguous support for Gavialoidea.

Four characters supporting Gavialoidea or less inclusive clades are new (C149-1, C191-0), recently introduced (C288-9; Iijima & Kobayashi, 2019), or have not previously been applied to crocodylian datasets (C154-0). These characters provide new support that unites some or all traditional gavialoids and tomistomines. Five morphological characters used in previous studies of crocodylian phylogeny did not support a close relationship between these groups; however, modifications to character scores, as well as character construction and/or taxon sampling, generates new support uniting them (C98-1, C136-1, C193-1, C219-3, C224-2). The remaining 18 character states that are newly recovered as synapomorphies of Gavialoidea, or less inclusive clades, were recognised in traditional tomistomines and gavialoids in previous datasets, but were optimised as being convergently acquired in these groups. The large amount of missing data in character scores for gavialoid taxa should also be considered. Some of the synapomorphies of Gavialoidea or less inclusive clades identified here are based on poorly preserved elements of the skeleton (*e.g*. C278 and C328), or which require the comparison of serial variation of interconnected elements that are typically displaced in burial and fossilisation (C288). In such cases, the condition is often only known in two members of a clade, and optimised as present in all others, although the implementation of extended implied weighting partially remedies this problem by down-weighting characters with a high proportion of missing data.

There has always been morphological character support uniting taxa traditionally assigned to Tomistominae and Gavialoidea; however, the similarities between these taxa appear to have been overwhelmed by the similarities between gavialoids and many non-crocodylian neosuchians. Modifications to characters that formerly supported the exclusion of Gavialoidea from Brevirostres have reduced the stemward ‘pull’ on Gavialoidea. Additionally, more widespread similarities have been recognised between traditional tomistomines and gavialoids through new observations (*e.g*. Iijima & Kobayashi, 2019), as well as *via* subtle changes to character delimitation (*e.g*. C136 and C224) and treatment (*e.g*. ordering C224).

#### Are ‘thoracosaurs’ gavialoids?

This study is topologically concordant with molecular datasets in recovering *Gavialis gangeticus* as more closely related to *Tomistoma schlegelii* than to any other extant crocodylian. However, the recovery of several latest Cretaceous and early Paleogene species in Gavialidae is temporally incongruent with molecular estimates for divergence times between *Tomistoma schlegelii* and *Gavialis gangeticus*, which place the timing of this split at ~30–16 Ma (Oaks, 2011; Pan et al., 2021). Most of the taxa resulting in this incongruence are informally known as ‘thoracosaurs’ (*e.g*. Brochu, 2004a). Previously, ‘thoracosaurs’ have been regarded as a paraphyletic group that includes the most stemward gavialoids that are typically from the latest Cretaceous and early Paleogene (Brochu, 2004a, 2006). The consideration of stratigraphic data in combined Bayesian analyses of morphology and molecules provided the first empirical evidence that ‘thoracosaurs’ do not belong in Gavialoidea, nor indeed in Crocodylia (Lee & Yates, 2018). Based on the results of Lee & Yates (2018), ‘thoracosaurs’ comprise a monophyletic group, consisting of *Eothoracosaurus*, *Thoracosaurus* (*T*. *isorhynchus* + *T*. *neocesariensis*), *Argochampsa krebsi*, *Eogavialis africanum*, and *Eosuchus* (*E*. *minor* + *E*. *lerichei*). In particular, the recovery of *Eogavialis africanum* and *Argochampsa krebsi* as ‘thoracosaurs’ and outside of Crocodylia in that study is surprising. Although these taxa are often recovered as ‘intermediate’ forms between ‘thoracosaurs’ and crownward gavialoids, several analyses have recovered them as more closely related to *Gryposuchus* and *Gavialis* than to ‘thoracosaurs’ (*e.g*. Brochu, 2004a; Hua & Jouve, 2004; Jouve et al., 2015; Iijima & Kobayashi, 2019). Nevertheless, the dataset of Lee & Yates (2018) incorporated stratigraphic data in their phylogenetic reconstruction, and the older ages of *Argochampsa* (early Paleocene) and *Eogavialis* (Eocene) must factor into this reconstruction.

In our study, ‘thoracosaurs’ form a polyphyletic assemblage nested in Gavialidae. Furthermore, a putative early crocodylian taxon, *Portugalosuchus azenhae* (Mateus, Puértolas-Pascual & Callapez, 2019), from the Cenomanian (early Late Cretaceous) of Portugal, is recovered as being closely related to *Eothoracosaurus* and *Thoracosaurus neocesariensis*. As such, below we evaluate the contrasting placements of ‘thoracosaurs’.

##### Character support for the inclusion of ‘thoracosaurs’ in Gavialoidea

There are 40 synapomorphies of Gavialoidea or less inclusive clades that potentially unite ‘thoracosaurs’ with other gavialoids (Table S4). Only six of these synapomorphies are known exclusively in Gavialoidea or less inclusive clades (Table 10): (1) anteriorly flaring lateral squamosal grooves (C107-1); (2) maxillary alveoli dorsal to maxillary palate (C158-1); (3) laterally directed capitate process of the laterosphenoid (C206-0); (4) dentary symphysis adjacent to 13–20 teeth (C221-3); (5) forked axial hypapophysis (C278-1); and (6) width across prezygapophyses constant through presacral vertebrae (C288-0).

**Table 10:** Distribution of exclusive gavialoid synapomorphies in ‘thoracosaurs’ and *Portugalosuchus azenhae*.

	107-1	206-0	278-1	288-0	221-3	158-1
Longirostrine character?	No	No	No	No	Yes	Yes
*Thoracosaurus isorhynchus*	1	?	1	?	?	**0**
*Thoracosaurus neocesariensis*	1	0	1	?	?	?
*Eothoracosaurus mississippiensis*	1	0	?	?	3	1
*Eosuchus minor*	1	**1**	1	?	**2**	1
*Eosuchus lerichei*	**0**	0	**0**	?	?	**0**
*Portugalosuchus azenhae*	**0**	?	?	?	?	?
*Eogavialis africanum*	1	0	1	?	3	1
*Argochampsa krebsi*	?	0	?	?	?	1

**Note:**

Emboldened values indicate character states that distinguish these taxa from other gavialoids.

Two of these characters are certainly associated with longirostry (C158-1 C221-3), and thus their presence in ‘thoracosaurs’ and other gavialoids could be explained by convergence in snout length. A further two characters (C107-1, C206-0) are more ambiguously associated with longirostry. It has been suggested that a flaring squamosal groove (C107-1) is also associated with longirostry (Brochu, 2004a; Groh et al., 2020); however, this feature is known only in Gavialoidea (including traditional tomistomines), and does not characterise any other neosuchian longirostrine (*e.g*. Tethysuchia) (Groh et al., 2020). Similarly, it has been suggested that a laterally directed capitate process of the laterosphenoid (C206-0) might be correlated with longirostry (Brochu, 2004a); however, this again has not been identified in other longirostrine neosuchians (Groh et al., 2020). As such, there are four exclusive synapomorphies of Gavialoidea that are not associated with snout length (C107-1, C206-0, C278-1, C288-0), although one of these characters (C288) cannot be assessed in any of the putative ‘thoracosaurs’.

An anteriorly flaring squamosal groove (C107-1) is a synapomorphy of Gavialoidea. Among the putative ‘thoracosaurs’ recovered by Lee & Yates (2018), this condition is present in *Thoracosaurus isorhynchus*, *Thoracosaurus neocesariensis*, *Eothoracosaurus*, *Eogavialis*, and *Eosuchus minor*. The condition is absent in *Eosuchus lerichei* and *Portugalosuchus*, and the condition is unknown in *Argochampsa krebsi*. Some other gavialoids also lack the flaring condition (*e.g. Maomingosuchus petrolica*, ‘*Tomistoma*’ *cairense*, and *Tomistoma schlegelii*).

A laterally directed capitate process of the laterosphenoid (C206-0) is another exclusive synapomorphy of Gavialoidea. This condition is exhibited in *Thoracosaurus neocesariensis*, *Eothoracosaurus*, *Eosuchus lerichei*, *Eogavialis africanum*, and *Argochampsa krebsi*, but is absent in *Eosuchus minor*. The condition is unknown in *Thoracosaurus isorhynchus* and *Portugalosuchus*. Where preserved, the condition is present in other gavialoids, with the exception of *Tomistoma schlegelii* and *Maomingosuchus petrolica*.

A forked axial hypophysis (278-1) is a synapomorphy of the least inclusive clade containing *Toyotamaphimeia machikanensis* and *Gavialis gangeticus*. This condition occurs in all members of this gavialid clade, where preserved, including *Thoracosaurus isorhynchus*, *Thoracosaurus neocesariensis*, *Eosuchus minor*, and *Eogavialis africanum*, but not *Eosuchus lerichei*, which has an unforked axial hypapophysis. The condition in *Argochampsa krebsi*, *Eothoracosaurus*, and *Portugalosuchus* is unknown.

An elongated dentary symphysis adjacent to 13–20 teeth (221-3) is a synapomorphy of Gavialidae. Among ‘thoracosaurs’, it is present in *Eogavialis* and *Eothoracosaurus*. *Eosuchus minor* has a slightly shorter dentary symphysis (221-2), and the condition is unknown in all other ‘thoracosaurs’. The maxillary alveoli are dorsal to the maxillary palate (158-1) in four ‘thoracosaurs’: *Eothoracosaurus*, *Eosuchus minor*, *Eogavialis*, and *Argochampsa*. Whereas the condition is unknown in *Thoracosaurus neocesariensis* and *Portugalosuchus*, *Eosuchus lerichei* and *Thoracosaurus isorhynchus* both exhibit the plesiomorphic condition in which the maxillary alveoli are ventral to the palate. There are a further 32 morphological features which ambiguously support the position of ‘thoracosaurs’ in Gavialoidea. These features are present in some but not all ‘thoracosaurs’, and also occur outside of Gavialoidea (both in other crocodylians, and taxa outside of Crocodylia), and thus might be considered less diagnostic. These characters and their distribution in ‘thoracosaurs’ are listed in Table S4.

##### Character support for the exclusion of ‘thoracosaurs’ from Gavialoidea

Despite the character support suggesting that ‘thoracosaurs’ are gavialoids, there are several morphological features in some ‘thoracosaurs’ that are typically absent in other gavialoids (Table 11). The margins of the orbit are flush against the skull surface (C72-0) in *Eothoracosaurus*, *Eosuchus minor*, *Eosuchus lerichei*, and *Portugalosuchus*. In all other gavialoids they are either upturned (C72-1) or telescoped (C72-2). In *Eothoracosaurus*, *Thoracosaurus neocesariensis*, *Thoracosaurus isorhynchus*, *Eosuchus lerichei*, and *Portugalosuchus*, the frontoparietal suture intersects deeply in the supratemporal fenestra, such that the postorbital-parietal suture is not exposed on the skull table (C75-0). This condition is discretised as the end member of an ordered character. In most traditional tomistomines, the frontoparietal suture is entirely on the skull table (C75-2), whereas all other gavialoids have an intermediate condition where the frontoparietal suture incipiently contacts the supratemporal fenestra (C75-1).

**Table 11:** Characters distinguishing ‘thoracosaurs’ from other gavialoids.

	72-0	75-0	88-0	136-0	144-0	217-0	235-0
*Thoracosaurus isorhynchus*	1	**0**	**0**	**0**	1	1	?
*Thoracosaurus neocesariensis*	?	**0**	**0**	?	?	?	?
*Eothoracosaurus mississippiensis*	**0**	**0**	?	**0**	**0**	**0**	?
*Eosuchus minor*	**0**	1	?	**0**	**0**	1	1
*Eosuchus lerichei*	**0**	**0**	1	**0**	**0**	1	?
*Portugalosuchus azenhae*	**0**	**0**	**0**	**0**	?	?	**0**
*Eogavialis africanum*	1	2	1	1	1	1	1
*Argochampsa krebsi*	1	1	1	1	1	?	?

**Note:**

Emboldened values indicate character states that distinguish these taxa from other gavialoids.

The posteroventral processes of the pterygoids are dorsoventrally expanded (C136-0) in *Eothoracosaurus*, *Thoracosaurus isorhynchus*, *Eosuchus minor*, *Eosuchus lerichei*, and *Portugalosuchus*. This is unlike all other gavialoids, which have dorsoventrally short pterygoid processes.

A medially inset ultimate premaxillary alveolus is recovered as a synapomorphy of Gavialoidea. This condition is absent in several ‘thoracosaurs’, including *Eothoracosaurus*, *Eosuchus minor*, and *Eosuchus lerichei*; however, it is also absent in a few other gavialoids, including *Thecachampsa antiquus* and *Aktiogavialis caribesi*.

Dentary alveoli 3 and 4 are essentially confluent in *Eothoracosaurus* (C217-0) (Brochu, 2004a). This is recovered here as an autapomorphy within Gavialoidea, as all other gavialoids have equally separated dentary alveoli. Otherwise, confluent alveoli are found in *Bernissartia*, *Borealosuchus*, *Leidyosuchus canadensis*, and *Diplocynodon*. A slit-like external mandibular fenestra, lacking a concavity on the angular dorsal margin, is present in *Portugalosuchus* (C235-0). All other gavialoids have a large external mandibular fenestra, producing a concavity on the angular, usually with the foramen intermandibularis caudalis obscured from view (C235-1). The condition in *Eothoracosaurus* was discussed in detail by (Brochu, 2004a). Here it is scored as missing data; however, it is likely that the external mandibular fenestra in *Eothoracosaurus* was either slit-like, or entirely absent. Slit-like external mandibular fenestrae are otherwise rare in Crocodylia, known only in *Mekosuchus*; however, outside of Crocodylia, this condition also occurs in some *Borealosuchus* species. Furthermore, the external mandibular fenestra is plesiomorphically absent in Eusuchia, as can be observed in *Bernissartia*, *Theriosuchus*, and, where preserved, all hylaeochampsids and allodaposuchids.

In several ‘thoracosaurs’, the postorbital-parietal suture passes medial to the orbitotemporal canal, and there is little to no development of a fossa medial to this canal (C88-0). This condition is present in *Thoracosaurus isorhynchus*, *Thoracosaurus neocesariensis*, *Eosuchus minor*, and *Portugalosuchus*. Unlike all the previous characters discussed, this condition is otherwise known solely outside of Crocodylia, in *Allodaposuchus precedens* and *Shamosuchus*.

##### Constrained searches

To test the significance of the phylogenetic position of ‘thoracosaurs’ and *Portugalosuchus*, four constrained searches were performed on Analysis 1.3. The analysis of Mateus, Puértolas-Pascual & Callapez (2019) recovered the traditional morphological topology, with *Portugalosuchus* as the sister taxon to all non-gavialoid crocodylians; however, those authors noted that support for this relationship was low, and that *Portugalosuchus* might not belong within Crocodylia. As such, the first constraint excludes *Portugalosuchus* from Crocodylia, and ‘thoracosaurs’ (as recovered by Lee & Yates (2018, fig. 2), *i.e. Argochampsa*, *Eothoracosaurus*, *Thoracosaurus*, *Eogavialis*, and *Eosuchus*) are set as floating taxa such that they could be included within Crocodylia, but were not forced to do so. The second constraint excludes ‘thoracosaurs’ from Crocodylia, while *Portugalosuchus* floats. Due to the labile position of planocraniids and *Borealosuchus* in other analyses, these were also set as floating taxa in both constraints.

Another hypothesis to be explored is whether ‘thoracosaurs’ are indeed gavialoids, but outside of the crown group Gavialidae. Such a relationship might reconcile the strong gavialoid affinities of ‘thoracosaurs’ with the stratigraphically young age of Gavialidae inferred from molecular analyses (~30 Ma) (Oaks, 2011; Pan et al., 2021). In fact, we should expect to find ‘basal’ gavialoids of approximately the same age as some ‘thoracosaurs’, since the earliest crocodyloids, such as *Jiangxisuchus nankangensis* (Li, Wu & Rufolo, 2019) and *Prodiplocynodon langi* (Mook, 1941a), are also known from the latest Cretaceous. This hypothesis was tested using a third positive constraint, which forced *Tomistoma schlegelii* and *Gavialis gangeticus* to form a clade (*i.e*. Gavialidae). All non-‘thoracosaur’ gavialoids recovered in the original unconstrained Analysis 1.3 (including *Portugalosuchus*) were set as floating taxa, which could be recovered within Gavialidae, but were not forced to do so.

A fourth constrained analysis was conducted to test the affinities of *Argochampsa* and *Eogavialis* with other gavialoids. As under Constraint 2, *Thoracosaurus*, *Eosuchus*, and *Eothoracosaurus* were forced outside of Crocodylia, but *Eogavialis* and *Argochampsa* were set as floating taxa, along with *Portugalosuchus*, *Borealosuchus*, and Planocraniidae.

Constraint 1 results in an insignificant tree length increase of 47.6 steps (Templeton test *p* > 0.05). *Portugalosuchus* is nested in *Borealosuchus*, in the stem of Crocodylia, whereas ‘thoracosaurs’ remain nested in the crown group Gavialidae (Fig. S9).

**Figure 22:**
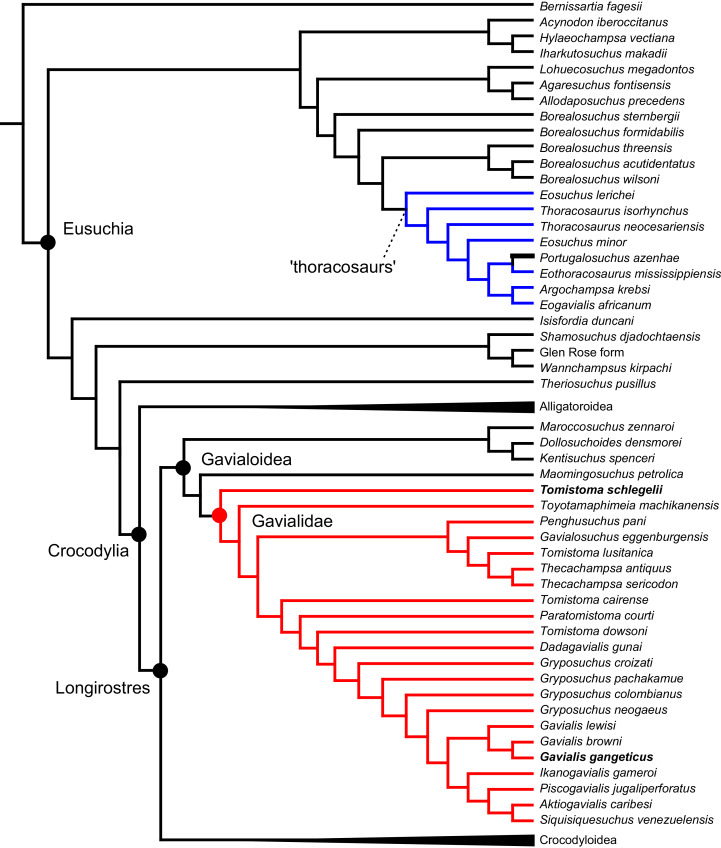
Strict consensus of the three MPTs resulting from Analysis 1.3 with ‘thoracosaurs’ constrained to be excluded from Crocodylia. Crown gavialids highlighted in red, ‘thoracosaurs’ highlighted in blue.

Constraint 2 results in a significant tree length increase of 192.1 steps (*p* < 0.01) (Fig. 22). ‘Thoracosaurs’ are recovered as deeply nested in a clade of non-crocodylian eusuchians, comprising Hylaeochampsidae, Allodaposuchidae, and a paraphyletic *Borealosuchus*. *Borealosuchus sternbergii* and *Borealosuchus formidabilis* are successively nested taxa ‘basal’ to ‘thoracosaurs’ + (*Borealosuchus threeensis* + (*Borealosuchus wilsoni* + *Borealosuchus acutidentatus*)). Within the ‘thoracosaur’ clade, *Eosuchus lerichei*, *Thoracosaurus isorhynchus*, *Thoracosaurus neocesariensis*, and *Eosuchus minor* are successively nested taxa that lie outside of two sister clades: ((*Eothoracosaurus mississippiensis* + *Portugalosuchus azenhae*) + (*Eogavialis africanum* + *Argochampsa krebsi*)). The principal difference to Gavialidae is the inclusion of the clade comprising (*Gavialosuchus eggenburgensis* + (‘*Tomistoma*’ *lusitanica* + (*Thecachampsa antiquus* + *Thecachampsa sericodon*)) as an early diverging gavialid sister taxon to *Penghusuchus pani*. In the unconstrained analysis, this clade was recovered outside of the crown group. *Moroccosuchus zennaroi* + (*Kentisuchus spenceri* + *Dollosuchoides densmorei*) is recovered as the earliest diverging gavialoid clade, as in the unconstrained analysis. A number of additional minor topological changes also occur (Fig. 22).

**Figure 23:**
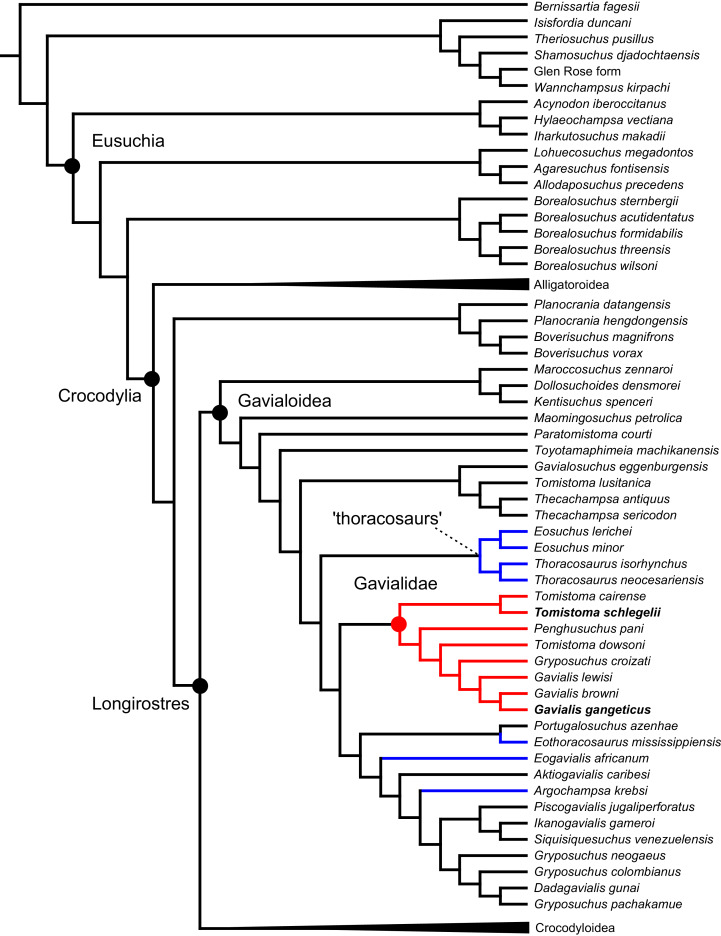
Strict consensus of the three MPTs resulting from Analysis 1.3 with ‘thoracosaurs’ constrained to be excluded from Gavialidae. Crown gavialids highlighted in red, ‘thoracosaurs’ highlighted in blue.

Under the third constraint, tree length increases by a statistically insignificant 100.6 steps (*p* > 0.05). The topology of Gavialoidea and composition of Gavialidae changes dramatically from both the unconstrained topology, and the topology under Constraint 2 (Fig. 23). The taxonomic content of Gavialidae is significantly reduced to eight taxa. All species of *Gryposuchus*, except for *G*. *croizati*, are excluded from Gavialidae. *Piscogavialis jugaliperforatus*, *Siquisiquesuchus venezuelensis*, and *Ikanogavialis gamerois* are similarly recovered outside of the crown group. ‘Thoracosaurs’ comprise a polyphyletic group within Gavialoidea, resulting in several ghost lineages, similar to the unconstrained analysis. Both species of *Eosuchus* and *Thoracosaurus* form a clade stemward of Gavialidae and its unnamed sister clade. The latter includes all other putative ‘thoracosaurs’, although these form a paraphyletic array. *Maroccosuchus*, *Kentisuchus*, and *Dollosuchoides* are once again recovered as the earliest diverging clade of gavialoids.

**Figure 24:**
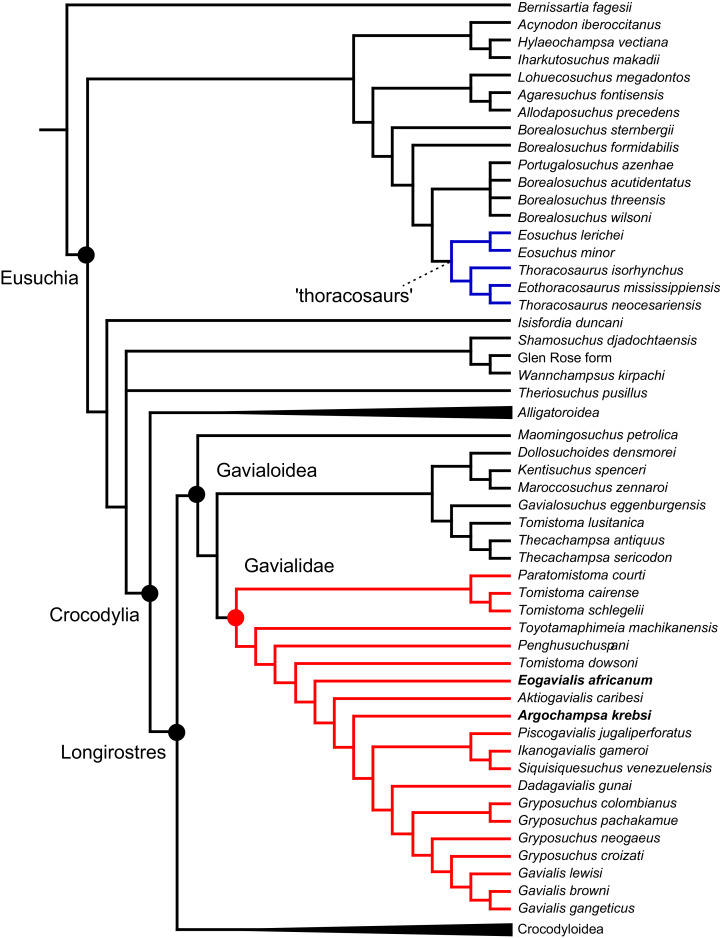
Strict consensus of the three MPTs resulting from Analysis 1.3 with ‘thoracosaurs’ constrained to be excluded from Crocodylia, but with *Eogavialis* and *Argochampsa* allowed to float. Crown gavialids highlighted in red, ‘thoracosaurs’ highlighted in blue.

Under the fourth constraint (Fig. 24), *Argochampsa* and *Eogavialis* are recovered within Gavialidae. All remaining ‘thoracosaurs’ and *Portugalosuchus* are recovered in a clade of early diverging non-crocodylian eusuchians, including hylaeochampsids, allodaposuchids, and *Borealosuchus*, as under Constraint 1. Gavialoidea is now shorn of ‘thoracosaurs’, and the relationships of *Eogavialis* and *Argochampsa* are identical to the unconstrained Analysis 1.3. A Templeton test indicates that the differences in character state distributions between this tree and that of the unconstrained analysis (1.3) are insignificant (*p* > 0.05).

##### Summary

In summary, whereas the exclusion of *Portugalosuchus* from Crocodylia results in an insignificant tree length increase, constraining the exclusion of ‘thoracosaurs’ produces significantly less parsimonious trees. ‘Thoracosaurs’ are still recovered as nested within Gavialoidea when forced outside of Gavialidae, resulting in a small, insignificant increase in tree length but resulting in numerous additional ghost lineages. *Eogavialis* and *Argochampsa* share a number of derived gavialid features, suggesting a closer relationship with later appearing Neogene taxa, such as *Gryposuchus* and *Gavialis*, than to the ‘early thoracosaurs’. The discovery of new non-crocodylian eusuchians may be the key to determining the phylogenetic affinities of ‘thoracosaurs’. Although ‘thoracosaurs’ share a number of features with gavialoids, very few of these are exclusive synapomorphies of the clade: they generally occur more widely in Crocodylia, are often also present in non-crocodylian eusuchians, and many of them are associated with longirostry. ‘Thoracosaurs’ may ultimately be shown to form a distinct non-crocodylian longirostrine clade. Indeed, this study has started to find character support uniting some ‘thoracosaurs’ with non-crocodylian eusuchians (*e.g*. the morphology of the orbitotemporal canal, (C88)); however, these similarities are overwhelmed by shared features with gavialoids.

### Anatomical support and implications for the systematics of Crocodyloidea

The interrelationships of Crocodyloidea are among the most poorly supported in this phylogeny (average Bremer support = 1.98); however, a number of the interrelationships are highly concordant with existing molecular and morphological hypotheses, as is discussed.

Crocodyloidea is supported by seven synapomorphies: (1) ratio of snout length to cranium length = 0.55–0.56 (C1); (2) ratio of posterior rostrum width to cranial table width = 1.47–1.52 (C2); (3) infratemporal fenestra length relative to cranial table length = 0.53–0.55 (C7); (4) dorsomedial margin of the orbit flush with skull surface (C72-0, ambiguous); (5) width of the supraoccipital on the skull table greater than half the parietal width (C78-1, ambiguous); (6) palatine ramus of cranial nerve V greater than or equal to half the diameter of the 6th maxillary alveolus (C159-1, exclusive); and (7) anterior palatine process at the same level or posterior to the anterior margin of the suborbital fenestra (C162-2, ambiguous).

Only C159-1 is exclusively known in Crocodyloidea; however, this condition reverses in *Australosuchus clarkae* and more crownward taxa. Outside of Crocodyloidea, an extremely shortened anterior palatine process (C162-2) is known only in *Lohuecosuchus megadontos*. Within Crocodyloidea, this condition is lost at the node comprising Mekosuchinae + Crocodylidae, and it is independently reacquired in some crocodyloids.

The most significant difference in Crocodyloidea to previous morphological studies is the exclusion of Tomistominae, which is usually recovered as the sister clade to Mekosuchinae + (Crocodylinae + Osteolaeminae) (Brochu, 2000; Brochu et al., 2012). With the exclusion of Tomistominae, new interrelationships emerge between the three main crocodyloid clades: Crocodylinae, Osteolaeminae, and Mekosuchinae. Furthermore, the taxonomic content and interrelationships of these clades show differences to previous studies.

#### Asiatosuchus germanicus, ‘Crocodylus’ affinis and ‘Asiatosuchus’ depressifrons – basal crocodyloids or stem longirostrines?

**Figure 25:**
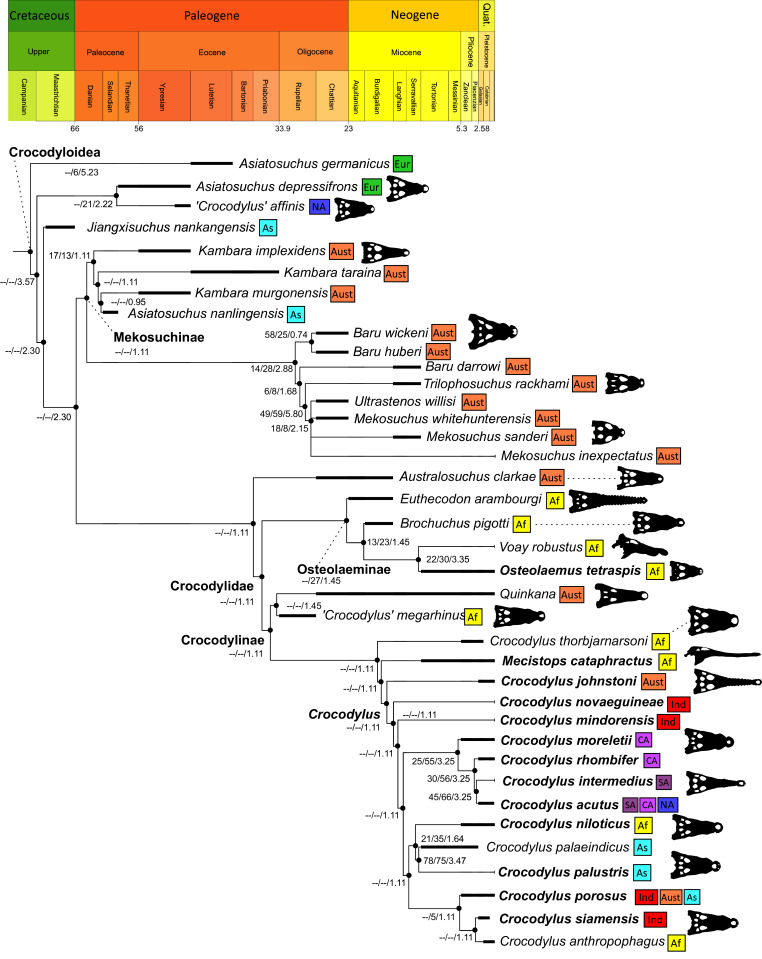
Time-calibrated phylogeny of Crocodyloidea from Analysis 1.3. Ages = Ma. Support values: Bootstrap/Jackknife/Bremer. Abbreviations: As, Asia; Aust, Australasia; CA, Central America; Eur, Europe; Ind, Indo-Pacific (Malay Archipelago); NA, North America; SA, South America.

Compared to combined morphological and molecular analyses (which also recover Longirostres), the topology of Crocodyloidea differs in the affinities of *Asiatosuchus germanicus*, ‘*Crocodylus*’ *affinis*, and ‘*Asiatosuchus*’ *depressifrons* (Fig. 25). Here, these taxa are consistently recovered as the earliest diverging crocodyloids, outside of the group including Mekosuchinae and Crocodylidae. By contrast, all combined analyses recover these taxa in the stem of Longirostres, *i.e*. outside of the clade uniting Gavialoidea and Crocodyloidea (Gatesy et al., 2003; Gold, Brochu & Norell, 2014; Lee & Yates, 2018; Iijima & Kobayashi, 2019). This hypothesis was tested by constraining Analysis 1.3 to recover these taxa in the stem of Longirostres, which resulted in an insignificant tree length increase of 2.2 steps (Templeton test, *p* > 0.05, Fig. S10). As originally proposed (Li, Wu & Rufolo, 2019), the latest Cretaceous Asian taxon, *Jiangxisuchus nankangensis*, is recovered as one of the earliest diverging crocodyloids, (Li, Wu & Rufolo, 2019), instead of a ‘basal’ alligatoroid as recently found by Massonne et al. (2019). However, it is important to note that several taxa belonging to their newly described alligatoroid clade, Orientalosuchinae, were not included in this dataset, meaning that the position of *Jiangxisuchus* will require further testing.

#### Phylogenetic affinities and taxonomic content of Mekosuchinae

Mekosuchinae is weakly supported (Bremer support = 1.11) by five synapomorphies: (1) ratio of infratemporal fenestra length to cranial table length = 0.33–0.40 (C7); (2) ratio of supratemporal fenestra width to length = 0.72–0.77 (C11); (3) quadrate condyle with prominent notch covering one-third of mediolateral width (C118-1, ambiguous); (4) anterior maxillary ramus of the ectopterygoid separated from the suborbital fenestra by the maxilla (C177-1, ambiguous); and (5) anteroposteriorly orientated ridges lateral to the choanae (C198-1, exclusive).

Mekosuchinae is recovered as the sister clade to *Australosuchus clarkae* + Crocodylidae in Analysis 1.3, but as the sister clade of Crocodylinae in analyses 1.1 and 2.3. Analysis 3.3 was unable to resolve the interrelationships of Osteolaeminae, Crocodylinae, and Mekosuchinae. The position of Mekosuchinae within Crocodylia is not well resolved in previous studies. The earliest phylogenetic analyses of the clade included a good sample of mekosuchines, but few members of Crocodylinae and Osteolaeminae, and a limited number of morphological characters (Willis, 1993; Salisbury & Willis, 1996). Furthermore, even in recent analyses, few studies have included more than three mekosuchines. Morphology-only analyses that recover the traditional topology (*i.e*. Crocodyloidea including Tomistominae) have placed Mekosuchinae as: (1) the sister clade to Tomistominae + (Crocodylinae + Osteolaeminae) (Salisbury & Willis, 1996); (2) in a polytomy with Tomistominae, Osteolaeminae and Crocodylinae (Yates & Pledge, 2017); or, most commonly, (3) in a polytomy with ‘*Crocodylus*’ *megarhinus* + (Osteolaeminae + Crocodylinae) (*e.g*. Brochu, 2000; Brochu et al., 2010, 2012; Brochu & Storrs, 2012; Jouve et al., 2015; Massonne et al., 2019). With the recovery of Longirostres in combined morphological and molecular analyses, different topologies are recovered still. Whereas Lee & Yates (2018) recovered Mekosuchinae as the sister clade to Longirostres, the analyses of Gatesy et al. (2003) and Gold, Brochu & Norell (2014) positioned Mekosuchinae as the sister clade to Crocodylidae, similar to the results presented here. Constraining Mekosuchinae to be recovered in the stem of Longirostres results in an insignificant tree length increase of 6.7 steps (Templeton test, *p* > 0.05, Fig. S11).

#### Phylogenetic intrarelationships of Mekosuchinae

Comparisons of the internal relationships of Mekosuchinae are limited to a handful of studies, given that few include more than three representatives (Salisbury & Willis, 1996; Stein, Hand & Archer, 2016; Yates & Pledge, 2017; Lee & Yates, 2018). The internal relationships of Mekosuchinae are highly congruent between analyses 1.1, 1.3, 2.3 and 3.3. Furthermore, they broadly align with existing hypotheses and are highly stratigraphically congruent. All species of the early appearing generalist form, *Kambara*, form a clade that is sister to the later-appearing altirostral and blunt snouted taxa *Baru*, *Mekosuchus*, *Ultrastenos*, and *Trilophosuchus* (Salisbury & Willis, 1996; Brochu, 2003; Lee & Yates, 2018; Ristevski et al., 2020) (Fig. 25). Furthermore, as in Lee & Yates (2018), *Trilophosuchus* and *Mekosuchus* are more closely related to each other than to *Baru*, which is paraphyletic. Despite these similarities, two principal differences in the taxonomic content are found. Whereas two putative mekosuchines, *Australosuchus clarkae* and *Quinkana*, are recovered outside of Mekosuchinae here, the Paleocene East Asian crocodyloid, ‘*Asiatosuchus*’ *nanlingensis*, is recovered within Mekosuchinae.

##### Is ‘Asiatosuchus’ nanlingensis a mekosuchine?

‘*Asiatosuchus*’ *nanlingensis* is known from mostly fragmentary mandibular remains, and its phylogenetic affinities have rarely been tested. When included in phylogenetic analyses, its relationships are usually unresolved within Crocodyloidea (Wang, Sullivan & Liu, 2016; Wu, Li & Wang, 2018) and it has even been recovered as a ‘basal’ alligatoroid by some authors (Li, Wu & Rufolo, 2019; Massonne et al., 2019). Analyses 1.1, 1.3, 2.3 and 3.3 recover ‘*Asiatosuchus*’ *nanlingensis* in Mekosuchinae. If correct, this relationship has significant implications for the biogeography of the clade, which has until now been recognised as an endemic Australasian radiation.

Support for the inclusion of ‘*Asiatosuchus*’ *nanlingensis* in Mekosuchinae is relatively weak. None of the synapomorphies of Mekosuchinae can be scored in this taxon. Furthermore, of the eight synapomorphies that support the least inclusive clade comprising ‘*Asiatosuchus*’ *nanlingensis* + *Kambara implexidens*, only two can be scored in the former species: (1) dorsal lobe of the dentary symphysis extends further posteriorly than the ventral lobe (C226-0); and (2) the surangular-articular suture is straight within the glenoid fossa (C247-0). The first of these is ambiguous, unknown in *Kambara taraina* and *Kambara murgonensis*, and shared with most crocodylines, ‘*Crocodylus*’ *affinis*, and most caimanines. A straight surangular-articular suture (C247-0) is also ambiguous. Within Crocodyloidea, this condition is otherwise only known in *Mecistops cataphractus*; however, it is the plesiomorphic condition in Eusuchia, occurring in all alligatoroids and most gavialoids. A sulcus excavating the dorsolateral surface of the surangular (C246-1) unites *Kambara taraina*, *Kambara murgonensis*, and ‘*Asiatosuchus*’ *nanlingensis*. This synapomorphy is again ambiguous, occurring in *Crocodylus anthropophagus*, ‘*Crocodylus*’ *megarhinus*, and ‘*Asiatosuchus*’ *depressifrons*. No alligatoroids exhibit this sulcus, and the only non-crocodyloid taxa to exhibit it are *Bernissartia fagesii*, *Borealosuchus sternbergii*, and *Kentisuchus spenceri*. Furthermore, this feature might also be size-dependent, with a more prominent sulcus in larger-bodied species (Wu, Li & Wang, 2018). The sister relationship between ‘*Asiatosuchus*’ *nanlingensis* and *Kambara murgonensis* is supported by one ambiguous synapomorphy: dentary symphysis adjacent to 6–8 alveoli (C221-1). Elongation of the dentary symphysis to 6–8 alveoli is very common in Crocodylia, present in *Asiatosuchus germanicus*, ‘*Crocodylus*’ *affinis*, ‘*Asiatosuchus*’ *depressifrons*, several crocodylines and alligatorines, as well as some non-crocodylian eusuchians.

Due to poor preservation, only three synapomorphies of Alligatoroidea can be compared with ‘*Asiatosuchus*’ *nanlingensis*, but all of these are absent. ‘*Asiatosuchus*’ *nanlingensis* lacks subequal anterior processes of the surangular (C240-0) (present in almost all alligatoroids), and it exhibits a surangular that extends to the posterior tip of the retroarticular process (C245-0) (truncated in most alligatoroids). Finally, the retroarticular process exceeds the dorsal margin of the articular glenoid fossa in ‘*Asiatosuchus*’ *nanlingensis* (C251-1), contrasting with the low retroarticular processes of most alligatoroids (C251-0).

Constraining Analysis 1.3 to recover ‘*Asiatosuchus*’ *nanlingensis* outside of Mekosuchinae results in a statistically insignificant tree length increase of 0.8 steps (Templeton test *p* > 0.05, Fig. S12). Under this constraint, ‘*Asiatosuchus*’ *nanlingensis* is recovered within Crocodylinae as the sister species of *Mecistops cataphractus*, immediately in the stem of the crown genus Crocodylus. This constraint results in significant changes to the topology of Crocodyloidea. Mekosuchinae is now recovered in crown group Crocodylidae as the sister clade to Crocodylinae. Constraining the search to recover ‘*Asiatosuchus*’ *nanlingensis* within Alligatoroidea also results in an insignificant tree length increase of 33.65 steps (Templeton test, *p* > 0.05). Under this constraint, ‘*Asiatosuchus*’ *nanlingensis* is recovered as an early diverging alligatoroid. The removal of this taxon from Crocodyloidea has a significant impact on topology within Crocodyloidea and Longirostres. *Asiatosuchus germanicus*, ‘*Asiatosuchus*’ *depressifrons*, ‘*Crocodylus*’ *affinis*, *Jiangxisuchus*, and *Australosuchus* all become stem longirostrines. Furthermore, Mekosuchinae is again recovered within the crown group Crocodylidae, as the sister clade of Crocodylinae.

In summary, ‘*Asiatosuchus*’ *nanlingensis* is most parsimoniously recovered within Mekosuchinae based on several ambiguous synapomorphies shared with *Kambara*. Forcing its inclusion into other crocodylian clades, including Alligatoroidea, results in insignificant tree length increases, but major differences in the topology of Longirostres.

##### Are Australosuchus clarkae and Quinkana mekosuchines?

*Australosuchus clarkae* has consistently been recovered within Mekosuchinae, either as the earliest diverging member (Lee & Yates, 2018), or closely related to *Kambara* (Salisbury & Willis, 1996). There are a number of characters which support the exclusion of *Australosuchus* from Mekosuchinae, as recovered here. All members of Mekosuchinae exhibit a prominent notch on the quadrate condyle, where preserved, which covers approximately a third of the quadrate width (C118-1). This condition is an ambiguous synapomorphy of Mekosuchinae, as it occurs convergently in most alligatoroids and *Borealosuchus*. However, this condition is not present in any other crocodyloid, including *Australosuchus*. Separation of the anterior process of the ectopterygoid from the suborbital fenestra (C176-1) is also recovered as a synapomorphy of Mekosuchinae, that is absent in *Australosuchus*. This is ambiguous, as it can only be scored in *Kambara implexidens*, *Mekosuchus sanderi*, *Baru wickeni*, and *Baru huberi*. Furthermore, *Trilophosuchus* exhibits the plesiomorphic condition. Outside of Mekosuchinae, this condition only occurs in *Mecistops cataphractus*. The presence of anteriorly directed ridges lateral to the choanae (C198-1) is the most robust synapomorphy of Mekosuchinae; although the presence of this feature cannot be scored for seven putative mekosuchines, it is absent in all other eusuchians. No specimens of *Australosuchus* preserve the palate sufficiently, and so the condition is unknown in this taxon. Finally, *Australosuchus* shares a small opening for the palatine ramus of cranial nerve V (C159-0) with Osteolaeminae and Crocodylinae. Mekosuchinae and all other stemward crocodyloids exhibit an enlarged foramen (C159-1).

Despite being represented by four species (Molnar, 1982c; Megirian, 1994; Willis & Mackness, 1996; Willis, 1997a), *Quinkana* is scored as an exemplifier due to the extremely limited material that comprises the genus. Furthermore, there has not been a revision of any of the species of *Quinkana* since their initial descriptions. The sister relationship of *Quinkana* and ‘*Crocodylus*’ *megarhinus* recovered here is supported by two ambiguous synapomorphies: (1) sulcus on the ventrolateral margin of the jugal and/or maxilla (C96-1); and (2) anterior palatine process at the same level or posterior to the anterior margin of the suborbital fenestra (C162-2). A ventrolateral sulcus appears multiple times in crocodyloid species, as well as convergently within Gavialoidea. Within Mekosuchinae, only *Baru wickeni* exhibits this sulcus. A reduced anterior process of the palatine has previously been identified as a common feature of several Australasian fossil crocodylians, and it is the plesiomorphic condition in Crocodyloidea (Willis, Murray & Megirian, 1990; Willis, 1993). A reduced anterior palatine process also characterises some mekosuchines, but here this is optimised as multiple acquisitions of this condition. This character was previously binary, lacking a precise delimitation between character states. Here, a further character state has been added which further subdivides the length of the anterior process. Consequently, fewer taxa are scored for the highly shortened condition (C162-2). Furthermore, of the three characters providing support for Mekosuchinae, *Quinkana* can only be scored for one (C177), for which it has the opposite condition (C177-0) to all other mekosuchines (C177-1).

Constraining Analysis 1.3 to recover *Australosuchus* and *Quinkana* within Mekosuchinae results in a significant tree length increase of 5.5 steps (Templeton test: *p* < 0.05, Fig. S13). Under this constraint, *Australosuchus* and *Quinkana* are recovered as the earliest successively branching taxa in Mekosuchinae. The remaining topology is almost identical to the unconstrained analysis, except that *Asiatosuchus germanicus* is recovered as the sister species of ‘*Crocodylus*’ *affinis* + ‘*Asiatosuchus*’ *depressifrons*.

#### Osteolaeminae

The sister taxon relationship of Osteolaeminae with Crocodylinae (Fig. 25), as well as the relationships among osteolaemines, is largely congruent with previous studies (Brochu, 2007a). Principal differences relate to the synapomorphies supporting Osteolaeminae and its internal nodes. Osteolaeminae is supported by five synapomorphies: (1) ratio of infratemporal fenestra maximum length to anteroposterior cranial table length = 0.49 (C7) (2); minimum angle subtended by lateral cranial table edge and sagittal axis of skull = 6° (C9); (3) anteroposteriorly orientated preorbital ridges (C30-1); (4) descending lamina of the squamosal on the quadrate ramus of the paroccipital process (C113-1); and (5) ectopterygoid forming medial wall of posterior maxillary alveoli (C175-2). Preorbital ridges (C30-1) and a descending squamosal lamina (C113-1) were previously recovered as ambiguous synapomorphies of Osteolaeminae (Brochu, 2007a); however, characters 7, 9, and 175 are newly recognised synapomorphies. All five synapomorphies are ambiguous; for example, the size of the infratemporal fenestrae in Osteolaeminae is approximately equal to most crocodylines, and it varies considerably in Osteolaeminae (*e.g*. ~30% of cranial table length in *Osteolaemus tetraspis* and 50% in *Voay robustus*). The angular difference between the lateral edge of the cranial table and the sagittal plane is low in most osteolaemines, resulting in square cranial tables. This contrasts with the trapezoidal shape of most crocodylines. Although this is true of *Euthecodon*, *Brochuchus*, and *Osteolaemus*, *Voay* exhibits a strongly trapezoidal skull table (angular difference ~23°). Contact between the ectopterygoid and the posterior maxillary alveoli (C175-2) is also ambiguous. This character has received considerable changes to delimitation and scoring from previous analyses. Here it is optimised as a synapomorphy of Osteolaeminae, but it also occurs in almost all crocodylines.

Within Osteolaeminae, *Euthecodon arambourgii* and *Brochuchus pigotti* are successively nested taxa that are ‘basal’ to the clade comprising (*Osteolaemus tetraspis* + Voay robustus). This contrasts with the results of Brochu (2007a) and Cossette et al. (2020), who recovered a sister relationship between *Brochuchus* and *Euthecodon arambourgii*. The clade comprising *Brochuchus* + (*Osteolaemus tetraspis* + *Voay robustus*) is supported by a posteroventrally sloping ventral orbital margin (C94-1).

As recovered in nearly all previous studies (Brochu, 2007a; Brochu et al., 2012; Cossette et al., 2020), *Osteolaemus tetraspis* and *Voay robustus* are sister taxa (though see Hekkala et al. (2021) for a crocodyline placement for *Voay* following the incorporation of ancient DNA). This relationship is supported by six continuous and four discrete synapomorphies: (1) rostrum length = 0.54–0.56 (C1); (2) width to length ratio of the external naris = 1.0 (C3); (3) cranial table length to width ratio = 0.68–0.75 (C8); (4) supratemporal fenestra size relative to cranial table length = 0.30 (C10); (5) ratio of width across the basioccipital tubera to occipital condyle width = 1.56 (C16); (6) number of maxillary alveoli = 13 (C17); (7) frontoparietal suture concavo-convex (C76-0, shared); (8) supratemporal fenestrae with overhanging rims (C81-1, shared); (9) premaxillary alveoli equally separated (C145-0, shared); and (10) margins of the angular and surangular everted to form a flange (C239-1, shared).

Three of these discrete synapomorphies are newly recognised, although they are all shared. A concavo-convex frontoparietal suture (C76-0) is also present in all crocodylines, some mekosuchines, ‘*Asiatosuchus*’ *depressifrons*, and ‘*Crocodylus*’ *affinis*; however, it is absent in *Euthecodon arambourgi* and *Brochuchus*. The equal separation of premaxillary alveoli (C145-0) is the plesiomorphic condition in Crocodylia, but this is lost in most crocodyloids. The presence of everted margins of the surangular and angular (C239-1) occurs in three other members of Longirostres, although all are mekosuchines (*Mekosuchus inexpectatus*, *Mekosuchus whitehunterensis*, and *Ultrastenos*). Two additional synapomorphies were considered to unite *Voay* and *Osteolaemus* in previous analyses: overhanging rims of the supratemporal fenestrae, and a short anterior palatine process (Brochu, 2007a). The former is also recovered here (C81-1); however, the palatine process is significantly longer in *Voay* (C162-1) than in *Osteolaemus tetraspis* (C162-2), and it is instead similar to the condition in most crocodylines.

#### Crocodylinae

Although its placement outside of the genus *Crocodylus* is consistent with previous studies, the position of ‘*Crocodylus*’ *megarhinus* within Crocodyloidea differs. This species is typically recovered in a polytomy with Mekosuchinae and Crocodylidae (*e.g*. Brochu, 2000; Delfino, Piras & Smith, 2005; Brochu & Storrs, 2012; Lee & Yates, 2018). Here, ‘*Crocodylus*’ *megarhinus* is recovered as an early diverging member of Crocodylinae. This relationship is supported by three synapomorphies: (1) an ectopterygoid which extends anteriorly more than two-thirds the length of the suborbital fenestra (C174-1); (2) dorsoventrally short posteroventral processes of the pterygoid (C136-1); and (3) a concavo-convex frontoparietal suture (C76-0). However, none of these features unambiguously unite ‘*Crocodylus*’ *megarhinus* with Crocodylinae. The frontoparietal suture is also concavo-convex in some osteolaemines, mekosuchines, and ‘basal’ crocodyloids. Although the elongated ectopterygoid (C174-1) is absent in all osteolaemines, it occurs in several mekosuchines, such as *Baru*, *Kambara taraina*, and *Trilophosuchus*. Dorsoventrally short posteroventral pterygoid processes are absent in all other crocodyloids, except *Ultrastenos*, but they are present in almost all members of Gavialoidea.

##### Intrarelationships of the crown genus Crocodylus

Although weakly supported, the intrarelationships of crown *Crocodylus* species recovered here show a number of similarities to molecular studies. As in Oaks (2011), the three Australasian species, *C*. *johnstoni*, *C*. *novaeguineae*, and *C*. *mindorensis*, are the earliest diverging members of the crown genus (Fig. 25). However, whereas they formed a clade in Oaks (2011) and Pan et al. (2021), they are recovered as successively nested species in this study. Like most morphological and all molecular studies, Neotropical *Crocodylus* forms a monophyletic group, within which *C*. *acutus* and *C*. *intermedius* are sister species. Also in agreement with molecular studies is the close relationship found between *C*. *porosus* and *C*. *siamensis* (Meganathan et al., 2010; Oaks, 2011; Pan et al., 2021). The principal difference to molecular studies is that *C*. *niloticus* is here more closely related to the remaining Australasian (*C*. *porosus*) and Indo-Pacific *Crocodylus* species, as well as *C*. *anthropophagus* from the Plio-Plesitocene of East Africa, than to Neotropical *Crocodylus*.

This study is the first to recover a sister relationship between *C*. *palaeindicus* (Miocene–Pliocene of Indo-Pakistan) and the extant mugger crocodile, *C*. *palustris*, of the Indian subcontinent and Iran. This relationship is moderately well-supported (Jackknife: 75, Bremer 3.5). Direct ancestry between these species has long been suggested based on their overlapping geographic range and similar morphology (Lydekker, 1886; Mook, 1933). The two species are united by three synapomorphies: (1) premaxilla-maxilla suture at the level or anterior to the posterior margin of the naris (C49-1, shared); (2) posterior margin of the incisive foramen rounded (C139-0, shared); and (3) anterior extent of the ectopterygoid less than two-thirds the length of the suborbital fenestra (C174-0, shared).

##### The phylogenetic affinities of Mecistops cataphractus

The phylogenetic affinities of the African slender-snouted crocodile, *Mecistops cataphractus*, are a source of ongoing debate. Most morphological and several molecular phylogenies (usually based on single genes) recover Osteolaeminae and *Mecistops* as successive outgroups of *Crocodylus* (*e.g*. Brochu, 2000; McAliley et al., 2006; Li et al., 2007; Brochu et al., 2010). By contrast, other molecular studies (typically based on multiple gene loci), as well as combined analyses, recover *Mecistops* within Osteolaeminae (*e.g*. Gatesy et al., 2003; Schmitz et al., 2003; Man et al., 2011; Oaks, 2011; Pan et al., 2021; Hekkala et al., 2021). Here, we recover *Mecistops* as more closely related to *Crocodylus*. Furthermore, the giant Plio-Pleistocene African species, *Crocodylus thorbjarnarsoni*, is recovered as the sister taxon of *Mecistops* + *Crocodylus*. Previously, *Crocodylus thorbjarnarsoni* was recovered as the sister taxon of *Crocodylus anthropophagus*, but in a large polytomy with all Neotropical *Crocodylus* species, *C*. *niloticus*, *C*. *palustris*, *C*. *siamensis*, and *C*. *palaeindicus* (Brochu & Storrs, 2012). Nevertheless, these results should be treated as tentative given the low support for *Mecistops* + *Crocodylus* (Bremer support = 1.11), and since constraining Analysis 1.3 to recover *Mecistops* within Osteolaeminae results in an insignificant tree length increase of 16.2 steps (Templeton test, *p* > 0.05, Fig. S14).

### Implications for the evolutionary and biogeographic history of Crocodylia

#### Eusuchia and the origin of Crocodylia

**Figure 26:**
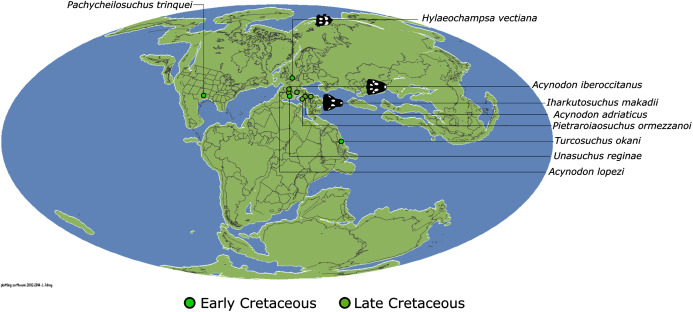
The distribution of named species referred to Hylaeochampsidae. Global palaeogeographical reconstruction at 125 Ma from Fossilworks (http://fossilworks.org/) (Alroy, 2013) based on data in the Paleobiology Database (https://paleobiodb.org/).

By contrast with some recent hypotheses (Turner, 2015; Turner & Pritchard, 2015; Narváez et al., 2016; Schwarz, Raddatz & Wings, 2017), *Theriosuchus* and Paralligatoridae are not recovered in Eusuchia (see also Groh et al., 2020), removing evidence for a Late Jurassic origin of the latter group. Other putative early eusuchian occurrences tend to be extremely fragmentary (*e.g*. Yi et al., 2016). As such, the earliest unambiguous appearances of Eusuchia come from representatives of Hylaeochampsidae in the Barremian of western Eurasia, comprising *Hylaeochampsa vectiana* from the United Kingdom (Clark & Norell, 1992), *Turcosuchus okani* from Turkey (Jouve et al., 2019), and *Unasuchus reginae* from Spain (Brinkmann, 1992; Jouve et al., 2019) (Fig. 26). The nested position of *Hylaeochampsa* implies long ghost lineages (~40 myr) for other members of Hylaeochampsidae that appear in the Late Cretaceous, *i.e. Iharkutosuchus makadii* from the Santonian of Hungary (Ősi, Clark & Weishampel, 2007), and the multispecific *Acynodon* from the Campanian–Maastrichtian of southern Europe (Buscalioni, Ortega & Vasse, 1997; Delfino, Martin & Buffetaut, 2008). Although Albian occurrences from Italy (*Pietrarosuchus ormezzanoi*; Buscalioni et al., 2011) and the USA (*Pachycheilosuchus trinquei*; Rogers, 2003) might partly fill this gap, these species tend to be recovered in a more ‘basal’ position within Hylaeochampsidae (*e.g*. Jouve et al., 2019), or outside of Eusuchia altogether (*e.g*. Narváez et al., 2015). However, these long ghost lineages can be explained by sampling failure: non-marine crocodyliform fossils are almost unknown from the early Late Cretaceous of Europe and North America (Martin & Delfino, 2010; Puértolas-Pascual et al., 2016; Mannion et al., 2019). This interval coincides with a marine transgression and a substantial decline in the preservation of terrestrial deposits on these two continents (see also Puértolas-Pascual et al., 2016), with this sampling gap mirrored in the record of other contemporaneous terrestrial groups (Mannion & Upchurch, 2011).

**Figure 27:**
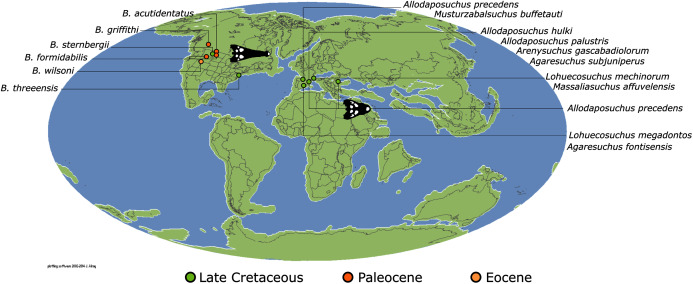
The distribution of named species referred to Allodaposuchidae and *Borealosuchus*. Global palaeogeographical reconstruction at 66 Ma from Fossilworks (http://fossilworks.org/) (Alroy, 2013) based on data in the Paleobiology Database (https://paleobiodb.org/).

Allodaposuchidae represents an endemic European clade of stem crocodylians that first appeared in the Campanian (or possibly the Santonian; Ősi et al., 2016), and went extinct at the K/Pg boundary (Buscalioni et al., 2001; Delfino et al., 2008; Puértolas-Pascual, Canudo & Moreno-Azanza, 2013; Blanco et al., 2014; Narváez et al., 2015; Martin et al., 2016; Narváez et al., 2016; Blanco, 2021) (Fig. 27). Most recent studies have placed Allodaposuchidae as the sister clade to Hylaeochampsidae (*e.g*. Narváez et al., 2015, 2016), whereas our analyses recover the former in a position closer to Crocodylia, similar to the recent study of Blanco (2021). Although a long ghost lineage remains regardless, our topology necessitates only a single unsampled lineage leading to Allodaposuchidae + Crocodylia.

Remains unambiguously referable to the North American eusuchian *Borealosuchus*, the sister taxon to Crocodylia herein, first appear in Maastrichtian deposits, with the genus passing through the K/Pg boundary until its disappearance in the early Eocene (Brochu, 1997a; Brochu et al., 2012) (Fig. 27). Unequivocal members of crown group Crocodylia are first recorded in the Campanian, ~80 Ma (Brochu, 2003, see below for discussion). Given that these occurrences are approximately contemporaneous with the stratigraphically oldest known members of Allodaposuchidae, this might be regarded as robust evidence that these clades originated in the latest Cretaceous and that some of their earliest representatives have been sampled. However, the evolutionary history of these clades might still be obscured by sampling biases. Furthermore, if the Portuguese neosuchian *Portugalosucus azenhae* is a member of Crocodylia, then this would push the origin of Crocodylia (and Allodaposuchidae) back to the Cenomanian, ~100 Ma (Mateus, Puértolas-Pascual & Callapez, 2019) (Table 12). Molecular analyses place the split between Alligatoroidea and other crocodylians at around 100–80 Ma (Roos, Aggarwal & Janke, 2007; Oaks, 2011; Green et al., 2014; Pan et al., 2021), which concurs with either of these scenarios.

**Table 12:** Earliest fossil occurrences and minimum divergence estimates of eusuchian clades compared with molecular divergence estimates (from Oaks, 2011).

	Age (Ma)		
**Clade**	**This study**	**Oaks (2011)**	**Earliest appearing member/s of clade**
Eusuchia	130–122	–	*Hylaeochampsa vectiana*
Allodaposuchidae	78–66	–	*Allodaposuchus precedens*
*Borealosuchus*	70–66	–	*Borealosuchus sternbergii* and *Borealosuchus threeensis*
Crocodylia	100–95	100–81	*Portugalosuchus azenhae* (earliest appearing gavialoid)
Alligatoroidea	78–72	100–81	*Leidyosuchus canadensis*
Crocodyloidea	70–66	100–81	*Jiangxisuchus nankangensis*
Alligatoridae	72–66	69–61	*Brachychampsa montana* (earliest appearing caimanine)
Alligatorinae	66–62	69–61	*Navajosuchus mooki*
*Alligator*	37.2–33.9	–	*Alligator prenasalis*
Crown *Alligator*	13–10	59–30	*Alligator mefferdi*
Crown caimanines	70–66	42–20	*Bottosaurus harlani*
Planocraniidae	61–59	–	*Planocrania datangensis*
Longirostres	100–95	70–40	*Portugalosuchus azenhae*
Mekosuchinae	62 – 59 (*56–48)	–	‘*Asiatosuchus*’ *nanlingensis* or *Kambara murgonensis
Crocodylidae	34–28	32–19	‘*Crocodylus*’ *megarhinus* (earliest appearing crocodyline)
Osteolaeminae	23–20	32–19	*Euthecodon arambourgi*
Crown *Crocodylus*	~12	17–8	*Crocodylus palaeindicus*
Gavialidae	100–95	31–16	*Portugalosuchus azenhae*
Tomistominae	41–38	31–16	*Paratomistoma courti*
*Gavialis*	11.6–5.3	–	*Gavialis browni*

#### Alligatoroidea

As traditionally reconstructed, Alligatoroidea seems to have originated in North America, with the earliest appearing representatives known from the Campanian, including the ‘basal’ members *Leidyosuchus canadensis* and *Deinosuchus* (Lambe, 1907; Holland, 1909; Brochu, 1997a; Schwimmer, 2002; Rivera-Sylva et al., 2011; Farke et al., 2014; Cossette & Brochu, 2020; Mohler, McDonald & Wolfe, 2021). Several additional latest Cretaceous alligatoroids are known from North America, comprising taxa (*Albertosuchus langstoni*, *Bottosaurus*, *Brachychampsa*, and *Stangerochampsa mccabei*) usually regarded as non-alligatorid alligatoroids (*e.g*. Brochu, 1999a, 2011; Hastings et al., 2013). However, consistent with a small number of recent analyses (Bona et al., 2018; Cossette & Brochu, 2018; Cossette, 2021), these taxa are recovered as caimanines. This also accords with most estimates for the timing of divergence between *Alligator* and *Caiman* based on molecular data (Table 12) (Roos, Aggarwal & Janke, 2007; Oaks, 2011), and suggests that the initial diversification of Alligatoroidea occurred prior to the K/Pg mass extinction, 66 Ma, rather than in its aftermath. Pan et al. (2021) estimated a more recent divergence time for *Alligator* and *Caiman* of 56–50 Ma; however, that study did not include well-established calibration points for Alligatoridae based on unequivocal early Paleocene occurrences of the clade.

Although Caimaninae is today restricted to Central and South America (Grigg & Kirshner, 2015; Roberto et al., 2020), it increasingly appears likely that the clade originated in North America, especially given the total absence of crocodylians currently known from South America during the Cretaceous (*e.g*. Cidade, Fortier & Hsiou, 2019). Whereas most of these taxa form an endemic North American clade that comprises the earliest diverging caimanine lineage, *Bottosaurus* is deeply nested within Caimaninae (see also Cossette & Brochu, 2018; Cossette, 2021). This indicates the presence of a second caimanine lineage in the latest Cretaceous of North America and, potentially, a much more complicated and unsampled evolutionary and biogeographic history for this clade that might have involved multiple early dispersals between the Americas. However, we caveat this with the fact that *Bottosaurus* is known from fragmentary materials and its phylogenetic position is weakly supported; it remains possible that characters diagnosing crownward clades in Caimaninae are homoplastic in presumptive ‘basal’ alligatoroids (*e.g. Bottosaurus*) (Cossette, 2021).

Earlier studies suggested the presence of alligatoroids in the latest Cretaceous of Europe too (Martin, 2007, 2010b; Delfino, Martin & Buffetaut, 2008), but these taxa (*Acynodon*, *Allodaposuchus*) have since been universally recovered outside of Crocodylia (*e.g*. Narváez et al., 2015), and there is no longer any unambiguous evidence of Alligatoroidea on this continent before the K/Pg boundary (Puértolas-Pascual et al., 2016). Massonne et al. (2019) recently erected Orientalosuchina for a newly recovered clade of early diverging East Asian alligatoroids. In addition to several early Paleogene species that were not included in the present study (*Eoalligator chunyii*, *Krabisuchus siamogallicus*, *Oreintalosuchus naduongensis*, and *Protoalligator huiningensis*), Massonne et al. (2019) also recovered *Jiangxisuchus nankangensis*, from the Maastrichtian of China (Li, Wu & Rufolo, 2019), in Orientalosuchina. If correct, this would suggest that Alligatoroidea dispersed out of North America earlier than previously thought. However, our analyses recover *Jiangxisuchus* as an early diverging crocodyloid, in agreement with Li, Wu & Rufolo (2019). Greater sampling of East Asian taxa in phylogenetic analyses will be necessary to fully test these competing hypotheses. There is no unequivocal fossil record of Alligatoroidea from Gondwanan continents during the Cretaceous. Referrals of teeth from the Maastrichtian of India to various crocodylian taxa (*e.g. Brachychampsa* and *Allognathosuchus*; Rana & Sati, 2000) are not supported herein: these elements can only be assigned to Crocodyliformes.

**Figure 28:**
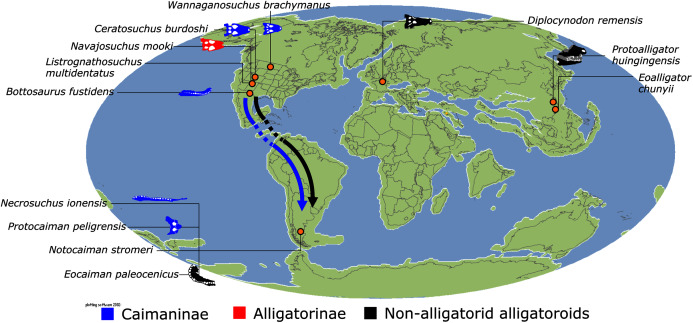
The distribution of named alligatoroids in the Paleocene. Global palaeogeographical reconstruction at 60 Ma from Fossilworks (http://fossilworks.org/) (Alroy, 2013) based on data in the Paleobiology Database (https://paleobiodb.org/).

In the early Paleocene of North America, the first alligatorines appear in the fossil record, represented by *Navajosuchus mooki* (Simpson, 1930; Brochu, 2004b). As such, the Paleocene of North America was characterised by the presence of: (1) alligatorines; (2) at least two caimanine lineages that survived the K/Pg mass extinction, as evidenced by *Bottosaurus* (Erickson, 1998; Cossette & Brochu, 2018; Cossette, 2021) and late Paleocene representatives (*Ceratosuchus burdoshi* (Schmidt, 1938; Bartels, 1984) and *Wannaganosuchus brachymanus* (Erickson, 1982)) of the early-diverging North American clade; and possibly (3) some additional non-alligatorid alligatoroids, represented by the incomplete and poorly studied remains of *Listrognathosuchus multidentatus* (Mook, 1930; Brochu, 1997a) (Fig. 28).

Meanwhile, the first South American caimanines appeared in the early Paleocene, with *Necrosuchus ionensis*, *Notocaiman stromeri*, and *Protocaiman peligrensis* in Argentina (Rusconi, 1937; Simpson, 1937; Brochu, 2011; Bona & Barrios, 2015; Bona et al., 2018; Cidade, Fortier & Hsiou, 2020). Despite their early stratigraphic appearance, *Necrosuchus* and *Protocaiman* are deeply nested in Caimaninae, resulting in long ghost lineages leading to several Neogene South American caimanines. *Eocaiman* also first appears in the early Paleocene of South America, represented by *E*. *palaeocenicus* in Argentina (Bona, 2007), with additional *Eocaiman* species (Simpson, 1930; Pinheiro et al., 2013; Godoy et al., 2020) present in the early–middle Eocene of Argentina and Brazil. Uniquely to our study, *Eocaiman* is not recovered within Caimaninae, but is instead part of a paraphyletic array of alligatoroids that lie outside of Alligatoridae. If correct, this would have a significant impact on our understanding of the biogeographic history of Alligatoroidea, given that all South American alligatoroids have previously been universally recovered as caimanines (*e.g*. Brochu, 1999a, 2003, 2011; Bona et al., 2018; Cidade, Fortier & Hsiou, 2019). As such, in addition to at least one caimanine lineage dispersing into South America close to the K/Pg boundary (Brochu, 1999a, 2011; Hastings et al., 2013; Bona et al., 2018; Cossette & Brochu, 2018), our results indicate that a non-caimanine alligatoroid lineage also reached South America around this time (Fig. 28). However, as with the conclusions regarding the phylogenetic position of *Bottosaurus*, we caveat these results with the fact that these stratigraphically early South American alligatoroids are all known from highly fragmentary specimens; thus, this surprising position of *Eocaiman*, and the deeply nested placement of the other taxa, awaits further testing following the discovery of more complete remains.

Although it is clear that multiple terrestrial groups dispersed between the Americas during the latest Cretaceous (*e.g*. Rage, 1981; Gayet et al., 1992), the geological evidence for this route is less apparent (*e.g*. Ezcurra & Agnolín, 2012). Most authors suggest that the likely dispersal route was *via* the proto-Greater Antillean Arc, which has been hypothesised to have formed a landbridge during the late Campanian–Maastrichtian (Iturralde-Vinent & MacPhee, 1999; Hedges, 2006; Iturralde-Vinent, 2006), although this might only have been a filter barrier, rather than a continuous land route (*e.g*. Fanti, 2012). If this connection was only present in the Cretaceous, this would imply that these alligatoroid lineages most likely arrived in South America prior to the K/Pg mass extinction (Hastings et al., 2013) and that their apparent absence is a sampling artefact.

**Figure 29:**
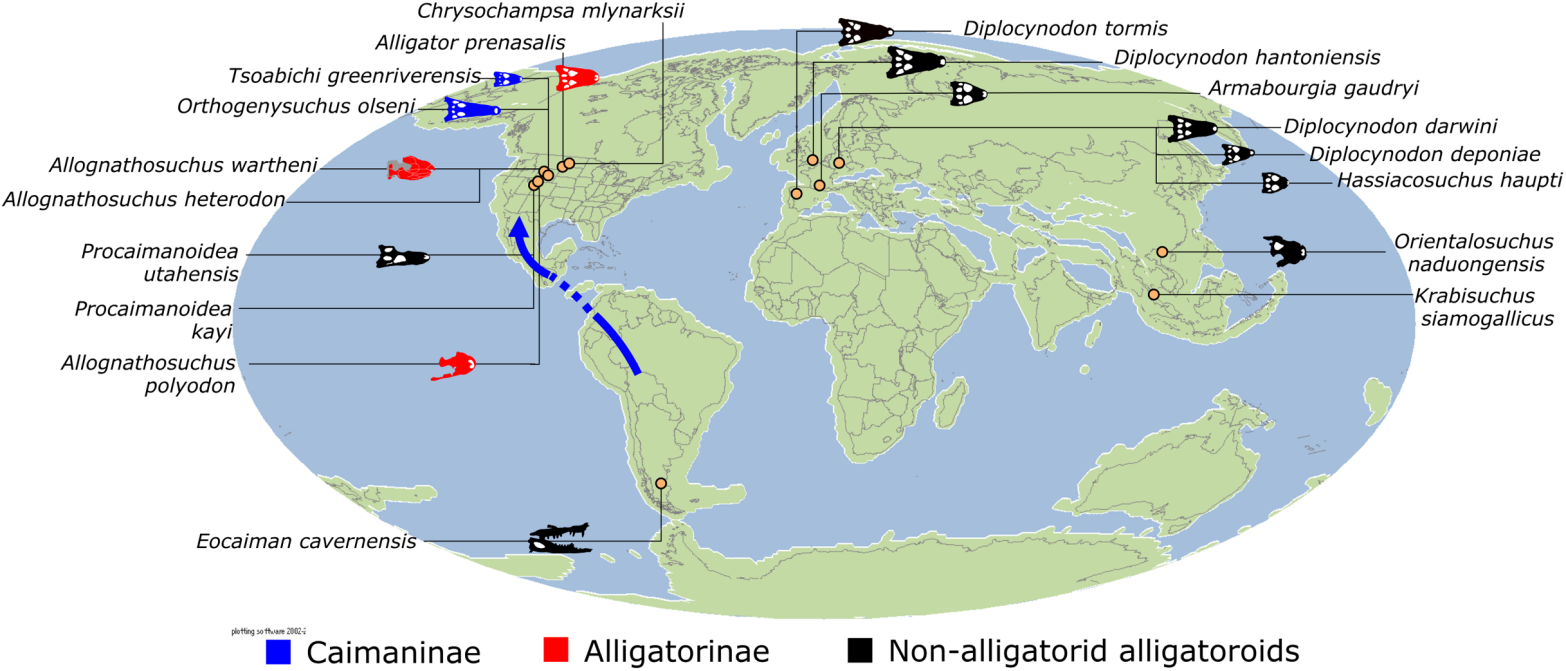
The distribution of named and valid alligatoroid species in the Eocene. Global palaeogeographical reconstruction at 40 Ma from Fossilworks (http://fossilworks.org/) (Alroy, 2013) based on data in the Paleobiology Database (https://paleobiodb.org/).

A presumed reinvasion of North America by South American caimanines occurred by the early Eocene (Fig. 29), evidenced by the appearance of *Tsoabichi greenriverensis* (Brochu, 2010), which does not appear to be closely related to *Bottosaurus* (see also Cossette & Brochu, 2018). Indeed, more than one alligatoroid lineage has been suggested to have reinvaded North America during this interval, given that the contemporaneous species *Orthogenysuchus olseni* (Mook, 1924) is also recovered as a distantly related caimanine (Brochu, 1999a, 2010; Hastings et al., 2013). However, the means of any such dispersal into North America is uncertain, given that there was no clear continuous terrestrial connection between the Americas during the Paleogene (Iturralde-Vinent, 2006), and an island chain or emergent landmass (the Greater Antilles-Northern Lesser Antilles) only probably became emergent in the late Eocene (Montes et al., 2012; Philippon et al., 2020). As such, either the ancestors of *Orthogenysuchus* and *Tsoabichi* were present, but unsampled, in the latest Cretaceous–Paleocene of North America, or these lineages dispersed across a marine barrier (Brochu, 2010; Hastings et al., 2013). The former is difficult to reconcile given the nested position of both species within Caimaninae and the quality of the North American fossil record, whereas the latter is problematic because of the salt intolerance that characterises extant alligatoroids (Taplin et al., 1982). However, given that some extant alligatorids are known to periodically inhabit saltwater environments (*e.g*. Grigg et al., 1998; Brochu & Carbot-Chanona, 2015), the crossing of a small marine barrier might be the most likely scenario. The phylogenetic relationships and biogeographic history of the recently erected caimainine *Chinatichampsus wilsonorum* from the middle Eocene of North America (Stocker, Brochu & Kirk, 2021) remains uncertain.

North American alligatorines are represented by several early Eocene species that have been referred to *Allognathosuchus* (Brochu, 2004b), but then there is a subsequent gap in that continent’s fossil record, prior to the first appearance of *Alligator* (*A*. *prenasalis*) in the late Eocene (Loomis, 1904; Brochu, 1999a; Whiting & Hastings, 2015). This gap was previously partly filled by the middle Eocene taxon *Procaimanoidea* (Mook, 1941b; Gilmore, 1946; Wassersug & Hecht, 1985), which has consistently been recovered as an alligatorine in previous studies (*e.g*. Brochu, 1999a, 2004b; Massonne et al., 2019). However, here we recover *Procaimanoidea* outside of Alligatoridae, forming a clade with the middle–late Eocene European species *Arambourgia gaudryi* (De Stefano, 1905; Kälin, 1939) and *Hassiacosuchus haupti* (Weitzel, 1935). Although its position as the sister taxon to Alligatoridae is novel to our analyses, some previous studies have also indicated a close relationship between the three species forming this clade (Mook, 1941b; Brochu, 2004b). This clade appears to have a long unsampled history that extends into the latest Cretaceous.

A similar ghost lineage is implied for the endemic European non-alligatorid alligatoroid *Diplocynodon* (Fig. 29), which first appears either in the late Paleocene (if *D*. *remensis* belongs to this genus (Martin et al., 2014)) or the middle Eocene (Delfino & Smith, 2012). These gaps might partly be explained by the limited availability of suitable Paleocene European deposits (Martin et al., 2014; Mannion et al., 2019), and there are rare, indeterminate occurrences from throughout the Paleocene of Europe that have been attributed to alligatoroids (Weigelt, 1940; Van Dyck, 1986; Smith et al., 2014) and could conceivably belong to these ghost lineages.

**Figure 30:**
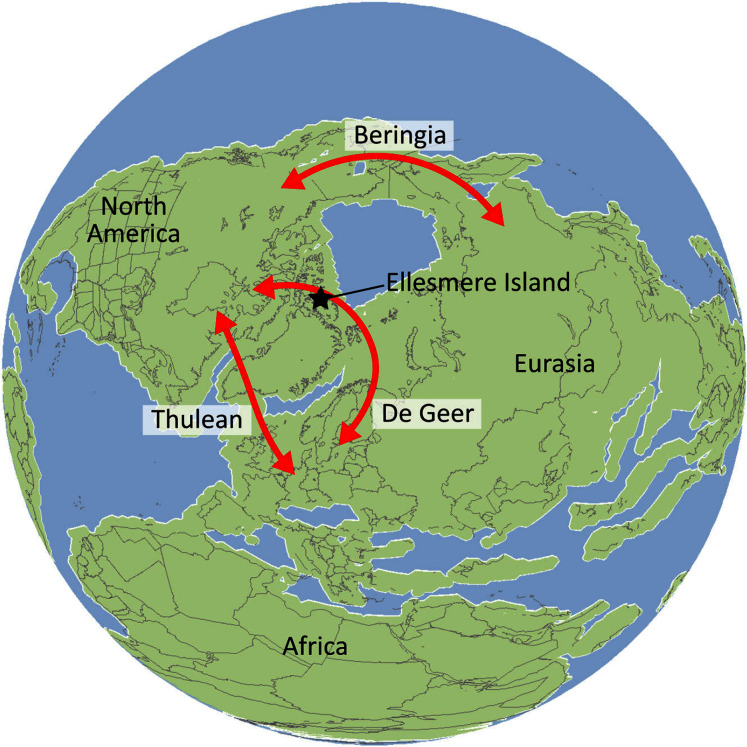
Summary of possible paleobiogeographic routes available from the latest Cretaceous–early Paleogene, after Brikiatis (2014). Global palaeogeographical reconstruction centered on the North Pole at 66 Ma from Fossilworks (http://fossilworks.org/) (Alroy, 2013). Star indicates the location of an indeterminate alligatorid from the early Eocene, based on data in the Paleobiology Database (https://paleobiodb.org/).

Combined with the presence of non-alligatorid alligatoroids (Orientalosuchina) in East Asia (Fig. 29), at least by the early–middle Paleocene (*Eoalligator chunyii* and *Protoalligator huiningensis*) (Martin & Lauprasert, 2010; Skutschas et al., 2014; Wang, Sullivan & Liu, 2016; Massonne et al., 2019), these clades imply biotic exchange between North America and Eurasia. The most likely route to explain the affinities of North American and East Asian taxa was *via* the Bering landbridge (Beringia) (Brochu, 1999a; Wang, Sullivan & Liu, 2016; Massonne et al., 2019) (Fig. 30). This connected northwestern North America with northeastern Asia during intervals of the latest Cretaceous and Paleocene (Brikiatis, 2014). European taxa could conceivably then have dispersed *via* Asia, although the apparent close relationship of European taxa with North American taxa suggests that this explanation is less likely. Instead, the De Geer route might have provided a high latitude connection between Greenland and Fennoscandia during the Maastrichtian–early Paleocene (Fig. 30), with a more southerly (Thulean) route between Greenland and western Europe possible in the late Paleocene (Brikiatis, 2014) (see also Buffetaut, 1985a; Kotsakis, Delfino & Piras, 2004) (Fig. 30). Although the subtropical distribution of crocodylians today argues against such high latitude dispersal routes, the occurrence of alligatorid remains in early Eocene deposits on Ellesmere Island, in the Canadian Arctic (Estes & Hutchison, 1980), demonstrates not only their polar presence (paleolatitude of ~76°), but also their existence along at least the De Geer route (Fig. 30).

The alligatoroid fossil record is sparse in the Oligocene, represented almost entirely by occurrences of *Diplocynodon* (*D*. *muelleri*) in western Europe (*e.g*. Piras & Buscalioni, 2006; Macaluso et al., 2019). Otherwise, there are rare North American occurrences of *Alligator* that might be earliest Oligocene, in addition to reports of late Oligocene specimens (Whiting & Hastings, 2015), and some fragmentary remains have been attributed to caimanines from the late Oligocene of Peru (Antoine et al., 2016). This dearth of Oligocene occurrences characterises much of the global record of Crocodylia in general (Markwick, 1998; Mannion et al., 2019; De Celis, Narváez & Ortega, 2020; Solórzano et al., 2020). Although sampling bias almost certainly plays a role in this apparent decline, it also likely reflects genuine latitudinal range retraction during the Eocene–Oligocene transition (Markwick, 1998; Martin, 2010a; Mannion et al., 2015; Whiting & Hastings, 2015; Jouve, Khalloufi & Zouhri, 2019; Macaluso et al., 2019; Stocker, Brochu & Kirk, 2021), which was characterised by global cooling and increased aridity (*e.g*. Zachos et al., 2001; Zanazzi et al., 2007; Hren et al., 2013; Li et al., 2016). *Diplocynodon* remained abundant in western Europe until its last appearance in the middle Miocene, after which no further alligatoroids are known from Europe (Martin, 2010a; Martin & Gross, 2011; Aráez et al., 2017; Rio et al., 2020; Vasilyan, 2020; Chroust et al., 2021).

**Figure 31:**
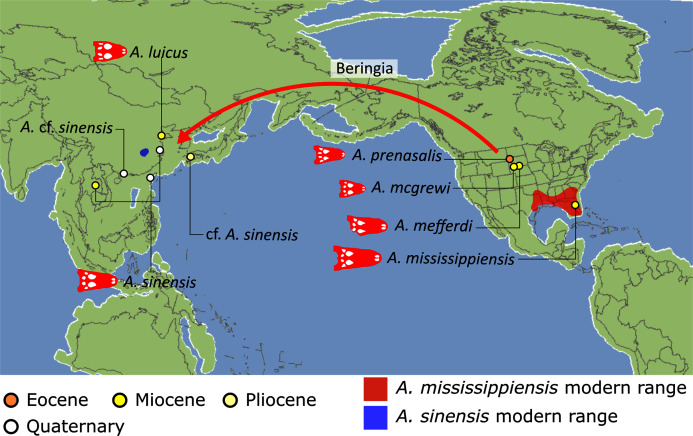
The distribution of *Alligator*. Global palaeogeographical reconstruction at 6 Ma from Fossilworks (http://fossilworks.org/) (Alroy, 2013) based on data in the Paleobiology Database (https://paleobiodb.org/). Note that only the oldest occurrence of *A*. *mississippiensis* is shown, because numerous specimens can be assigned to this species from the Quaternary, all of which approximately fall within its present-day range.

Most species of *Alligator* made their first appearance in the Miocene. These are predominantly North American (Mook, 1923, 1946; Schmidt, 1941; White, 1942; Brochu, 1999a; Snyder, 2007), and include the earliest occurrences of the American alligator, *A*. *mississippiensis*, in the late Miocene, ~8 Ma (Whiting, Steadman & Vliet, 2016, see also Whiting & Head, 2020) (Fig. 31). This extant species is generally thought to have been the only North American member of *Alligator* to survive into the Pliocene (Brochu, 1999a; Whiting, Steadman & Vliet, 2016), although Stout (2020) recently erected *A*. *hailensis* for remains from the early Pleistocene of Florida, which would indicate the persistence of a second North American species of *Alligator*.

A small number of Miocene Asian occurrences have also been assigned to *Alligator*. As well as an isolated tooth (Chow & Wang, 1964), the most notable occurrence is *Alligator luicus* from the middle Miocene of China, which was erected based on a skull and partial postcranial skeleton (Li & Wang, 1987) (Fig. 31). A skull from either the late Miocene or Pleistocene of Thailand was also briefly described and figured by Claude et al. (2011, fig. 3), who assigned it to *Alligator* cf. *sinensis*. The earliest unequivocal occurrence of the Chinese alligator, *Alligator sinensis*, comes from the late Pliocene of Japan (Iijima, Takahashi & Kobayashi, 2016), with Pleistocene remains known from mainland China and Taiwan (Shan, Cheng & Wu, 2013; Iijima, Takahashi & Kobayashi, 2016). No other alligatoroid genus is currently recognised from the Neogene of Asia or North America. There is substantial discordance between the fossil record of *Alligator* and molecular estimates for the divergence of *A*. *mississippiensis* and *A*. *sinensis*, which has been placed at 58–31 Ma (Oaks, 2011; Pan et al., 2021; see discussion in Massonne et al., 2019) (Table 12). *Alligator luicus* has only received a brief description and is yet to be incorporated into a phylogenetic analysis; determining its relationship to other species of *Alligator*, especially *A*. *sinensis*, remains critical to resolving the evolutionary and biogeographic history of *Alligator*. Depending on the interrelationships of the genus, the presence of these two Asian species indicates one or more dispersals from North America to Asia during the Neogene, probably *via* Beringia (Brochu, 1999a, 2003; Snyder, 2007; Iijima, Takahashi & Kobayashi, 2016; Massonne et al., 2019).

**Figure 32:**
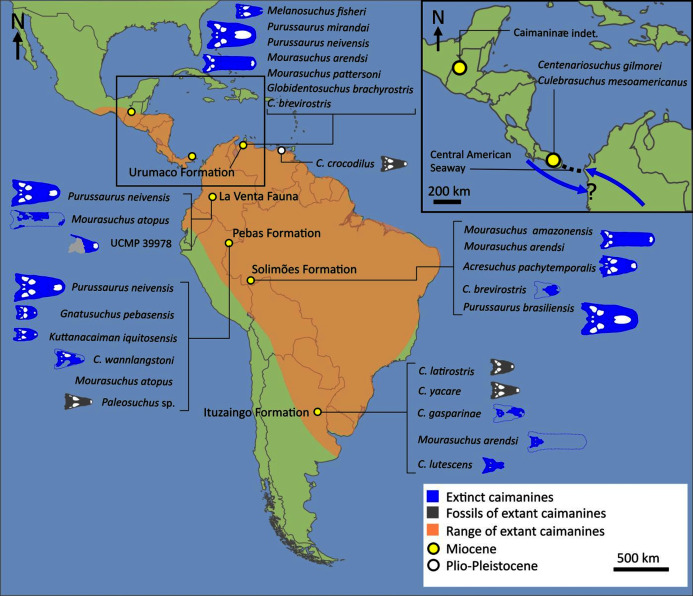
Principal South American fossil localities showing caimanine distribution from the Neogene to the Quaternary. Based on data in the Paleobiology Database (https://paleobiodb.org/). Extant caimanine range taken from https://www.iucnredlist.org/. Modern map from https://en.wikipedia.org/wiki/Wikipedia:Blankmaps.

Caimanines also appear to have diversified both in terms of numbers of species and ecomorphological disparity in South America during the middle–late Miocene (Scheyer et al., 2013; Salas-Gismondi et al., 2015; Scheyer & Delfino, 2016; Souza-Filho et al., 2018; Cidade, Fortier & Hsiou, 2019; Cidade & Rincón, 2021) (Fig. 32). This included the giant (~12.5 m long) generalist predator *Purussaurus* (Aureliano et al., 2015; Souza et al., 2021), the indiscriminate ‘gulp-feeder’ *Mourasuchus* (Cidade et al., 2017; Cidade, Riff & Hsiou, 2019), as well as the durophagous taxa, *Gnatusuchus pebasensis*, *Kuttanacaiman iquitosensis*, and *Caiman wannlangstoni* (Salas-Gismondi et al., 2015). The analyses of Solórzano et al. (2020) suggest that this diversification might have occurred in the early Miocene (see also Pan et al., 2021), with long ghost lineages indicating that the early evolutionary history of much of this diversity might not be sampled (see also Hastings et al., 2013; Hastings, Reisser & Scheyer, 2016; Solórzano et al., 2019), although many of these ghost lineages would be removed if the positions of *Bottosaurus*, *Necrosuchus*, *Protocaiman*, and *Tsoabichi* were resolved as early diverging caimanines. Most extant caimanine species also make their first appearance in the Miocene fossil record (Cidade, Fortier & Hsiou, 2019). This includes the first putative occurrences of *Paleosuchus* in the middle Miocene (Salas-Gismondi et al., 2007, 2015), as well as late Miocene specimens of *Melanosuchus* (Bona et al., 2017; Foth et al., 2018; Souza-Filho et al., 2020), *Caiman latirostris* (Bona, Riff & Gasparini, 2012; Scheyer & Delfino, 2016), and *Caiman yacare* (Bona, Riff & Gasparini, 2012).

Although absent from the USA post-Eocene, caimanines were present in Central America in the Miocene, with three species currently identified in the early Miocene of Panama (Hastings et al., 2013; Hastings, Reisser & Scheyer, 2016) (Fig. 32), and an isolated occurrence described from the late Miocene of southern Mexico (Brochu & Carbot-Chanona, 2015). These occurrences all predate the main phase of the Great American Biotic Interchange (GABI), 2.6 Ma (Woodburne, 2010), as well as the full emergence of the Isthmus of Panama, between 3.5–2.8 Ma (O’Dea et al., 2016; Jaramillo, 2018). This could be taken to support the view that these Central American caimanines were descended from unsampled North-Central American species, as appears to be the case for early Miocene Panamanian terrestrial mammals, which nearly all show clear affinities with contemporaneous North American faunas (MacFadden et al., 2014). This might explain the ancestry of at least one Panamanian species (*Culebrasuchus mesoamericanus*), which has been recovered as one of the earliest diverging caimanines (excluding the early North American clade) in recent studies (Hastings et al., 2013; Hastings, Reisser & Scheyer, 2016; Cidade et al., 2017; although see Salas-Gismondi et al. (2019), who recovered it as a species of *Alligator*). However, a number of species from the middle–late Miocene of northern South America (*Globidentosuchus brachyrostris*, *Gnatusuchus pebasensis*, and *Kuttanacaiman iquitosensis*) also occupy early diverging positions within Caimaninae (*e.g*. Scheyer et al., 2013; Salas-Gismondi et al., 2015; Hastings, Reisser & Scheyer, 2016, this study), and the remaining Central American Miocene caimanine occurrences appear to be most closely related to South American taxa, including specimens that are similar to *Purussaurus* (Hastings et al., 2013; Hastings, Reisser & Scheyer, 2016; Brochu & Carbot-Chanona, 2015).

As such, it seems most likely that an earlier wave of caimanine dispersal between South and Central America occurred well before the main phase of the GABI. At face value, this would imply dispersal across the Central American Seaway (Hastings et al., 2013), which might still have separated Central and South America by ~200 km in the early Miocene (Montes et al., 2012) (Fig. 32), prior to its closure ~10 Ma (Montes et al., 2015) and the emergence of a permanent landbridge 3.5–2.8 Ma (O’Dea et al., 2016; Jaramillo, 2018). However, both molecular divergence times and the fossil records of plants, turtles, snakes, and non-volant birds and mammals, all provide evidence for much earlier dispersals of non-marine species, some initiating ~20 Ma (Bacon et al., 2015; Jaramillo, 2018). Although this does not rule out the possibility that these species might have had to cross marine barriers, and rafting was likely a distinct possibility even for salt-intolerant species (O’Dea et al., 2016), it remains possible that ephemeral terrains might have facilitated these dispersals (Bacon et al., 2015).

Today, *Caiman crocodilus* is the only caimanine species present naturally in Central America, extending from Brazil to as far north as Mexico (Fig. 32); molecular divergence estimates between subspecies and populations support a recent range extension coincident with the timing of the formation of the Isthmus of Panama and the main phase of the GABI (Venegas-Anaya et al., 2008). The fossil record of *Caiman crocodilus* is currently restricted to a single occurrence from the Plio-Pleistocene of Venezuela (Fortier & Rincón, 2013; Cidade et al., 2019a), although a late Miocene specimen from Brazil has been assigned to *Caiman* aff. *crocodilus* (Silva Lacerda et al., 2020). If either this specimen and/or the late Miocene Mexican occurrence also belongs to this species, then *Caiman crocodilus* would represent an additional early dispersal of Caimaninae (Brochu & Carbot-Chanona, 2015).

As noted above, the Laurasian distribution of Alligatoroidea was restricted to the two extant species of *Alligator* (plus the lineage leading to *A*. *hailensis*) by the Pliocene. A substantial diversity decline also characterises post-Miocene Alligatoroidea in South America (Riff et al., 2010; Fortier & Rincón, 2013; Scheyer et al., 2013; Mannion et al., 2015; Moreno-Bernal, Head & Jaramillo, 2016; De Celis, Narváez & Ortega, 2020), including the extinction of the giant taxa *Purussaurus* and *Mourasuchus*, with only extant genera remaining (Cidade, Fortier & Hsiou, 2019). This extinction has been attributed to hydrographic changes and the disappearance of the proto-Amazonian mega-wetlands (Scheyer et al., 2013; Salas-Gismondi et al., 2015), driven by Andean uplift (Hoorn et al., 2010; Rohrmann et al., 2016).

#### Planocraniidae

**Figure 33:**
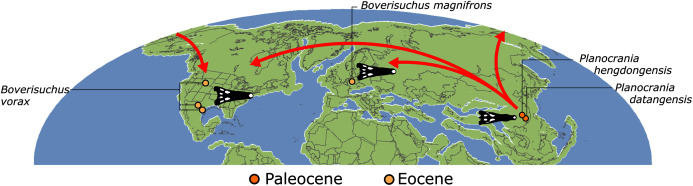
The distribution of Planocraniidae. Arrows indicate possible dispersal routes. Global palaeogeographical reconstruction at 50 Ma from Fossilworks (http://fossilworks.org/) (Alroy, 2013) based on data in the Paleobiology Database (https://paleobiodb.org/).

Although Planocraniidae is recovered in different positions to most previous studies (*e.g*. Brochu, 2012), our results still indicate that it was a short-lived Paleogene radiation of early diverging terrestrial Laurasian crocodylians. The earliest known members of Planocraniidae appear in the middle–late Paleocene of Asia, with *Planocrania datangensis* and *Planocrania hengdongensis* (Li, 1976, 1984; Brochu, 2012) (Fig. 33). Following this, planocraniids most likely dispersed to North America and Europe (Kotsakis, Delfino & Piras, 2004), where the earliest known non-Asian remains (*Boverisuchus*) unequivocally referred to the clade come from the early and middle Eocene, respectively (Kuhn, 1938; Langston, 1975; Rossmann, 2000; Brochu, 2012; Hastings & Hellmund, 2015). Regardless of the contrasting positions recovered for Planocraniidae within Crocodylia, there is a ghost lineage extending at least into the latest Cretaceous. Fragmentary remains from the Paleocene of Europe and North America might be referable to Planocraniidae (Brochu, 2012), which would both shorten this gap and question a necessarily Asian origin for the clade. Planocraniidae appears to have gone extinct by the late Eocene (Brochu, 2012; Hastings & Hellmund, 2015).

#### Crocodyloidea

**Figure 34:**
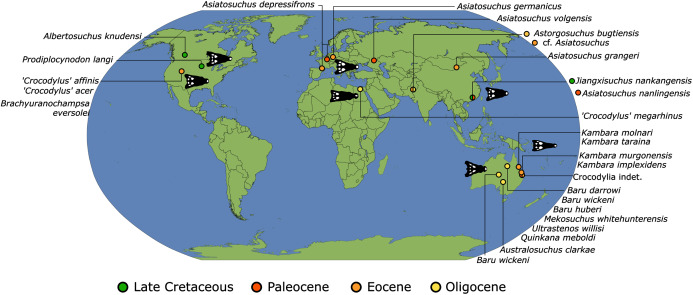
The distribution of named/distinct crocodyloids in the latest Cretaceous and Paleogene. Indeterminate remains discussed in text. Based on data in the Paleobiology Database (https://paleobiodb.org/). Modern map from https://en.wikipedia.org/wiki/Wikipedia:Blankmaps. Note that *Australosuchus* and *Baru darrowi* are known from sites dated to late Oligocene–early Miocene.

As with alligatoroids, the earliest known crocodyloids are from the latest Cretaceous of Laurasia. They first appear in the fossil record later than alligatoroids, in the Maastrichtian, represented by *Albertosuchus knudsenii* (Wu & Brinkman, 2015) and *Prodiplocynodon langi* (Mook, 1941a) in North America, and possibly by *Jiangxisuchus nankangensis* in Asia (Li, Wu & Rufolo, 2019, though see above regarding the possibility of alligatoroid affinities of this species) (Fig. 34). The late Maastrichtian Spanish species *Arenysuchus gascabadiolorum* was originally described as a crocodyloid (Puértolas-Pascual, Canudo & Cruzado-Caballero, 2011), but is now regarded as an allodaposuchid (Narváez et al., 2015). As such, the first European appearance of Crocodyloidea is not until the late Paleocene, represented by *Asiatosuchus depressifrons* (Delfino et al., 2019) (Fig. 34), with several authors suggesting that crocodyloids only dispersed to Europe after the K/Pg mass extinction (Martin et al., 2014; Puértolas-Pascual et al., 2016). *Asiatosuchus depressifrons* continued into the early Eocene of Europe (Delfino & Smith, 2009), with a second European species assigned to *Asiatosuchus* known from the middle Eocene (*A*. *germanicus*; Von Berg, 1966). Although species of *Asiatosuchus* are restricted to the early Paleogene of Eurasia (Vasse, 1992; Delfino et al., 2019; Kuzmin & Zvonok, 2021), with the earliest known remains from the early–middle Paleocene of Russia (*A*. *volgensis*; Efimov & Yarkov, 1993) and China (*A*. *nanlingensis*; Young, 1964), it is not clear that they form a clade (Angielczyk & Gingerich, 1998; Jouve et al., 2008; Delfino & Smith, 2009; Wang, Sullivan & Liu, 2016; Wu, Li & Wang, 2018; Delfino et al., 2019; Groh et al., 2020). Our analyses recover *Asiatosuchus depressifrons* and the early–middle Eocene North American species ‘*Crocodylus*’ *affinis* (Marsh, 1871) as sister taxa, with *Asiatosuchus germanicus* and *Asiatosuchus nanlingensis* as distant lineages. Most authors consider *Asiatosuchus*-like taxa to have an Asian origin (*e.g*. Jouve, 2016; Wang, Sullivan & Liu, 2016), and for the European species to have dispersed from there. Pending a revision of the systematics of *Asiatosuchus*, it remains unclear which species belong to this genus, limiting our ability to determine the biogeographic history of early crocodyloids (Delfino et al., 2019). This also includes determining the affinities of a partial skeleton with similarities to *Asiatosuchus* from the middle Eocene of Pakistan (Angielczyk & Gingerich, 1998), which by then was part of Asia (Hu et al., 2016). Finally, the position of these species as some of the earliest diverging members of Crocodyloidea implies unsampled histories extending into the latest Cretaceous.

No unambiguous occurrences of crocodyloids are known from the Paleocene of North America (Fig. 34). Their presence in the Maastrichtian and Eocene of that continent (Brochu, 2000) suggests that this Paleocene dearth is most likely a sampling artefact, rather than a genuine absence: given that they were a rare component of latest Cretaceous ecosystems (Brochu, 2000), they might have remained low in abundance and diversity during the Paleocene. However, it is possible that Crocodyloidea became regionally extinct in North America at the K/Pg boundary, with their Eocene representatives (*Brachyuranochampsa eversolei*, ‘*Crocodylus*’ *acer*, and ‘*Crocodylus*’ *affinis* (Marsh, 1871; Cope, 1882; Mook, 1921; Zangerl, 1944)) descended from Eurasian immigrants (see also Planocraniidae). Regardless of their biogeographic stock, Crocodyloidea appears to have gone extinct in North America in the late Eocene, with no unambiguous occurrences until the appearance of *Crocodylus* in the late Pliocene (Miller, 1980), although the clade might have returned as early as the late Miocene (Carbot-Chanona, 2017). A similar pattern characterises Europe, with crocodyloids absent from the late Eocene until the appearance of *Crocodylus* (or close relatives) in the late Miocene (Delfino, Böhme & Rook, 2007; Delfino et al., 2021; Delfino & Rook, 2008; Delfino & Rossi, 2013). By contrast, at least one crocodyloid (*Astorgosuchus bugtiensis*) has been recognised from the Oligocene of Asia (Martin et al., 2019a).

**Figure 35:**
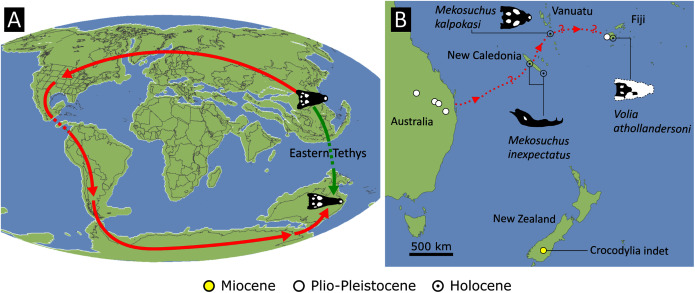
The distribution and biogeographic history of Mekosuchinae. (A) Global palaeogeographical reconstruction at 50 Ma from Fossilworks (http://fossilworks.org/) (Alroy, 2013) with arrows showing alternative dispersal routes for an Asian origin of Mekosuchinae. (B) the distribution of named mekosuchines in eastern Australia and South Pacific islands from the Neogene and Quaternary. Based on data in the Paleobiology Database (https://paleobiodb.org/). Modern map from https://en.wikipedia.org/wiki/Wikipedia:Blankmaps.

The first record of crocodylians in Australasia occurs in the early Eocene, with the mekosuchine crocodyloids *Kambara implexidens* and *Kambara murgonensis* from Australia (Willis, Molnar & Scanlon, 1993; Salisbury & Willis, 1996) (Fig. 34) (an indeterminate occurrence from either the late Paleocene or early Eocene of Australia could predate this (Willis & Molnar, 1991a; Willis, 1997b)). Mekosuchinae has been universally regarded as endemic to Australasia; however, its origin has remained enigmatic given both its labile position in relation to other members of Longirostres (*e.g*. Salisbury & Willis, 1996; Brochu & Storrs, 2012; Lee & Yates, 2018), and the near-absence of a non-marine Australasian fossil record from ~90 Ma until the clade’s first appearance. Here, *Asiatosuchus nanlingensis* is recovered as a mekosuchine within the *Kambara* clade. Combined with the recovery of *Jiangxisuchus nankangensis* as the sister taxon of Mekosuchinae + Crocodylidae in our analyses, these results provide support for an Asian origin of the clade. An early, cosmopolitan distribution of Crocodyloidea, prior to Gondwanan fragmentation, is extremely unlikely. A terrestrial dispersal route from Asia to Australasia (Fig. 35A) would require dispersal through North and South America, as well as Antarctica. Australasia and Antarctica likely retained a connection with South America until the late early Eocene (Wilf et al., 2013), which would fit well with the timing of the earliest appearance of Mekosuchinae in Australasia. Dispersal between Asia and North America in the early Paleogene appears to have been possible, and the presence of ‘basal’ crocodyloids (*e.g*. ‘*Crocodylus*’ *affinis*) on the latter continent accords with this scenario. However, this implies that there is an unsampled latest Cretaceous–early Paleogene crocodyloid fossil record in South America. The earliest unambiguous crocodyloid occurrence in South America is *Crocodylus falconensis* from the Pliocene of Venezuela (Scheyer et al., 2013). *Brasilosuchus* and *Charactosuchus*, from the middle–late Miocene of Brazil, Colombia, and Venezuela (Langston, 1965; Souza-Filho & Bocquentin, 1989; Souza-Filho, 1991; Scheyer et al., 2013), have been tentatively assigned to Crocodylidae or Tomistominae by several authors (Langston, 1965; Piras et al., 2007; Riff et al., 2010), but even if this is correct these occurrences substantially postdate the possible window for South America to Australasia dispersal. An additional species, ‘*Charactosuchus*’ *kugleri*, from the middle Eocene of Jamaica (Von Berg, 1969), could provide support that southward dispersal of crocodyloids from North America did occur around this time, but this species is generally thought to be most closely related to taxa traditionally regarded as tomistomines (*e.g*. Brochu, 2007b). Furthermore, as noted above, it is not clear that a continuous terrestrial dispersal route between North and South America existed during the Paleogene.

Although we cannot rule out the possibility that crocodyloids might have been present in the latest Cretaceous–early Paleogene of South America, especially given that paleogeographic constraints suggest caimanines might also be unsampled in the latest Cretaceous of this continent, the remaining alternative is that mekosuchines colonised Australasia from Asia *via* oceanic dispersal (Fig. 35A). Today, the Indo-Australian (=Malay/Malesia) Archipelago comprises a chain of more than 20,000 islands extending between southeast Asia and Australia (Lohman et al., 2011), and has probably facilitated the distribution of extant *Crocodylus porosus* (Webb, Manolis & Brien, 2010). However, this archipelago did not exist in the early Cenozoic, with the eastern Tethys forming a large oceanic barrier between Asia and Australasia, which only began to reduce in the middle Eocene with the northward movement of the Australian plate (Lohman et al., 2011). Although most mekosuchines are known from freshwater environments (Salisbury & Willis, 1996), saltwater tolerance was probably plesiomorphic for Longirostres, based on adaptations in all extant members of the clade, such as a keritanised buccal cavity and osmoregulatory pores on the tongue (Taplin & Grigg, 1989). Furthermore, mekosuchines reached several islands in the South Pacific, with their remains known from late Pleistocene–Holocene deposits in Fiji, New Caledonia, and Vanuatu (Balouet & Buffetaut, 1987; Mead et al., 2002; Molnar, Worthy & Willis, 2002) (Fig. 35B), and possibly from the early Miocene of New Zealand (Molnar & Pole, 1997; Molnar, Worthy & Willis, 2002). At face value, these remains would support the notion that Mekosuchinae was capable of oceanic dispersal, although the distances involved would have been dwarfed by that posed by an early Paleogene dispersal from Asia to Australia. Lower sea levels during the Pleistocene glaciations might have facilitated some of these shorter dispersals (Mead et al., 2002), but given that there are potentially long ghost lineages (>20 myr) leading to some of these Quaternary species (*e.g*. Lee & Yates, 2018, this study), it remains possible that the ancestors of these island occurrences were present in the South Pacific much earlier, with the early Miocene New Zealand occurrence providing tentative additional support for this scenario (Molnar, Worthy & Willis, 2002). Zealandia (including New Zealand and New Caledonia) started to separate from Gondwana in the latest Cretaceous, with continental connections severed in the early Paleogene, and its approximate present-day distance from Australia established during the middle Eocene (Schellart, Lister & Toy, 2006; Mortimer et al., 2017). Seamounts might have provided stepping-stones to Zealandia, but these were submerged from approximately the early Miocene, which could provide some constraints on when these crocodylians (and other terrestrial groups) dispersed (Worthy, De Pietri & Scofield, 2017). Vanuatu and Fiji lie northeast of New Caledonia; they likely formed in the late Eocene (Neall & Trewick, 2008) and probably never had a direct continental connection (Mead et al., 2002). As such, it seems difficult to reconcile the distribution of mekosuchines in the South Pacific without some degree of oceanic dispersal. Interestingly, this study is not the first time that a close affinity between *Asiatosuchus* and Mekosuchinae has been proposed. Salisbury & Willis (1996) recovered a sister taxon relationship between the Australasian clade and *Asiatosuchus germanicus*, although they regarded this as probably convergence, especially given their geographic separation. Whilst we also remain sceptical of these results, it is noteworthy that at least two occurrences of an *Asiatosuchus*-like taxon have been discovered in marine deposits (Efimov & Yarkov, 1993; Angielczyk & Gingerich, 1998), indicating the possibility that these early crocodyloids might have possessed adaptions for saltwater tolerance (see also Von Berg, 1969).

Whereas the Oligocene appears to represent a global nadir in crocodylian diversity (Mannion et al., 2015), crocodyloids are the exception (De Celis, Narváez & Ortega, 2020). This is almost entirely a result of the apparent diversification of mekosuchines in the late Oligocene–early Miocene of Australia, including the appearance of platyrostral taxa such as *Baru* (Willis, Murray & Megirian, 1990; Yates, 2017), as well as the dwarf forms *Mekosuchus whitehunterensis* (Willis, 1997a) and *Ultrastenos willisi* (Stein, Hand & Archer, 2016). Mekosuchines remained diverse throughout the Miocene, including *Baru darrowi* (Willis, Murray & Megirian, 1990), *Trilophosuchus rackhami* (Willis, 1993), and *Mekosuchus sanderi* (Willis, 2001). A number of mekosuchines are also known from the Plio-Pleistocene of Australia, including *Kalthifrons aurivellensis* (Yates & Pledge, 2017) and *Paludirex* (Ristevski et al., 2020 (including material originally assigned to ‘*Pallimnarchus*’; Willis & Molnar, 1997)). Mekosuchinae appears to have gone extinct on the Australian mainland as part of the late Pleistocene megafaunal extinction (*e.g*. Hocknull et al., 2020), with the South Pacific island species of *Mekosuchus* (*M*. *inexpectatus* and *M*. *kalpokasi*) and *Volia athollandersoni* the last remnants of this clade (Balouet & Buffetaut, 1987; Mead et al., 2002; Molnar, Worthy & Willis, 2002).

Our analyses place two Australian taxa that are usually recovered as mekosuchines on the crocodylid line instead. *Australosuchus clarkae* and *Quinkana* both first appear in the late Oligocene–early Miocene (Willis & Molnar, 1991b; Megirian, 1994; Willis, 1997a), with remains attributed to the latter taxon present until the Pleistocene (Molnar, 1982c; Willis & Mackness, 1996; Sobbe, Price & Knezour, 2013). Whereas *Quinkana* was deeply nested within Mekosuchinae in the analyses of Yates & Pledge (2017) and Lee & Yates (2018), *Australosuchus* was recovered as the earliest diverging mekosuchine, and thus its placement as the sister taxon to Crocodylidae in our study does not have much overall effect on the crocodyloid topology. Furthermore, the position of both taxa in our study is a better stratigraphic fit, reducing the length of ghost lineages. However, our results would mean that crocodylids reached Australasia at least twice, with Pliocene remains assigned to *Crocodylus* the stratigraphically oldest unequivocal occurrences of the clade on this continent (Molnar, 1982d; Yates & Pledge, 2017). The decline and eventual extinction of mekosuchines might have been driven by increasing aridification of Australia (*e.g*. Martin, 2006; Hocknull et al., 2020), exacerbated by competition from the arrival of *Crocodylus* (Willis, 1997b; Yates & Pledge, 2017).

The early Oligocene records the first appearance of Crocodyloidea in Africa, represented by the crocodyline crocodylid ‘*Crocodylus*’ *megarhinus* in Egypt (Andrews, 1905; Mook, 1927; Brochu, 2000). It is here recovered as the sister taxon of *Quinkana*, forming the earliest diverging crocodyline clade. The Egyptian species also indicates that the divergence between Crocodylinae and Osteolaeminae had occurred at least by the Oligocene, in accordance with molecular divergence estimates, which place this between 40–20 Ma (Oaks, 2011; Pan et al., 2021) (Table 12). Whereas the consensus of recent molecular phylogenies places *Mecistops* as the sister taxon to Osteolaeminae (Schmitz et al., 2003; McAliley et al., 2006; Man et al., 2011; Oaks, 2011; Shirley et al., 2014; Pan et al., 2021), our topology is consistent with previous morphological analyses (*e.g*. Brochu et al., 2010; Conrad et al., 2013; Brochu, 2020) that recover *Mecistops* as closer to *Crocodylus*, and thus part of Crocodylinae.

**Figure 36:**
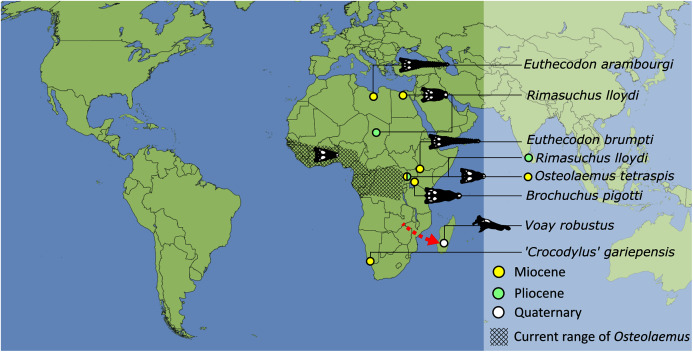
The distribution of named osteolaemines. Fossil occurrences based on data in the Paleobiology Database (https://paleobiodb.org/), present-day range of *Osteolaemus* based on Shirley et al. (2015). Modern map from https://en.wikipedia.org/wiki/Wikipedia:Blankmaps.

Osteolaemines first appear in the fossil record later than Crocodylinae, with the stratigraphically oldest occurrences known from the early Miocene of north and east Africa (Brochu, 2000, 2007a; Conrad et al., 2013; Cossette et al., 2020), represented by the earliest diverging taxa *Euthecodon arambourgi* (Ginsburg & Buffetaut, 1978) and *Brochuchus* (Tchernov & Van Couvering, 1978; Conrad et al., 2013; Cossette et al., 2020) (Fig. 36). The clade remained endemic to Africa (Brochu, 2007a), including the early–middle Miocene taxon *Rimasuchus lloydi* (Fourtau, 1920; Joleaud, 1920; Storrs, 2003; Brochu, 2020). The early Miocene Namibian species ‘*Crocodylus*’ *gariepensis* (Pickford, 2003) might represent an additional osteolaemine based on its position in the topologies of Brochu & Storrs (2012) and Cossette et al. (2020), although the analysis of Conrad et al. (2013) recovered it (and *Euthecodon*) in a clade with *Mecistops*. Remains attributed to the extant genus *Osteolaemus* first appear in the late Miocene (Aoki, 1976; Pickford, 1994).

One species usually recovered within Osteolaeminae, *Voay robustus*, is known from outside mainland Africa, with numerous remains present in late Pleistocene–Holocene deposits on Madagascar (Brochu, 2007a; Bickelmann & Klein, 2009). However, Hekkala et al. (2021) were able to extract ancient DNA from subfossil specimens of *Voay*. The inclusion of these data into phylogenetic analyses resulted in the recovery of *Voay* as the sister taxon to *Crocodylus*, *i.e*. within Crocodylinae rather than Osteolaeminae, indicating extensive homoplasy in skull shape between these two clades. Given Madagascar’s geologically long history of isolation, the *Voay* lineage must have undergone oceanic dispersal to have reached the island (Brochu, 2007a) (Fig. 36). Whereas a sister taxon relationship with *Osteolaemus* means that there is at least a 10 myr ghost lineage leading to *Voay*, the analysis of Hekkala et al. (2021) indicated that *Voay* diverged from *Crocodylus* at least 22 Ma. As such, this dispersal could potentially have occurred at any point in that unsampled window. Osteolaeminae appears to have been a relatively depauperate clade, but many Neogene specimens previously attributed to *Rimasuchus* and other taxa likely represent a richer diversity than currently recognised (*e.g*. Brochu, 2007a; Brochu & Storrs, 2012, see also Brochu & Sumrall, 2020).

**Figure 37:**
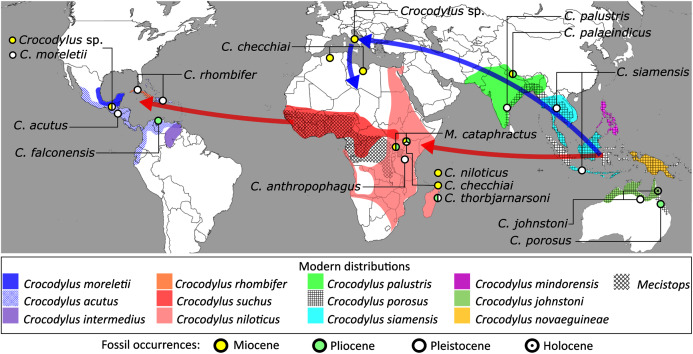
The distribution of *Crocodylus*, Neogene to Recent. Fossil occurrences based on data in the Paleobiology Database (https://paleobiodb.org/), modern ranges taken from https://www.iucnredlist.org/. Modern map taken from https://en.wikipedia.org/wiki/Wikipedia:Blankmaps.

Crocodylinae seems to have diversified in the Miocene (Densmore, 1983; Brochu, 2000; Oaks, 2011), with the first appearances of both the sub-Saharan African taxon *Mecistops* (Tchernov, 1986; Pickford, 1994; Storrs, 2003; Brochu, 2020) and species of the crown genus *Crocodylus* (Brochu, 2000; Brochu & Storrs, 2012), which corresponds well with molecular divergence estimates for the origins of these genera (Oaks, 2011; Pan et al., 2021). Late Miocene *Crocodylus* comprises *C*. *palaeindicus* in Indo-Pakistan (Lydekker, 1886; Mook, 1933), *C*. *checchiai* from north and east Africa (Maccagno, 1947; Delfino, 2008; Brochu & Storrs, 2012; Delfino et al., 2020), *C*. *niloticus* in sub-Saharan Africa (Storrs, 2003; Brochu & Storrs, 2012) and possibly in the northwest of the continent too (*e.g*. Zouhri et al., 2012), and indeterminate occurrences that extend the distribution of the genus into southern Europe (Kotsakis, Delfino & Piras, 2004; Delfino, Böhme & Rook, 2007; Delfino et al., 2021; Delfino & Rook, 2008; Delfino & Rossi, 2013) and possibly even Central America (Carbot-Chanona, 2017) (Fig. 37). By the Pliocene, *Crocodylus* had achieved a circumtropical distribution (Brochu, 2000; Meredith et al., 2011; Oaks, 2011), as exemplified by the earliest remains referable to *C*. *porosus* in Australia (Molnar, 1982b) and the extinct species *C*. *falconensis* in Venezuela (Scheyer et al., 2013) (Fig. 37).

Biogeographic implications based on the intrarelationships of Crocodylinae recovered here are tentative given their low support in our topology, and the notable difference in the placement of *Croodylus niloticus* compared to most previous studies. Determining the biogeographic origin of *Crocodylus* has proven difficult (Brochu, 2000; Delfino et al., 2020), partly because of disagreements and poor resolution in phylogenetic relationships, but also because the genus appears in the late Miocene fossil record of Africa, Asia, and Europe approximately simultaneously. The recovery of *Mecistops* as the sister taxon to the crown genus *Crocodylus* in our analysis, the ‘basal’ positions of ‘*Crocodylus*’ *megarhinus* and the Plio-Pleistocene East African species *Crocodylus thorbjarnarsoni* (Brochu & Storrs, 2012) within Crocodylinae, and the geographic distribution of the crocodyline sister taxon Osteolaeminae, all potentially support an African origin of *Crocodylus*, which is the long-held ‘traditional’ view (*e.g*. Brochu, 2000). This contrasts with recent studies based only on extant crocodylian species, which recover an Indo-Pacific origin for *Crocodylus*, either in Australasia (Oaks, 2011) or Asia (Nicolaï & Matzke, 2019). Excluding species outside of crown *Crocodylus*, our topology is generally biogeographically congruent with those latter two studies. Although we recover them as a paraphyletic array, rather than a ‘basal’ clade, the Australasian species *C*. *johnstoni* and the two Indo-Pacific island species, *C*. *novaeguineae* and *C*. *mindorensis*, are the earliest-diverging species of *Crocodylus* in our study (though note that the latter two species have no fossil record, and the earliest occurrence of *C*. *johnstoni* is from the Pleistocene (Willis & Archer, 1990)). We also recover a division between a clade of primarily Indo-Pacific species (including *C*. *porosus*) and an entirely Neotropical clade, in which the main difference with that of previous studies is that *C*. *niloticus* is recovered in the former, rather than the latter clade. Even here, this difference only places *C*. *niloticus* at the ‘base’ of one clade rather than the other. This means that our topology is still concordant with a trans-Atlantic oceanic dispersal from Africa for the Neotropical radiation of *Crocodylus* (Densmore & White, 1991; Brochu, 2000; Brochu et al., 2007; Meredith et al., 2011; Oaks, 2011; Nicolaï & Matzke, 2019; Delfino et al., 2020) (Fig. 37), prior to its first unequivocal appearance in the Pliocene of northern South America (Scheyer et al., 2013; Moreno-Bernal, Head & Jaramillo, 2016; Salas-Gismondi et al., 2019).

None of the extant Neotropical *Crocodylus* species appear in the fossil record until the Pleistocene (*e.g*. Mook, 1959; Varona, 1984; Morgan, Franz & Crombie, 1993; Cisneros, 2005; Morgan & Albury, 2013), which supports the view that this clade diversified in the Americas. Our results indicate at least two dispersal events of *Crocodylus* from the Indo-Pacific to Africa. The first of these took place by the late Miocene, giving rise to *C*. *niloticus* (*e.g*. Oaks, 2011), with a more recent, second dispersal leading to *C*. *anthropophagus*, known from the Plio-Pleistocene of East Africa (Brochu et al., 2010; Azzarà et al., 2021). The earlier dispersal has generally been presumed to have been trans-oceanic (*e.g*. Oaks, 2011), but it remains possible that dispersal from Asia to Africa occurred *via* Europe (Fig. 37), and that the southern European occurrences of *Crocodylus* do not necessarily represent African immigrants (Nicolaï & Matzke, 2019), as is usually proposed (*e.g*. Delfino, Böhme & Rook, 2007; Delfino & Rook, 2008). However, less fragmentary European specimens are required to test this hypothesis. A European route for the second dispersal event is harder to envisage given the complete absence of crocodylian fossils from Europe from the earliest Pliocene, which likely reflects a regional extinction (Kotsakis, Delfino & Piras, 2004; Delfino, Böhme & Rook, 2007; Delfino et al., 2021; Delfino & Rossi, 2013). Although the number of recognised extant species in Africa has increased in the last two decades (*e.g*. Schmitz et al., 2003; Eaton et al., 2009; Hekkala et al., 2011; Shirley et al., 2014, 2018), and it is likely that additional species will be recognised in the African fossil record too (*e.g*. Brochu & Sumrall, 2020), the phylogenetic diversity and distribution of Crocodylidae has clearly contracted over the last 10 myr. This might have been affected by increasing aridification, including the formation of the Sahara Desert (*e.g*. Zhang et al., 2014), as well as the sub-Saharan expansion of savannah environments (*e.g*. Strömberg, 2011), as suggested by Mannion et al. (2015). However, this decline was likely a gradual and more nuanced process, characterised by the waxing and waning of both species richness and the availability of suitable environments (Brochu, 2020).

*Crocodylus palaeindicus* remained present in Indo-Pakistan during the Pliocene (and was possibly present in Myanmar at this time too (Iijima et al., 2020)), and it has been suggested that the extant species in that region, *C*. *palustris*, might be its descendent (*e.g*. Lydekker, 1886; Mook, 1933). Our analysis recovers them as sister taxa, providing tentative support for an ancestor-descendent relationship. Currently, the published Quaternary record of *Crocodylus* from Indo-Pakistan comprises predominantly fragmentary materials, and much of this is probably attributed to *C*. *palustris* based on geography rather than autapomorphies (Mannion et al., 2019; Brochu & Sumrall, 2020). The most complete remains referred are still highly fragmentary and latest Pleistocene in age (Shankar & Rao, 1994); as such, better preserved material that bridges the temporal gap between the last occurrences of *C*. *palaeindicus* and earliest appearance of *C*. *palustris* are needed to further test this hypothesis, as well as to determine whether there was a greater species richness of *Crocodylus* present in Indo-Pakistan during the Plio-Pleistocene. This is also important with regards to understanding the evolutionary and biogeographic history of the Critically Endangered Indo-Pacific species *C*. *siamensis* (Bezuijen et al., 2012). Based on its Pleistocene fossil record (Delfino & De Vos, 2010; Lauprasert et al., 2019), it has been suggested that this species dispersed to Java from Indo-Pakistan, *via* Thailand (the Siva-Malayan route). However, our analysis positions *C*. *porosus* as its closest living relative, which is supported by some molecular analyses (*e.g*. Meganathan et al., 2010; Meredith et al., 2011), but not others, which recover it as the sister taxon of *C*. *palustris* (*e.g*. Li et al., 2007; Oaks, 2011; Pan et al., 2021; Hekkala et al., 2021). New molecular and morphological data might be needed to reconcile these differences.

#### Gavialoidea

Until now, the biogeographic histories of Tomistominae and Gavialoidea could be discussed independently, and when treated as such the interrelationships of the two groups were relatively stratigraphically congruent (*e.g*. Piras et al., 2007; Jouve et al., 2015; Jouve, 2016; Salas-Gismondi et al., 2016, 2019; Nicholl et al., 2020). Here, taxa usually included within Tomistominae comprise a paraphyletic array, primarily forming successive outgroups to Gavialidae (*Gavialis* + *Tomistoma*), which is similar to the topologies recovered in combined (morphology + molecular) analyses (Gold, Brochu & Norell, 2014; Lee & Yates, 2018; Iijima & Kobayashi, 2019). This also fills the ‘gavialoid gap’ that has been noted in the Eocene (Brochu, 2006). However, although we are able to robustly reconcile the placement of *Gavialis* as more closely related to *Tomistoma* than to any other extant crocodylian for the first time solely from morphology, our topology still results in a number of conspicuously long ghost lineages for many taxa, including most taxa traditionally included with Tomistominae. This partly stems from a much earlier divergence between *Gavialis* and *Tomistoma* than estimated from molecular analyses, with the most recent studies placing this split at 30–16 Ma (Oaks, 2011; Pan et al., 2021). Our topology places this split no later than ~100 Ma if *Portugalosuchus* is a gavialid, as recovered here (Table 12). Even excluding *Portugalosuchus*, the placement of two clades of ‘thoracosaurs’ within Gavialidae would still extend the latter clade back into the latest Cretaceous, ~80–70 Ma, given the Campanian age of the earliest putative specimens of *Thoracosaurus neocesariensis*, and the Maastrichtian age of unequivocal remains of this and additional species (Brochu, 2004a) (Table 12). The forced exclusion of *Portugalosuchus* and ‘thoracosaurs’ (except *Argochampsa krebsi* and *Eogavialis*) from Gavialoidea in one of our constrained analyses only goes some way to resolving these issues, with Gavialidae appearing in the Paleocene in our topology and long ghost lineages still present within the clade. Even without considering the Paleocene Moroccan taxon *Argochampsa krebsi* (Hua & Jouve, 2004; Jouve et al., 2006) or the earliest known *Eogavialis* (*E*. *africanum* from the late Eocene of Egypt; Andrews, 1906), there are still two gavialids in the topology that appear in the fossil record earlier than 30 Ma: *Paratomistoma courti* and ‘*Tomistoma*’ *cairense* from the middle Eocene of Egypt (Müller, 1927; Brochu & Gingerich, 2000). In addition to *Portugalosuchus* and ‘thoracosaurs’, this might indicate that the recovery of these taxa within Gavialidae is also a misleading phylogenetic signal.

**Figure 38:**
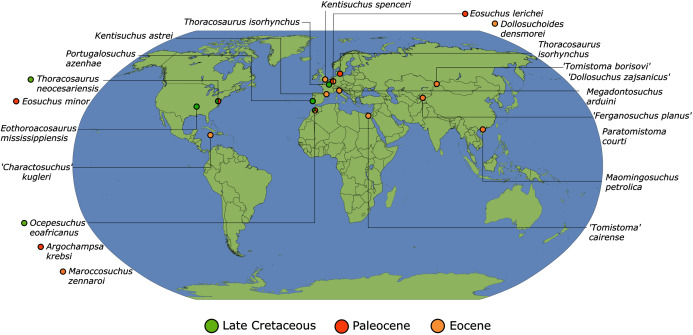
The distribution of named gavialoid species from the Cretaceous to Eocene. Fossil occurrences based on data in the Paleobiology Database (https://paleobiodb.org/). Modern ranges taken from https://www.iucnredlist.org/. Modern map taken from https://en.wikipedia.org/wiki/Wikipedia:Blankmaps.

We recover two gavialoid clades outside of Gavialidae. The earliest diverging of these consists of the early Eocene Moroccan species *Maroccosuchus zennaroi* (Jonet & Wouters, 1977; Jouve et al., 2015) as the sister taxon to an early–middle Eocene western European clade comprising *Dollosuchoides densmorei* and *Kentisuchus* (Brochu, 2007b; Jouve, 2016). Previous analyses have recovered these taxa as a paraphyletic array leading to more nested ‘tomistomines’ (Brochu, 2007b; Jouve, 2016; Iijima & Kobayashi, 2019; Nicholl et al., 2020; Ristevski et al., 2021). The recovery of this Afro-European clade supports previous suggestions that western Tethys was important in the early evolutionary history of the group (Jouve et al., 2015; Jouve, 2016) (Fig. 38). Other contemporaneous species in this region might also belong to this clade, including the middle Eocene European taxon *Megadontosuchus arduini* (Zigno, 1880; Piras et al., 2007), as well as several poorly known and neglected Eocene–Oligocene Central Asian taxa (‘*Dollosuchus zajsanicus*’, *Ferganosuchus planus*, and ‘*Tomistoma borisovi*’ Efimov, 1982, 1988, 1993), whose affinities remain uncertain (Brochu, 2007b; Piras et al., 2007; Jouve et al., 2015; Kuzmin & Zvonok, 2021) (Fig. 38). Future work should further scrutinise whether the stratigraphically problematic taxa (*i.e. Argochampsa krebsi*, *Eogavialis*, *Paratomistoma courti*, and ‘*Tomistoma*’ *cairense*) might instead be part of this early radiation, given their similar spatiotemporal distribution.

**Figure 39:**
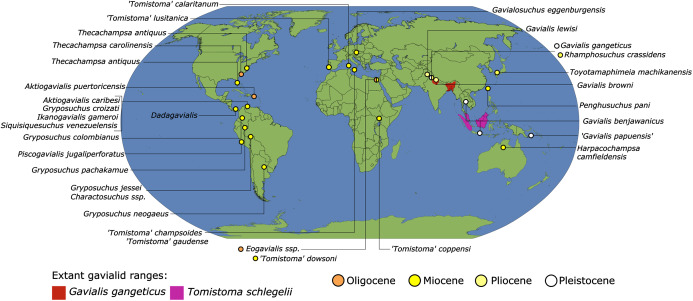
The distribution of named gavialoid species from the Oligocene to present day. Fossil occurrences based on data in the Paleobiology Database (https://paleobiodb.org/). Modern ranges taken from https://www.iucnredlist.org/. Modern map taken from https://en.wikipedia.org/wiki/Wikipedia:Blankmaps.

The second of our non-gavialid gavialoid clades places the late Eocene East Asian species *Maomingosuchus petrolica* (Yeh, 1958; Shan et al., 2017; Martin et al., 2019b) as the sister taxon to a clade of late Oligocene–Miocene western European (*Gavialosuchus eggenburgensis* and ‘*Tomistoma*’ *lusitanica*) and North American (*Thecachampsa*) taxa. A close relationship between *Maomingosuchus petrolica* and these European species was also recovered by Shan et al. (2017, see also Brochu (2007b)). There is a rich fossil record of gavialoids in the Mediterranean region of Europe during the Miocene (Piras et al., 2007) (Fig. 39), comprising *Gavialosuchus eggenburgensis* (Toula & Kail, 1885) and ‘*Tomistoma*’ *lusitanica* (Vianna & Moraes, 1945; Antunes, 1961), as well as fragmentary remains that form the basis of four taxa named in the 19th century: *Melitosaurus champsoides* (Owen, 1850), ‘*Tomistoma*’ *gaudense* (Hulke, 1871), ‘*Tomistoma*’ *calaritanum* (Capellini, 1890a, 1890b), and ‘*Tomistoma lyceense*’ (Costa, 1848). A recent revision suggests that these fragmentary remains belong to the same lineage as *Gavialosuchus eggenburgensis* and ‘*Tomistoma*’ *lusitanica*, but that they might represent one or two additional contemporaneous species (Nicholl et al., 2020). The multispecific *Thecachampsa* is known from abundant remains from the late Oligocene–Miocene of North America (Sellards, 1915; Auffenberg, 1954; Erickson & Sawyer, 1996; Myrick, 2001; Laurito & Valerio, 2008; Whiting, Steadman & Krigbaum, 2016; Weems, 2018). The recovery of *Thecachampsa* within this clade differs from the results of nearly all previous phylogenetic analyses, which have recovered *Gavialosuchus* and *Thecachampsa* as more distantly related members of Tomistominae (*e.g*. Brochu, 2007b; Jouve, 2016; Nicholl et al., 2020; Ristevski et al., 2021; though see Lee & Yates (2018) for a sister taxon relationship). Although we do not advocate their generic synonymisation, it is interesting to note that this relationship partly accords with earlier workers who referred the North American remains to *Gavialosuchus* (*e.g*. Sellards, 1915; Auffenberg, 1954; Erickson & Sawyer, 1996). The close relationship with European forms suggests biotic exchange between North America and Europe around this time, in addition to an earlier connection with Asia (Jouve, 2016). These late Miocene occurrences mark the last appearances of gavialoids in both Europe and North America.

Our topology results in only one fossil species recognised as belonging to Tomistominae **sensu* stricto* here, and this is the aforementioned *Paratomistoma courti*. Similarly, combined analyses have also recovered a depauperate Tomistominae, with ‘*Tomistoma*’ *lusitanica* the only fossil species in Lee & Yates (2018), and the clade monospecific in Iijima & Kobayashi (2019). All of these scenarios result in a long ghost lineage leading to the extant species, *Tomistoma schlegelii*, which currently lacks a fossil record (Piras et al., 2007; Mannion et al., 2019). The earliest diverging members of non-tomistomine Gavialidae are two species that we might predict should be close relatives of *Tomistoma schlegelii*, given their spatiotemporal distribution. These are *Penghusuchus pani* from the late Miocene of Taiwan (Shan et al., 2009) and *Toyotamaphimeia machikanensis* from the Pleistocene (and possibly Pliocene too) of Japan (Kobatake et al., 1965; Kobayashi et al., 2006; Iijima et al., 2018). Yet, previous analyses have also failed to recover a close relationship between these taxa and *Tomistoma schlegelii* (Shan et al., 2009; Jouve, 2016; Lee & Yates, 2018; Iijima & Kobayashi, 2019), and thus might indicate the presence of several gavialid lineages in the late Neogene–Quaternary of east and southeast Asia.

Although not included in our analysis, Lee & Yates (2018) recovered *Harpacochampsa camfieldensis*, from the middle Miocene of Australia (Megirian, Murray & Willis, 1991), as a gavialid (see also Yates & Pledge, 2017). Usually considered a mekosuchine (*e.g*. Salisbury & Willis, 1996; Willis, 1997b; Brochu, 2001; Jouve et al., 2008), or ‘basal’ crocodyloid (Ristevski et al., 2021), its slender snout is unusual for the clade, as has been previously discussed (*e.g*. Megirian, Murray & Willis, 1991). Until recently, the otherwise complete absence of Gavialoidea from the Australasian fossil record (*e.g*. Willis, 1997b), including taxa traditionally referred to Tomistominae (Piras et al., 2007), might indicate that the position of *Harpacochampsa camfieldensis* reflects long branch attraction as a result of convergence of longirostrine features (*e.g*. Brochu, 2003; Ballell et al., 2019; Groh et al., 2020). However, Ristevski et al. (2021) recently described *Gunggamarandu maunala* from the Plio-Pleistocene of Australia; these authors recovered this species in a spatiotemporally surprising position, as the sister taxon of *Dollosuchoides*, nested within the ‘basal’ assemblage of Paleogene European gavialoid taxa discussed above. Pending further study and incorporation into additional analyses, it therefore remains possible that gavialoids did reach Australasia (Yates & Pledge, 2017), and *Harpacochampsa* and *Gunggamarandu* might prove important taxa in understanding the group’s biogeographic history, especially with regards to southeast Asian gavialids.

As noted, we recover two ‘thoracosaur’ clades within Gavialidae. One of these comprises the middle Eocene north African species ‘*Tomistoma*’ *cairense* as the sister taxon of a clade consisting of *Thoracosaurus isorhynchus* + *Eosuchus*. The taxonomy of the various Eurasian species and remains referred to *Thoracosaurus* remains problematic and in need of revision, but *T*. *isorhynchus* appears to be the appropriate senior synonym for at least some of these remains (Martin & Delfino, 2010; Brignon, 2017). Regardless, at least one species of *Thoracosaurus* appears to be present from the Maastrichtian–early Paleogene in Europe (Brochu, 2004a; Martin & Delfino, 2010; Puértolas-Pascual et al., 2016). *Eosuchus* is currently known from the late Paleocene of Europe (*E*. *lerichei* Dollo, 1907; Delfino, Piras & Smith, 2005) and late Paleocene–early Eocene of North America (*E*. *minor* Marsh, 1870; Brochu, 2006). The second of the ‘thoracosaur’ clades comprises two latest Cretaceous North American species, *Thoracosaurus neocesariensis* and *Eothoracosaurus mississippiensis* (Carpenter, 1983; Brochu, 2004a), which together from a sister taxon relationship with the early Late Cretaceous European species *Portugalosuchus azenhae* (Mateus, Puértolas-Pascual & Callapez, 2019). Our topology is consistent with previous analyses in that ‘thoracosaurs’ do not appear to be a monophyletic group (*e.g*. Brochu, 2004a), and provides further evidence that oceanic dispersal between Europe and North America occurred in multiple coastal to marine eusuchian lineages.

Two north African taxa form successive outgroups to a clade consisting primarily of South American gavialoids and *Gavialis*, comprising the early Miocene species ‘*Tomistoma*’ *dowsoni* (Fourtau, 1920; Agrasar, 2004), as well as the aforementioned *Eogavialis*. As well as the late Eocene species, *Eogavialis africanum*, an additional species (*E*. *andrewsi*) appears to extend this lineage into the Miocene of Africa (Storrs, 2003). ‘*Tomistoma*’ *coppensi*, from the late Miocene–Pliocene of East Africa (Pickford, 1994), seems to represent the stratigraphically youngest gavialoid known from Africa.

**Figure 40:**
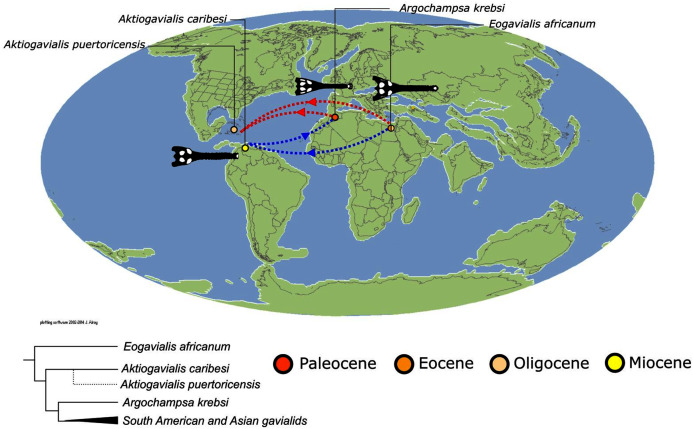
Alternative biogeographic scenarios for the origin of Neotropical gavialoids. Global palaeogeographical reconstruction at 50 Ma from Fossilworks (http://fossilworks.org/) (Alroy, 2013) based on data in the Paleobiology Database (https://paleobiodb.org/).

Since the description of *Aktiogavialis puertoricensis* from the late Oligocene of Puerto Rico, several studies have recovered this species as the stratigraphically earliest member of the Neotropical gavialoid radiation, Gryposuchinae (*e.g*. Vélez-Juarbe, Brochu & Santos, 2007; Salas-Gismondi et al., 2019). The analyses of Salas-Gismondi et al. (2016, 2019) also recovered the Paleocene north African species, *Argochampsa krebsi*, as the sister taxon to *Aktiogavialis*. Interestingly, Jouve et al. (2021) noted similarities between the Maastrichtian South American species *Dolichochampsa minima* and *Argochampsa*, which could indicate an earlier radiation, although this has yet to be tested *via* phylogenetic analysis. In our topology, these taxa are successively nested, with *Argochampsa* in a more crownward position than *Aktiogavialis*. Either scenario results in long ghost lineages and supports the hypothesis of a trans-Atlantic dispersal of gavialoids in the Paleogene (Buffetaut, 1982; Langston & Gasparini, 1997; Brochu & Rincón, 2004; Delfino, Piras & Smith, 2005; Vélez-Juarbe, Brochu & Santos, 2007; Salas-Gismondi et al., 2016; Jouve, Khalloufi & Zouhri, 2019) or latest Cretaceous (*e.g*. Jouve et al., 2021). Furthermore, our topology might indicate two independent transoceanic dispersal events, one giving rise to *Aktiogavialis* and another giving rise to all other South American and Asian gavialids (see also Salas-Gismondi et al., 2016) (Fig. 40). Alternatively, this topology could be interpreted as indicating bidirectional dispersals between Africa and the Neotropics, *i.e*. the common ancestor of *Aktiogavialis* and *Gavialis* dispersed to the Neotropics from Africa, and *Argochampsa* represents a back-dispersal (Fig. 40). By contrast to some studies that recover a diverse Gryposuchinae (*e.g*. Salas-Gismondi et al., 2016, 2019), here this clade is restricted to members of *Gryposuchus*. This is partly a result of the nested position of *Gavialis*, as well as its sister taxon relationship to a paraphyletic array of *Gryposuchus* species. Nevertheless, a similar topology has been recovered in previous studies (*e.g*. Jouve et al., 2008; Lee & Yates, 2018; Iijima & Kobayashi, 2019).

Gavialoids reached an apparent peak in diversity during the middle to late Miocene (De Celis, Narváez & Ortega, 2020), with a near global distribution. Much of this diversity comes from South America (Fig. 39), although the relatively limited early Miocene record on this continent indicates that diversity was probably also high during that interval too (*e.g*. Solórzano, Núñez-Flores & Rincón, 2018). Gavialoids were particularly speciose in the Caribbean (Salas-Gismondi et al., 2019) and northern South America during the Miocene (*e.g*. Langston, 1965; Brochu & Rincón, 2004; Riff & Aguilera, 2008; Riff et al., 2010; Scheyer et al., 2013; Salas-Gismondi et al., 2016) (comprising *Aktiogavialis caribesi*, *Dadagvialis gunai*, *Gryposuchus* ssp., *Ikanogavialis gameroi*, *Piscogavialis jugaliperforatus*, and *Siquisiquesuchus venezuelensis*), with fewer occurrences in the remainder of South America (*e.g*. Gasparini, 1968; Bona, Riff & Gasparini, 2012). The concentration of gavialoids in northern South America probably corresponds to the global pattern of latitudinal contraction of crocodylian diversity (Mannion et al., 2015), as well as the presence of extensive proto-Amazonian wetlands (Scheyer et al., 2013; Salas-Gismondi et al., 2015), with several Miocene species showing clear preferences for estuarine and freshwater environments over coastal to marine settings (*e.g*. Salas-Gismondi et al., 2016; Solórzano, Núñez-Flores & Rincón, 2018). It remains to be seen whether *Charactosuchus* might be closely related to these gavialoids, or if this represents a distinct Neotropical lineage. In particular, an understanding of the phylogenetic affinities of the middle Eocene Caribbean species, ‘*Charactosuchus*’ *kugleri* (Von Berg, 1969), will be important in this regard. Nearly all South American and Caribbean gavialoids were extinct by the end of the Miocene, with *Piscogavialis* possibly surviving later into the Pliocene (Salas-Gismondi et al., 2019). The timing of this extinction likely corresponds to the same orographic and drainage impacts that led to the demise of many other South American crocodylians at the Miocene/Pliocene boundary (*e.g*. Scheyer et al., 2013; Salas-Gismondi et al., 2015).

**Figure 41:**
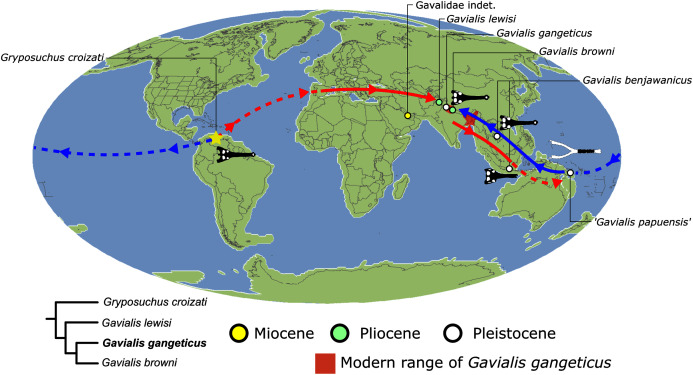
The distribution of named *Gavialis* species, illustrating alternative biogeographic routes suggested from a close relationship between South American and Asian gavialids. Global palaeogeographical reconstruction at 2 Ma from Fossilworks (http://fossilworks.org/) (Alroy, 2013) based on data in the Paleobiology Database (https://paleobiodb.org/).

*Gavialis* first appears in the Pliocene of the Indian sub-continent, represented by the extinct species *G*. *browni* and *G*. *lewisi* (Lydekker, 1886; Mook, 1932; Lull, 1944; Martin, 2019) (Fig. 39). Stratigraphically earlier referrals cannot unequivocally be referred to *Gavialis* and might represent occurrences of the poorly known species *Rhamphosuchus crassidens* (Cautley & Falconer, 1840; Head, 2001), or an additional gavialoid taxon (Martin, 2019), although late Miocene remains recently described from Myanmar could conceivably belong to the genus (Iijima et al., 2020). The earliest occurrences of the extant species, *Gavialis gangeticus*, are from the Pleistocene of India (Martin, 2019). By the Pleistocene, *Gavialis* is represented in Thailand and Indonesia by *G*. *benjawanicus* (Delfino & De Vos, 2010; Martin et al., 2012) (Fig. 41); it appears to have dispersed *via* river drainage systems, potentially reaching Indonesia during an interval of lower sea level (Martin et al., 2012, see also *Crocodylus siamensis* above). ‘*Gavialis papuensis*’ represents additional gavialid material from the Quaternary of Oceania (De Vis, 1905; Molnar, 1982a). Previously likened to the South American gavialoid *Ikanogavialis* (Molnar, 1982a; Martin et al., 2012), the preserved material is undiagnostic at species level, but it might be referable to *Gavialis* (personal observations of syntype), extending the distribution of the genus further eastwards and probably requiring the crossing of some small oceanic barriers (Fig. 41).

As noted above, *Gavialis* is often recovered as the sister taxon to Gryposuchinae, with several African taxa (*e.g. Eogavialis*) immediately outside of this clade (*e.g*. Brochu & Rincón, 2004; Delfino, Piras & Smith, 2005; Brochu, 2006; Salas-Gismondi et al., 2016, 2019). This scenario supports the hypothesis for an ‘Old World’ (*i.e*. African) origin of gavialids in both the Neotropics and Indo-Pakistan (Buffetaut, 1982; Langston & Gasparini, 1997; Brochu & Rincón, 2004; Delfino, Piras & Smith, 2005; Vélez-Juarbe, Brochu & Santos, 2007). By contrast, our results indicate a ‘New World’ origin of the *Gavialis* lineage, given its nested position in a clade of South American gavialids (see also Jouve et al., 2008). This implies a relatively recent transoceanic dispersal to Asia by the late Miocene or early Pliocene, *via* either the Atlantic or Pacific oceans (Fig. 41). Fragmentary occurrences from the late Miocene of the Arabian Peninsula provide some possible evidence to support an Atlantic, rather than Pacific, route: Rauhe et al. (1999) described a partial dentary and posterior portion of a skull, which they tentatively identified as *Ikanogavialis* (with a ‘?’) and Gavialidae, respectively, noting similarities with *Gavialis* in the latter specimen. If these specimens do belong to members of this South American-Asian clade, then they would partly fill the current spatial gap and provide tentative support for a trans-Atlantic dispersal (Fig. 41). *Gavialis gangeticus* is known exclusively from freshwater environments today, and unequivocal fossil *Gavialis* specimens are known only from fluvio-lacustrine and fluvio-deltaic environments (Delfino, Piras & Smith, 2005; Delfino & De Vos, 2010; Martin et al., 2012). However, *Gavialis gangeticus* exhibits vestigial adaptations for saltwater tolerance, including a keratinised buccal cavity and (reduced) osmoregulatory pores (Taplin & Grigg, 1989; Brochu & Rincón, 2004; Vélez-Juarbe, Brochu & Santos, 2007). As noted by previous authors (*e.g*. Taplin & Grigg, 1989; Vélez-Juarbe, Brochu & Santos, 2007), this indicates that the ancestors of *Gavialis* passed through a marine phase, which is also evident from numerous gavialoids discovered in coastal and marine deposits; however, our results indicate that this occurred very recently. Our knowledge of the saltwater tolerance of the closest South American relatives of *Gavialis* is limited, as physiological adaptations for saltwater tolerance leave no trace on the skeleton and must be inferred from the depositional environment (Vélez-Juarbe, Brochu & Santos, 2007). That several early diverging Neotropical taxa are known from coastal environments (*e.g. Piscogavialis*, *Siquisiquesuchus*, *Aktiogavialis*, *Dadagavialis*) hints towards saltwater tolerance early in the evolutionary history of neotropical gavialids. This might be expected since they are inferred to have colonised the Neotropics from Africa *via* the Atlantic Ocean (Vélez-Juarbe, Brochu & Santos, 2007; Salas-Gismondi et al., 2019). However, saltwater tolerance is more ambiguous in the taxa most closely related to *Gavialis*. *Gryposuchus croizati*, from the Urumaco Formation in Venezuela, and *Gryposuchus pachakamue*, from the Pebas Formation of Peru, are known from depositional environments with varying marine influence (Vélez-Juarbe, Brochu & Santos, 2007; Riff & Aguilera, 2008; Salas-Gismondi et al., 2016). By contrast, *Gryposuchus colombinaus* and *Gryposuchus neogaeus* are known from freshwater environments (Gasparini, 1968; Vélez-Juarbe, Brochu & Santos, 2007), but this does not necessarily mean that these species were saltwater intolerant. The late Neogene transoceanic dispersal implied by our topology indicates that gavialids closely related to *Gavialis* (*i.e. Gryposuchus*) were saltwater tolerant, and it supports the view that the current restriction of *Gavialis* to freshwater environments is a recent environmental shift (Vélez-Juarbe, Brochu & Santos, 2007).

## Conclusions

We present a new, extensively illustrated morphological dataset and a series of Parsimony analyses of the phylogenetic relationships of Crocodylia. For the first time based on a morphology-only dataset, *Gavialis gangeticus* is robustly recovered as the closest living relative of *Tomistoma schlegelii*. This result is consistent across all analyses, regardless of treatment of quantitative characters or weighting strategies, and measures of internal consistency for Gavialoidea are the highest amongst crocodylian clades. These results concur with the consensus from molecular and combined morphological and molecular phylogenetic studies undertaken over the last four decades. Constraining our analyses to recover the traditional morphological topology results in significantly less parsimonious trees. The recovery of Gavialidae (*Gavialis* + *Tomistoma*) does not appear to be an artefact of convergence in long-snouted taxa. Indeed, an evaluation of characters supporting this hypothesis indicates that it is a result of changes to character scores, character construction, and taxon sampling. These changes reveal that: (1) several species recovered in Gavialoidea lack plesiomorphic features that formerly drew them towards the ‘base’ of Crocodylia; and (2) more widespread similarities occur between taxa usually classified within Tomistominae and Gavialoidea, which are here interpreted as homology rather than homoplasy.

Despite the newly recovered topological congruence between morphological and molecular data, temporal incongruence still exists within Gavialoidea. This principally results from the inclusion of a polyphyletic array of putative gavialoids (‘thoracosaurs’ and *Portugalosuchus azenhae*) within Gavialidae. *Portugalosuchus* might represent a non-crocodylian eusuchian given its early stratigraphic age (early Late Cretaceous), and the insignificant tree length increase when constrained to lie outside of Crocodylia. By contrast, the constrained exclusion of ‘thoracosaurs’ from Crocodylia is significantly less parsimonious. Nevertheless, synapomorphies uniting ‘thoracosaurs’ with other gavialoids are mostly ambiguous and/or associated with longirostry. Indeed, newly presented anatomical data supports the exclusion of *Portugalosuchus* and at least some ‘thoracosaurs’ from Crocodylia. It is possible that these taxa belong to a currently unrecognised clade of non-crocodylian eusuchians and are drawn into Gavialoidea as a result of convergence in snout length. As such, ‘thoracosaurs’ and other putative gavialoids that result in this temporal incongruence should be targets for future re-appraisal. The interrelationships of Gavialidae result in two key biogeographical implications: (1) Neotropical gavialids are descended from African ancestors and their interrelationships imply more than one instance of trans-Atlantic oceanic dispersal; and (2) *Gavialis* is more closely related to South American than African taxa. This latter result suggests that the ancestors of *Gavialis* originated in the Neotropics and crossed a large marine barrier (most likely the Atlantic) by the late Miocene, before acquiring specialisations for inhabiting freshwater environments.

The taxonomic content of Alligatoroidea is largely consistent with most previous studies, although new interrelationships and support for internal clades are recovered. In accordance with some recent analyses, an early diverging North American clade is recovered within Caimaninae. Furthermore, our study suggests that Caimaninae originated in North America rather than South America, and that Alligatoroidea began to diversify before the K/Pg mass extinction, rather than in its aftermath. The early Paleogene South American taxon *Eocaiman* is recovered outside of Caimaninae, which would make it the only non-caimanine member of Alligatoroidea ever known from this continent. Although this result is weakly supported, if accurate, it would suggest a second dispersal of alligatoroids occurred around the K/Pg boundary from North to South America.

The recovery of *Tomistoma schlegelii* and species traditionally recovered as tomistomines within Gavialoidea, rather than Crocodyloidea, introduces several changes to the interrelationships of this latter clade. The early Paleogene European taxa *Asiatosuchus depressifrons* and *Asiatosuchus germanicus*, as well as the North American species ‘*Crocodylus*’ *affinis* are recovered as ‘basal’ crocodyloids. Similarly, the primarily Australasian clade Mekosuchinae probably resides outside of the crown group Crocodylidae. The taxonomic composition of Mekosuchinae differs to previous studies in the inclusion of *Asiatosuchus nanlingensis* from Asia, and the exclusion of the Australasian taxa *Australosuchus clarkae* and *Quinkana*. The former result, as well as the close relationship of *Jiangxisuchus* to Mekosuchinae + Crocodylidae, suggests an Asian origin for Mekosuchinae. If correct, the ancestors of Mekosuchinae most likely reached the continent *via* oceanic dispersal across Eastern Tethys in the early Paleogene.

Our study has numerous additional ramifications for the evolutionary and biogeographical history of Crocodylia, and our new dataset provides a platform for future studies to evaluate macroevolutionary patterns in this clade. Furthermore, we demonstrate that the application of extended implied weighting with higher *k*-values produces optimal phylogenetic results with regards to stratigraphic congruence and internal accuracy, at least for crocodylians. Extended implied weighting tends to override any differences in the treatment of quantitative data, *i.e*. whether continuously coded, discretised, or excluded. However, the main results of our study are consistent across analyses, regardless of treatment of quantitative characters or weighting strategies. This suggests that such methods can be applied to improve resolution in morphological datasets, without producing spurious results.

## Supplemental Information

10.7717/peerj.12094/supp-1 Supplemental Information 1 Appendix 1. Source of data for all taxa included in the phylogenetic analysis.

10.7717/peerj.12094/supp-2 Supplemental Information 2 Appendix 2. Illustrated morphological character list.

10.7717/peerj.12094/supp-3 Supplemental Information 3 Strict consensus of trees from analyses 1.3, 2.3 and 3.3.

10.7717/peerj.12094/supp-4 Supplemental Information 4 Strict consensus of trees from analyses 1.1, 1.2 and 1.3.

10.7717/peerj.12094/supp-5 Supplemental Information 5 Strict consensus tree from constraining monophyletic *Diplocynodon*.

10.7717/peerj.12094/supp-6 Supplemental Information 6 Strict consensus of 3 trees when *Eocaiman* is constrained to be recovered in Caimaninae.

10.7717/peerj.12094/supp-7 Supplemental Information 7 Strict consensus of 3 trees when *Eocaiman* is constrained to be recovered in Caimaninae, excluding the early diverging North American clade.

10.7717/peerj.12094/supp-8 Supplemental Information 8 Strict consensus of 3 trees when extant jacareans (*Caiman* and *Melanosuchus*) are forced to form a monophyletic group.

10.7717/peerj.12094/supp-9 Supplemental Information 9 Strict consensus of 3 trees, when the traditional morphological topology is constrained (*i.e*. "Gavialoidea" + (Alligatoroidea + "Crocodyloidea")).

10.7717/peerj.12094/supp-10 Supplemental Information 10 Strict consensus of 3 trees, when Analysis 1.3 is repeated, but excluding characters associated with longirostry.

10.7717/peerj.12094/supp-11 Supplemental Information 11 Strict consensus of 9 trees when *Portugalosuchus azenhae* is constrained to be excluded from Crocodylia.

10.7717/peerj.12094/supp-12 Supplemental Information 12 Strict consensus of 3 trees when ’basal’ crocodyloids are constrained to be recovered as stem longirostrines.

10.7717/peerj.12094/supp-13 Supplemental Information 13 Strict consensus of 3 trees when a monophyletic Mekosuchinae is constrained to be recovered in the stem of Longirostres.

10.7717/peerj.12094/supp-14 Supplemental Information 14 Strict consensus of 3 trees when *Asiatosuchus nanlingensis* is constrained to be recovered outside of Mekosuchinae.

10.7717/peerj.12094/supp-15 Supplemental Information 15 Strict consensus of 3 trees when *Australosuchus* and *Quinkana* are constrained to be recovered in Mekosuchinae.

10.7717/peerj.12094/supp-16 Supplemental Information 16 Strict consensus of 3 trees, when *Mecistops* is constrained to be recovered in Osteolaeminae.

10.7717/peerj.12094/supp-17 Supplemental Information 17 List of characters omitted from the character list with justifications.

10.7717/peerj.12094/supp-18 Supplemental Information 18 Stratigraphic age ranges of taxa used in stratigraphic congruence analyses.

10.7717/peerj.12094/supp-19 Supplemental Information 19 Summary of the results of constrained analyses.

10.7717/peerj.12094/supp-20 Supplemental Information 20 List of gavialoid synapomorphies and the scores of these characters in ’thoracosaurs’.

10.7717/peerj.12094/supp-21 Supplemental Information 21 Strict consensus trees from all analyses.

10.7717/peerj.12094/supp-22 Supplemental Information 22 Plots and histograms of measured values for continuous characters 1–26.

10.7717/peerj.12094/supp-23 Supplemental Information 23 Raw data: TNT file with continuous and discrete morphological characters (used in Analysis 1 and 3).

10.7717/peerj.12094/supp-24 Supplemental Information 24 Raw data: TNT file with analyses with re-discretised continuous characters (used in Analysis 2).

10.7717/peerj.12094/supp-25 Supplemental Information 25 Text file with commands for use in TNT to implement constraints.

10.7717/peerj.12094/supp-26 Supplemental Information 26 Consistency index for each character from Analysis 1.3.

## INSTITUTIONAL ABBREVIATIONS

AMNH: American Museum of Natural History, New York, USA
CAMSM: Sedgwick Museum of Earth Sciences, University of Cambridge, UK
FMNH: Field Museum of Natural History, Chicago, Illinois, USA
HLMD: Hessiches Landesmuseum Darmstadt, Germany
IGM: Instituto Nacional de Investigaciones en Geociencias, Minería y Quimica, Bogotá, Colombia
IRScNB: Institut Royal des Sciences Naturelles de Belgique, Brussels, Belgium
IVPP: Institute of Vertebrate Paleontology and Paleoanthropology, Beijing, China
MACN: Museo Argentino de Ciencias Naturales, Buenos Aires, Argentina
MCZ: Museum of Comparative Zoology, Harvard University, Cambridge, Massachusetts, USA
MLP: Museo de la Plata, Buenos Aires Province, Argentina
MMS/VBN: Musée du Moulin seigneurial/Velaux-La Bastide Neuve, Bouches-du-Rhône, France
MNHN: Museum national d’Histoire naturelle, Paris, France
MPEF: Museo Paleontológico Egidio Feruglio, Trelew, Argentina
NHMUK: Natural History Museum, London, UK
NMC: Canadian Museum of Nature, Ottowa, Ontario, Canada
QM: Queensland Museum, Brisbane, Australia
SMF: Senckenberg Museum, Frankfurt, Germany
SMNK: Staatliches Museum für Naturkunde, Karlsruhe, Germany
SMNS: Staatliches Museum für Naturkunde, Stuttgart, Germany
UCMP: University of California Museum of Paleontology, Berkeley, California, USA
UCMZ: University of Cambridge Museum of Zoology, Cambridge, UK
UFAC: Universidade Federal do Estado de Acre, Rio Branco, Brazil
USNM: United States National Museum, Smithsonian Institution, Washington DC, USA
YPM: Yale Peabody Museum of Natural History, Yale University, New Haven, Connecticut, USA
ZMNH: Zhejiang Museum of Natural History, Zhejiang Province, China

## Additional Information and Declarations

### Data Availability

The following information was supplied regarding data availability: The raw data for analyses are available in the Supplemental Files.
