# Supplementary material for: Phylogenetic analysis of a new morphological dataset elucidates the evolutionary history of Crocodylia and resolves the long-standing gharial problem
Source: PeerJ. 2021 Sep 6;9:e12094. doi: 10.7717/peerj.12094 (PMC8428266; doi:10.7717/peerj.12094)
Supplement: Supplemental Information 1 [file peerj-09-12094-s001.pdf]

Table 1: Sources of data for specimens included in the phylogenetic analysis

Taxon	Specimens Examined	Literature Used
Acresuchus pachytemporalis	UFAC 2507 (holotype), UFAC 1187, UFAC 1379, UFAC 1486, UFAC 3142, UFAC 4153, UFAC 4154, UFAC 4183, UFAC 4678, UFAC 5256, UFAC 6384	Souza-Filho et al. (2019)
Acynodon iberoccitanus	–	Buscalioni et al. (1997); Martin (2007)
Agaresuchus fontisensis	–	Narváez et al. (2016)
Aktiogavialis caribesi	–	Salas-Gismondi et al. (2019)
Alligator mcgrewi	FMNH 26242 (holotype), AMNH FAM 7905, AMNH FAM 8700, AMNH 17090	Schmidt (1941)
Alligator mefferdi	AMNH 7016 (holotype)	Mook (1941)
Alligator mississippiensis	AMNH 71621, FMNH 8202, NHMUK 1873.2.21.1, NHMUK 68.2.12.6, USNM 25148, USNM 30691	–
Alligator olseni	MCZ 1887 (holotype), MCZ 1888, MCZ 1889, MCZ 4698, MCZ 4702, MCZ 1899, MCZ 4719, MCZ 4736, MCZ 4716, MCZ 4717, MCZ 4730, MCZ 4732, MCZ 4723, MCZ uncat.	White (1942)
Alligator prenasalis	AMNH 4994, FMNH 12141 (holotype of *Allognathosuchus riggsi*), YPM PU 13799, YPM PU 16273, YPM PU 14063	Loomis (1904)
Alligator sinensis	AMNH 23907, USNM 292078, USNM 67712, USNM 52558, USNM 52557, NHMUK X184	Cong et al. (1998)
Allodaposuchus precedens	MMS/VBN-12-10A, MMS/VBN-12-42 (Photographs by Jeremy Martin)	Buscalioni et al. (2001); Martin (2010); Delfino et al. (2008);Martin et al. (2016); Narváez et al. (2019)
Allognathosuchus polyodon	USNM 4112	Mook (1921a, 1961); Brochu (2004b)
Allognathosuchus wartheni	YPM PU 16989	Case (1925); Brochu (2004b)
Arambourgia gaudryi	MNHN QU17155	Kälin (1939)
Argochampsa krebsi	NHMUK R 36872	Hua and Jouve (2004); Jouve et al. (2006)
Asiatosuchus depressifrons	MNHN G 159, IRScNB IG 9912, IRScNB R 251, IRScNB R 253, IRScNB R 254	Delfino and Smith (2009)

Table 1 continued from previous page

Taxon	Specimens Examined	Literature Used
Asiatosuchus germanicus	SMF Me 1801, HLMD-Me-1352, HLMD-Me-5344, HLMD-Me-5640, HLMD-Me-5649, HLMD-Me-5652, HLMD-Me-5669, HLMD-Me-5670, HLMD-Me-6020, HLMD-Me-7499, HLMD-Me-3092	Berg (1966)
Asiatosuchus nanlingensis	IVPP V 2772, IVPP V 2773	Wang et al. (2016)
Australosuchus clarkae	QM F18151, QM F16788 (holotype), QM F18102, QM uncat. (maxilla), AMNH 12177, AMNH 12200, AMNH 23049, AMNH 23050, AMNH 23051, UCMP 71396, UCMP 100028, UCMP 88192, UCMP 6213, UCMP 100027, UCMP 154338	Willis and Molnar (1991)
Baru darrowi	–	Willis et al. (1990)
Baru huberi	QM F31060 (holotype), QM F31061, QM F31062, QM F31063, QM F31064, QM F31065, QM F31066, QM F31067, QM F31069 (paratype)	Willis (1997)
Baru wickeni	QM F16822 (holotype), QM F16823, QM F31070, QM F31071, QM F31072, QM F31073, QM F31074	Willis (1997); Yates (2017)
Bernissartia fagesii	IRScNB 1538	Buffetaut and Ford (1979); Norell and Clark (1990)
Borealosuchus acutidentatus	NMC 8544 (Photographs provided by Jordon Mallon)	Sternberg (1932); Brochu (1997)
Borealosuchus formidabilis	YPM 16242, YPM 16241, YPM 6512	Erickson (1976); Brochu (1997)
Borealosuchus sternbergii	USNM V 6533 (holotype), UCMP 284939, UCMP 173976, UCMP 154335, UCMP 137144, UCMP 134470, UCMP 133930, UCMP 133903, UCMP 130469, UCMP 130435, UCMP 130434, UCMP 129345, UCMP 129206, UCMP 126099, UCMP 119282, UCMP 77111	Gilmore (1910); Brochu (1997)
Borealosuchus threensis	–	Brochu et al. (2012)
Borealosuchus wilsoni	AMNH 6050 (holotype *Diplocynodon stuckeri*), AMNH 7637 (holotype of *Leidyosuchus wilsoni*), FMNH PR 1674	Mook (1959)
Bottosaurus harlani	–	Cossette and Brochu (2018)
Boverisuchus magnifrons	–	Rossmann (1998); Brochu (2012)
Boverisuchus vorax	YPM 249 (holotype), FMNH PR 399, UCMP 170767	Rossmann (2000); Brochu (2012)

Table 1 continued from previous page

Taxon	Specimens Examined	Literature Used
Brachychampsa montana	AMNH 5032 (holotype), UCMP 133901	Norell et al. (1994)
Brochuchus pigotti	NHMUK PV R 7729	Conrad et al. (2013)
Caiman brevirostris	UFAC 196 (holotype), UFAC 5388	Fortier et al. (2014)
Caiman crocodilus	*Caiman crocodilus apaporiensis*: FMNH 69812 (holotype), UCMP 42844, UCMP 42843, USNM 69822 (paratype); *Caiman crocodilus chiapasius*: FMNH 73694, FMNH 73701, FMNH 73712, FMNH 73721, FMNH 73724; *Caiman crocodilus fuscus*: FMNH 69835, FMNH 69837, FMNH 69844, FMNH 69848, FMNH 69849, FMNH 69859; *Caiman crocodilus* (subspecies indet): USNM 142089, USNM 217264, USNM 54094, USNM 306645, USNM 134499,	–
Caiman gasparinae	MLP-IV-15-1, MLP-IV-15-2, MLP-IV-15-4	Bona and Carabajal (2013)
Caiman latirostris	AMNH 143183, FMNH 9713, MACN-PV 1420, NHMUK 86.10.4.2, USNM 98780	Bona and Desojo (2011)
Caiman lutescens	MACN-PV 13551	Rovereto (1912)
'*Caiman* cf. *lutescens*', ('La Venta Caiman')	UCMP 39978	Langston (1965)
Caiman wannlangstoni	–	Salas-Gismondi et al. (2015)
Caiman yacare	AMNH 97300, FMNH 9141, MLP uncat.	Medem (1960)
Ceratosuchus burdoschi	FMNH P 15576 (holotype), FMNH PR 208	Schmidt (1938)
Crocodylus acutus	AMNH 7121, FMNH 69884, FMNH 59070, NHMUK 1975.997, USNM 243433, USNM 52491	Mook (1921c)
'*Crocodylus*' *affinis*	AMNH 1213, AMNH 1719, AMNH 6166, AMNH 16609, AMNH 16622, AMNH 6176, FMNH 12202, UCMP 131090, UCMP 81325, UCMP 154341, YPM 1352, YPM 265, YPM 266, YPM 246, USNM 18171 (originally referred to *Crocodylus acer*)	Mook (1921b)
Crocodylus anthropophagus	NHMUK R5893	Brochu et al. (2010)
Crocodylus intermedius	FMNH 75659, FMNH 75662, FMNH 75658, NHMUK 1851.8.25.29, NHMUK 62.10.19.1	–
Crocodylus johnstoni	QM J4280, QM J4281, QM F39230, QM J45309, QM J58446, USNM 299810, USNM 64091	–

Table 1 continued from previous page

Taxon	Specimens Examined	Literature Used
'*Crocodylus*' *megarhinus*	AMNH 5061, AMNH 5062, SMNS 2650, YPM 058532, NHMUK PV R 3327	Adams (2016)
Crocodylus mindorensis	FMNH 1135 (holotype), FMNH 19891 (paratype), USNM 228407, USNM 37435	–
Crocodylus moreletii	NHMUK 1861.4.1.4, USNM 71954, USNM 71959	–
Crocodylus niloticus	AMNH 88634, AMNH 137163, AMNH 137180, NHMUK 1934.6.3.1, NHMUK 64.1.25.4, NHMUK 1904.10.10.1, USNM 233977, USNM 63592, USNM 195448	–
Crocodylus novaeguineae	NHMUK 1886.5.20.1	–
Crocodylus palaeindicus	AMNH 1915, NHMUK 39795 ('C. sivalensis'), NHMUK 39799, NHMUK 39797, NHMUK 39801	Lydekker (1886)
Crocodylus palustris	NHMUK 97.12.31.1 (*C. palustris kimbula*), NHMUK 1845.1.8.204, NHMUK 1861.4.1.5, NHMUK 1868.4.9.11, AMNH 77632	–
Crocodylus porosus	AMNH 7115, FMNH 22026, FMNH 15226, FMNH 15229, NHMUK 1886.5.20.3, NHMUK 1864.9.11.1, NHMUK 1852.12.9.2, QM J93350, QM J5005, QM J39283, QM J47446, QM J47447, QM J47448, QM J48127, USNM 72730, USNM 61206	–
Crocodylus rhombifer	AMNH 77595, AMNH 141073, AMNH R154087 (Fidel), USNM 544377	–
Crocodylus siamensis	AMNH 118712, NHMUK 1921.4.1.171, NHMUK 1921.4.1.168, NHMUK 1921.4.1.172, NHMUK 1931.12.6.6, USNM 8893	–
Crocodylus thorbjarnarsoni	–	Brochu and Storrs (2012)
Diplocynodon darwini	SMF Me 896, SMF Me 898, SMF Me 900, SMF Me 1137, SMF Me 1289, SMF Me 2748, HLMD-Me-233, HLMD-Me-236, HLMD-Me-5317, HLMD-Me-5349, HLMD-Me-5485, HLMD-Me-5650, HLMD-Me-7492, HLMD-Me-7500, HLMD-Me-10262, HLMD-Me-10496, HLMD-Me-14600a	Ludwig (1877)

Table 1 continued from previous page

Taxon	Specimens Examined	Literature Used
Diplocynodon deponiae	SMF Me 899 (holotype), SMF Me 1114a, SMF Me 1348a, SMF Me 2609, SMF Me 11053b, HLMD-Be-147, HLMD-Me-8080, IRScNB R 261	Frey et al. (1987); Delfino and Smith (2012)
Diplocynodon hantoniensis	Specimens too numerous to list, see Rio et al. (2020).	Rio et al. (2020)
Diplocynodon muelleri	–	Piras and Buscalioni (2006)
Diplocynodon ratelii	MNHN SG13736, MNHN SG 539, MNHN SG 557, MNHN uncat. (exposing braincase), MNHN G 660, MNHN SG 623, MNHN SG 624, MNH SG 625, MNHN SG 523, MNHN SG 631, MNHN SG 632, MNHN SG 12985, MNHN SG 635, MNHN SG 678, MNHN SG 614, MNHN SG 493, MNHN SG 643, MNHN SG 648, Catalogued as '*Diplocynodon gracilis*': NHMUK 26841, NHMUK 30945, NHMUK 27751, NHMUK 26846a, NHMUK R.789, NHMUK 26842.	Aráez et al. (2017)
Diplocynodon remensis	MNHN F BR 4020	Martin et al. (2014)
Diplocynodon tormis	–	Buscalioni et al. (1992); Serrano-Martínez et al. (2019b)
Dollosuchoides densmorei		Brochu (2007b)
Eocaiman cavernensis	AMNH 3158 (holotype), AMNH 19170 (*Eocaiman* sp. consulted but not scored)	Simpson (1933); Godoy (2014)
Eocaiman palaeocenicus	MPEF PV 1933a, MACN-PV CH1914, MACN-PV CH 1915, MACN-PV CH 1916, MACN-PV CH 1627	Bona (2007)
Eogavialis africanum	NHMUK PV R 3329 (cast of holotype), NHMUK PV R3108, NHMUK PV R 3430, NHMUK PV R 3325, SMNS 11224-1, SMNS 11224-3, SMNS 11225, SMNS 11785, YPM 6263; Catalogued as '*Tomistoma*' *gavialoides* (= *E. africanum*): AMNH FR 5066, AMNH 5068, AMNH 5069, AMNH 5070, AMNH 5071, AMNH 5073, AMNH 5074 , AMNH 5075	Andrews (1906)
Eosuchus lerichei	IRScNB R 49	Delfino et al. (2005)
Eosuchus minor	USNM 321932, USNM 321933, USNM 299730, YPM 282	Brochu (2006)

Table 1 continued from previous page

Taxon	Specimens Examined	Literature Used
Eothoracosaurus mississippiensis	AMNH 3841	Brochu (2004a)
Euthecodon arambourgi	MNHN ZEL 001	Ginsburg and Buffetaut (1978)
Gavialis browni	AMNH 6279	Mook (1932)
Gavialis gangeticus	AMNH 110145, FMNH 98864, NHMUK uncat., NHMUK 1846.1.7.3, NHMUK 1935.6.4.1, NHMUK 1974.3009, NHMUK 704, NHMUK 96.7.7.4, UMZC R 5783, USNM 576261	–
Gavialis lewisi	YPM VP 3226	Lull (1944)
Gavialosuchus eggenburgensis	NHMUK PV R 797 (cast of holotype)	Toula and Kail (1885)
Glen Rose form	MCZ 4384, USNM 22039	
Globidentosuchus brachyrostris	MCZ 4336, AMU-CURS-222 (Photographs provided by Torsten Scheyer)	Scheyer and Delfino (2016); Hastings et al. (2016)
Gnatusuchus pebasensis	–	Salas-Gismondi et al. (2015)
Gryposuchus colombianus	UCMP 41136 (holotype), UCMP 40293, UCMP 40062, UCMP 38358	Langston (1965); Langston and Gasparini (1997)
Gryposuchus croizati	–	Riff and Aguilera (2008)
Gryposuchus neogaeus	MACN-PV 6218, MLP 26-413, MLP 68-18-5-1	Gasparini (1968)
Gryposuchus pachakamue	–	Salas-Gismondi et al. (2016)
Hassiacosuchus haupti	HLMD-Be-137, HLMD-Me-1435, HLMD-Me-4415, HLMD-Me-5261, HLMD-Me-6117, HLMD-Me-9119	Weitzel (1935); Berg (1966); Brochu (2004b)
Hylaeochampsa vectiana	NHMUK R177	Clark and Norell (1992)
Iharkutosuchus makadii	–	Ösi et al. (2007); Ösi (2008); Ösi and Weishampel (2009); Mateus et al. (2019, Supporting Information)
Ikanogavialis gameroi		Sill (1970)
Isisfordia duncani	QM F36211 (holotype), QM F44320 (paratype)	Salisbury et al. (2006)
Jiangxisuchus nankangensis		Li et al. (2019)
Kambara implexidens	QM F29662 (holotype), QM F29663, QM F29680, QM F30077, QM F21116, QM F21118, QM F21131, QM F29664, QM F29678	Salisbury and Willis (1996)
Kambara murgonensis	QM F11625, QM F21125, QM F21134, QM F29667, QM F29669, QM F29683, QM F29691, QM F21117, QM F29665, QM F29666, QM F31234, QM F31284	Willis et al. (1993)

Table 1 continued from previous page

Taxon	Specimens Examined	Literature Used
Kambara taraina	–	Buchanan (2009)
Kentisuchus spenceri	NHMUK 19633 (holotype), NHMUK 38974, NHMUK 37717 (‘*Crocodilus spenceri*’)	Brochu (2007b)
Kuttanacaiman iquitosensis	–	Salas-Gismondi et al. (2015)
Leidyosuchus canadensis	AMNH 5352, NHMUK R10904 (cast of RTMP 29.28.1), UCMP 131696	Wu et al. (2001)
Lohuecosuchus megadontos	–	Narváez et al. (2015); Serrano-Martínez et al. (2019a)
Maomingosuchus petrolica	IVPP V.2303, IVPP V.5015, IVPP (unnumbered skull), ZMNH M1201, ZMNH S1	Yeh (1958); Shan et al. (2017)
Maroccosuchus zennaroi	MNHN APH 18, IRScNB R408	Jouve et al. (2015)
Mecistops cataphractus	AMNH 73803, NHMUK 62.6.30.8, NHMUK 1865.4.6.1, NHMUK 1900.2.27.1, 1924.5.10.1, USNM 60578	
Mekosuchus inexpectatus	MNHN NCP 06, MNHN NCP 07	Balouet and Buffetaut (1987)
Mekosuchus sanderi	QM F31188 (holotype), QM F31186, QM F31187, QM F31166	Willis (2001)
Mekosuchus whitehunterensis	QM F31051 (holotype), QM F31052, QM F31053, QM F31054	Willis (1997)
Melanosuchus niger	AMNH 97325, FMNH 45653, NHMUK 1872.6.4.1, NHMUK 45.8.25.125	
Mourasuchus amazonensis	UFAC 1424	Langston (1966); Cidade et al. (2019b)
Mourasuchus arendsi	MLP 73-IV-15-8 (holotype of *Mourasuchus nativus*), MLP 73-IV-15-9, UFAC 1431, UFAC 1477, UFAC 1666, UFAC 2515, UFAC 3717, UFAC 4259, UFAC 5716, UFAC 5883, UFAC 1484, UFAC 1485	Gasparini (1985); Bona et al. (2013); Cidade et al. (2019c)
Mourasuchus atopus	UCMP 38012 (holotype)	Langston (1965)
Navajosuchus mooki	AMNH 6780, AMNH 5186, MCZ 8381	Simpson (1930); Mook (1942); Brochu (2004b)
Necrosuchus ionensis	AMNH 3219 (holotype)	Brochu (2011); Cidade et al. (2019a)
Osteolaemus tetraspis	AMNH 69057, AMNH 117801, FMNH 229974, NHMUK 1862.6.30.5, USNM 233978, USNM 285136	–
Paleosuchus palpebrosus	AMNH 93812, AMNH 97326	Medem (1958)
Paleosuchus trigonatus	AMNH 66391, NHMUK 1868.10.8.1, USNM 302052	Medem (1958)
Paratomistoma courti	–	Brochu and Gingerich (2000)

Table 1 continued from previous page

Taxon	Specimens Examined	Literature Used
Penghusuchus pani	–	Shan et al. (2009); Iijima and Kobayashi (2019)
Piscogavialis jugaliperforatus	SMNK 1282 PAL	Kraus (1998)
Planocrania datangensis	IVPP V5016	Brochu (2012)
Planocrania hengdongensis	IVPP V6074	Brochu (2012)
Portugalosuchus azenhae	–	Mateus et al. (2019)
Procaimanoidea utahensis	USNM 15996 (holotype)	Gilmore (1946)
Protocaiman peligrensis	MLP 80X-10-1 (holotype)	Bona et al. (2018)
Purussaurus brasiliensis	UFAC 1118, UFAC 1403, UFAC 1773, UFAC 2505, UFAC 4249, UFAC 4770, UFAC 5299, UFAC 5300, UFAC 5507, UFAC 5862, UFAC 1709, UFAC 3520, UFAC 4686	Bocquentin-Villanueva et al. (1989)
Purussaurus mirandai	–	Aguilera et al. (2006)
Purussaurus neivensis	USNM 10889 (holotype), UCMP 38108, UCMP 38932, UCMP 39657, UCMP 39704, UCMP 41101, UCMP 45719	Langston (1965)
Quinkana	*Quinkana barbarra*: QM F23220 (holotype), QM F23223, QM F23222, QM F23221; *Quinkana meboldi*: QM F31056 (holotype), QM F31059 (paratype), QM F31057 (paratype), QM F31058	Molnar (1981); Megirian (1994); Willis and Mackness (1996)
Shamosuchus djadochtaensis	–	Pol et al. (2009); Turner (2015)
Siquisiquesuchus venezuelensis	–	Brochu and Rincón (2004)
Stangerochampsa mccabei	–	Wu et al. (1996)
Thecachampsa antiquus	Catalogued as '*Gavialosuchus americanus*': AMNH 1651 , AMNH FR 5662, AMNH FR 5663, AMNH 6160 (holotype)	Weems (2018)
Thecachampsa sericodon	USNM 24938	Weems (2018)
Theriosuchus pusillus	NHMUK 48328, NHMUK 48240, NHMUK 48282, NHMUK 48228, NHMUK 48329A, NHMUK 48279, NHMUK 48218, NHMUK 48216, NHMUK 48330	Turner (2015); Tennant et al. (2016)
Thoracosaurus isorhynchus	MNHN 1902-22 (holotype), MNHN.F.MTA 61	Piveteau (1927)
Thoracosaurus neocesariensis	AMNH 3841, AMNH 2542, AMNH 2200, AMNH 2199, AMNH 2209, AMNH 2388, AMNH 1429, YPM 404	Brochu (2004a)

Table 1 continued from previous page

Taxon	Specimens Examined	Literature Used
Tomistoma cairense	SMNS 10575, SMNS 50739, SMNS 50739a, SMNS 50740, SMNS 50741, SMNS 50742, SMNS 50745	Müller (1927)
Tomistoma dowsoni	NHMUK PV R 4769	Fourtau (1920)
Tomistoma lusitanica	–	Antunes (1961)
Tomistoma schlegelii	AMNH 113078, NHMUK 1886.5.20.3, NHMUK 1894.2.21.1, NHMUK 1899.1.31.1, NHMUK 1848.10.31.19, USNM 52972, USNM 211323	–
Toyotamaphimeia machikanensis	–	Kobayashi et al. (2006); Iijima and Kobayashi (2019)
Trilophosuchus rackhami	QM F16856 (holotype), QM F31185	Willis (1993)
Tsoabichi greenriverensis	AMNH 3666, FMNH 1793	Brochu (2010)
Ultrastenos willisi	QM 31075, QM 31076, QM 31077, QM31078	Stein et al. (2016)
Voay robustus	NHMUK R 36666, NHMUK R36659, NHMUK R 36661, NHMUK R 36685, NHMUK R 36686, NHMUK R 220, NHMUK R 36653, NHMUK R 36685	Brochu (2007a)
Wannaganosuchus brachymanus	–	Erickson (1982)
Wannchampsus kirpachi	–	Adams (2014)

References

Adams, A. J. (2016). *A reassessment of the late Eocene–early Oligocene crocodylids Crocodylus megarhinus Andrews 1905 and Crocodylus articeps Andrews 1905 from the Fayúm Province, Egypt* (Thesis).

Adams, T. L. (2014). Small crocodyliform from the Lower Cretaceous (Late Aptian) of Central Texas and its systematic relationship to the evolution of Eusuchia. *Journal of Paleontology*, *88*(5), 1031–1049.

Aguilera, O. A., Riff, D., & Bocquentin-Villanueva, J. (2006). A new giant *Purussaurus* (Crocodyliformes, Alligatoridae) from the upper Miocene Urumaco formation, Venezuela. *Journal of Systematic Palaeontology*, *4*(3), 221–232.

Andrews, C. W. (1906). *A descriptive catalogue of the Tertiary Vertebrata of the Fayum, Egypt*. British Museum, London.

Antunes, M. T. (1961). *Tomistoma lusitanica, crocodilien du Miocéne du Portugal*. Revista da Faculdade de Ciencias de Lisboa.

Aráez, J. L. D., Delfino, M., Luján, À. H., Fortuny, J., Bernardini, F., & Alba, D. M. (2017). New remains of *Diplocynodon* (Crocodylia: Diplocynodontidae) from the early Miocene of the Iberian Peninsula. *Comptes Rendus Palevol*, *16*(1), 12–26.

Balouet, J.-C., & Buffetaut, E. (1987). *Mekosuchus inexpectatus*, ng, n. sp., crocodilien nouveau de l'Holocene de Nouvelle Calédonie. *Comptes rendus de l'Académie des sciences. Série 2, Mécanique, Physique, Chimie, Sciences de l'univers, Sciences de la Terre*, *304*(14), 853–856.

Berg, D. E. (1966). *Die Krokodile, insbesondere "Asiatosuchus" und aff. "Sebecus?", aus dem Eozaen von Messel bei Darmstadt/Hessen* (Vol. 52). Hessisches Landesamt für Bodenforschung.

Bocquentin-Villanueva, J., Souza-Filho, J. P., Buffetaut, E., & Negri, F. R. (1989). Nova interpretação do gênero *Purussaurus* (Crocodylia, Alligatoridae), In *Anais do xi congresso brasileiro de paleontologia. curitiba: Sociedade brasileira de paleontologia*, Sociedade Brasileira de Paleontologia, Rio de Janeiro, Brazil.

Bona, P. (2007). Una nueva especie de *Eocaiman* Simpson (Crocodylia, Alligatoridae) del Paleoceno Inferior de Patagonia. *Ameghiniana*, *44*(2), 435–445.

Bona, P., & Carabajal, A. P. (2013). *Caiman gasparinae* sp. nov., a huge alligatorid (Caimaninae) from the late Miocene of Paraná, Argentina. *Alcheringa: An Australasian Journal of Palaeontology*, *37*(4), 462–473.

Bona, P., Degrange, F. J., & Fernández, M. S. (2013). Skull anatomy of the bizarre crocodylian *Mourasuchus nativus* (Alligatoridae, Caimaninae). *The Anatomical Record*, *296*(2), 227–239.

Bona, P., & Desojo, J. B. (2011). Osteology and cranial musculature of *Caiman latirostris* (Crocodylia: Alligatoridae). *Journal of Morphology*, *272*(7), 780–795.

Bona, P., Ezcurra, M. D., Barrios, F., & Fernandez Blanco, M. V. (2018). A new Palaeocene crocodylian from southern Argentina sheds light on the early history of caimanines. *Proceedings of the Royal Society B: Biological Sciences*, *285*(1885), 20180843.

Brochu, C. A., & Rincón, A. D. (2004). A gavialoid crocodylian from the Lower Miocene of Venezuela. *Special Papers in Palaeontology*, *71*(71), 61–79.

Brochu, C. A. (1997). A review of "*Leidyosuchus*" (Crocodyliformes, Eusuchia) from the Cretaceous through Eocene of North America. *Journal of Vertebrate Paleontology*, *17*(4), 679–697.

Brochu, C. A. (2004a). A new Late Cretaceous gavialoid crocodylian from eastern North America and the phylogenetic relationships of thoracosaurs. *Journal of Vertebrate Paleontology*, *24*(3), 610–633.

Brochu, C. A. (2004b). Alligatorine phylogeny and the status of *Allognathosuchus* Mook, 1921. *Journal of Vertebrate Paleontology*, *24*(4), 857–873.

Brochu, C. A. (2006). Osteology and phylogenetic significance of *Eosuchus minor* (Marsh, 1870) new combination, a longirostrine crocodylian from the late Paleocene of North America. *Journal of Paleontology*, *80*(1), 162–186.

Brochu, C. A. (2007a). Morphology, relationships, and biogeographical significance of an extinct horned crocodile (Crocodylia, Crocodylidae) from the Quaternary of Madagascar. *Zoological Journal of the Linnean Society*, *150*(4), 835–863.

Brochu, C. A. (2007b). Systematics and taxonomy of Eocene tomistomine crocodylians from Britain and Northern Europe. *Palaeontology*, *50*(4), 917–928.

Brochu, C. A. (2010). A new alligatorid from the lower Eocene Green River Formation of Wyoming and the origin of caimans. *Journal of Vertebrate Paleontology*, *30*(4), 1109–1126.

Brochu, C. A. (2011). Phylogenetic relationships of *Necrosuchus ionensis* Simpson, 1937 and the early history of caimanines. *Zoological Journal of the Linnean Society*, *163*, S228–S256.

Brochu, C. A. (2012). Phylogenetic relationships of Palaeogene ziphodont eusuchians and the status of *Pristichampsus* Gervais, 1853. *Earth and Environmental Science Transactions of the Royal Society of Edinburgh*, *103*(3-4), 521–550.

Brochu, C. A., & Gingerich, P. D. (2000). New tomistomine crocodylian from the middle Eocene (Bartonian) of Wadi Hitan, Fayum Province, Egypt. *Contributions from the Mueum of Paleontology, The University of Michigan*, *30*(10), 251–268.

Brochu, C. A., Njau, J., Blumenschine, R. J., & Densmore, L. D. (2010). A new horned crocodile from the Plio-Pleistocene hominid sites at Olduvai Gorge, Tanzania. *PLoS One*, *5*(2), e9333.

Brochu, C. A., Parris, D. C., Grandstaff, B. S., Denton Jr, R. K., & Gallagher, W. B. (2012). A new species of *Borealosuchus* (Crocodyliformes, Eusuchia) from the Late Cretaceous–early Paleogene of New Jersey. *Journal of Vertebrate Paleontology*, *32*(1), 105–116.

Brochu, C. A., & Storrs, G. W. (2012). A giant crocodile from the Plio-Pleistocene of Kenya, the phylogenetic relationships of Neogene African crocodylines, and the antiquity of *Crocodylus* in Africa. *Journal of Vertebrate Paleontology*, *32*(3), 587–602.

Buchanan, L. A. (2009). *Kambara taraina* sp. nov. (Crocodylia, Crocodyloidea), a new Eocene mekosuchine from Queensland, Australia, and a revision of the genus. *Journal of Vertebrate Paleontology*, *29*(2), 473–486.

Buffetaut, E., & Ford, R. L. E. (1979). The crocodilian *Bernissartia* in the Wealden of the Isle of Wight. *Palaeontology*, *22*(4), 905–912.

Buscalioni, A., Ortega, F., Weishampel, D., & Jianu, C. (2001). A revision of the crocodyliform *Allodaposuchus precedens* from the Upper Cretaceous of the Hateg Basin, Romania. Its relevance in the phylogeny of Eusuchia. *Journal of Vertebrate Paleontology*, *21*(1), 74–86.

Buscalioni, A. D., Ortega, F., & Vasse, D. (1997). New crocodiles (Eusuchia: Alligatoroidea) from the Upper Cretaceous of southern Europe. *Comptes Rendus de l'Académie des Sciences. Series IIA, Earth and Planetary Science*, *325*(7), 525–530.

Buscalioni, A. D., Sanz, J. L., & Casanovas, M. L. (1992). A new species of the eusuchian crocodile *Diplocynodon* from the Eocene of Spain. *Neues Jahrbuch für Geologie und Paläontologie Abhandlungen*, *187*, 1–29.

Case, E. C. (1925). Note on a new species of the Eocene crocodilian *Allognathosuchus*, *A. wartheni*. *Contributions from the Mueum of Geology, University of Michigan*, *2*, 93–97.

Cidade, G. M., Fortier, D., & Hsiou, A. S. (2019a). Taxonomic and phylogenetic review of *Necrosuchus ionensis* (Alligatoroidea: Caimaninae) and the early evolution and radiation of caimanines. *Zoological Journal of the Linnean Society*. https://doi.org/https://doi.org/10.1093/zoolinnean/zlz051

Cidade, G. M., Riff, D., De Souza-Filho, J. P., & Hsiou, A. S. (2019b). A reassessment of the osteology of *Mourasuchus amazonensis* Price, 1964 with comments on the taxonomy of the species. *Palaeontologia Electronica*, *22*(2), 1–23.

Cidade, G. M., Souza-Filho, J. P., Hsiou, A. S., Brochu, C. A., & Riff, D. (2019c). New specimens of *Mourasuchus* (Alligatoroidea, Caimaninae) from the Miocene of Brazil and Bolivia and their taxonomic and morphological implications. *Alcheringa: An Australasian Journal of Palaeontology*, *43*(2), 261–278.

Clark, J. M., & Norell, M. (1992). The Early Cretaceous crocodylomorph *Hylaeochampsa vectiana* from the wealden of the Isle of Wight. *American Museum Novitates*, *3032*, 1–19.

Cong, L., Hou, L., Wu, X., & Hou, J. (1998). *The gross anatomy of Alligator sinensis Fauvel [in Chinese]*. China Forestry Publishing House, Beijing.

Conrad, J. L., Jenkins, K., Lehmann, T., Manthi, F. K., Peppe, D. J., Nightingale, S., Cossette, A., Dunsworth, H. M., Harcourt-Smith, W. E., & Mcnulty, K. P. (2013). New specimens of '*Crocodylus*' *pigotti* (Crocodylidae) from Rusinga Island, Kenya, and generic reallocation of the species. *Journal of Vertebrate Paleontology*, *33*(3), 629–646.

Cossette, A. P., & Brochu, C. A. (2018). A new specimen of the alligatoroid *Bottosaurus harlani* and the early history of character evolution in alligatorids. *Journal of Vertebrate Paleontology*, *38*(4), (1)–(22).

Delfino, M., Codrea, V., Folie, A., Dica, P., Godefroit, P., & Smith, T. (2008). A complete skull of *Allodaposuchus precedens* Nopcsa, 1928 (Eusuchia) and a reassessment of the morphology of the taxon based on the Romanian remains. *Journal of Vertebrate Paleontology*, *28*(1), 111–122.

Delfino, M., Piras, P., & Smith, T. (2005). Anatomy and phylogeny of the gavialoid crocodylian *Eosuchus lerichei* from the Paleogene of Europe. *Acta Palaeontologica Polonica*, *50*(3), 565–580.

Delfino, M., & Smith, T. (2009). A reassessment of the morphology and taxonomic status of '*Crocodylus*' *depressifrons* (Crocodylia, Crocodyloidea) based on the Early Eocene remains from Belgium. *Zoological Journal of the Linnean Society*, *156*(1), 140–167.

Delfino, M., & Smith, T. (2012). Reappraisal of the morphology and phylogenetic relationships of the middle Eocene alligatoroid *Diplocynodon deponiae* (Frey, Laemmert, and Riess, 1987) based on a three-dimensional specimen. *Journal of Vertebrate Paleontology*, *32*(6), 1358–1369.

Erickson, B. R. (1976). *Osteology of the early eusuchian crocodile Leidyosuchus formidabilis, sp. nov* (Vol. 2). Monographs of the Science Museum of Minnesota (Paleontology).

Erickson, B. R. (1982). *Wannaganosuchus*, a new alligator from the Paleocene of North America. *Journal of Paleontology*, 492–506.

Fortier, D. C., De Souza-Filho, J. P., Guilherme, E., Maciente, A. A., & Schultz, C. L. (2014). A new specimen of *Caiman brevirostris* (Crocodylia, Alligatoridae) from the Late Miocene of Brazil. *Journal of Vertebrate Paleontology*, *34*(4), 820–834.

Fourtau, R. (1920). *Contribution à l'étude des vertébrés miocènes de l'Egypte*. Cairo Government Press.

Frey, E., Laemmert, A., & Riess, J. (1987). *Baryphracta deponiae* n. g. n. sp. (Reptilia, Crocodylia), ein neues Krokodil aus der Grube Messel bey Darmstadt (Hessen, Bundesrepublik Deutschland). *Neues Jahrbuch für Geologie und Paläontologie, Monatschefte*, *1987*, 15–26.

Gasparini, Z. (1985). Un nuevo crocodilo (Eusuchia) Cenozoico de America de Sur. *MME-DNPM Série Geologia Paleontologia*, *2*, 51–53.

Gasparini, Z. B. (1968). Nuevos restos de *Rhamphostomopsis neogaeus* (Burm.) Rusconi 1933, (Reptilia, Crocodilia) del "Mesopotamiense" (Plioceno medio-superior) de Argentina. *Ameghiniana*, *5*(8), 299–311.

Gilmore, C. W. (1910). *Leidyosuchus sternbergii*, a new species of crocodile from the Ceratops Beds of Wyoming. *Proceedings of the United States National Museum*, *38*(1762), 485–502.

Gilmore, C. W. (1946). A new crocodilian from the Eocene of Utah. *Journal of Paleontology*, *20*, 62–67.

Ginsburg, L., & Buffetaut, é. (1978). *Euthecodon arambourgi* n. sp., et l'évolution du genre *Euthecodon*, Crocodilien du Néogène d'Afrique. *Géologie méditerranéenne*, *5*(2), 291–301.

Godoy, P. L. (2014). *Osteologia e filogenia de dois Crocodyliformes fósseis: Aplestosuchus sordidus do Cretáceo do Brasil e Eocaiman cavernensis do Eoceno da Argentina* (Thesis).

Hastings, A. K., Reisser, M., & Scheyer, T. M. (2016). Character evolution and the origin of Caimaninae (Crocodylia) in the New World Tropics: new evidence from the Miocene of Panama and Venezuela. *Journal of Paleontology*, *90*(2), 317–332.

Hua, S., & Jouve, S. (2004). A primitive marine gavialoid from the Paleocene of Morocco. *Journal of Vertebrate Paleontology*, *24*(2), 341–350.

Iijima, M., & Kobayashi, Y. (2019). Mosaic nature in the skeleton of East Asian crocodylians fills the morphological gap between "Tomistominae" and Gavialinae. *Cladistics*, *35*(6), 623–632.

Jouve, S., Bouya, B., Amaghzaz, M., & Meslouh, S. (2015). *Maroccosuchus zennaroi* (Crocodylia: Tomistominae) from the Eocene of Morocco: Phylogenetic and palaeobiogeographical implications of the basalmost tomistomine. *Journal of Systematic Palaeontology*, *13*(5), 421–445.

Jouve, S., Iarochene, M., Bouya, B., & Amaghzaz, M. (2006). New material of *Argochampsa krebsi* (Crocodylia: Gavialoidea) from the Lower Paleocene of the Oulad Abdoun Basin (Morocco): phylogenetic implications. *Geobios*, *39*(6), 817–832.

Kälin, J. A. (1939). Ein extrem kurzschnauziger Crocodilide aus den Phosphoriten des Quercy *Arambourgia* (nov. gen.) *gaudryi* de Stefano. *Abhandlungen der Schweizerischen paläontologischen Gesellschaft*, *62*, 1–18.

Kobayashi, Y., Tomida, Y., Kamei, T., & Eguchi, T. (2006). Anatomy of a Japanese tomistomine crocodylian, *Toyotamaphimeia machikanensis* (Kamei et Matsumoto, 1965), from the middle Pleistocene of Osaka Prefecture: the reassessment of its phylogenetic status within Crocodylia. *National Science Museum Monographs*, *35*, 1–121.

Kraus, R. (1998). The cranium of *Piscogavialis jugaliperforatus* n. gen., n. sp. (Gavialidae, Crocodylia) from the Miocene of Peru. *Paläontologische Zeitschrift*, *72*(3-4), 389–405.

Langston, W. (1965). Fossil crocodilians from Colombia and the Cenozoic history of the Crocodilia in South America. *University of California Publications in Geological Sciences*, *52*.

Langston, W. (1966). *Mourasuchus* Price, *Nettosuchus* Langston, and the family Nettosuchidae (Reptilia: Crocodilia). *Copeia*, *1966*(4), 882–885.

Langston, W., & Gasparini, Z. (1997). Crocodilians, *Gryposuchus*, and the South Americans gavials. In R. V. Kay, R. H. Madden, R. L. Cifelli, & J. J. Flynn (Eds.), *Vertebrate paleontology in the neotropics - the miocene fauna of la venta, colombia* (pp. 113–154). Washington, DC (Smithsonian Institution Press).

Li, C., Wu, X.-C., & Rufolo, S. J. (2019). A new crocodyloid (Eusuchia: Crocodylia) from the Upper Cretaceous of China. *Cretaceous Research*, *94*, 25–39.

Loomis, F. B. (1904). Two New River Reptiles from the Titanothere Beds. *American Journal of Science (ser. 4)*, *18*, 427–432.

Ludwig, R. (1877). *Fossile Crocodiliden aus der Tertiärformation des mainzer Beckens* (Vol. 3). Paleontographica Supplement.

Lull, R. S. (1944). Fossil gavials from north India. *American Journal of Science*, *242*(8), 417–430.

Lydekker, R. (1886). Indian Tertiary and Post-tertiary Vertebrata: Siwalik Crocodilia, Lacertilia, & Ophidia. *Palaeontologia Indica Ser. 10*, (3), 209–240.

Martin, J. E. (2007). New material of the Late Cretaceous globidontan *Acynodon iberoccitanus* (Crocodylia) from southern France. *Journal of Vertebrate Paleontology*, *27*(2), 362–372.

Martin, J. E. (2010). *Allodaposuchus* Nopsca, 1928 (Crocodylia, Eusuchia), from the Late Cretaceous of southern France and its relationships to Alligatoroidea. *Journal of Vertebrate Paleontology*, *30*(3), 756–767.

Martin, J. E., Delfino, M., Garcia, G., Godefroit, P., Berton, S., & Valentin, X. (2016). New specimens of *Allodaposuchus precedens* from France: intraspecific variability and the diversity of European Late Cretaceous eusuchians. *Zoological Journal of the Linnean Society*, *176*(3), 607–631.

Martin, J. E., Smith, T., de Lapparent de Broin, F., Escuillié, F., & Delfino, M. (2014). Late Palaeocene eusuchian remains from Mont de Berru, France, and the origin of the alligatoroid *Diplocynodon*. *Zoological Journal of the Linnean Society*, *172*(4), 867–891.

Mateus, O., Puértolas-Pascual, E., & Callapez, P. M. (2019). A new eusuchian crocodylomorph from the Cenomanian (Late Cretaceous) of Portugal reveals novel implications on the origin of Crocodylia. *Zoological Journal of the Linnean Society*, *186*(2), 501–528.

Medem, F. (1958). The crocodilian genus *Paleosuchus*. *Fieldiana Zoology*, *39*.

Medem, F. (1960). Notes on the Paraguay caiman, *Caiman yacare* Daudin. *Mitteilungen aus dem Museum für Naturkunde in Berlin. Zoologisches Museum und Institut für Spezielle Zoologie (Berlin)*, *36*, 129–142.

Megirian, D. (1994). A new species of *Quinkana* (Molnar) (Eusuchia: Crocodylidae) from the Miocene Camfield beds of Northern Australia. *The Beagle: Records of the Museums and Art Galleries of the Northern Territory*, *11*, 145–166.

Molnar, R. E. (1981). Pleistocene ziphodont crocodilians of Queensland. *Records of the Australian Museum*, *33*(19), 803–834.

Mook, C. C. (1921a). *Allognathosuchus*, a new genus of Eocene crocodilians. *Bulletin of the American Museum on Natural History*, *44*, 105–110.

Mook, C. C. (1921b). Description of a skull of a Bridger crocodilian. *Bulletin of the American Museum on Natural History*, *44*, 111–116.

Mook, C. C. (1921c). Notes on the postcranial skeleton in the Crocodilia. *Bulletin of American Museum of Natural History*, *44*, 67–100.

Mook, C. C. (1932). A new species of fossil gavial from the Siwalik beds. *American Museum Novitates*, *514*, 1–5.

Mook, C. C. (1941). A new crocodilian from the Lance Formation. *American Museum Novitates*, *1128*, 1–5.

Mook, C. C. (1942). A new fossil crocodilian from the Paleocene of New Mexico. *American Museum Novitates*, *1189*, 1–4.

Mook, C. C. (1959). A new species of fossil crocodile of the genus *Leidyosuchus* from the Green River beds. *American Museum Novitates*, *1933*, 1–6.

Mook, C. C. (1961). Notes on the skull characters of *Allognathosuchus polyodon*. *American Museum Novitates*, *2072*, 1–5.

Müller, L. (1927). Ergebnisse der Forschungsreisen Prof. E. Stromers in den Wüsten Ägypteus. Abhandlungen Bayerisch Akademie der Wissenschaften. *Mathematisch-Naturwissenschaftelischt abtelund*, *31*, 1–97.

Narváez, I., Brochu, C. A., Escaso, F., Pérez-García, A., & Ortega, F. (2016). New Spanish Late Cretaceous eusuchian reveals the synchronic and sympatric presence of two allodaposuchids. *Cretaceous Research*, *65*, 112–125.

Narváez, I., Brochu, C. A., De Celis, A., Codrea, V., Escaso, F., Pérez-García, A., & Ortega, F. (2019). New diagnosis for *Allodaposuchus precedens*, the type species of the European Upper Cretaceous clade Allodaposuchidae. *Zoological Journal of the Linnean Society*, *189*(2), 618–634.

Narváez, I., Brochu, C. A., Escaso, F., Pérez-García, A., & Ortega, F. (2015). New crocodyliforms from southwestern Europe and definition of a diverse clade of European Late Cretaceous basal eusuchians. *PLoS One*, *10*(11), e0140679.

Norell, M. A., & Clark, J. M. (1990). A reanalysis of *Bernissartia fagesii*, with comments on its phylogenetic position and its bearing on the origin and diagnosis of the Eusuchia. *Bulletin de l'Institut Royal des Sciences Naturelles de Belgique*, *60*, 115–128.

Norell, M., Clark, J. M., & Hutchison, J. H. (1994). The Late Cretaceous alligatoroid *Brachychampsa montana* (Crocodylia): new material and putative relationships. *American Museum Novitates*, *3116*, 1–26.

Ösi, A. (2008). Cranial osteology of *Iharkutosuchus makadii*, a Late Cretaceous basal eusuchian crocodyliform from Hungary. *Neues Jahrbuch für Geologie und Paläontologie-Abhandlungen*, *248*(3), 279–299.

Ösi, A., Clark, J. M., & Weishampel, D. B. (2007). First report on a new basal eusuchian crocodyliform with multicusped teeth from the Upper Cretaceous (Santonian) of Hungary. *Neues Jahrbuch für Geologie und Paläontologie-Abhandlungen*, *243*(2), 169–177.

Ösi, A., & Weishampel, D. B. (2009). Jaw mechanism and dental function in the Late Cretaceous basal eusuchian *Iharkutosuchus*. *Journal of Morphology*, *270*(8), 903–920.

Piras, P., & Buscalioni, A. D. (2006). *Diplocynodon muelleri* comb. nov., an Oligocene diplocynodontine alligatoroid from Catalonia (Ebro Basin, Lleida province, Spain). *Journal of Vertebrate Paleontology*, *26*(3), 608–620.

Piveteau, J. (1927). *Études sur quelques amphibiens et reptiles fossiles, II: Reptile du Montien* (Vol. 16). Annales de Paléontologie.

Pol, D., Turner, A. H., & Norell, M. A. (2009). Morphology of the Late Cretaceous crocodylomorph *Shamosuchus djadochtaensis* and a discussion of neosuchian phylogeny as related to the origin of Eusuchia. *Bulletin of the American Museum of Natural History*, *324*, 1–104.

Riff, D., & Aguilera, O. A. (2008). The world's largest gharials *Gryposuchus*: description of *G. croizati* n. sp. (Crocodylia, Gavialidae) from the Upper Miocene Urumaco Formation, Venezuela. *Paläontologische Zeitschrift*, *82*(2), 178–195.

Rio, J. P., Mannion, P. D., Tschopp, E., Martin, J. E., & Delfino, M. (2020). Reappraisal of the morphology and phylogenetic relationships of the alligatoroid crocodylian *Diplocynodon hantoniensis* from the late Eocene of the United Kingdom. *Zoological Journal of the Linnean Society*, *188*(2), 579–629.

Rossmann, T. (1998). Studien an känozoischen Krokodilen: 2. Taxonomische Revision der Familie Pristichampsidae Efimov (Crocodilia: Eusuchia). *Neues Jahrbuch für Geologie und Paläontologie Abhandlungen*, *210*(1), 85–128.

Rossmann, T. (2000). Skelettanatomische Beschreibung von *Pristichampus rollinatii* (Gray) (Crocodilia, Eusuchia) aus dem Paläogen von Europa, Nordamerika und Ostasien. *Courier Forschungsinstitut Senckenberg*, *221*, 1–107.

Rovereto, C. (1912). Los crocodilos fósiles en las capas de Paraná. *Anales de Museo Nacional de Buenos Aires*, *22*.

Salas-Gismondi, R., Flynn, J. J., Baby, P., Tejada-Lara, J. V., Claude, J., & Antoine, P.-O. (2016). A new 13 million year old gavialoid crocodylian from proto-Amazonian mega-wetlands reveals parallel evolutionary trends in skull shape linked to longirostry. *PloS one*, *11*(4), e0152453.

Salas-Gismondi, R., Flynn, J. J., Baby, P., Tejada-Lara, J. V., Wesselingh, F. P., & Antoine, P.-O. (2015). A Miocene hyperdiverse crocodylian community reveals peculiar trophic dynamics in proto-Amazonian mega-wetlands. *Proceedings of the Royal Society B: Biological Sciences*, *282*(1804), 20142490.

Salas-Gismondi, R., Moreno-Bernal, J. W., Scheyer, T. M., Sánchez-Villagra, M. R., & Jaramillo, C. (2019). New Miocene Caribbean gavialoids and patterns of longirostry in crocodylians. *Journal of Systematic Palaeontology*, *17*(12), 1049–1075.

Salisbury, S. W., & Willis, P. M. A. (1996). A new crocodylian from the early Eocene of south-eastern Queensland and a preliminary investigation of the phylogenetic relationships of crocodyloids. *Alcheringa*, *20*(3), 179–226.

Salisbury, S. W., Molnar, R. E., Frey, E., & Willis, P. M. A. (2006). The origin of modern crocodyliforms: new evidence from the Cretaceous of Australia. *Proceedings of the Royal Society B: Biological Sciences*, *273*(1600), 2439–2448.

Scheyer, T. M., & Delfino, M. (2016). The late Miocene caimanine fauna (Crocodylia: Alligatoroidea) of the Urumaco Formation, Venezuela. *Palaeontologia Electronica*, *19*(3), 1–57.

Schmidt, K. P. (1938). New crocodilians from the upper Paleocene of western Colorado. *Geological Series of the Field Museum of Natural History*, *6*, 315–321.

Schmidt, K. P. (1941). A new fossil alligator from Nebraska. *Fieldiana*, *8*, 27–32.

Serrano-Martínez, A., Knoll, F., Narváez, I., Lautenschlager, S., & Ortega, F. (2019a). Inner skull cavities of the basal eusuchian *Lohuecosuchus megadontos* (Upper Cretaceous, Spain) and neurosensorial implications. *Cretaceous Research*, *93*, 66–77.

Serrano-Martínez, A., Knoll, F., Narváez, I., & Ortega, F. (2019b). Brain and pneumatic cavities of the braincase of the basal alligatoroid *Diplocynodon tormis* (Eocene, Spain). *Journal of Vertebrate Paleontology*, *39*(1), e1572612.

Shan, H.-Y., Wu, X.-C., Cheng, Y.-N., & Sato, T. (2017). *Maomingosuchus petrolica*, a restudy of '*Tomistoma*' *petrolica* Yeh, 1958. *Palaeoworld*, *26*(4), 672–690.

Shan, H.-y., Wu, X.-c., Cheng, Y.-n., & Sato, T. (2009). A new tomistomine (Crocodylia) from the Miocene of Taiwan. *Canadian Journal of Earth Sciences*, *46*(7), 529–555.

Sill, W. D. (1970). Nota preliminar sobre un nuevo Gavial del Plioceno de Venezuela y una discusion de los gaviales Sudamericanos. *Ameghiniana*, *7*(2), 151–159.

Simpson, G. G. (1930). *Allognathosuchus mooki*, a new crocodile from the Puerco Formation. *American Museum Novitates*, *445*, 1–16.

Simpson, G. G. (1933). A new crocodilian from the Notostylops beds of Patagonia. *American Museum Novitates*, *623*, 1–9.

Souza-Filho, J. P., Souza, R. G., Hsiou, A. S., Riff, D., Guilherme, E., Negri, F. R., & Cidade, G. M. (2019). A new caimanine (Crocodylia, Alligatoroidea) species from the Solimões Formation of Brazil and the phylogeny of Caimaninae. *Journal of Vertebrate Paleontology*, *38*(5), e1528450.

Stein, M., Hand, S. J., & Archer, M. (2016). A new crocodile displaying extreme constriction of the mandible, from the late Oligocene of Riversleigh, Australia. *Journal of Vertebrate Paleontology*, *36*(5), e1179041.

Sternberg, C. M. (1932). A new fossil crocodile from Saskatchewan. *The Canadian Field-Naturalist*, *44*, 128–133.

Tennant, J. P., Mannion, P. D., & Upchurch, P. (2016). Evolutionary relationships and systematics of Atoposauridae (Crocodylomorpha: Neosuchia): implications for the rise of Eusuchia. *Zoological Journal of the Linnean Society*, *177*(4), 854–936.

Toula, F., & Kail, J. A. (1885). Über einen Krokodil-Schädel aus den Tertiärablagerungen von Eggenburg in Niederösterreich: eine paläontologische Studie. *Denkschriften der Keiserlichen Akademie der Wissenschaften, Mathematisch-Naturwissenschatflliche Klasse*, *50*.

Turner, A. H. (2015). A review of *Shamosuchus* and *Paralligator* (Crocodyliformes, Neosuchia) from the Cretaceous of Asia. *PLoS One*, *10*(2), e0118116.

Wang, Y.-y., Sullivan, C., & Liu, J. (2016). Taxonomic revision of *Eoalligator* (Crocodylia, Brevirostres) and the paleogeographic origins of the Chinese alligatoroids. *PeerJ*, *4*, e2356.

Weems, R. E. (2018). Crocodilians of the Calvert Cliffs. In S. J. Godfrey (Ed.), *The Geology and Vertebrate Paleontology of Calvert Cliffs, Maryland, USA* (pp. 213–240). Smithsonian Institution Scholarly Press.

Weitzel, K. (1935). *Hassiacosuchus haupti* ngn sp., ein durophages Krokodil aus dem Mitteleozän von Messel. *Notizblatt des Vereins für Erdkunde und der hessischen geologischen Landesantalt zu Darmstadt*, *16*.

White, T. E. (1942). A new alligator from the Miocene of Florida. *Copeia*, *1942*(1), 3–7.

Willis, P. M. A. (1997). New crocodilians from the Late Oligocene White Hunter Site, Riversleigh, northwestern Queensland. *Memoirs of the Queensland Museum*, *41*, 423–438.

Willis, P. M. A., & Mackness, B. S. (1996). *Ouinkana babarra*, a new species of ziphodont mekosuchine crocodile from the early Pliocene Bluff Downs local fauna, northern Australia with a revision of the genus. *Proceedings of the Linnean Society of New South Wales*, *116*, 143–151.

Willis, P. M. A., Murray, P. F., & Megirian, D. (1990). *Baru darrowi* gen. et sp. nov., a large broad-snouted crocodyline (Eusuchia: Crocodylidae) from mid-Tertiary freshwater limestones in northern Australia. *Memoirs of the Queensland Museum*, *29*(2), 521–540.

Willis, P. M. A., & Molnar, R. E. (1991). A new middle Tertiary crocodile from Lake Palankarinna, South Australia. *Records of the South Australian Museum*, *25*(1), 39–55.

Willis, P. M. A., Molnar, R. E., & Scanlon, J. D. (1993). An early Eocene crocodilian from Murgon, southeastern Queensland. *Kaupia*, *3*, 27–33.

Willis, P. M. (1993). *Trilophosuchus rackhami* gen. et sp. nov., a new crocodilian from the early Miocene limestones of Riversleigh, northwestern Queensland. *Journal of Vertebrate Paleontology*, *13*(1), 90–98.

Willis, P. (2001). New crocodilian material from the Miocene of Riversleigh (northwestern Queensland, Australia). *Crocodilian biology and evolution*, 64–74.

Wu, X.-C., Brinkman, D. B., & Russell, A. P. (1996). A new alligator from the Upper Cretaceaous of Canada and the relationship of early eusuchians. *Palaeontology*, *39*, 351–376.

Wu, X.-C., Russell, A. P., & Brinkman, D. B. (2001). A review of *Leidyosuchus canadensis* Lambe, 1907 (Archosauria: Crocodylia) and an assessment of cranial variation based upon new material. *Canadian Journal of Earth Sciences*, *38*(12), 1665–1687.

Yates, A. M. (2017). The biochronology and palaeobiogeography of *Baru* (Crocodylia: Mekosuchinae) based on new specimens from the Northern Territory and Queensland, Australia. *PeerJ*, *5*, e3458.

Yeh, H. K. (1958). A new crocodile from Maoming, Kwangtung. *Vertebrata PalAsiatica*, *2*(4), 237–242.
