# Supplementary material for: Phylogenetic analysis of a new morphological dataset elucidates the evolutionary history of Crocodylia and resolves the long-standing gharial problem
Source: PeerJ. 2021 Sep 6;9:e12094. doi: 10.7717/peerj.12094 (PMC8428266; doi:10.7717/peerj.12094)
Supplement: Supplemental Information 2 [file peerj-09-12094-s002.pdf]

Appendix 2: Morphological Character List

Continuous characters

Skull

1. Snout length in dorsal view, ratio of anteroposterior snout length (measured from level of anterior orbital margin, to anteriormost point of rostrum), to total skull length (measured from posteriormost level of quadrate condyle to anteriormost point of rostrum): ≤ 0.5 (0); > 0.5 (1) (after Wu and Sues, 1996 [4]; Groh et al., 2020 [16]).

   This character differs to that of Groh et al. (2020) only in the measurement of snout length, which is measured in a straight anteroposterior line rather than diagonally from the anterior orbit corner (Fig. 1A). Crocodylian taxa with the most elongated snouts are predominantly "gavialoids", e.g. *Gryposuchus neogaeus* (0.79) (MLP 26-413) and "tomistomines", e.g. *Toyotamaphimeia* (0.71) (Kobayashi et al., 2006), but also the giant caimanine *Mourasuchus amazonensis* (0.75) (Price, 1964). On the other end of the spectrum, the shortest snout lengths are observed in alligatorines, such as *Hassiacosuchus haupti* (0.42) (HLMD Be 137) and *Arambourgia gaudryi* (0.38) (MNHN QU 17155). The re-discretised state boundary follows Groh et al. (2020), but snout length is normally distributed (Shapiro-Wilk's test $= 0.98$, $p = 0.14$), with no discrete discontinuity in the data (Document S2).

2. Skull proportions, ratio of mediolateral rostrum width at the level of the anterior orbital margin, to mediolateral width across anterior margin of the cranial table: < 3 (0); ≥ 3 (1) (new character, after Jouve, 2004 [170]; Jouve et al., 2008 [170]).

   This character captures the differences in mediolateral width of the cranial table in crocodylians (Fig. 1B). At one extreme, some alligatoroids (e.g. *Mourasuchus*) have narrow cranial tables in proportion to their rostral widths. By contrast, several (mostly "gavialoid") crocodylians exhibit a cranial table that is almost equal to the antorbital rostral width. Measured values have a skewed distribution (Shapiro-Wilk's test = 0.94, $p < 0.001$). The discrete character boundaries are based on the marked discontinuity between two species of *Mourasuchus* and all other taxa in the dataset (Document S2).

3. External naris, mediolateral width to anteroposterior length ratio: ≤ 1 (0); > 1 (1) (after Brochu, 1997a [161]; Groh et al., 2020 [4]).

   Brochu (1997a [161]) originally delimited the morphology for the external naris as either being "*circular or keyhole-shaped (0) or wider than long (1)*". A keyhole-shaped naris could not be identified in any taxon in this dataset, and therefore this character was simply quantified following Groh et al. (2020). The re-discretised thresholds follow earlier studies (Brochu, 1997b; Groh et al.,

2020). Measured values are positively skewed (Shapiro-Wilk's test = 0.93, $p < 0.001$) and aside from the morphology in *Mourasuchus amazonensis* (Price, 1964, fig.1), which is the outlier in this dataset (naris width to length >2) (Document S2), there is no other obvious discontinuity in the data.

4. External nares, anterior margin thickness, ratio of distance between anterior margin of nares and anterior margin of rostrum to maximum anteroposterior length of external nares in dorsal view: $<$ 0.5 (0); $\geq$ 0.5 (1) (after Hastings et al., 2010, [2]; Groh et al., 2020 [3]).

   Variation in thickness of the anterior margin of the external naris (Fig. 1D) is rarely considered in studies of crocodylian phylogeny, yet it exhibits considerable variation between taxa (Document S2) (Groh et al., 2020). The anterior wall of the naris is thickest in a series of longirostrine crocodylians, e.g. *Argochampsa krebsi* (Hua & Jouve, 2004, fig.2) and *Tomistoma schlegelii* (NHMUK 1894.2.21.1). By contrast, the anterior wall is exceptionally thin in several alligatoroids, e.g. *Brachychampsa montana* (UCMP 133901) and *Stangerochampsa mccabei* (Wu et al., 1996, fig.A1). The rediscretised state boundary (0.5) follows Hastings et al. (2010) and Groh et al. (2020), but measured values appear to vary continuously with a positive skew (Shapiro-Wilk's test = 0.95, $p < 0.05$).

5. Rostral depth, ratio of maximum dorsoventral height of the maxilla to mediolateral width of the maxilla at the 5th maxillary alveolus: <0.5 (0); $\geq$ 0.5 (1) (new character, after Wu et al., 1997 [3]; Groh et al., 2020 [8]).

   Rostral depth is measured similarly to that Groh et al. (2020), except that the measurement of depth is made at the tallest point of the rostrum, rather than at the premaxilla-maxilla suture (Fig. 1E). One might expect an altirostral crocodylian, such as *Baru wickeni* (QM F16822), to exhibit the highest value for this character; however, since snout width is taken into consideration, higher values of rostral depth correspond with the mediolaterally narrow, tubular snouts of most longirostrine crocodylians, e.g. *Tomistoma schlegelii* (rostral depth = 0.64) and *Gavialis gangeticus* (rostral depth = 0.58). Shallow rostra are exhibited primarily in some alligatoroids, e.g. *Brachychampsa montana* (UCMP 133901, rostral depth = 0.16). A small discontinuity occurs at a cut-off value of 0.5, which was adapted from Wu et al. (1997) (Document S2); however, the data are normally distributed (Shapiro-Wilk's test = 0.98, p = 0.50), with several small discontinuities that might be considered character state boundaries.

6. Interorbital distance, ratio of minimum mediolateral width between orbits to maximum mediolateral width across anterior cranial table: $<$ 0.5 (0); $\geq$ 0.5 (1) (after Jouve, 2004 [181]; Jouve et al., 2008 [177]; Salas-Gismondi et al., 2015 [190]; Groh et al., 2020 [26]).

Characters describing variation in interorbital width have been implemented in several earlier studies, but using different points of comparison, e.g. interorbital width in relation to orbital width (Salas-Gismondi et al., 2015) or minimum rostrum width (Jouve et al., 2008). Comparisons here are made relative to cranial table width, similar to that of Groh et al. (2020) (Fig. 1F). Some of the largest interorbital distances were measured in extant *Crocodylus* species, e.g. *C. acutus* (interorbital distance = 0.53), *C. moreletti* (0.52), and *C. niloticus* (0.50). By contrast, the interorbital distance is extremely narrow in taxa such as *Bernissartia fagesii* (0.17) (IRScNB 1538) and *Borealosuchus formidabilis* (0.20) (Erickson, 1976, fig.4). There is no existing cut-off value, so this is set at 0.5, where a modest discontinuity can be seen (Document S2). Otherwise the data appear entirely continuous, although it is not normally distributed (Shapiro-Wilk's test = 0.96, $p < 0.05$).

7. Infratemporal fenestra size, ratio of maximum anteroposterior infratemporal fenestra length, to maximum anteroposterior length of the cranial table: < 0.8 (0); ≥ 0.8 (1) (adapted from Ortega et al., 2000 [74]; Groh et al., 2020 [33]).

Earlier studies characterised the morphology of the infratemporal fenestra using the ratio of its anteroposterior length to dorsoventral height (Ortega et al., 2000; Groh et al., 2019). A similar character is adapted here, but characterises the maximum anteroposterior length of the infratemporal fenestra relative to the cranial table length instead (Fig. 1G). This is based on the observation that several caimanines, e.g. *Mourasuchus* and *Purussaurus* have extremely enlarged infratemporal fenestrae. Indeed, there is a marked discontinuity between the size of the fenestra in these taxa (infratemporal fenestra size > 0.8) and all other taxa in the dataset (Document S2). These few taxa impart a positive skew in the data (Shapiro-Wilk's test = 0.91, $p < 0.001$).

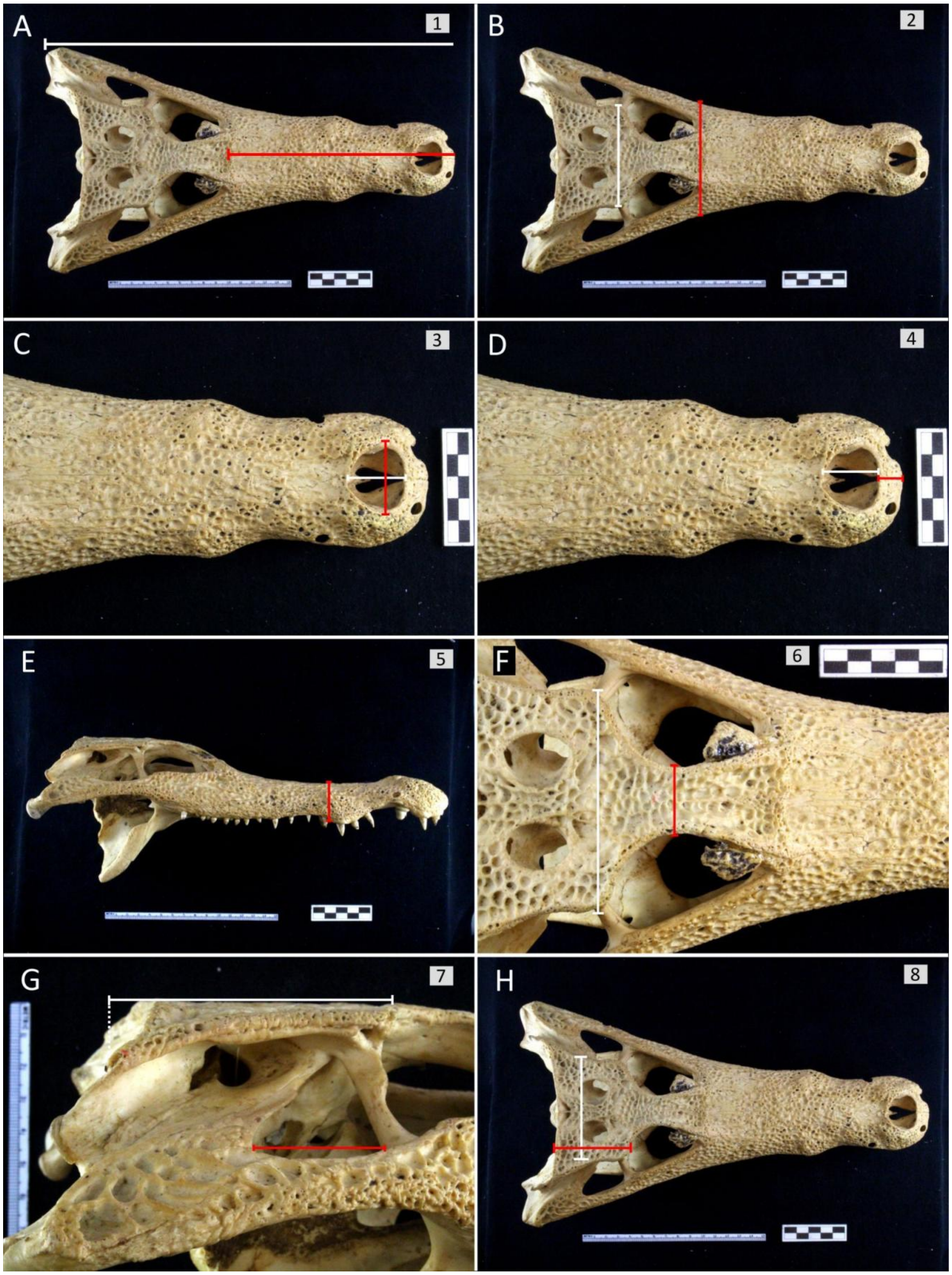

Figure 1: Continuous characters 1—8. All characters illustrated using Caiman crocodilus apaporiensis (FMNH 69812). Scale bars = cm.

8. Cranial table shape, ratio of maximum anteroposterior cranial table length (measured from the level of the frontal-postorbital suture), to maximum mediolateral width (measured at the level of the anterior table corner): < 1 (0); ≥ 1 (1) (after Wu et al., 2001b [131]; Groh et al., 2020 [41]).

   Cranial table shape was characterised similarly to that of Groh et al. (2020), except that its length is measured as the ratio of the distance between the anteriormost extent of the frontal-postorbital suture, to the posterior margin of the cranial table (Fig. 1H) instead of the maximum length including the squamosal prong (Groh et al., 2020, fig.S35). In almost all taxa in this dataset, the cranial table is wider than long, but a few taxa have approximately square-shaped cranial tables, including *Hylaeochampsa vectiana* (length to width = 1.1) *Diplocynodon hantoniensis* (NHMUK OR 30393, 0.97), and *Diplocynodon muelleri* (0.97) (Piras and Buscalioni, 2006). By contrast, the cranial table is approximately twice as wide as long in some *Borealosuchus species*, e.g. *B. formidabilis* (0.51) (Erickson, 1976, fig.4) and *B. wilsoni* (0.55) (FMNH PR 1674), and some gavialoids, e.g. *Gryposuchus neogaeu*s (0.48) (MLP 26-413) and *Ikanogavialis gameroi* (0.52) (Sill, 1970: fig.1). The rediscretised character state boundary follows earlier studies (Groh et al., 2020; Wu et al., 2001b), but the measured values appear completely continuous (Document S2) and are normally distributed (Shapiro-Wilk's test = 0.99, p = 0.26).

9. Cranial table shape, minimum angle subtended by the posterolateral cranial table margin and sagittal axis of skull: $< 10^\circ$ (0); $\geq 10^\circ$ (1) (new character, after Brochu and Storrs, 2012).

   Brochu and Storrs (2012) described a strongly trapezoidal outline of the cranial table in *Crocodylus thobjarnarsoni* resulting from anteriorly converging lateral margins of the cranial table (Fig. 2A). Measurements of taxa in the present study reveal continuous variation in this feature (Document S2). Several paralligatorids exhibit lateral margins of the cranial table that are subparallel with the sagittal plane, e.g. *Shamosuchus djadochtaensis* (Pol et al., 2009) and *Wannachampsus kirkpachi* (Adams, 2014). By contrast, some crocodyloids exhibit strongly anteriorly converging margins, e.g. *Asiatosuchus germanicus* (16°) (HLMD Me-7499) and *Voay robustus* (23°) (NHMUK R 36685). Measured values are positively skewed (Shapiro-Wilk's test = 0.93, $p < 0.001$). As there is no similar existing character, nor a discontinuity in the data, the cut-off between character states is set at 10°, which is the measured value for the outgroup, *Bernisartia fagesii*.

10. Supratemporal fenestra size, ratio of maximum anteroposterior supratemporal fenestra length to anteroposterior cranial table length (measured from the level of the frontal-postorbital suture): $<$ 0.5 (0); ≥ 0.5 (1) (after Wu et al., 2001b [67]; Groh et al., 2020 [34]).

    Supratemporal fenestra size (Fig. 2B) varies considerably in eusuchians. Measurements of fenestra size reveal a continuous range of values (Document S2), which appears normally distributed, but this is not statistically significant (Shapiro-Wilk's test = 0.96, p <0.001). Some of the largest

supratemporal fenestrae are measured in "gavialoids", e.g. *Gryposuchus neogaeus* (MLP 26-413, supratemporal fenestra size ratio = 0.66) and *Gavialis gangeticus* (0.62) (NHMUK 1974.3009), as well as alligatoroids, e.g. *Stangerochampsa mccabei* (0.68) (Wu et al., 1996). The smallest supratemporal fenestrae are measured almost exclusively in caimanines, which exhibit varying degrees of closure of the fenestrae, e.g. *Melanosuchus niger* (0.18) (NHMUK 45.8.25.125) and *Paleosuchus*, in which they are completely closed (NHMUK 1868.10.8.1). The rediscretised character state boundary follows earlier studies (cut-off = 0.5) (Wu et al., 2001b).

11. Supratemporal fenestra shape, ratio of maximum mediolateral width to maximum anteroposterior length: ≤ 1 (0); > 1 (1) (after Jouve et al., 2008 [199]; Jouve, 2016 [198]; Lee and Yates, 2018 [75]).

    Earlier studies typically characterised the shape of the supratemporal fenestrae as either circular or wider than long (Jouve et al., 2008; Lee & Yates, 2018). However, when measured (Fig. 2C), supratemporal fenestra shape varies continuously between these limits (Document S2). Most eusuchians in this dataset exhibit supratemporal fenestrae that are slightly longer than wide (mean = 0.81). The widest supratemporal fenestrae were measured in *Borealosuchus wilsoni* (FMNH PR 1674) (fenestra shape = 1.30) and several longirostrine crocodylians, e.g. *Thecachampsa antiquus* (1.24) (AMNH 5663), *Maroccosuchus zennaroi* (1.19) (IRScNB R408), and *Gavialis gangeticus* (1.20) (NHMUK 1974.3009). By contrast, highly narrow supratemporal fenestrae occur in *Diplocynodon deponiae* (0.33) (SMF Me 2609), *Trilophosuchus rackhami* (0.35) (QM F16856) and *Tsoabichi greenriverensis* (0.39) (FMNH PR 1793). The rediscretised threshold value 1 used here is adapted from Jouve et al. (2008), but there is no discontinuity here, nor at any other value. Indeed, measured values are continuous and normally distributed (Shapiro-Wilk's test = 0.99, $p = 0.64$).

12. Incisive foramen size, ratio of maximum mediolateral width to the mediolateral width of the rostrum at the premaxilla-maxilla suture: < 0.3 (0); ≥ 0.3 (1) (after Brochu, 1997a [124]; Jouve et al., 2008 [124]; Groh et al., 2020 [5]).

    The size of the incisive foramen (Fig. 2D) was previously characterised in a multistate character that combined quantitative and more subjectively defined character states, e.g. "*incisive foramen small and less than half the greatest width of the premaxillae*" or "*extremely reduced and thin*" (Jouve et al., 2008, character 124). Measurements of eusuchians in this dataset reveal that the incisive foramen is always less than half the width across the premaxillae (Document S2). Even in taxa with the largest incisive foramina, the foramen does not exceed 40% of the width across the premaxillae e.g. *Brachychampsa montana* (0.33) (UCMP 133901). As in earlier studies, the smallest incisive foramina were predominantly found in "gavialoids", e.g. *Piscogavialis jugaliper-*

foratus (SMNK 1282 PAL) (foramen size = 0.04), and *Gryposuchus neogaeus* (MLP 26-413) (0.5). A rediscretised threshold of 0.5 as implied in the original character (Brochu, 1997b), would be uninformative, so the threshold is set at 0.3, where a small discontinuity is observed. Nevertheless, the measured values are normally distributed (Shapiro-Wilk's test = 0.97, p = 0.15).

13. Suborbital fenestra shape, ratio of maximum mediolateral width to maximum anteroposterior length: ≤ 0.5 (0); > 0.5 (1) (after Buscalioni et al., 2011 [183]; Groh et al., 2020 [30]).

    Despite significant variation in the proportions of the suborbital fenestrae (Fig. 2E), this variation is not discretised in most studies of crocodylian phylogeny. The fenestra is longer than wide in all taxa in this dataset (average = 0.40), but a few crocodylians have more equidimensional fenestrae, e.g. *Eocaiman cavernensis* (fenestra shape = 0.63) (AMNH 3158) and *Alligator mcgrewi* (0.53) (AMNH FAM 7905). Taxa with highly elongated suborbital fenestrae tend to be longirostrines, e.g. *Crocodylus johnstoni* (0.28) (QM J45309) and *Piscogavialis jugaliperforatus* (SMNK 1282 PAL) (0.30), but not exclusively, e.g. *Borealosuchus sternbergii* (0.30) (UCMP 126099). The re-discretised threshold value of 0.5 follows Buscalioni et al. (2011); however, the data is almost entirely continuous and normally distributed (Shapiro-Wilk's test = 0.98, p = 0.25).

14. Choana shape, ratio of maximum mediolateral choanal width to maximum anteroposterior length: < 2 (0) ≥ 2 (1) (after Wu et al., 1997 [42]; Jouve et al., 2006 [18]; Groh et al., 2020 [46]).

    In most eusuchians in this dataset, the choana is approximately equidimensional (Fig. 2F). Measured values are positively skewed, with an average value of 1.5 (Shapiro-Wilk's test = 0.69, p = $<$0.001). The upper tail end of the distribution corresponds with a highly divergent choanal morphology, which is mediolaterally wide. This condition predominantly occurs in caimanines, such as *Mourasuchus atopus* (choanae width to length = 5.79) (UCMP 38012) and *Purussaurus neivensis* (3.6) (UCMP 39704). Earlier studies did not provide a clear threshold for discrete delimitation, but a small discontinuity in the data supports a cut-off value of 2 (Document S2).

15. Pterygoid, proportions of pterygoid wing: maximum mediolateral width to maximum anteroposterior length ratio: ≥ 3 (0); < 3 (1) (after Turner, 2015 [303]; Jouve et al., 2015 [237]).

    The width across both pterygoids (Fig. 2G) is more than twice their anteroposterior length in most eusuchians (average = 2.71). The highest values of pterygoid width to length were measured in "tomistomines", e.g. *Toyotamaphimeia* (Kobayashi et al., 2006) (pterygoid width to length = 3.82) and *Tomistoma cairense* (SMNS 50739) (3.70). By contrast, the pterygoids are more equidimensional in *Osteolaemus tetraspis* (NHMUK 1862.6.30.5) (1.68) and *Mecistops cataphractus* (NHMUK 1924.5.10.1) (1.79). The rediscretised character state boundary is adopted from earlier studies, at which point a small discontinuity can be observed; nevertheless, the data appears

normally distributed (Shapiro-Wilk's test = 0.96, $p = 0.051$).

16. Basioccipital tubera, ratio of maximum mediolateral width of basioccipital tubera to maximum mediolateral width of the occipital condyle: < 2 (0); ≥ 2 (1) (new character, based on personal observations).

    Mediolaterally wide basioccipital tubera are known in several taxa, typically "gavialoid" crocodylians, e.g. *Gavialis gangeticus* (NHMUK 1974.3009) and *Gryposuchus* (Riff & Aguilera, 2008; Salas-Gismondi et al., 2016), but variation in tubera width has not been discretised in previous studies. Measured values (Fig. 2H) are not normally distributed (Shapiro-Wilk's test = 0.90, $p < 0.001$), but positively skewed, with most eusuchians exhibiting basioccipital tubera that are > 1.5 times the width of the occipital condyle (average = 1.63). As expected, "gavialoids" exhibit the widest tubera, e.g. *Gavialis lewisi* (YPM VP 3226) (ratio = 2.5), and *Gavialis gangeticus* (1.9) (NHMUK 1974.3009). By contrast, the narrowest basioccipital tubera were measured in *Paleosuchus trigonatus* (1.3) (NHMUK 1868.10.831) and *Crocodylus porosus* (1.2) (NHMUK 1852.12.9.2). A prominent discontinuity in the measured values supports a cut-off value of 2 (Document S2).

17. Number of maxillary alveoli: < 18 (0); 18–22 (1); > 22 (2) (after Wu and Sues, 1996 [30]; Jouve, 2004 [169]; Jouve et al., 2008 [169]; Groh et al., 2020) (ORDERED).

    Most eusuchians examined in this dataset have 13–14 maxillary alveoli, including all extant alligatorids and crocodylids; however, there is continuous variation in alveolar counts above and below this value (Document S2). Longirostrine crocodylians exhibit the most alveoli, e.g. *Ikanogavialis gameroi* (30 alveoli) (Sill, 1970) and *Piscogavialis jugaliperforatus* (28 alveoli) (SMNK 1282 PAL). By contrast, *Gnatusuchus pebasensis* has only nine maxillary alveoli (Salas-Gismondi et al., 2015), whereas the paralligatorids *Wannchampsus kirkpachi* (Adams, 2014) and the 'Glen Rose Form' (USNM 22039) have 11 alveoli. Although counts of maxillary alveoli do not appear to be normally distributed (Shapiro-Wilk's test = 0.83, $p < 0.001$), there are no obvious discontinuities that naturally delimit the data. Nevertheless, character state boundaries follow Jouve et al. (2008).

Mandible

18. External mandibular fenestra shape, ratio of anteroposterior length (between anterior and posterior limits) to dorsoventral height (between dorsal and ventral limits): < 2.5 (0); ≥ 2.5 (1) (after Montefeltro et al., 2013 [306]; Groh et al., 2020 [56]).

    The proportions of the external mandibular fenestra (EMF) are measured using the maximum length of its axes horizontally and vertically (Fig. 3A), not diagonally (i.e. the maximum and minimum axes used by Groh et al., (2020: fig. 56). The EMF is approximately two times longer than tall

in most eusuchians examined here (average = 1.89). The most elongate fenestrae were measured in *Diplocynodon darwini* (2.82) (HLMD Me 7500) and *Borealosuchus sternbergii* (2.79) (UCMP 133930). By contrast, several *Crocodylus* species have more equidimensional fenestrae, e.g. *C. palustris* (1.21) (NHMUK 1868.4.9.11) and *C. niloticus* (1.26) (NHMUK 1900.9.22.2). Measured values are normally distributed (Shapiro-Wilk's test = 0.97, p = 0.08). The cut-off value used in earlier datasets (3) is uninformative, as all taxa examined here have a lower ratio. A discontinuity at a value of 2.5 was instead used to delimit character states (Document S2).

19. External mandibular fenestra shape, minimum angle subtended by dorsal margin of fenestra and the horizontal: $< 25°$ (0); $\geq 25°$ (1) (after Andrade et al., 2011; Groh et al., 2020 [55]).

    This character essentially describes the orientation of the long axis of the EMF using the inclination of its dorsal margin (Fig. 3B). As originally formulated, the character distinguished between a horizontally or anterodorsally orientated long axis, and was applied to a dataset comprising mostly non-crocodylian neosuchians (Andrade et al., 2011; Montefeltro et al., 2013). The original delimitation of the character is uninformative here, as the long axis of the EMF is inclined in all taxa in this dataset. Minimal inclination of the EMF is found in *Alligator mississippiensis* (10°) (NHMUK 68.2.12.6) and is steepest in *Mekosuchus inexpectatus* (55°) (MNHN NCP 06), with a full range of normally distributed values in between (Shapiro-Wilk's test = 0.97, $p = 0.16$). The character state boundary is based on a small discontinuity at 25° (Document S2).

20. Articular, retroarticular process, ratio of anteroposterior length (measured from the transverse ridge to the posteriormost tip of articular) to the mediolateral width across the glenoid fossa: < 1.5 (0); ≥ 1.5 (1) (after Lee and Yates, 2018 [217]).

    Lee and Yates (2018) characterised the length of the retroarticular process relative to its 'width'. Here the width is measured across the articular glenoid fossa, since the width of the retroarticular process is variable (narrowing posteriorly) (Fig. 3C). A cut-off of 1.5 is retained in the rediscretised character; however, although the data are not normally distributed (Shapiro-Wilk's test = 0.93, $p < 0.05$) there does not appear to be any natural discontinuity in this dataset (Document S2). Indeed, retroarticular process length varies continuously from the longest process measured in *Gavialis gangeticus* (1.89 times the width) (NHMUK 1974.3009), to the shortest process measured in *Mekosuchus inexpectatus* (1.0 times the width) (MNHN NCP 06).

Postcrania

21. Scapular blade, anteroposterior flare of dorsal end (at maturity): angle subtended by anterior and posterior margins $\geq 35°$ (0); $< 35°$ (1) (after Benton and Clark, 1988; Brochu, 1997a [22]).

Benton and Clark (1988) considered a narrow, subparallel-sided scapular blade to be diagnostic of Crocodylia, but a dorsally flaring scapula has been recognised in several crocodylians, e.g. *Gavialis gangeticus* and *Paleosuchus* (Brochu, 1999). In earlier datasets, dorsal flare of the scapular blade was essentially described as present or absent (e.g Brochu, 1997b; Brochu et al., 2012; Jouve et al., 2015; Lee & Yates, 2018); however, Brochu (1999) alluded to different degrees of flare that could be further delimited. The angle subtended by the anterior and posterior margins of the scapular blade was measured for this dataset (Fig. 3D), revealing a fully continuous and normally distributed range of values (Shapiro-Wilk's test = 0.96, $p = 0.12$). Scapular blade flare ranges from a minimum in *Caiman yacare* (flare = 8°) (AMNH 97300) to a prominent flare in *Hassiacosuchus haupti* (flare = 73°) (HLMD Be-137) (Document S2). A high degree of flare is also observed in *Bernissartia fagesii* (68°) (IRScNB 1538), *Borealosuchus formidabilis* (63°) (Erickson, 1976, fig.24), and *Diplocynodon darwini* (61°) (SMF Me-1289). When plotted, the largest discontinuity in the data occurs between a scapula flare of 32° (*Alligator prenasalis*, YPM PU 13799) and 38° (*Brachychampsa montana*, UCMP 133901), and so the rediscretised boundary was set in between these values at 35°.

22. Scapula-coracoid, ratio of maximum proximodistal coracoid length to maximum proximodistal scapula length: < 1.0 (0); ≥ 1 (1) (after Clark, 1994 [83]; Pol and Norell, 2004 [83]; Groh et al., 2020 [68]).

As originally formulated (Clark, 1994), this character described a coracoid that is either two-thirds the length of the scapula or equal in length to the scapula (Clark, 1994). This distinction is uninformative in this dataset, as all measurements of the coracoid to scapula length ratio are greater than 0.7 (Document S2). Measured values of the coracoid-scapula ratio are normally distributed (Shapiro-Wilk's test = 0.94, $p = 0.22$), but there is a small discontinuity between a number of taxa with a ratio greater than 1, e.g. *Gavialis gangeticus* (UCMZ R5783) and *Crocodylus johnstoni* (QM J58446), and all other taxa.

23. Coracoid shape, ratio of maximum expansion of distal coracoid, to maximum proximo-distal coracoid length: < 0.5 (0); ≥ 0.5 (1) (new character, based on personal observations).

The coracoids of several longirostrine crocodylians are proportionally slenderer than those of other crocodylians, exhibiting a small distal expansion relative to the proximo-distal length of the element (Fig. 3E). For example, whereas the ratio of distal expansion to coracoid length is <0.4 in *Piscogavialis jugaliperforatus* (SMNK 1282 PAL) and *Eogavialis africanum* (NHMUK R 3199), this ratio is > 0.6 in *Voay robustus* (NHMUK R36659) and *Asiatosuchus germanicus* (SMF Me 1801). A range of intermediate, normally distributed values occur between these extremes (Shapiro-Wilk's test = 0.99, $p = 0.99$), with no obvious discontinuities in the data (Document S2).

As such, a cut-off is set at 0.5 for the rediscretised analysis.

24. Ulna length, ratio of maximum proximodistal ulna length to maximum proximodistal humeral length: < 0.7 (0); ≥ 0.7 (1) (after Jouve, 2009 [330]; Groh et al., 2020 [69]).

    There are broad differences in the relative lengths of the ulna and humerus in Crocodylia (Document S2). In line with Iijima et al. (2018), *Gavialis gangeticus* has the shortest ulna in proportion to the humerus among extant crocodylians (ratio = 0.6) (Fig. 3F). This is similar to extinct "tomistomines", e.g. *Toyotamaphimeia* (0.5) (Iijima et al., 2018) and *Penghusuchus* (0.6) (Shan et al., 2009, fig.14). In general, the proportional length of the ulna to the humerus is lower in crocodyloids than in alligatoroids, in which they are more equidimensional in some taxa, e.g. *Hassiacosuchus haupti* (0.9) (HLMD Be-137) and *Wannanganosuchus brachymanus* (0.8) (Iijima et al., 2018). Measured values are not normally distributed (Shapiro-Wilk's test = 0.92, $p < 0.05$) and although the rediscretised threshold follows earlier studies (cut-off = 0.7) (Groh et al., 2020; Jouve, 2009) there is no notable discontinuity in the data.

25. Femur length, ratio of maximum proximodistal femur length to maximum proximodistal humeral length (at maturity): < 1.2 (0); ≥ 1.2 (1) (after Brochu, 1997a [33]; Jouve, 2009 [328]; Groh et al., 2020 [80]).

    Brochu (1997a) originally distinguished taxa with slender limbs, in which the fore- and hindlimb are subequal in length (*Borealosuchus*), from taxa with 'robust' limbs, with a longer hindlimb than forelimb (*Bernissartia fagesii* and all other crocodylians). Whereas the distinction in slenderness between *Borealosuchus* and all other crocodylians is captured in character 306 here, the current character quantifies the proportional differences in forelimb and hindlimb length. Ideally, total limb lengths would be measured; however, this would only allow a few exceptionally preserved fossil crocodylians to be considered, and therefore the relative lengths of the stylopodials are used as a proxy (Fig. 3G). Measurements are restricted to mature individuals given that the hindlimb grows with negative allometry relative to the forelimb in most extant crocodylians (Iijima & Kubo, 2019a). Alligatoroids tend to exhibit much longer femora than humeri, e.g. *Hassiacosuchus haupti* (1.5) (HLMD Be 137), *Tsoabichi greenriverensis* (1.3) (FMNH PR 1793), and *Wannaganosuchus brachymanus* (1.3) (Iijima et al., 2018). By contrast, the stylopodials of other crocodylians are subequal in length, e.g. *Gavialis gangeticus* (1.1) (UMZC R5783), *Tomistoma schlegelii* (1.1) (AMNH 113078), and most *Crocodylus* species ($\sim$1.0). Measured values are not normally distributed (Shapiro-Wilk's test = 0.90, $p < 0.05$), and a small discontinuity at a value of 1.2 is used to delimit the rediscretised character states (Document S2).

26. Ischial blade shape, ratio of maximum expansion of distal ischial blade to maximum proximodistal length of ischium: < 0.5 (0); ≥ 0.5 (1) (new character, based on personal observations).

The distal end of the ischial blade in *Gavialis gangeticus* prominently flares (AMNH 110145, UCMZ R5783). Indeed, measurements of the degree of flare (relative to ischium length, Appendix 3G) reveal that *Gavialis gangeticus* exhibits the highest value among taxa in this dataset (0.7). A similar expansion also characterises *Tomistoma schlegelii* (AMNH 113078) (0.6), contrasting with the narrower ischial blades of *Borealosuchus wilsoni* (FMNH PR 1674) (0.4) and most extant crocodylids, e.g. *Crocodylus johnstoni* (QM J58446) (0.4). A range of intermediate, normally distributed values occurs between these limits (Shapiro-Wilk's test = 0.99, $p = 0.96$). There is no informative discontinuity in the data and so a cut-off value is set at 0.5 based on those aforementioned values (Document S2).

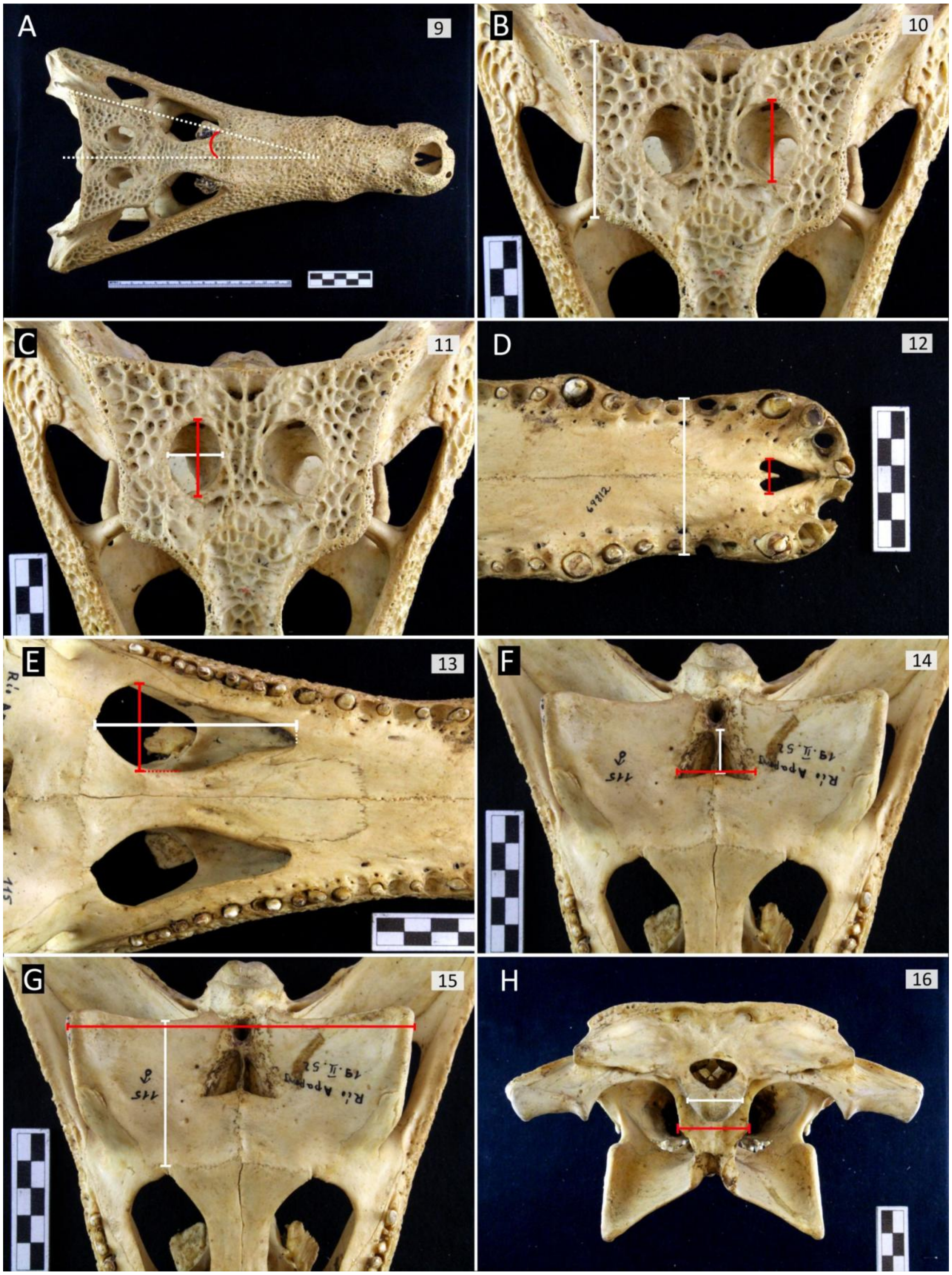

Figure 2: Continuous characters 9–16. All characters illustrated using *Caiman crocodilus apaporiensis* (FMNH 69812). Scale bars = cm.

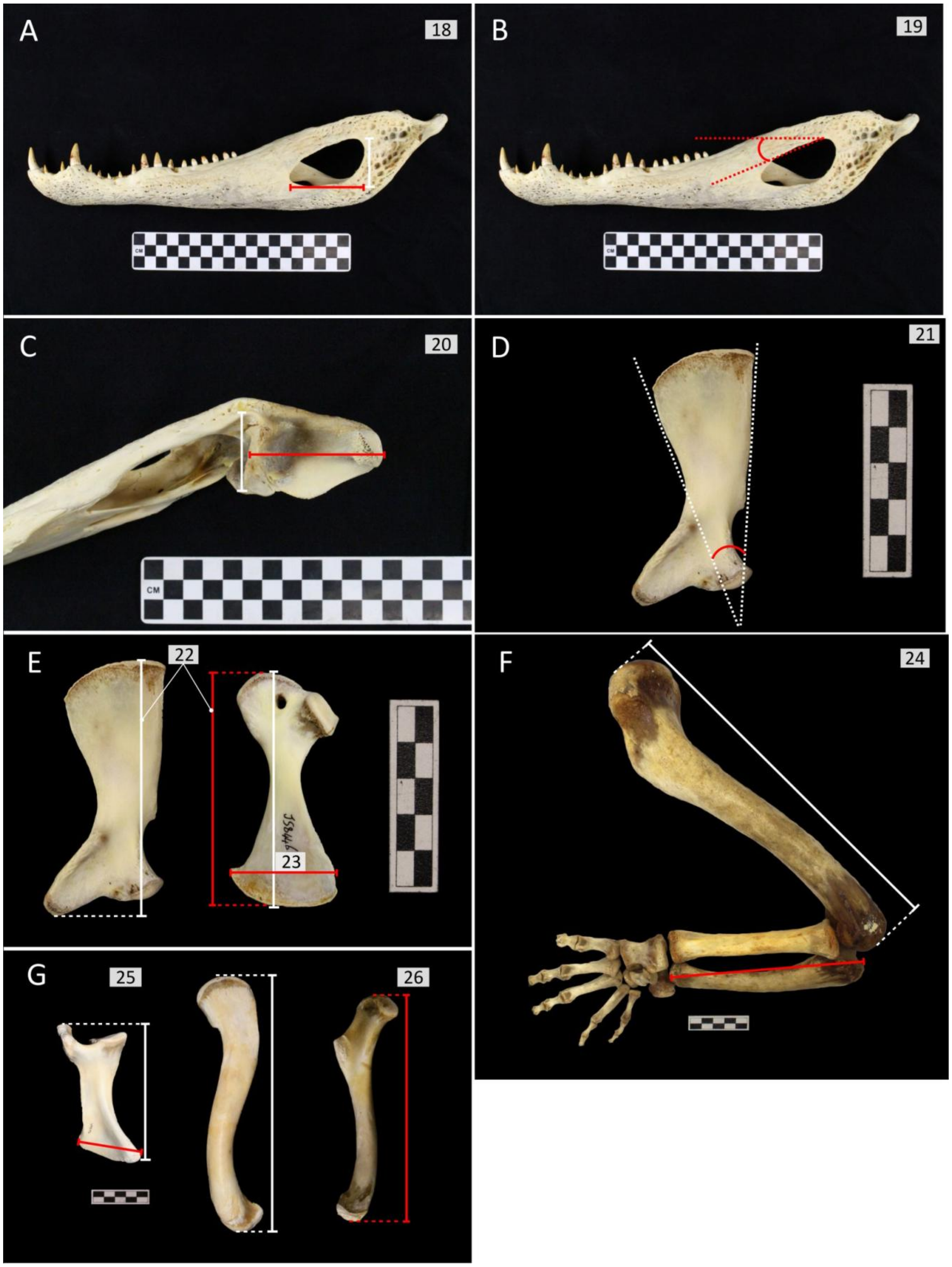

Figure 3: Continuous characters 18–26. A, C and E, *Caiman yacare* (AMNH 97300); B and G, *Crocodylus johnstoni* (QM J58446); D, left forelimb of *Gavialis gangeticus* (UMZC R 5783); F, (from left to right) left ischium, femur and humerus of *Crocodylus porosus* (QM J 48127). All scale bars = cm.

Discrete characters

Skull

Ornamentation

27. Rostral ornamentation, canthi rostralii: absent (0); present (1) (new character, after Norell, 1988 [34]; Brochu, 1997a [143]).

    Brochu (1997b) and later studies (e.g. Brochu et al., 2012; Jouve, 2016) used the term ‘canthus rostralis’ to describe anterolaterally directed rostral ridges that occur prominently in caimanines, e.g. *Melanosuchus niger* and *Caiman latirostris*. In this study, canthi rostralii (or canthal ridges) are distinguished from ‘rostral ridges’ (see Character 28), in that they extend anterolaterally from the dorsomedial margin of the orbit to the level of the 4th maxillary alveolus, imparting an angle that separates two planar surfaces on the skull (Fig. 4B). Taxa with canthi rostralii include *Paleosuchus palpebrosus* (Medem, 1958), *Hassiacosuchus haupti* (Fig. 4B), *Arambourgia gaudryii* (MNHN QU 17155), and *Boverisuchus vorax* (UCMP 170767), none of which have rostral ridges.

28. Rostral ornamentation, development of anterolaterally directed ridges on the lateral surface of the rostrum (at maturity): absent (0); present (1) (after Norell, 1988 [34]; Brochu, 1997a [143]).

    Anterolaterally directed rostral ridges typically occur in pairs, and are often associated with a spectacle (see Character 31). The largest ridge originates from the anteromedial margin of the orbit, and extends anterolaterally across the prefrontal, lacrimal and maxilla (Fig. 4D). A second, shorter anterior ridge extends anterolaterally over the nasal and maxilla. These ridges are not necessarily associated with a spectacle nor are they always paired, as is the case in *Purussaurus brasiliensis* (UFAC 1403), in which one prominent anterolateral ridge extends from the anteromedial orbital margin to the lateral edge of the maxilla.

29. Rostral ornamentation, dorsal boss on sagittal axis: absent (0); present (1) (after Brochu, 1997a [101]).

    As discussed by Brochu (2000), Neotropical *Crocodylus* species i.e. *C. acutus* (Fig. 4H), *C. intermedius*, *C. moreletti*, and *C. rhombifer*, are characterised by sulci on the nasal-maxilla sutures. This imparts a median elevation (boss) on the rostrum that is restricted mostly to the nasals, and is present throughout posthatching ontogeny (Brochu, 2000).

30. Rostral ornamentation, anteroposteriorly orientated preorbital ridges extending from the anterior corner of the orbit (at maturity): absent (0); present (1) (after Brochu, 1997a [144]).

A pair of anteroposteriorly orientated ridges extend from the anterior corner of the orbit in several crocodylians (Brochu, 2000). These ridges are particularly well-developed in Indopacific *Crocodylus* species such as *Crocodylus porosus* (Fig. 4F), *Crocodylus mindorensis*, *Crocodylus novaeguineae*, and *Crocodylus siamensis*. These ridges are always positioned on the medial edge of the lacrimal, adjacent to the lacrimal-prefrontal suture, and are typically straight; however, in some *Crocodylus siamensis* individuals (NHMUK 1924.4.1.168), the ridges are sigmoidal in shape. As discussed by Brochu (2000), juvenile *Crocodylus johnstoni* is also characterised by such ridges, but they are lost at maturity. This is also the case in *Crocodylus palustris* (NHMUK 1845.1.8.204, 1868.4.9.11). Preorbital ridges are not restricted to *Crocodylus*, but occur in several osteolaemines e.g. *Osteolaemus tetraspis* (NHMUK 1862.6.30.5), *Voay robustus* (NHMUK R 36685), ans *Euthecodon armabourgi* (MNHN ZEL 001), as well as mekosuchines such as *Baru wickeni* (QM 16822) and *Quinkana* ssp. (Megirian, 1994; Molnar, 1981). A number of taxa are newly scored for the derived character state in this study, including several species of *Diplocynodon* where they are very weakly developed: *D. hantoniensis* (Chapter 2) *D. ratelii* (MNHN SG 539), and *D. remensis* (Martin et al., 2014).

31. Rostral ornamentation, transverse ridge between the orbits (i.e. spectacle): absent (0); present (1) (after Barrios, 2011 [109]; Cidade et al., 2017 [186]; Lee and Yates, 2018 [56]).

32. Rostral ornamentation, morphology of the transverse orbital ridge (i.e. spectacle): low, lacking a posterior fossa (0); tall, with deep posterior fossa (1) (new character, based on personal observations).

33. Rostral ornamentation: anterior extent of transverse bridge between orbits (i.e. spectacle): posterior to anterior orbital margin (0); level with or anterior to anterior orbital margin (1) (new character, after Cossette and Brochu, 2018).

The presence of a step approximately at the level of the anterior orbital margin is widespread among both extant and fossil crocodyliforms (Delfino et al., 2008a). This step is often referred to as a ‘spectacle’, especially in reference to extant caimanines (e.g. the spectacled caiman – *Caiman crocodilus*) in which this bony interorbital bridge is very prominent. In this study, presence (Character 31), size variation (Character 32), and position (Character 33) of a spectacle are all recognised as characters. The first of these describes the presence or absence of any change in elevation around the anterior orbit margin, and is equivalent to characters 186 in Cidade et al. (2017) and 56 in Lee and Yates (2018). By contrast with the character scores in those studies, here the spectacle is found to be more widely distributed among Crocodylia (Fig. 5). For example, in addition to most extant caimanines, the spectacle is found in the putative early caiman *Eocaiman cavernensis* (AMNH 3158), all but one species of *Alligator* (*A. mcgrewi*), *Navajosuchus mooki*

(as scored in Lee and Yates [2018], but not Cidade et al. [2017]), *Leidyosuchus canadensis* (Wu et al., 2001a, fig.2.1) and most species of *Diplocynodon* (except *D. darwini* and *D. deponiae*). Also noteworthy is the presence of a spectacle in some basal crocodyloids, such as *Asiatosuchus grangeri* (AMNH 6607) and *Jiangxisuchus nankangensis* (Li et al., 2019, fig.2A).

Most of the above listed taxa, such as *Alligator* and *Diplocynodon* have a low spectacle, which lacks a deep fossa on the anterior margin of the step (C32-0, Fig. 6). A different condition is expressed in several caimanines such as *Caiman latirostris*, in which the vertical wall of the spectacle is excavated by a deep fossa (C32-1, Fig. 5E–F). Further variation occurs in the posteriormost extent of the spectacle relative to the anterior margin of the orbit (character 33) (Fig. 6). The spectacle rarely forms a straight horizontal bridge across the rostrum; instead, the spectacle is anteriorly concave. The apex of the concavity varies in position relative to the orbit. In all extant caimanines, the spectacle does not extend posteriorly beyond the level of the anterior margin of the orbit (C33-1). However, several fossil taxa, including *Purussaurus neivensis*, *Diplocynodon*, and *Bottosaurus harlani* exhibit a strong posterior shift in the spectacle (C33-0). The morphology of the spectacle in *Bottosaurus* was originally described as a "distinct 'U'-shaped depression", and used to diagnose the genus (Cossette & Brochu, 2018, p.4). Here, it is regarded as a posteriorly shifted spectacle, a condition similar to that of the giant Miocene caimanine, *Purussaurus neivensis* (UCMP 39704).

34. Rostral ornamentation, extensive fossa extending anteriorly from the frontal to the posterior margin of the external naris: absent (0); present (1) (new character, based on personal observations).

    The derived character state describes a unique condition of the naris present only in *Purussaurus brasiliensis* (Fig. 7B) and *Purussaurus mirandai* (Aguilera et al., 2006). Unlike all other eusuchians, the naris covers almost the entire anteroposterior length of the rostrum in these two species, and the posterior margin of the naris merges continuously into a large fossa on the rostrum.

35. Prefrontal, prominence at anteromedial orbital margin: not thickened (0); hypertrophied, forming rounded protuberances (1) (after Bona et al., 2013b [167]; Cidade et al., 2017 [186]; Souza-Filho et al., 2019 [182]).

    As discussed by Cidade et al. (2017), a protuberance or 'knob' at the anteromedial margin of the orbit is diagnostic of the caimanine *Mourasuchus*, present in all three *Mourasuchus* species studied here (e.g. *Mourasuchus arendsi*, Fig. 8B). This thickening occurs on the prefrontal orbital margin, and in all *Mourasuchus* species occurs along with a spectacle (31-1). The spectacle is nonetheless independent of the prefrontal protuberances, since several crocodylians with a spectacle lack a prefrontal protuberance, e.g. *Caiman* (Fig. 6G–I).

36. Cranial table ornamentation, fossa on the sutural intersection of the postorbital, frontal and parietal:

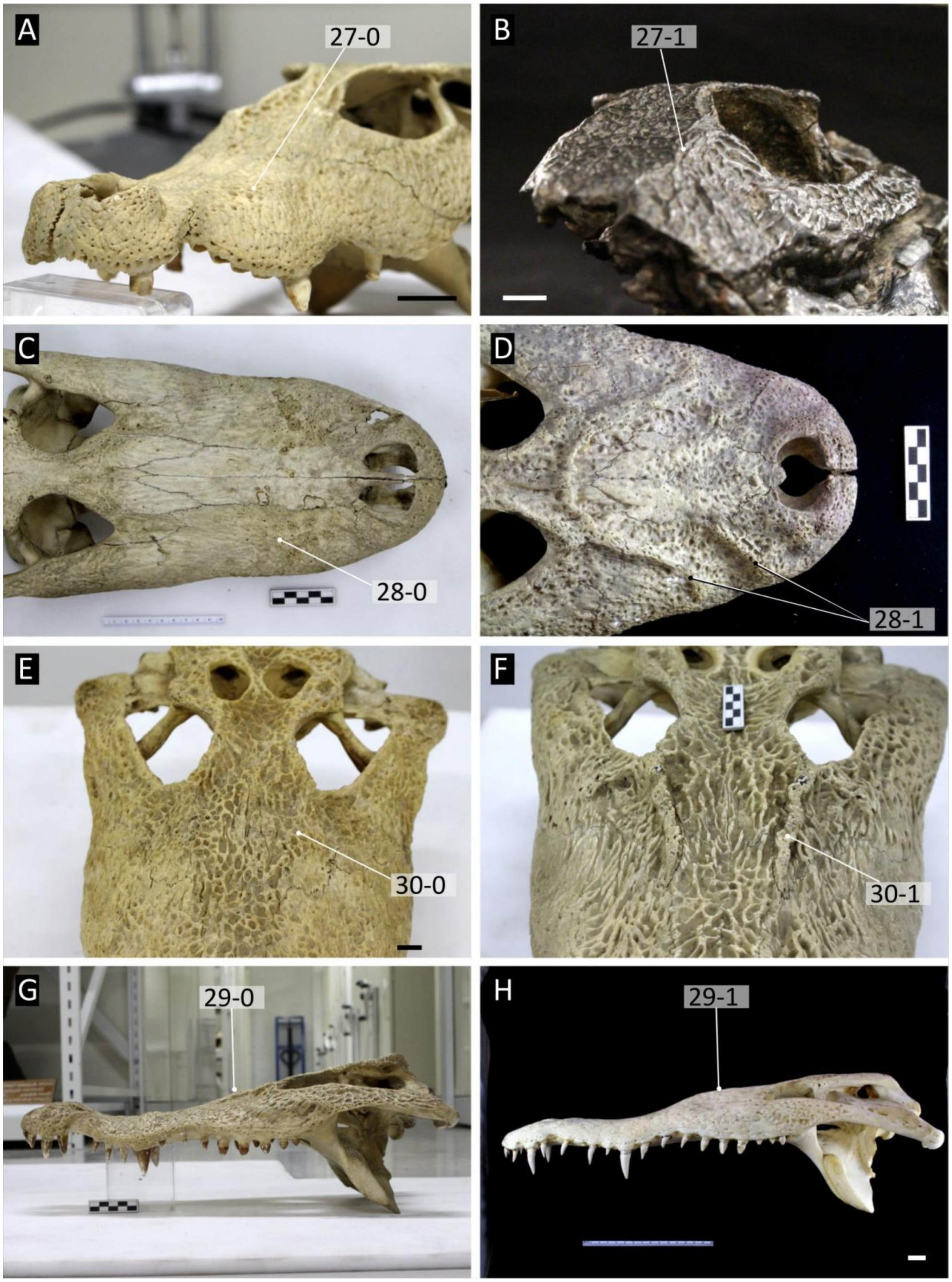

Figure 4: Variation in rostral ornamentation in Crocodylia. **A**, *Osteolameus tetraspis* (NHMUK 1862.6.30.5); **B**, *Hassiacosuchus haupti* (HLMD Me-4415); **C**, *Alligator mississippiensis* (NHMUK 1873.2.21.1); **D**, *Caiman latirostris* (FMNH 9713); **E**, *Crocodylus palustris* (1897.12.31.1); **F**, *Crocodylus porosus* (NHMUK 1852.12.9.2); **G**, *Crocodylus siamensis* (NHMUK 1921.4.1.168); **H**, *Crocodylus acutus* (FMNH 69884). Scale bars in A, B, E, and H = 2 cm, all other scale bars = cm.

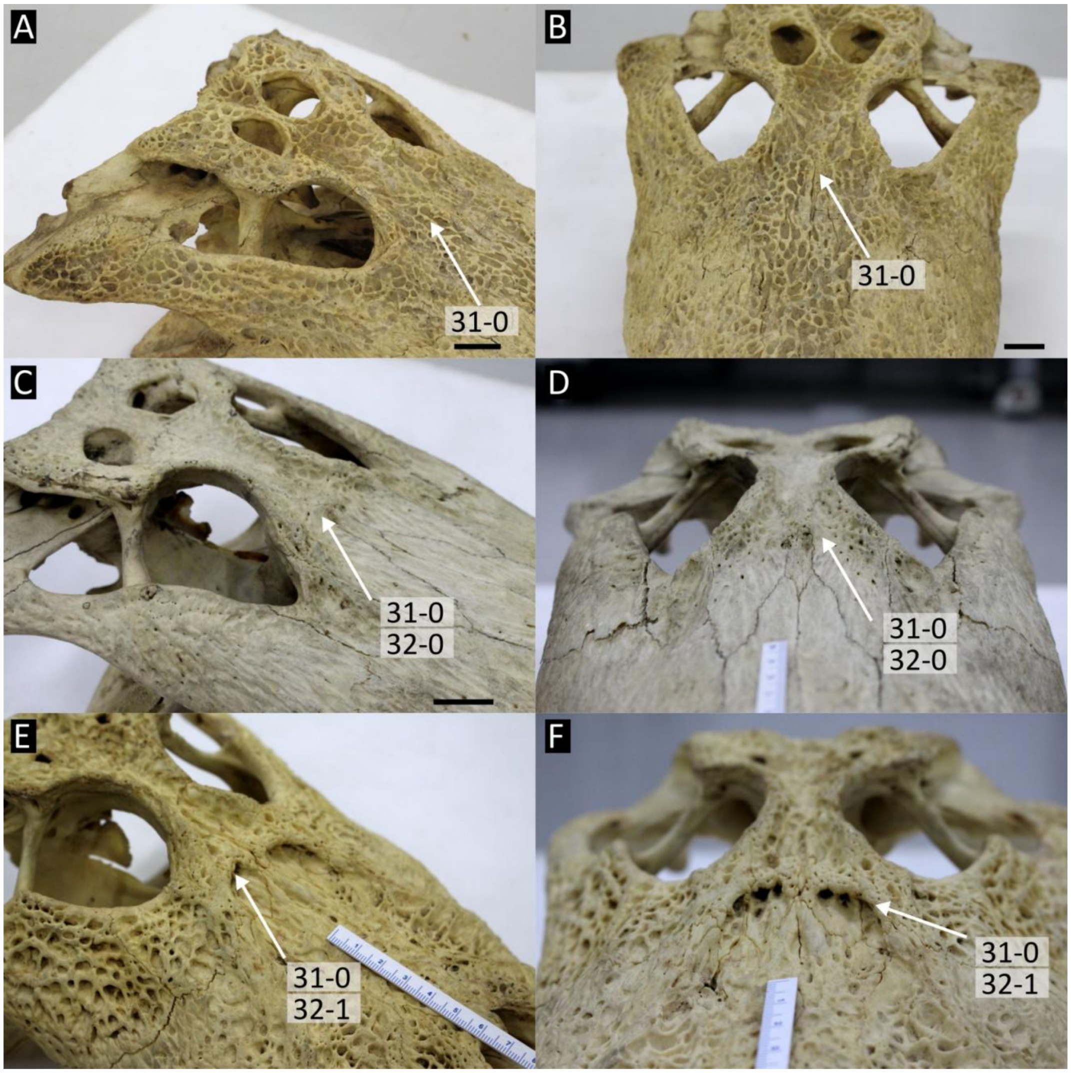

Figure 5: Variation in development of a transverse orbital ridge (spectacle). (**A**–**B**) *Crocodylus palustris* (NHMUK 1897.12.31.1); (**C**–**D**) *Alligator mississippiensis* (NHMUK 1873.2.21.1); (**E**–**F**) *Caiman latirostris* (NHMUK 1897.12.31.1). All scale bars = 2 cm.

absent (0); present (1) (after Lee and Yates, 2018 [71]; adapted from Willis et al., 1993).

A pit that occurs on each side of the skull at the triple junction of the postorbital, parietal and frontal was first described in *Kambara murgonensis* (Willis et al., 1993), and later observed in all species of *Kambara* that preserve the cranial table (Buchanan, 2009; Salisbury & Willis, 1996). These pits are easily distinguished from the characteristic pitted crocodylian dermatocranium by their large size, as well as their paired nature (Fig. 9).

37. Skull table morphology: posterolateral edges directed ventrolaterally from the sagittal axis (0); planar across entire length, or lateral edges directed dorsolaterally $<20°$ across entire length (1); lateral edges directed dorsolaterally $\geq 20°$ along entire length (2) (after Brochu, 1997a [123]; Barrios, 2011 [108]; Jouve, 2016 [123]; Cidade et al., 2017 [185]) (ORDERED).

This character is modified from Brochu (1997b, character 123) by the addition of a third character state (37-2), and the quantification and ordering of the character states. In occipital view, the skull table of *Bernissartia fagesii* (IRScNB 1538) is approximately planar, with the lateral edges

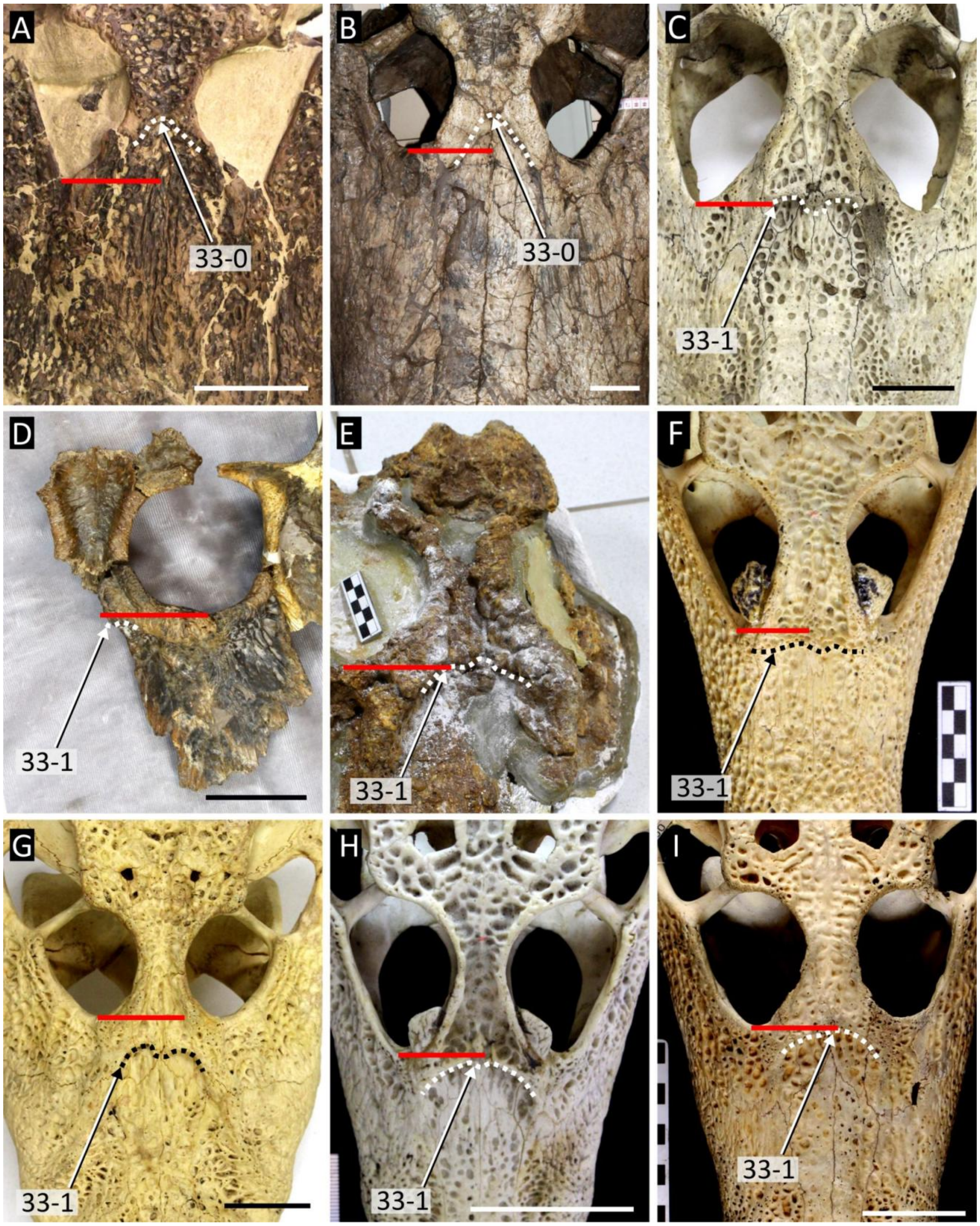

Figure 6: Variation in the position of the transverse orbital ridge (spectacle). **A**, *Diplocynodon hantoniensis* (CAMSM TN 907); **B**, *Purussaurus neivensis* (UCMP 39704); **C**, *Melanosuchus niger* (NHMUK 45.8.25.125); **D**, *Mourasuchus atopus* (UCMP 38012); **E**, *Mourasuchus arendsi* (UFAC 5883); **F**, *Caiman crocodilus apaporiensis* (FMNH 69812); **G**, *Caiman latirostris* (NHMUK 1897.12.31.1); **H**, *Caiman crocodilus chiapasius* (FMNH 73701); **I**, *Caiman yacare* (AMNH 97300). Dashed lines mark spectacle position, red line marks anterior margin of orbit. All scale bars = 5 cm.

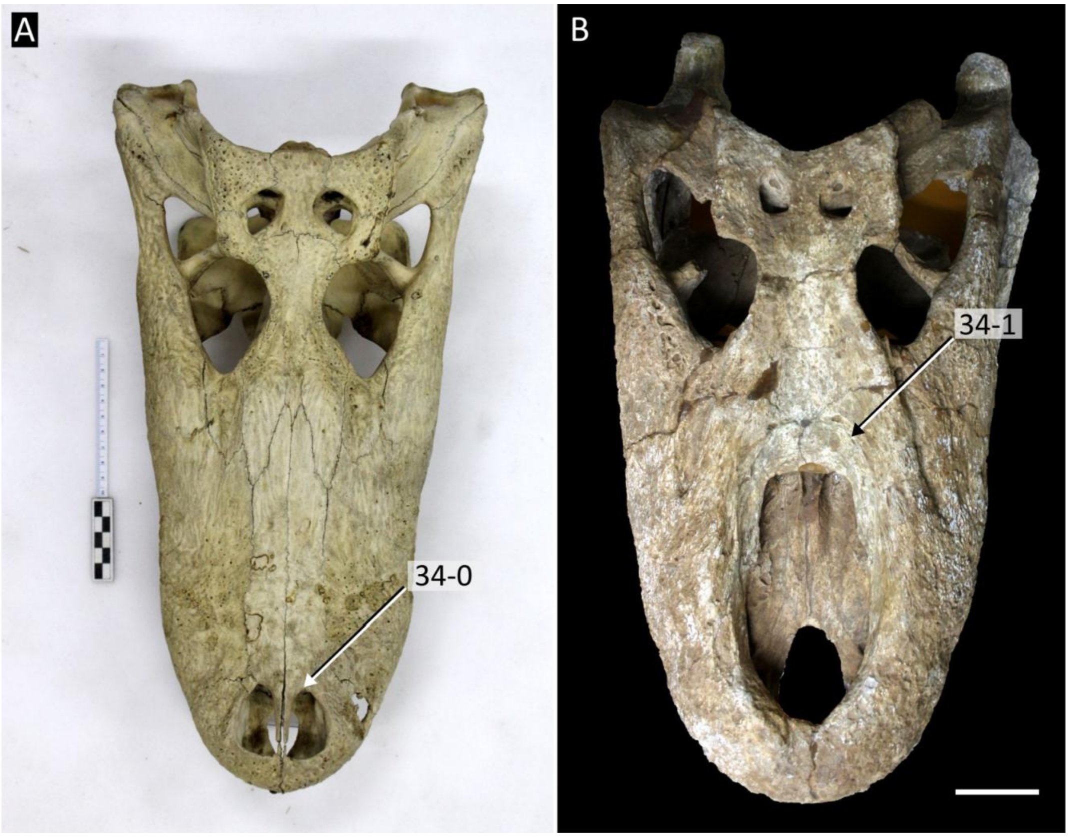

Figure 7: Comparison of the morphology of the naris in **A**, *Alligator mississippiensis* (NHMUK 1873.2.21.1); **B**, *Purussaurus brasiliensis* (UFAC 1403). Scale bar = 30 cm.

upturned less than 20° (37-1). This condition is common to most eusuchians in this dataset (Fig. 10C–F). By contrast, the lateral edges of the cranial table slope ventrally from the sagittal axis in several "gavialoids" (37-0), e.g. *Gavialis gangeticus* (Fig. 10A–B) and *Gryposuchus neogaeus* (MLP 26-413), as well as in the non-crocodylian eusuchian *Hylaeochampsa vectiana* (NHMUK R177) (albeit to a lesser degree). Thus far, these observations follow the original scoring of the character as implemented in earlier studies (e.g. Brochu, 1999; Brochu et al., 2012; Lee & Yates, 2018; Salas-Gismondi et al., 2015). However, a third character state is added based on character 185 in Cidade et al. (2017), which appears to describe a continuation of the dorsal upturning of the lateral cranial table edges, which is found in species of *Purussaurus* (37-2, edges orientated $\geq 20^\circ$) (Fig. 10G–H). Jouve (2016) also introduced a third state to the same character, which described a skull table that is "medially depressed". This morphology is distinct from the condition in *Purussaurus*; furthermore, it is not considered homologous to the morphology described in this character. As such it is discretised separately as Character 82.

It is possible for Character 37 to be conflated with the presence or absence of squamosal horns (discrete bony protrusions on the posterior margin of the cranial table, see characters 38–40). In-

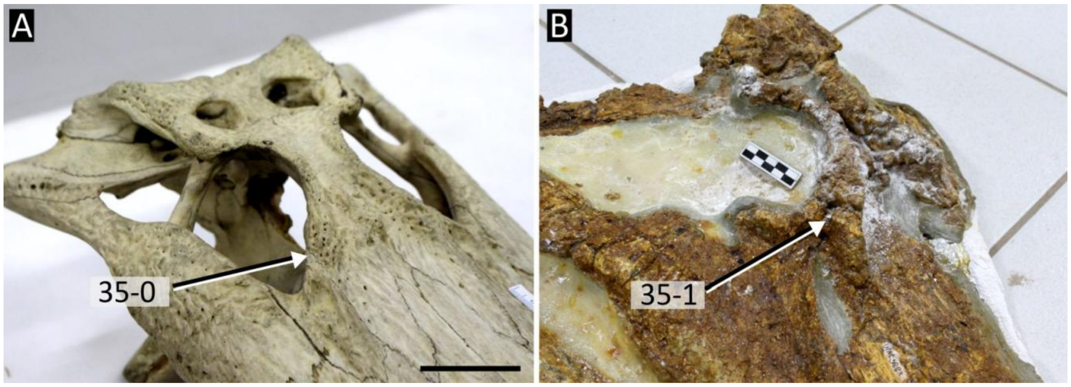

Figure 8: Dorsolateral view of the orbit, showing development of a protuberance on the prefrontal in **A**, *Alligator mississippiensis* (NHMUK 1873.2.21.1); **B**, *Mourasuchus arendsi* (UFAC 5883). All scale bars = 5 cm.

deed, in the data matrix of Souza-Filho et al. (2019), *Purussaurus* is scored as having squamosal horns. By contrast, we regard *Purussaurus* as lacking horns, and suggest that Souza-Filho et al. (2019) conflate the broad concavity of the skull table in this taxon (resulting in upturned lateral cranial table margins, Fig. 10G–H) with a hypertrophied skull table (true squamosal horns, Fig. 11C–H). We regard the curvature of the cranial table and the development of horns as independent. For example, whereas some taxa have squamosal horns and a concave skull table (*Acresuhcus pachytemporalis*, UFAC 2507), others have squamosal horns and a flat skull table (*Certaosuchus burdoschi*, FMNH P 15576) and some species (including *Purussaurus neivensis*, UCMP 39704) lack squamosal horns, but have a concave skull table.

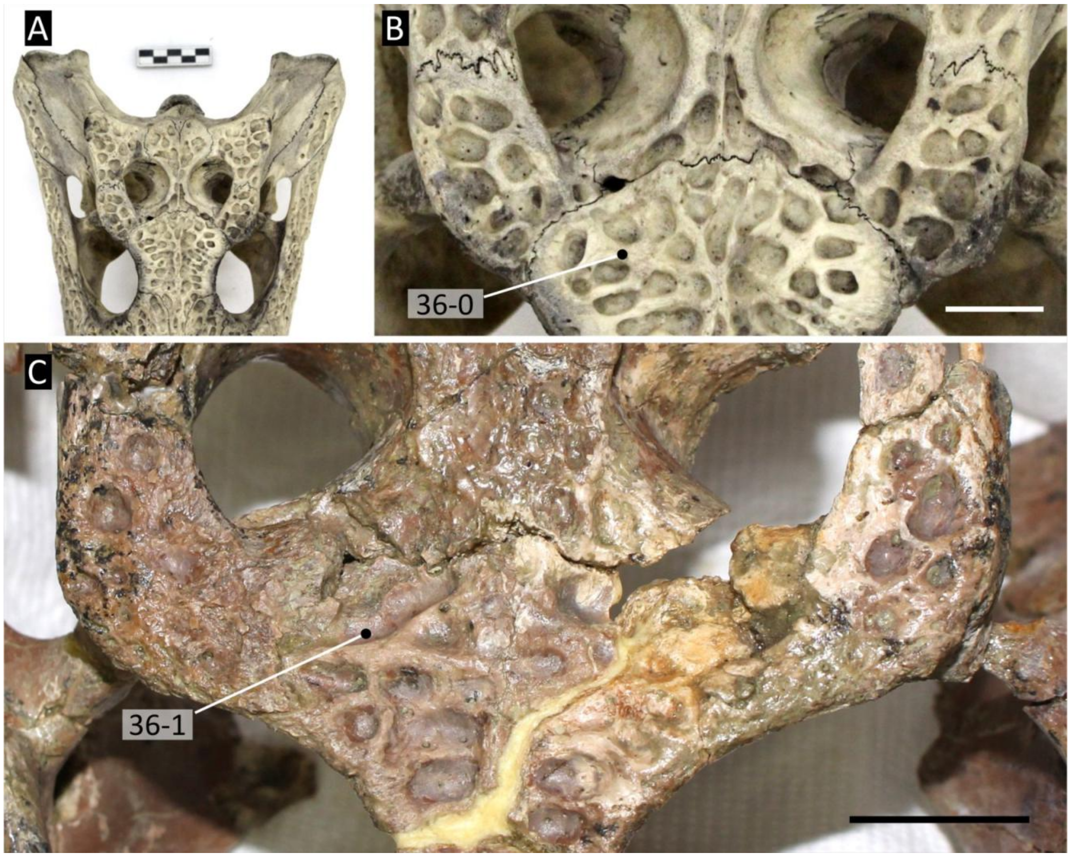

Figure 9: Dorsal view of the cranial table in **A**-**B**, *Crocodylus acutus* (NHMUK 1975.997); and **C**, *Kambara implexidens* (QM 29662). Scale bar A = cm, scale bars B, C = 2 cm.

38. Cranial table ornamentation: posterolateral and/or posterior margin of squamosal flat (0); upturned to form a discrete eminence (i.e. a squamosal horn) (1) (after Brochu, 2011 [157]).

39. Cranial table ornamentation, squamosal horn position: restricted to posterior end of skull table (0); extends anteriorly along the whole lateral margin of the skull table (1) (after Salas-Gismondi et al., 2015 [157]; Souza-Filho et al., 2019 [156]).

40. Cranial table ornamentation, direction of squamosal horn expansion from cranial table: dorsally only (0); dorsally and laterally (1) (after Souza-Filho et al., 2019 [156]).

Squamosal horns are abrupt dorsal/dorsolateral projections of the squamosal (sometimes also including the postorbital) (Fig. 11C–H). Among extant crocodylians, squamosal horns are consistently found only in *Crocodylus siamensis* and *Crocodylus rhombifer* (Brochu & Storrs, 2012). Large, overgrown individuals of *Crocodylus* species can also develop a thickening of the lateral margin of the cranial table, which resembles squamosal horns (Brochu et al., 2010). Here these are differentiated from 'true' squamosal horns by having more rounded/ less acute dorsal apices, and by a gradual transition from the anterior end of the skull table to the posterior end. As orig-

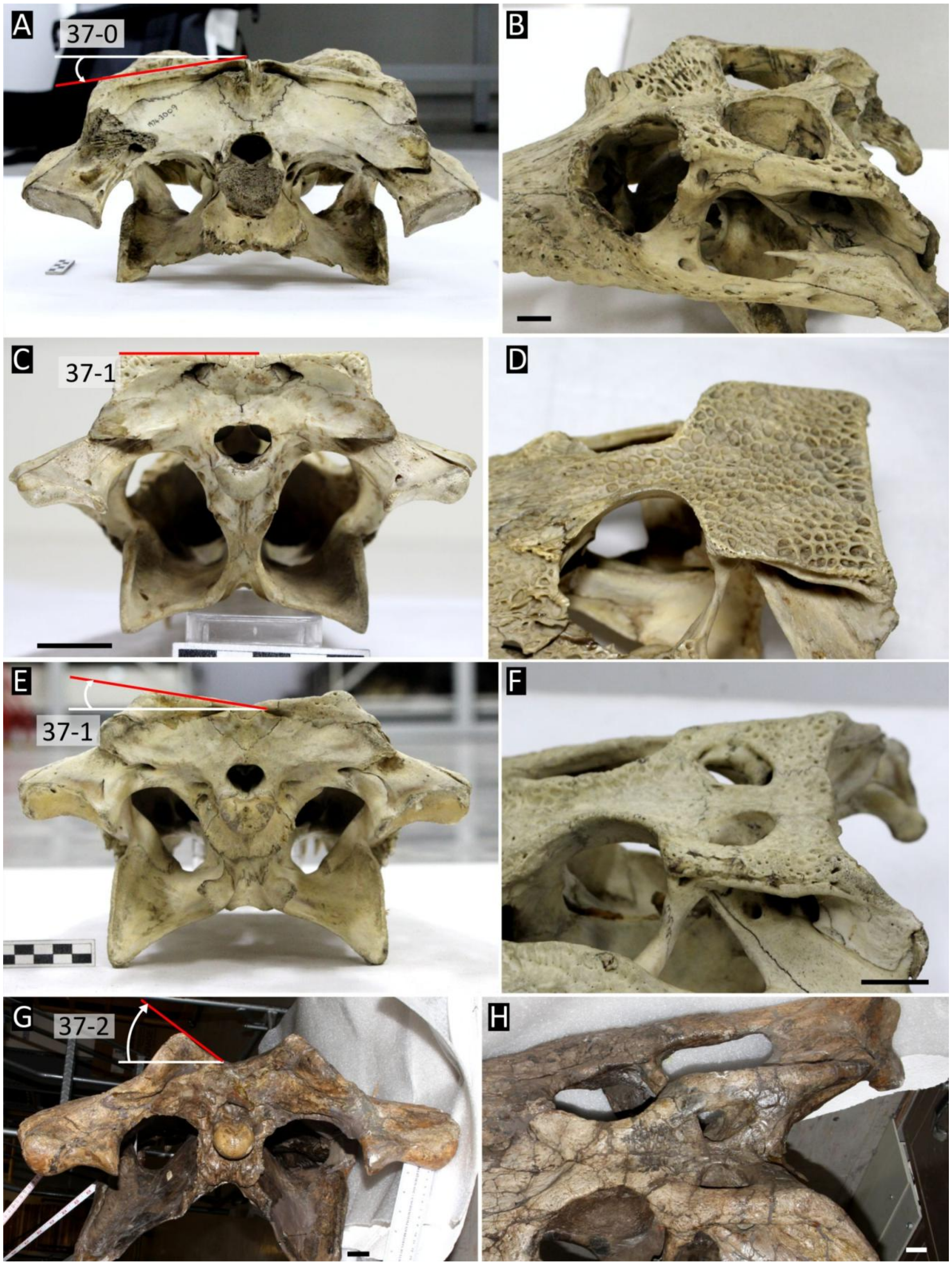

Figure 10: Comparisons of cranial table morphology in occipital (left) and dorsolateral (right) views. **A–B**, *Gavialis gangeticus* (NHMUK 1974.3009); **C–D**, *Paleosuchus trigonatus* (NHMUK 1868.10.8.1); **E–F**, *Alligator mississippiensis* (NHMUK 1873.2.21.1); **G–H**, *Purussaurus neivensis* (UCMP 39704). All scale bars = 2 cm.

inally formulated by Brochu (2011), the absence or presence of a squamosal horns is treated as a binary character; however, as illustrated by Souza-Filho et al. (2019, fig.13), squamosal horns can exhibit discrete morphological differences. Characters 38–40 are derived by reductively coding a multistate character (156) in Souza-Filho et al. (2019). Variation in squamosal horn morphology can be broadly divided into: (1) the position of the horn (Character 39); and (2) the direction of the horn's projection (Character 40). In the caimanine *Mourasuchus* the squamosal horns are entirely restricted to the posterior end (39-0) (Fig. 11C–D). By contrast, the squamosal horns of *Crocodylus siamensis*, *Crocodylus rhombifer*, *Voay robustus*, *Acresuchus*, and *Ceratosuchus* extend over most of the anteroposterior length of the cranial table, being tallest at the posterior end (39-1) (Fig. 11E, G). Whereas *Ceratosuchus* and *Voay* share anteriorly extensive squamosal horns, the horns are dorsally directed in *Ceratosuchus* (40-0) (Fig. 11F), but dorsolaterally directed in *Voay* (40-1) (Fig. 11H).

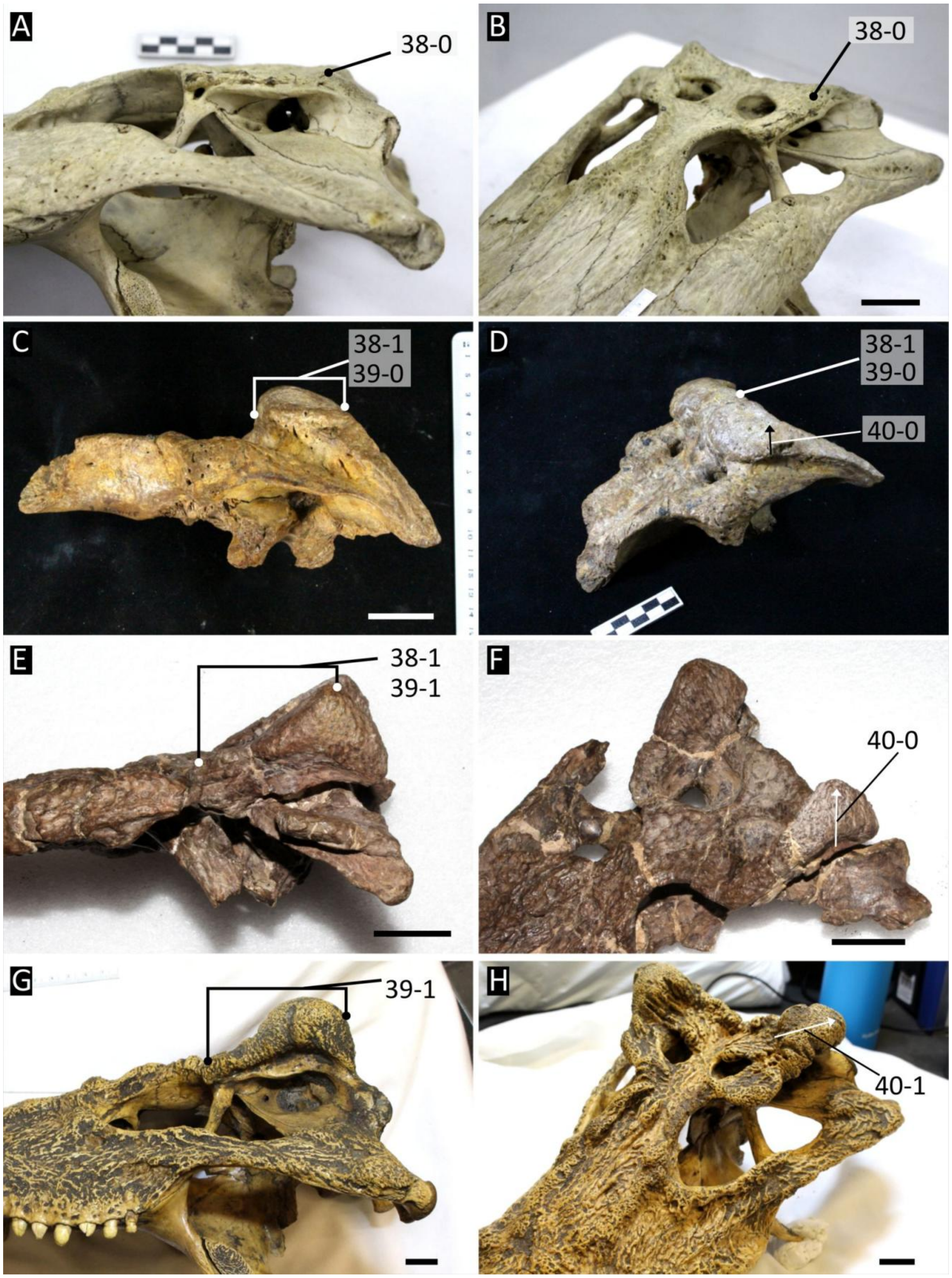

Figure 11: Left lateral (left) and dorsolateral (right) views of the cranium showing variation in development of squamosal horns. **A**-**B**, *Alligator mississippiensis* (NHMUK 1873.2.21.1); **C**-**D**, *Mourasuchus arendsi* (MLP 73-IV15-8); **E**-**F**, *Ceratosuchus burdoschi* (FMNH P 15576); **G**-**H**, *Voay robustus* (NHMUK R 366885). All scale bars = 3 cm.

External nares

41. External nares, orientation: projects anterodorsally (0); dorsally (1) (after Brochu, 1997a [79]).

In most crocodylians, the margins of the external naris are approximately in the same plane, resulting in dorsally facing external nares (Fig. 12C). By contrast, the anterior and anterolateral margins of the nares in some crocodylians are depressed, resulting in anterodorsally facing nares (Fig. 12A–B). Similar to previous studies, anterodorsally facing external nares are herein scored as present in 'basal' alligatorines such as *Navajosuchus* and *Allognathosuchus*, *Borealosuchus* (Fig. 12A), and several non-crocodylian taxa (*Bernissartia fagesii*, *Shamosuchus*, *Theriosuchus*, and the 'Glen Rose Form') (Brochu, 1999; Brochu et al., 2012; Jouve, 2016; Salisbury et al., 2006). A number of mekosuchines also exhibit anterodorsally facing nares, such as *Baru wickeni* (QM F16822), *Mekosuchus inexpectatus* (MNHN NCP 06), and *Quinkana* (Megirian, 1994). By contrast with some studies (e.g. Brochu et al., 2012; Jouve, 2016; Lee & Yates, 2018), anterodorsally facing nares are also identified in some species of *Diplocynodon* (e.g. *D. hantoniensis*, Fig. 12b), and in the osteolaemine, *Voay robustus* (NHMUK R36685).

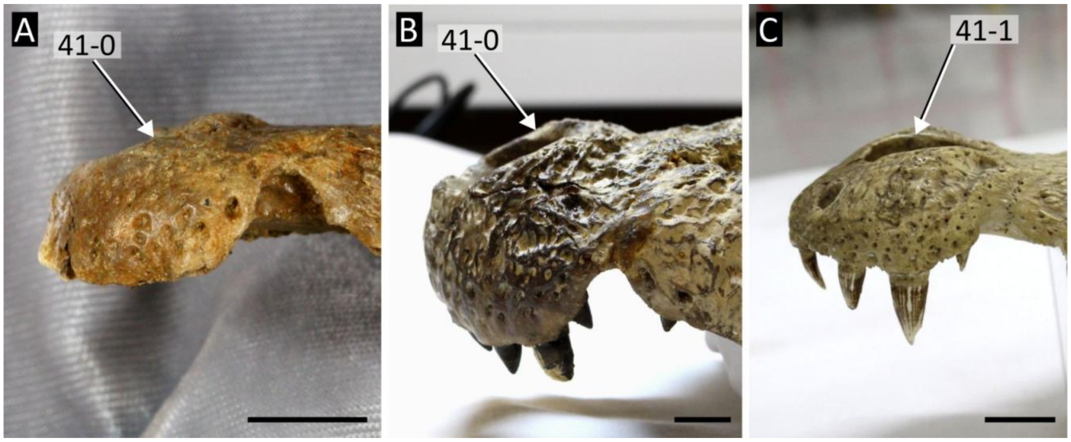

Figure 12: Lateral view of the external naris in **A**, *Borelaosuchus sternbergii* (UCMP 126099); **B**, *Diplocynodon hantoniensis* (NHMUK 25166) and **C**, *Crocodylus siamensis* (NHMUK 1921.4.1.168). All scale bars = 2 cm.

42. External nares, development of bony excrescence (ghara) in reproductively mature males: absent (0); present (1) (after Brochu, 2011 [84]).

The ghara is a bulbous, bony outgrowth on the dorsal margin of the nares. Among extant crocodylians, the ghara is found only in mature, male *Gavialis gangeticus*, where it serves as a resonating chamber (Grigg & Kirshner, 2015) (Fig. 13B). It generally occurs in male individuals of at least 3 m total body length; however, smaller individuals (2.4 m) and females in captivity have on occasion exhibited a ghara (Martin & Bellairs, 1977). In osteological specimens, evidence for the ghara occurs as a fossa, delimited by a thin crest anterior to the external nares. In dorsal view, the premaxillae also

appear more circular in *Gavialis gangeticus* specimens that have a ghara. This partially obscures the characteristic posteriorly tapering outline of the premaxillae in *Gavialis*. In this study, only taxa known from at least two large (and therefore mature) crania preserving the nares were scored. The ghara is here recognised only in *Gavialis gangeticus* and *Gryposuchus colombianus*; however, a number of other fossil crocodylians that were not included in this study also possess a ghara including *Gavialis bengawanicus* (Martin et al., 2012) and *Rhamphosuchus crassidens* (Cautley & Falconer, 1840).

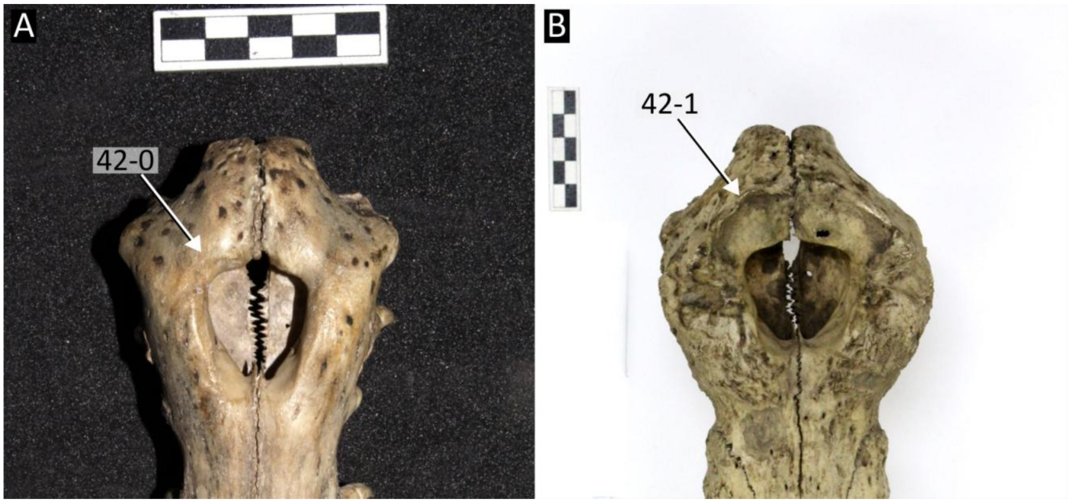

Figure 13: Dorsal view of the external naris showing development of the ghara in *Gavialis gangeticus*. **A**, NHMUK 61.4.1.2; **B**, NHMUK 1974.3009.

43. External nares, thin crest circumscribing narial margin: absent (0); present (1) (after Brochu, 2011 [85]).

In most crocodylians the margins of the external nares are flush with the dorsal surface of the premaxilla (Fig. 14A). A slightly everted margin of the nares was first described in the 'basal' alligatoroid *Diplocynodon muelleri* (Piras & Buscalioni, 2006), and later recognised in the caimanine *Tsoabichi greenriverensis* (Brochu, 2010) (Fig. 14B). A homologous crest is newly recognised in *Diplocynodon tormis* (Buscalioni et al., 1992), *Diplocynodon deponiae* (SMF Me 2609), and occurs variably in *Diplocynodon darwini* (absent in HLMD Me 7500, present in SMNK uncatalogued material). A narial crest is not recognised in *Paleosuchus palpebrosus* contrary to character scores in Lee and Yates (2018).

44. Premaxilla, notch posterolateral to naris: absent (0); present (1) (after Brochu, 1997a [142]).

A sulcus on each side of the rostrum, posterolateral to the external nares, was originally recovered as an unambiguous synapomorphy of *Alligator* (Brochu, 1999) (Fig. 14A). This sulcus is

often associated with a swelling of the posterolateral margins of the nares. Later analyses identified this sulcus in a larger number of mostly alligatoroid taxa, such as *Arambourgia gaudryi*, *Procaimanoidea utahensis*, and *Brochuchus pigotti* (Brochu, 2011; Brochu et al., 2012). In this study, a posterolateral notch is also recognised in several *Diplocynodon* species, such as *D. hantoniensis* (Chapter 2), *D. muelleri* (Piras & Buscalioni, 2006), *D. tormis* (Buscalioni et al., 1992), and *D. ratelii* (MNHN SG 539). Furthermore, it occurs in some *Mourasuchus* species, e.g. *M. atopus* (UCMP 38012) and *M. arendsi* (UFAC 5716). The occurrence of a notch in *Mourasuchus* was also recognised by Cidade et al. (2017), who added a third character state in their analysis to distinguish *Mourasuchus* from all other crocodylians. According to Cidade et al. (2017) *Mourasuchus* has a: "*naris surrounded by a dorsoventrally developed rim*" (Cidade et al., 2017, character 86). However, the morphology of the narial notch in *Mourasuchus* is not notably different to that in *Alligator* (i.e. 44-1), and thus the addition of a third character state is rejected.

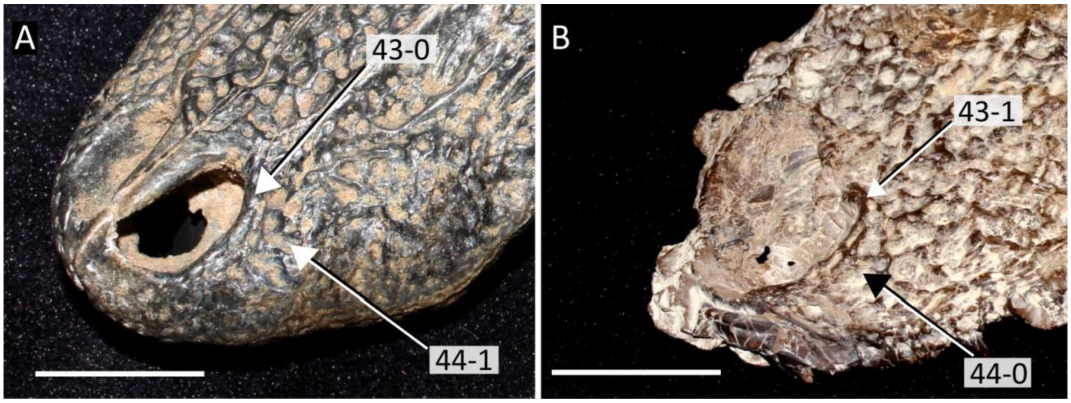

Figure 14: Dorsolateral view of the external naris in **A**, *Alligator mcgrewi* (AMNH F.A.M 7905); and **B**, *Tsoabichi greenriverensis* (AMNH 3666). All scale bars = 2 cm.

45. Premaxilla, fossa on the lateral margin of the naris: absent (0); present (1) (new character, based on personal observations).

    The derived character state applies to three alligatoroid taxa in this analysis: *Brachychampsa montana* (Gilmore, 1911; Norell et al., 1994), *Stangerochampsa mccabei* (Wu et al., 1996), and *Wannaganosuchus brachymanus* (Erickson, 1982). Unlike all other crocodylians, the lateral edges of the nares in these taxa bear a fossa, such that the inner lateral walls of the external nares are bevelled (Fig. 15B). The derived condition appears to be incipiently developed in some alligatorids (particularly *Alligator*); however, the condition is not developed strongly enough to be scored for the derived condition.

46. Nasals, external contact with naris: present (0); absent (1) (after Norell, 1988 [3]; Clark, 1994 [13, 14]; Brochu, 1997a [95]).

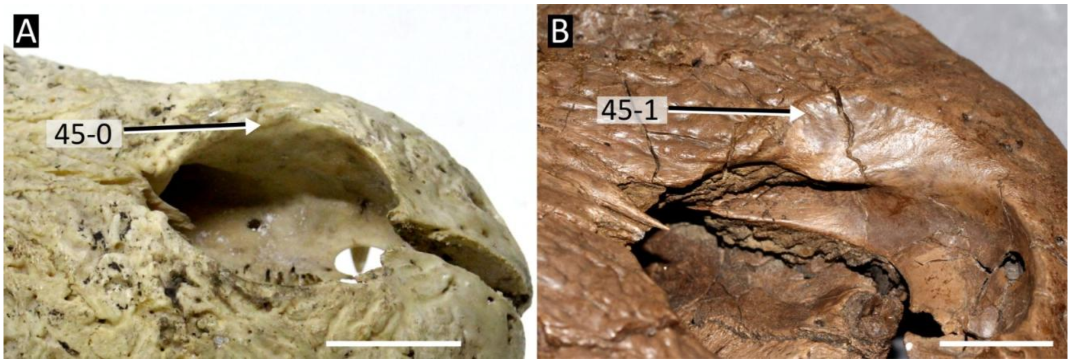

Figure 15: Dorsolateral view of the medial wall of the naris in **A**, *Crocodylus niloticus* (NHMUK 1934.6.3.1); **B**, *Brachychampsa montana* (UCMP 133901). All scale bars = 2 cm.

47. Nasals, bisect nares completely (0); protrude partially into posterior narial margin (1); excluded internally from posterior narial margin (2) (after Norell, 1988 [3]; Clark, 1994 [13, 14]; Brochu, 1997a [95]) (ORDERED).

Characters 46–48 are derived from reductively coding character 95 in Brochu (1999):

"*External naris bisected by nasals (0) or nasals contact external naris, but do not bisect it (1), or nasals excluded, at least externally, from naris; nasals and premaxillae still in contact (2), or nasals and premaxillae not in contact (3)*"

Whereas Character 46 describes the presence or absence of an external contact between the nares and nasals, Character 47 describes the degree of protrusion of the nasals internally. The distinction between external and internal contact was not accounted for in the original formulation of the character, i.e. some taxa could be scored for both character states 1 and 2 in character 95 of Brochu (1999). For example, *Caiman yacare* (AMNH 97300) and several other *Caiman* species lack an external nasal-naris contact (46-1), but exhibit an internal protrusion on the posterior wall of the naris (47-1). By contrast, *Tomistoma schlegelii* (NHMUK 1894.2.21.1), *Gavialis gangeticus* (NHMK 1974.3009), and *Diplocynodon hantoniensis* (NHMUK 30392), among other taxa, lack an external (46-1) and internal (47-2) nasal-narial contact.

48. Nasals, contact with premaxillae: present with overlap (0); present, point contact (1); absent (2) (after Norell, 1988 [3]; Clark, 1994 [13, 14]; Brochu, 1997a [95]; Jouve et al., 2008 [95]) (ORDERED).

The nasals extensively contact the premaxillae on the anterior end of the rostrum in almost all crocodylians, being wedged between the posterodorsal processes of the premaxillae (Fig. 16A–F). An exception to this occurs in *Gavialis*, in which the nasals terminate anterior to the mid-length of the rostrum (Fig. 16I). An intermediate condition was recognised by Jouve et al. (2008, pp. 95–3) in several longirostrines,who described this as a "weak contact" between the premaxilla and nasals.

This condition is incorporated here, but as part of an ordered multistate character describing the progressive separation of the nasals from the premaxillae. The original description as "weak" is replaced with "point contact" here, which is more precise. Taxa scored for character state 48-1 are mostly longirostrines such as *Piscogavialis jugaliperforatus* (Fig. 16G), *Ikanogavialis gameroi* (Sill, 1970) and *Gryposuchus neogaeus* (Fig. 16H). By contrast to the character scores in Jouve (2016), the condition in *Thoracosuaurs isorhynchus* (MNHN 1902.22; MNHN.F.MTA 61) and *Thoracosaurus neocesariensis* (multiple specimens in AMNH, YPM) is scored as missing, and *Eothoracosuaurs mississippiensis* (Brochu, 2004a) is considered to exhibit an extensive premaxilla-nasal contact (48-0). Furthermore, two brevirostrine taxa (*Purussaurus mirandai* [Aguilera et al., 2006] and *Purussaurus brasiliensis* [UFAC 1403]) are scored for the intermediate condition here, which is related to the peculiar development of an extensive narial opening in these taxa (see Character 34).

Premaxilla

49. Premaxilla-maxilla suture, anterior limit relative to posterior margin of external naris: posterior to (0); level with or anterior to (1) (after Jouve et al., 2008 [198]).

    The anterior limit of the premaxilla-maxilla suture on the dorsal surface of the rostrum typically coincides with the level of the pit or notch for the 4th dentary tooth. The anterior extent of this suture varies with respect to the posterior margin of the external naris. In most crocodylians, the anteriormost extent is posterior to the naris (Fig. 17G–I), but a large number of taxa (almost exclusively alligatorids) have an anteriorly positioned suture (Fig. 17A). By contrast to previous studies (Iijima & Kobayashi, 2019; Jouve, 2016), the derived condition is also recognised in some crocodylid species (Fig. 17D–F), such as *Crocodylus palaeindicus* (NHMUK 39795), *Crocodylus palustris* (NHMUK 1897.12.31.1), and variably in *Crocodylus porosus* (e.g. present in NHMUK 1852.12.9.2, absent in QM J47447).

50. Premaxilla, posterior extent of dorsal process: terminating level with or anterior to the third maxillary alveolus (0); extending posterior to third maxillary alveolus (1) (after Brochu, 1997a [145]).

    The length of each posterodorsal premaxillary process is characterised as originally formulated by Brochu (1997a). Long processes, exceeding the level of the 3rd maxillary alveoli, are commonly found in longirostrine crocodylians, including all putative "tomistomines" and "gavialoids" (Fig. 17B). By contrast, in *Bernissartia fagesii* (IRScNB 1538), as well as most crocodyloids and alligatoroids, the posterodorsal processes are short, at most reaching the level of the 3rd maxillary alveolus (Fig. 17A).

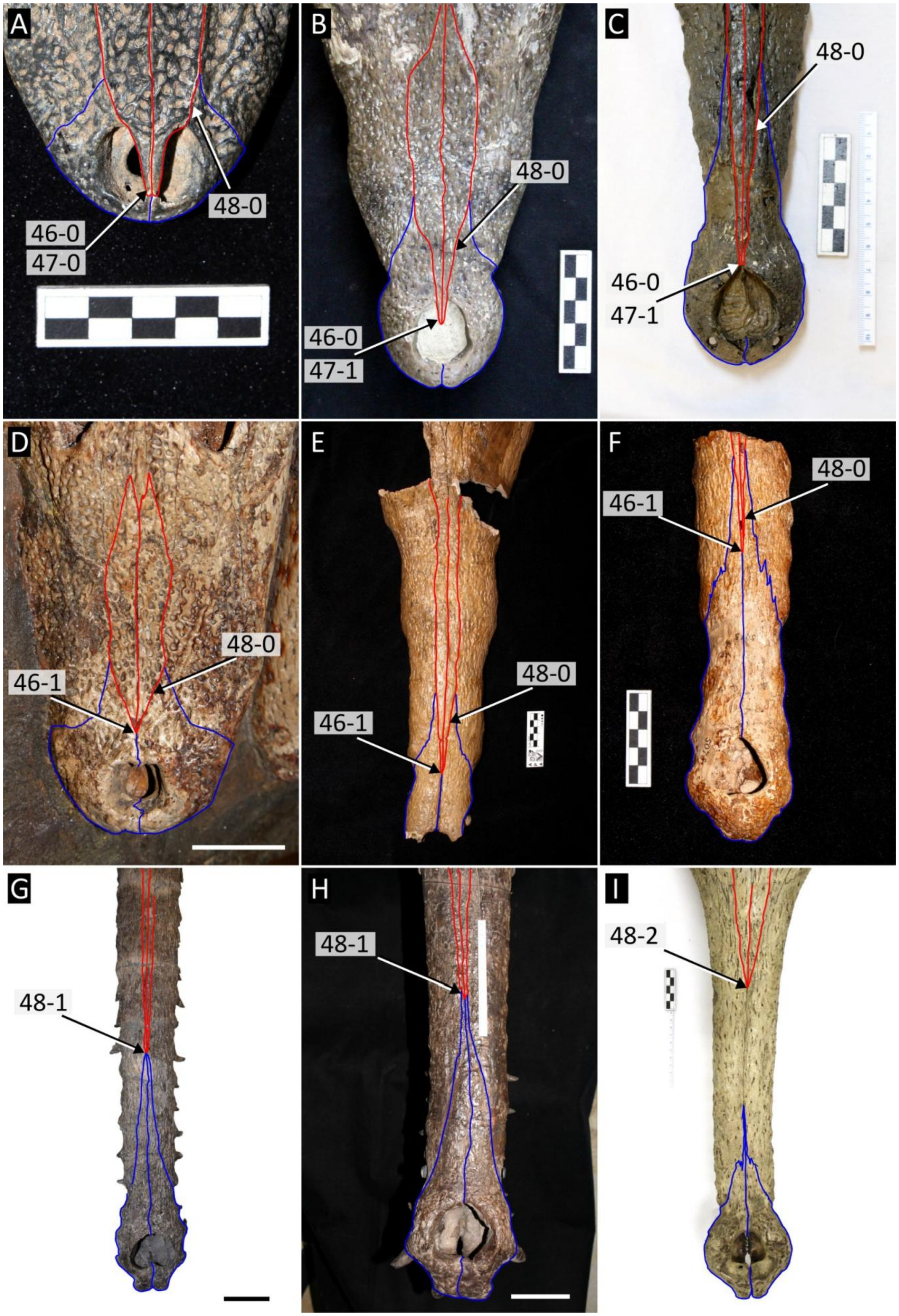

Figure 16: Morphology of the nasal-premaxilla suture. **A**, *Alligator mcgrewi* (AMNH F.A.M 7905); **B**, *'Crocodylus' affinis* (USNM 18171); **C**, *Kentisuchus spenceri* (NHMUK 38975); **D**, *Diplocynodon darwini* (HLMD-Me-7492); **E**, *Thecachampsa antiquus* (AMNH 5663); **F**, *Eogavialis africanum* (AMNH 5075); **G**, *Piscogavialis jugaliperforatus* (SMNK 1282 PAL); **H**, *Gryposuchus neoagaeus* (MLP 26-413); **I**, *Gavialis gangeticus* (NHMUK 1974.3009). Red = nasals, blue = premaxillae. All scale bars = 5 cm.

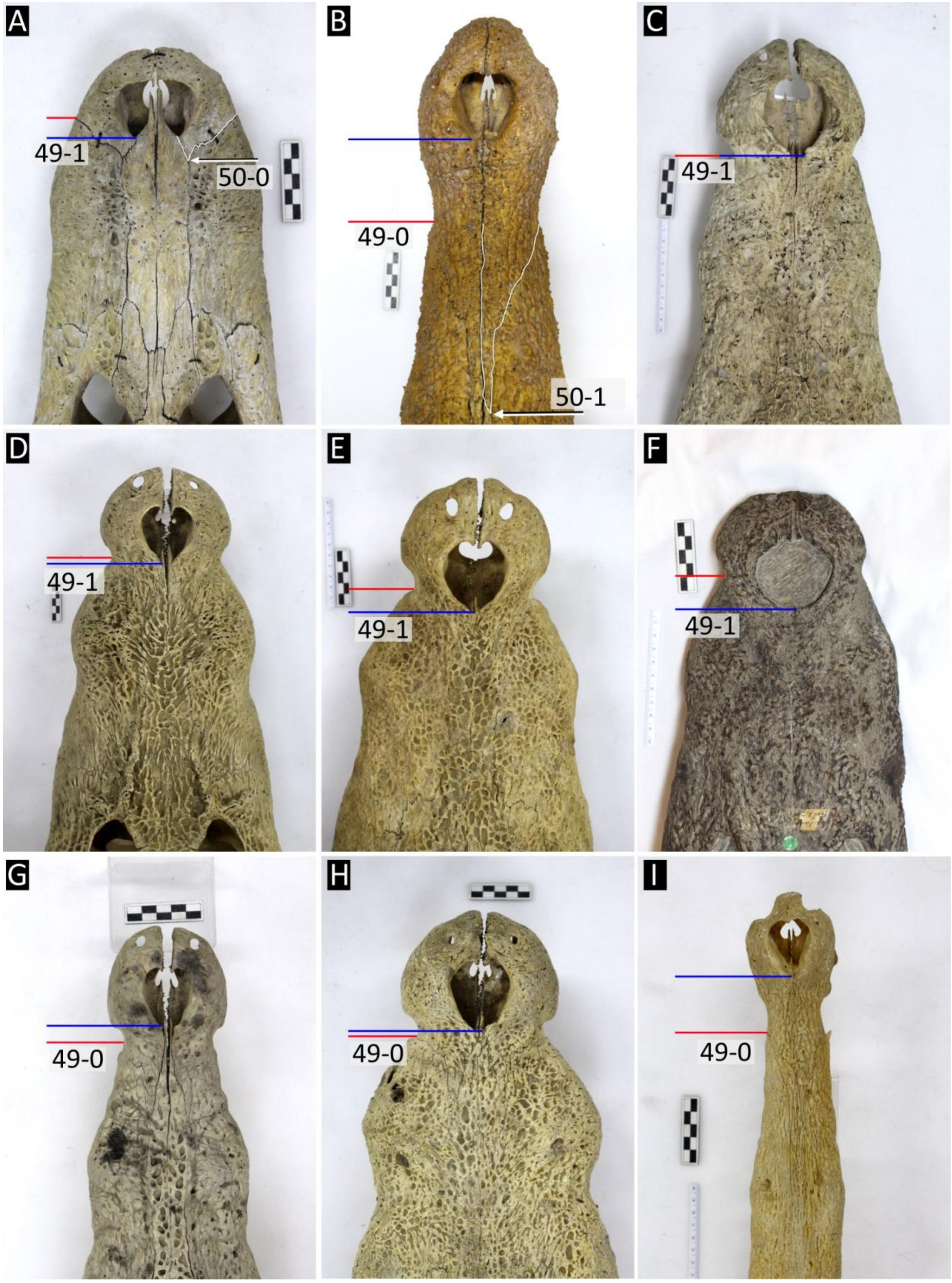

Figure 17: Dorsal view of the rostrum is crocodylian taxa showing variation in position of the premaxilla-maxilla suture (red line) relative to the posterior margin of the naris (blue line). **A**, *Alligator sinensis* (NHMUK X184); **B**, *Tomistoma schlegelii* (NHMUK 1894.2.21.1); **C**, *Crocodylus niloticus* (NHMUK 1934.6.3.1); **D**, *Crocodylus porosus* (NHMUK 1852.12.9.2); **E**, *Crocodylus palustris* (NHMUK 1897.12.31.1); **F**, *Crocodylus palaeindicus* (NHMUK 39795); **G**, *Crocodylus acutus* (NHMUK 1975.997); **H**, *Crocodylus moreletii* (NHMUK 1861.4.1.4); **I**, *Mecistops cataphractus* (NHMUK 1924.5.10.1). All scale bars = 5 cm.

Maxillae

51. Maxilla, linear array of pits (cecal recesses) on lateral margin of narial canal: absent (0); present (1) (after Brochu, 1997a [148]).

As discussed by Brochu (2000), the lateral walls of the narial canal (caviconchal recess) are lined with a series of pits in all extant species of *Crocodylus* (Fig. 18B), but are smooth in all other crocodylians (Fig. 18A). The presence or absence of cecal recesses can only be scored in well preserved and disarticulated maxillae or from CT scan data.

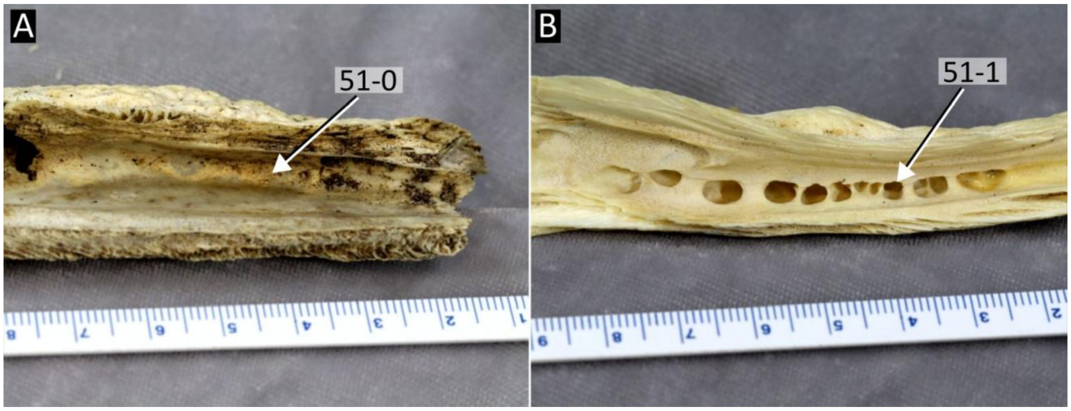

Figure 18: Medial view of the narial canal lateral wall in **A**, *Alligator mississippiensis* (UCMP 71672); **B**, *Crocodylus acutus* (UCMP 81699). Scale bars = cm.

52. Maxilla, posterior extent relative to anterior margin of postorbital bar: terminates anterior to the level of the postorbital bar (0); level with or posterior to the postorbital bar (1) (after Brochu, 2011 [105]).

The anatomical meaning of this character remains unchanged from Brochu (2011); however, the character has been rephrased to use the postorbital bar instead of the lower temporal bar as a more precise landmark for the posterior extent of the maxilla. In earlier studies (e.g. Brochu, 2011; Brochu et al., 2012; Jouve, 2016; Salas-Gismondi et al., 2019) the derived condition was only scored as present in *Hylaeochampsa vectiana* (Clark & Norell, 1992) (Fig. 19B), *Iharkutosuchus makadii* (Ösi et al., 2007), *Acynodon iberoccitanus* (Buscalioni et al., 1997), and *Acynodon adriaticus* (Delfino et al., 2008b). A number of additional taxa have been scored for the derived state in this study including *Portugalosuchus azenhae* (Mateus et al., 2019), *Gavialis gangeticus* (NHMUK 1974.3009) (Fig. 18D), *Gavialis lewisi* (YPM 3226), *Gavialis browni* (AMNH 6279), and *Trilophosuchus rackhami* (QM F16856). Although *Gavialis* and *Hylaeochampsa* share the derived condition, there are subtle differences in their morphologies. Whereas the maxilla is positioned more on the ventral side of the lower temporal bar in *Gavialis*, the maxilla is more laterally

exposed in *Hylaeochampsa*. However, this difference is too small, and not consistently found in enough taxa in the present analysis to warrant the addition of another character state here.

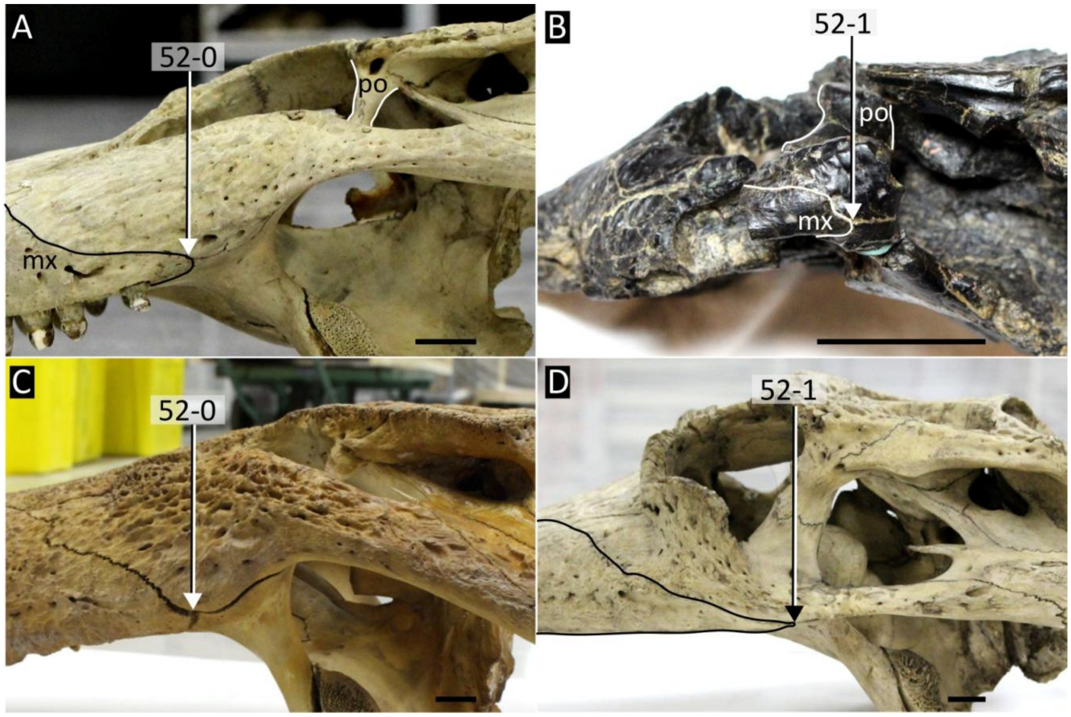

Figure 19: Variation in posterior extent of the maxilla. Lateral view of **A**, *Alligator mississippiensis* (NHMUK 1873.2.21.1); **B**, *Hylaeochampsa vectiana* (NHMUK PV R 177); **C**, *Tomistoma schlegelii* (NHMUK 1894.2.21.1); **D**, *Gavialis gangeticus* (NHMUK 1974.3009). Abbreviations: **po**, postorbital bar. All scale bars = 3 cm.

Lacrimal

53. Lacrimal, sutural contact with nasal: present (0); absent (1) (after Brochu, 1997a [93]).

54. Maxilla, posterior process in the lacrimal: absent (0); present (1) (after Brochu, 1997a [93]).

55. Maxilla, posterior process extends between lacrimal and prefrontal: absent (0); present (1) (after Brochu, 1997a [93]).

56. Maxilla, posterior process extends between nasal and lacrimal: absent (0); present (1) (after Brochu, 1997a [93]; Jouve et al., 2008 [93]; Jouve, 2016 [93]).

Characters 53–56 are derived from Character 93 in Brochu (1997b) and later modifications to the character (Jouve et al., 2008 [93]; Jouve, 2016 [93]). Brochu (1997b) originally formulated the character as follows:

"*Lacrimal makes broad contact with nasal; no posterior process of maxilla (0), or maxilla with posterior process within lacrimal (1), or maxilla with posterior process between lacrimal and prefrontal (2)*"

Jouve (2016) added a fourth character state, which described a process "*between the lacrimal and nasal*".

As originally worded, this character suggests that contact between the nasal and lacrimal, as well as a posterior process of the maxilla in the lacrimal are mutually exclusive. However, both conditions are commonly present in crocodylians. For example in all datasets examined here, *Caiman* are characterised as sending a posterior process of the maxilla into the lacrimal (Brochu, 1999; Brochu et al., 2012; Cidade et al., 2017; Iijima & Kobayashi, 2019; Jouve, 2016; Jouve et al., 2008; Lee & Yates, 2018); however, most *Caiman* species also exhibit contact between the lacrimal and nasal (Fig. 20C). The choice to include *Caiman* under character state 1 in previous studies, is probably based on the short length of the nasal-lacrimal contact; however, the length of the nasal-lacrimal suture in some *Caiman* species can be equal to that of some *Crocodylus* species, which have a broad lacrimal nasal contact (Fig. 20A). In addition, there are some taxa which lack a nasal-lacrimal sutural contact, but still retain a posterior maxillary process in the lacrimal (e.g. *Purussaurus neivensis*, UCMP 39704). In this study, the presence of a lacrimal-nasal contact is not considered homologous to the development of any process in or between the lacrimal, prefrontal, or nasal. As such, a new character has been formulated which describes the presence or absence of a lacrimal-nasal sutural contact (Character 53). Additional issues concern the homology of the posterior processes between the lacrimal, prefrontal and nasals that are implied in the original character. Some taxa, such as '*Caiman* cf. *lutescens*' (UCMP 39978) and the 'Glen Rose form' (USNM 22039), possess both a posterior process of the maxilla in the lacrimal and a posterior process between the lacrimal and prefrontal. As a result, these features have been discretised in separate characters here. Furthermore, the posterior maxillary process between the lacrimal and nasal is not homologous to the posterior maxillary process in the lacrimal, because both are present in *Thecachampsa sericodon* (Fig. 20F).

57. Lacrimal, mediolateral width in relation to prefrontal: equal to or greater than twice the maximum prefrontal width (0); less than twice the maximum prefrontal width (1) (after Jouve, 2016 [242]).

This character was rephrased to improve repeatability of scoring from "*lacrimal nearly twice wider (0) or nearly as wide as the prefrontal (1)*" in Jouve (2016). The mediolateral widths of the prefrontal and lacrimal are approximately equidimensional in most crocodylians, e.g. *Crocodylus porosus* (Fig. 20A) and *Alligator mississippiensis* (Fig. 20B). Extremely widened lacrimals, usually greater than twice the width of the prefrontals, are present in most extant caimanines (Fig.

20C), such as *Melanosuchus niger* (NHMUK 45.8.25.125), *Caiman yacare* (AMNH 97300), and *Paleosuchus trigonatus* (NHMUK 1868.10.8.1), as well as some "tomistomines" such as *Thecachampsa sericodon* (USNM 25243). By contrast to previous studies (e.g. Iijima & Kobayashi, 2019; Jouve, 2016), the width of the lacrimals in *Brachychampsa montana* (UCMP 133901, Fig. 20D), *Tomistoma schlegelii* (1894.2.21.1), *Tomistoma lusitanica* (Antunes, 1961), and *Moroccosuchus zennaroi* (Jouve et al., 2015), is not found to be significantly greater than that of the prefrontals.

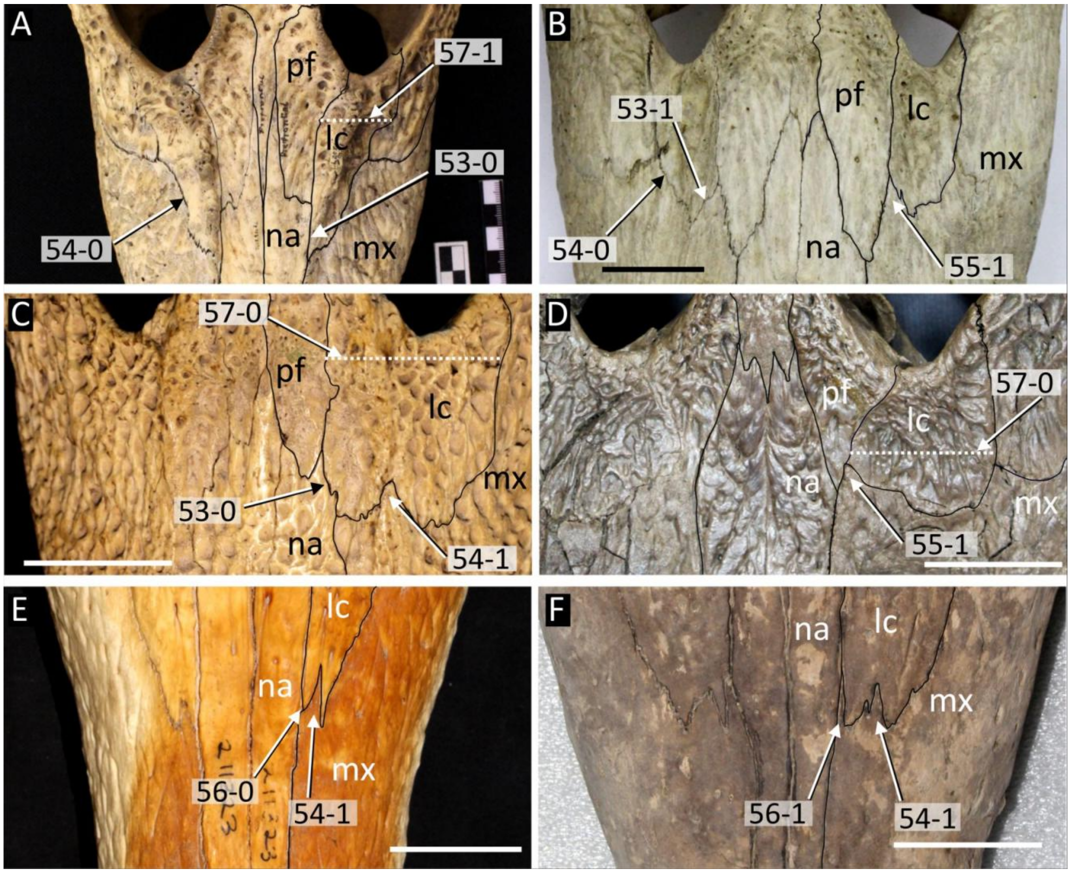

Figure 20: Dorsal view showing sutural relationships of the rostrum in selected crocodylian taxa. **A**, *Crocodylus porosus* (QM J47447); **B**, *Alligator mississippiensis* (NHMUK 1873.2.21.1); **C**, *Caiman yacare* (MACN uncatalogued); **D**, *Brachychampsa montana* (UCMP 133901); **E**, *Tomistoma schlegelii* (USNM 211323); **F**, *Thecachampsa sericodon* (USNM 25243). Abbreviations: **lc**, lacrimal; **mx**, maxilla; **na**, nasal; **pf**, prefrontal. All scale bars = 5 cm.

58. Lacrimal, anteroposterior length relative to that of prefrontal: longer (0); equal in length (1); shorter (2) (after Norell, 1988 [7]; Brochu, 1997a [117]) (ORDERED).

This character has been restructured and rephrased from Brochu (1997b) to enable the character to be ordered. In most crocodylians, the lacrimal is much longer than the prefrontal (Fig. 21G), some taxa (mostly alligatorids) exhibit a clearly shorter lacrimal than the prefrontal (Fig. 21I), and

a small number have a lacrimal and prefrontal that are sub-equal in length (Fig. 21H). The latter condition was previously found only in *Borealosuchus* species, such as *B. formidabilis* (Erickson, 1976), *B. acutidentatus* (Sternberg, 1932), and *B. wilsoni* (Mook, 1959) (Brochu, 1997a; Brochu et al., 2012). This intermediate condition is newly recognised in a number of additional species, such as *Kentisuchus spenceri* (NHMUK 38975), *Baru wickeni* (QM 16822), and *Acresuchus pachytempralis* (UFAC 2507).

Frontal-Prefrontal

59. Prefrontals, sutural contacts (at maturity): separated by frontal and nasals at maturity (0); prefrontals meet medially (1) (after Norell, 1988 [27]; Brochu, 1997a [100]).

    Whereas the prefrontals are separated by the frontal and nasals in most crocodylians (Fig. 21A), they are sutured in some caimanine taxa, blocking the anterior frontal process from the nasals (Fig. 21C). For example, *Caiman yacare* exhibits inter-prefrontal contact consistently at maturity (AMNH 97300, MACN uncatalogued, FMNH 9141) (Medem, 1960). By contrast it appears to occur variably in *Caiman latirostris* (present in NHMUK 86.10.4.2, absent in FMNH 9713, MACN V 1420).

60. Frontal, anterior process morphology: forms an acute, 'v' shape that extends anteriorly into posterior margins of nasals (0); forms broad sutural contact with the nasals or prefrontals (1) (after Brochu, 2011 [131]; Salas-Gismondi et al., 2015 [131]).

    The anterior tip of the frontal process forms an acute point in most crocodylians, regardless of whether it contacts the nasals. By contrast, a number of crocodylians exhibit a broader (sometimes undulating) frontal processes. The modification to character wording by Salas-Gismondi et al. (2015) is followed here, which recognises that the frontal might or might not contact the prefrontals. The derived condition is present in several alligatoroids, including *Brachychampsa montana* (Fig. 21D), *Mourasuchus atopus* (Fig. 21E), *Purussaurus neivensis* (Fig. 21C), as well as some species of *Mekosuchus*, e.g. *M. sanderi* (QM F31166).

61. Frontal, position of tip of anterior process relative to anterior tip of prefrontal: posterior or at the same level (0); anterior (1) (after Jouve, 2004 [172]; Jouve et al., 2008 [171])

62. Frontal, position of tip of anterior process relative to anterior orbital margin: anterior (0); level with or posterior (1) (after Jouve, 2004 [178]; Jouve et al., 2008 [175]).

63. Jugal, anterior extent relative to anterior tip of frontal: anterior to or level with frontal (0): posterior to frontal (1) (after Jouve, 2004 [177]; Jouve et al., 2008 [174]; Jouve, 2016 [174]).

Characters 61–63 were introduced by Jouve (2004), and describe variation in the anterior extent of the frontal relative to the prefrontal, orbit, and jugal respectively. Characters 61 and 63 have been modified by the removal of a character state. Character 61 was originally formulated with an additional state in which the frontal reached the same level as the prefrontal (Jouve, 2016). This condition is not practical to score as the frontal rarely lies precisely at the same level as the prefrontal. For example, *Euthecodon arambourgi* (MNHN ZEL 001) was scored for this condition by Jouve (2016) but the frontal extends anterior to the prefrontal in that taxon.

As originally formulated by Jouve (2004), Character 63 had an additional character state which described a condition whereby the jugal does not extend beyond the anterior margin of the orbit. This condition was only scored in *Iharkutosuchus makadii* and *Hylaeochampsa vectiana* in a more recent iteration of this data matrix (Jouve, 2016). This state has been removed here because the condition is absent in *Hylaeochampsa* (NHMUK R177, Clark & Norell, 1992) rendering it as an uninformative autapomorphy of *Iharkutosuchus*. As such *Iharkutouchus* is scored as 63-1 here along with other crocodylian taxa.

It might appear that these characters describe the same anatomical feature, namely the length of the anterior frontal process, and that taxa with a 'long' frontal process might receive the same scores for each of these characters i.e. 61-1, 62-0, and 63-1. However, these characters are scored with different combinations in several taxa, reflecting their independence. For example many crocodylians have a frontal process that exceeds the anterior margin of the orbits (62-0). This is the case in *Brachychampsa montana* and *Gavialis gangeticus*; however, whereas the frontal process is anterior to the prefrontal tip in *Gavialis* (61-1) (Fig. 21F), it is posterior to the prefrontal tip in Brachychampsa (61-0) (Fig. 21D). Similarly, if the frontal exceeds the anterior margin of the prefrontal, it does not necessarily exceed the jugal anterior tip. This is the case in *Navajosuchus mooki* (AMNH 5186) and *Gavialosuchus eggenburgensis* (NHMUK PV R797), which share an anterior frontal tip that extends beyond the level of the prefrontal (61-1), but not the jugal (63-0).

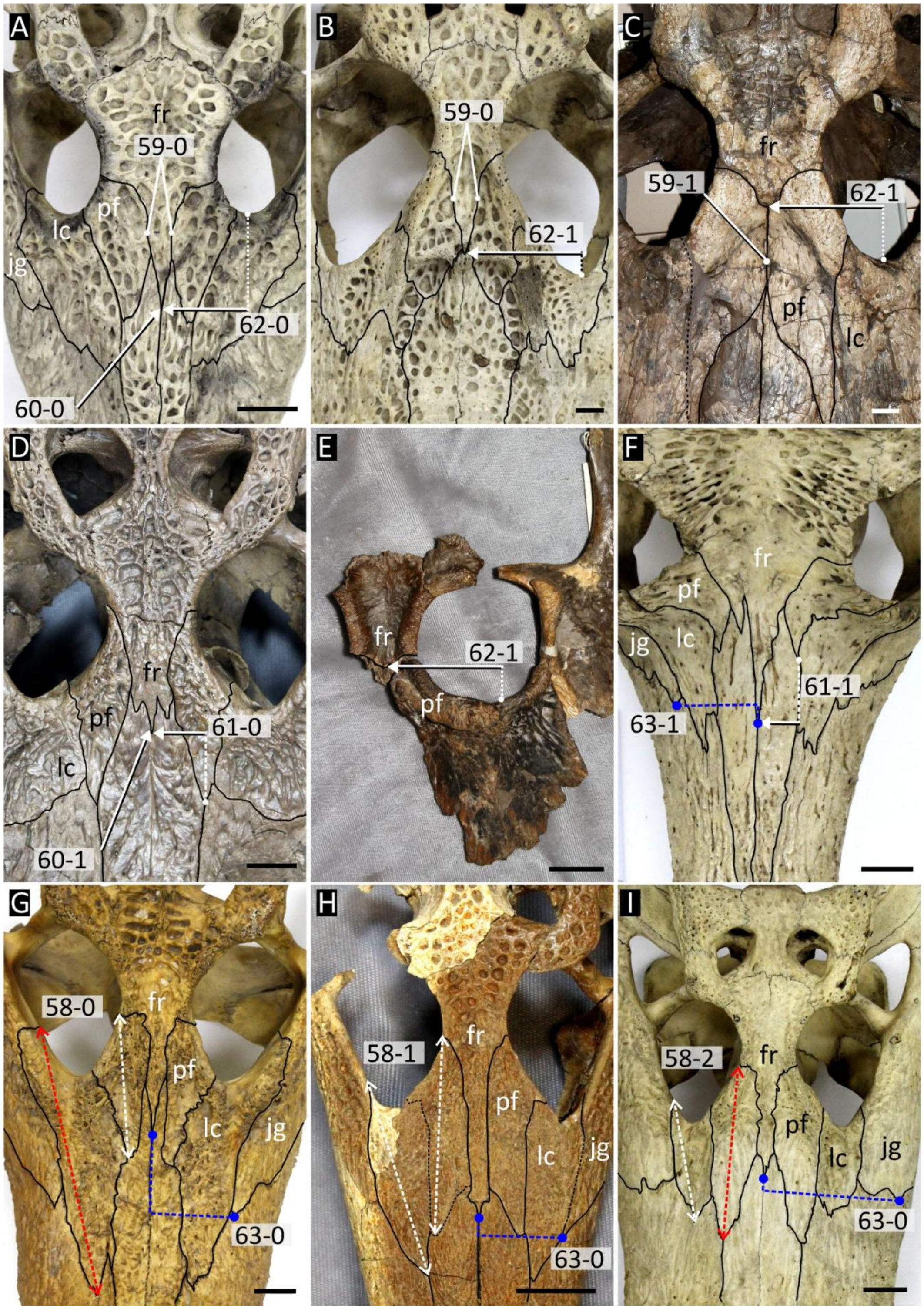

Figure 21: Preorbital sutural relationships in selected crocodylian taxa. **A**, *Crocodylus acutus* (NHMUK 1975.997); **B**, *Melanosuchus niger* (NHMUK 45.8.25.125); **C**, *Purussaurus neivensis* (UCMP 39704); **D**, *Brachychampsa montana* (UCMP 133901); **E**, *Mourasuchus atopus* (UCMP 38012); **F**, *Gavialis gangeticus* (NHMUK 1974.3009); **G**, *Tomistoma schlegelii* (NHMUK 1894.2.21.1) **H**, *Borealosuchus sternbergii* (UCMP 126099); **I**, *Alligator mississippiensis* (NHMUK 1873.2.21.1). Abbreviations: **fr**, frontal; **jg**, jugal; **lc**, lacrimal; **pf**, prefrontal. All scale bars = 2 cm.

64. Prefrontal, linear sulcus adjacent to medial orbital margin: absent (0); present (1) (new character, after Delfino et al., 2005)

Delfino et al. (2005) described a “step-like” structure on the prefrontal, forming the medial margin of the orbit in *Eosuchus lerichei* (Fig. 22C). This step is the result of a linear sulcus which runs adjacent to the medial orbital margin, and this feature is newly recognised here in *Eosuchus minor* (USNM 321933) and *Thoracosaurus isorhynchus* (MNHN.F.MTA 61) (Fig. 22B).

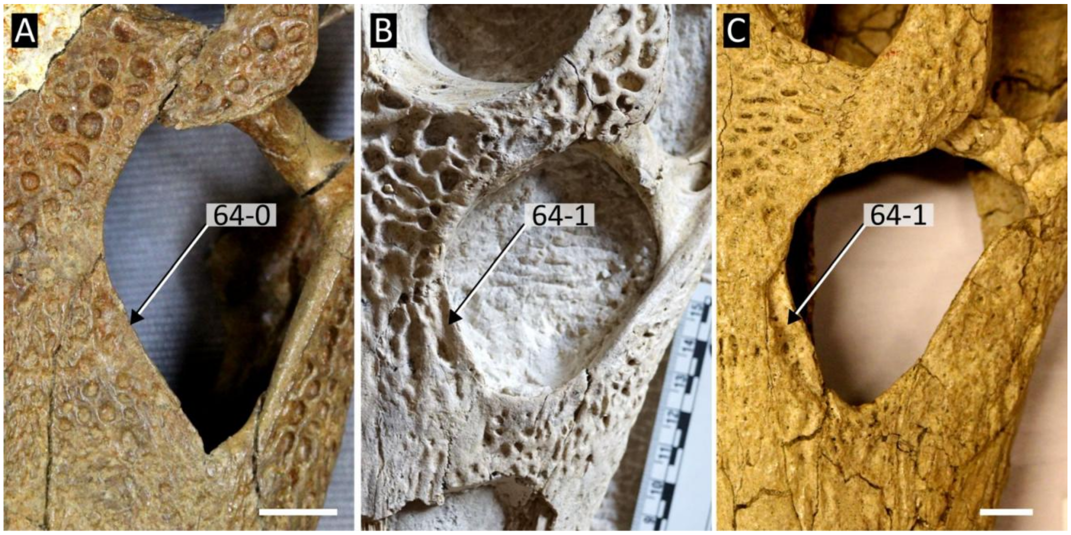

Figure 22: Dorsal view of the orbit in **A**, *Borealosuchus sternbergii* (UCMP 126099); **B**, *Thoracosaurus isorhynchus* (MNHN.F.MTA 61); **C**, *Eosuchus lerichei* (IRSNB R 49). Scale bar in A and C = 1 cm, B = cm.

65. Jugal, suture with lacrimal: long, widely separating maxilla from orbital margin (0); point contact, narrowly separating maxilla from orbital margin (1); jugal-lacrimal contact absent, maxilla contributes to orbital margin (2) (after Willis 1997, 2001; Lee and Yates 2018 [38]) (ORDERED).

In nearly all crocodylians, the maxilla is distantly separated from the orbital margin by the jugal and lacrimal (Fig. 23B). By contrast, the maxillae of *Mekosuchus inexpectatus* (MNHN NCP 06), *Mekosuchus sanderi* (QM F31188) (Fig. 23D), and *Mekosuchus whitehunterensis* (QM F31051) form a small portion of the lateral orbital margin (Willis, 2001). The orbital contribution of the maxilla can be challenging to recognise in these taxa due to the nature of their preservation as isolated elements; however, the orbital contribution is evidenced by a short, suture-free length of the maxillary dorsal outline (Fig. 23D). *Trilophosuchus rackhami* (QM F16856) exhibits an intermediate condition (65-1), in which the jugal and lacrimal have a point contact that narrowly separates the maxilla from the orbit (Willis, 1997) (Fig. 23C). As such, this character is ordered here.

66. Prefrontal pillar morphology: dorsal half of pillar narrow, less than twice minimum anteroposterior

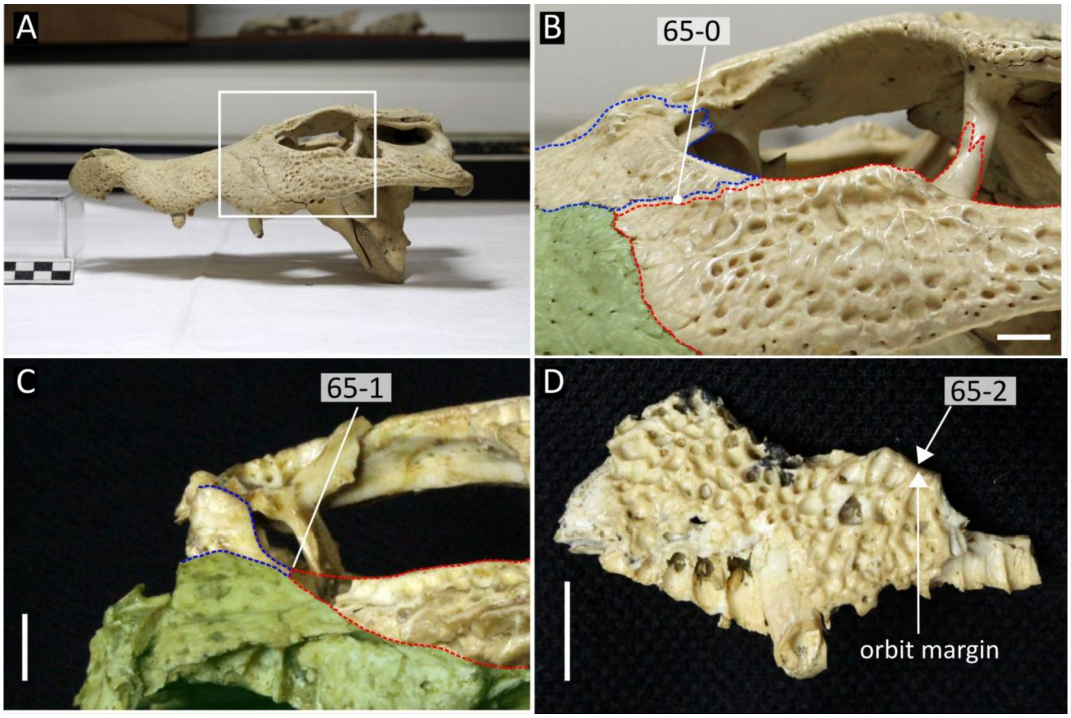

Figure 23: Variation in extent of jugal-lacrimal contact in crocodylian taxa. **A**–**B**, *Osteolaemus tetraspis* (NHMUK 1862.6.30.5); **C**, *Trilophosuchus rackhami* (QM F16856); **D**, *Mekosuchus sanderi* (QMF 31188), left maxilla in lateral view showing maxilla contribution to orbital margin. Blue and red dashed lines outline the lacrimal and jugal bones respectively. Green shading shows position of the maxilla. All scale bars = 1 cm.

length (0); equal to or greater than twice minimum anteroposterior length (1) (after Norell, 1988 [41]; Brochu, 1997a [137]).

In most crocodylians, the dorsal half of the prefrontal pillar is anteroposteriorly expanded, such that in transverse section the pillar appears to be flared (66-1). This is visible in CT scan data (Brochu, 1999, fig.53B) and sometimes externally too (Fig. 24B). This character has received modification to the original wording of Brochu (1997b) by quantifying the degree of expansion to improve the consistency of scoring. Previous studies have traditionally scored "gavialoids" (e.g. *Gavialis gangeticus*, Fig. 24A) as having the narrow condition (i.e. 66-0). Here, the flared condition is newly recognised in several "gavialoids", inluding *Eogavialis africanum* (YPM 6263), and *Eosuchus lerichei* (IRScNB R49).

67. Prefrontal pillar, morphology of medial processes, long axis orientation: dorsoventrally (0); anteroposteriorly (1) (after Brochu, 1997a [136]).

The medial surface of each prefrontal pillar contacts its counterpart via a distinct ventromedial projection, referred to as a medial process. These processes are seldom preserved and often obscured by matrix. When preserved, the articular facet of the medial process is usually anteroposteriorly expanded (67-1) (Fig. 24C, F, I). Less commonly, the medial processes are dorsoventrally expanded

(67-0) (Fig. 24A, B, G, H). Following previous studies, the anteroposteriorly expanded condition has been observed in all crocodyloids (where preserved), and extant species of *Alligator*. By contrast to scores in previous studies (e.g. Brochu, 1999; Brochu et al., 2012), *Tomistoma schlegelii* (Fig. 24B) and some caimanines (e.g. *Caiman yacare*, Fig. 24G) and *Paleosuchus trigonatus*, Fig. 24H), are herein found to exhibit the dorsoventrally expanded condition. The condition in *Hylaeochampsa vectiana* is also changed from being dorsoventrally expanded to unknown, as this portion of anatomy is not well enough preserved (NHMUK R177).

68. Prefrontal pillar internal morphology: solid (0); with pneumatic recess (1) (state 1 is synonymous with the prefrontal recess of Witmer, 1997) (after Brochu, 1997a [99])

In most crocodylians, the prefrontal pillar is a solid vertical bar that descends from the skull roof to contact the palatines and pterygoids. In *Alligator mississippiensis* the prefrontal pillar is hollow, as evident from CT data (Brochu, 1999, fig.53). However, CT data is not always required to score this character, as the hollow condition is associated with a discernible inflation of the prefrontal pillar that is visible externally (Fig. 24I). For example, the fossil alligatorid *Alligator mcgrewi* is scored as having a pneumatic recess (68-1) based on the inflation of the prefrontal pillars in AMNH FAM 8700.

69. Prefrontal pillar, morphology of medial process at base of pillar: wide (0); constricted (1) (after Brochu, 1997a [138]).

The ventral margin of the prefrontal medial process bears a notch in most crocodyloids, giving a dorsoventrally constricted appearance to the medial process (69-1) (Fig. 24F). This constriction is absent in all alligatoroids and gavialoids, where preserved (69-0). Minor changes have been made to character scores here, such as the recognition of the constricted condition in *Borealosuchus formidabilis* (Erickson, 1976, fig.10) ('wide' according to Brochu et al. (2012)), and *Borealosuchus sternbergii* (Fig. 24D) (unknown according to Brochu et al. (2012)).

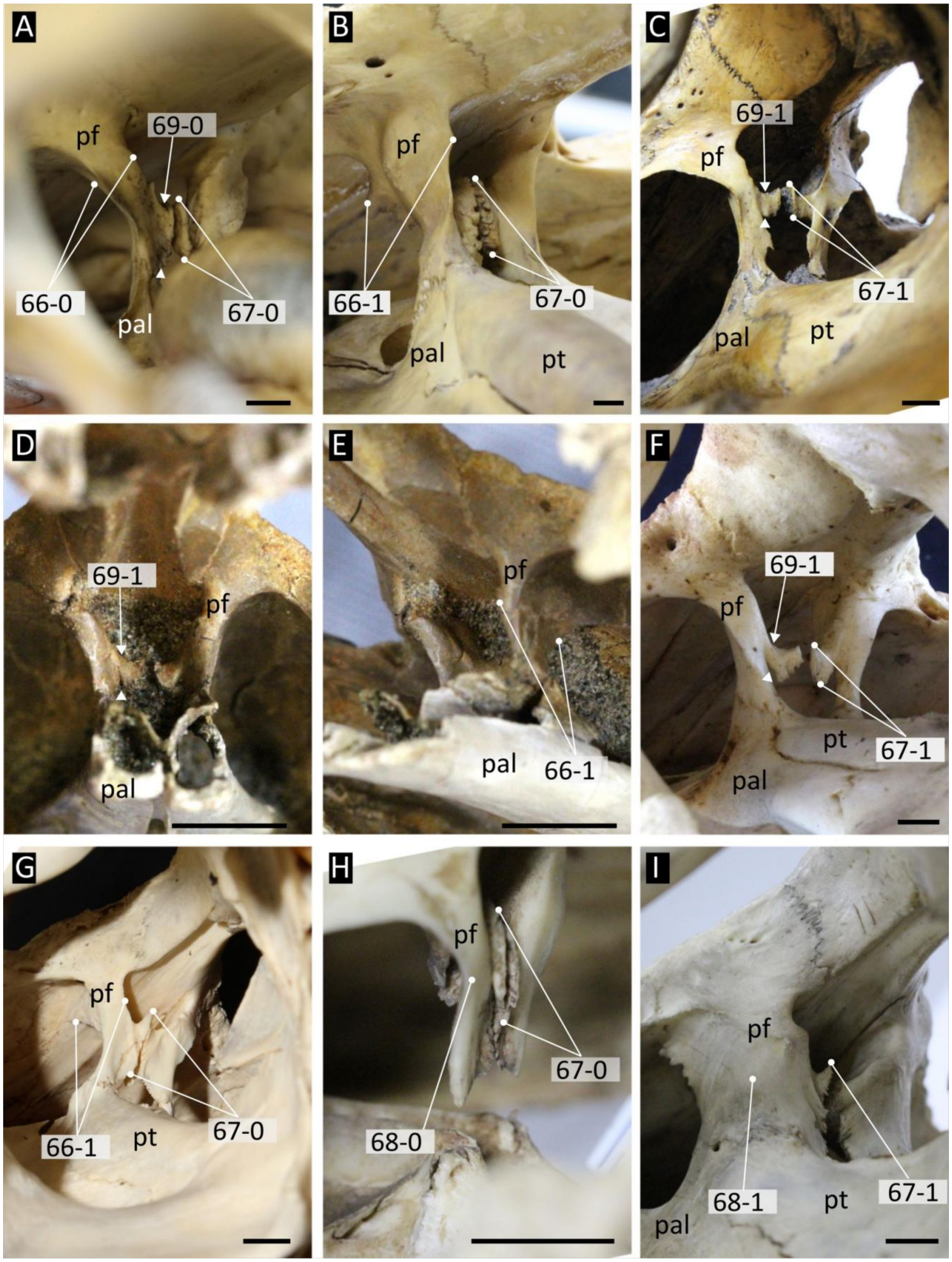

Figure 24: Anteromedial view of the prefrontal pillars in selected crocodylians. **A**, *Gavialis gangeticus* (NHMUK 61.4.1.2); **B**, *Tomistoma schlegelii* (NHMUK 1894.2.21.1) **C**, *Voay robustus* (NHMUK R 36685); **D**, *Borealosuchus sternbergii* (UCMP 126099), anterior view; **E**, *Borealosuchus sterbergii* (UCMP 126099), anteromedial view; **F**, *Crocodylus acutus* (FMNH 69884); **G**, *Caiman yacare* (AMNH 97300); **H**, *Paleosuchus trigonatus* (NHMUK 1868.10.8.1); **I**, *Alligator mississippiensis* (NHMUK 1873.2.21.1). Abbreviations: **pal**, palatine; **pf**, prefrontal; **pt**, pterygoid. All scale bars = 1 cm.

70. Frontal, ornamentation, midsagittal crest on fused frontals: absent (0); or present (1) (after Brochu and Storrs, 2012 [188]).

In the dataset of Brochu and Storrs (2012) a sagittal interorbital ridge on the frontal is known only in *Crocodylus siamensis* (Fig. 25B); however, it is recognised as a relatively common ornament in Eusuchia here. A sagittal frontal ridge is present in several non-crocodylian eusuchians such as *Wannchampsus kirkpachi* (Adams, 2014), and *Theriosuchus pusillus* (NHMUK 48270), as well as mekosuchines such as *Trilophosuchus rackhami* (QM F16856), *Mekosuchus sanderi* (QM F31188), and *Mekosuchus inexpectatus* (MNHN NCP 06). A frontal ridge is considered diagnostic of *Crocodylus siamensis* among extant *Crocodylus* species (Delfino & De Vos, 2010). This was observed in all *Crocodylus siamensis* specimens studied here (NHMUK 1921.4.1.168, NHMUK 1921.4.1.172; NHMUK 1931.12.6.6); however, a frontal ridge is also variably present in *Crocodylus niloticus* (present in NHMUK 1934.6.3.1; absent in NHMUK 1864.6.5.53).

71. Orbit, ornamentation, protuberance on the frontal-prefrontal suture intersection with the orbit: absent (0); or present (1) (new character, based on personal observation).

The medial orbital margins are slightly upturned in almost all crocodylians. In addition to this, some taxa exhibit a rounded protuberance at the intersection of the frontal-prefrontal suture with the orbital margin (Fig. 25B). This condition is observed in all *Crocodylus siamensis* specimens examined here (NHMUK 1921.4.1.168, NHMUK 1921.4.1.172; NHMUK 1931.12.6.6) and occurs variably in *Crocodylus palustris* (e.g. absent in NHMUK 1868.4.9.11, present in NHMUK 1861.4.1.5), *Crocodylus porosus* (e.g. absent in NHMUK 1852.12.9.2, present in NHMUK 67.4.2.188), and *Crocodylus palaeindicus* (e.g. absent in NHMUK 39799, present in NHMUK 39795).

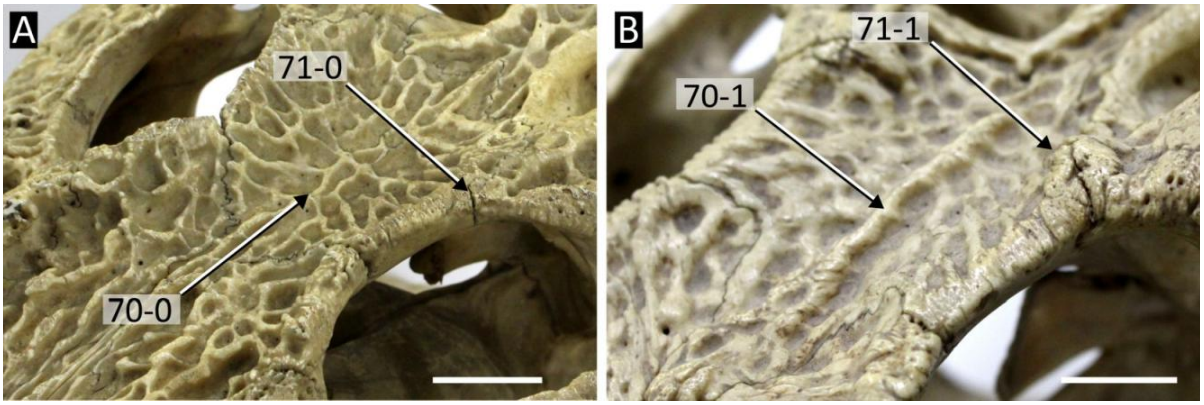

Figure 25: Dorsolateral view of the frontal in **A**, *Crocodylus porosus* (NHMUK 1852.12.9.2); **B**, *Crocodylus siamensis* (NHMUK 1921.4.1.168). All scale bars = 2 cm.

72. Orbit, dorsomedial margin: flush with skull surface (0); upturned (1); projecting into orbit (2) (after Brochu, 1997a [103]) (ORDERED).

This character has received no modifications to character states, but is newly ordered and several scores have been changed. The dorsomedial margins of the orbit are slightly upturned (72-1) in most crocodylians (Fig. 26C). Among other eusuchians, this condition is here considered intermediate between the flush orbital margins exhibited in *Borealosuchus* (72-0) and the telescoped orbits that characterise *Gavialis gangeticus* (72-2) (Fig. 26F). A comparison between the character scores in different datasets suggests the distinction between upturned (72-1) and telescopic (72-2) orbital margins is ambiguous. Fro example, according to Brochu et al. (2012), the late Paleogene gavialoid, *Eogavialis africanum*, has telescoped orbits. However, following Salas-Gismondi et al. (2019), the orbital margins of *Eogavialis africanum* are here considered upturned (Fig. 26D), reminiscent of the condition in juvenile individuals of *Gavialis* (NHMUK 96.7.7.4, NHMUK 96.7.7.4.2). The upturned condition is shared by some South American Miocene gavialoids, such as *Ikanogavialis gameroi* (Sill, 1970) and *Gryposuchus pachakamue* (Salas-Gismondi et al., 2016) and might represent an incipient telescoped condition, leading to the fully everted orbital margins of *Gavialis* and all other *Gryposuchus* species. Telescoped orbits are newly recognised in several species of the giant caimanine *Mourasuchus*, e.g. *M. atopus* (Fig. 26G–H). Previously, these taxa were scored as having upturned orbits (Cidade et al., 2017; Souza-Filho et al., 2019); however, the orbital margins are everted from the cranial surface in a similar style to *Gavialis*, although not to the same degree.

73. Orbit, position of posterior margin (measured at the level of the postorbital-frontal suture) relative to posterior margin of suborbital fenestra: posterior to or at the same level (0); anterior (1) (after Jouve, 2004 [195]; Jouve et al., 2008 [186]; Salas-Gismondi et al., 2015 [195]).

The anatomical meaning of this character is consistent with earlier studies; however, there are several score changes here relative to earlier studies (e.g. Jouve, 2016). This character was assessed by examining crania in dorsal view and noting the relative positions of the posterior margin of the suborbital fenestra and orbit (at the level of the frontal-postorbital suture). In many "gavialoids", e.g. *Gavialis gangeticus* (Fig. 27A), the posterior margin of the suborbital fenestra cannot be observed through the orbits, as it is positioned further posteriorly (73-1). By contrast, in all extant alligatorids and most crocodylids, the posterior margin of the suborbital fenestra is positioned anterior to the posterior margin of the orbits, such that it is visible through the orbits in dorsal view (Fig. 27B). By contrast with scores in Jouve (2016), several "tomistomines" share the same condition as *Gavialis*, including *Tomistoma schlegelii* (NHMUK 1894.2.21.1) and *Tomistoma cairense* (SMNS 10575, SMNS 50739).

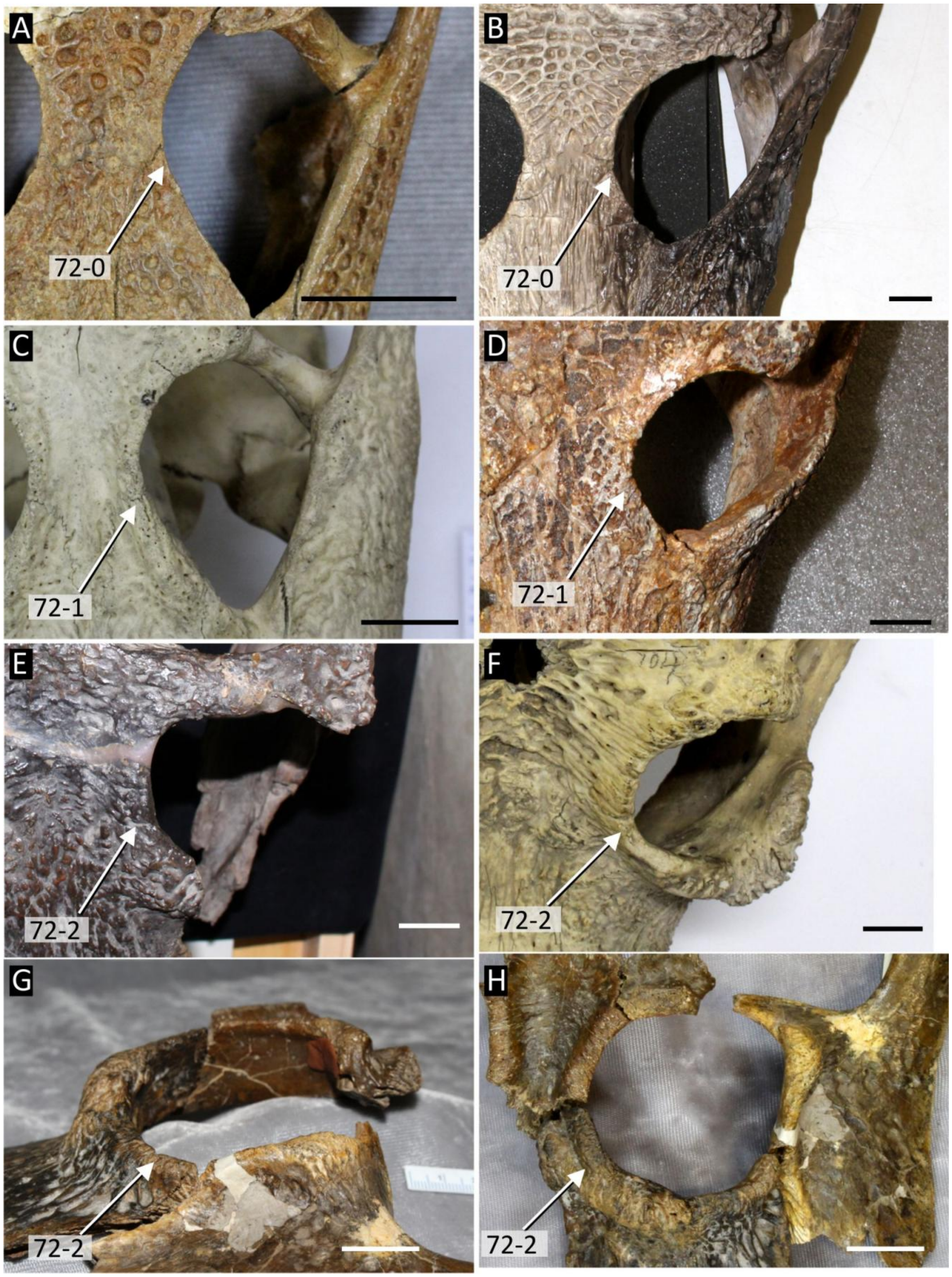

Figure 26: Variation in degree of upturning of orbital margins. **A**, *Borealosuchus sternbergii* (UCMP 126099); **B**, *Piscogavialis jugaliperforations* (SMNK 1282 PAL); **C**, *Alligator mississippiensis* (NHMUK 1873.2.21.1); **D**, *Eogavialis africanum* (YPM 6263); **E,** *Gryposuchus neogaeus* (MLP 26-413); **F**, *Gavialis gangeticus* (NHMUK 704); **G**-**H**, *Mourasuchus atopus* (UCMP 38012). All scale bars = 2 cm.

74. Orbit, lateral-most margin relative to the lateral margin of the maxilla at the level of alveoli 3–6: lateral (0); level with or medial (1) (after Jouve, 2004 [206]; Jouve, 2016 [208]).

Following Jouve (2004), this character essentially describes the width of the rostrum, broadly distinguishing brevirostrine crocodylians from longirostrines. For example, whereas in all "tomistomines" and "gavialoids" the orbit is positioned lateral to the level of maxillary alveoli 3–6 (Fig. 27A), it is medial in most alligatoroids and crocodyloids (Fig. 27B). Not all taxa exhibiting the plesiomorphic condition are longirostrines, this morphology also occurs in *Caiman crocodilus* (FMNH 69812), *Caiman yacare* (AMNH 97300), *Borealosuchus* (e.g. *B. sternbergii*, USNM 6533), '*Crocodylus*' *affinis* (USNM 18171), and *Asiatosuchus depressifrons* (IRScNB R251).

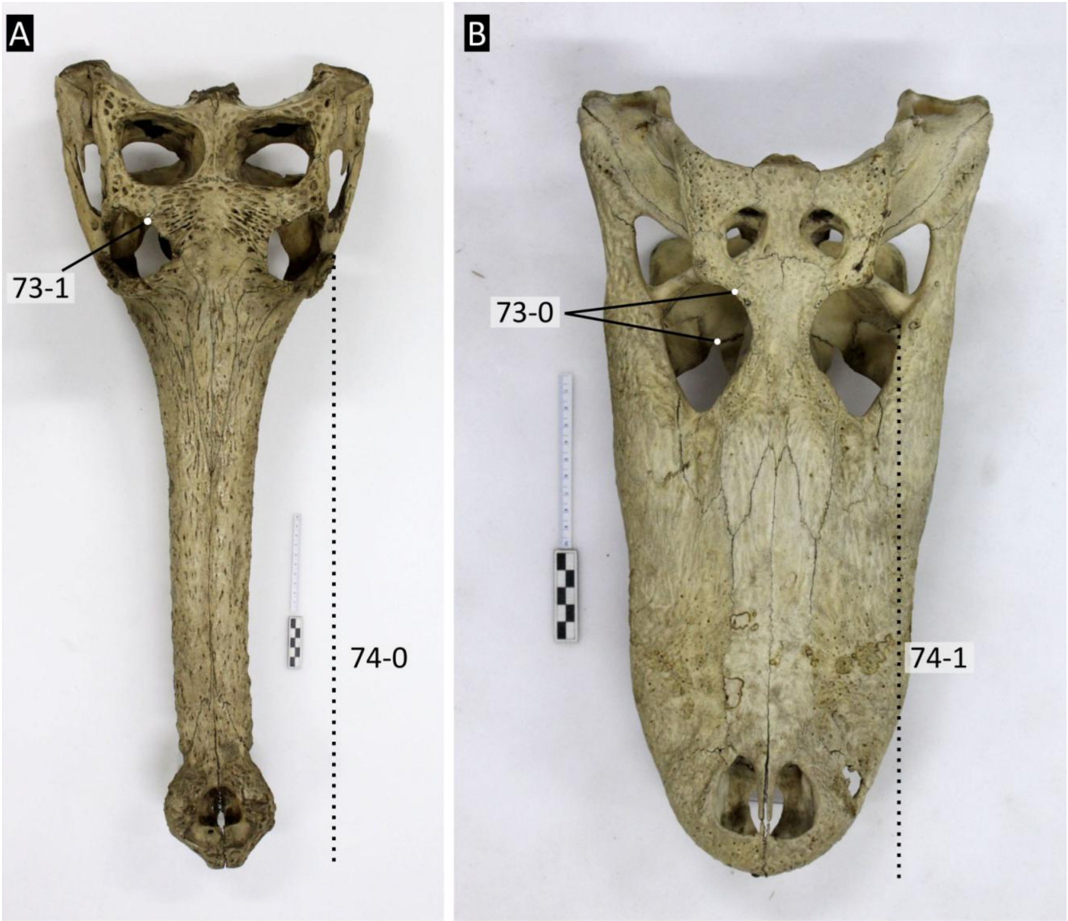

Figure 27: Dorsal view of the cranium of **A**, *Gavialis gangeticus* (NHMUK 1974.3009); **B**, *Alligator mississippiensis* (NHMUK 1873.2.21.1). Scale bars = cm.

75. Frontoparietal suture, intersection with supratemporal fenestra (at maturity): deep intersection, postorbital-parietal suture not exposed on skull table (0); frontoparietal suture incipiently contacts supratemporal fenestra, postorbital-parietal suture slightly visible (1); frontoparietal suture does not intersect supratemporal fenestra, postorbital-parietal contact fully exposed on skull table (2)

(after Brochu, 1997a [81]) (ORDERED).

Character state definitions for this character are unchanged from Brochu (1997b). Minor character score changes have been made, and the character is newly ordered. The frontoparietal suture runs transversely across the anterior cranial table in all eusuchians; however, its proximity to the supratemporal fenestrae varies. In most crocodylians, the suture does not enter the supratemporal fenestrae (75-2), and a triple intersection between the postorbital, parietal, and frontal is visible (Fig. 28A). This condition occurs in most alligatorids and crocodyloids. By contrast, some crocodylians exhibit a deep intersection of the frontoparietal suture with the supratemporal fenestrae (Fig. 28H), such that the postorbital-parietal suture is hidden on the inner wall of the supratemporal fenestra. Several gavialoids and species of *Borealosuchus* among other taxa exhibit an intermediate condition, in which the frontoparietal suture grazes the anterior edge of the supratemporal fenestrae (Fig. 28G). As in character state 2, the postorbital-parietal suture is hidden on the inner wall of the supratemporal fenestra, but the unique nature of the intersection with the supratemporal fenestrae warrants a separate character state. By conrast with the data matrix of Brochu et al. (2012), *Brachychampsa montana* is scored as lacking an intersection of the frontoparietal suture (75-2) (Fig. 28C) and *Navajosuchus mooki* (AMNH 6780) and *Hassiacosuchus haupti* (HLMD-Me-4415) are characterised by the intermediate condition (75-1).

76. Frontoparietal suture, shape between supratemporal fenestrae: concavo-convex (0); straight (1) (after Brochu, 1997a [86]).

The anatomical meaning of this character follows Brochu (1997b). A concavo-convex frontoparietal suture is exemplified by *Alligator mississippiensis*, *Crocodylus*, and *Brachychampsa montana* (Fig. 28A–C). By contrast, the straight condition is exhibited clearly in *Gavialis gangeticus* (Fig. 28G). Sookias (2019) did not consider this character to be robust based on the sample of extant crocodylians studied therein; however, broad differences can be observed and it is therefore retained herein. There are some differences in character scores between this dataset and that of earlier studies (e.g. Brochu et al., 2012), e.g. *Melanosuchus niger* $1 \rightarrow 0$ (Fig. 28D) and *Tomistoma schlegelii* $1 \rightarrow 0$ (NHMUK 1894.2.21.1). This probably represents the subtle difference between concavo-convex and straight in some taxa. Here, any degree of curvature in the suture was taken to be representative of the plesiomorphic condition.

Supraoccipital

77. Supraoccipital, exposure on dorsal skull table: present (0); absent (1) (after Norell, 1988 [11]; Brochu, 1997a [92])

78. Supraoccipital, extent of exposure on skull table: small, mediolateral width across dorsal supraoccipital exposure less than half that along the posterior margin of the parietal (0); moderate, mediolateral width across dorsal supraoccipital exposure more than half to equal that along the posterior margin of the parietal (1); large, such that the parietal is excluded from the posterior edge of skull table (2) (after Norell, 1988 [11]; Brochu, 1997a [82]) (ORDERED).

    Characters 77 and 78 were derived by reductively coding Character 92 from Brochu (1997b). These characters describe the degree of dorsal exposure of supraoccipital on the cranial table. Character state definitions are essentially the same as Brochu (1997b), but the supraoccipital exposure is quantified relative to the parietal width to improve repeatability. Furthermore, Character 78 is ordered, as it describes a transformational series. As in previous studies, caimanines exhibit the largest supraoccipital exposure, to the extent that the parietal is excluded from the posterior margin of the cranial table (Fig. 28D).

79. Supraoccipital, posterolateral tuberosities in dorsal view: not visible (0); visible (1) (after Jouve, 2004 [193]; in Jouve, 2016 [201]).

    As well as being dorsally exposed on the skull table, the supraoccipital can also be visible as two rounded processes extending beyond the level of the posterior margin of the cranial table in some taxa (Fig. 28C). This condition occurs in a variety of eusuchians, including *Hylaeochampsa vectiana* (NHMUK R177), most *Alligator* species (e.g. *A. mississippiensis* [NHMUK 68.2.12.16], *A. mcgrewi* [AMNH 7905]), and several gavialoids, e.g. *Gavialis gangeticus* (NHMUK 1974.3009) and *Piscogavialis jugaliperforatus* (SMNK 1282).

80. Supraoccipital, acute process projecting posteriorly from the midline of the cranial table: absent (0); present (1) (new character, after Hua and Jouve, 2004 [82]; Jouve, 2016 [82]).

    In a small number of mostly “gavialoids”, the supraoccipital forms a mediolaterally narrow, med-midline posterior projection on the cranial table, e.g. *Gavialis gangeticus* (NHMUK 1974.3009) (Fig. 28G) and *Piscogavialis jugaliperforatus* (SMNK 1282) (Fig. 28H). This projection is distinct from the paired posterior processes described in Character 79. This feature was recognised by Jouve (2016), who discretised this condition under a character describing supraoccipital size (Character 78 here). Here, this character state has been converted into an independent character, because the posterior projection of the supraoccipital, and degree of dorsal exposure of the supraoccipital, are not considered homologous.

Supratemporal fenestrae

81. Supratemporal fenestra, morphology of fenestral rim (at maturity): with fossa, dermal bones of skull roof do not overhang rim (0); dermal bones overhang rim (1); supratemporal fenestra completley closed (2) (after Norell, 1988 [9]; Brochu, 1997a [87]) (ORDERED).

The supratemporal fenestrae of most crocodylians are surrounded by fossae, such that the fenestral margins do not overhang (Brochu, 1999) (Fig. 28A, B). By contrast, bones surrounding the fenestrae in several crocodylians bear laminae that constrict the fenestra. This condition commonly occurs in caimanines, e.g. *Caiman* and *Melanosuchus niger* (Fig. 28D), but also in some crocodyloids, such as *Osteolaemus tetraspis* (NHMUK 1862.6.30.5) and *Voay robustus* (NHMUK R 36685). In rarer cases, the supratemporal fenestrae become completely closed, a condition only observed consistently in *Paleosuchus trigonatus* (Fig. 28E), *Paleosuchus palpebrosus* (AMNH 93812), and *Iharkutosuchus makadii* (Ösi et al., 2007), in this dataset. The supratemporal fenestrae may appear incipiently closed in some *Caiman crocodilus* specimens (e.g. FMNH 69859); however, this is disregarded in character scoring due to its rarity. As noted by Brochu (1999), the supratemporal fenestrae of hatchling caimans bear fossa, like all other extant crocodylians, with overhanging fenestral rims developing later in ontogeny. This could potentially distort character scores, as fossil taxa exhibiting supratemporal fossae, and scored as such, might be juveniles in which the overhanging condition has yet to be acquired. Similarly, fossil taxa exhibiting overhanging rims could later develop closed supratemporal fenestrae. There is little data on the precise timing of such ontogenetic changes. Based on illustrations in Blanco et al. (2015), juvenile *Caiman yacare* and *Caiman latirostris* (defined by SVL $<$ 500 mm) exhibit overhanging supratemporal fenestral rims. This suggests that although hatchling caimanines might not yet develop overhanging fenestrae, it is acquired relatively early in ontogeny. This is corroborated by personal observations of *Caiman crocodilus*, in which overhanging fenestral rims are observed in cranial specimens with a skull length $<$ 70 mm (FMNH 73712, FMNH 69837). Similar observations are made for the timing of closure of supratemporal fenestrae in *Paleosuchus*. Supratemporal fenestrae are closed in juvenile *Paleosuchus palpebrosus* (AMNH 93812) as reported by Medem (1958). Although they are open in juvenile *Paleosuchus trigonatus*, these are notably more constricted than the overhanging fenestrae of all other extant caimanines (Medem, 1958, AMNH 66391). Since no specimens included in this analysis could be regarded as hatchlings, and the development of overhanging and closed supratemporal fenestrae appears early in ontogeny, this character could be scored in all taxa where preserved.

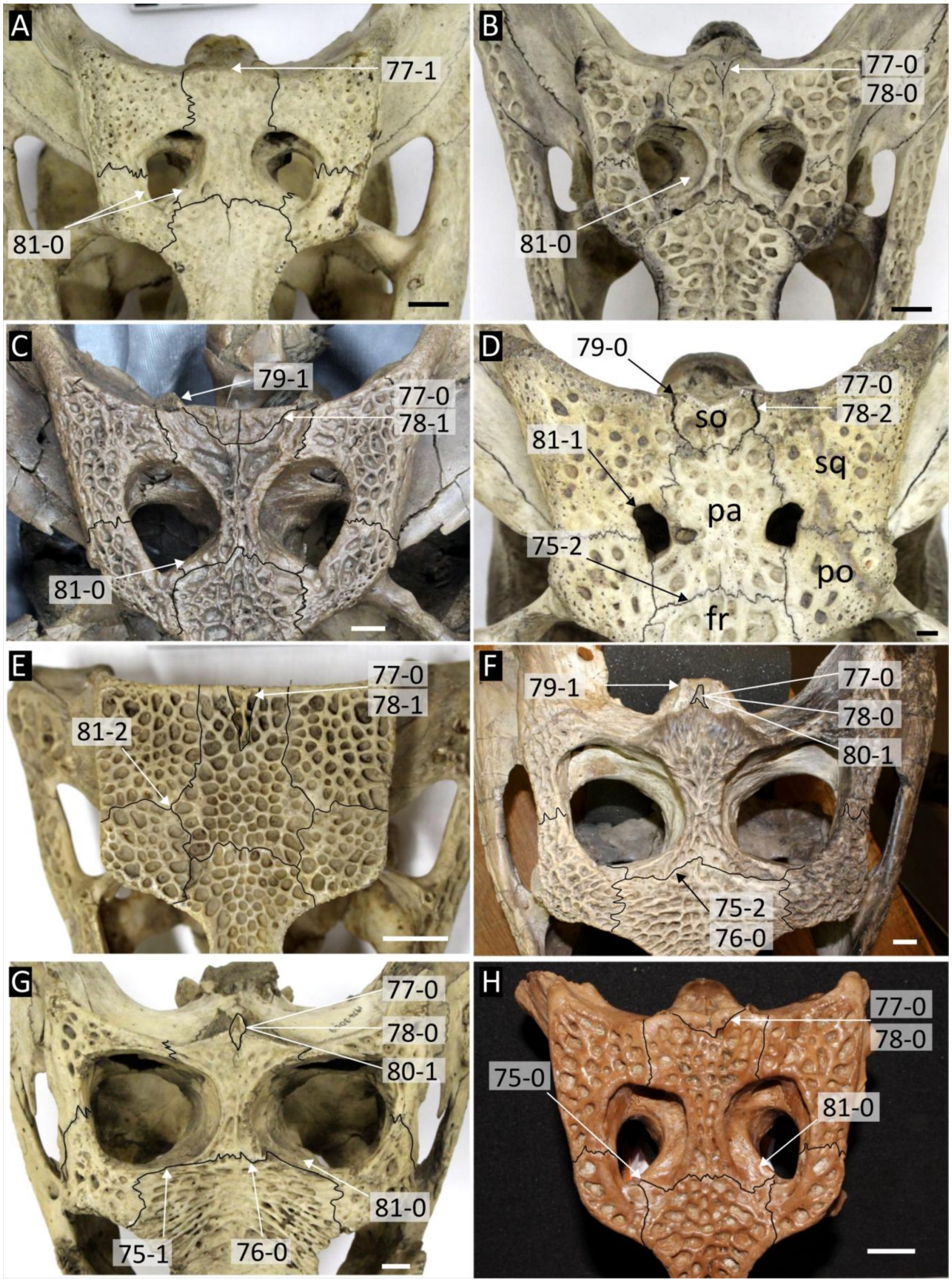

Figure 28: Sutural relationships and morphology of the cranial table in selected crocodylians. **A**, *Alligator mississippiensis* (NHMUK 1873.2.21.1); **B**, *Crocodylus acutus* (NHMUK 1975.997); **C**, *Brachychampsa montana* (UCMP 133901); **D**, *Melanosuchus niger* (NHMUK 45.8.25.125), **E**, *Paleosuchus trigonatus* (NHMUK 1868.10.8.1); **F**, *Piscogavialis jugaliperforatus* (SMNK 1282 PAL); **G**, *Gavialis gangeticus* (NHMUK 1974.3009); **H**, *Diplocynodon ratelii* (MNHN SG 539). Abbreviations: **fr**, frontal; **pa**, parietal; **po**, postorbital; **so**, supraoccipital; **sq**, squamosal. All scale bars = 1 cm.

82. Skull table morphology, acute dorsal indentation on the supraoccipital (and sometimes the parietal): absent (0); present (1) (new character, after Brochu 1997a [123]; Jouve, 2016 [123]).

The cranial table in most crocodylians is flat or weakly concave about its sagittal axis (82-0) (Fig. 29A). By contrast, some crocodylians exhibit a prominent indentation on the sagittal axis, which is most apparent in occipital view as a sharp notch on the supraoccipital (82-1). Jouve (2016) recognised this condition and introduced a new character state to Character 123 of Brochu (1997b). However, this morphological feature is considered as an independent, binary character here. In the data matrix of Jouve (2016), this condition only occurs in two taxa: *Tomistoma schlegelii* and *Kentisuchus spenceri*. However, the presence of this indentation is additionally recognised in several caimanines herein, including *Caiman latirostris* (Fig. 29C) and *Caiman gasparinae* (Fig. 29D).

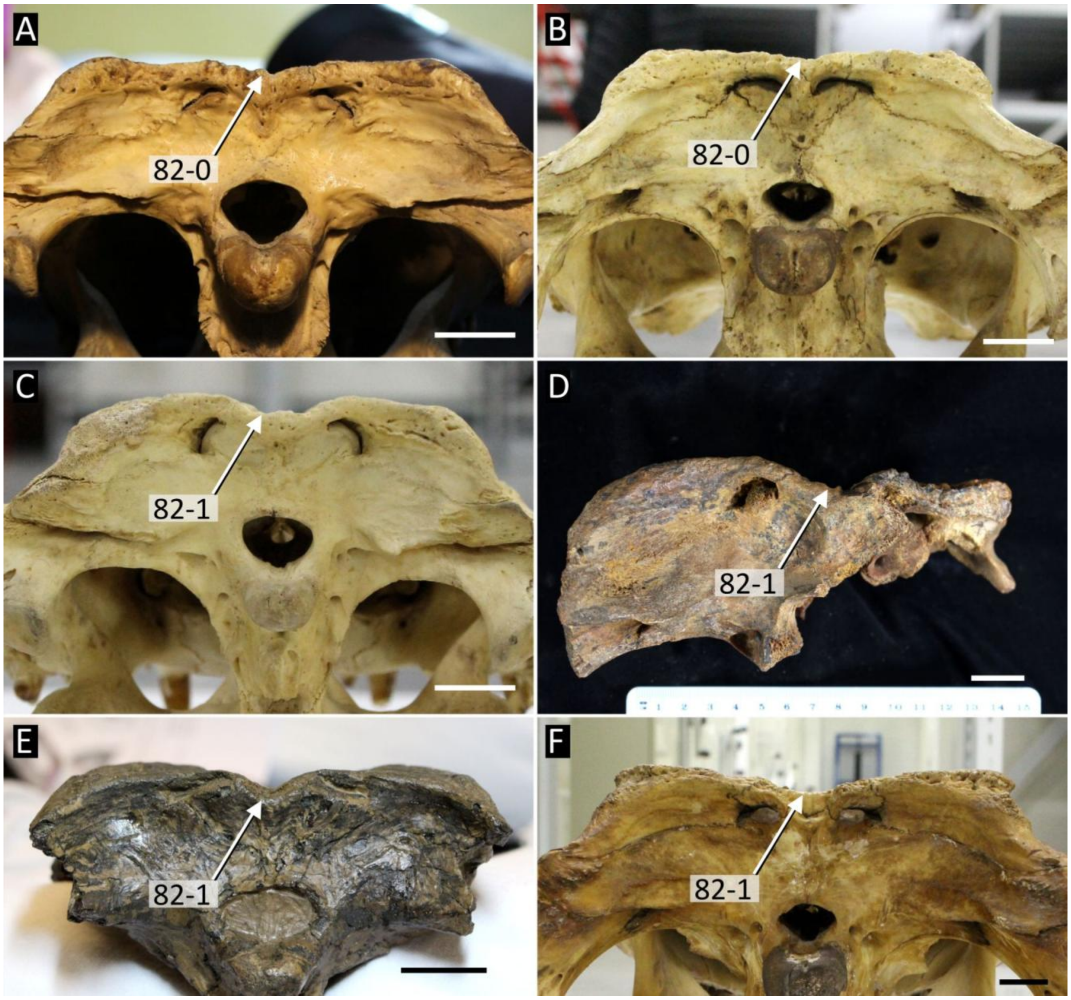

Figure 29: Occipital view of the cranium showing presence or absence of a dorsal midline indentation of the cranial table. **A**, *Caiman yacare* (MACN uncatalogued); **B**, *Crocodylus plaustris* (NHMUK 1897.12.31.1); **C**, *Caiman latirostris* (NHMUK 86.10.4.2); **D**, *Caiman gasparinae* (MLP 73-IV-15-1); **E**, *Kentisuchus spenceri* (NHMUK 38974); **F**, *Tomistoma schlegelii* (NHMUK 1894.2.21.1). All scale bars = 2 cm.

83. Parietal, sagittal crest between supratemporal fenestrae: absent (0); present (1) (after Clark, 1994 [33]; Pol et al., 2009 [33]).

Most eusuchians exhibit ornamentation on the cranial table in the form of regular, equally distributed pits. The Glen Rose Form (MCZ 3484), as well as several taxa commonly assigned to Paralligatoridae (including *Shamosuchus djadochtaensis* [Pol et al., 2009] and *Wannchampsus kirkpachi* [Adams, 2014]), also exhibit a sagittal crest between the supratemporal fenestrae. A handful of crocodylian taxa also exhibit this crest, including *Trilophosuchus rackhami* (Fig. 30C), *Mourasuchus amazonensis* (UFAC 1424), and *Mourasuchus arendsi* (MLP 73-IV-15-8).

84. Supratemporal fenestra, shallow fossa at anteromedial corner: present (0); absent (1) (after Brochu, 1997a [92]).

A shallow fossa at the anteromedial corner of the supratemporal fenestra is distinct from the development of fossae described in Character 81. The 'anteromedial fossa' is a discrete shelf that is ventrally inset in the supratemporal fenestrae, and is present in several taxa commonly assigned to the clade Allodaposuchidae, including *Allodaposuchus precedens* (Fig. 30C), *Lohuecosuchus megadontos* (Narváez et al., 2015), and *Agaresuchus fontisensis* (Narváez et al., 2016).

85. Parietal, medial wall of the supratemporal fenestra with one or more foramina: absent (0); present (1) (after Norell, 1988 [51]; Brochu, 1997a [104]).

Unlike most crocodylians, the medial parietal walls of extant *Caiman* species and *Melanosuchus niger* are perforated. This condition has recently been identified in the putative early caimanine *Protocaiman peligrensis* (Bona et al., 2018) (Fig. 30G) and herein in *Brachychampsa montana* (Fig. 30H). Despite reports of perforations in *Paleosuchus* (Norell, 1988; Brochu, 1999), closure of the supratemporal fenestrae in this taxon precludes observation of the medial parietal wall.

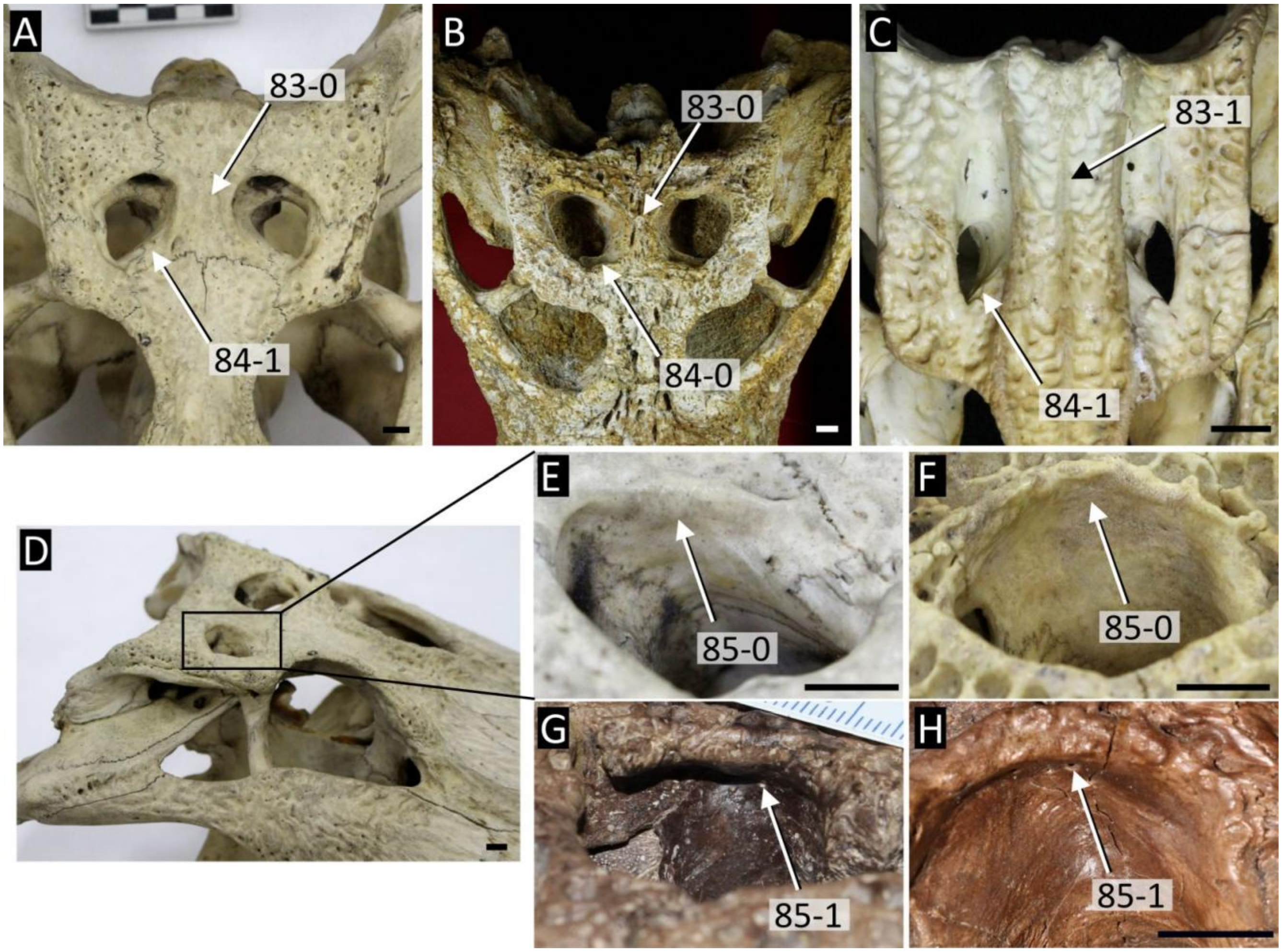

Figure 30: Morphology of the parietal in selected crocodylians. **A**, *Alligator mississippiensis* (NHMUK 1873.2.21.1); **B**, *Allodaposuchus precedens* (MMSVBN-12-10A); **C**, *Trilophosuchus rackhami* (QM F16856); **D**, area enlarged in E–F, **E**, *Alligator mississippiensis* (NHMUK 1873.2.21.1); **F**, *Crocodylus palustris* (NHMUK 1897.12.31.1); **G**, *Protocaiman peligrensis* (MLP 80X-10-1); **H**, *Brachychampsa montana* (UCMP 133901). All scale bars = 1 cm.

86. Parietal, recess communicating with pneumatic system: present (0); absent (1) (after Brochu, 1997a [154]).

As illustrated by Brochu (2004a, fig.17) this character describes an internal cavity (recess) in the parietal, which requires CT scans or a cross section through the skull. In the dataset of Brochu et al. (2012), the character is only scored in extant crocodylians, with a recess occurring in *Gavialis gangeticus*, *Tomistoma schlegelii*, and all extant crocodylids (86-0), but absent in alligatorids (86-1). It was only possible to evaluate the distribution of this feature in a small number of taxa, based on limited (and often low resoluion) CT scans, as well as cross sections through some extant crocodylian skulls (Fig. 31). Given the scarcity of data, character scores largely follow those of Brochu et al. (2012), although the recess appears to occur in at least two alligatorids, *Alligator mississippiensis* (Brochu, 2004a, fig.17C) and *Caiman yacare* (Fig. 31F).

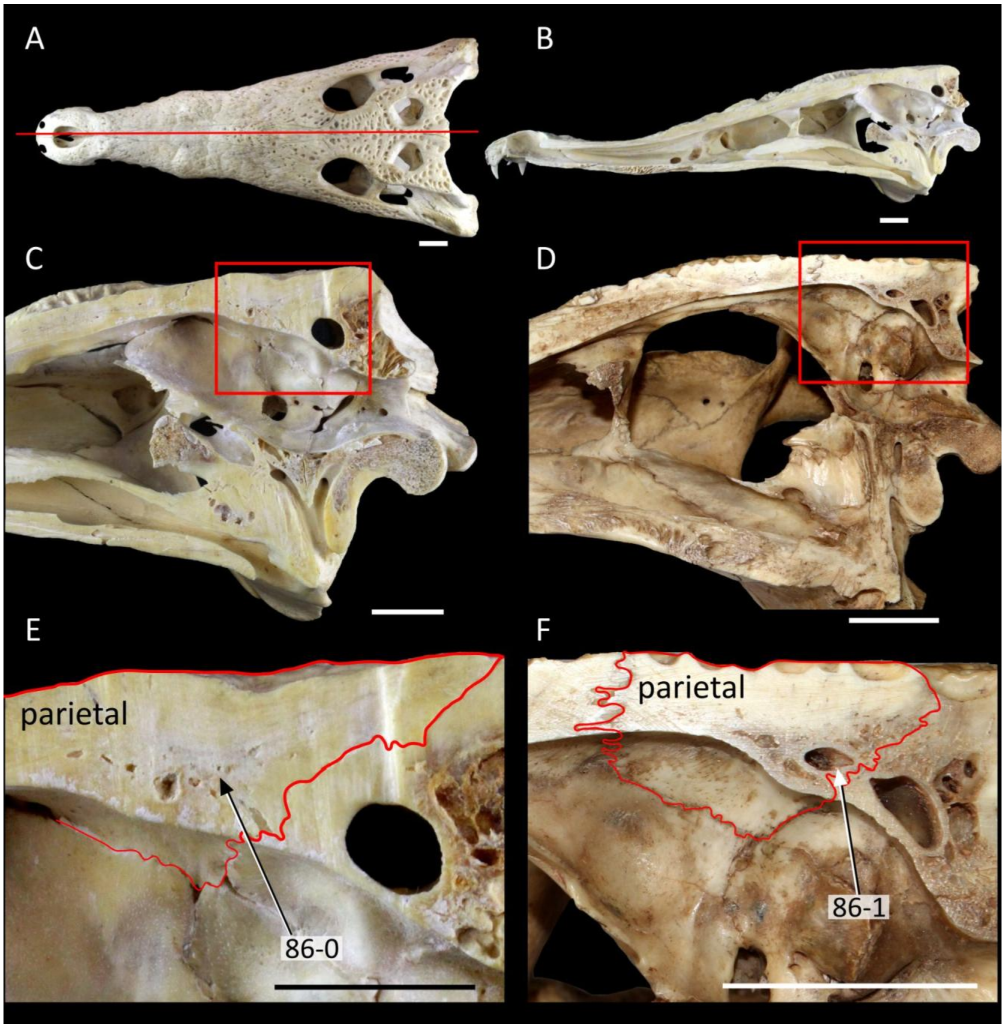

Figure 31: Sagittal sections of the skull showing the development of a parietal recess. The line of section in B–F is illustrated in *Crocodylus acutus* (**A**), with cross sections though *Crocodylus porosus* (FMNH 15229) (**B**, **C** and **D**) and *Caiman yacare* (MLP uncatalogued specimen) (**D**, **F**). All scale bars = 2 cm.

87. Supratemporal fenestra, posterior wall: quadrate forms entire ventral margin of orbitotemporal canal (no parietal-squamosal contact) (0); quadrate partially forms ventral margin of orbitotemporal canal (parietal and squamosal narrowly separated) (1); quadrate excluded from ventral margin of orbitotemporal canal (parietal and squamosal in contact) (2) (after Brochu, 1997a [131]) (ORDERED).

The orbitotemporal canal is a circular passage on the posterior wall of the supratemporal fenestra that is bound by the squamosal, parietal and, to varying degrees, the quadrate (Fig. 32). In most crocodylians, the quadrate forms the entire ventral margin of the canal, preventing contact between the parietal and squamosal here (Fig. 32A). Among extant crocodylians, this condition is observed in crocodylids, *Gavialis gangeticus* (NHMUK 1974.3009), and *Tomistoma schlegelii* (NHMUK 1894.2.21.1). By contrast, there is no participation of the quadrate to the ventra margin of the orbtotemporal canal in extant alligatorids (Fig. 32C). A handful of crocodylian taxa exhibit an intermediate condition, in which the quadrate forms a small portion of the ventrolateral orbitotemporal margin, constricted between the squamosal and parietal. This condition is observed in *Brachychampsa montana* (Fig. 32B), and most species of *Diplocynodon* (where preserved), including *D. ratelii* (MNHN SG 539) and *D. hantoniensis* (CAMSM TN 907).

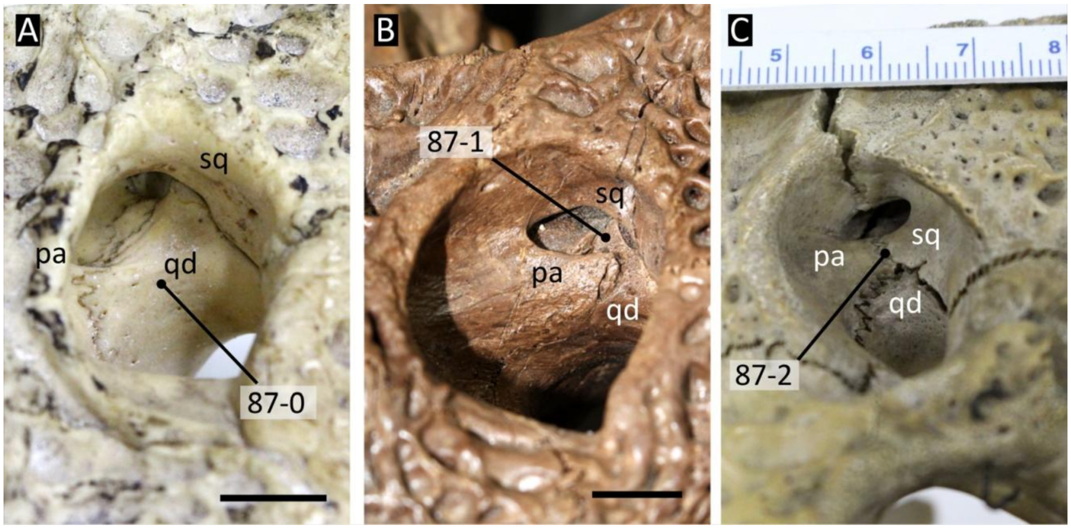

Figure 32: Variation in sutural relationships of the orbitotemporal canal in selected crocodylians. **A**, *Crocodylus niloticus* (NHMUK 1934.6.3.1); **B**, *Brachychampsa montana* (UCMP 133901); **C**, *Alligator sinensis* (NHMUK X184). Abbreviations: **pa**, parietal; **qd**, quadrate; **sq**, squamosal. Scale bars = 1 cm.

88. Supratemporal fenestra, posterior wall: squamosal-parietal suture passes medially to the orbitotemporal foramen, little to no development of fossa medial to orbitotemporal foramen (0); squamosal-parietal suture intersects dorsal margin of orbitotemporal foramen, large medial fossa (1); squamosal-parietal suture intersects dorsal margin of orbitotemporal canal, medial fossa extends over entire

width of posterior supratemporal fenestra wall (2) (new character, based on personal observations) (ORDERED).

The orbitotemporal canal in most crocodylians is large and circular to elliptical in shape. Within the orbitotemporal canal, there is a discrete fossa which forms the floor of the canal, on which the supraoccipital and prootic are exposed. There is notable variation in the morphology of the canal, which appears to be constrained by its mediolateral extent (Fig. 33). *Gavialis gangeticus* and *Thoracosaurus isorhynchus* represent extreme end members of this morphological variation. In *Thoracosaurus isorhynchus* (Fig. 33A), the canal is mediolaterally restricted: the parietal forms a large portion of the posterior wall of the supratemporal fenestra, and the parietal-squamosal suture intersects the orbitotemporal canal on the medial or ventromedial edge. This condition is also observed in *Allodaposuchus precedens* (Martin et al., 2016, fig.7) and *Portugalosuchus azenhae* (Mateus et al., 2019, fig.8). In *Gavialis gangeticus* (Fig. 33F), the parietal does not contribute much to the posterior supratemporal fenestra wall, exposing the prootic and supraoccipital on the floor of the canal. Also contrasting with *Thoracosaurus isorhynchus*, the squamosal-parietal suture intersects the dorsal margin of the orbitotemporal canal. The same condition is observed in *Gryposuchus colombianus* (Fig. 33E) and *Gryposuchus neogaeus* (MLP 26-413). In between these extremes lie almost all other crocodylians, e.g. *Piscogavialis jugaliperforatus*, *Eogavialis africanum*, and *Tomistoma schlegelii* (Fig. 33B–D). In those taxa the orbitotemporal canal is intermediate in size between *Thoracosaurus isorhynchus* and *Gavialis gangeticus*. Whereas the squamosal-parietal suture intersects the dorsal margin of the canal in those taxa, as in *Gavialis*, the parietal still forms a large portion of the posterior wall of the supratemporal fenestra. As these character states appear to belong on a morphological continuum, the character is ordered.

Postorbital

89. Postorbital, morphology of postorbital bar: anteroposteriorly expanded, elliptical in cross section (0); columnar and slender, circular in cross section (1) (after Norell, 1989 [3]; Brochu, 1997a [70]; Groh et al., 2020 [213]).

The morphology of the postorbital bar can be divided into two morphotypes in Eusuchia. In most eusuchians, the bar is narrow and columnar with an approximately circular cross section (Fig. 34D–F). By contrast, some taxa exhibit a postorbital bar that is anteroposteriorly longer than mediolaterally wide (Fig. 34A–C). The latter condition occurs in the outgroup, *Bernissartia fagesii* (IRScNB 1538), and several non-crocodylian eusuchians such as *Hylaeochampsa vectiana* (NHMUK R177), indicating that it is the plesiomorphic condition for Eusuchia. The character states have been modified from Brochu (1997b) by describing the shape of the postorbital bar in

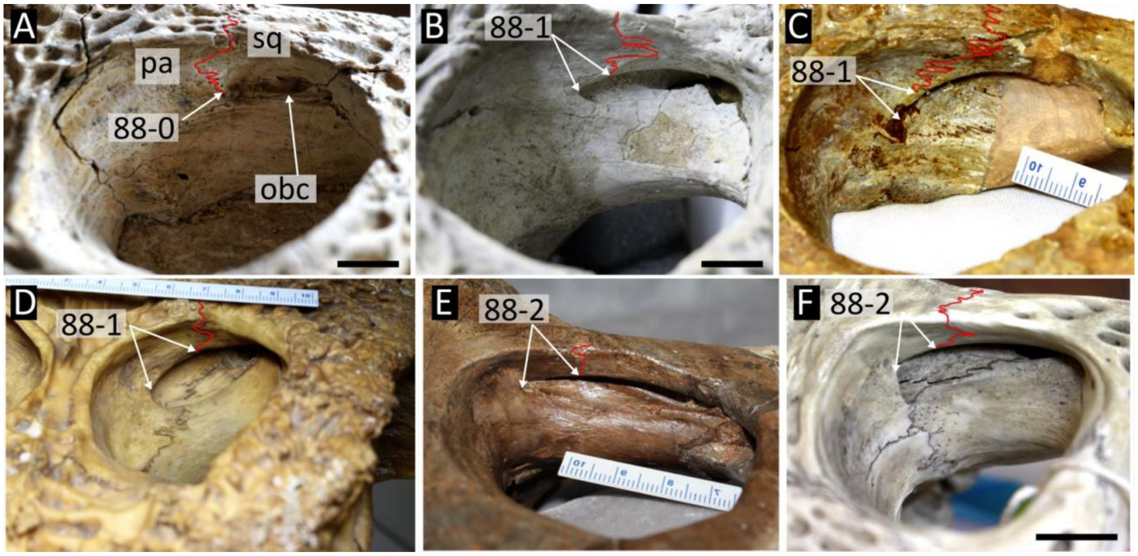

Figure 33: Variation in morphology of the orbitotemporal canal in crocodylian taxa. **A**, *Thoracosaurus isorhynchus* (MNHN.F.MTA 61); **B**, *Piscogavialis jugaliperforatus* (SMNK 1282 PAL); **C**, *Eogavialis africanum* (NHMUK PV R3430); **D**, *Tomistoma schlegelii* (NHMUK 1894.2.21.1); **E**, *Gryposuchus colombianus* (UCMP 38358); **F**, *Gavialis gangeticus* (NHMUK 61.4.1.2). Abbreviations: **obc**, orbitotemporal canal; **pa**, parietal; **sq**, squamosal. Scale bars in A, B, F = 1 cm, all other scale bars = cm.

cross section.

90. Postorbital, protuberance on the dorsolateral margin of the postorbital bar (at maturity): present (0); absent (1) (after Norell, 1989 [2]; Brochu, 1997a [134]; Brochu et al., 2012 [132]).

This character was modified from its formulation in Brochu et al. (2012): "*Postorbital bar bears process that is prominent, dorsoventrally broad, and divisible into two spines (0) or bears process that is short and generally not prominent (1)*". This character has been simplified to describe the presence or absence of a postorbital process at maturity. This modification is based on the variability of the postorbital bar process morphology. It is not always divisible into two spines, nor is it always dorsoventrally tall. Two discrete spines could only be observed in *Gavialis gangeticus* in this study (NHMUK 1974.3009). Where a process is present in other taxa, it can form a single sharp projection (e.g. *Thoracosaurus isorhynchus*, Fig. 34C), an anteroposteriorly elongated ridge (e.g. *Eogavialis africanum*, Fig. 34B, *Hylaeochampsa vectiana*, NHMUK R177, and *Allodaposuchus precedens*, MMS/VBN-12-10A), or a single and large irregular protuberance (e.g. *Gryposuchus colombianus*, UCMP 41136). The character is also only scored for matrue individuals, since prominent processes occur in juvenile individuals of several extant crocodylians but are lost at maturity, e.g. *Alligator mississippiensis* (Norell, 1989), and *Tomistoma schlegelii* (Aoki, 1976; Buffetaut, 1985).

91. Postorbital bar, orientation: laterally inclined, greater than or equal to 20° (bar visible in dorsal

view) (0); slightly inclined to vertical, lateral inclination $< 20^\circ$ (not visible in dorsal view) (1) (after Jouve, 2004 [192]; Jouve et al., 2008 [184]; Hastings et al., 2010 [50]; Jouve, 2016 [184]; Groh et al., 2019 [211]).

The postorbital bar is subvertical in *Gavialis gangeticus* (Fig. 34I), such that it is concealed underneath the cranial table in dorsal view. A similar condition is observed in several "gavialoids", such as *Piscogavialis jugaliperforatus* (SMNK 1282 PAL) and *Eogavialis africanum* (NHMUK R3108). Most other eusuchians exhibit a less inclined postorbital bar, such that it is visible in dorsal view (Fig. 34G–H).

92. Postorbital bar: flush with dorsolateral margin of jugal (0); dorsolateral margin of jugal raised to form ridge, with sulcus separating it from postorbital bar (1) (after Benton and Clark, 1988; Norell and Clark, 1990 [3]; Brochu, 1997a [146]).

The wording of this character has received slight modifications from the original but its anatomical meaning is unchanged; however, there have been several character score changes compared to other datasets. In most eusuchians, the ventral margin of the postorbital bar is prominently delimited from the dorsolateral margin of the jugal arch by a sulcus, as in *Tomistoma schlegelii* (Fig. 35A). In fewer cases, the demarcation between the ventral margin of the postorbital bar and the dorsolateral edge of the jugal arch is not apparent, such that one merges into the other, e.g. *Gavialis gangeticus* (Fig. 35B). By contrast to the scores of existing datasets, the 'flush' condition is no longer recognised in several "gavialoid" taxa such as *Piscogavialis jugaliperforatus* (Fig. 35C), *Thoracosaurus isorhynchus* (Fig. 35D), and *Eogavialis africanum* (Fig. 35E) (Brochu et al., 2012; Narváez et al., 2016). Furthermore, changes have been made to the scores of some non-crocodylian taxa, such as *Bernissartia fagesii* (92: $0 \rightarrow 1$) and *Hylaeochampsa vectiana* (NHMUK R177). The latter has consistently been scored for the flush condition (e.g. Brochu, 1999; Brochu et al., 2012; Lee & Yates, 2018; Narváez et al., 2016); however, the jugal of the holotype is damaged and the condition cannot be determined (92: $0 \rightarrow ?$) (Fig. 35F).

Jugal

93. Jugal, posterodorsal jugal foramen, at base of postorbital bar: absent or small, diameter less than half the minimum mediolateral width of the jugal arch (0); large, equal to or greater than half the minimum jugal arch width (1) (after Jouve, 2016 [239]).

In most crocodylians, one or more foramina are often present at the base of the postorbital bar, on the dorsal surface of the jugal arch. These foramina are typically small (Fig. 35A, C), but in several, mostly longirostrine crocodylians they are enlarged, e.g. *Gavialis gangeticus* (Fig. 35B),

Thoracosaurus isorhynchus (Fig. 35D), and *Eogavialis africanum* (Fig. 35E). Alhough it was considered impractical to measure the diameter of the foramen, it is nevertheless quantified simply in proportion to the mediolateral width of the jugal arch to improve repeatability.

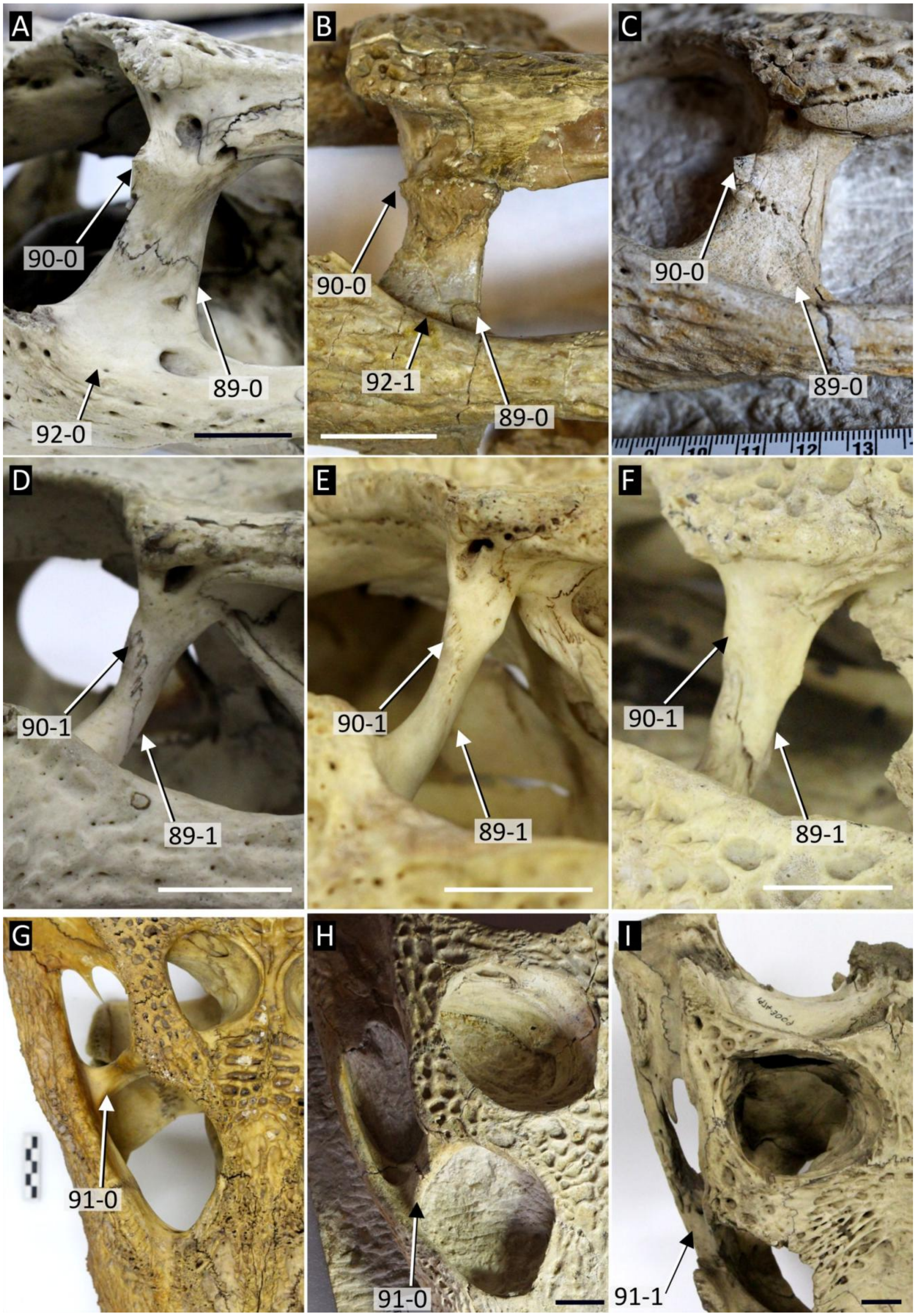

Figure 34: Morphology of the postorbital bar in selected crocodylians. **A**, **I**, *Gavialis gangeticus* (NHMUK 1974.3009); **B**, *Eogavialis africanum* (NHMUK PV R3108); **C**, **H**, *Thoracosaurus isorhynchus* (MNHN.F.MTA 61); **D**, *Alligator mississippiensis* (NHMUK 1873.2.21.1); **E**, *Caiman latirostris* (NHMUK 1897.12.31.1); **F**, *Crocodylus palustris* (NHMUK 1897.12.31.1); **G**, *Tomistoma schlegelii* (NHMUK 1894.2.21.1). All scale bars = 2 cm.

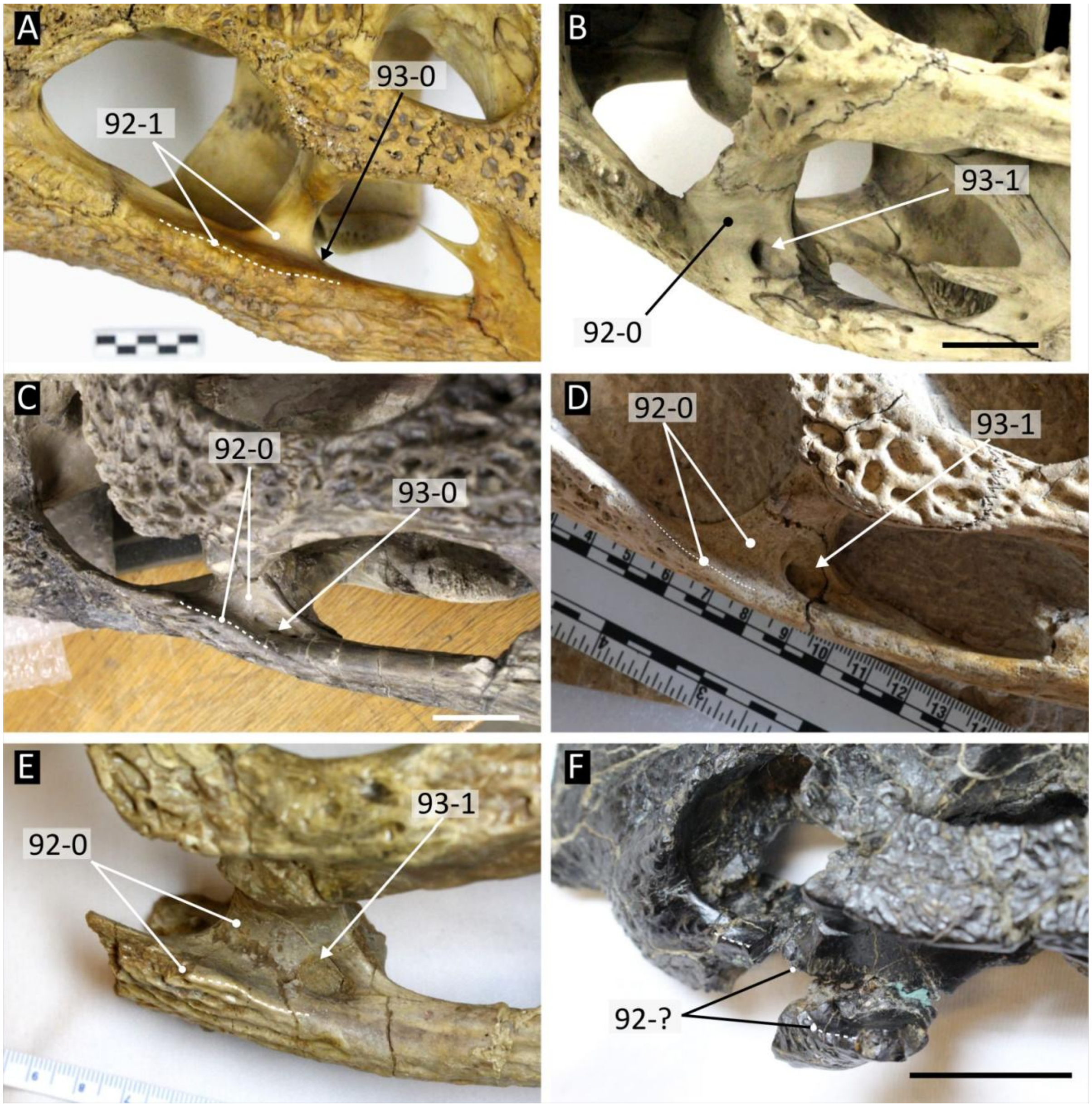

Figure 35: Dorsal view of the postorbital bar in selected crocodylians, showing variation in the inset of the postorbital bar. **A**, *Tomistoma schlegelii* (NHMUK 1894.2.21.1); **B**, *Gavialis gangeticus* (NHMUK 1974.3009); **C**, *Piscogavialis jugaliperforatus* (SMNK 1282 PAL); **D**, *Thoracosaurus isorhynchus* (MNHN.F.MTA 61); **E**, *Eogavialis africanum* (NHMUK PV R3108); **F**, *Hylaeochampsa vectiana* (NHMUK PV R 177). All scale bars = 2 cm.

94. Orbit, dorsal profile of jugal forming posteroventral margin: convex or straight, continuous with the dorsal margin of the lower temporal bar (0); posteroventrally sloping, gradually descending into the lower temporal bar (1); strongly convex with a step anterior to the lower temporal bar (2); abruptly angled ventrally, creating a near vertical margin descending toward the postorbital bar (i.e. a notch) (3) (after Brochu, 1997a [139]; Jouve et al., 2006: fig.7; Jouve, 2016 [139]; Lee and Yates, 2018 [61]).

This character originated in Brochu (1997b) (Character 139), wherein it described the absence or presence of a 'notch' in the orbital margin. The notched condition is typified by *Gavialis gangeticus* (94-3) (Fig. 36D), and was scored in several other "gavialoids", e.g. *Gryposuchus*, *Piscogavialis* and *Eogavialis* (Brochu et al., 2012). Later studies modified this character, adding new character states to reflect the greater variation in the dorsal profile of the jugal. Jouve (2016) recognised two additional states: a convex shaped jugal profile that is present in most species of *Crocodylus* and *Tomistoma schlegelii* (Fig. 36A), and a step-like condition that is present mainly in caimanines (Fig. 36C). Furthermore, Lee and Yates (2018) described a posteroventrally sloping condition in their modification of the character, which can be exemplified by *Piscogavialis* (Fig. 36B). Character score changes have also been made, including the recognition of the abruptly angled, 'notched' condition (94-3) in several species of the giant caimanine genus, *Mourasuchus*, e.g. *M. atopus* (UCMP 38012). All species of *Mourasuchus* were formerly scored as lacking a notch (e.g. Brochu et al., 2012; Cidade et al., 2017; Souza-Filho et al., 2019), but the morphology of the jugal is strikingly similar to *Gavialis gangeticus*, which might be a result of similar modifications towards a telescopic orbit. The decision was made to assimilate the various character states into an unordered multistate character, but it is recognised that some of these character states might belong to a transformational series that could be ordered. In particular, character states 0, 1, and 3 (Fig. 36A, B, D) could be considered part of an ordered character describing the progressive deepening of a notch in the jugal. Similar gradational differences in gavialoids were described by Salas-Gismondi et al. (2016). On the other hand, difficulty arises in the placement of the typical caimanine condition (Fig. 36C), which does not have an obvious place in this continuum. As such, the character is currently best treated as unordered.

95. Jugal, ventral margin of jugal arch: concave (0); or straight (1) (after Jouve, 2004 [182]; in Jouve et al., 2008 [178]).

The ventral margin of the jugal arch is strongly concave in most crocodylians (Fig. 36A). By contrast, some taxa exhibit a straight jugal arch, which is typically dorsoventrally shallow, including *Gavialis gangeticus* (Fig. 36D), *Eosuchus lerichei* (IRScNB R 49), *Eosuchus minor* (USNM 299730) and *Piscogavialis jugaliperforatus* (Fig. 36B).

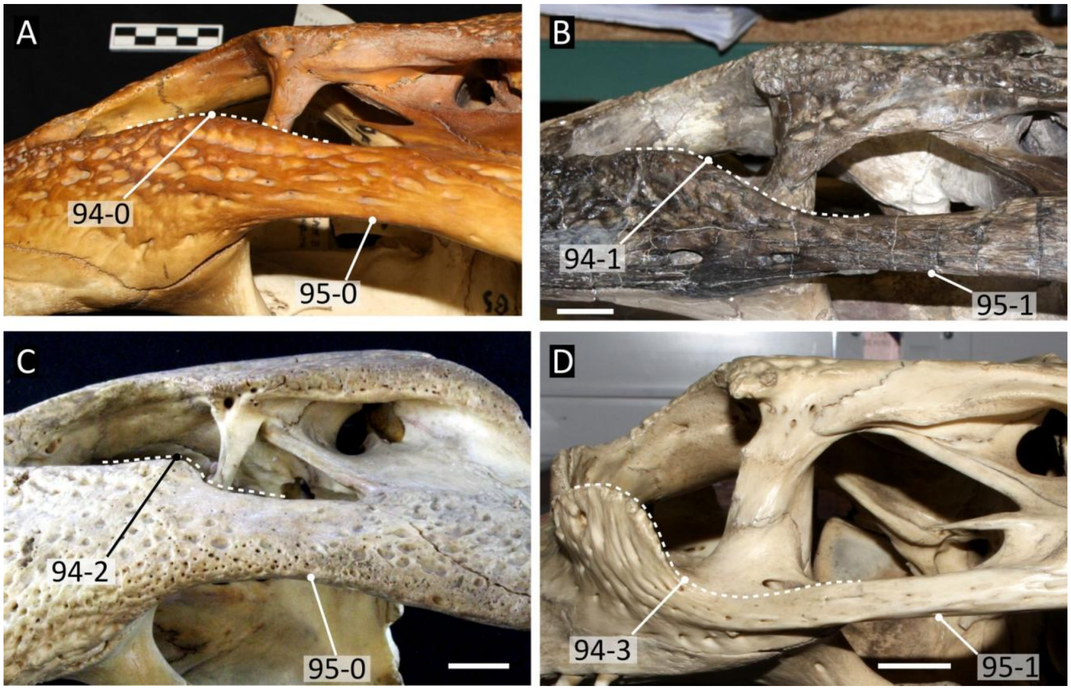

Figure 36: Left lateral view of the jugal arch in: **A**, *Tomistoma schlegelii* (NHMUK 1894.2.21.1); **B**, *Piscogavialis jugaliperforatus* (SMNK 1282 PAL); **C**, *Caiman latirostris* (FMNH 9713); **D**, *Gavialis gangeticus* (NHMUK 1974.3009). Scale bar A = cm, all other scale bars = 2 cm.

96. Jugal, ventrolateral sulcus on jugal and maxilla, at level of the jugal-maxilla suture: absent (0); present (1) (new character, adapted from Wu et al., 1996; 2001a; Kraus, 1998).

97. Jugal, ventrolateral foramina adjacent to the jugal-maxilla suture: small, less than half the diameter of the last maxillary alveolus (and usually numerous) (0); large, equal to or greater than half the diameter of the last maxillary alveolus (usually 2–3 foramina) (new character, based on personal observations).

The specific epithet of *Piscogavialis jugaliperforatus* (Kraus, 1998) arises from the presence of large ventrolateral foramina on the jugal, adjacent to the jugal-maxilla suture. Foramina are present in this position in most crocodylians; however, there is variation in their size, number, and whether or not they are situated in a prominent sulcus. In addition to *Piscogavialis*, enlarged foramina, equal in diameter to the last maxillary alveolus, are present in *Argochampsa krebsi* (Hua & Jouve, 2004) (Fig. 37D), *Gryposuchus colombianus* (Fig. 37C), and *Dadagavialis gunai* (Salas-Gismondi et al., 2019). This contrasts to the condition in *Gavialis gangeticus*, wherein a linear array of small foramina pierce the jugal in both juvenile and adult specimens (Fig. 37B). This appears to be the plesiomorphic eusuchian condition, with small foramina observed in *Bernissartia fagesii* (IRScNB 1538), and *Hylaeochampsa vectiana* (NHMUK R177). The size of the sulcus (if present at all) in which these foramina sit appears to be independent of foramen size. For example, in *Piscogavialis* (SMNK 1282 PAL), there is little to no sulcus, whereas the foramina are equally large in *Gryposuchus colombianus* (Fig. 37C), but the sulcus is much deeper. The 'groove-shaped recess' described in *Stangerochampsa* (Wu et al., 1996) and *Leidyosuchus* (Wu et al., 2001a), and a conspicuous depression on the jugal present in *Asiatosuchus depressifrons* (Delfino et al., 2019; Delfino & Smith, 2009) (IRScNB R 0251), are herein considered homologous to this sulcus.

Infratemporal fenestra

98. Infratemporal fenestra, dorsal margin shape: acute, triangular (0); broadly curved, oval-shaped (1) (after Salas-Gismondi et al., 2016 [204]).

The infratemporal fenestra is triangular in most eusuchians, with an acute dorsal margin (98-0). This includes all extant alligatorids and most crocodylids (Fig. 38C–E). By contrast, several "gavialoids" exhibit a rounded dorsal margin, such that the fenestra is more oval-shaped (98-1). In common with this analysis, Salas-Gismondi et al. (2016) scored several taxa for the rounded condition, such as *Eosuchus*, *Gryposuchus colombianus*, and *Eogavialis africanum*. However, by contrast to that study, the derived condition is additionally recognised in *Gavialis gangeticus* (Fig. 38A), *Tomistoma schlegelii* (Fig. 38B), and several non-gavialoid crocodylians, including *Asiatosuchus depressifrons* (IRScNB R251) and *Crocodylus johnstoni* (e.g. QM J4280).

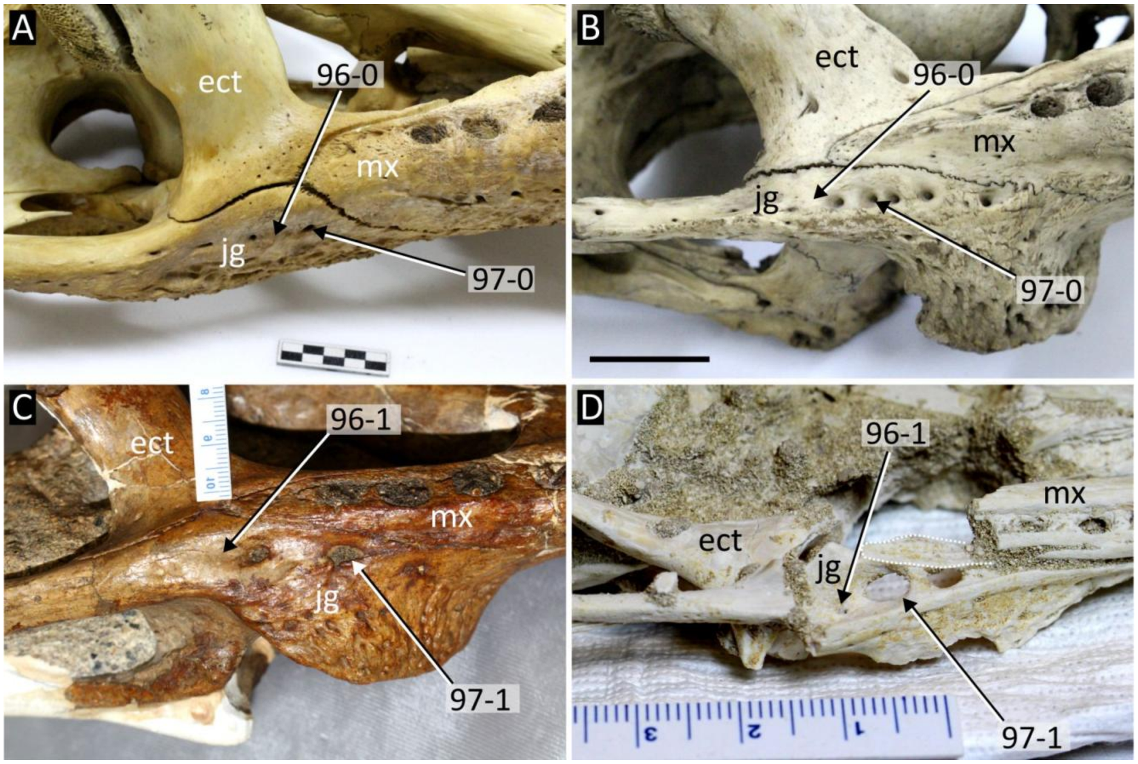

Figure 37: Ventrolateral view of the maxilla-jugal-ectopterygoid sutural intersections. **A**, *Tomistoma schlegelii* (NHMUK 1894.2.21.1); **B**, *Gavialis gangeticus* (NHMUK 1974.3009); **C**, *Gryposuchus colombianus* (UCMP 41136) (digitally reversed); **D**, *Argochampsa krebsi* (NHMUK R 36872). Abbreviations: **ect**, ectopterygoid; **jg**, jugal; **mx**, maxilla. Scale bar B = 5 cm, all other scale bars = cm.

99. Infratemporal fenestra, dorsal extent of quadratojugal: reaches dorsal angle of fenestra (0); does not reach dorsal angle of fenestra (1) (after Buscalioni et al., 1992 [6]; Brochu, 1997a [80]).

    The quadratojugal forms the posterior margin of the infratemporal fenestra in all crocodylians, but variation occurs in its dorsal extent. In *Bernissartia fagesii* and most eusuchians the quadratojugal reaches the dorsal angle of the fenestra (Fig. 38B), preventing the quadrate from participating in its posterior margin (99-0) (Buscalioni et al., 1992, fig.9; Norell et al., 1994, fig.8; Brochu, 1999, fig.25). By contrast, the quadratojugal forms only half the length of the posterior margin of the infratemporal fenestra in extant *Crocodylus* species, with the remainder formed by the quadrate (Fig. 38E). Several caimanines exhibit a similar condition, but differ in that the quadratojugal is dorsally truncated by the postorbital, which forms the remainder of the posterior fenestral margin (Fig. 38D).

100. Postorbital, posteroventral process in quadratojugal at dorsal corner of the infratemporal fenestra: absent (0); present (1) (after Norell, 1989 [11]; Brochu, 1997a [76]).

101. Postorbital, morphology of posteroventral process in quadratojugal: narrow with acute 'V' shaped tip (0); broad, blunt tip (1) (after Norell, 1989 [11]; Brochu, 1997a [76]).

The dorsal margin of the infratemporal fenestra is a complex region where several bones intersect, including the quadrate, quadratojugal, squamosal and postorbital. Norell (1989) described the presence of a 'postorbital process' in *Bernissartia fagesii*, *Gavialis gangeticus*, and all alligatorids, which descends along the posterior margin of the infratemporal fenestra and is ost clearly observed in lateral view (Fig. 38A). Brochu (1999) also recognised this 'postorbital process' but suggested that the intersection of the postorbital, quadrate, and quadratojugal was more complex, and best viewed from a ventromedial direction. Accordingly, Brochu (1999) incorporated the presence of a postorbital process into a multistate character that also described various sutural intersections between the postorbital, quadrate, and quadratojugal ventromedially. This version of the character is commonly used in crocodylian phylogenetics; however, examination of several datasets reveals that the state describing the presence of a postorbital process is not scored in any taxon that clearly possesses it (e.g. Brochu, 1999; Brochu et al., 2012; Cidade et al., 2017; Iijima & Kobayashi, 2019; Jouve, 2016; Narváez et al., 2016). Here, the presence of a postorbital process is treated as a binary character following Norell (1989), independent of the ventromedial sutural relationships of the postorbital, quadrate, and quadratojugal (Characters 105 and 106). Furthermore, variation in the morphology of the postorbital process is also recognised (Character 101). Where preserved, all species of *Alligator* exhibit a small, acute postorbital process (101-0, Fig. 38C). This contrasts with the condition exhibited by most caimanines, which have a notably broader postorbital process (101-1, Fig. 38D).

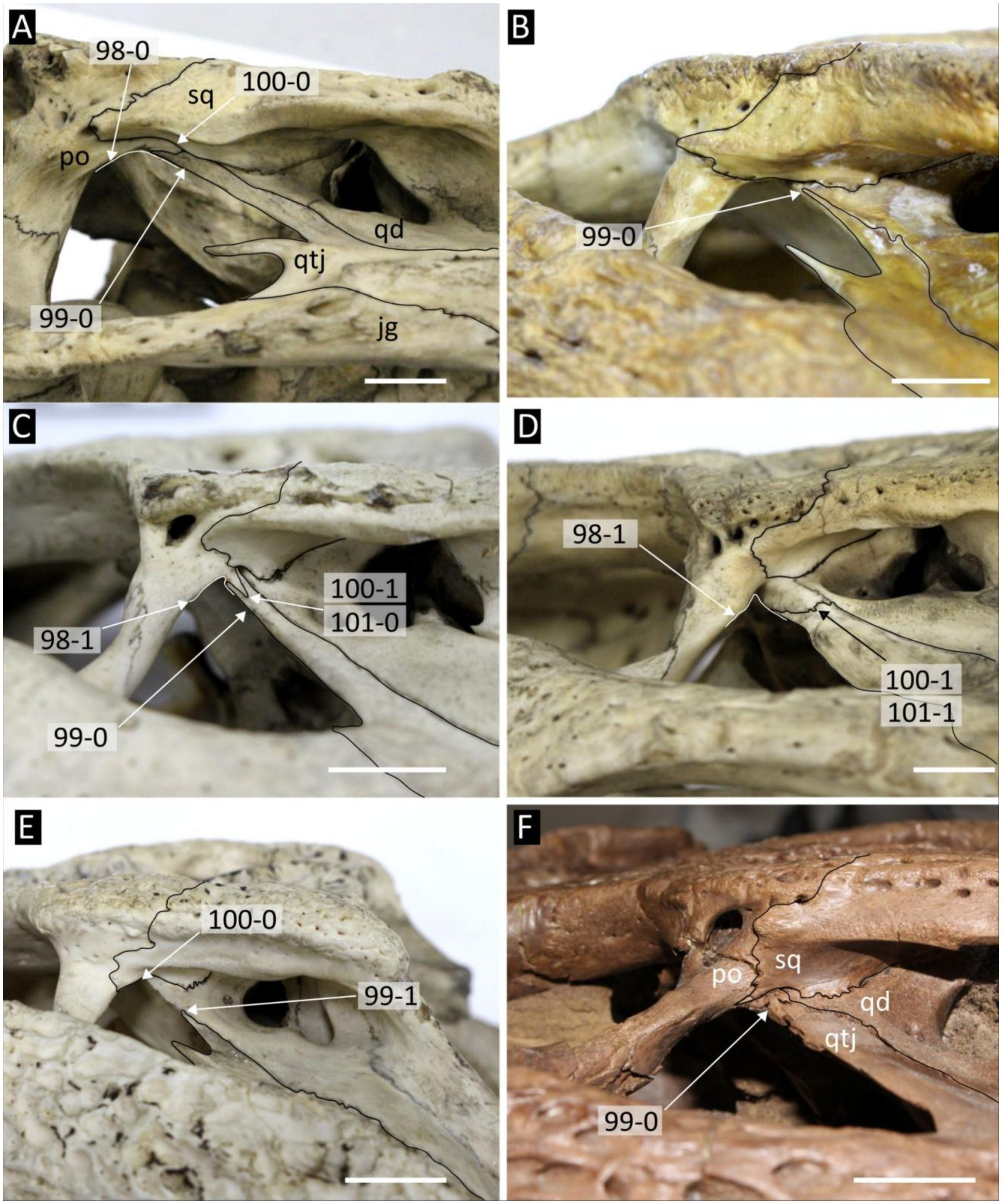

Figure 38: Sutural relationships and morphology of the infratemporal fenestra in selected crocodylians. **A** *Gavialis gangeticus* (NHMUK 1974.3009); **B**, *Tomistoma schlegelii* (NHMUK 1894.2.21.1); **C**, *Alligator mississippiensis* (NHMUK 1873.2.21.1); **D**, *Melanosuchus niger* (NHMUK 45.8.25.125); **E**, *Crocodylus niloticus* (NHMUK 1934.6.3.1); **F**, *Brachychampsa montana* (UCMP 133901). Abbreviations: **po**, postorbital; **qd**, quadrate; **qtj**, quadratojugal; **sq**, squamosal. All scale bars = 2 cm.

102. Infratemporal fenestra, posterior angle: quadratojugal forms posterior angle (0); quadratojugal-jugal suture lies at posterior angle (1); jugal forms posterior angle (2) (after Norell, 1989 [5]; Brochu, 1997a [75]) (ORDERED).

Norell (1989) recognised that the quadratojugal forms the posterior angle of the infratemporal fenestra in *Bernissartia fagesii*, *Gavialis gangeticus*, and all alligatorids (Fig. 39A), contrasting with the condition in extant *Crocodylus* species, in which the angle is formed by the jugal (Fig. 39D). Brochu (1999) later introduced a condition in which the jugal-quadratojugal suture lies directly on the posterior angle of the infratemporal fenestra (Fig. 39C) (102-1), a condition which appears to be restricted to several mekosuchines according to the data matrices of Brochu (2007a) and Brochu et al. (2012). This latter condition is recognised more widely in crocodylids in this analysis, with a polymorphic condition present in several *Crocodylus* species, e.g. *C. porosus* (102-1 in NHMUK 1852.12.9.2, 102-2 in NHMUK 85.2.4.1). Furthermore, the character is ordered, describing the progressive decrease in participation of the quadratojugal in the posterior angle of the infratemporal fenestra.

103. Quadratojugal, development of spina quadratojugalis (at maturity): prominent (0); greatly reduced or absent (1) (after Norell, 1989 [1]; Brochu, 1997a [69]).

This character has received minor modifications to wording only, and the meaning of the character is as originally described by Norell (1989). A prominent spine (Fig. 39A, C, D) is considered plesiomorphic in Crocodylia as it occurs in *Bernissartia fagesii* as well as taxa such as *Allodaposuchus precedens* (Narváez et al., 2019). All extant crocodylids, as well as *Tomsitoma schlegelii* (NHMUK 1894.2.21.1), and *Gavialis gangeticus* (NHMUK 1974.3009), exhibit the prominent spine, which is absent or restricted to a small protuberance in alligatorids (Fig. 39B).

104. Quadratojugal, position of spina quadratojugalis:low, near posterior angle of infratemporal fenestra (0); high, between posterior and dorsal angles of infratemporal fenestra (1) (after Brochu, 1997a [114]).

This character has received only minor modifications to wording but several character score changes. In most eusuchians that possess a quadratojugal spine, it occurs at a ‘low’ position, below the dorsoventral mid-height of the infratemporal fenestra (Fig. 39A). In all alligatorids that preserve a quadratojugal spine, it occurs beyond the dorsoventral mid-height of the infratemporal fenestra (Fig. 39B). Fewer taxa are scored for the derived condition than to previous studies (e.g. Brochu et al., 2012; Cidade et al., 2017; Salas-Gismondi et al., 2015), as the quadratojugal spine is too poorly developed or absent to determine its position in some taxa.

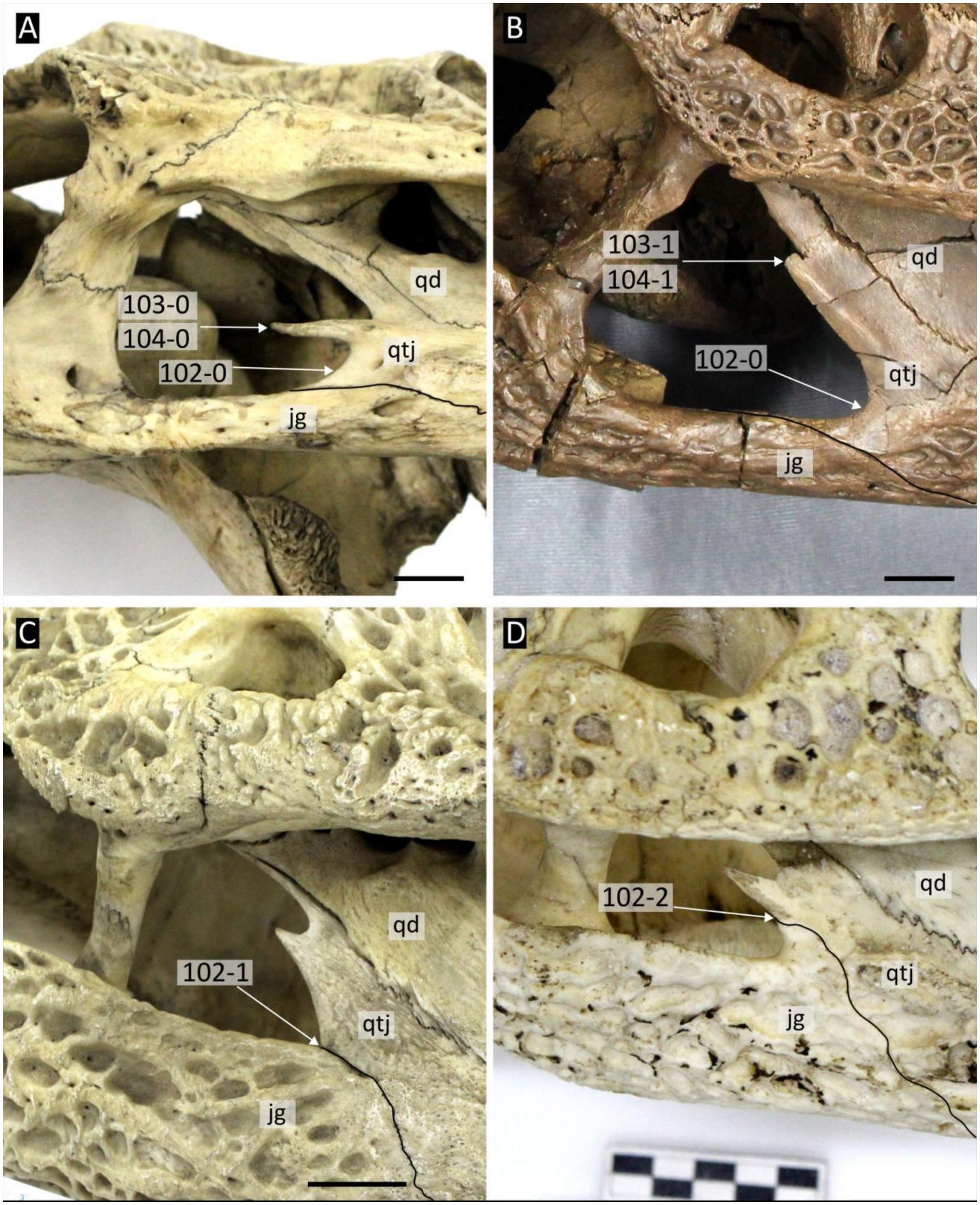

Figure 39: Variation in the contribution of the quadratojugal to the infratemporal fenestra in selected crocodylians. **A**, *Gavialis gangeticus* (NHMUK 1974.3009); **B**, *Brachychampsa montana* (UCMP 133901); **C**, *Crocodylus porosus* (NHMUK 1852.12.9.2); **D**, *Crocodylus niloticus* (NHMUK 1934.6.3.1). Abbreviations: **jg**, jugal; **qd**, quadrate; **qtj**, quadratojugal. All scale bars = 2 cm.

105. Postorbital, medial contact with quadrate at dorsal corner of the infratemporal fenestra: absent (0); present (1) (after Brochu, 1997a [76]).

106. Postorbital, medial contact with quadratojugal at dorsal angle of infratemporal fenestra: absent (0); present (1) (after Brochu, 1997a [76]).

Characters 105 and 106 describe variation in sutural relationships between the postorbital, quadratojugal, and quadrate in a ventromedial orientation (Fig. 40). This variation was originally discretised as follows: "*Postorbital neither contacts quadrate nor quadratojugal medially (0), or contacts quadratojugal, but not quadrate, medially (1), or contacts quadrate and quadratojugal at dorsal angle of infratemporal fenestra (2), or contacts quadratojugal with significant descending process (3)*" (Brochu, 1997b). The original formulation incorrectly implies that contact between the quadratojugal and postorbital is homologous to contact between the quadrate and postorbital. It also precludes the recognition of evolutionary relationships between taxa that share a postorbital-quadratojugal contact. Furthermore, alternative combinations of postorbital-quadrate-quadratojugal contact cannot be accounted for in the original formulation. For example, Jouve (2016) recognised a contact between the postorbital and quadrate to the exclusion of the quadratojugal in *Maroccosuchus zennaroi*, which is also recognised here in *Tomistoma schlegelii* (Fig. 40F). As a result, the original character was converted into two binary presence/absence characters. Generally, crocodylids exhibit no medial contact between the quadrate, quadratojugal, and postorbital (Fig. 40A–C). By contrast, alligatorids exhibit contact between the postorbital and both the quadrate and quadratojugal (Fig. 40G–I). Other taxa exhibit different combinations of these conditions. For example, whereas *Gavialis gangeticus* exhibits a quadratojugal-postorbital contact (106-1), but no quadrate-postorbital contact (105-0) (Fig. 40E), *Tomistoma schlegelii* exhibits the opposite conditions (106-0, 105-1) (Fig. 40F).

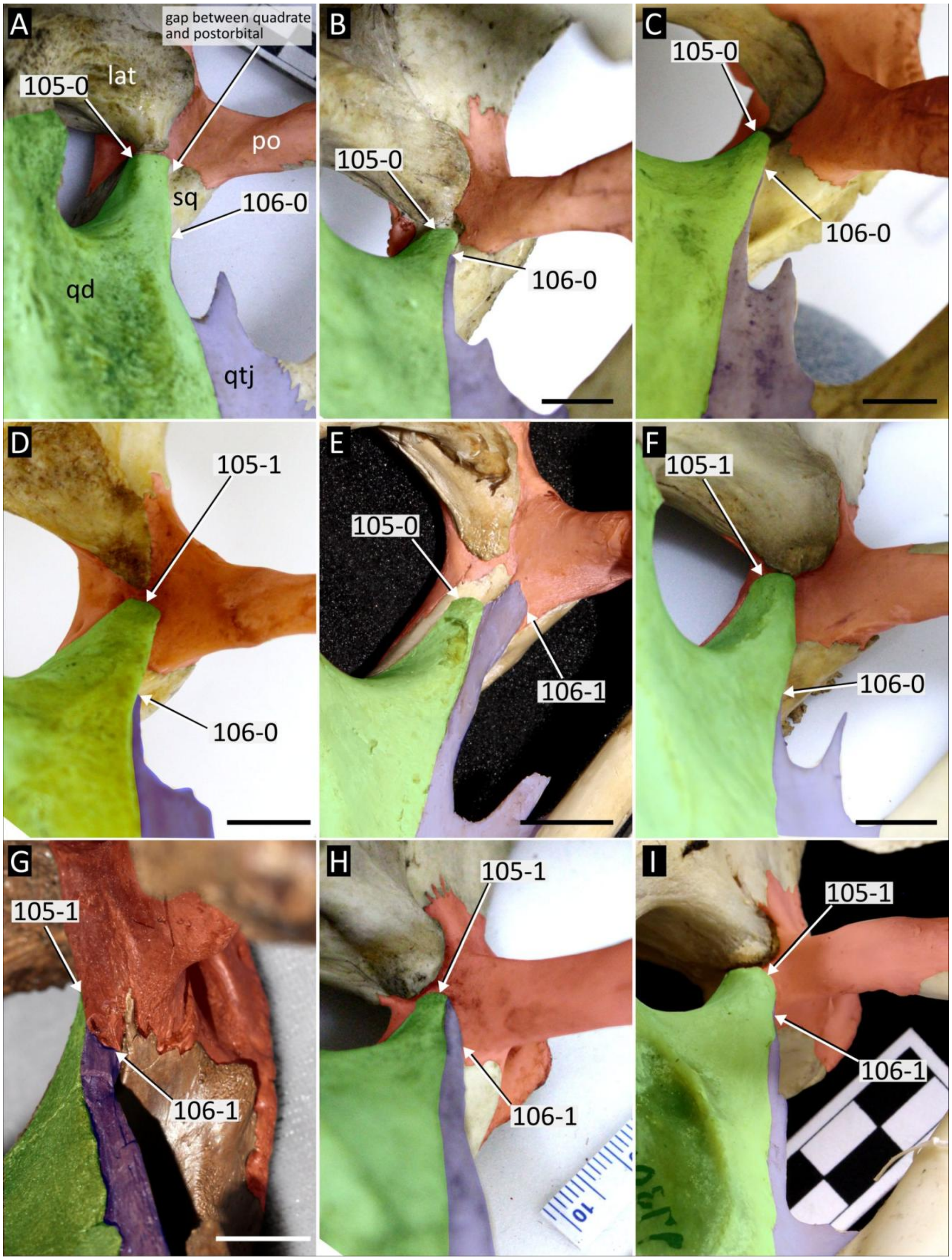

Figure 40: Ventromedial view of the dorsal corner of the postorbital showing sutural relationships of the postorbital (red), quadrate (green), and quadratojugal (blue). **A**, *Crocodylus moreletti* (NHMUK 1861.4.1.4); **B**, *Crocodylus siamensis* (NHMUK 1921.4.1.168); **C**, *Crocodylus palustris* (NHMUK 1897.12.31.1); **D**, *Mecistops cataphractus* (NHMUK 1924.5.10.1); **E**, *Gavialis gangeticus* (NHMUK uncatalogued); **F**, *Tomistoma schlegelii* (NHMUK 1894.2.21.1); G, *Brachychampsa montana* (UCMP 133901); **H**, *Alligator mississippiensis* (NHMUK 1873.2.21.1); **I**, *Caiman yacare* (AMNH 97300). Abbreviations: **lat**, laterosphenoid; **po**, postorbital; **qd**, quadrate; **qtj**, quadratojugal. All scale bars = 1 cm.

Squamosal

107. Squamosal, anterior divergence of dorsal and ventral rims of lateral groove: absent (0); present (1) (after Brochu, 1997a [84]).

Only minor modifications have been made to the wording of this character and scores are similar to previous studies (e.g. Brochu et al., 2012). The lateral groove of the squamosal is a narrow sulcus on the lateral cranial table edge that serves as an attachment site for external ear valve musculature (Fig. 41). In most crocodylians, the dorsal and ventral margins of the squamosal groove are either sub-parallel (Fig. 41A) or slightly taper anteriorly (Fig. 41D). By contrast, several (mostly longirostrine) crocodylians exhibit a dorsoventral expansion of the groove anteriorly, including *Thecachampsa sericodon* (Fig. 41), *Kentisuchus spenceri* (Fig. 41C), and *Piscogavialis jugaliperforatus* (Fig. 41E).

108. Squamosal, shape of the lateral cranial table edge, dorsal to the otic aperture: vertical, dorsal and ventral edges equally expanded laterally; (0) bevelled, ventral edge projects further laterally than dorsal edge (1) (after Lee and Yates, 2018 [81]).

The lateral cranial table margins slope prominently (i.e. they are bevelled) in *Hylaeochampsa vectiana* (NHMUK R177) and several (mostly longirostrine) crocodylians, including the "gavialoids" *Gavialis gangeticus* (NHMUK 1974.3009), *Piscogavialis jugaliperforatus* (Fig. 41E), and *Gryposuchus neoageus* (MLP 26-413), and the "tomistomines" *Kentisuchus spenceri* (Fig. 41C) and '*Tomistoma*' *dowsoni* (NHMUK PV R4769). This contrasts with the more commonly observed vertical lateral edge of the cranial table found in all extant alligatorids (Fig. 41A, C), crocodylids, and *Tomistoma schlegelii* (NHMUK 1894.2.21.1).

109. Squamosal, angle between dorsal profile of the paroccipital process and dorsal margin of the cranial table: $< 10°$ (approximately horizontal) (0); 10–50° (1); $> 50°$ (2) (after Lee and Yates, 2018 [88]) (ORDERED).

This character was modified from Lee and Yates (2018) by the addition of a state (109-0) and by ordering of the character. In most eusuchians, the dorsal profile of the paroccipital process is posteroventrally inclined, around 45° (109-1), as in *Hylaeochampsa vectiana* (NHMUK R177), most extant alligatorids (e.g. *Alligator mississippiensis*, Fig. 41A), crocodylids (e.g. *Crocodylus*), and "tomistomines" (e.g. *Kentisuchus spenceri*, Fig. 41C). In some crocodylians, the paroccipital process curves off abruptly to form a 90° angle between the dorsal and posterior edges of the squamosal (109-2), e.g. *Paleosuchus trigonatus* (Fig. 41D), *Osteolaemus tetraspis* (NHMUK 1862.6.30.5), and *Mekosuchus* (e.g. *M. sanderi*, QM F31166). A small number of crocodylians exhibit a sub-horizontal dorsal profile of the paroccipital process (109-0). This condition is prin-

cipally observed in "gavialoids", such as *Piscogavialis jugaliperforatus* (Fig. 41E), *Gryposuchus colombianus* (UCMP 41136), and *Gryposuchus neogaeus* (MLP 26-413).

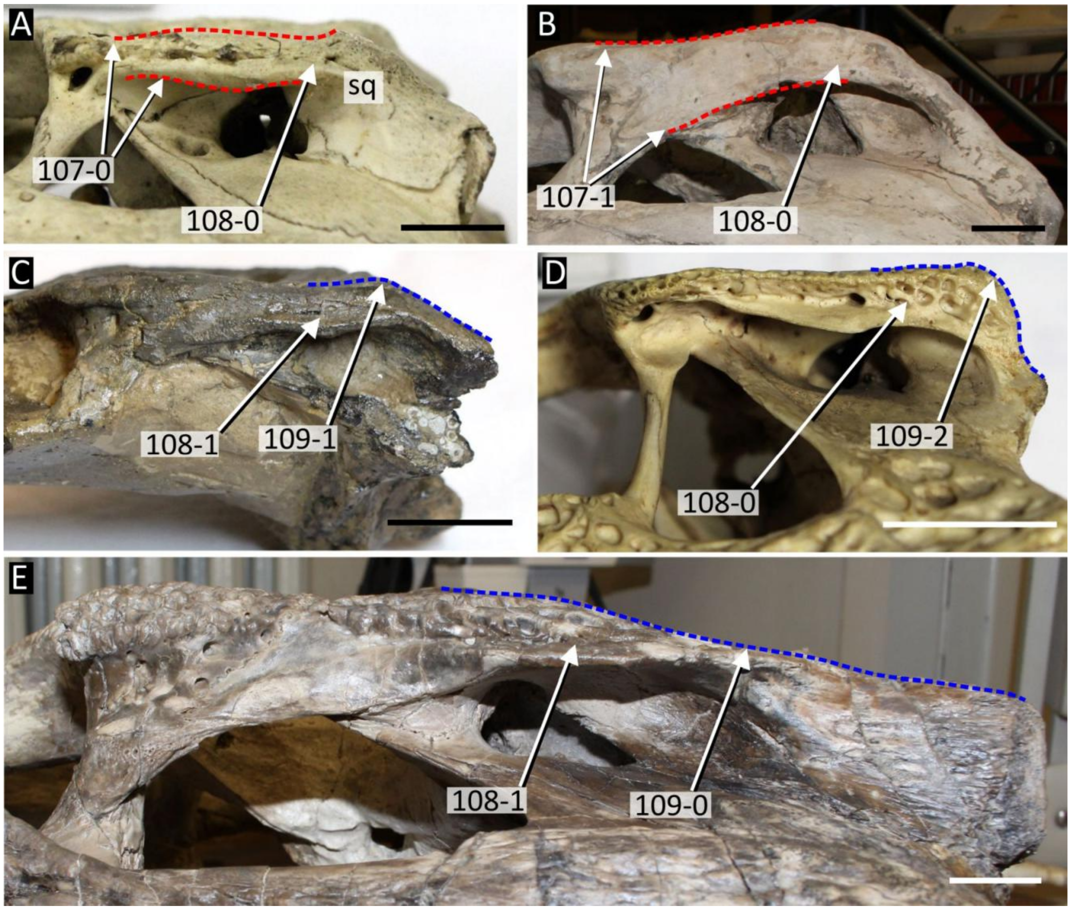

Figure 41: Left lateral view of the posterior cranium showing variation in lateral cranial table morphology in **A**, *Alligator mississippiensis* (NHMUK 1873.2.21.1); **B**, *Thecachmapsa sericodon* (USNM 25243); **C**, *Kentisuchus spenceri* (NMHUK PV R 38975); **D**, *Paleosuchus trigonatus* (NHMUK 1868.10.8.1, digitally reversed); **E**, *Piscogavialis jugaliperforatus* (SMNK 1282 PAL). Abbreviations: **sq**, squamosal. All scale bars = 2 cm.

110. Squamosal, posterolateral prongs: absent, or very short, barely exceeding the level of the posterior wall of the cranial table behind supratemporal fenestrae (0); long, exceeding the level of the posterior margin of the cranial table, less than half anteroposterior cranial table length (1); long, greater than or equal to half anteroposterior cranial table length (2) (after Brochu, 1997a [140]; Jouve et al., 2008 [140]; Jouve, 2016 [64]) (ORDERED).

Squamosal prongs are posterolateral projections of the squamosal that extend from the cranial table (Fig. 42). As originally formulated by Brochu (1997b), this character was binary, describing the presence or absence of squamosal prongs. Jouve (2016) introduced a third character state describing 'very long' squamosal prongs. This modification is followed, but the length of the squamosal prongs is measured in proportion to the anteroposterior cranial table length. Further-

more, the character is now ordered, as it describes the progressive lengthening of the squamosal prongs. *Bernissartia fagesii* (IRScNB 1538) and most crocodylians exhibit the intermediate condition (Fig. 42B). *Hylaeochampsa vectiana* (NHMUK R177), *Iharkutosuchus makadii* (Ösi et al., 2007), and *Paleosuchus* (Fig. 42A) exhibit the shortened condition. Highly elongated squamosal prongs occur exclusively in several longirostrine crocodylians (Jouve, 2016), including *Piscogavialis jugaliperforatus* (Fig. 42C), *Argochampsa krebsi* (NHMUK R36872), and *Gryposuchus colombianus* (UCMP 41136). Equally elongate prongs are newly recognised here in *Tomistoma cairense* (SMNS 50740) and *Tomistoma lusitanica* (Antunes, 1961).

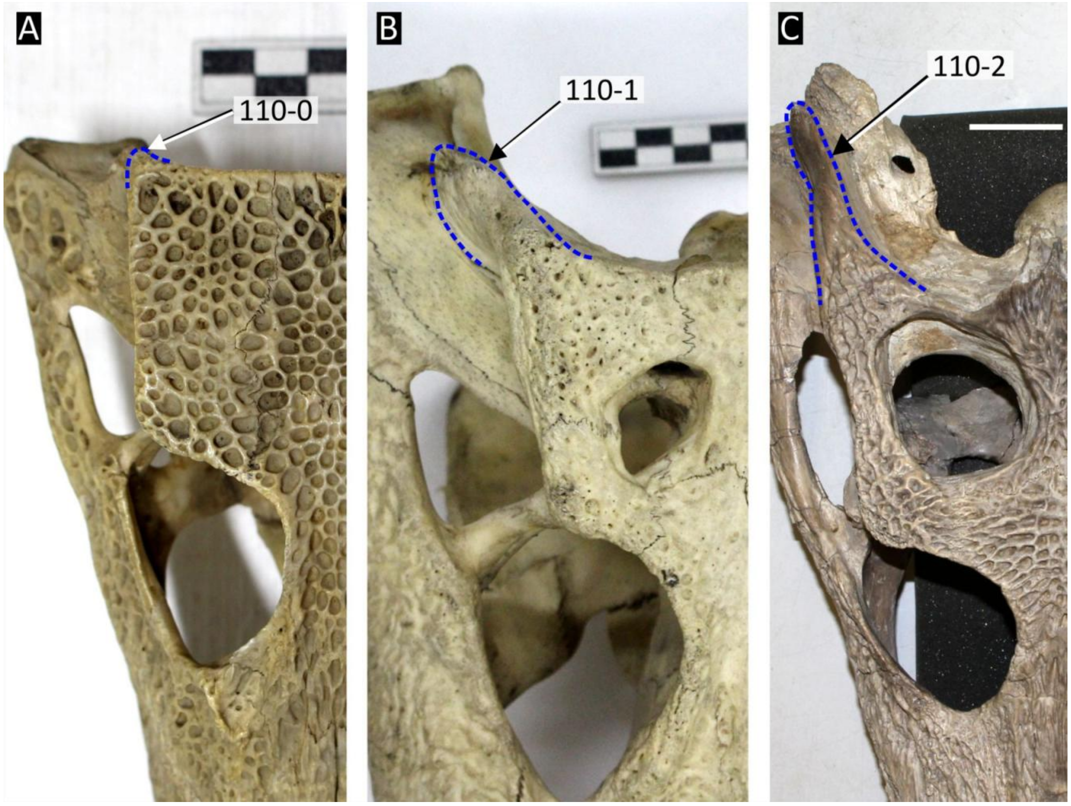

Figure 42: Dorsal view of the cranial table showing variation in length of the squamosal prongs (blue). **A**, *Paleosuchus trigonatus* (NHMUK 1868.10.8.1); **B**, *Alligator mississippiensis* (NHMUK 1873.2.21.1); **C**, *Piscogavialis jugaliperforatus* (SMNS 1282 PAL). Scale bar in C = 5 cm, all other scale bars = cm.

External Auditory Meatus

111. External auditory meatus, position of ventral margin: ventral to the level of the dorsal margin of infratemporal fenestra (0); level with or dorsal to the dorsal margin of the infratemporal fenestra (1) (new character, based on personal observations).

The ventral margin of the external auditory meatus is lower than the level of the dorsal apex of the infratemporal fenestra in almost all eusuchians (Fig. 43A, C). By contrast, in *Purussaurus*

neivensis (Fig. 43B), *Purussaurus brasiliensis* (UFAC 1403), and *Acresuchus pachytemporalis* (Fig. 43D), the external auditory meatus is positioned in a notably more dorsal position, beyond the dorsal margin of the infratemporal fenestra.

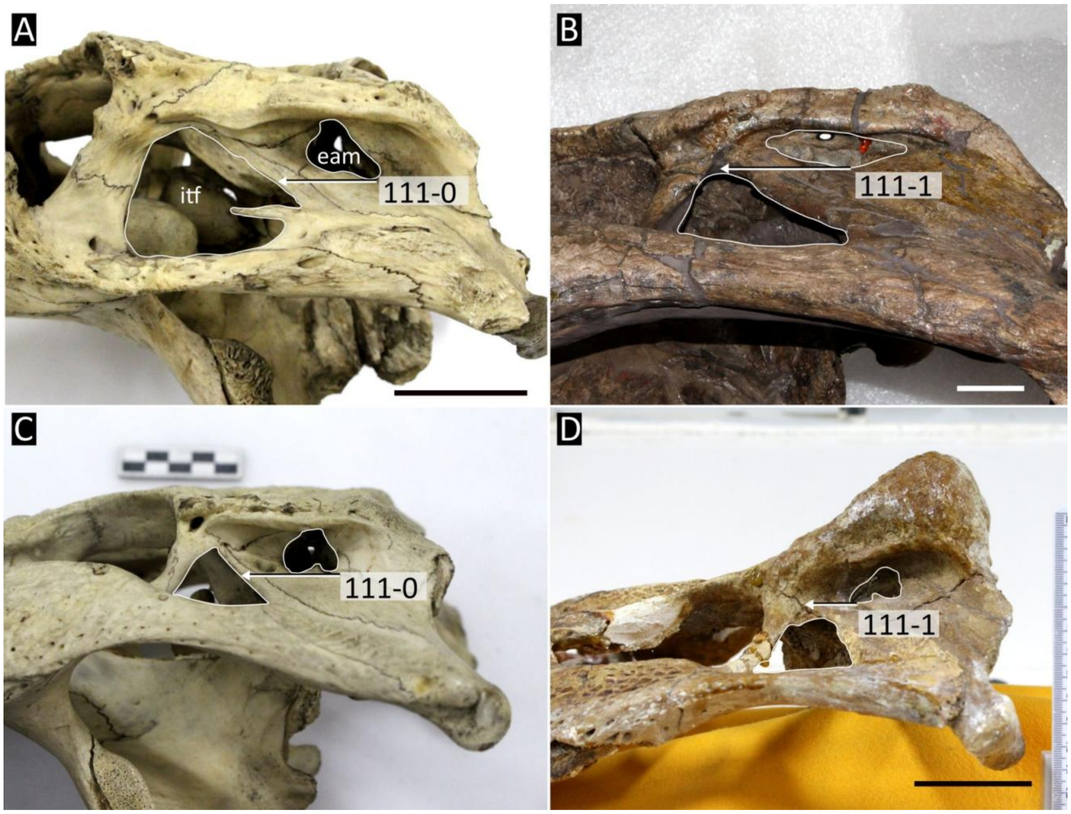

Figure 43: Lateral view of the cranium showing variation in dorsoventral height of the external auditory meatus relative to the infratemporal fenestra. **A**, *Gavialis gangeticus* (NHMUK); **B**, *Purussaurus neivensis* (UCMP 39704); **C**, *Alligator mississippiensis* (NHMUK 1873.2.21.1); **D**, *Acresuchus pachytemporalis* (UFAC 2507). Abbreviations: **eam**, external auditory meatus; **itf**, infratemporal fenestra. All scale bars = 5 cm.

112. Quadrate, sutural contact with squamosal posterior to external auditory meatus: present (0); absent, exoccipital separates squamosal and quadrate posterior to external auditory meatus (1) (after Brochu, 1997a [132]; Delfino et al., 2008a [132]; Lee and Yates, 2018 [106]).

In all crocodylians, the squamosal and quadrate are in sutural contact posterior to the external auditory meatus (EAM), enclosing the cranioquadrate canal (112-0) (Fig. 44C–F). In *Allodaposuchus precedens* (Fig. 44A), *Hylaeochampsa vectiana* (Fig. 44B), and several other non-crocodylian eusuchians, the squamosal and quadrate are not in contact in this region, laterally exposing the cranioquadrate canal and the exoccipital that floors it (112-1) (Buscalioni et al., 2001; Delfino et al., 2008a). The condition in *Bernissartia fagesii* (IRScNB 1538) is unknown.

Characters 113 to 115 describe variation in the posterior margin of the EAM, which can only be scored if the squamosal and quadrate are in sutural contact (112-1). Several earlier analyses im-

plemented these characters, without consideration for the absence of squamosal-quadrate contact in some taxa (e.g. Brochu, 1999; Brochu et al., 2012; Cidade et al., 2017; Salas-Gismondi et al., 2015). Other analyses included a modification used by Delfino et al. (2008a), in which characters 114 and 115 were each augmented with a character state, which effectively describes the absence of squamosal-quadrate contact (Delfino et al., 2008a; Iijima & Kobayashi, 2019; Jouve, 2016; Narváez et al., 2015). This modification was not included here following the application of reductive coding, and because the inclusion of an additional state describing the same anatomical feature in two characters would result in overweighting.

113. Squamosal, descending lamina extending anteriorly over quadrate ramus from paroccipital process: absent (0); present (1) (after Brochu, 1997a [150]).

In taxa that exhibit contact between the squamosal and quadrate, the squamosal may extend ventrally along the paraoccipital process as a descending lamina. This condition occurs in several osteolaemines, comprising *Osteolaemus tetraspis* (Fig. 44F), *Brochuchus pigotti* (NHMUK R7729), *Euthecodon armabourgi* (MNHN ZEL 001), and variably in *Voay robustus* (Brochu, 2007a). In all other crocodylians, there is no descending lamina, and the squamosal-quadrate suture is straight (Fig. 44C–E).

114. Quadrate-squamosal suture, intersection with external auditory meatus (EAM): extends dorsally along posterior margin of EAM (suture separated from posterior margin) (0); or extends only to posteroventral corner of EAM (suture incipiently contacts posterior margin) (1) (after Brochu, 1997a [132]).

As described by Brochu (1999) and following most earlier studies, the quadrate-squamosal suture intersects the posteroventral corner of the EAM in most alligatoroids (Fig. 44E), *Boverisuchus vorax* (FMNH PR 399), and *Trilophosuchus rackhami* (QM F16856), whereas it ascends the posterior margin in most other crocodylians (Fig. 44C–D) (Brochu et al., 2012; Iijima & Kobayashi, 2019; Jouve, 2016; Salas-Gismondi et al., 2015). In several caimanines, the suture ascends the posterior EAM margin for a short distance, e.g. *Caiman latirostris* (NHMUK 86.10.4.2, FMNH 9713), *Melanosuchus niger* (NHMUK 45.8.25.125), and *Paleosuchus trigonatus* (NHMUK 1868.10.8.1); however, as with previous authors, we regard the condition in these taxa as closer to the derived condition.

115. External auditory meatus, posterior margin shape: straight (0); invaginated (1) (after Brochu, 1997a [102]; Salisbury et al., 2006 [102]; Delfino et al., 2008a [102]).

In most crocodylians that exhibit contact between the quadrate and squamosal, the posterior wall of the EAM is infolded to form an anterior process (Fig. 44F). This condition occurs in most

extant crocodylids (Fig. 44D), alligatorids (Fig. 44E), and *Tomistoma schlegelii*. By contrast, the suture is straight in most "gavialoid" crocodylians, e.g. *Gavialis gangeticus* (Fig. 44C), as well as planocraniids, among other crocodylians.

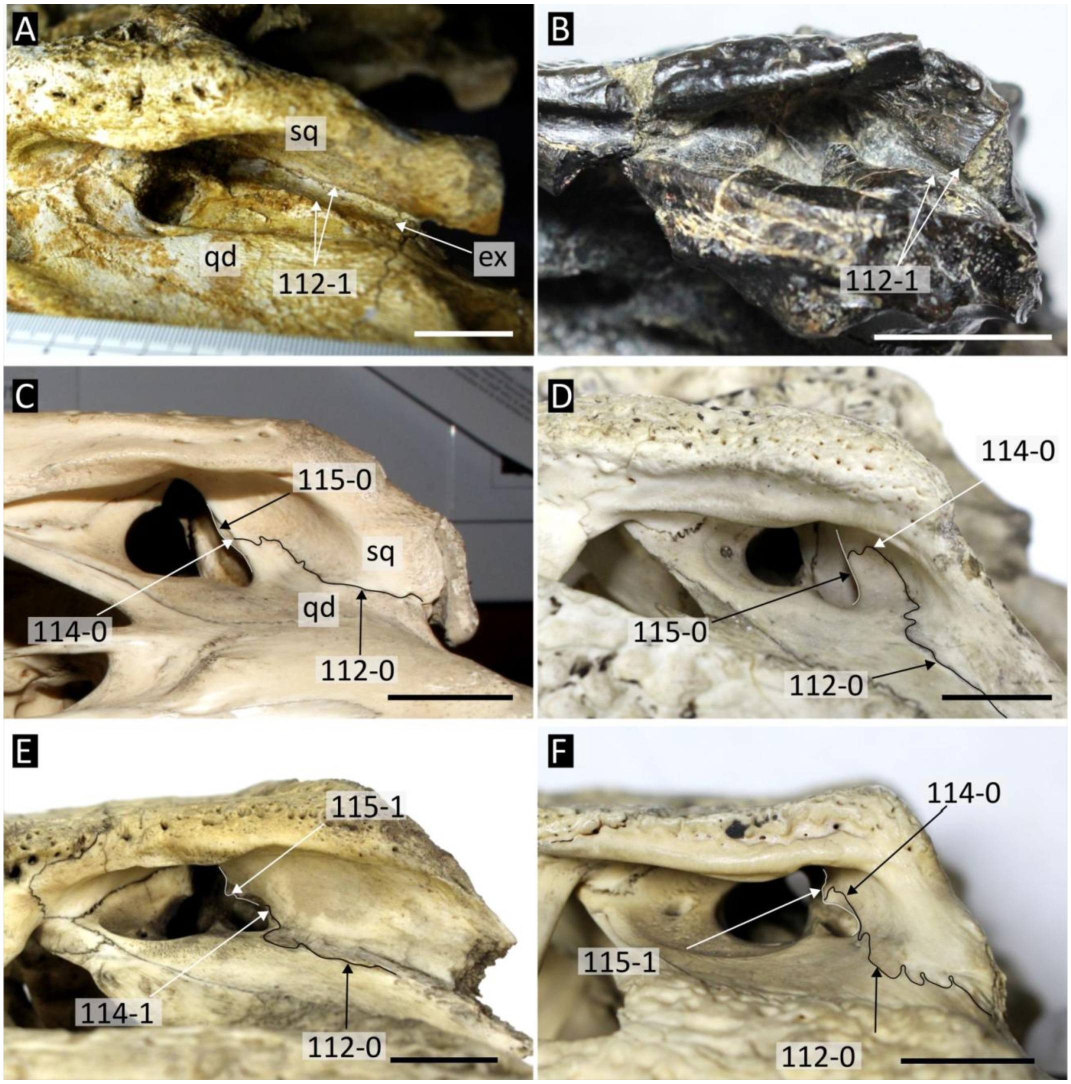

Figure 44: Sutural relationships of the external auditory meatus in Eusuchia. **A**, *Allodaposuchus precedens* (MMSVBN-12-10A); **B**, *Hylaeochampsa vectiana* (NHMUK PV R 177); **C**, *Gavialis gangeticus* (NHMUK 1974.3009); **D**, *Crocodylus niloticus* (NHMUK 1934.6.3.1); **E**, *Melanosuchus niger* (NHMUK 45.8.25.125); **F**, *Osteolaemus tetraspis* (NHMUK 1862.6.30.5). All scale bars = 2 cm.

Quadrate

116. Quadrate, foramen aereum size: small, diameter less than half dorsoventral height of medial hemicondyle (0); large, equal to or greater than half dorsoventral height of medial hemicondyle (1) (after Brochu, 2006 [165]; Brochu, 2011 [178]).

The quadratic foramen aerum is a circular opening on the dorsomedial margin of the quadrate condyle. This foramen accommodates an epithelial tube which runs towards a corresponding foramen on the articular (Brochu, 2006b). In most crocodylians, the quadratic foramen aerum is very small (Fig. 45R), but it is notably enlarged in *Eosuchus lerichei* (Fig. 45S) and *Eosuchus minor* (YPM 282).

117. Quadrate, foramen aereum position on posterior quadrate ramus: on dorsomedial corner (0); or on dorsal surface (1) (after Brochu, 1997a [121]).

The derived character state describes a dorsally positioned foramen aerum that has long been held as an alligatoroid synapomorphy (Brochu, 1999). Indeed, this condition occurs in all extant alligatorids such as *Alligator mississippiensis* (Fig. 45H), *Melanosuchus niger* (Fig. 45I), and *Caiman yacare* (Fig. 45J), as well as 'basal' alligatoroids such as *Diplocynodon hantoniensis* (Fig. 45F). The condition has also been recognised in some non-crocodylian eusuchians, such as *Allodaposuchus precedens* (Delfino et al., 2008a; Martin et al., 2016; Narváez et al., 2019) and *Lohuecosuchus megadontos* (Narváez et al., 2015). *Bernissartia fagesii* (IRScNB 1538) and most non-alligatoroid crocodylians have a medially positioned foramen (Fig. 45N, O). Character scores between this study and previous studies (e.g. Brochu et al., 2012; Lee & Yates, 2018; Narváez et al., 2016), are mostly in agreement, except that the dorsally positioned foramen aerum is newly recognised in two *Borealosuchus* species: *B. sternbergii* (Fig. 45G) and *B. formidabilis* (Erickson, 1976, fig.6).

118. Quadrate condyle, notch on the dorsal articular border: absent or small, restricted to dorsomedial edge of quadrate articular border (0); large, as an extensive indentation of the dorsal articular border, covering up to a third of the mediolateral width of the quadrate condyle (1); inset from dorsomedial edge of the condyle (2) (adapted from Brochu, 1997a [112]).

119. Quadrate condyle shape, dorsal and ventral margins: subparallel across length (sub-rectangular condyle) (0); medially tapering (1); constricted at mid-length (2); ventrally reflected medial hemicondyle (3) (adapted from Brochu, 1997a [112]).

Characters 118 and 119 attempt to capture the seemingly nebulous variation in the morphology of the quadrate condyle that was originally discretised in one multistate character: "*Quadrate with small, ventrally-reflected medial hemicondyle (0) or with small medial hemicondyle; dorsal notch for foramen aerum (1), or with prominent dorsal projection between hemicondyles (2), or with expanded medial hemicondyle (3)*" (Brochu, 1997b). There are several issues with the previous delimitation of this character, as well as scores in earlier datasets. Firstly, the original character describes morphological features that might not be homologous: the presence of a dorsal notch in

state 1; the presence of a dorsal projection between the hemicondyles in state 2; and the shapes of the medial and lateral hemicondyles in states 0, 1, and 3. Furthermore, examination of taxa assigned to each state reveals differences in morphology. Taxa usually scored for character state 0 in the original character such as *Gavialis gangeticus*, *Eogavialis africanum*, *Eosuchus lerichei*, and *Borealoschus*, do not share the same morphology of the quadrate condyle. *Gavialis gangeticus* has a rectangular quadrate condyle (Fig. 45R), with indistinct medial and lateral hemicondyles, and little to no notch at maturity. By contrast, *Borealosuchus sternbergii* has a large notch on the medial hemicondyle (Fig. 45G), similar to the condition in *Alligator* (Fig. 45E, H) and *Diplocynodon hantoniensis* (Fig. 45F). *Eogavialis africanum* also differs (Fig. 45Q), with a dorsoventral constriction in the quadrate condyle similar to *Voay robustus* (Fig. 45M), *Crocodylus* (Fig. 45N), and *Eosuchus lerichei* (Fig. 45S). Taxa scored for character state 112-1 of Brochu (1997a) are almost entirely alligatoroids, including *Diplocynodon*, *Caiman*, *Melanosuchus*, and *Alligator*, as well as some mekosuchines (Brochu et al., 2012). Although it is agreed that the medial hemicondyle in all alligatoroids bears a notch (Fig. 45E–F, I–I), the morphology of the notch is variable. In all extant caimanines, there is a small dorsal notch, inset from the medial edge of the quadrate condyle (Fig. 45I–J). This contrasts with the condition in *Alligator* (Fig. 45E, H), *Diplocynodon hantoniensis* (Fig. 45F) and several mekosuchines (Fig. 45C–D), in which the notch is wide and deep, reaching up to one third of the quadrate condyle width. Character state 112-2 (Brochu, 1997a) describes "*a prominent dorsal projection between hemicondyles*" that is shared only by *Boverisuchus vorax* and *Boverisuchus magnifrons* in the dataset of Brochu et al. (2012). The quadrate condyles of *Boverisuchus* were figured by Brochu (2012, fig.14), but their morphology is considered more similar to the condition in mekosuchines and alligatoroids here (Fig. 45C–D). The morphology described in character state 112-3, and the taxa assigned to this character state, are mostly agreed on here. The dorsoventrally expanded lateral hemicondyle is well expressed in most crocodyloids, resulting in an hour-glass shaped quadrate condyle (Fig. 45M–N). These observations have led to the division of the original character into two multistate characters: one describing the morphology of the notch (118) and the other describing the shapes of the medial and lateral hemicondyles (119). Neither character is ordered. The notch on the quadrate condyle is extremely small (118-0) in all *Crocodylus* species (118-0) (Fig. 45N), *Gavialis gangeticus* (Fig. 45R), and *Tomistoma schlegelii* (Fig. 45O). In caimanines, the notch becomes medially inset and remains small (118-2), e.g. *Melanosuchus niger* (Fig. 45I). The notch is deep and wide (118-1) in *Alligator olseni* (Fig. 45E), *Diplocynodon hantoniensis* (Fig. 45F), *Borealosuchus sternbergii* (Fig. 45G), *Trilophosuchus rackhami* (Fig. 45D), and *Boverisuchus vorax* (Fig. 45A). Character 119 describes four morphotypes of the quadrate: sub-rectangular (119-0), e.g. *Boverisuchus vorax* (Fig. 45A and *Alligator* (Fig. 45E); medially tapering (119-1), e.g. *Melanosuchus* (Fig. 45I), *Caiman yacare*

(Fig. 45J), *Protocaiman peligrensis* (Fig. 45K), and *Procaimanoidea utahensis* (Fig. 45L); constricted at the mid-length (119-2), e.g. *Voay robustus* (Fig. 45M), *Crocodylus acutus* (Fig. 45N), *Tomistoma schlegelii* (Fig. 45O), and *Eogavialis africanum* (Fig. 45Q); and ventrally reflected, e.g. *Piscogavialis jugaliperforatus* (Fig. 45T).

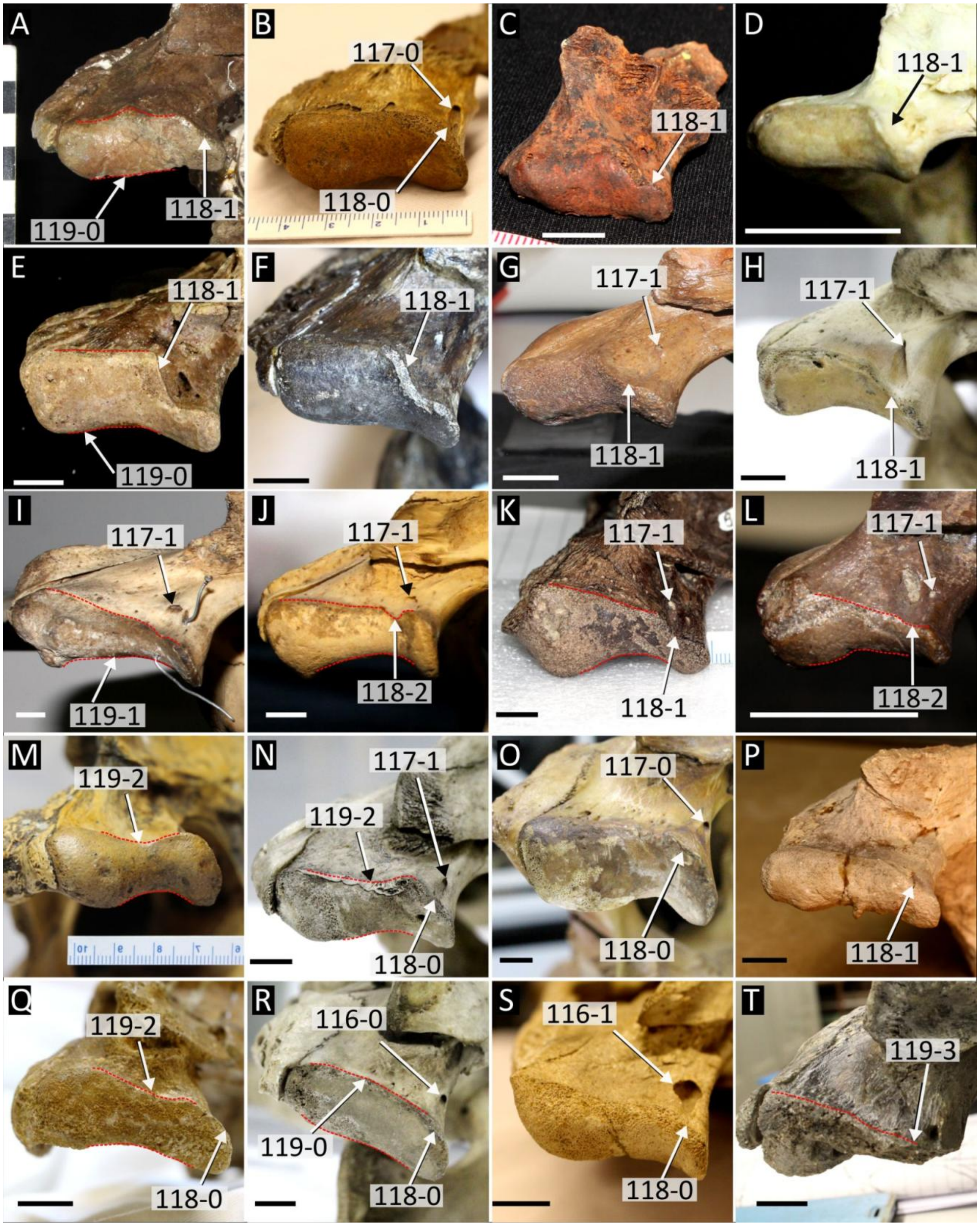

Figure 45: Posterior view of the quadrate condyle showing variation in condyle morphology in selected crocodylians. **A**, *Boverisuchus vorax* (FMNH PR 399); **B**, *Asiatosuchus depressifrons* (IRSNB R 0251); **C**, *Mekosuchus inexpectatus* (MNHN NCP 06); **D**, *Trilophosuchus rackhami* (QM F16856); **E**, *Alligator olseni* (MCZ uncatalogued); **F**, *Diplocynodon hantoniensis* (NHMUK 30392); **G**, *Borealosuchus sternbergii* (USNM V6533) **H**, *Alligator mississippiensis* (NHMUK 1873.2.21.1); **I**, *Melanosuchus niger* (NHMUK 45.8.25.125); **J**, *Caiman yacare* (MACN uncatalogued); **K**, *Protocaiman peligrensis* (MLP 80X-10-1), **L**, *Procaimanoidea utahensis* (USNM V 15996); **M**, *Voay robustus* (NHMUK R 36685); **N**, *Crocodylus acutus* (NHMUK 1975.997) **O**, *Tomistoma schlegelii* (NHMUK 1894.2.21.1); **P**, *Tomistoma cairense* (SMNS 50739); **Q**, *Eogavialis africanum* (NHMUK PV R3108, digitally reversed); **R**, *Gavialis gangeticus* (NHMUK 1974.3009); **S**, *Eosuchus lerichei* (IRSNB R 49); **T**, *Piscogavialis jugaliperforatus* (SMNK 1282 PAL). All scale bars = 1 cm.

120. Quadratojugal, extent over lateral surface of posterior quadrate ramus: covers entire lateral surface (0); notch in quadratojugal, exposing quadrate ventrolaterally (1) (new character, based on personal observations).

In *Bernissartia fagesii* (IRScNB 1538) and most eusuchians, the jugal and quadratojugal extend posteriorly to conceal the lateral surface of the quadrate condyle (Fig. 46A, C). By contrast, a small gap is left where the quadrate remains exposed in all extant alligatorids (Fig. 46B, D). Several fossil alligatoroids also exhibit exposure of the quadrate here, including *Brachychampsa montana* (UCMP 133901), *Navajosuchus mooki* (MCZ 8381), *Procaimanoidea utahensis* (USNM 15996), and *Protocaiman peligrensis* (MLP 80X-10-1).

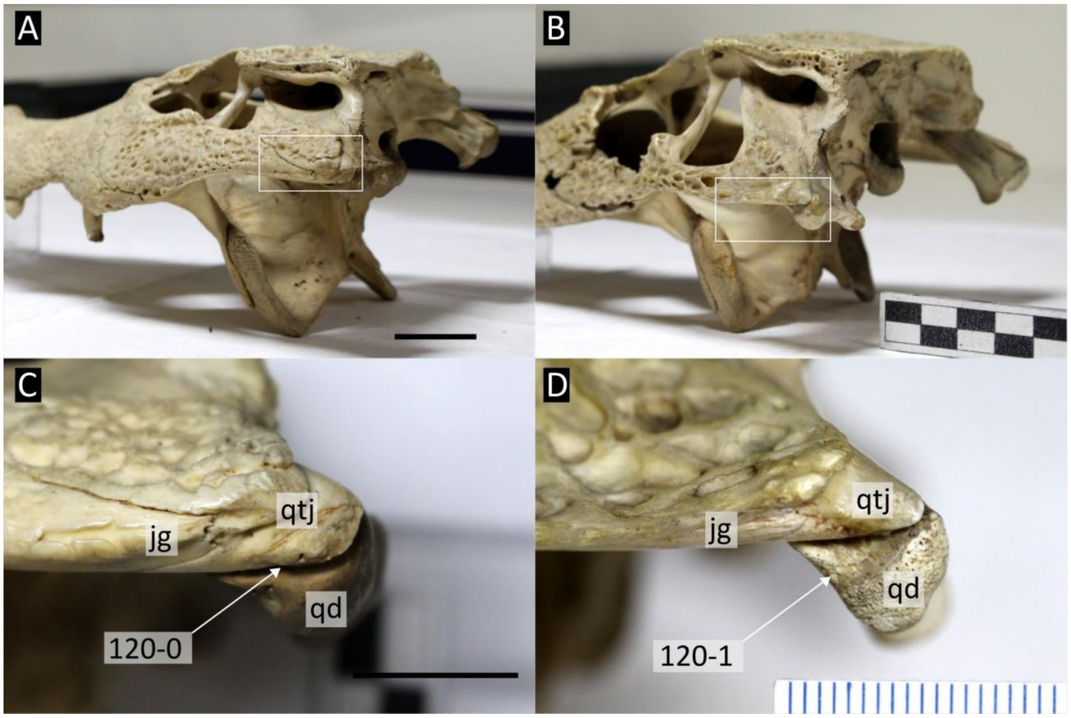

Figure 46: Lateral view of the quadrate ramus, showing variation in exposure of the quadrate beneath the quadratojugal. **A**, *Osteolaemus tetraspis* (NHMUK 1862.6.30.5); **B**, *Paleosuchus trigonatus* (NHMUK 1868.10.8.1). All scale bars = 2 cm.

121. Quadrate, posterior ramus length: distance between posterior margin of quadrate condyle and the level of the anterior margin of the occipital condyle, less than quadrate condyle mediolateral width (0); equal to or greater than quadrate condyle mediolateral width (1) (after Buscalioni et al., 2011 [184]).

The posterior ramus of the quadrate is anteroposteriorly long in most eusuchians, exceeding the posterior margin of the cranial table by at least the width of the quadrate condyle (Fig. 47A). By contrast, in *Bernissartia fagesii* (IRScNB 1538), *Isisfordia duncani* (QM F44320), and *Hylaeochampsa vectiana* (Fig. 47B), the quadrate ramus is short, barely exceeding the posterior edge

of the cranial table at most. The same condition occurs in some crocodylians, including *Alligator mcgrewi* (AMNH FAM 8700) and *Trilophosuchus rackhami* (QM F16856).

122. Exoccipital, extent on dorsal surface of quadrate ramus: small, not reaching articular border of quadrate condyle (0); large, extending to border of quadrate condyle (1) (new character, based on personal observations).

The exoccipital is minimally exposed on the dorsal surface of the posterior quadrate ramus in *Bernissartia fagesii* (IRScNB 1538) and nearly all eusuchians (Fig. 47A). However, in *Hylaeochampsa vectiana* (Fig. 47B), and *Iharkutosuchus makadii* (Mateus et al., 2019, fig.S14), the quadrate exoccipital suture extends over the dorsal surface of the quadrate ramus, such that the exoccipital reaches the border of the quadrate condyle.

123. Exoccipital, posterior projection of the paroccipital process: absent (0); present (1) (after Brochu, 1997a [141]).

Clark and Norell (1992) described a large protuberance positioned medial to the cranioquadrate canal in *Hylaeochampsa vectiana* (Fig. 47B). A similar process was subsequently described in several non-crocodylian eusuchians, such as *Allodaposuchus precedens* (Buscalioni et al., 2001), *Iharkutosuchus makadii* (Ösi, 2008), and *Lohuecosuchus megadontos* (Narváez et al., 2015). Furthermore, scores in existing matrices indicate that it occurs in *Bernissartia fagesii* and *Acynodon iberoccitanus* (e.g. Brochu et al., 2012; Jouve, 2016), which is agreed upon here. A protuberance or lamina occasionally occurs in large individuals of some crocodylians (Clark & Norell, 1992), but it is never as prominent as in *Hylaeochampsa*. Small differences occur in the morphology of the protuberance; for example, it is more of a ridge in *Allodaposuchus precedens* (Buscalioni et al., 2001, fig.10; Delfino et al., 2008a), but a discrete boss in *Hylaeochampsa* (NHMUK R177). These differences are not consistently found in enough taxa to allow further categorisations of the morphology.

124. Quadrate, paroccipital process, distance between distal tip of paroccipital process and distal end of the quadrate condyle: less than the maximum mediolateral width of the quadrate condyle (0); equal to or greater than the maximum mediolateral width of the quadrate condyle (1) (after Lee and Yates, 2018 [111]).

In most crocodylians, the paroccipital process extends towards the posterior end of the quadrate ramus, terminating shortly before the quadrate condyle (Fig. 48A). As recognised by Lee and Yates (2018), several crocodylians exhibit a notably wider gap between the distal tip of the paroccipital process and the distal tip of the quadrate ramus, e.g. *Australosuchus clarkae* (Fig. 48B). Whereas Lee and Yates (2018) scored the derived condition exclusively in a series of mekosuchines

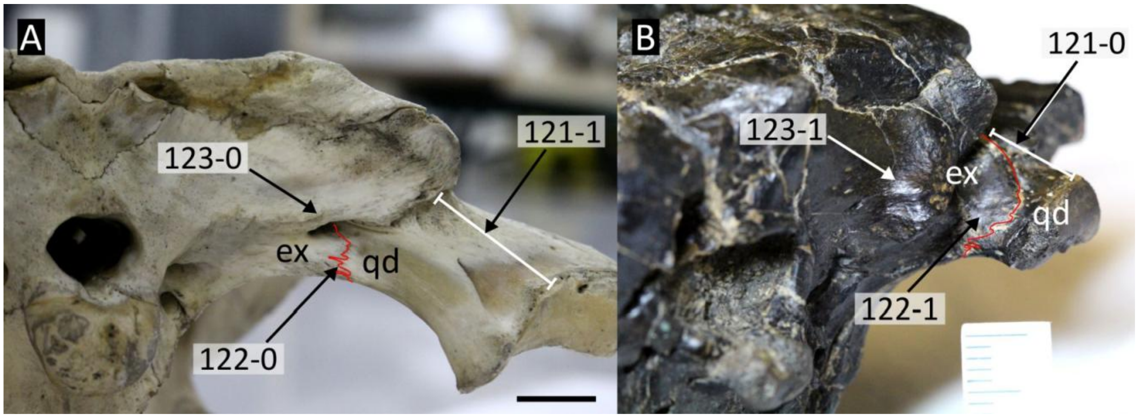

Figure 47: Posterolateral view of the occiput in **A**, *Alligator mississippiensis* (NHMUK 1873.2.21.1); and **B**, *Hylaeochampsa vectiana* (NHMUK PV R 177). Abbreviations: **ex**, exoccipital; **qd**, quadrate. Scale bar = 1 cm.

(*Palimnarchus gracilis*, *Baru*), here it is recognised in a broader sample of crocodylians, including alligatoroids, such as *Mourasuchus arendsi* (UFAC 2515) and *Alligator mississippiensis* (NHMUK 68.2.12.6), and "tomistomines", e.g. *Thecachampsa sericodon* (USNM 24938).

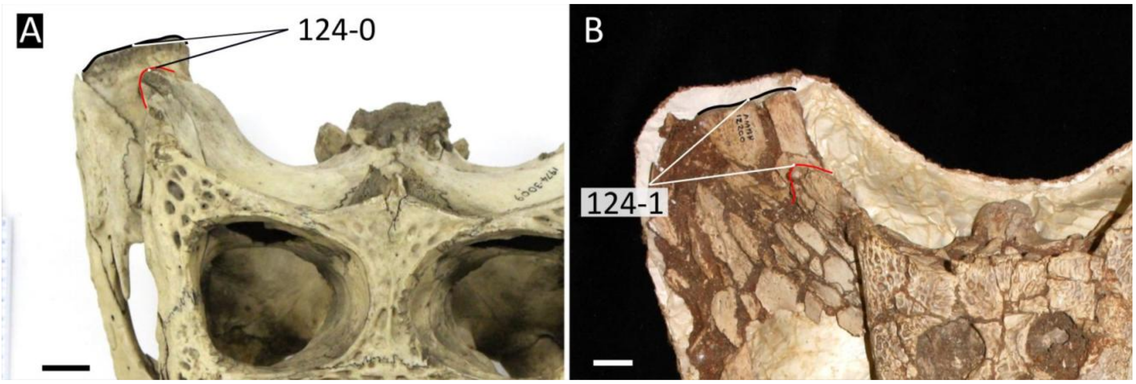

Figure 48: Dorsal view of the posterior end of the cranium showing variation in posterior extent of the paroccipital process on the quadrate ramus. **A**, *Gavialis gangeticus* (NHMUK 1974.3009); **B**, *Australosuchus clarkae* (AMNH 12200). Scale bars = 2cm.

Exoccipital

125. Exoccipital, paroccipital process dorsal margin: squamosal-exoccipital suture sub-horizontal (0); dorsolaterally directed (1) (new character, adapted from Clark and Norell, 1992).

Clark and Norell (1992) noted that the dorsolateral margin of the paroccipital process in *Hylaeochampsa vectiana* (NHMUK R177) curves dorsolaterally along the squamosal-exoccipital suture (Fig. 49B). A similar condition occurs in *Iharkutosuchus makadii* (Mateus et al., 2019, fig.S14). This differs to the condition in *Bernissartia fagesii* (IRScNB 1538) and all other eu-

suchians examined here, wherein the squamosal-exoccipital suture is straight and sub-horizontal (Fig. 49A).

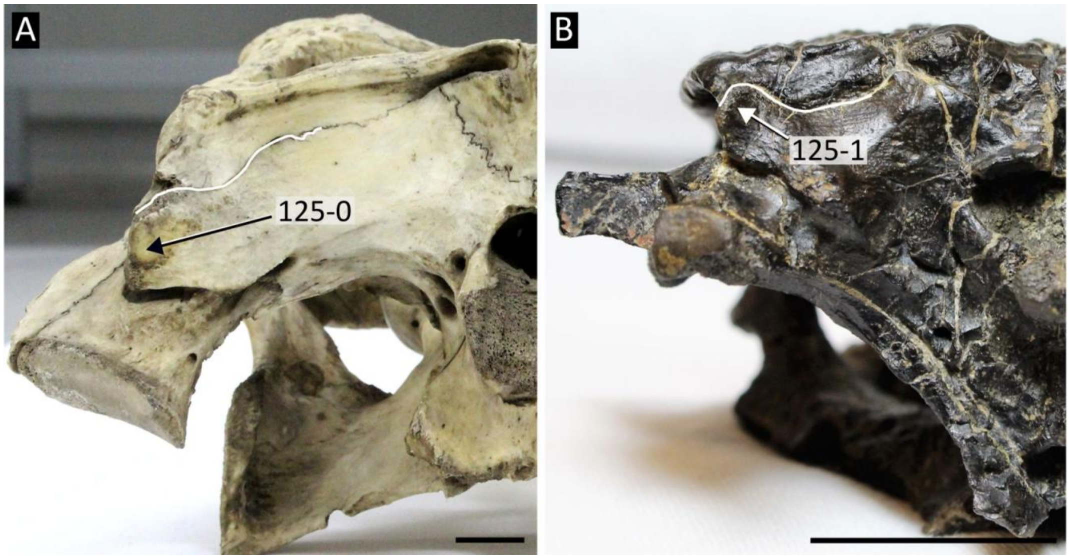

Figure 49: Occipital view of the cranium showing variation in orientation of the paroccipital process in. **A**, *Gavialis gangeticus* (NHMUK 1974.3009); **B**, *Hylaeochampsa vectiana* (NHMUK PV R 177). All scale bars = 2cm.

126. Lateral carotid foramen, proximity to metotic foramen: separated (positioned ventral to metotic foramen) (0); adjacent to the metotic foramen (1) (new character, based on personal observations).

Several foramina pierce the exoccipital lateral to the foramen magnum in crocodylians (Fig. 50). Typically, there are two medially positioned openings for the hypoglossal nerves (CN XII), lateral to which is the much larger metotic foramen, which houses CN IX–XI (Bona & Desojo, 2011; Iordansky, 1973). The lateral carotid foramen is distantly separated and ventral to the metotic foramen in most crocodylians (Fig. 50A). By contrast, the lateral carotid foramen in several "gavialoids" is adjacent to the metotic foramen, separated by a thin wall. This latter condition occurs in *Gavialis gangeticus* (Fig. 50B), *Gryposuchus neogaeus* (Fig. 50C), *Gryposuchus colombianus* (UCMP 38358), *Eogavialis africanum* (YPM 6263), and *Piscogavialis jugaliperforatus* (SMNK 1282 PAL). It is also present in some non-crocodylian taxa such as *Hylaeochampsa vectiana* (NMHUK R177) and the 'Glen Rose Form' (MCZ 4384).

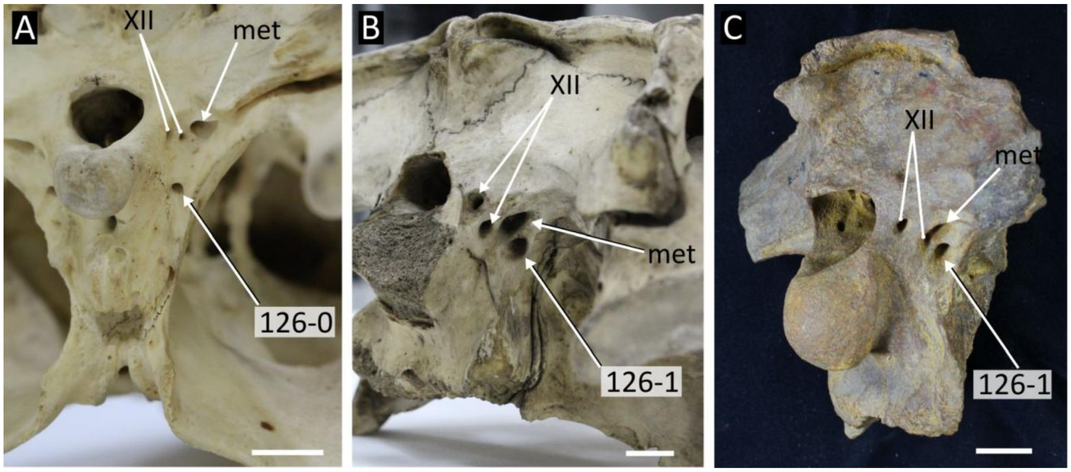

Figure 50: Proximity of the lateral carotid foramen relative to the metotic foramen in selected crocodylian taxa. **A**, *Caiman latirostris* (NHMUK 1897.12.31.1); **B**, *Gavialis gangeticus* (NHMUK 1974.3009); and **C**, *Gryposuchus neogaeus* (MLP 68-IX-V-1) (digitally reversed). Abbreviations: **met**, metotic foramen; **XII**, foramen for cranial nerve XII. All scale bars = 2 cm.

127. Exoccipitals, contact with basioccipital tubera: absent (0); present (1) (after Norell, 1988 [20]; Clark, 1994 [57, 60]; Brochu, 1997a [151]).

Basioccipital tubera refer to the rugose ventral and ventrolateral surfaces of the basioccipital. In most crocodylians, the exoccipitals suture laterally to the basioccipital and do not extend ventrally to contact the tubera (Fig. 51A–B). An alternative condition is exemplified by *Gavialis gangeticus*, in which the exoccipitals have long descending processes that contact the tubera (Fig. 51C–D). This condition also occurs in several additional "gavialoids", including *Eogavialis africanum* (YPM 6263) and *Gryposuchus neogaeus* (MLP 68-IX-V-1). Brochu (1997b) included an additional state

in his original formulation of the character, which described slender ventral processes that "participate in basioccipital tubera", which was scored in most caimanines. However, Brochu (1999) later noted that these processes do not actually contact the tubera in Caimaninae, but only extend slightly further ventrally than most other crocodylians. The description of these processes as slender is considered vague herein. Furthermore, the descending processes of caimanines examined here (e.g. *Caiman latirostris*: NHMUK 86.10.4.2; *Caiman crocodilus*: USNM 69812; *Melanosuchus niger*: NHMUK 1872.6.4.1) do not appear more slender than other taxa which lack an exoccipital-tubera contact. As a result, this character state has been removed and taxa formerly assigned to this state are now scored as lacking contact between the basioccipital tubera and exoccipital.

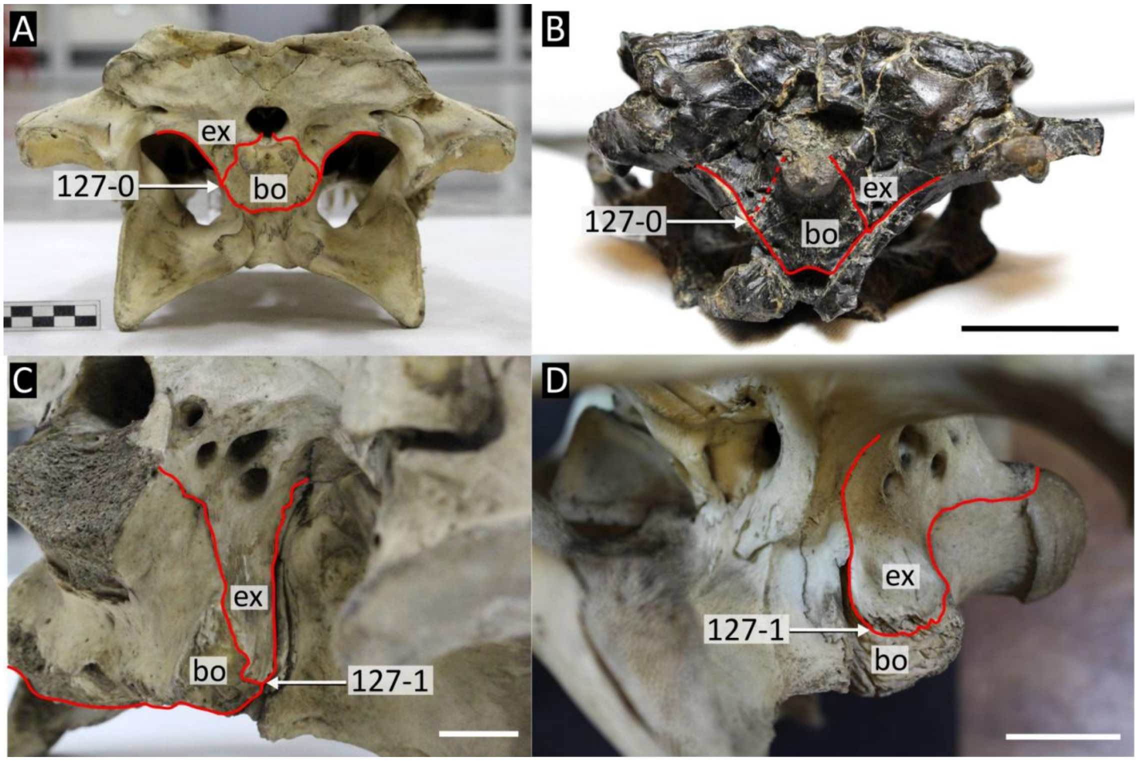

Figure 51: Ventral extent of the exoccipitals in selected crocodylian taxa. **A**, *Alligator mississippiensis* (NHMUK 1873.2.21.1); **B**, *Hylaeochampsa vectiana* (NHMUK PV R 177); **C**, *Gavialis gangeticus* (NHMUK 1974.3009) in posterolateral view; **D**, *Gavialis gangeticus* (NHMYK uncatalogued) lateral view of braincase. Abbreviations, **ex**, exoccipital; **bo**, basioccipital. All scale bars = 2 cm.

128. Exoccipitals, posteroventral inclination: absent, occiput vertical and not visible in dorsal view (0); present, occiput inclined posteriorly, visible in dorsal view (1) (after Hua and Jouve, 2004 [167]; Jouve et al., 2008 [167]).

When viewed dorsally, the occipital surface of most crocodylians is concealed, as a result of the vertical orientation of the exoccipitals that form much of the occipital surface (Fig. 52A, C). In rare instances, the exoccipitals are steeply inclined posteriorly, such that they are visible in

dorsal view. The latter condition is exhibited exclusively in taxa recovered as "gavialoids" in most analyses, such as *Gavialis gangeticus* (Fig. 52B, D), *Gryposuchus neogaeus* (MLP 26-413), and *Piscogavialis jugaliperforatus* (SMNK 1282 PAL). By contrast with the scores in Jouve (2016), the condition is considered absent in *Thoracosaurus isorhynchus* (MNHN.F.MTA 61), *Borealosuchsus sternbergii* (USNM 6533, UCMP 126099), and *Borealosuchus formidabilis* (Erickson, 1976, fig.4).

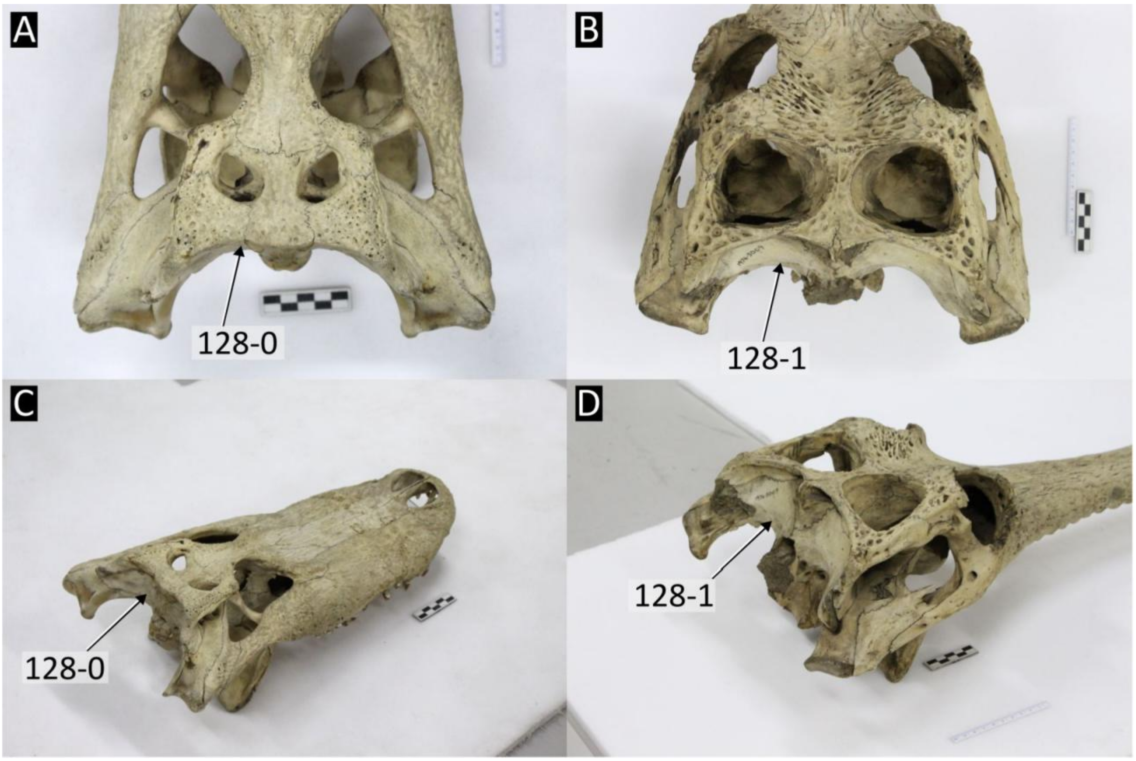

Figure 52: Variation in orientation of the occiput in *Alligator mississippiensis* (NHMUK 1873.2.21.1) (**A**, **B**); and *Gavialis gangeticus* (NHMUK 1974.3009) (**C**, **D**).

Basioccipital

129. Basioccipital, orientation of lateral margins of ventral basioccipital plate: parallel or ventrally convergent (0); ventrally divergent (1) (after Jouve, 2004 [176]; Jouve et al., 2008 [189]; Salas-Gismondi et al., 2015 [196]).

The lateral margins of the basioccipital are parallel for a short distance before converging ventrally in most eusuchians (Fig. 53A). However, some crocodylians, mainly "gavialoids", exhibit a differnet condition, in which the lateral margins flare ventrally, e.g. *Gavialis gangeticus* (Fig. 53B), *Eogavialis africanum* (NHMUK R3108), and *Piscogavialis jugaliperforatus* (SMNK 1282 PAL). Flaring basioccipital margins are additionally present in some species of the caimanine genus, *Mourasuchus*, including *M. arendsi* (UFAC 2515) and *M. amazonensis* (UFAC 1424).

130. Basioccipital, dorsoventral height of ventral plate exposed below occipital condyle relative to occipital condyle height: greater or equal in height (0); shorter (1) (adapted from Jouve, 2004 [197]; Jouve et al. 2008 [187]).

Most crocodylians exhibit a basioccipital that is dorsoventrally tall, reflecting the verticalisation of the cranium that is common to most eusuchians (Tarsitano et al., 1989) (Fig. 53A). Variation in height of the basioccipital was discretised by previous authors using vague terms such as 'short' and 'tall' (Jouve, 2004; Jouve et al., 2008). Here the morphology is quantified using the relative dorsoventral height of the portion of the basioccipital below the occipital condyle (basioccipital plate) to that of the occipital condyle. The basioccipital plate is shorter than the occipital condyle in *Gavialis gangeticus* (Fig. 53B), *Gryposuchus* (e.g. *G. neogaeus*, MLP 68-18-5-1), *Eogavialis africanum* (NHMUK R3108), and *Thoracosaurus isorhynchus* (MNHN.F.MTA 61). In addition to these "gavialoids", a shortened basioccipital plate occurs in *Mourasuchus* (e.g. *M. atopus*, UCMP 38012), *Toyotamaphimeia machikanensis* (Kobayashi et al., 2006) and, despite its otherwise verticalised cranium, *Alligator mississippiensis* (e.g. NHMUK 68.2.12.6).

131. Basioccipital and ventral portion of exoccipital (otoccipital), orientation (at maturity): inclined anteriorly (0); vertical (1) (after Gomani, 1997 [32]; Hua and Jouve, 2004 [167]; Salisbury et al., 2006 [174]; Pol et al., 2009 [112]; Brochu et al., 2012 [168]).

In extant hatchling crocodylians, the ventral occipital surface formed by the exoccipitals and basioccipital is strongly inclined anteriorly (Tarsitano et al., 1989). With maturity, the occipital surface becomes vertical, which is common to most eusuchians (Fig. 53D). A small number of taxa studied here that are considered ontogenetically mature also exhibit this condition, including *Trilophosuchus rackhami* (QM F16856), *Shamosuchus djadochtaensis* (Pol et al., 2009), the 'Glen Rose Form' (MCZ 4384), and *Isisfordia duncani* (QM F44320).

132. Basioccipital, sagittal crest on ventral plate: present (0); absent (1) (after Jouve, 2004 [185]; Jouve et al., 2008 [180]).

The ventral plate of the basioccipital bears a midline crest in most eusuchians, including alligatorids (e.g. *Alligator mississippiensis*, Fig. 53E; *Caiman latirostris*, NHMUK 86.10.4.2; and *Paleosuchus trigonatus*, NHMUK 1868.10.8.1), crocodylids (e.g. *Osteolaemus tetraspis*, NHMUK 1862.6.30.5 and *Crocodylus porosus*, QM J47448), and *Borealosuchus sternbergii* (USNM 6533). Less commonly, the sagittal crest is absent, which mostly occurs in "gavialoids", e.g. *Gavialis gangeticus* (Fig. 53F), *Gavialis lewisi* (YPM 3226), *Gryposuchus neogaeus* (MLP 68-18-5-1), but also '*Tomistoma*' *dowsoni* (NHMUK R4769).

133. Basioccipital, concavity on ventral margin, posterior to median eustachian foramen: absent (0);

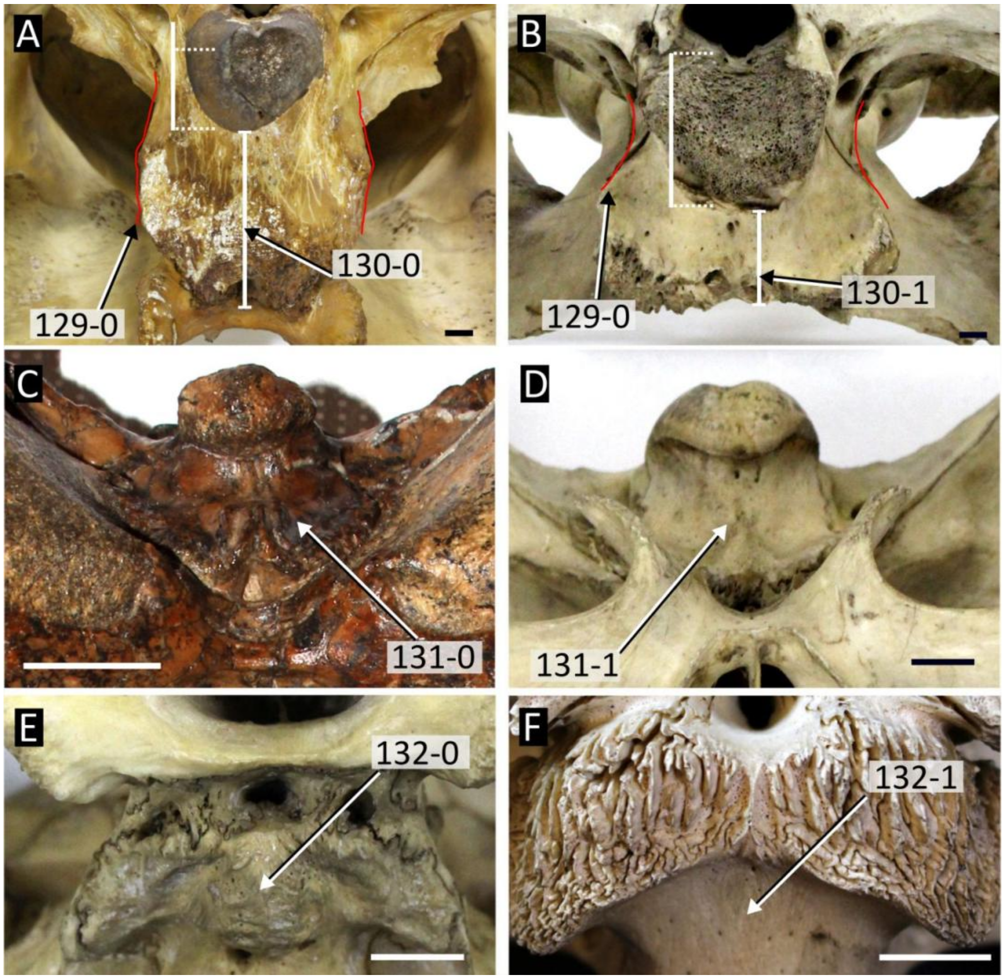

Figure 53: Morphology of the basioccipital. (**A**–**B**) Posterior view in *Tomistoma schlegelii* (NHMUK 1894.2.21.1) (**A**), and *Gavialis gangeticus* (NHMUK 1974.3009) (**B**); (**C**–**D**) ventral view in *Isisfordia duncani* (QM F44320) (**C**); *Alligator mississippiensis* (NHMUK 1873.2.21.1) (**D**); (**E**–**F**) posteroventral view in *Crocodylus porosus* (NHMUK 1852.12.9.2) (**E**), and *Gavialis gangeticus* (NHMUK 61.4.1.2) (**F**). All scale bars = 1 cm.

present (1) (after Jouve, 2004 [198]; Jouve et al., 2008 [187]).

The profile of the ventral margin of the basioccipital plate is straight or ventrally convex in most crocodylians (Fig. 54A–B). Jouve (2004) recognised that in some "gavialoids", the ventral margin has a marked concavity at the midline, e.g. *Piscogavialis jugaliperforatus* (Fig. 54C), *Gryposuchus neogaeus* (Fig. 54D), and *Ikanogavialis gameroi* (Sill, 1970). This condition is additionally recognised here in two caimanines: *Mourasuchus atopus* (UCMP 38012) and *Mourasuchus arendsi* (UFAC 4925).

134. Lateral eustachian foramina, position relative to medial eustachian foramen: dorsal (0); lateral (at the same level) (1) (after Norell, 1988 [46]; Brochu, 1997a [147]).

As explained by Brochu (2000), the three openings of the eustachian tube (one median and two lateral) are in-line in all hatchling crocodylians. By maturity, the lateral eustachian foramina mi-

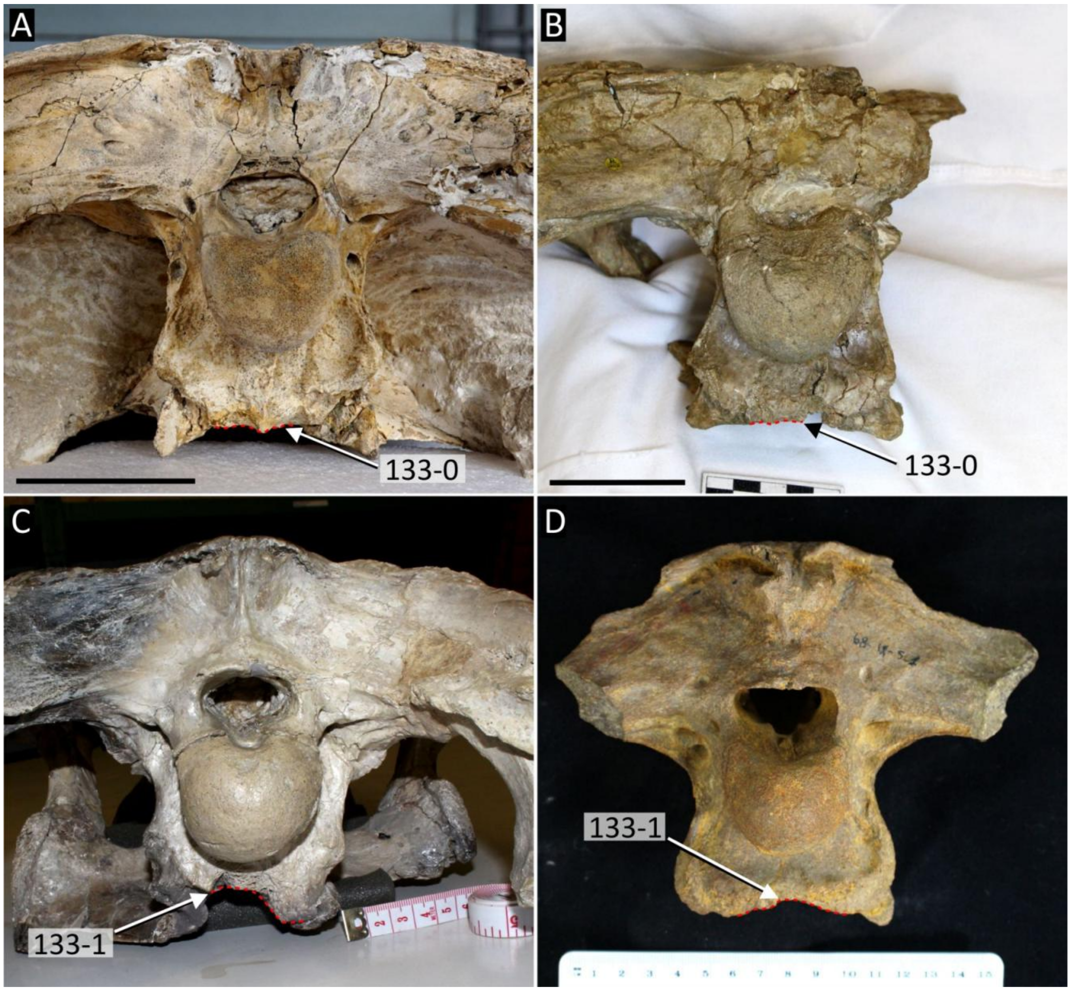

Figure 54: Occipital view of the cranium in **A**, *Thoracosuarus isorhynchus* (MNHN.F.MTA 61); **B**, *Eogavialis africanum* (NHMUK R 3108); **C**, *Piscogavialis jugaliperforatus* (SMNK 1282 PAL); **D**, *Gryposuchus neogaeus* (MLP 68-IX-V-1). Scale bars in A and B = 5 cm.

grate dorsal to the median eustachian foramen in many crocodylians. This condition occurs in *Borealosuchus* (e.g. *B. sternbergii*, Fig. 55A), *Asiatosuchus depressifrons* (Fig. 55A), *Gavialis gangeticus* (NHMUK 1974.3009), *Tomistoma schlegelii* (1894.2.21.1), and *Alligator mississippiensis* (NHMUK 1873.2.21.1). By contrast, in all extant *Crocodylus* species, as well as *Crocodylus thorbjarnarsoni* (Brochu & Storrs, 2012) and *Crocodylus anthropophagus* (Brochu et al., 2010), the lateral eustachian foramina remain in line with the median eustachian foramen.

135. Basisphenoid, dorsoventral exposure ventral to basioccipital, in posterior view (at maturity): little to no exposure (0); large exposure (1) (after Brochu, 1997a [119]).

As noted in Character 131, most crocodylians exhibit a “verticalised” cranium, characterised by a dorsoventrally tall skull, pterygoid wings that extend below the basioccipital, and several discrete morphological changes in the braincase (Tarsitano et al., 1989). Whereas the verticalised condition

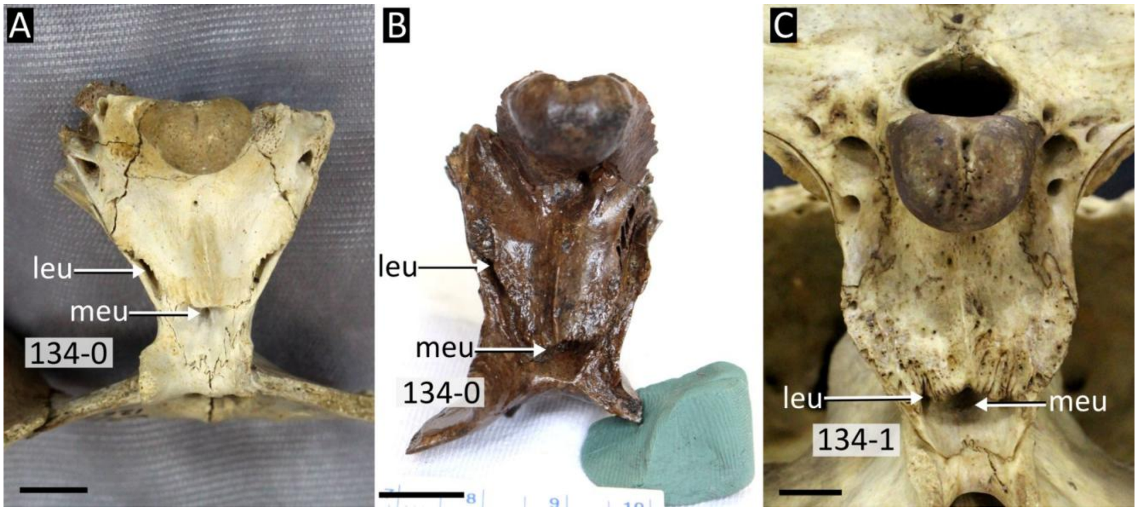

Figure 55: Posterior view of the occiput showing position of the lateral and medial eustachian foramina. **A**, *Borealosuchus sternbergii* (UCMP 126099); **B**, *Asiatosuchus depressifrons* (IRSNB R 0253); **C**, *Crocodylus palustris* (NHMUK 1897.12.31.1). Abbreviations: **leu**, lateral eustachian foramen, **meu**, median eustachian foramen. All scale bars = 1 cm.

is considered plesiomorphic for Crocodylia, the flattened cranium of *Gavialis gangeticus* is thought to be secondarily evolved (Brochu, 2006b). Increased dorsoventral exposure of the basisphenoid between the pterygoid and basioccipital is associated with this verticalisation (Fig. 56A). This condition occurs in *Hylaeochampsa vectiana* (NHMUK R177), *Allodaposuchus precedens* (Delfino et al., 2008a, fig.3A), *Borealosuchus sternbergii* (UCMP 126099), and most alligatoroids, e.g. *Melanosuchus niger* (Fig. 56A), *Paleosuchus palpebrosus* (AMNH 93812), and *Diplocynodon hantoniensis* (NHMUK 30392). By contrast, "gavialoids" such as *Gavialis gangeticus* (Fig. 56B), *Piscogavialis jugaliperforatus* (SMNK 1282 PAL), and *Eosuchus lerichei* (IRScNB R49), appear to lack any dorsoventral exposure of the basisphenoid. The basisphenoid is dorsoventrally exposed in many crocodyloids (e.g. *Crocodylus*) and "tomistomines", but this exposure is small, and considered more like the "gavialoid" condition, following (Brochu, 2006b).

136. Pterygoid, shape of posterior process ventrolateral to basioccipital: tall, long axis orientated dorsoventrally (0); dorsoventrally short, no discernible long axis (1) (after Brochu, 1997a [98]).

Posterior processes of the pterygoid are projections from the pterygoid wings that occur ventral to the basioccipital. Brochu (1997b) recognised that pterygoid processes varied in being tall and prominent in some taxa, but smaller in others. He also subdivided the 'small condition' based on their orientation, from being posteriorly directed (some derived "gavialoids", e.g. *Gavialis gangeticus*) or posteroventrally directed (most crocodyloids and "tomistomines"). This formulation precludes taxa sharing small pterygoid processes from being assigned the same character state. Furthermore, differences in orientation of the pterygoid processes were not discernible during the

examination of specimens. Consequently, this character has been simplified to a binary character describing only the size (dorsoventral elongation) of the pterygoid processes. Whereas most crocodylians exhibit dorsoventrally expanded processes, including all alligatoroids (Fig. 56A), most crocodyloids, "tomistomines", and "gavialoids" exhibit dorsoventrally short pterygoid processes (Fig. 56B).

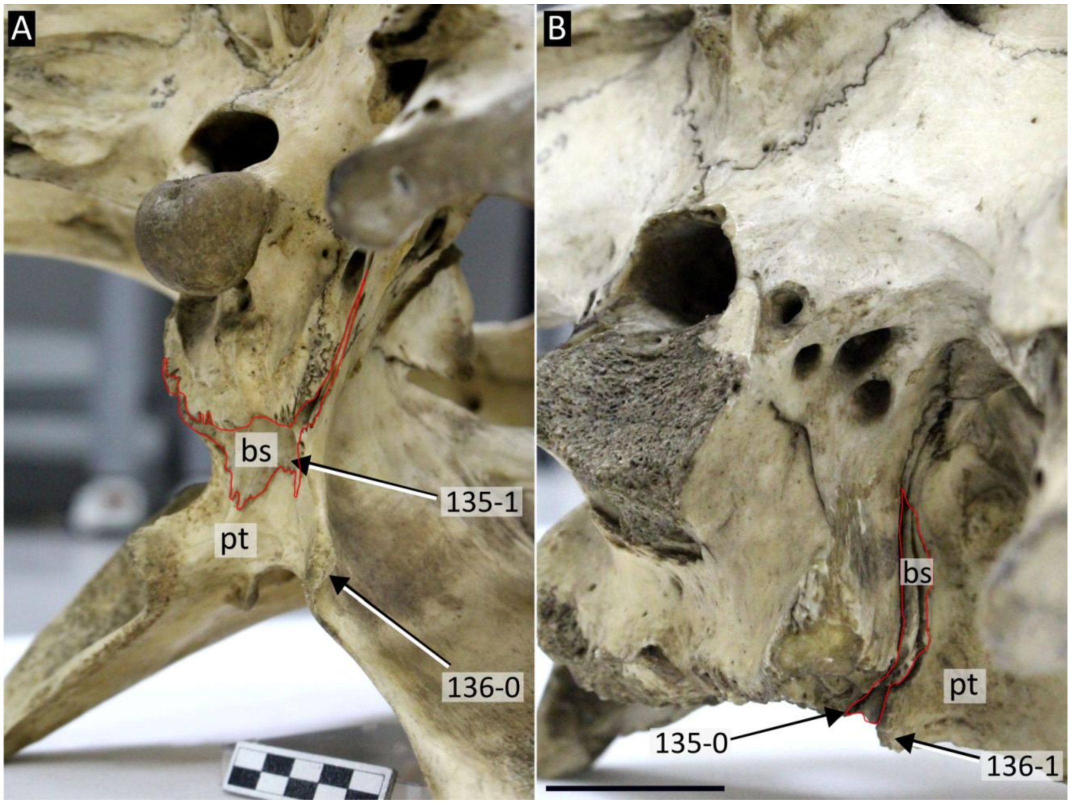

Figure 56: Occipital view of the cranium in **A**, *Melanosuchus niger* (NHMUK 45.8.25.125); **B**, *Gavialis gangeticus* (NHMUK 1974.3009). Abbreviations: **bs**, basisphenoid; **pt**, pterygoid. All scale bars = 5 cm.

Palate

Incisive foramen

137. Incisive foramen, intersection of premaxilla-maxilla suture: separated by inter-premaxillary suture (0); intersecting at posterior margin (1); intersecting at lateral margin (2) (after Brochu, 1997a [124]) (ORDERED).

In palatal view, the premaxilla-maxilla suture is separated from the incisive foramen by the inter-premaxillary suture in most crocodylians (Fig. 57A–B). Less commonly, the premaxilla-maxilla suture intersects the incisive foramen on its lateral margin e.g. *Brachychampsa montana* (Fig. 57E).

Brochu (1997b) characterised this morphological variation in terms of the size of the incisive foramen, which was considered either small (less than half the width across premaxillae), large (greater than half the width across the premaxillae), or so large that it intersects the incisive foramen (as in *Brachychampsa*). Here, the size of the incisive foramen is characterised in a continuous character (Character 12) and the intersection of the premaxilla-maxilla suture is treated independently. This is justified because taxa with proportionally similar-sized incisive foramina may or may not exhibit contact with the premaxilla-maxilla suture. Sutural contact with the incisive foramen (137-1) is newly recognised in *Alligator prenasalis* (Fig. 57C), *Navajosuchus mooki* (Fig. 57D), and *Allognathosuchus wartheni* (YPM PU 16989), all of which have intermediate-sized incisive foramina that are similar to many other crocodylians. In these taxa, the sutural intersection with the incisive foramen is considered intermediate between most crocodylians (Fig. 57A) and *Brachychampsa* (Fig. 57E), and thus the character is ordered.

138. Incisive foramen, anterior margin intersection with premaxillary tooth row: absent (anterior margin around 2nd or 3rd alveolus) (0); present (projects between or abuts first premaxillary teeth) (1) (after Brochu, 1997a [153]).

The distance between the anterior margin of the incisive foramen and the premaxillary toothrow varies in Crocodylia (Fig. 57E–F). As originally formulated by Brochu (1997a), this variation could be delimited with three character states: "*Incisive foramen … at the level of second or third alveolus (0); abuts premaxillary toothrow (1); projects between first premaxillary teeth (2)*" (Brochu, 1997a).

Brochu (1999) scored most caimanines where preserved, with his character state 2, e.g. *Caiman crocodilus* (Fig. 57B) and *Melanosuchus niger* (Fig. 57F). This is agreed on here; however, the distinction between this condition and character state 1 is not apparent (Brochu, 1999, fig.45A–B). As such, these character states have been combined here. Under the new binary character construction, most crocodylians exhibit a broad separation of the incisive foramen from the toothrow by the premaxilla (Fig. 57A, E), whereas several *Alligator* species and caimanines exhibit the derived condition (Fig. 57F). In recent datasets that use the character as formulated by Brochu (1997b), character state 2 is absent from scores altogether (e.g. Brochu, 2011; Brochu et al., 2012; Brochu & Storrs, 2012; Narváez et al., 2016). This appears to be an error carried over to subsequent iterations of this dataset, as other authors (e.g. Cidade et al., 2017) follow the scores of Brochu (1999).

139. Incisive foramen, posterior margin: rounded (0); invaginated by anterior process of premaxilla (spade-shaped foramen) (new character, based on personal observations).

In *Bernissartia fagesii* (IRScNB 1538) and many eusuchians, the incisive foramen is circular to oval. This condition is exhibited by *Allodaposuchus precedens* (MMS/VBN-12-10A), *Borealo-*

suchus sternbergii (USNM 6533), *Boverisuchus vorax* (FMNH PR 399), and many alligatoroids, e.g. *Alligator mississippiensis* (NHMUK 1873.2.21.1) and *Purussaurus neivensis* (UCMP 39704). A different condition is expressed in several caimanines, "tomistomines", and *Crocodylus* species, which have strongly spade-shaped incisive foramina due to an anterior projection of both pre-maxillae into the posterior margin, e.g. *Caiman crocodilus* (Fig. 57B), *Crocodylus johnstoni* (QM J45309), *Crocodylus moreletti* (NHMUK 1861.4.1.4), *Paleosuchus* (NHMUK 1868.10.8.1, AMNH 93812), and *Tomistoma schlegelii* (NHMUK 1894.2.21.1).

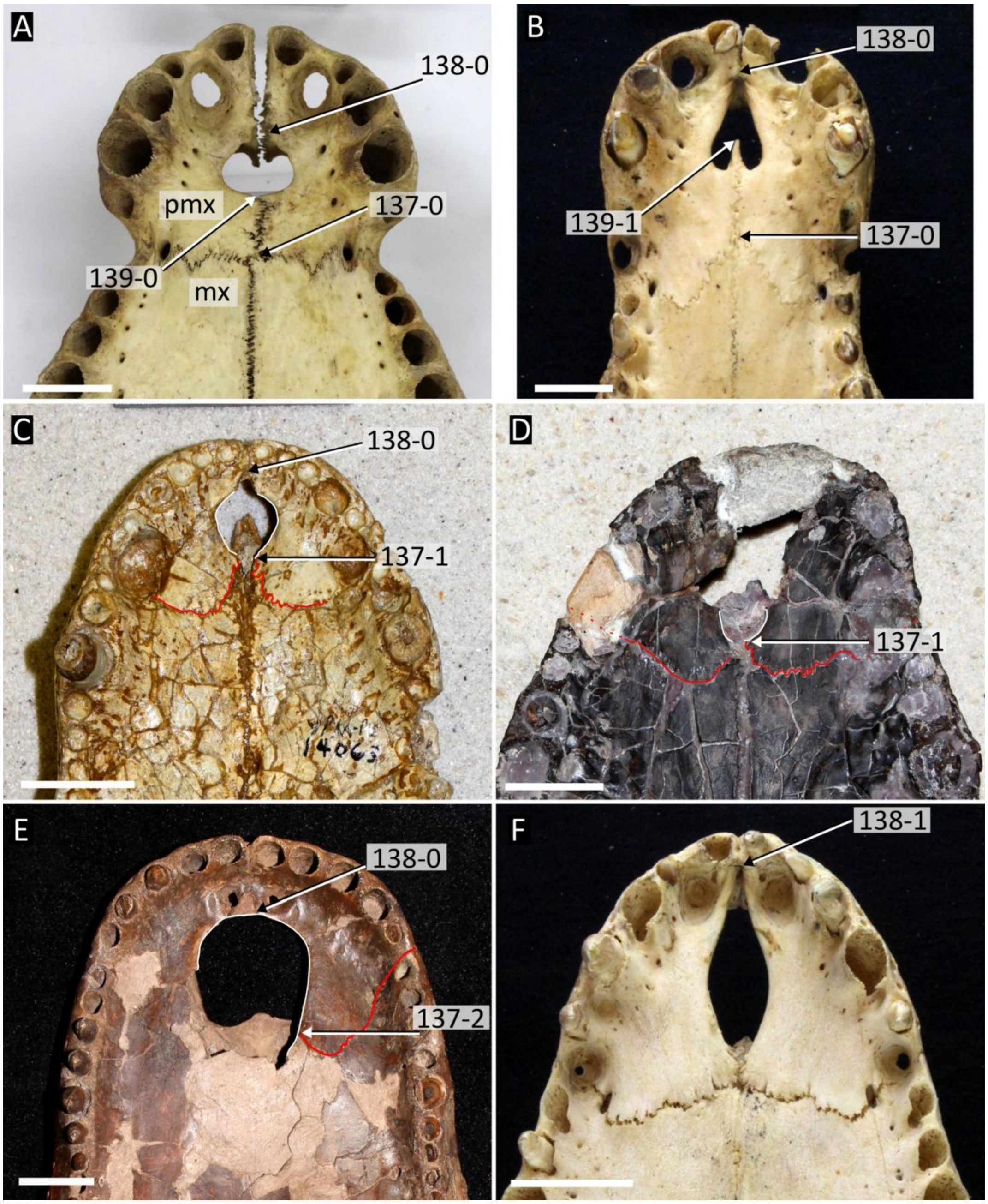

Figure 57: Ventral view of the anterior palate showing variation in morphology of the incisive foramen. **A,** *Crocodylus palustris* (NHMUK 1897.12.31.1); **B**, *Caiman crocodilus apaporiensis* (FMNH 69812); **C**, *Alligator prenasalis* (YPM PV 14063); **D**, *Navajosuchus mooki* (AMNH 5186); **E**, *Brachychampsa montana* (AMNH 5032); **F**, *Melanosuchus niger* (FMNH 45653). Red lines trace premaxilla-maxilla suutre, white lines mark boundary of incisive foramen. Abbreviations: **mx**, maxilla; **pmx**, premaxilla. All scale bars = 2 cm.

Premaxillary palate

140. Premaxilla, number of teeth early in post-hatching ontogeny: five (0); four (1) (after Norell, 1988 [17]; Brochu, 1997a [97]).

Where known, most eusuchians have five premaxillary alveoli upon hatching (Brochu, 1999) (Fig. 58B–C). By contrast, *Paleosuchus trigonatus* and *Paleosuchus palpebrosus* only have four (Fig. 58A) (Norell, 1988). Some longirostrine crocodylians, e.g. *Tomistoma schlegelii* (Fig. 58B), appear to only have four alveoli in adulthood (Iordansky, 1973); however, this is a result of reduction and loss of the 2nd premaxillary alveolus, which occurs secondarily. Evidence for secondary loss of the 2nd alveolus can occasionally be found as a scar for the missing alveolus immediately anterior to the third alveolus (Fig. 58C). In cases where four alveoli are preserved, with no evidence of a scar for the second alveolus, and no information is known of the post hatchling condition, taxa are scored as a "?". This is the case for *Piscogavialis jugaliperforatus* (SMNK 1282 PAL) and *Gryposuchus colombianus* (Fig. 58D).

141. Premaxilla, size of the three most posterior alveoli: penultimate alveolus is the largest (0); penultimate and antepenultimate alveoli are largest and similar in size (1); the antepenultimate alveolus is largest (2); alveoli are same size (3) (after Jouve et al., 2014 [225]; Salas-Gismondi et al., 2015 [201]; Salas-Gismondi et al., 2016 [201]).

Of the three posteriormost premaxillary alveoli, the penultimate is the largest in most eusuchians (141-0), including allodaposuchids (Narváez et al., 2016; Narváez et al., 2019), most crocodylids (e.g. *Crocodylus porosus*, Fig. 58B), alligatorids (e.g. *Alligator mcgrewi*, AMNH 7905; *Caiman yacare*, AMNH 97300), and some "tomistomines", e.g. *Maroccosuchus zennaroi* (IRScNB R408). The penultimate and antepenultimate alveoli are equally enlarged (141-1) in all *Borealosuchus* species, where preserved, *Diplocynodon hantoniensis* (NHMUK 25188), *Tomistoma schlegelii* (Fig. 58B), and *Gavialis gangeticus* (NHMUK 1974.3009). In a smaller number of taxa, the antepenultimate alveolus is the largest (141-2), e.g. *Gryposuchus neogaeus* (MLP 26-413) and *Gryposuchus pachakamue* (Salas-Gismondi et al., 2016), whereas in others, all premaxillary alveoli are equally enlarged (141-3), e.g. *Mourasuchus atopus* (Fig. 58E) and *Gavialosuchus eggenburgensis* (NHMUK R797).

142. Premaxilla, posterior extent on palate, relative to number of maxillary alveoli, in ventral view: 0 (0); 1 (1); 2 (2); 3 (3); 4 (4); 5 or more (5) (after Jouve, 2004 [168]; Jouve et al., 2008 [168]) (ORDERED).

Variation in the posterior extent of the premaxillae on the palate has been characterised by previous authors by means of a binary character that described the presence or absence of extension beyond

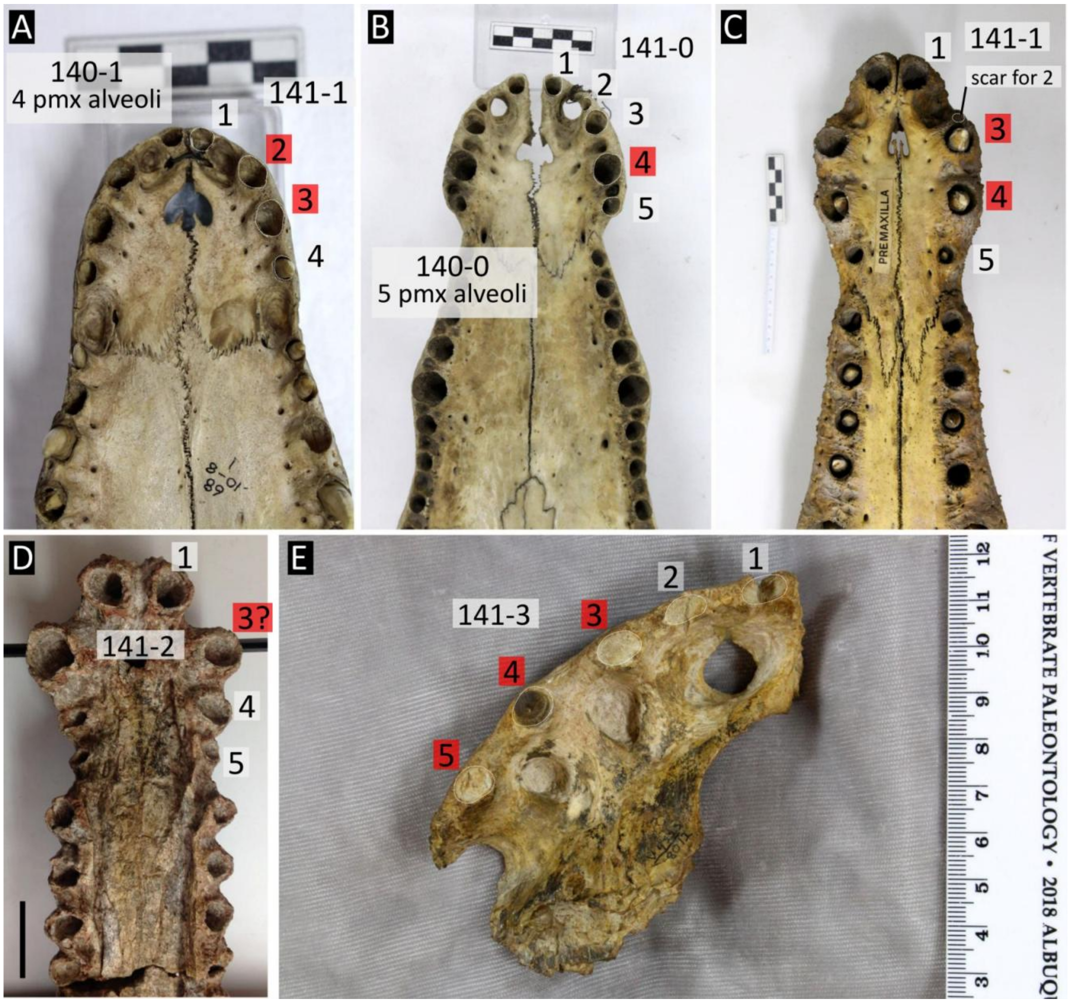

Figure 58: Ventral view of premaxilla in selected crocodylians showing variation in alveolar size and number. **A**, *Paleosuchus trigonatus* (NHMUK 1868.10.8.1); **B**, *Crocodylus porosus* (NHMUK 86.2.4.1); **C**, *Tomistoma schlegelii* (NHMUK 1894.2.21.1); **D**, *Gryposuchus colombianus* (IGM 201400011); **E**, *Mourasuchus atopus* (UCMP 38012). Red boxes indicate position of largest alveoli (character 141) Scale bar in D = 5 cm.

the 3rd maxillary alveolus (Jouve, 2004). Finer variation is recognised here with the addition of numerous, ordered character states. In *Toyotamaphimeia machikanensis* (Kobayashi et al., 2006), *Maomingosuchus petrolica* (Shan et al., 2017), and *Diplocynodon* species (e.g. *D. hantoniensis*, NHMUK 25166) and *D. ratelii*, MNHN SG 539), the premaxillae do not reach the level of even one maxillary alveolus. In most species of *Crocodylus*, *Alligator*, and *Borealosuchus*, they reach one alveolus (Fig. 59A). The premaxillae reach two full maxillary alveoli in *Eosuchus lerichei* (IRScNB R49), *Tomistoma schlegelii* (Fig. 59B), and *Gryposuchus colombianus* (Fig. 59C). The premaxillae extend to the level of the 3rd, 4th, and 5th maxillary alveoli in *Eogavialis africanum* (Fig. 59D), *Gavialis gangeticus* (Fig. 59E), and *Piscogavialis jugaliperforatus* (Fig. 59F), respectively.

143. Premaxilla, position of the penultimate premaxillary alveolus relative to the antepenultimate alveolus: posterolateral or in the same line (0); posteromedial (1) (after Jouve et al., 2015 [202])

144. Premaxilla, position of the last premaxillary alveolus relative to the penultimate alveolus: posterior or posterolateral (0); or posteromedial (1) (after Jouve et al., 2015 [204])

Characters 143–144 capture variation in the premaxillary alveolar arrangement and are modified from characters 202 and 204 in Jouve et al. (2015). Overlapping statements occurred in the original character descriptions that would result in overweighting. Furthermore, the original characters did not account for the full range of morphological variation observed in crocodylians. In most eusuchians the premaxillary toothrow is arranged in an arched, posterolateral line (Fig. 59A). In this condition the antepenultimate, penultimate and last premaxillary alveoli are positioned progressively further laterally (143-0, 144-0). Several longirostrines exhibit the opposite condition, in which the final three alveoli are arranged in a posteromedial line (143-1, 144-1) (Fig. 59C). This gives rise to the characteristic widened premaxilla ('Greifapparat') of several "gavialoids" (Kälin, 1933). In other longirostrines, the penultimate alveolus is lateral to, or at the same level as the antepenultimate alveolus (143-0), but the final alveolus is positioned medial to them (144-1) (Fig. 59B, F). This morphological variation could alternatively be described in one character describing the three aforementioned conditions; however, this would ignore the shared presence of a medially inset last premaxillary alveolus in *Gavialis gangeticus* (Fig. 59E), *Gryposuchus colombianus* (Fig. 59C), *Tomistoma schlegelii* (Fig. 59B), *Thecachampsa sericodon* (USNM 24938), and *Maroccosuchus zennaroi* (IRScNB R408), among other longirostrines.

145. Premaxilla, alveolar spacing (at maturity): all alveoli equally separated (0); second alveolus separated from the first and close to the third (1) (after Jouve et al., 2015 [224]).

As explained in Character 140, the second premaxillary alveolus may be completely lost in adult forms of some species, but more often it remains small and weakly separated from the third (Fig. 59E) (Brochu, 1999). This condition occurs in most *Crocodylus* species (e.g. *C. intermedius*, FMNH 75659), all *Caiman* species, where preserved (e.g. *Caiman crocodilus*, Fig. 58B), *Borealosuchus sternbergii* (USNM 6533), some *Diplocynodon* species (e.g. *D. ratelii*, MNHN SG 539), and *Baru huberi* (QM F31060). An almost equal number of taxa exhibit equidistant premaxillary alveoli. This condition occurs in all *Alligator* species (Fig. 59A), *Mourasuchus atopus* (Fig. 58E), *Thoracosaurus isorhynchus* (MNHN 1902-22), *Eosuchus lerichei* (IRScNB R49), and *Boverisuchus vorax* (FMNH PR 399).

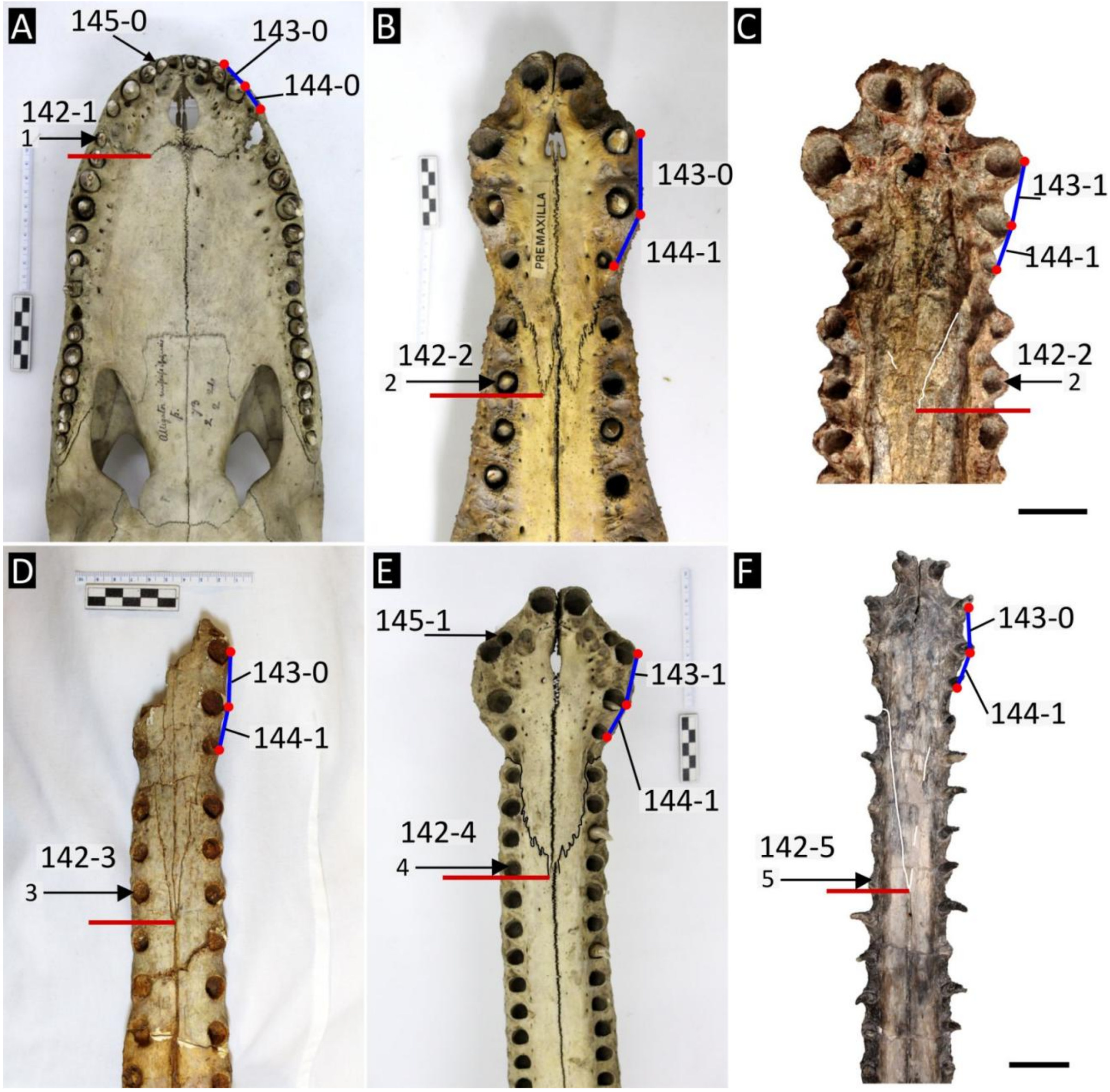

Figure 59: Ventral view of the rostrum in selected crocodylians showing posterior extent of the premaxilla and variation in alveolar arrangement. **A**, *Alligator mississippiensis* (NHMUK 1873.2.21.1); **B**, *Tomistoma schlegelii* (NHMUK 1894.2.21.1); **C**, *Gryposuchus colombianus* (IGM 201400011); **D**, *Eogavialis africanum* (NHMUK PV R3329); **E**, *Gavialis gangeticus* (NHMUK 1974.3009); **F**, *Piscogavialis jugaliperforatus* (SMNK 1282 PAL). Red lines indicate posterior extent of premaxillae, black lines indicate alveolus position. Red points mark premaxillary alveolar positions All scale bars = 5 cm.

146. Premaxilla-maxilla suture, shape on palate in ventral view: horizontal (0); posteriorly bowed, with one rounded apex (1); posteriorly bowed, with one acute apex (2); posteriorly bowed with two or more apicies (3) (after Groh et al., 2020 [122]).

The ventral premaxilla-maxilla suture undulates in most crocodylians, most commonly with two posterior projections (146-3). This condition occurs in most extant alligatorids (e.g. *Caiman crocodilus*, FMNH 69812), crocodylids (e.g. *Crocodylus niloticus*, Fig. 60D), "tomistomines" (e.g. *Tomistoma schlegelii*, NHMUK 1894.2.21.1), and some "gavialoids" (e.g. *Eosuchus lerichei*, IRScNB R49). Alternatively, the suture may be straight (146-0), as in *Bernissartia fagesii* (IRScNB 1538), *Voay robustus* (Fig. 60A), *Asiatosuchus germanicus* (HLMD Me 5649), *Baru* (QM F16822, F31060), and *Diplocynodon hantoniensis* (NHMUK 25166). Several taxa exhibit one median posterior projection of the premaxilla-maxilla suture, which might be acute (146-2) (Fig. 60C), or as newly introduced here, rounded (146-1) (Fig. 60B). Whereas the acute condition commonly occurs in longirostrine crocodylians, e.g. *Crocodylus johnstoni* (QM J4280), *Gavialis gangeticus* (NHMUK 1974.3009), and *Kentisuchus spenceri* (NHMUK 38974), the rounded condition is less common, occurring in some crocodyloids, e.g. *C. moreletti* (Fig. 60B), '*C*'. *affinis* (AMNH 16622, UCMP 131090), and '*C*'. megarhinus (YPM 53582).

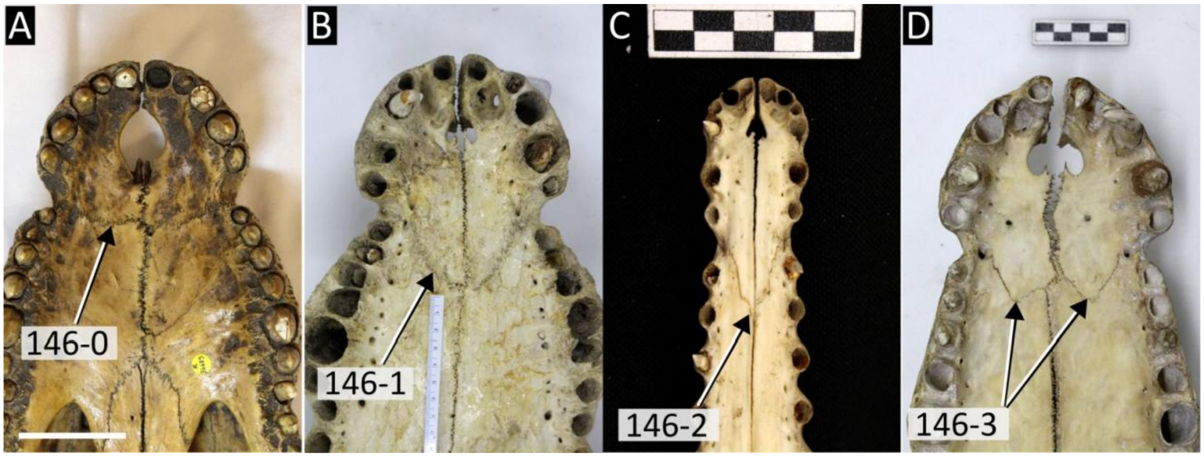

Figure 60: Ventral view of the premaxilla-maxilla suture. **A**, *Voay robustus* (NHMUK R 36685); **B**, *Crocodylus moreletti* (NHMUK 1861.4.1.4); **C**, *Crocodylus johnstoni* (QM J4280); **D**, *Crocodylus niloticus* (NHMUK 1934.6.3.1). Scale bar in A = 5 cm, all other scale bars = cm.

Maxillary alveoli

147. Maxilla, number of the largest alveolus: 3 (0); 5 (1); 4 (2); 4 and 5 (3); 6 (4); 7 (5); maxillary alveoli gradually increase in diameter posteriorly toward penultimate alveolus (6); homodont (7) (after Norell, 1988 [1]; Brochu, 1997a [89]; Shan et al., 2009 [89]; Brochu, 2010 [73]; Jouve et al., 2015 [89]).

The number of states in this character has steadily grown with the discovery of new size arrangements of maxillary alveoli. The largest maxillary alveolus is the third in paralligatorids (Turner, 2015). This includes the 'Glen Rose Form' (Fig. 61A), *Wannchampsus kirkpachi* (Adams, 2014), and *Shamosuchus djadochtaensis* (Pol et al., 2009). The 5th maxillary alveolus is enlarged in all crocodyloids (e.g. *Crocodylus porosus*, Fig. 61E) and *Asiatosuchus germanicus*, HLMD Me 5652) and most "tomistomines" (e.g. *Tomistoma schlegelii*, Fig. 61F and *Thecachampsa sericodon*, Fig. 61G). In memebrs of Allodaposuchidae (e.g. *Allodaposuchus precedens*, MMS/VBN-12-10A), as well as most alligatoroids (e.g. *Caiman yacare*, Fig. 61B) and *Navajosuchus mooki*, AMNH 5186) the 4th maxillary alveolus is largest. The 4th and 5th maxillary alveoli are equally enlarged in all *Borealosuchus* species (Brochu, 1997a) (Fig. 61D), planocraniids (e.g. *Boverisuchus vorax*, FMNH PR 399), and 'basal' alligatoroids, e.g. *Diplocynodon hantoniensis* (Fig. 61C) and *Leidyosuchus canadensis* (NHMUK R10904). Enlargement of the 6th maxillary alveolus has only been observed in *Gavialosuchus eggenburgensis* (Fig. 61H). Similarly, enlargement of the 7th alveolus is rare, only observed in *Penghusuchus pani* (Shan et al., 2009, fig.2B) and *Toyotamaphimeia machikanensis* (Kobayashi et al., 2006, fig.8). A progressive enlargement of the posteriormost maxillary alveoli is only recovered in *Hylaeochampsa vectiana* (NHMUK R177), *Iharkutosuchus makadii* (Ösi, 2008, fig.9) and *Acynodon iberoccitanus* (Martin, 2007, fig.3D). Equally sized (homodont) maxillary alveoli are recovered in several longirostrine crocodylians, including *Gavialis gangeticus* (Fig. 61K), *Eosuchus lerichei* (Fig. 61J), and '*Tomistoma*' *dowsoni* (Fig. 61I). Given the great degree of variation that does not form a clear, continuous range of values, this character is unordered.

148. Maxilla, interalveolar distances between alveoli 1–10: less than or equal to diameter of first maxillary alveolus (0); greater than the diameter of the first maxillary alveolus (1) (after Jouve et al. 2015 [235]).

In most brevirostrine crocodylians, the maxillary teeth sit close together such that most of the interalveolar spaces between maxillary alveoli 1–10 are small, being equal to or less than the diameter of the first maxillary alveolus (Fig. 61A–E). Although variation occurs depending on how the dentary teeth occlude (see Character 151), the interalveolar distances are never consistent, nor widely spaced, across the maxillary toothrow. Several longirostrine crocodylians exhibit very evenly spaced maxillary alveoli; however, the interalveolar distances are still small, typically less than the diameter of the first maxillary alveolus, e.g. *Eosuchus lerichei* (Fig. 61J) and *Gavialis gangeticus* (Fig. 61K). By contrast, a small number of longirostrine crocodylians exhibit maxillary alveoli that are both consistently distributed and large, approximately 1.5 times the diameter of the first maxillary alveolus. This occurs in *Gavialosuchus eggenburgensis* (Fig. 61H), '*Tomistoma*' *dowsoni* (Fig. 61I), *Tomistoma cairense* (SMNS 50739), *Eogavialis africanum* (YPM 6263), and

Piscogavialis jugaliperforatus (SMNK 1282 PAL), among other longirostrine crocodylians.

149. Maxilla, shape of the lateral profile between alveoli 1 to 5: flaring posteriorly (0); or straight (1) (new character, based on personal observations).

The rostra of all brevirostrine eusuchians widen posteriorly from the level of the first maxillary alveolus to either the 3rd, 4th, or 5th, depending on which is the largest (149-0) (Fig. 61A–E). This is sometimes described as the first 'wave' of the maxilla (i.e. a convexity in the lateral profile), with a second wave occurring posteriorly. Almost all longirostrine crocodylians with homodont dentition lack such a wave, and the lateral profile of the maxilla is straight between alveoli 1–5 (149-1), being parallel to the sagittal axis (Fig. 61I–K). This might suggest that this morphological variation is associated with the presence or absence of homodont dentition, which is described in Character 147-7. Indeed, longirostrines with heterodont dentition tend to exhibit the same widening as all brevirostrines e.g. *Tomistoma schlegelii* (Fig. 61F), *Maroccosuchus zennaroi* (IRScNB R408), and *Kentisuchus spenceri* (NHMUK 38974); however, some longirostrines with heterodont dentition have a straight profile between alveoli 1–4, e.g. *Thecachampsa sericodon* (Fig. 61G), *Gavialosuchus eggenburgensis* (Fig. 61H), and *Maomingosuchus petrolica* (IVPP unnumbered complete skull). This indicates that the shape of the lateral maxillary margin is not always associated with differentiation of the maxillary alveoli, supporting the independence of this character.

150. Maxilla, shape of the toothrow posterior to the first six alveoli: laterally convex or linear (0); laterally concave (1) (after Brochu, 1997a [135]; Clark, 1994 [79]).

In most eusuchians, the posteriormost maxillary alveoli are arranged in a straight line which, although orientated posterolaterally, recurves medially at its distal end (Fig. 61A–E). Brochu (1997a) recognised that the posterior end of the toothrow does not medially recurve in *Borealosuchus* species (Fig. 61D), a condition which has since been used to diagnose the genus (Brochu et al., 2012). However, this condition is recognised much more widely in crocodylians in this study, particularly in longirostrines e.g. *Tomistoma schlegelii* (Fig. 61F), *Thecachampsa sericodon* (Fig. 61G), *Eosuchus lerichei* (Fig. 61J), and *Gavialis gangeticus* (Fig. 61K). Furthermore, the posterior toothrow is considered linear in *Borealosuchus sternbergii* (Fig. 61D).

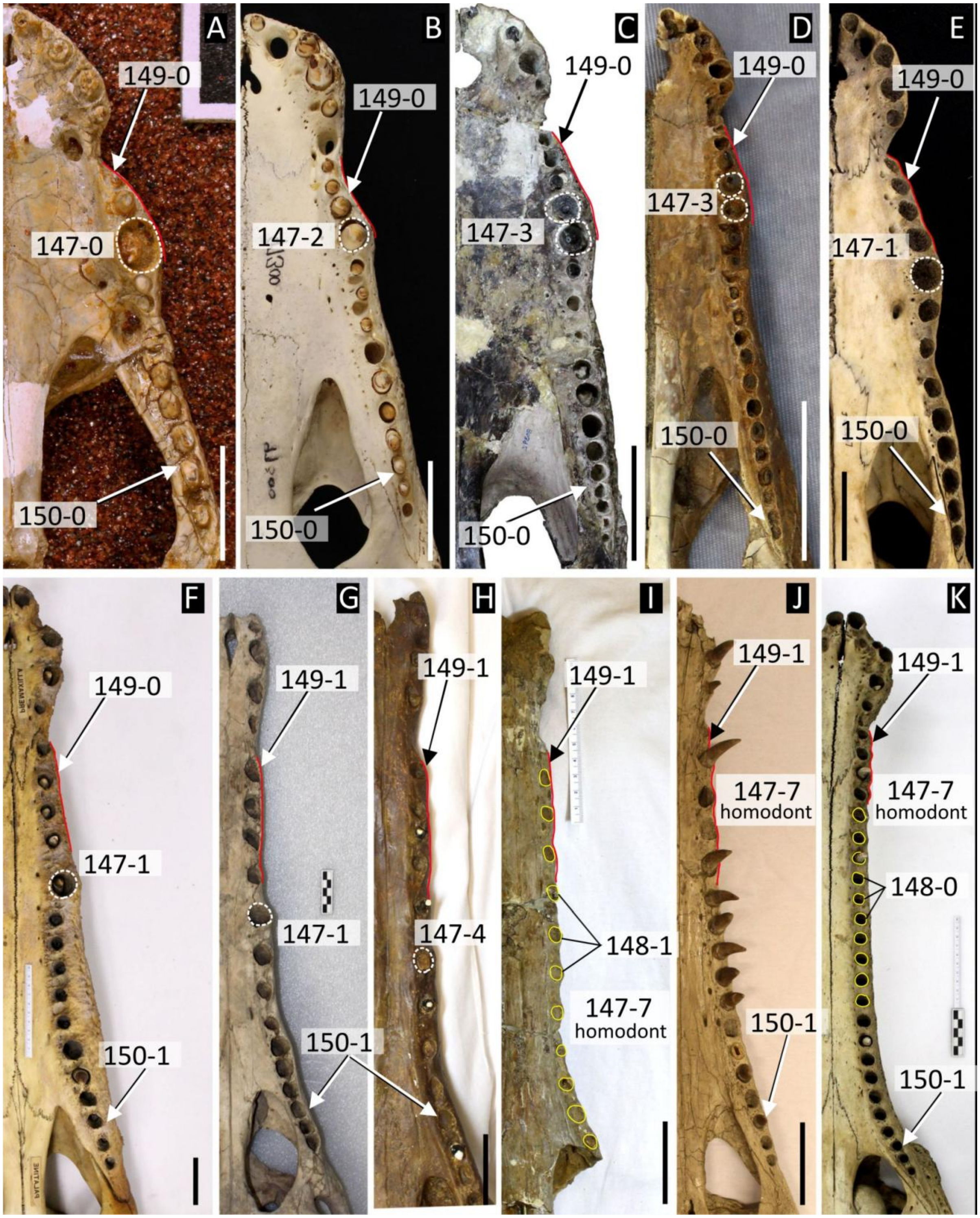

Figure 61: Ventral view of the palate in selected crocodylians showing variation in maxillary alveolar morphology. **A**, the Glen Rose Form (USNM 22039); **B**, *Caiman yacare* (AMNH 97300, digitally reversed); **C**, *Diplocynodon hantoniensis* (NHMUK 30392, digitally reversed); **D**, *Borealosuchus sternbergii* (UCMP 126099); **E**, *Crocodylus porosus* (QM J47447); **F**, *Tomistoma schlegelii* (NHMUK); **G**, *Thecachampsa sericodon* (USNM); **H**, *Gavialosuchus eggenburgensis* (NHMUK PV R 797); **I**, *Tomistoma dowsoni* (NHMUK PV R 4769); **J**, *Eosuchus lerichei* (IRSNB R 49); **K**, *Gavialis gangeticus* (NHMUK 1974.3009). Scale bar A = 1 cm, all other scale bars = 5 cm.

151. Occlusion pattern: all dentary teeth occlude lingual to maxillary teeth (0); partial interlocking occlusion, with at least one pit between maxillary teeth 5–8, all other dentary teeth occlude lingually (1); all dentary teeth occlude in line with maxillary teeth (2) (after Norell, 1988 [5]; Willis, 1993 [1]; Brochu, 1997a [78]; Lee and Yates 2018 [27] (ORDERED).

In *Bernissartia fagesii* (IRScNB 1538), Hylaeochampsidae, Paralligatoridae and most alligatoroids, the dentary teeth occlude lingual to the maxillary teeth (Fig. 62A–B). This also occurs in some 'basal' crocodyloids, such as *Asiatosuchus germanicus* (HLMD Me 5652). By contrast, the dentary teeth occlude in line with the maxillary teeth in most crocodyloids (e.g. *Crocodylus*, Fig. 62D), all gavialoids (e.g. *Tomistoma schlegelii*, NHMUK 1894.2.21.1 and *Gavialis gangeticus*, NHMUK 1974.3009), and most *Borealosuchus* species (e.g. *B. acutidentatus*, NMC 8544). In a less common condition that was recognised by Brochu (1997b), occlusal pits can occur between maxillary teeth 5–8, but lingual to all other maxillary teeth (Fig. 62C). Previously, only a single pit was recognised between alveoli 7 and 8 (Brochu, 1997b) or 7–9 (Lee & Yates, 2018), but greater variation exists. Whereas occlusal pits are present between alveoli 6 and 7, and 7 and 8 in '*Crocodylus*' *affinis* (USNM 18171) and *Diplocynodon hantoniensis* (NHMUK 25166), an occlusal pit only occurs between alveoli 7 and 8 in *Asiatosuchus depressifrons* (Fig. 62C). In *Caiman crocodilus apaporiensis* (FMNH 69812) an occlusal pit occurs between alveoli 5–8, and in Caiman yacare (AMNH 97300) they occur between alveoli 5–7. These conditions are considered intermediate between a full lingual occlusion (151-0) and fully interlocking dentition (151-2), hence the character is ordered.

152. Occlusion pattern, 4th dentary tooth occludes in notch between premaxilla and maxilla early in ontogeny (0); occludes in a pit between premaxilla and maxilla; no notch early in ontogeny (1) (after Norell, 1988 [29]; Brochu, 1997a [77]).

The occlusal position of the 4th dentary caniniform tooth has classically been used to distinguish alligatorids from all other crocodylians (Brochu, 1999, 2003; Duméril, 1806; Norell et al., 1994). Whereas alligatoroids exhibit a pit that encloses the 4th dentary tooth (Fig. 62E), crocodyloids and gavialoids exhibit a notch, such that the 4th dentary tooth is laterally exposed (Fig. 62F). The character must be qualified with a statement about ontogeny, as it has long been recognised that a pit can become worn to a notch with maturity (Brochu, 1999; Kälin, 1933; Norell et al., 1994). This is exemplified by *Caiman crocodilus*, hatchling specimens of which exhibit a pit (Fig. 62G), but the lateral wall of the pit can become worn out and entirely lost with maturity (Fig. 62H). Similar intraspecific variation is observed in fossil specimens e.g. *Diplocynodon hantoniensis* (Brochu, 1999; Norell et al., 1994, see Chapter 2 also).

153. Maxilla, diastema between alveoli 5 and 6: absent (0); present (1) (new character, based on personal

observation).

A prominent diastema occurs between alveoli 5 and 6 in the 'Glen Rose Form' (USNM 22039, Fig. 62A), *Wannchampssus kirkpachi* (Adams, 2014, fig.8), and *Thoracosaurus isorhynchus* (MNHN 1902-22). All other eusuchians exhibit sub-equally separated maxillary alveoli.

154. Maxilla, diastema between alveoli 6–8: absent (0); present (1) (after Montefeltro et al. 2013 [484]; Jouve et al. 2015 [235]).

The derived character state describes the presence of a diastema between alveoli 6–8. This might appear redundant with character 151, as taxa with occlusal pits between alveoli 5–8 (151-1) naturally exhibit increased interalveolar spacing similar to that described in 154-1. However, several crocodylians exhibit increased interalveolar spacing between alveoli 6–8 regardless of occlusal pattern. For example, most *Crocodylus* species (which have fully interlocking dentition, 151-2) exhibit increased interalveolar spaces (154-1). By contrast, *Gavialis gangeticus*, which also has interlocking dentary and maxillary teeth, lacks a diastema between alveoli 6–8 (154-0). Furthermore, despite the full lingual occlusion of all *Alligator* species (151-0), some exhibit slightly larger interalveolar spaces between alveoli 6–8, e.g. *A. mississippiensis* (Fig. 62B).

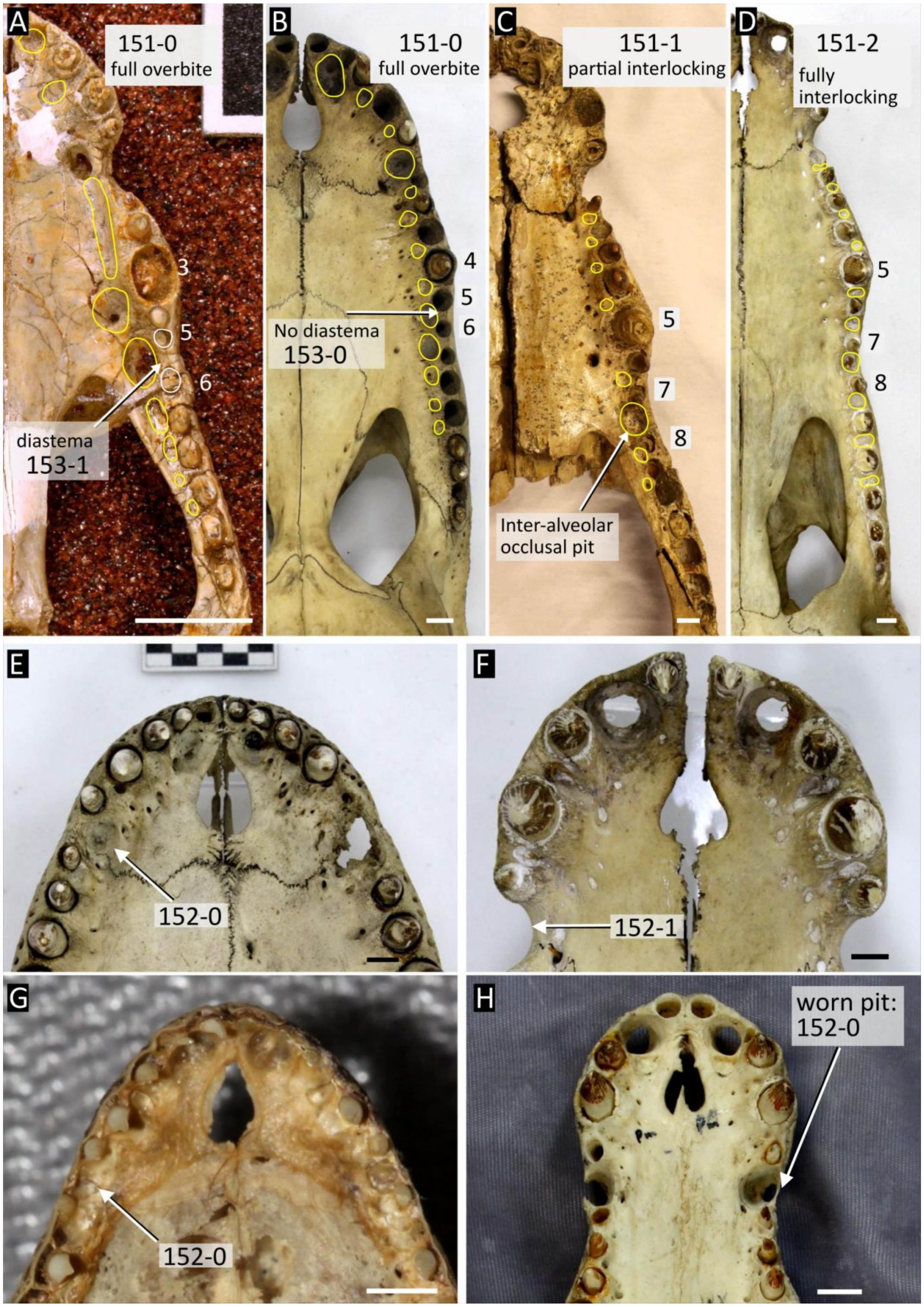

Figure 62: Ventral view of the palate showing variation in occlusal pattern in **A**, the Glen Rose Form (USNM 22039); **B**, *Alligator mississippiensis* (NHMUK 1873.2.21.1); **C**, *Asiatosuchus depressifrons* (IRSNB R 0251); **D**, *Crocodylus siamensis* (NHMUK 1921.4.1.171); **E**, *Alligator mississippiensis* (NHMUK 1873.2.21.1); **F**, *Crocodylus siamensis* (NHMUK 1921.4.1.171); **G**, *Caiman crocodilus apaporiensis* (hatchling) (UCMP unnumbered); **H**,*Caiman crocodilus apaporiensis* (adult) (UCMP 42843). All scale bars = 1 cm, except G = 2 mm.

155. Maxillary and dentary alveoli, shape: all circular in cross-section (0); posterior alveoli mediolaterally compressed (1); all alveoli mediolaterally compressed (2) (after Brochu, 2004 [165]; Brochu, 2010 [61]).

The maxillary and dentary alveoli of *Bernissartia fagesii* (IRScNB 1538) and most eusuchians are circular throughout the toothrow (Fig. 63A). By contrast, the posteriormost alveoli are mediolaterally compressed in *Isisfordia duncani* (QM F44320) and some alligatoroids, e.g. *Paleosuchus* (Fig. 63B), *Arambourgia gaudryi* (MNHN QU17155), *Procaimanoidea utahensis* (USNM 15996), and *Bottosaurus harlani* (Cossette & Brochu, 2018). In planocraniids such as *Boverisuchus vorax* (Fig. 63D), and the crocodyloid *Quinkana* (Fig. 63C), the alveoli are mediolaterally compressed throughout the toothrow (Brochu, 2004b, 2012).

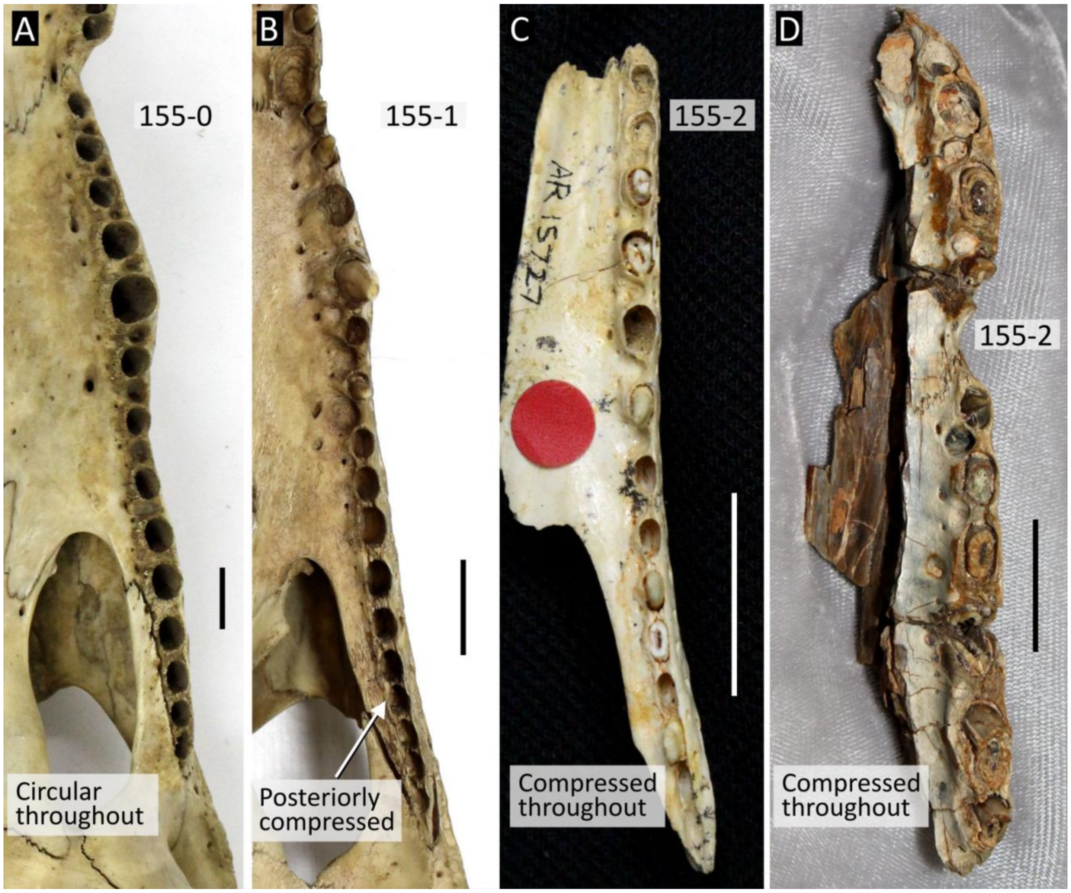

Figure 63: Ventral view of the maxillary toothrow showing variation in alveolar compression. **A**, *Crocodylus porosus* (NHMUK 85.2.4.1); **B**, *Paleosuchus trigonatus* (NHMUK 1868.10.8.1); **C**, *Quinkana meboldi* (QM F31056); **D**, *Boverisuchus vorax* (UCMP 170767). All scale bars = 2 cm.

156. Dentary and maxillary teeth, shape behind alveoli 12–13: pointed to slightly blunt (0); globular (1); molariform, multicusped (2) (after Salas-Gismondi et al. 2015 [198]).

Moving posteriorly through the toothrow, the maxillary and dentary teeth of most alligatoroids

become short and stout, sometimes with blunter apices than the anteriormost teeth (Cidade et al., 2019a). Some taxa exhibit a prominent increase in size become bulbous and blunt (156-1) (Brochu, 1999, 2004b). This condition occurs in several alligatoroids including *Brachychampsa montana* (Fig. 64C), *Hassiacosuchus haupti* (Fig. 64D), *Navajosuchus mooki* (AMNH 6780), *Allognathosuchus wartheni* (YPM PU 16989), and the caimanines *Globidentosuchus brachyrostris* (Scheyer & Delfino, 2016), *Gnatusuchus pebasensis*, and *Caiman wannlangstoni* (Salas-Gismondi et al., 2015). In fewer cases, the posteriormost teeth are not only enlarged and flattened, but develop multiple furrows and ridges (cusps) to give a molariform appearance e.g. *Iharkutosuchus makadii* (Ösi, 2008, fig.7).

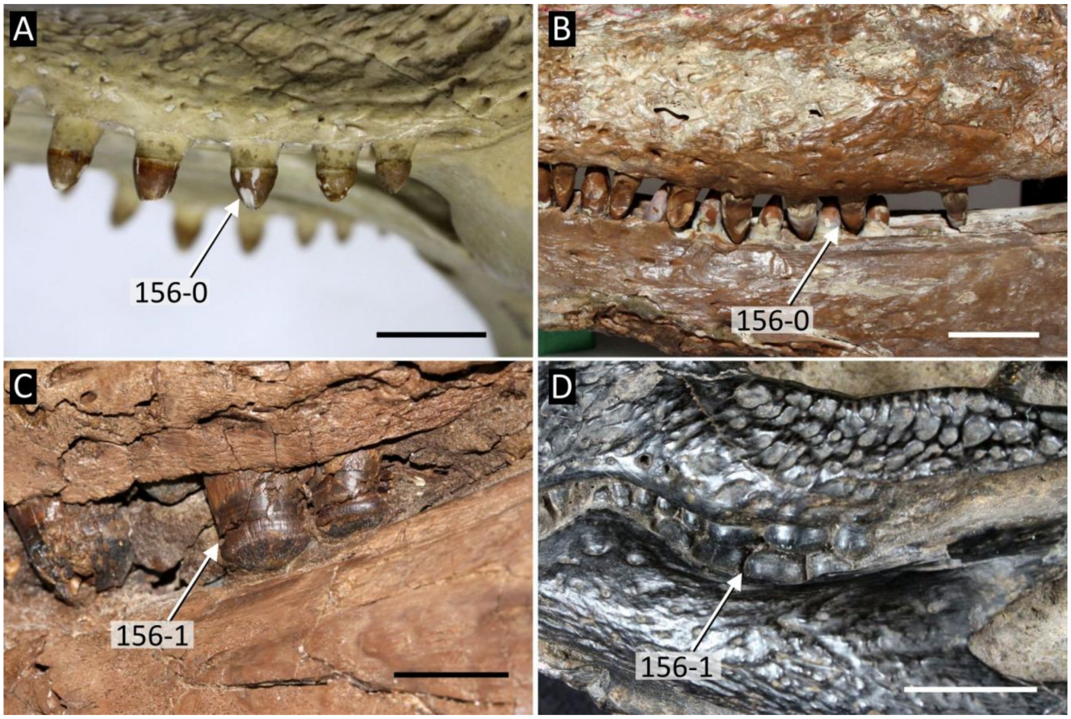

Figure 64: Lateral view of the posteriormost maxillary and dentary teeth in selected crocodylians. **A**, *Crocodylus siamensis* (NHMUK 1921.4.1.171); **B**, *Diplocynodon ratelii* (MNHN SG 539); **C**, *Brachychampsa montana* (UCMP 133901); **D**, *Hassiacosuchus haupti* (HLMD-Me-4415). All scale bars = 2 cm.

157. Maxillary and dentary tooth carinae: smooth (0); serrated (1) (after Brochu 2010 [62]).

Whereas the sharp anterior (mesial) and posterior (distal) edges (carinae) of the teeth of most crocodylians are smooth (Fig. 65A), those of *Boverisuchus vorax* (Fig. 65C, D), *Boverisuchus magnifrons* (Brochu, 2012), and species of *Quinkana* (e.g. *Q. fortirostrum*, QM 32153) bear saw-like serrations. Teeth associated with the giant caimanine crocodylian, *Purussaurus neivensis* (UCMP 38932, Fig. 65A, B), have structures that superficially resemble serrations, but lack a saw-like edge. Lee and Yates (2018) modified this character by the addition of a character state: "weakly crenulated (i.e. serrated) with microscopic crenulations". Of the five taxa scored for this condition

in their study (*Planocrania datangensis*, *Baru darrowi*, *Baru* ‘Alcoota’, *Quinkana timara*, and *Volia athollandersoni*), only *P. datangensis* was examined first hand here, and the condition was not observed; as such, this character state is excluded.

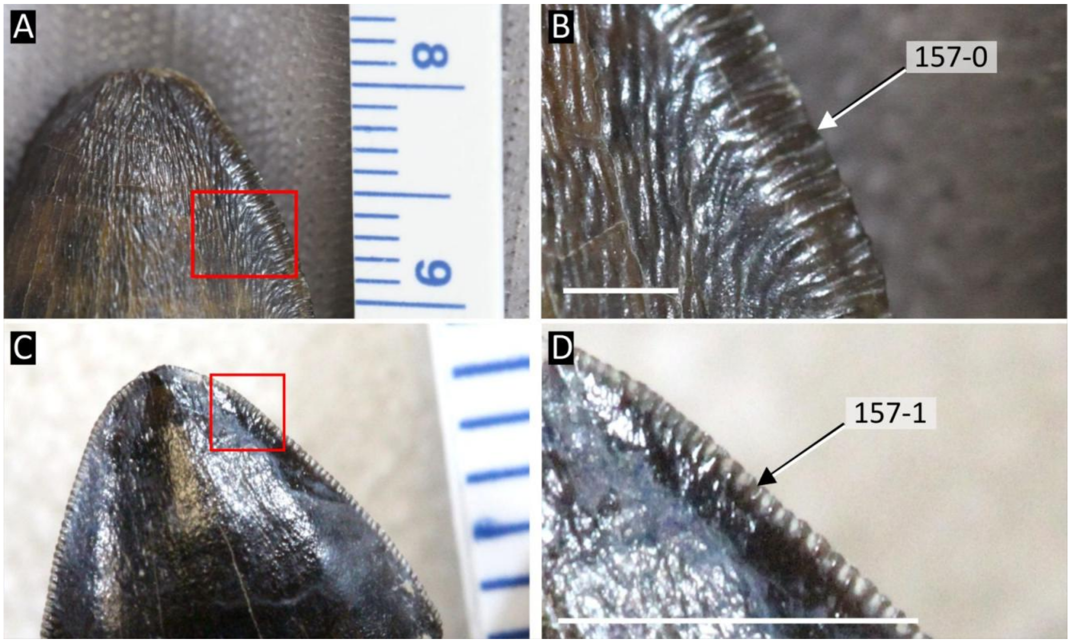

Figure 65: Tooth morphology showing variation in development of serrations. **A**, *Purussaurus neivensis* (UCMP 38932); **B**, enlargement of highlighted area in A; **C**, *Boverisuchus vorax* (UCMP 170767). **D**, enlargement of highlighted area in C. Scale bars in B and D = 1 mm.

158. Maxilla, position of alveoli relative to maxillary palate separating toothrows: ventral or at the same level (0); dorsal (1) (after Hua and Jouve, 2004 [165]; in Jouve 2016 [165]).

In *Bernissartia fagesii* (IRScNB 1538) and most eusuchians, the maxillary alveolar walls are positioned ventral to the remainder of the palatal surface. This occurs is all longirostrines recovered as “tomistomines”, e.g. *Tomistoma schlegelii* (Fig. 66A), *Maroccosuchus zennaroi* (IRScNB R408), and *Thecachampsa sericodon* (USNM 24938). By contrast, the maxillary toothrow is dorsally inset relative to the remainder of the palate in several exclusively longirostrine crocodylians, all of which have been recovered as “gavialoids”, e.g. *Gavialis gangeticus* (Fig. 66B), *Piscogavialis jugaliperforatus* (SMNK 1282 PAL), *Eosuchus minor* (USNM 299730), and ‘*Tomistoma*’ *dowsoni* (NHMUK R4769).

159. Maxilla, size of foramen for palatine ramus of cranial nerve V: small or absent, less than half diameter of 6th maxillary alveolus (0); large, equal to or greater than half diameter of 6th maxillary alveolus (1) (after Brochu, 1997a [111]; Groh, 2020 [136]).

The maxillary foramen for the palatine ramus of cranial nerve V is usually the largest of a linear series of foramina adjacent to the maxillary toothrow, posterior to the level of the 5th maxillary

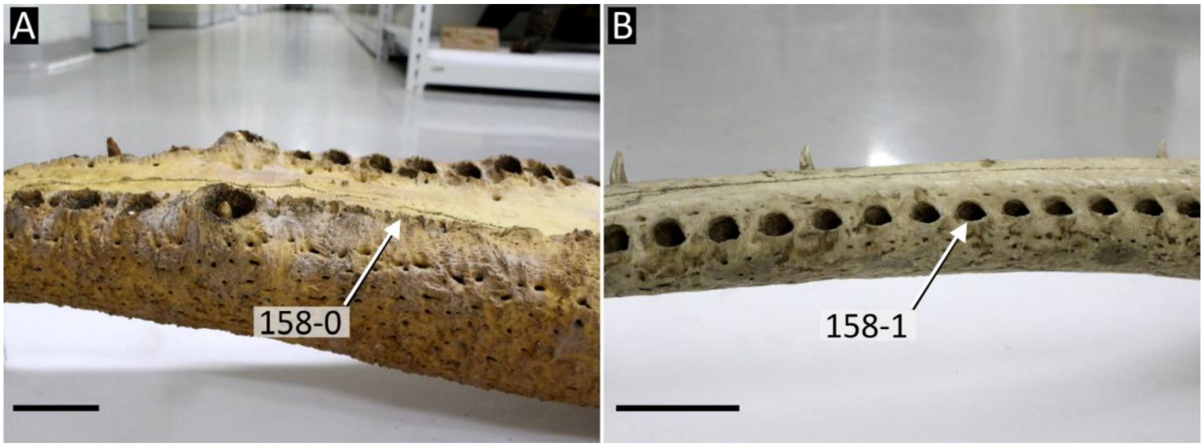

Figure 66: Lateral view of the cranial rostrum showing differences in elevation between the alveolar walls of: **A**, *Tomistoma schlegelii* (NHMUK 1894.2.21.1); **B**, *Gavialis gangeticus* (NHMUK 1974.3009). All scale bars = 5 cm.

alveolus (Fig. 67). In most crocodylians, this foramen is inconspicuously small, e.g. all species of *Crocodylus* (Fig. 67A). In several 'basal' crocodyloids, this foramen is notably larger, e.g. '*Crocodylus*' *affinis* (Fig. 67B), A*siatosuchus germanicus* (HLMD Me 5652), and *Asiatosuchus depressifrons* (IRScNB R251). A slit-like, but enlarged foramen is also found in *Thecachampsa sericodon* (USNM 24938). This character is modified from its original formulation by Brochu (1997b) only by the quantification of foramen size by comparison with the adjacent maxillary alveolus, following Groh et al. (2020).

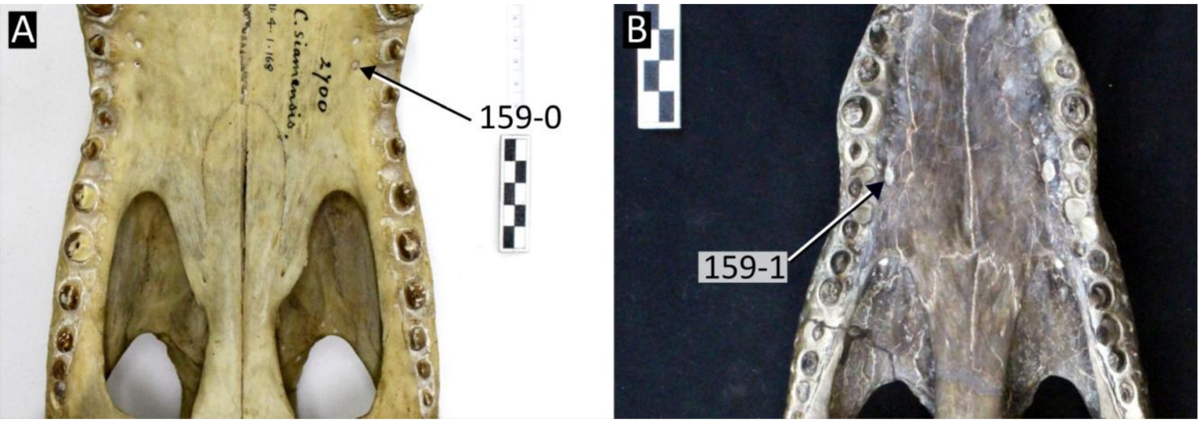

Figure 67: Ventral view of the palate showing variation in cranial nerve V in **A**, *Crocodylus siamensis* (NHMUK 1921.4.1.168); and **B**, *'Crocodylus' affinis* (USNM 18171). Scale bars = cm.

Palatine

160. Palatine, anterior process shape: rounded or quadrangular (0); wedge shaped (i.e. forms a 'V' shape anteriorly) (1) (after Brochu, 1997a [118]).

The anterior palatine process is broadly rounded or squared-off at its anterior end in *Bernissartia fagesii* (IRScNB 1538) and most eusuchians (Fig. 68A–C). This condition contrasts with the anteriorly acute, wedge-shaped palatine process that occurs almost exclusively in longirostrine crocodylians, e.g. *Mecistops cataphractus* (Fig. 68D), *Gavialis gangeticus* (Fig. 68F), *Tomistoma schlegelii* (NHMUK 1894.2.21.1), *Brochuchus pigotti* (NHMUK R7729), and *Baru darrowi* (Willis et al. 1990: fig.1).

161. Palatine, invagination of anterior process: absent (0); present (1) (after Brochu, 1997a [108]; Brochu, 2010 [84]; Delfino and De Vos, 2010).

The derived character state describes the invagination of a short posterior projection of the maxilla into the palatine anterior process that occurs in several crocodylians. In taxa with a broad palatine process (160-0), this results in a heart-shaped palatine process, e.g. *Paleosuchus* (Fig. 68E) and *Caiman latirostris* (NHMUK 86.10.4.2, MACN PV 1420, FMNH 9713). By contrast, taxa with wedge-shaped palatine processes (160-1) exhibit a narrow, bifurcated process (Fig. 68F), e.g. *Eosuchus lerichei* (IRScNB R49), *Dollosuchoides densmorei* (Brochu, 2007b, fig.3), and *Maomingosuchus petrolica* (Shan et al., 2017, fig.4C). In both cases the invagination is considered homologous. Delfino and De Vos (2010) recognised a similar invagination in *Gavialis benjawanicus* (not studied here), which was used to distinguish that species from all other *Gavialis* species. However, all specimens of *Gavialis gangeticus* studied here possess a similar (albeit smaller) invagination, e.g. NHMUK 1974.3009 (Fig. 68F), NHMUK 704, NHMUK 1846.1.7.3. Whereas the palatines are not bifurcated in *Gavialis browni* (Mook, 1932, fig.2), the condition is unknown in *Gavialis lewisi* (YPM 3226).

162. Palatine, anterior process position relative to anterior margin of suborbital fenestra: anterior to, and at the level of more than two full alveoli; (0) anterior to and at the level of two or fewer full alveoli (1); at the same level or posterior to anterior margin of suborbital fenestra (2) (after Willis, 1993 [2]; Brochu, 1997a [110]) (ORDERED).

Willis et al. (1990) noted similarities in the relative length of the palatine process of the crocodyloids *Baru darrowi* and *Brachyuranochampsa eversolei* (Zangerl, 1944). In both taxa, the palatine processes do not exceed the anterior margin of the palatal fenestrae. Willis (1993) discretised this morphological variation as a binary character (see also Brochu (1997b), distinguishing between a palatine process that exceeds the anterior margin of the suborbital fenestra (common to most eusuchians) (Fig. 68A–B), and one that remains posterior to the level of the anterior margin of the suborbital fenestrae (Fig. 68C). The latter condition is described here in character state 2 and is found in several 'basal' crocodyloids, e.g. '*Crocodylus*' *affinis* (UCMP 131090, USNM 18171), *Asiatosuchus depressifrons* (IRScNB R251), *Asiatosuchus germanicus* (HLMD Me 5652),

and *Quinkana fortirostrum* (Molnar, 1982, fig.3). The character has been modified here to describe varying lengths of the palatine process beyond the suborbital fenestra. Most eusuchians have an intermediately long palatine process, which reaches the level of less than two maxillary alveoli beyond the anterior margin of the suborbital fenestra (162-1) e.g. *Hylaeochampsa vectiana* (NHMUK R177), *Borealosuchus sternbergii* (USNM 6533), *Alligator mississippiensis* (Fig. 68G), and *Crocodylus siamensis* (Fig. 68B). By contrast, some crocodylians have a highly elongated palatine process, which reaches the level of two or more alveoli beyond the suborbital fenestra (162-0), e.g. *Melanosuchus niger* (Fig. 68A) and *Gavialis gangeticus* (Fig. 68F).

163. Palatine, palatal bar, lamina projecting into suborbital fenestrae from anterolateral margin: absent (0); present (1) (after Brochu, 1997a [94]).

The anatomical meaning of this character is consistent with Brochu (1999, fig.44E), who recognised that some crocodylians exhibit an anterolateral process/flange of the palatine that projects into the suborbital fenestra (Fig. 68E). This process probably serves as an attachment site for *M. pterygoideus dorsalis* (Holliday et al., 2013). As previously scored (e.g. Brochu et al., 2012), the derived condition characterises both species of *Paleosuchus* (Fig. 68E), some *Alligator* species (e.g. *A. olseni*, MCZ 1887), *Diplocynodon muelleri* (Piras & Buscalioni, 2006, fig.4), and *Borealosuchus sternbergii* (USNM 6533). It is additionally recognised in several further *Diplocynodon* species, e.g. *D. ratelii* (Fig. 68H) and *D. hantoniensis* (Fig. 68I). Furthermore, the condition in *Leidyosuchus canadensis* is scored here as polymophic, given that specimens variably exhibit the flange (Wu et al., 2001a).

164. Palatine, palatal bar, orientation of posterolateral margin realtive to sagittal axis: sub-parallel (angle $< 40°$) (0); flared (angle equal to or greater than $40°$) (1) (after Norell, 1988 [2]; Brochu, 1997a [90]).

The posterolateral margin of the palatal bar is sub-parallel to the sagittal axis in *Bernissartia fagesii* (IRScNB 1538) and most eusuchians, with minimal flare posteriorly (Fig. 68B). In several crocodylians, the palatal bar flares abruptly at its posterior end to produce a shelf (Brochu, 1999, fig.44G). This occurs mainly in alligatoroids, e.g. *Melanosuchus niger* (Fig. 68A), *Alligator mississippiensis* (Fig. 68G), *Paleosuchus trigonatus* (Fig. 68E), and *Mourasuchus atopus* (UCMP 38012), but also in the crocodylid *Osteolaemus tetraspis* (Fig. 68C). Fewer taxa are scored for the derived condition compared to previous studies, because the character states are quantified here with an angular measurement of flare. For example, the palatines flare $<40°$ in *Diplocynodon ratelii* (Fig. 68H) and *Diplocynodon hantoniensis* (Fig. 68I), and thus they were scored for the plesiomorphic condition (differing to Brochu et al., [2012]). The development of pterygoid bullae, or the inflation of the palatines and pterygoids can result in the appearance of flared palatines (Fig.

68D, F). This is distinguished from truly flared palatines in that the lateral margins of the palatal bar remain sub-parallel, and no shelf is developed.

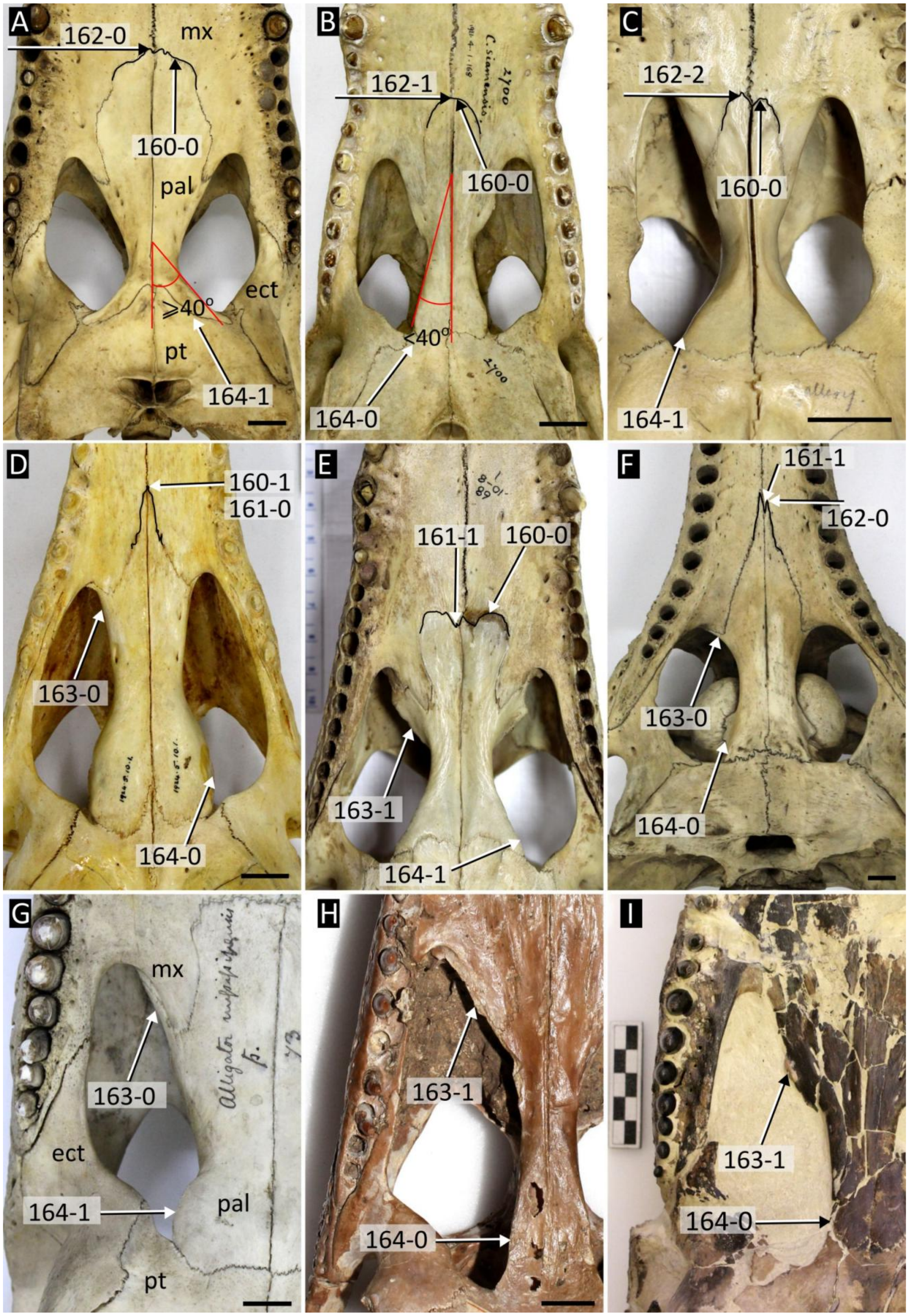

Figure 68: Variation in morphology of the palatine in ventral view. **A**, *Melanosuchus niger* (NHMUK 45.8.25.125); **B**, *Crocodylus siamensis* (NHMUK 1921.4.1.168); **C**, *Osteolaemus tetraspis* (NHMUK 1862.6.30.5); **D**, *Mecistops cataphractus* (NHMUK 1924.5.10.1); **E**, *Paleosuchus trigonatus* (NHMUK 1868.10.8.1); **F**, *Gavialis gangeticus* (NHMUK 1974.3009); **G**, *Alligator mississippiensis* (NHMUK 1873.2.21.1); **H**, *Diplocynodon ratelii* (MNHN SG 539); **I**; *Diplocynodon hantoniensis* (CAMSM TN 907). Abbreviations: **ect**, ectopterygoid; **mx**, maxilla; **pal**, palatine, **pt**, pterygoid. All scale bars = 2 cm.

165. Palatine, ventrolateral ‘ear-shaped’ process projecting from base of prefrontal pillar: absent (0); present (1) (new character, adapted from Wu et al. 2001).

This process refers to a small, elliptical-shaped projection either side of the palatine bar, first noted in *Leidyosuchus canadensis* (Wu et al., 2001a, fig.14C). In their study, Wu et al. (2001a) noted intraspecific variation in the occurrence of such processes in *Leidyosuchus*, and briefly mentioned their occurrence in some specimens of *Caiman*, *Crocodylus*, and *Alligator*. Most eusuchians studied here lack these features at all stages of ontogeny, where known (Fig. 69A), but they occur variably in some crocodylids such as *Crocodylus niloticus* (present in NHMUK 1882.3.7.1 and NHMUK 1934.6.3.1, absent in NHMUK 1894.6.5.33) and *Osteolaemus tetraspis* (present in FMNH 229974, absent in NHMUK 1862.6.30.5). By contrast, they were consistently observed in neotropical *Crocodylus* species. For example, they are more consistently observed in Neotropical *Crocodylus* species. For example, they occur in *C. intermedius* (FMNH 75658, FMNH 75659, FMNH 75662, NHMUK 1851.8.25.29, NHMUK 62.10.19.1) and most specimens of *C. rhombifer* (AMNH 77595, AMNH R154087), although only some specimens of *C. acutus* (FMNH 69884). Other than *Leidyosuchus canadensis*, the only fossil eusuchian found to exhibit these processes in this study was *Agaresuchus fontisensis* (Narváez et al., 2016, fig.2C). Further study of a larger sample of specimens of extant species is required to explore intraspecific variation and the utility of this character.

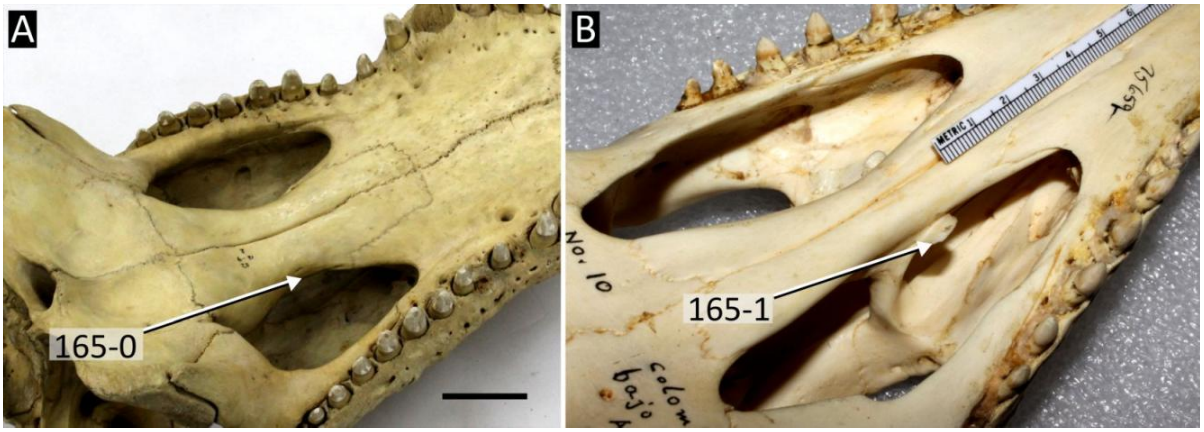

Figure 69: Development of ventrolateral processes of the palatines. **A**, *Crocodylus porosus* (NHMUK 1852.12.9.2); **B**, *Crocodylus intermedius* (FMNH 75659). Scale bar = 5 cm.

Suborbital fenestra

166. Suborbital fenestra, position of anterior margin relative to anterior orbital margin: anterior to (0); level with, or posterior to (1) (after Jouve et al., 2008 [201]).

In *Bernissartia fagesii* (IRScNB 1538) and most eusuchians, the anterior margin of the suborbital fenestra is positioned substantially anterior to that of the orbital margin. As such, only a portion of the orbit can be viewed through the suborbital fenestrae (Fig. 70A, C). Less commonly, the anterior margins of the suborbital fenestra and orbit are approximately level. In this condition, most of the orbit is visible through the suborbital fenestra, e.g. *Gavialis gangeticus* (Fig. 70B), *Hylaeochampsa vectiana* (NHMUK R177), and *Procaimanoidea utahensis* (USNM 15996).

167. Suborbital fenestra, anteromedial margin, intersection of maxilla-palatine suture: at the anteromedial margin (0); at the anterior corner (1) (after Brochu and Storrs, 2012 [187]).

In most eusuchians, the maxilla-palatine suture intersects the suborbital fenestra at its anteromedial margin (Fig. 70A, C). Brochu and Storrs (2012) recognised a new condition, in which this suture intersects the anterior corner of the suborbital fenestra, but this was only recognised in *Mecistops cataphractus* (Fig. 70F). Jouve (2016) scored the condition more widely in crocodylians, noting the same condition in several hylaeochampsids (*Hylaeochampsa vectiana* [Clark and Norell, 1992, fig.5], and *Iharkutuosuchus makadii* [Ösi et al., 2008, fig.1D]) and gavialoids (e.g. *Gavialis gangeticus*, Fig. 70B, *Piscogavialis jugaliperforatus*, SMNK 1282 PAL, *Gryposuchus colombianus*, UCMP 41136), which were similarly observed in this study. The condition is recognised in several additional crocodylians, including *Eosuchus lerichei* (IRScNB R 49), *Thecachampsa sericodon* (USNM 24938), *Thoracosaurus isorhynchus* (MNHN 1902-22), and *Maomingosuchus petrolica* (Shan et al., 2017, fig.4C).

168. Suborbital fenestra, anterolateral margin width, distance from medial edge of the toothrow to fenestral margin: narrow, less than or equal to one alveolus width (0); broader than one alveolar width (usually at least twice alveolar width) (1) (after Jouve et al. 2008 [146], adapted from Wu et al. 2001a).

The derived character state describes a broad lateral margin of the suborbital fenestra formed by the maxilla and ectopterygoid. As noted by Wu et al. (2001a), this area is especiall broad in *Leidyosuchus canadensis*, a condition that is shared by most alligatoroids, e.g. *Eocaiman cavernensis* (Fig. 70H), *Caiman crocodilus* (Fig. 70D), and *Alligator mississippiensis* (Fig. 70E), as well as some crocodyloids, e.g. *Osteolaemus tetraspis* (Fig. 70G) and *Baru wickeni* (QM F16822). By contrast, most crocodyloids as well as gavialoids have narrow lateral margins of the suborbital fenestra, that are typically no more than one alveolar width, e.g. *Gavialis gangeticus* (Fig. 70B), *Crocodylus porosus* (Fig. 70C), and *Mecistops cataphractus* (Fig. 70F).

169. Suborbital fenestra, lateral margin shape: straight (0); projecting medially into fenestra (1) (rephrased from Brochu, 1997a [105]).

170. Suborbital fenestra, contribution of maxilla to medial projection: absent, projection entirely formed by ectopterygoid (0); present (1) (new character, adapted from Brochu, 1997a).

Characters 169 and 170 describe the presence and position of a medial projection into the suborbital fenestra from its lateral wall, and were derived by reductively coding Character 105 in Brochu (1997b). A medial projection (169-1) was recognised in *Bernissartia fagesii* (IRScNB 1538), some alligatoroids, (e.g. *Alligator mcgrewi*, AMNH 7905 and *Eocaiman cavernensis*, Fig. 70H), and the crocodyloids *Osteolaemus tetraspis* (Fig. 70G) and *Trilophosuchus rackhami* (QM F16856). Brochu (1999) noted that the degree to which the maxilla participates varies between taxa, which is herein captured in Character 170. Whereas the process is formed entirely by the ectopterygoid (170-0) in the crocodyloids *Osteolaemus tetraspis* (Fig. 70G) and *Trilophosuchus*, the maxilla contributes to this process (170-1) in the alligatoroids *Eocaiman cavernensis* (Fig. 70H) and *Alligator mcgrewi* (AMNH 7905).

171. Suborbital fenestra, posterolateral margin shape at ectopterygoid-pterygoid suture intersection: straight (0); bowed anteromedially (1) (after Brochu, 1997a [88]; Brochu, 2010 [119]).

In *Bernissartia fagesii* (IRScNB 1538) and most eusuchians, the posterior margin of the suborbital fenestra is rounded (Brochu, 1999) (Fig. 70B, D). By contrast, some eusuchians exhibit a convexity in the fenestral margin around the level of the intersection of the ectopterygoid-pterygoid suture, which produces an embayment near the posterior corner of the suborbital fenestra (Brochu, 1999). Among eusuchians, this condition occurs in some *Alligator* species (e.g. *A. mississippiensis*, Fig. 70E and *A. sinensis*, USNM 292078), and most *Crocodylus* species, e.g. *C. porosus* (Fig. 70C), *C. acutus* (NHMUK 1975.997), and *C. johnstoni* (QM J39230).

172. Suborbital fenestra, posterior margin, intersection of palatine-pterygoid suture: at the posterior corner (0); on the posteromedial margin (1) (after Brochu, 1997a [85]).

This character has received minor changes to its wording. Originally, the plesiomorphic condition described the palatine-pterygoid suture being "nearly at" the posterior margin of the suborbital fenestra (Brochu, 1997b, Character 85). In the revised format, taxa scored for the plesiomorphic condition must exhibit a pterygoid-palatine suture which intersects precisely at the posterior corner of the suborbital fenestra (Fig. 70E). Consequently, by contrast with previous studies (e.g. Brochu et al., 2012), some taxa are newly scored for the derived condition, e.g. *Paleosuchus trigonatus* (Fig. 70I), *Procaimanoidea utahensis* (USNM 15996), and *Allodaposuchus precedens* (MMS/VBN-12-10-A).

173. Ectopterygoid, anterior extent relative to maxillary alveoli: reaches the level of two or fewer alveoli (0); more than two alveoli (1) (after Jouve, 2016 [91], Lee and Yates, 2018 [28]).

The ectopterygoid extends beyond two maxillary alveoli in most eusuchians, e.g. *Borealosuchus sternbergii* (Fig. 70A), *Boverisuchus vorax* (FMNH PR 399), *Crocodylus porosus* (Fig. 70C), *Caiman crocodilus* (Fig. 70D), and *Alligator mississippiensis* (Fig. 70E). By contrast, the ectopterygoid reaches fewer than two maxillary alveoli in *Bernissartia fagesii* (IRScNB 1538), some gavialoids (e.g. *Gavialis gangeticus*, Fig. 70B), *Tomistoma schlegelii* (NHMUK 1894.2.21.1), and some alligatoroids, e.g. *Brachychampsa montana* (UCMP 133901) and *Melanosuchus niger* (NHMUK 45.8.25.125).

174. Ectopterygoid, anterior extent relative to anteroposterior length of suborbital fenestra: less than two thirds of fenestra length (0); equal to or greater than two thirds of fenestra length (1) (after Brochu and Storrs, 2012 [185]).

The anterior extent of the ectopterygoid relative to the anteroposterior length of the suborbital fenestra is independent of ectopterygoid extent relative to maxillary alveoli (Character 173). This is evidenced by the occurrence of all possible combinations of characters 173 and 174 in multiple taxa examined here. For example, in *Gavialis gangeticus* (Fig. 70B), *Brachychampsa montana* (UCMP 133901), and *Stangerochampsa mccabei* (Wu et al., 1996, fig.1B), the ectopterygoid anteriorly reaches the level of fewer than two alveoli (173-0), but the anterior ectopterygoid ramus still forms more than two thirds the length of the suborbital fenestra (174-1). By contrast, in *Borealosuchus sternbergii* (Fig. 70A), *Borealosuchus formidabilis* (Erickson, 1976, fig.5), and *Diplocynodon hantoniensis* (NHMUK 30392), the ectopterygoid is adjacent to two or more maxillary alveoli (173-1), but the anterior ectopterygoid ramus forms around half the anteroposterior length of the suborbital fenestra (174-0).

Ectopterygoid

175. Ectopterygoid, contact with maxillary toothrow, forming the medial wall of at least one maxillary alveolus: absent, ectopterygoid-maxilla suture anteromedially orientated and separated from toothrow margin (0); absent, ectopterygoid-maxilla suture parallel and adjacent to medial toothrow margin (1); present (2) (after Norell, 1988 [19]; Brochu, 1997a [91]; Jouve, 2016 [91]) (ORDERED).

Most studies follow the original formulation of this character by Brochu (1997b) (e.g. Brochu et al., 2012; Lee & Yates, 2018; Narváez et al., 2016; Salas-Gismondi et al., 2016; Salas-Gismondi et al., 2015; Salas-Gismondi et al., 2019): "*Ectopterygoid abuts maxillary toothrow (0); or maxilla broadly separates ectopterygoid from maxillary tooth row (1)*" (Brochu, 1997b, Character 91). The condition in which the maxilla broadly separates the ectopterygoid from the toothrow (e.g. Fig. 71H) has long been considered diagnostic of Alligatoroidea, and strongly contrasts with the fully

abutting ectopterygoid of crocodylids (e.g. Fig. 71R) (Norell et al., 1994, fig.6; Brochu, 1999, fig.24A–B). Accordingly, alligatoroids are scored for character state 1 in most matrices, with gavialoids, "tomistomines", *Borealosuchus*, and *Bernissartia fagesii* all described as having an ectopterygoid which abuts the maxillary toothrow, as in crocodylids. However, this is inaccurate, as the ectopterygoid is separated by the maxilla in many of the aforementioned taxa, e.g. *Bernissartia fagesii* (Fig. 71A) (see also Martin et al., 2020, fig.2D), *Borealosuchus sternbergii* (Fig. 71B–C), *Tomistoma schlegelii* (Fig. 71D), and *Gavialis gangeticus* (Fig. 71E). This was alluded to by Delfino et al. (2005), who indicated that it is inappropriate to describe *Gavialis gangeticus* and *Crocodylus niloticus* as sharing the same condition, and that the character should be better defined. Jouve (2016) evidently reached the same conclusion, as he introduced a new character state that distinguished several gavialoids, "tomistomines", 'basal' crocodyloids, and all *Borealosuchus* species, from the alligatoroid and crocodylid conditions. According to Jouve (2016), these taxa exhibit an "*ectopterygoid* (that) *does not abut the maxillary teeth, and the ectopterygoid-maxillary suture parallels the toothrow*". The difference between the condition in taxa such as *Gavialis*, *Tomistoma*, and *Borealosuchus*, to that of an alligatoroid such as *Alligator mississippiensis* (Fig. 71H), is only a matter of degree. The important distinction is between those with an ectopterygoid-toothrow contact, and those without. Furthermore, the difference in degree of separation can be attributed, in part, to differences in the width of the lateral margin of the suborbital fenestra, which, as established earlier in Character 168, tends to be wider in alligatoroids (Wu et al., 2001a) (Fig. 71I–J). Consequently, most of the taxa scored for the new condition by Jouve (2016) have been changed to character state 0 here, i.e. the same condition exhibited by alligatoroids. Nevertheless, Jouve's additional character state has been co-opted to describe a distinct condition (175-1) that occurs mainly in crocodyloids (e.g. *Asiatosuchus depressifrons*, Fig. 71K, *Kambara implexidens*, Fig. 71L and *Australosuchus clarkae*, UCMP 71396), but also *Diplocynodon ratelii* (Fig. 71M), *Boverisuchus vorax* (Fig. 71N), and *Piscogavialis jugaliperforatus*(Fig. 71O). In these taxa, the ectopterygoid incipiently contacts the toothrow, barely separated by a thin slither of the maxilla. The revised character is ordered given that this character state is considered intermediate (175-1) between a widely separated ectopterygoid (175-0) and a fully abutting ectopterygoid (175-2), the latter characterising most crocodylids (Fig. 71P–T) as in previous studies.

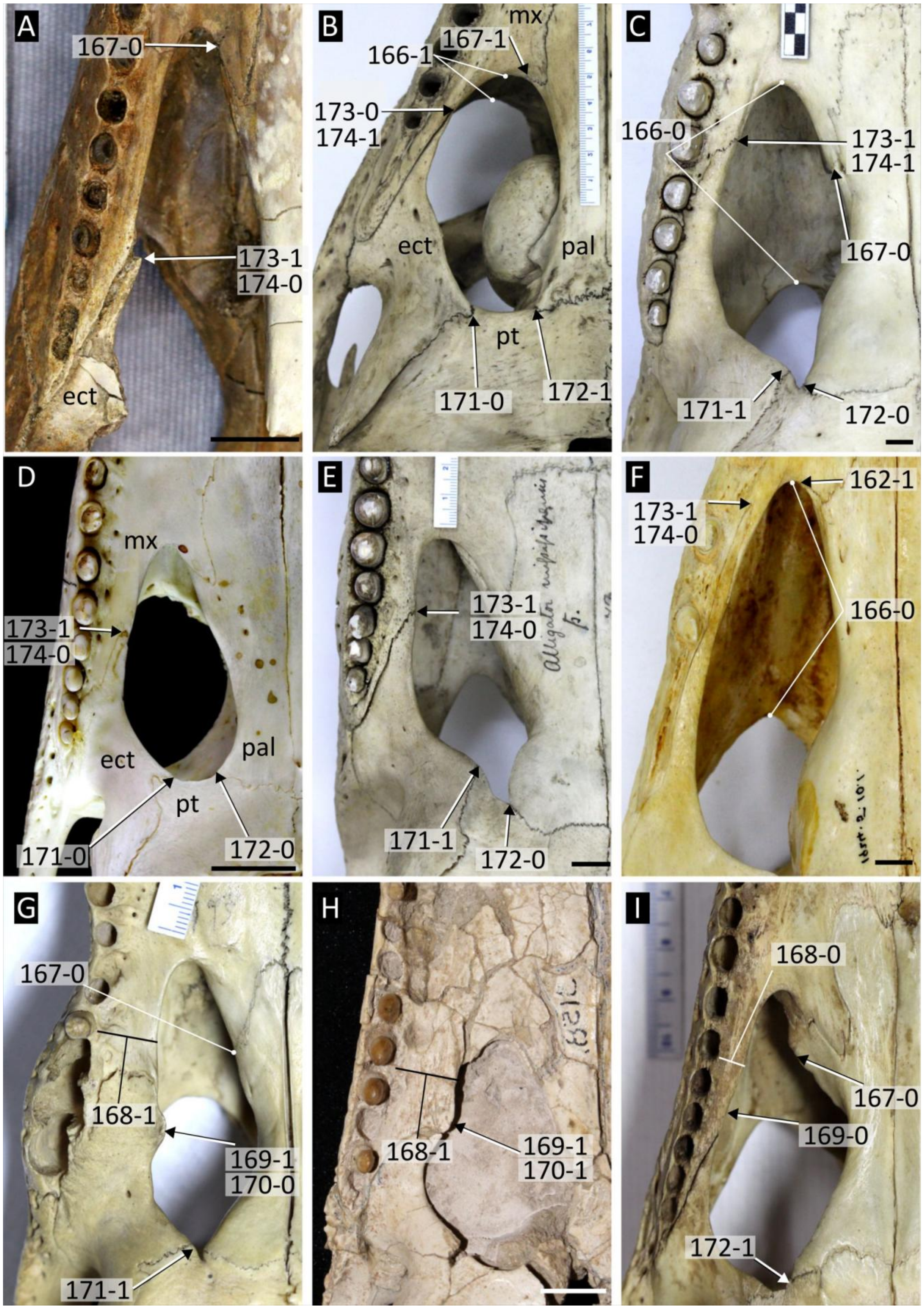

Figure 70: Sutural relationships and morphology of the suborbital fenestra in selected crocodylians. **A**, *Borealosuchus sternbergii* (UCMP 126099); **B**, *Gavialis gangeticus* (NHMUK 1974.3009) (digitally reversed); **C**, *Crocodylus porosus* (NHMUK 1852.12.9.2); **D**, *Caiman crocodilus chiapasius* (FMNH 73694); **E**, *Alligator mississippiensis* (NHMUK 1873.2.21.1); **F**, *Mecistops cataphractus* (NHMUK 1924.5.10.1) (digitally reversed); **G**, *Osteolaemus tetraspis* (NHMUK 1862.6.30.5); **H**, *Eocaiman cavernensis* (AMNH 3158) (digitally reversed); **I**, *Paleosuchus trigonatus* (NHMUK 1868.10.8.1). Abbreviations: **ect**, ectopterygoid; **mx**, maxilla; **pal**, palatine; **pt**, pterygoid. All scale bars = 1 cm.

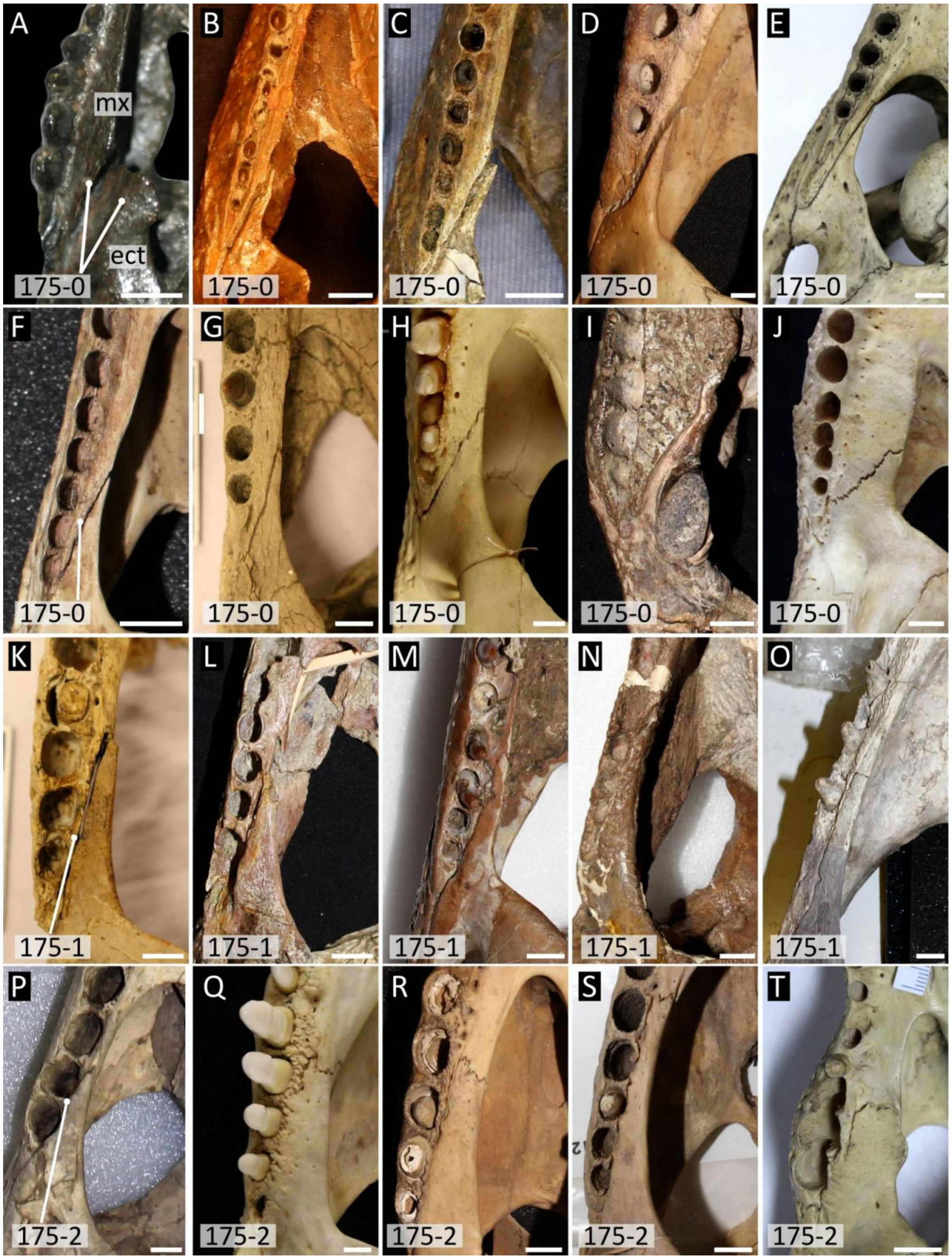

Figure 71: Variation in the ectopterygoid-maxilla suture. **A**, *Bernissartia fagesii* (IRSNB 1538); **B**, *Borealosuchus sternbergii* (USNM 6533); **C**, *Borealosuchus sternbergii* (UCMP 126099); **D**, *Tomistoma schlegelii* (USNM 211323); **E**, *Gavialis gangeticus* (NHMUK 1974.3009); **F**, *Paleosuchus trigonatus* (NHMUK 1868.10.8.1); **G**, *Eosuchus lerichei* (IRSNB R 49); **H**, *Alligator mississippiensis* (AMNH 71621); **I**, *Navajosuchus mooki* (AMNH 6780); **J**, *Caiman latirostris* (FMNH 9713); **K**, '*Crocodylus*' *depressifrons* (IRSNB R 251); **L**, *Kambara implexidens* (QM 29662); **M**, *Diplocynodon ratelii* (MNHN SG 539); **N**, *Boverisuchus vorax* (FMNH PR 399); **O**, *Piscogavialis jugaliperforatus* (SMNK 1282 PAL); **P**, *Thecachampsa sericodon* (USNM 25243); **Q**, *Crocodylus rhombifer* (AMNH R 154087); **R**, *Crocodylus porosus* (NHMUK 1852.12.9.2); **S**, *Crocodylus niloticus* (NHMUK 1934.6.3.1); **T**, *Osteolaemus tetraspis* (NHMUK 1862.6.30.5). Abbreviations: **ect**, ectopterygoid; **mx**, maxilla. All scale bars = 1 cm.

176. Ectopterygoid, morphology of anterior maxillary ramus on lateral suborbital fenestra wall: acute, tapering to a single point (0); forked (1) (after Brochu, 1997a [109]).

The anterior tip of the ectopterygoid forms an acute point in most crocodylians (Fig. 72A). In *Mecistops cataphractus* (NHMUK 62.6.30.8), *Brochuchus pigotti* (NHMUK R7729), *Crocodylus palaeindicus* (NHMUK 39795), and some individuals of all extant *Crocodylus* species, the anterior margin bears a cleft (Fig. 72B) (Brochu, 2000). Following the observations of Brochu et al. (2010), the condition is scored as polymorphic in extant *Crocodylus* species.

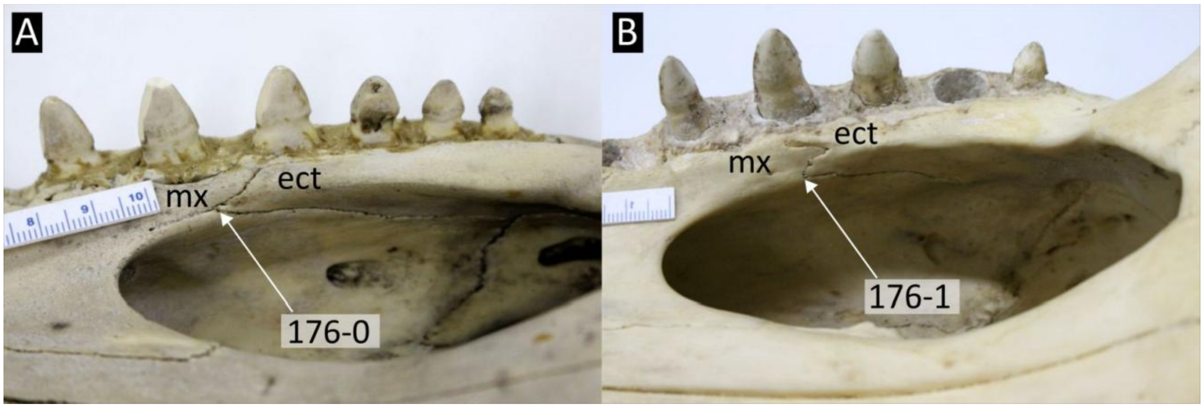

Figure 72: Variation in morphology of the ectopterygoid tip in crocodylians. **A**, *Crocodylus acutus* (NHMUK 1975.997); **B**, *Crocodylus niloticus* (NHMUK 1934.6.3.1) (digitally reversed). Abbreviations: **ect**, ectopterygoid; **mx**, maxilla. Scale bars = cm.

177. Ectopterygoid, anterior maxillary ramus: contacts suborbital fenestra (0); separated from the suborbital fenestra by the maxilla (1) (after Brochu and Storrs, 2012 [186]).

In most crocodylians, the anterior tip of the ectopterygoid contributes to the lateral margin of the suborbital fenestra, e.g. *Crocodylus porosus* (Fig. 73A). Less commonly, the ectopterygoid may be blocked from the suborbital fenestra at its anterior tip by a thin posterior projection of the maxilla. This condition occurs in *Mecistops cataphractus* (Fig. 73B), some mekosuchines (e.g. *Mekosuchus sanderi* [QM F31188], *Kambara implexidens* [QM F29662], *Baru wickeni* [QM F16822], and *Baru huberi* [QM F31063]), and is polymorphic in *Crocodylus johnstoni* (e.g. present in QM J39230, absent in QM J45309).

178. Ectopterygoid, position relative to maxillary alveoli: restricted to medial side (0); forming posterior and lateral margins (1) (new character, adapted from Clark and Norell, 1992).

Clark and Norell (1992) recognised that the putative ‘palatal foramen’ of *Hylaeochampsa vectiana* is actually a highly enlarged posterior maxillary alveolus (Fig. 74B), as is also the case in *Iharkutosuchus makadii* (Ösi, 2008). Furthermore, the ectopterygoid partially roofs this alveolus in both taxa, forming the posterior and lateral alveolar walls (178-1) (Fig. 74B) (Ösi, 2008, fig.1D).

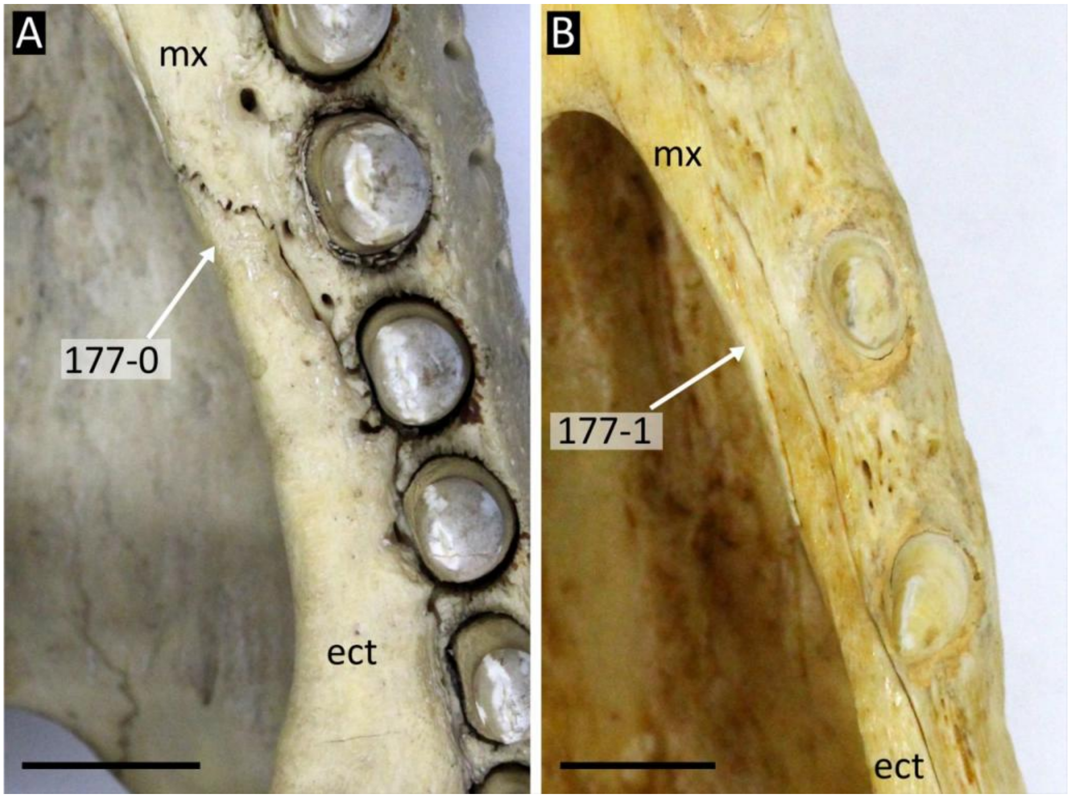

Figure 73: Relationship of the ectopterygoid anterior tip and suborbital fenestra. **A**, *Crocodylus porosus* (NHMUK 1852.12.9.2) (digitally reversed); **B**, *Mecistops cataphractus* (NHMUK 1924.5.10.1). Abbreviations: **ect**, ectopterygoid; **mx**, maxilla. All scale bars = 1 cm.

In *Bernissartia fagesii* (IRScNB 1538) and all other eusuchians, the ectopterygoid is completely excluded from the posterior and lateral margins of the toothrow (178-0), e.g. *Alligator mississippiensis* (Fig. 74A).

179. Maxilla, non-dentigerous posterior process between jugal and ectopterygoid: short, less than anteroposterior length across last three maxillary alveoli (0); long, equal to or greater than anteroposterior length across last three maxillary alveoli (1) (after Jouve et al., 2008 [172]).

In all crocodylians the maxilla forms an acute posterior process between the jugal and ectopterygoid (Fig. 75). In most species this process is short, approximately the length of one or two maxillary alveoli e.g. *Alligator mississippiensis* (Fig. 75A). This process is notably longer in *Mecistops cataphractus* (NHMUK 1924.5.10.1), and several gavialoids, e.g. *Gavialis gangeticus* (Fig. 75B), *Piscogavialis jugaliperforatus* (SMNK 1282 PAL), and *Argochampsa krebsi* (NHMUK R36872).

180. Ectopterygoid, dorsal extent along medial surface of postorbital bar: large, extends dorsal to level of ventral orbital margin (0); small, level with or ventral to level of ventral orbital margin (1) (after Brochu, 1997a [133]).

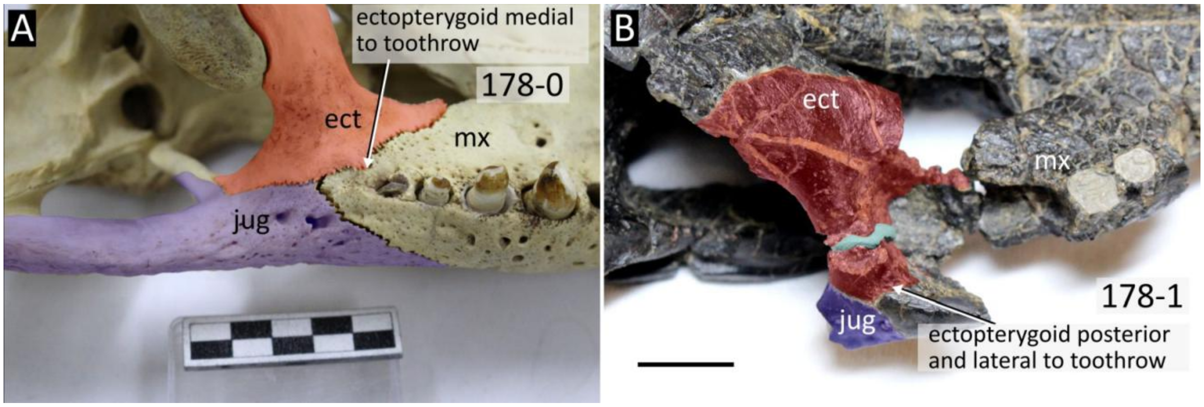

Figure 74: Ventrolateral view of the posterior maxillary toothrow showing the relationship of the ectopterygoid to the toothrow. **A**, *Caiman latirostris* (NHMUK 1897.12.31.1); **B**, *Hylaeochampsa vectiana* (NHMUK PV R 177). Abbreviations: **ect**, ectopterygoid; **jug**, jugal; **mx**, maxilla. Scale bar in B = 1 cm.

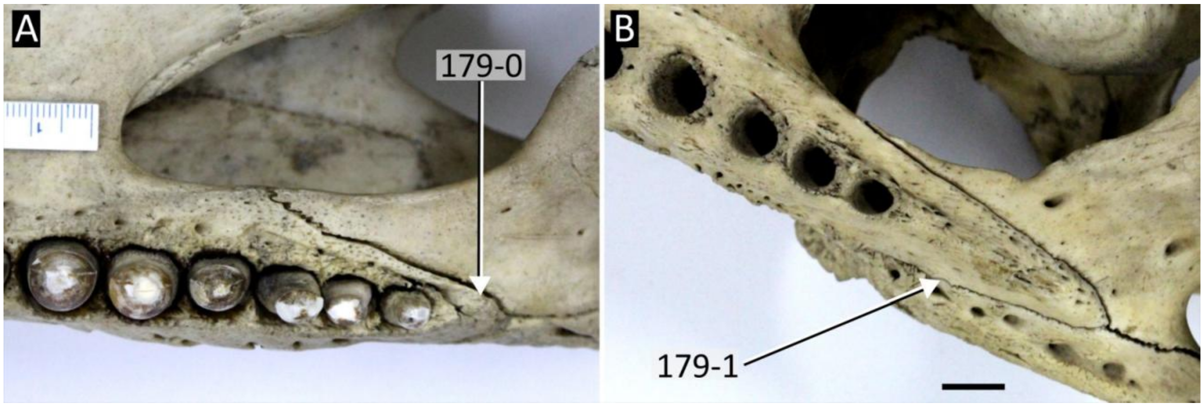

Figure 75: Ventral views of the suborbital fenestra showing variation in size of the maxillary non-dentigerous process in **A**, *Alligator mississippiensis* (NHMUK 1873.2.21.1); and **B**, *Gavialis gangeticus* (NHMUK 1974.3009). Scale bar = 1 cm.

The ectopterygoid sutures to the medial surface of the jugal, ascending the postorbital bar in most neosuchians, dorsal to the level of the ventral orbital margin (180-0) (Brochu, 1999). This occurs in *Bernissartia fagesii* (IRScNB 1538), *Hylaeochampsa vectiana* (NHMU R177), planocraniids (e.g. *Boverisuchus vorax*, FMNH PR 399), crocodyloids (e.g. *Crocodylus acutus*, Fig. 76A), gavialoids (e.g. *Gavialis gangeticus*, Fig. 76C) and *Tomistoma schlegelii* (NHMUK 1894.2.21.1). The dorsal extent of the ectopterygoid is low in all extant alligatorids (180-1) and several fossil alligatoroids (e.g. *Brachychampsa montana*, UCMP 133901), not exceeding the level of the ventral margin of the orbit (Fig. 76B). All species of *Borealosuchus* were previously scored for the plesiomorphic condition, where preserved (Brochu et al., 2012), but the derived condition can be observed in *Borealosuchus sternbergii* (UCMP 126099).

181. Ectopterygoid, morphology of posterior process on the medial jugal surface: acute, extends beyond level of posterior margin of postorbital bar (0); acute, terminating before posterior margin

of postorbital bar (1); rounded (2) (after Norell, 1989 [9]; Jouve, 2004 [146]; Jouve, 2016 [243]) (ORDERED).

In addition to the dorsomedial ascending ramus of the ectopterygoid (Character 180), some taxa exhibit a posteromedial process, which runs along the medial surface of the jugal arch (Fig. 76). Norell (1989), and later Jouve (2016) characterised this morphological variation in a binary, presence/ absence character. One additional intermediate character state is included here, and the character is ordered. Taxa exhibiting a long posterior process that exceeds the level of the postorbital bar (181-0) include *Bernissartia fagesii* (IRScNB 1538), most "gavialoids" (e.g. *Gavialis gangeticus* [Fig. 76C] and *Eogavialis africanum* [YPM 6263]), some crocodyloids (e.g. *Trilophosuchus rackhami* [QM F16856] and *Kambara implexidens* [QM F29662]), and some caimanines (e.g. *Mourasuchus atopus* [UCMP 38012] and *Acresuchus pachytemporalis* [UFAC 2507]). By contrast, the process is absent (182-2) in all extant crocodylids (e.g. *Crocodylus acutus* [Fig. 76A] and *Osteolaemus tetraspis* [NHMUK 1862.6.30.5]), and most "tomistomines" (e.g. *Tomistoma schlegelii* [NHMUK 1894.2.21.1] and *Thecachampsa sericodon* [USNM 24938]). In the intermediate condition (181-1), the ectopterygoid forms a posteromedial process, but it does not exceed the posterior margin of the postorbital bar (Fig. 76B). This occurs in some *Diplocynodon* species (e.g. *D. ratelii* [MNHN SG 539] and *D. hantoniensis* [NHMUK OR 30392]), *Eosuchus lericheri* (IRScNB R49), and all extant caimanines, e.g. *Caiman latirostris* (Fig. 76B).

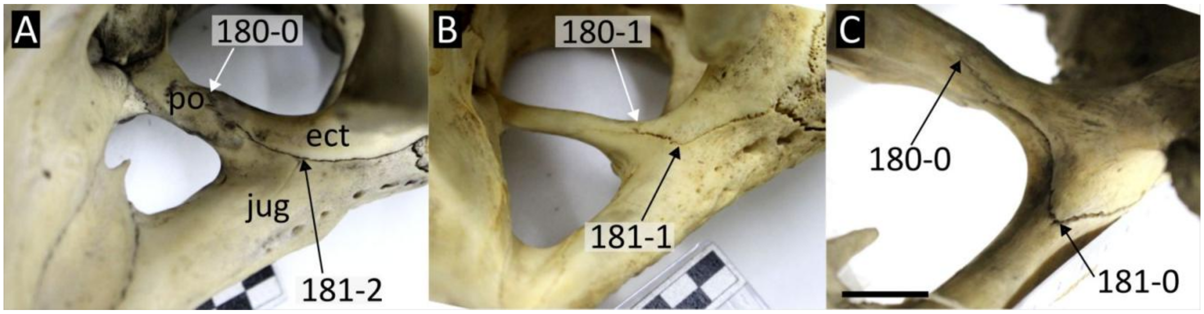

Figure 76: Ventromedial view of the temporal bar in selected crocodylians. **A**, *Crocodylus acutus* (NHMUK 1975.997); **B**, *Caiman latirostris* (NHMUK 1897.12.31.1); **C**, *Gavialis gangeticus* (NHMUK 1974.3009) (digitally reversed). Abbreviations: **ect**, ectopterygoid; **jug**, jugal; **po**, postorbital. Scale bar in C = 2 cm, all other scale bars = cm.

182. Jugal, extent of ectopterygoid and maxilla on medial surface, anterior to the postorbital bar: minimal, jugal visible (0); extensive, covering medial surface of jugal (1) (new character, based on personal observations).

In most crocodylians, a large portion of the medial jugal surface is exposed anterior to the postorbital bar in between the sutural contacts of the ectopterygoid and maxilla (Fig. 77A). By contrast, the ectopterygoid and maxilla cover most of the medial jugal surface in all *Mekosuchus* species,

where preserved (e.g. *M. inexpectatus* [Fig. 77B] and *M. sanderi* [QM F31166]), and also in *Trilophosuchus rackhami* (QM F16856).

183. Quadratojugal, anterior process on medial surface of lower temporal bar: present (0); absent (or very modest) (1) (Brochu, 1997a [83]).

In *Bernissartia fagesii* (IRScNB 1538) and most eusuchians, the medial surface of the jugal forming the lower temporal bar bears an anterior process of the quadratojugal (Fig. 77B). This process occurs in all “gavialoids”, alligatoroids, and some crocodyloids, e.g. *Mekosuchus inexpectatus* (Fig. 77B), ‘*Crocodylus*’ *affinis* (USNM 1811), and *Asiatosuchus depressifrons* (IRScNB IG 9912). By contrast, the process is absent in all extant crocodylids and some “tomistomines”, e.g. *Tomistoma schlegelii* (NHMK 1894.2.21.1).

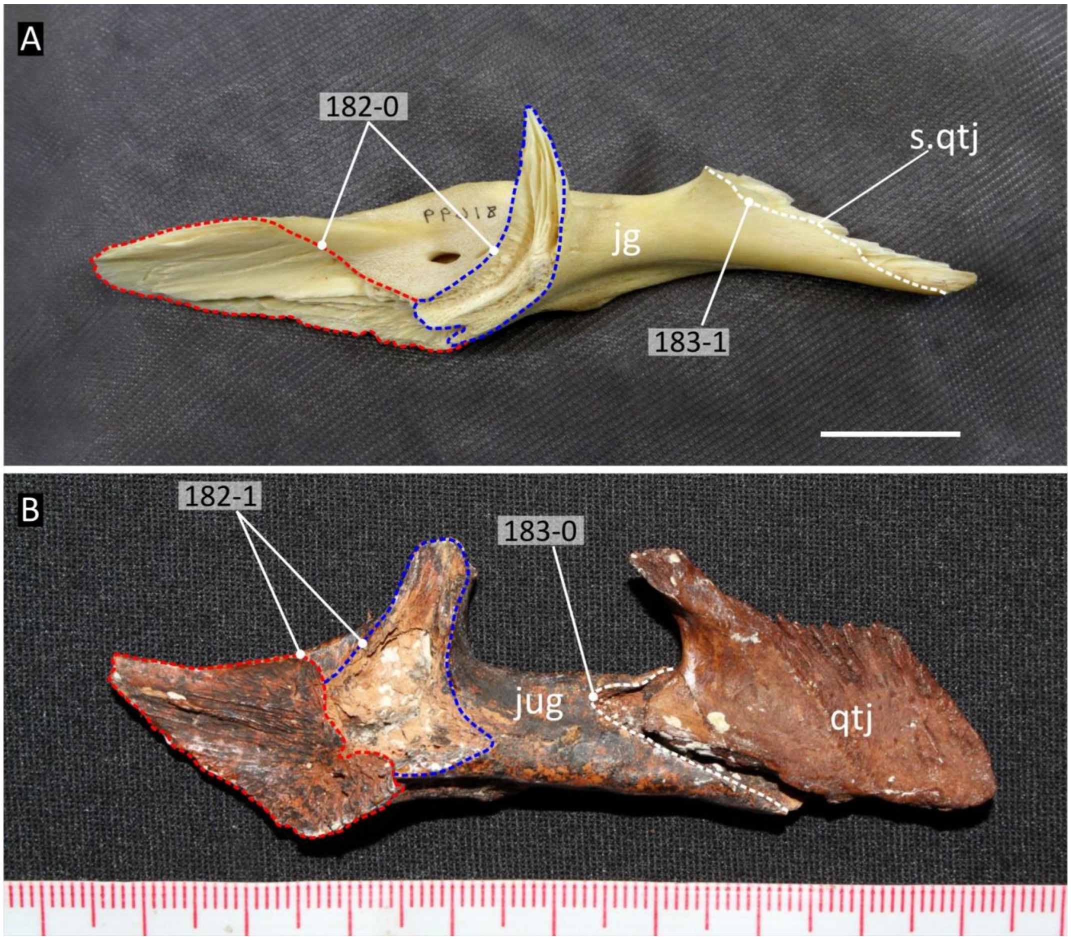

Figure 77: Isolated jugals in medial view showing the development of the anterior quadratojugal process, and extent of ectopterygoid and maxilla sutures. **A**, *Crocodylus acutus* (UCMP 81699); **B**, *Mekosuchus inexpectatus* (MNHN NCP 06). Abbreviations: **jug**, jugal; **qtj**, quadratojugal. Scale bar = 1 cm.

184. Jugal, morphology of medial foramen anterior to postorbital bar: small foramen (0); large recess (1) (Brochu, 1997a [120]).

As noted by Brochu (1997a), the medial surface of the jugal is perforated by a foramen in all eusuchians (Fig. 78). This foramen usually remains small, as in all alligatoroids (Fig. 78A) and "gavialoids". By contrast, the foramen is enlarged to the extent that it forms a deep recess in *Borealosuchus sternbergii* (Brochu, 1997a, fig.5A), all extant crocodylids, and most "tomistomines", e.g. *Tomistoma schlegelii* (Fig. 78B) and *Thecachampsa sericodon* (USNM 24938). A measure of foramen size was not used because the distinction between the two character states is clear, and intermediate sized foramina are not present in any of the taxa in this dataset.

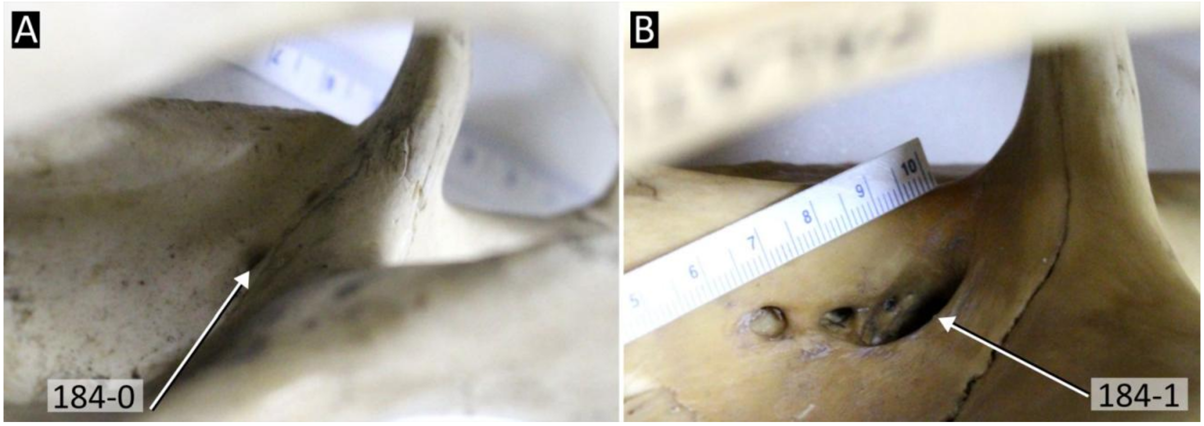

Figure 78: Medial view of the right jugal through the orbit in **A**, *Alligator mississippiensis* (NHMUK 1873.2.21.1); **B**, *Tomistoma schlegelii* (NHMUK 1894.2.21.1). Scale bars = cm.

185. Ectopterygoid-pterygoid suture, shape (at maturity): straight (0); kinked (i.e. with 'flexure') (1) (after Brochu, 1997a [116]).

In juvenile individuals of all extant crocodylians, the pterygoid-ectopterygoid suture posterior to the suborbital fenestra is prominently kinked (Brochu, 1999). This results from a process of the pterygoid that projects into the descending process of the ectopterygoid. Brochu (1999) recognised that whereas adult individuals of all extant caimanines paedomorphically retain this feature (e.g. *Caiman latirostris*, Fig. 79B), all other crocodylians lose it at maturity (e.g. *Crocodylus acutus*, Fig. 79C). Accordingly, Brochu (1999) recovered this feature as a synapomorphy of the crown group of caimanines, also present in some extinct species, e.g. *Purussaurus neivensis* (UCMP 45719). This condition is also present in some species of the 'basal' alligatoroid genus *Diplocynodon*, e.g. *D. deponiae* (Delfino & Smith, 2012) and *D. hantoniensis* (Chapter 2, Fig. 79A).

186. Ectopterygoid, posterior extent on pterygoid flange: reaches posterior tip (0); does not reach posterior tip (1) (after Norell, 1988 [32]; Brochu, 1997a [149]).

The descending process of the ectopterygoid underlies the pterygoid flange (Fig. 80). In almost all eusuchians, the ectopterygoid terminates before reaching the posterior tip of the pterygoid flange,

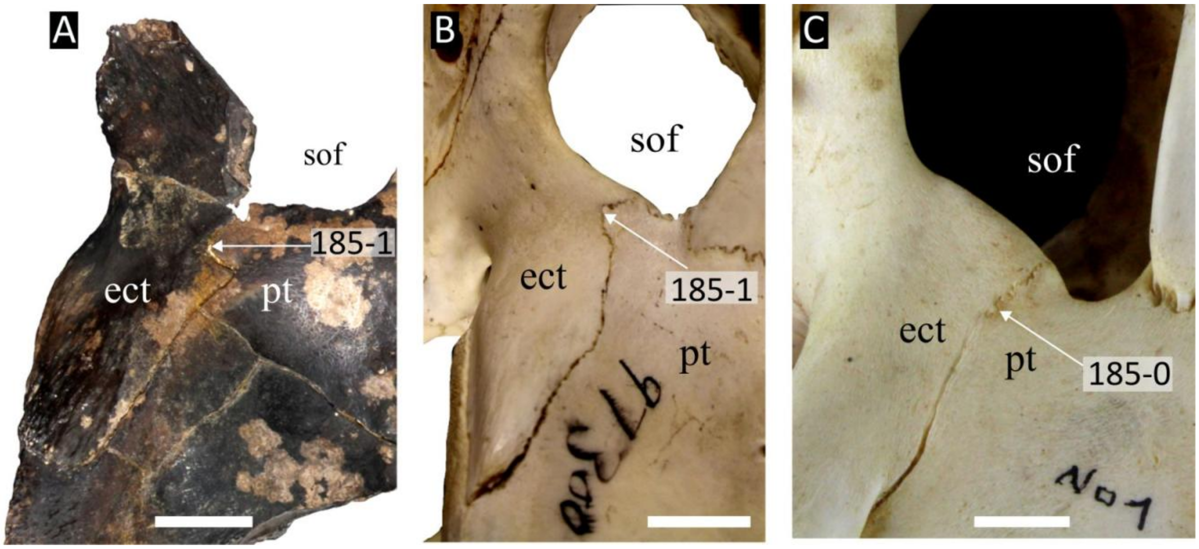

Figure 79: Ventral view of the ectopterygoid-pterygoid suture in selected crocodylians. **A**, *Diplocynodon hantoniensis* (NHMUK); **B**, *Caiman yacare* (AMNH 97300); **C**, *Crocodylus acutus* (FMNH 69884). Abbreviations: **ect**, ectopterygoid; **pt**, pterygoid; **sof**, suborbital fenestra. Scale bars = 2 cm.

e.g. *Crocodylus siamensis* (Fig. 80C). As in previous datasets, the plesiomorphic condition is rare, occurring only in *Bernissartia fagesii* (Fig. 80A) and *Penghusuchus pani* (Shan et al., 2009, fig.3b). This condition was previously considered to be present in the 'Glen Rose Form' (e.g. Brochu, 1999). However, although the ectopterygoid almost reaches the posterior end of the pterygoid in this taxon, it nonetheless terminates shortly before the posterior tip (Fig. 80B).

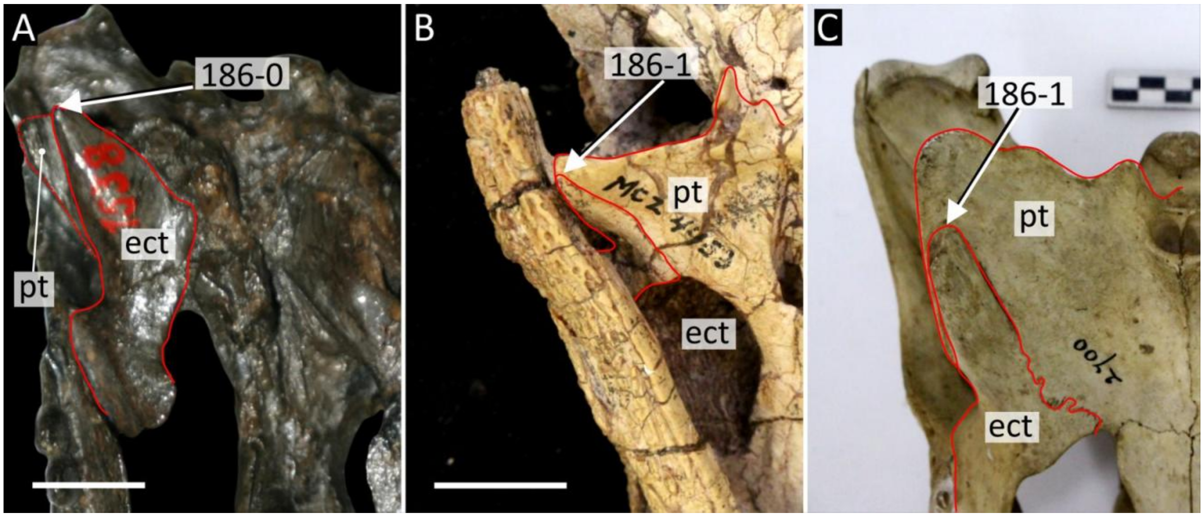

Figure 80: Ventral view of the pterygoids showing variation in posterior extent of the ectopterygoid on the pterygoid. **A**, *Bernissartia fagesii* (IRScNB 1538); **B**, the 'Glen Rose Form' (MCZ 4453); **C**, *Crocodylus siamensis* (NHMUK 1921.4.1.168). Abbreviations: **ect**, ectopterygoid; **pt**, pterygoid. Scale bar = 1cm.

Choanae

187. Choanae, palatine participation: present, forms anterior margin of choanae (0); absent, choanae entirely surrounded by the pterygoids (1) (adapted from Benton and Clark, 1988; Norell and Clark, 1990 [1]; Clark, 1994 [43]; Brochu, 1997a [71]).

The degree of involvement of the palatines in the choanae has long been recognised as an evolutionarily significant morphological feature of crocodyliforms (e.g. Benton & Clark, 1988; Clark, 1994; Norell & Clark, 1990; Wu & Brinkman, 1993). Indeed, Huxley (1875) originally diagnosed Eusuchia by the enclosure of the choanae by the pterygoids (among other features). There are several existing morphological characters that describe the degree of palatine participation in the choanae, with slightly different formulations (e.g. Clark, 1994; Groh et al., 2020; Pol et al., 2009; Tennant et al., 2016). Here the character is binary, describing the presence or absence of palatine contact with the choanae, as variation in the degree of palatine participation was not observed in the taxa in the current dataset. The palatines contribute to the anterior margin of the choanae in *Bernissartia fagesii* (Norell & Clark, 1990) and some non-crocodylian eusuchians e.g. the 'Glen Rose Form' (Fig. 81A), *Theriosuchus pusillus* (Tennant et al., 2016, NHMUK 48330), and *Shamosuchus djadochtaensis* (Pol et al., 2009). By contrast, the choanae are fully enclosed by the pterygoids in allodaposuchids (e.g. *Allodaposuchus precedens* [MMS/VBN-12-10A]), hylaeochampsids (e.g. *Hylaeochampsa vectiana* [NHMUK R177]), and all crocodylians (Fig. 81C–H).

188. Choanae, position of anterior margin: anterior to posterior margin of suborbital fenestra (0); level with posterior margin of suborbital fenestra (1); posterior to posterior margin of suborbital fenestra (2) (after Clark, 1994 [44]; Pol and Norell, 2004 [44]; Pol et al., 2009 [44]) (ORDERED).

In all eusuchians with pterygoid-bound choanae, the anterior margin of the choanae lies considerably posterior to the suborbital fenestrae (188-2) (Fig. 81C–H). Taxa in which the palatine contributes to the choanae exhibit variation in choanal position relative to the suborbital fenestra. In *Bernissartia fagesii*, the anterior margin of the choana is at the level of the posterior margin of the suborbital fenestra (188-1) (Pol et al., 2009, fig.40). By contrast, the anterior margin of the choanae is positioned anterior to the posterior margin of the suborbital fenestra (188-0) in Paralligatoridae (e.g. the 'Glen Rose Form', Fig. 81A), and in *Isisfordia duncani* (Turner & Pritchard, 2015) (Fig. 81B). The character is ordered to capture the anterior to posterior transition of the choanae.

189. Choanae, position of posterior margin relative to posterior edge of pterygoid flange: anterior to or at the same level as the posterior edge of pterygoid flange (0); posterior to posterior edge of pterygoid flange (1) (after Jouve 2016 [209]; Pol et al. 2009 [44]).

This character describes the relative positions of the posterior margins of the choanae and the ptery-

goid flange, which is independent of the variation described in Character 188. The derived state in Character 189 occurs exclusively in longirostrine crocodylians, in which the fully pterygoid-bound choanae (187-1) are positioned posterior to the suborbital fenestra (188-2). In taxa with character state 189-1, the posterior margin of the pterygoid flange tends to be straight, and positioned anterior to the posterior choanal margin across its entire length, e.g. *Gavialis gangeticus* (Fig. 81E), *Thecachampsa sericodon* (USNM 24938), *Eogavialis africanum* (YPM 6263), and *Thoracosaurus isorhynchus* (MNHN 1902-22). In *Bernissartia fagesii* (IRScNB 1538) and most eusuchians (Fig. 81C, D, G), the posterior margin of the pterygoid flange is concave and its posterolateral tip extends beyond the level of the posterior choanal margin (189-0).

190. Choanae, shape: circular or elliptical (0); sub-triangular, tapering posteriorly (1); sub-rectangular (long-axis orientated mediolaterally) (2); sub-triangular, tapering anteriorly (3) (after Montefeltro et al., 2013 [22]; Jouve et al., 2015 [236]; Groh et al., 2020 [360]).

Several studies of crocodylian systematics have included a character similar to this, but it usually characterises choanal shape as either circular or triangular (Jouve et al., 2015; Iijima and Kobayashi, 2019). Character states from studies of neosuchian phylogeny have been incorporated here (e.g. Groh et al., 2020; Montefeltro et al., 2013), as they are also recognised in crocodylian taxa. In *Bernissartia fagesii* and most eusuchians the choanae are circular to elliptical (190-0) (Fig. 81C, E). Exclusively to some alligatoroids, the choanae are triangular, tapering posteriorly (190-1), e.g. *Diplocynodon ratelii* (Fig. 81F), *Caiman latirostris* (Fig. 81G), and *Paleosuchus trigonatus* (NHMUK 1868.10.8.1). By contrast, the choanae are triangular and taper anteriorly (190-3) in *Thecachampsa sericodon* (Fig. 82C), *Tomistoma lusitanica* (Antunes, 1961), and *Penghusuchus pani* (Shan et al., 2009). Rectangular choanae that are strongly mediolaterally elongate (190-2) occur in the giant caimanine taxa *Mourasuchus atopus* (Fig. 81H), *Purussaurus neivensis* (UCMP 39704), and *Purussaurus mirandai* (Aguilera et al., 2006), as well as *Gavialis lewisi* (YPM 3226).

191. Choanae, anterior margin shape: linear or curved (0); invaginated (1) (new character, based on personal observations).

In most eusuchians, the anterior margin of the choanae is straight or slightly curved, e.g. *Crocodylus porosus* (Fig. 81C) and *Gavialis gangeticus* (Fig. 81D). By contrast, some (almost exclusively alligatoroid) crocodylians exhibit a posterior midline projection of the pterygoids into the choanae (191-1), giving it a heart-shaped outline, e.g. *Caiman latirostris* (Fig. 81D), *Diplocynodon ratelii* (Fig. 81F) and *Diplocynodon hantoniensis* (NHMUK OR 25167). Several taxa that have this feature are also charactersied by a protruding choanal septum (Character state 194-1), potentially calling into question the independence of these characters. Nevertheless, some taxa that lack this protruding choanal septum (194-0), e.g. *Leidyosuchus canadensis*, do have an invaginated anterior

choanal margin (191-1) (Wu et al., 2001a, fig.2).

192. Choanae, direction of choanal projection (at maturity): posteroventrally (0); ventrally to anteroventrally (1) (after Clark, 1994 [39]; in Brochu, 1997a [72]).

As discussed by Norell (1989) and Brochu (1999, fig.43), the nasopharyngeal duct terminates in posteriorly-to-posteroventrally opening choanae in extant crocodylids (Fig. 81C), *Gavialis gangeticus* (Fig. 81E), and *Tomistoma schlegelii* (e.g. NHMUK 1894.2.21.1). This condition appears to be plesiomorphic for Crocodylia, given that it occurs in *Bernissartia* and most non-crocodylian eusuchians e.g. *Hylaeochampsa vectiana* (NHMUK R177). By contrast, the choanae of extant alligatorids face ventrally to anteroventrally, e.g. *Caiman latirostris* (Fig. 81D). Several 'basal' alligatoroids exhibit the posteroventrally-opening condition (192-0), e.g. *Diplocynodon ratelii* (Fig. 81F).

193. Choanae, septum: present (0); absent (1) (after Brochu, 1997a [152]; Groh et al., 2019 [353]).

194. Choanae, external projection of septum: absent, septum remains recessed within choanae (0); present, septum approaches external margin of choanae (1) (after Brochu, 1997a [152]).

Characters 193 and 194 were derived by reductively coding character 152 in Brochu (1997b). A choanal septum occurs in *Bernissartia fagesii* (IRScNB 1538), all alligatoroids, where preserved (e.g. *Caiman yacare* [Fig. 81D] and *Diplocynodon ratelii* [Fig. 81F], all extant crocodylids (e.g. *Crocodylus porosus* [Fig. 81C]), and most "tomistomines", e.g. *Tomistoma schlegelii* (NHMUK 1894.2.21.1) and *Thecachampsa sericodon* (USNM 24938). By contrast, most "gavialoids" lack a choanal septum; for example, it is absent in *Gavialis gangeticus* (Fig. 81E) and *Piscogavialis jugaliperforatus* (SMNK 1282 PAL), but present in *Eosuchus lerichei* (IRScNB R49). A choanal septum is also absent in several paralligatorids (e.g. the 'Glen Rose Form' [Fig. 81A]) and some allodaposuchids (e.g. *Lohuecosuchus megadontos* [Narváez et al., 2015]). Among taxa that possess a choanal septum, it is rarely so prominent that it approaches the external surface of the choanae (194-1). This condition occurs in some *Alligator* species (e.g. *A. mississippiensis*, Fig. 82D), all extant species of *Caiman* (e.g. *C. latirostris* [Fig. 82E]), and *Melanosuchus niger* (NHMUK 45.8.25.125). A prominent choanal septum was very likely present in *Diplocynodon ratelii* (194-1) but subsequently worn down (Fig. 82H).

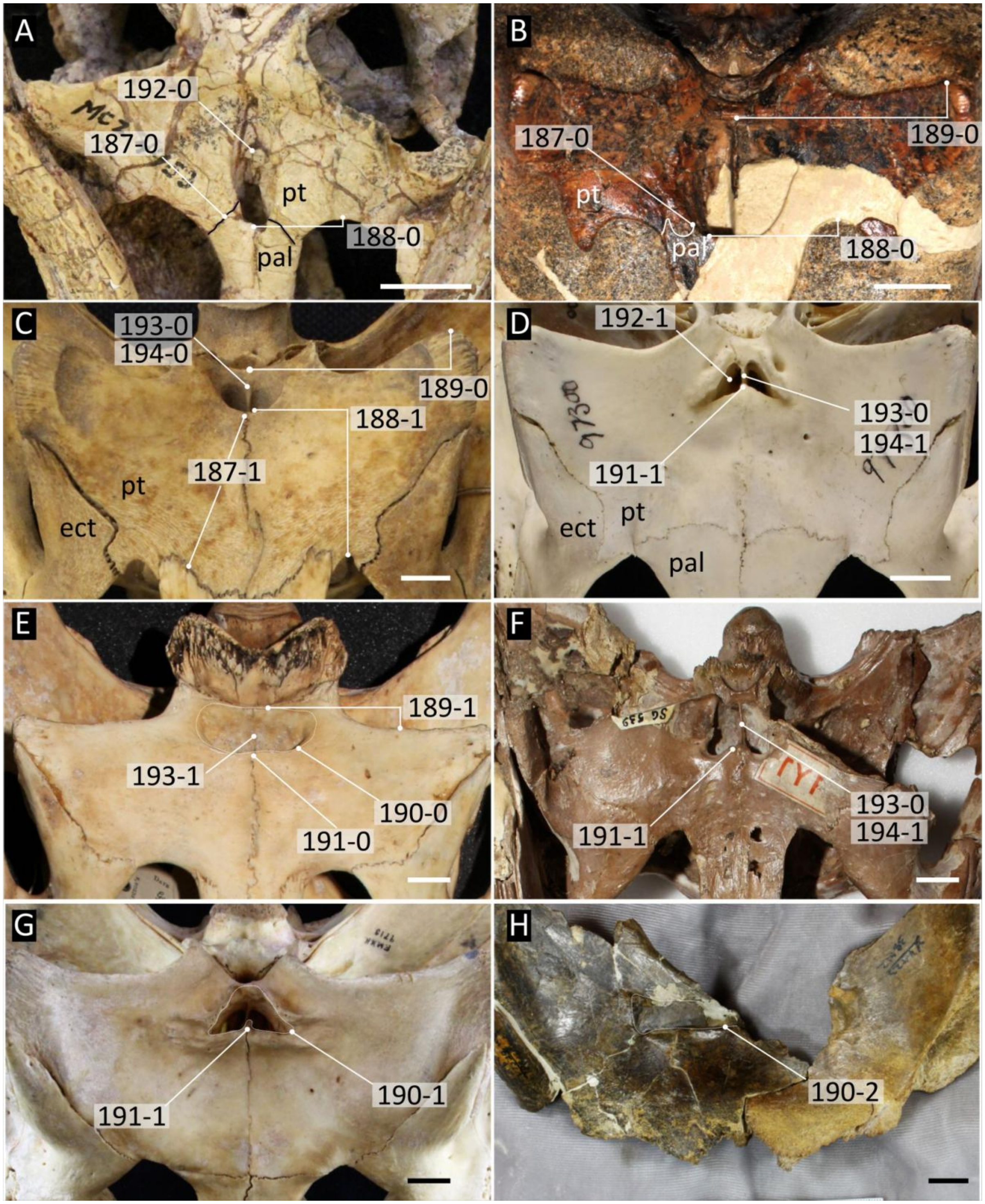

Figure 81: Position and morphology of choanae in selected crocodylian taxa. **A**, Glen Rose Form (MCZ 4453); **B**, *Isisfordia duncani* (QM F44320); **C**, *Crocodylus porosus* (QM J47448); **D**, *Caiman yacare* (AMNH 97300); **E**, *Gavialis gangeticus* (NHMUK 1935.6.4.1); **F**, *Diplocynodon ratelii* (MNHN SG 539); **G**, *Caiman latirostris* FMNH 9713; **H**, *Mourasuchus atopus* (UCMP 38012). Abbreviations: **ect**, ectopterygoid; **pal**, palatine; **pt**, pterygoid. Scale bars = 1 cm.

195. Choanae, ornamentation of margins: unornamented, margins (usually) flush with pterygoid surface (0); elevated, forming a wall restricted to the posterior and posterolateral margins (1); elevated forming a wall which extends to the anterolateral (but not anterior) margins of the choanae (2); elevated, forming a wall which completely circumscribes the choanae (3) (after Brochu, 1997a [73]; Pol and Norell, 2004 [183]).

In *Bernissartia fagesii* (IRScNB 1538) and most eusuchians, the choanal margins are unornamented (195-0) and essentially flush with the pterygoids. Some species variably develop a thin lip surrounding the choanae (Fig. 82B), or a slight thickening of the margins, which might be ontogenetic (Fig. 82C), but this is still considered unornamented. Furthermore, in taxa wherein the choanae faces posteroventrally (e.g. *Crocodylus porosus*, Fig. 82A), they cannot strictly be described as flush (hence "usually"). Nevertheless, the margins are all equally developed and considered unornamented (195-0). Character state 1 describes a condition that occurs in all extant alligatorids, in which the posterior and posterolateral margins form a ventrally projecting lamina. This was noted by Brochu (1999), although he only discretised the presence or absence of a notch in this posterior wall, a feature that occurs in caimanines (see Character 196). This posterior wall also occurs in *Brachychampsa montana* (UCMP 133901), *Stangerochampsa maccabei* (Wu et al., 1996, fig.1B), and *Eocaiman cavernensis* (AMNH 3158). Similarly upturned walls were recognised in *Diplocynodon hantoniensis* (Fig. 82G, Chapter 2), but in a slightly different arrangement. In that taxon, the lateral to anterolateral margins of the choanae are prominently upturned, but not the posterior margins (195-2). This condition is observed in all other *Diplocynodon* species, where preserved, e.g. *D. darwini* (HLMD Me 17680a), *D. deponiae* (IRScNB R 261), and (although worn) *D. ratelii* (Fig. 82H). It is also tentatively recognised in *Leidyosuchus canadensis*, based on the description and figures of Wu et al. (2001a). Character state 3 describes the choanal morphology exhibited by *Voay robusts* (Fig. 82L), in which the choanae is completely circumscribed by ventrally projecting lamina. This condition also occurs in both species of *Osteolaemus* (Fig. 82J), *Brochuchus pigotti* (Fig. 82K), and *Maomingosuchus petrolica* (Shan et al., 2017, fig.3C). Given that there is not a clear transition from the flush (195-0) to the fully-walled states, this character is not ordered.

196. Choanae, morphology of posterior wall: not notched, or with broadly rounded notch (0); acutely notched (1) (after Brochu, 1997a [107]).

This character describes the presence or absence of an acute midline incision of the posterior choanal wall described in Character state 195-1. As recognised by Brochu (1999), the acutely notched condition occurs in all extant caimanines and several fossil species (Fig. 82N). The derived condition is also recognised in *Alligator mcgrewi* (Fig. 82O). The distinction between 'broadly

rounded' (196-0) and 'acutely notched' (196-1) is important, as several alligatorids have a notch in the posterior choanal margin (e.g. *Alligator mississippiensis*, Fig. 82A), but it is notably different to the condition in caimanines (e.g. *Paleosuchus trigonatus*, Fig. 82N).

197. Pterygoid, surface lateral and anterior to choanae flush (0); depressed to form 'neck' (1) (after Brochu, 1997a [73]).

The presence of a choanal 'neck' was recovered as diagnostic of Osteolaeminae (Brochu, 2007a). This condition arises from a depression anteriorly and anterolateral to the choanae, and was indeed observed in *Osteolaemus tetraspis* (Fig. 82J), *Brochuchus pigotti* (Fig. 82K), and *Voay robustus*. A 'neck' is additionally observed in several other crocodyloids, including most extant *Crocodylus* species as well as *Gavialis lewisi* (YPM 3226).

198. Pterygoid, ornamentation lateral to choanae, anteriorly directed ridges on the pterygoid extending from the lateral margins of the choanae: absent (0); present (1) (after Lee and Yates, 2018 [161]).

Ridges on the pterygoid, lateral to the choanae, were first identified in *Kambara implexidens* (Salisbury & Willis, 1996) (Fig. 83B), and later found in several additional mekosuchines, e.g. *Kambara taraina* (Buchanan, 2009) and *Baru wickeni* (Yates, 2017). Here they are still exclusively recognised in mekosuchines. These ridges are distinguished from the upturned lateral margins of the choanae described in Character state 195-2: they are much lower and extend anteriorly on the pterygoid beyond the choanae.

Pterygoid

199. Pterygoid, bulbous differentiated bullae (at maturity): absent (0); present (1) (after Lee and Yates, 2018 [158]; Salas-Gismondi et al., 2019 [206]).

The pterygoids and palatines forming the walls of the nasopharyngeal duct can become inflated through ontogeny in several species of *Crocodylus*, e.g. *C. porosus* (Fig. 84A). However, this condition is distinguished from the condition exhibited by all species of *Gavialis* examined here (e.g. *G. gangeticus*, Fig. 84B), which develop bulbous, differentiated 'bullae' that are formed entirely by the pterygoids at maturity (199-1).

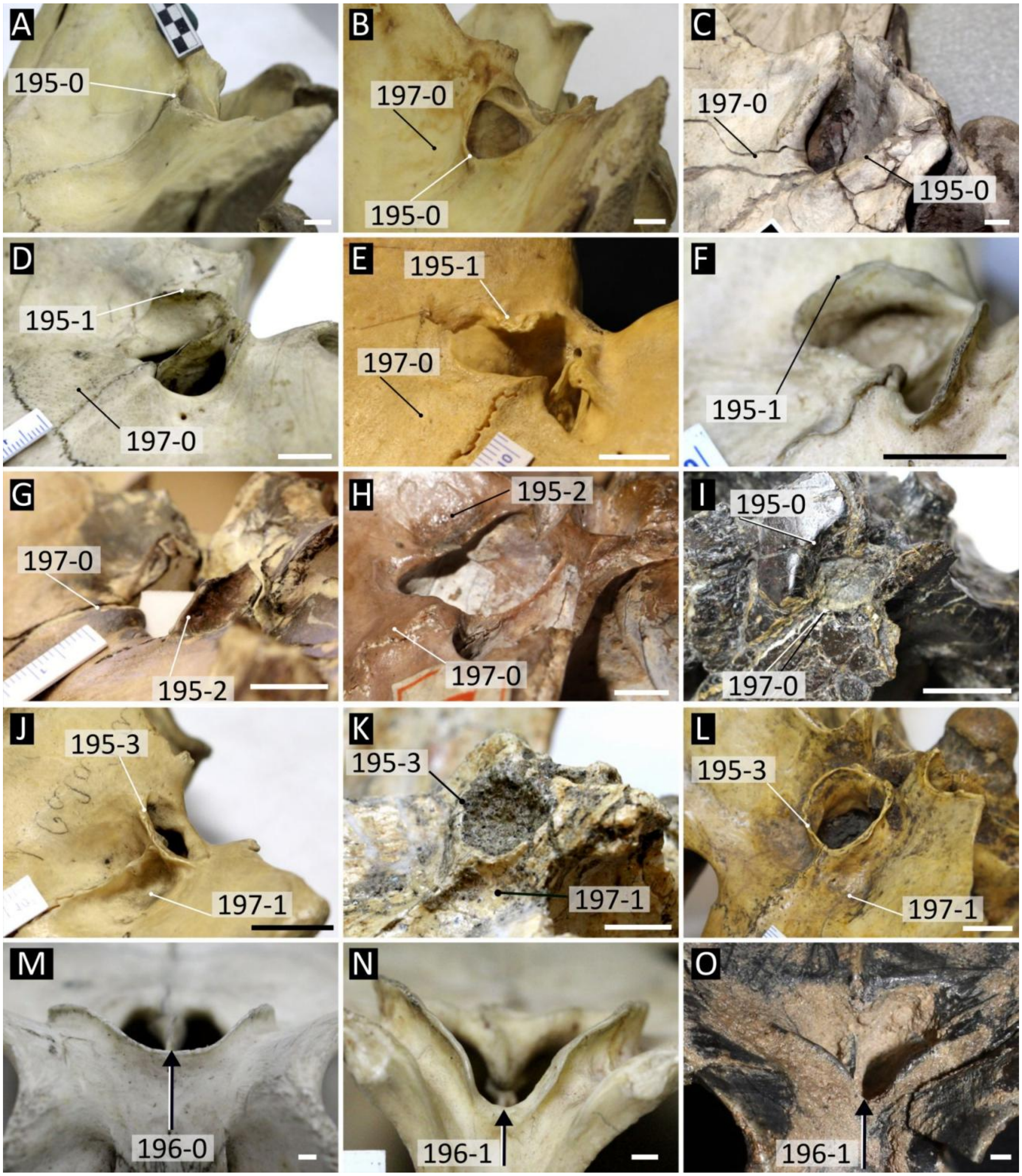

Figure 82: Morphology of the choanal rim in selected crocodylians. **A**, *Crocodylus porosus* (NHMUK 1852.12.9.2); **B**, *Mecistops cataphractus* (NHMUK 1924.5.10.1); **C**, *Thecachampsa sericodon* (USNM); **D**, *Alligator mississippiensis* (NHMUK 1873.2.21.1); **E**, *Caiman yacare* (MACN uncatalogued specimen); **F**, *Paleosuchus trigonatus* (NHMUK 1868.10.8.1); **G**, *Diplocynodon hantoniensis* (CAMSM TN 907) ; **H**, *Diplocynodon ratelii* (MNHN SG 539); **I**, *Hylaeochampsa vectiana* (NHMUK R 177); **J**, *Osteolaemus tetraspis* (NHMUK 1862.6.30.5); **K**, *Brochuchus pigotti* (NHMUK R 7729); **L**, *Voay robustus* (NHMUK R 36685); **A**, *Alligator mississippiensis* (NHMUK 1873.2.21.1); **B**, *Paleosuchus trigonatus* (NHMUK 1868.10.8.1); **D**, *Alligator mcgrewi* (AMNH FAM 7905). Scale bars A–L = 1 cm, M–O = 2 mm.

200. Quadrate, ventral surface, attachment scar for posterior mandibular adductor muscle, morphology: linear crests (0); ventrally directed knob (1) (after Brochu, 2011 [180]; Ösi et al., 2007 [165]).

The ventral surface of the quadrate is ornamented with several low, linear crests (200-0) that form the attachment sites for the mandibular adductor muscles in *Bernissartia fagesii* and most eusuchians (Fig. 85A) (Iordansky, 1973). By contrast, in *Hylaeochampsa vectiana* (Fig. 85B), and *Iharkutosuchus makadii* (Ösi, 2008), the ventral surface of the quadrate bears a discrete knob-like protuberance (200-1).

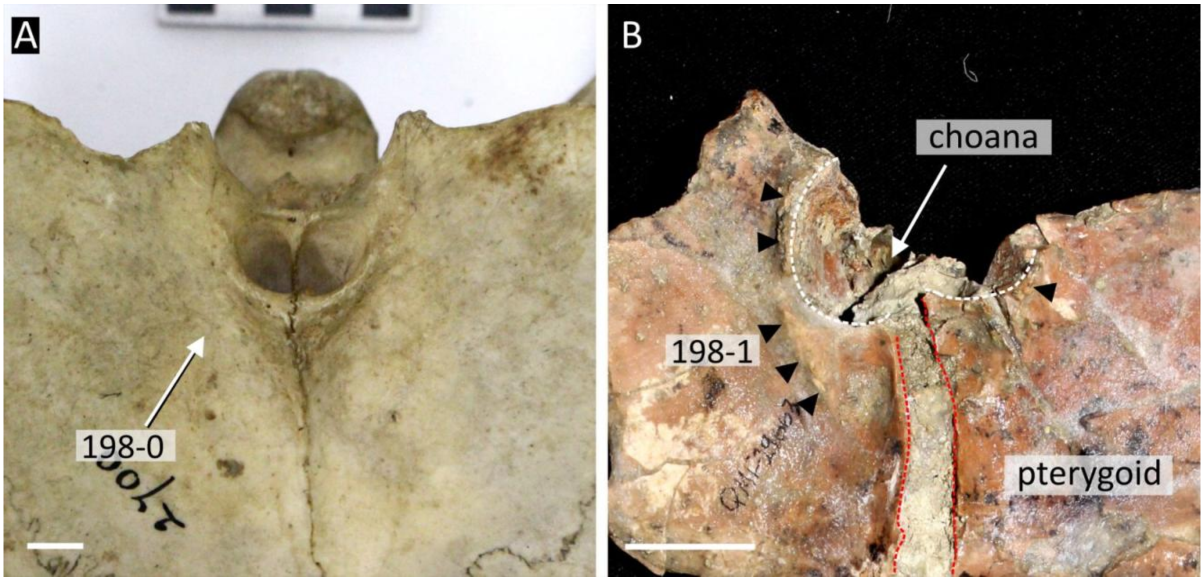

Figure 83: Ventral view of the choanae showing variation in development of ridges anterolateral to the choanae in: **A**, *Crocodylus siamensis* (NHMUK 1921.4.1.168); **B**, *Kambara implexidens* (QM 29663). Black arrows mark position of ridge. All scale bars = 1 cm.

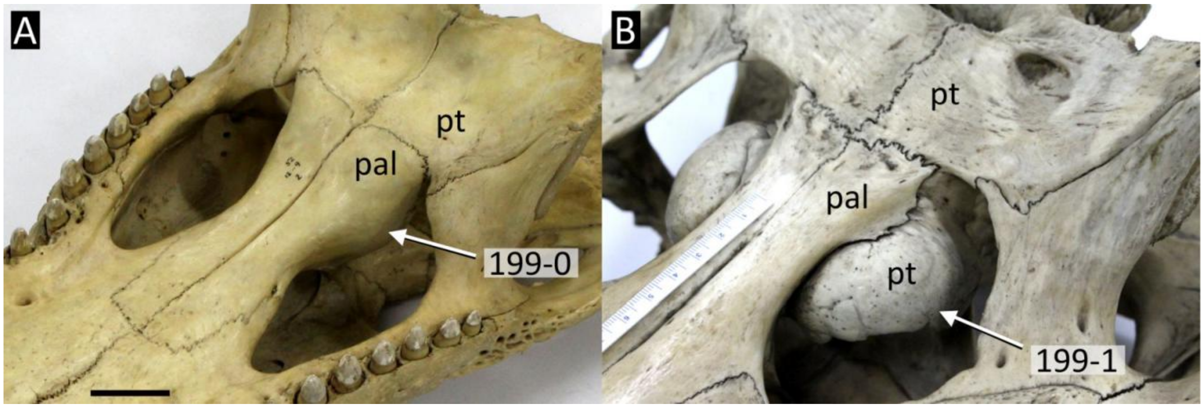

Figure 84: Ventrolateral view of the palatine bar showing the development of pterygoid bullae. **A**, *Crocodylus porosus* (NHMUK 1852.12.9.2); **B**, *Gavialis gangeticus* (NHMUK 1974.3009). Abbreviations: **pal**, palatine; **pt**, pterygoid. Scale bar in A = 3cm, B = cm.

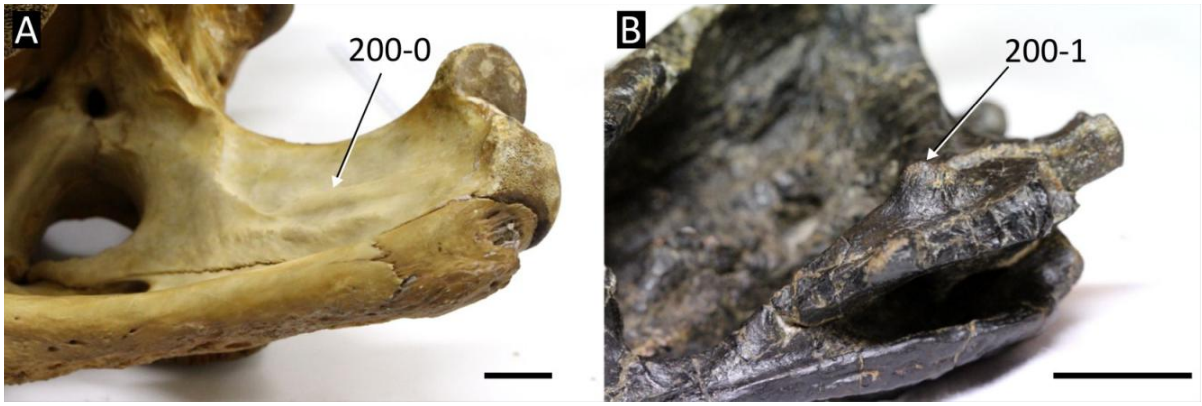

Figure 85: Ventromedial view of the quadrate ramus in **A**, *Tomistoma schlegelii* (NHMUK 1894.2.21.1); and **B**, *Hylaeochampsa vectiana* (NHMUK PV R 177). All scale bars = 2 cm.

201. Basisphenoid, exposure between basioccipital and pterygoids in ventral view: not or poorly exposed, basisphenoid anteroposteriorly short (0); largely exposed, basisphenoid anteroposteriorly long (1) (after Brochu, 1997a [113]; Jouve, 2016 [113]).

The derived character state describes the anteroposteriorly long basisphenoid exposure that is unique to *Gavialis gangeticus* (Fig. 86C) among extant crocodylians (Fig. 86A–B) (Brochu, 2006b). The derived condition also occurs in most fossil "gavialoids", e.g. *Eogavialis africanum* (YPM 6263) and *Eosuchus minor* (Brochu, 2006b).

202. Jugal, posterior extent relative to basioccipital tubera: extends beyond level of posterior margin of basioccipital tubera (0); level with or anterior to posterior margin of basioccipital tubera (1) (after Jouve, 2004 [186]; Jouve et al., 2008 [181]; Jouve, 2016 [181]).

In most eusuchians, the jugal extends beyond the level of the posterior margin of the basioccipital tubera (Fig. 86A–B). By contrast, in some "gavialoids", e.g. *Gavialis gangeticus* (Fig. 86C) and *Argochampsa krebsi* (NHMUK R36872), the jugals do not extend beyond this margin, usually terminating anterior to the level of the basioccipital tubera. This feature is best observed in ventral view.

Braincase

203. Basisphenoid rostrum, posteroventrally directed ridge on lateral margins: absent (0); present (1) (new character, based on personal observations).

Few taxa preserve the basisphenoid rostrum sufficiently to score this character; however, in all extant alligatorids examined here, an arcuate ridge occurs on the posterolateral surface of the basisphenoid rostrum, e.g. *Alligator mississippiensis* (Fig. 87A). This ridge is absent in *Gavi-*

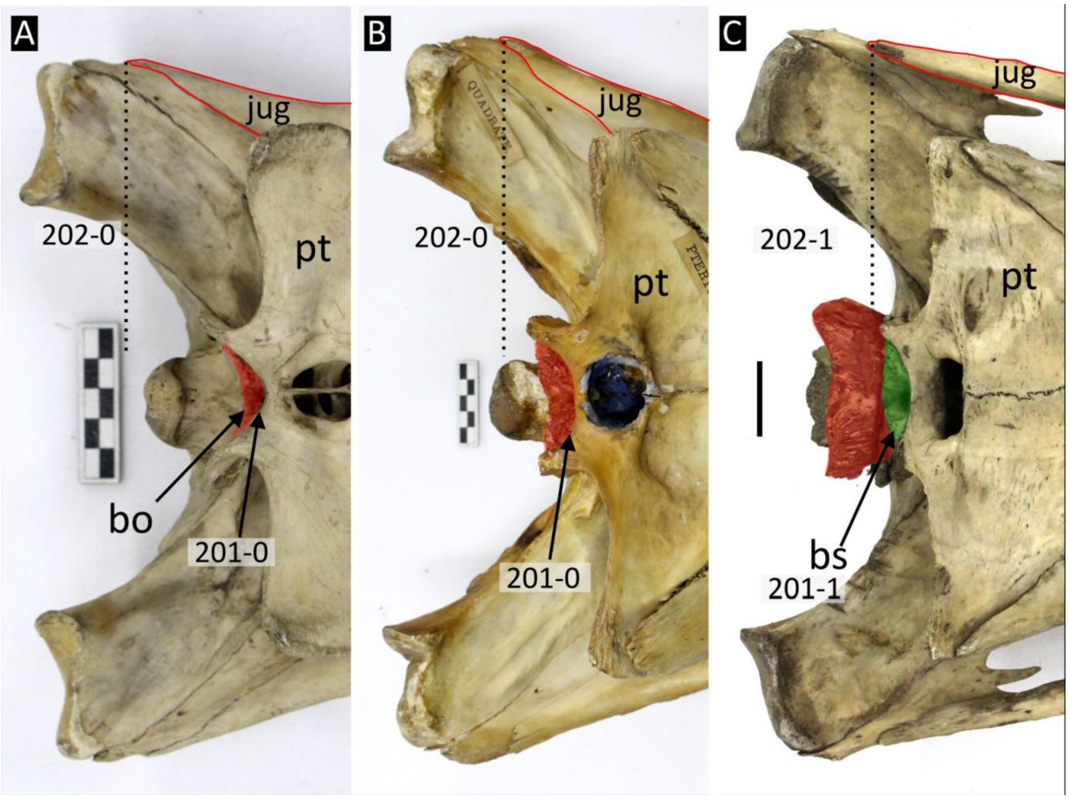

Figure 86: Variation in the posterior extent of the jugal relative to the basioccipital tubera. **A**, *Alligator mississippiensis* (NHMUK 1873.2.21.1); **B**, *Tomistoma schlegelii* (NHMUK 1894.2.21.1); **C**, *Gavialis gangeticus* (NHMUK 1974.3009). Basioccipital tubera highlighted in red, dotted line marks posterior extent of jugal. Abbreviations: **bo**, basioccipital; **bs**, basisphenoid; **jug**, jugal; **pt**, pterygoid. Scale bar = 5 cm.

alis gangeticus (NHMUK 96.7.7.4.2), *Tomistoma schlegelii* (NHMUK 1894.2.21.1) and all extant *Crocodylus* species, e.g. *C. siamensis* (Fig. 87B).

204. Basisphenoid, exposure on the lateral braincase wall, anteroventral to the trigeminal foramen: absent (0); present (1) (after Brochu, 1997a [129]).

The derived character state describes a posterior extension of the basisphenoid onto the lateral braincase wall that typically reaches the level of the laterosphenoid bridge in all extant crocodylids, e.g. *Crocodylus* (Fig. 87B), *Osteolaemus tetraspis* (NHMUK 1862.6.30.5), and *Mecistops cataphractus* (NHMUK 1924.5.10.1), as well as *Tomistoma schlegelii* (Fig. 87F). By contrast, the basisphenoid is not visible on the lateral braincase wall, or extends posteriorly by only a small amount, in *Gavialis gangeticus* (NHMUK 1974.3009) and all extant alligatorids, e.g. *Alligator mississippiensis* (Fig. 87A), *Caiman crocodilus* (Fig. 87E), and *Melanosuchus niger* (Fig. 87C).

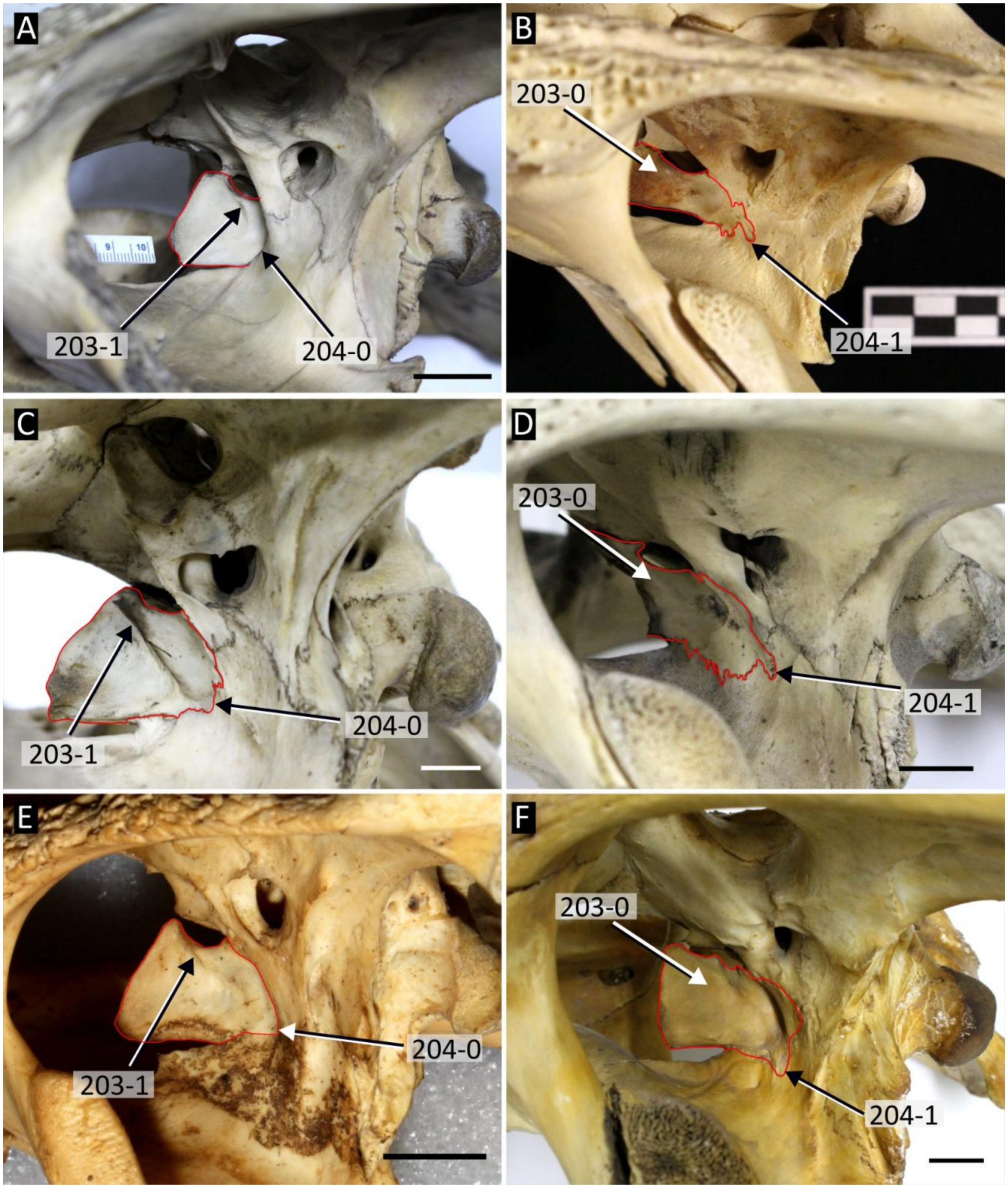

Figure 87: Variation in morphology of the basisphenoid rostrum. Left lateral view of the braincase in **A**, *Alligator mississippiensis* (NHMUK 1873.2.21.1); **B**, *Crocodylus rhombifer* (AMNH 77595); **C**, *Melanosuchus niger* (NHMUK 45.8.25.125); **D**, *Crocodylus acutus* (NHMUK 1975.997); **E**, *Caiman crocodilus* (FMNH 69812); **F**, *Tomistoma schlegelii* (NHMUK 1894.2.21.1). Basisphenoid outlined in red. All scale bars = 2 cm.

205. Basisphenoid, sulcus on anterior braincase wall, lateral to basisphenoid rostrum: present (0); absent (1) (after Brochu, 1997a [122]).

In anteromedial view of the braincase, a sulcus can be observed on the posterolateral surface of the basisphenoid in some crocodylians (205-1), e.g. *Gavialis gangeticus* (Fig. 88A). This condition is recognised here in almost all extant crocodylians, except for some species of *Crocodylus* (*C. intermedius*, *C. johnstoni*, *C. mindorensis*, and *C. novaeguineae*); this contrasts with previous datasets, which scored all extant crocodylids and *Tomistoma schlegelii* as lacking this fossa. These differences may be a result of interspecific variation in the degree of development of the fossa, which could lead some authors to score the condition as absent when it is here considered 'weakly' developed.

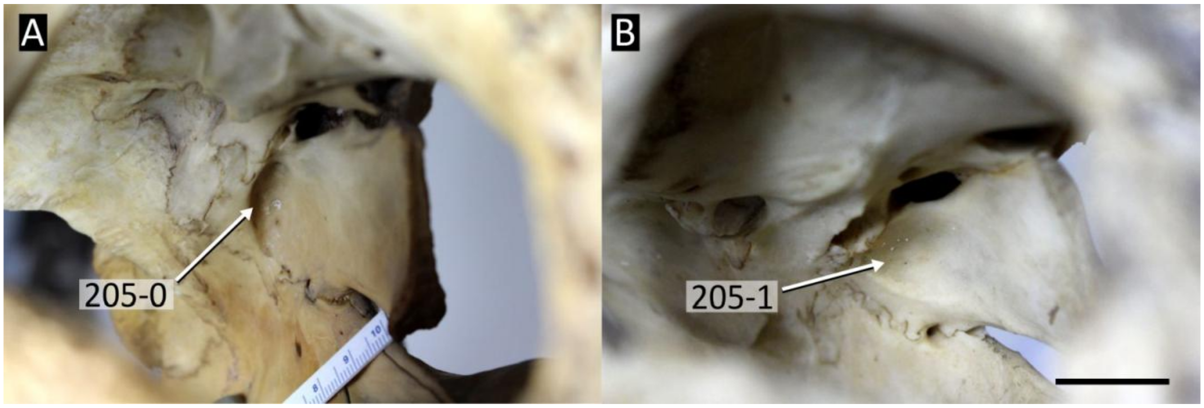

Figure 88: Anteromedial view of the basisphenoid rostrum in **A**, *Gavialis gangeticus* (NHMUK 1935.6.4.1); and **B**, *Crocodylus niloticus* (NHMUK 1934.6.3.1). Scale bar = 2 cm.

206. Laterosphenoid, orientation of capitate process anterior margin: perpendicular to the sagittal plane (0); directed anterolaterally from the sagittal plane (1) (after Brochu, 1997a [130]).

The capitate process is a dorsolateral extension of the laterosphenoid, that contacts the ventral margins of the postorbital and frontal at the anterolateral corner of the cranial table (Fig. 89). In most crocodylians, the anterior margin of the capitate process is orientated anterolaterally relative to the sagittal plane (Fig. 89A). By contrast, the anterior margin is orientated almost perpendicular to the sagittal plane in some, longirostrine crocodylians, e.g. *Gavialis gangeticus* (Fig. 89B), *Eogavialis africanum* (NHMUK R 3325), *Piscogavialis jugaliperforatus* (SMNK 1282 PAL), *Thecachampsa sericodon* (USNM 24938), and *Thoracosaurus neocesariensis* (AMNH 2542).

207. Laterosphenoid, lateral laterosphenoid bridge over cavum epiptericum: absent (0); present (1) (after Lee and Yates, 2018 [122]; Brochu, 1999; Holliday and Witmer, 2009).

208. Laterosphenoid, lateral laterosphenoid bridge morphology: short process, which does not suture to

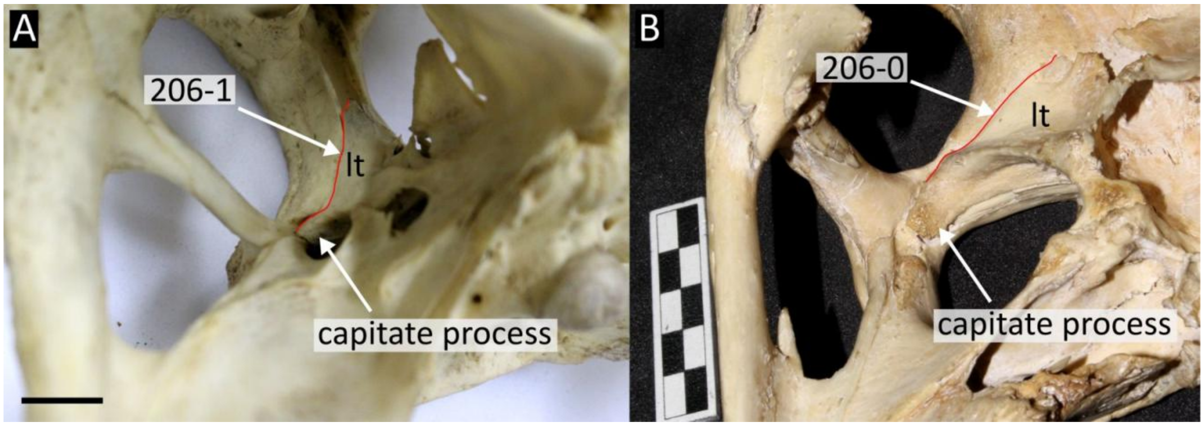

Figure 89: Ventromedial view of the braincase in **A**, *Caiman latirostris* (NHMUK 86.10.4.2); **B**, *Gavialis gangeticus* (NHMUK uncatalogued). Abbreviations: **lt**, laterosphenoid. Scale bar in A = 2 cm, B = cm.

the pterygoid ventrally (0); robust process, which sutures to the pterygoid ventrally (1) (adapted from Holliday and Witmer, 2009; Lee and Yates, 2018 [122]).

The lateral laterosphenoid bridge is a dorsoventrally orientated strut of bone formed predominantly by a descending process of the laterosphenoid anterior to the foramen ovale (Fig. 90). This bridge encloses the ophthalmic branch of cranial nerve V (Brochu, 1999; Holliday et al., 2013; Iordansky, 1973). Where preserved, all extant and most fossil crocodylians possess a lateral laterosphenoid bridge (207-1) (Fig. 90B–F). By contrast, the bridge is absent (207-0) in *Hylaeochampsa vectiana* (NHMUK R177), *Portugalosuchus azenhae* (Mateus et al., 2019, fig.9B), *Shamosuchus djadochtaensis* (Pol et al., 2009) and several "gavialoids" including *Piscogavialis jugaliperforatus* (Fig. 90A), *Eogavialis africanum* (NHMUK R3325), and *Gryposuchus colombianus* (UCMP 38358) (Holliday & Witmer, 2009, fig.12A). Brochu and Gingerich (2000) commented that the laterosphenoid bridge of most "crocodylids" is incomplete, such that it does not contact the pterygoid to fully enclose the ophthalmic branch of CN V. By contrast, the bridge was found to contact the pterygoid in all extant "crocodylids" examined here (208-1), with the exception of *Tomistoma schlegelii*, in which it forms a small discontinuous process (Fig. 90B) (208-0), similar to that described in the "tomistomines" *Paratomistoma courti* (Brochu & Gingerich, 2000).

209. Laterosphenoid, caudal laterosphenoid bridge over cavum epiptericum: absent (0); present (1) (after Lee and Yates, 2018 [124]; adapted from Holliday and Witmer, 2009).

210. Laterosphenoid, caudal laterosphenoid bridge morphology: short ventrally directed strut (0); long ventral process joining with extra process of the quadrate (1); hypertrophied wall, which bisects the foramen ovale (2) (after Lee and Yates, 2018 [124]; adapted from Holliday and Witmer, 2009) (ORDERED).

Characters 209 and 210 were derived by reductively coding Character 124 from Lee and Yates

(2018), and by the addition of a character state. The caudal (=posterior) bridge of the laterosphenoid (sensu Holliday & Witmer, 2009) is a ventral process positioned at the dorsal margin of the foramen ovale, at the level of the laterosphenoid-quadrate suture (Fig. 90). This bridge is absent in most fossil crocodylians in which the braincase is preserved (e.g. *Piscogavialis jugaliperforatus*, Fig. 90A), but occurs in some form in most extant crocodylians. For example, the bridge forms a short, discontinuous strut (210-0) in *Tomistoma schlegelii* (Fig. 90B) and several species of *Crocodylus* (e.g. *Crocodylus novaeguineae*, Fig. 90D). As noted by (Holliday & Witmer, 2009), the bridge is elongate in some extant *Crocodylus* species, with extra bony processes that form a continuous bridge e.g. *Crocodylus siamensis* (Fig. 90E). The new character state (210-2) included here is based on observations of *Mourasuchus arendsi* (Cidade et al., 2019b) (Fig. 90F), and describes a single robust caudal bridge, which is not known in any other eusuchian studied here. The character is also ordered. (Fig. 91)

211. Laterosphenoid, ascending process of the pterygoid forming ventral portion of lateral laterosphenoid bridge: absent (0); present (1) (after Brochu, 1997a [115]).

Most crocodylian character lists describe an ascending process of the palatine that contributes to the base of the laterosphenoid bridge, which appears to be an error carried forward from the character list of Brochu (1997a). As described and clearly figured by Brochu (1999, fig.52C), the derived character state refers in fact to an ascending pterygoid process, which forms a robust ventral portion of the lateral laterosphenoid bridge in some crocodylians, e.g. *Alligator mississippiensis* (Fig. 91B) and *Diplocynodon ratelii* (Fig. 91A). By contrast, the lateral bridge receives little to no contribution from the pterygoid in most crocodylians, where known (Fig. 91C–F).

212. Epipterygoid: present (0); absent (1) (after Lee and Yates, 2018 [121]; adapted from Holliday and Witmer, 2009).

213. Epipterygoid, retraction from the cavum epitericum: epipterygoid overhangs cavum epitericum (0); or epipterygoid isolated from cavum epitericum (1) (after Lee and Yates, 2018 [121]; adapted from Holliday and Witmer, 2009).

Characters 212 and 213 were derived by reductively coding Character 121 in Lee and Yates (2018). The epipterygoid links the palate with the braincase plesiomorphically in Crocodylomorpha, forming the lateral wall of the cavum epitericum (Holliday & Witmer, 2009). The epipterygoid appears to be absent in nearly all crocodylians (212-0), where the laterosphenoid bridge forms an analogous structure; however, Holliday and Witmer (2009) identified the epipterygoid in *Leidyosuchus canadensis*, *Eosuchus minor*, and *Borealosuchus sternbergii* (212-1). The morphology of the epipterygoid is variable. For example, in *Eosuchus minor* the epiterygoid is small and triangular, and does not overhang the cavum epitericum (213-1) (Holliday & Witmer, 2009, fig.11H).

By contrast, the epiterygoid is larger and more ventrally developed in *Leidyosuchus* and *Borealosuchus sternbergii*, such that it overhangs the cavum epitericum (213-0) (Holliday & Witmer, 2009, fig.11D).

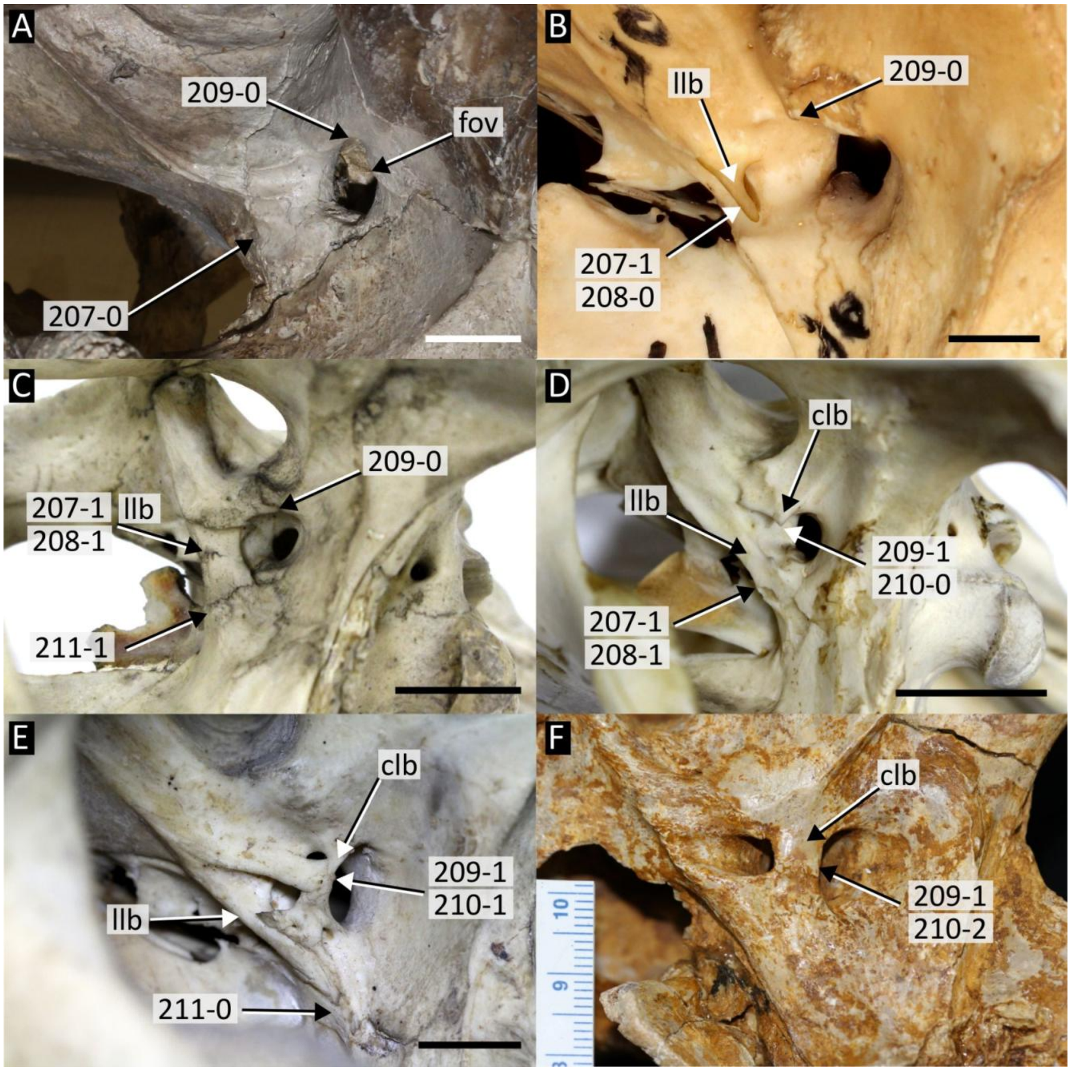

Figure 90: Left lateral view of the braincase showing variation in morphology of the laterosphenoid in **A**, *Piscogavialis jugaliperforatus* (SMNK 1282 PAL); **B**, *Tomistoma schlegelii* (USNM 211323); **C**, *Alligator mississippiensis* (NHMUK 1873.2.21.1); **D**, *Crocodylus novaeguineae* (NHMUK 1886.5.20.1); **E**, *Crocodylus siamensis* (NHMUK 1897.12.31.1); **F**, *Mourasuchus arendsi* (UFAC 2515). Abbreviations: **clb**, caudal laterosphenoid bridge; **fov**, foramen ovale; **llb**, lateral laterosphenoid bridge. All scale bars = 2 cm.

214. Prootic, exposure on external braincase wall: small, little to no exposure dorsal and ventral to the trigeminal foramen (0); large exposure ventral to trigeminal foramen only (1); extensive exposure dorsal and ventral to trigeminal foramen (2) (after Norell, 1989 [5]; Brochu, 1997a [74]) (ORDERED).

The prootic is a poorly exposed bone that is partially visible in the walls of the foramen ovale

in most eusuchians (Fig. 91). As originally formulated, this character was binary, distinguishing between a small or extensive exposure of the prootic. Here, an intermediate state is added and the character is ordered. In previous datasets (e.g. Brochu et al., 2012), the extensive prootic exposure was recognised in *Gavialis gangeticus*, "tomistomines" (e.g. *Tomistoma schlegelli*, *Paratomistoma courti*, *Thecachamapsa antiquus*), some *Borealosuchus* (e.g. *B. sternbergii*, and *Diplocynodon* species (e.g. *D*textitratelii and *D. hantoniensis*). Here, the extensive exposure (214-2) is only considered present in *Gavialis gangeticus* and *Tomistoma schlegelii*. The condition in these two taxa is remarkably similar, with prominent, anteroposteriorly narrow dorsal and ventral extension of the prootic on the braincase wall (Fig. 91C, E). By contrast, *Diplocynodon ratelii* has a modest ventral exposure of the prootic, but it is hidden dorsally in the walls of the foramen ovale (Fig. 91A). This condition is similar to that of a number of additional crocodylians, including *Borealosuchus sternbergii* (USNM 6533), *Paratomistoma courti* (Brochu & Gingerich, 2000, fig.3D), *Alligator mississippiensis* (Fig. 91B) and some *Crocodylus* species (Fig. 91D). These taxa have been assigned to a new character state (214-1), which is considered intermediate between the very small prootic exposure of most crocodylians (214-0) (Fig. 91F), and the extensive exposure of *Gavialis gangeticus* nad *Tomistoma schlegelii*.

215. Quadrate-pterygoid suture, path on lateral braincase wall between basisphenoid exposure and foramen ovale: ventrally deflected (0); straight (1) (after Brochu, 1997a [127]).

In extant alligatorids, the quadrate-pterygoid suture runs diagonally along the lateral braincase wall, from the basisphenoid exposure to the foramen ovale, with a prominent ventral deflection (215-0), e.g. *Alligator mississippiensis* (Fig. 92A). The same condition occurs in *Gavialis gangeticus* (NHMUK 1935.6.4.1), *Tomistoma schlegelii* (USNM 211323), and several fossil crocodylians, including *Brachychampsa montana* (UCMP 133901) and *Gryposuchus colombianus* (UCMP 38358). By contrast, the quadrate-pterygoid suture is approximately straight in *Diplocynodon* (e.g. *D. ratelii*, Fig. 92B), and all extant crocodylids. The condition is unknown in the outgroup *Bernissartia fagesii*, and the only non-crocodylian eusuchian scored for this character (*Iharkutosuchus makadii*) exhibits the ventrally deflected condition (215-0) (Mateus et al., 2019, fig.S13).

Mandible

Dentary-Splenial

216. Dentary, anteriormost teeth: strongly procumbent, appraoching sub-horizontal (0); project dorsally or steeply anterodorsally (1) (after Brochu, 1997a [53]).

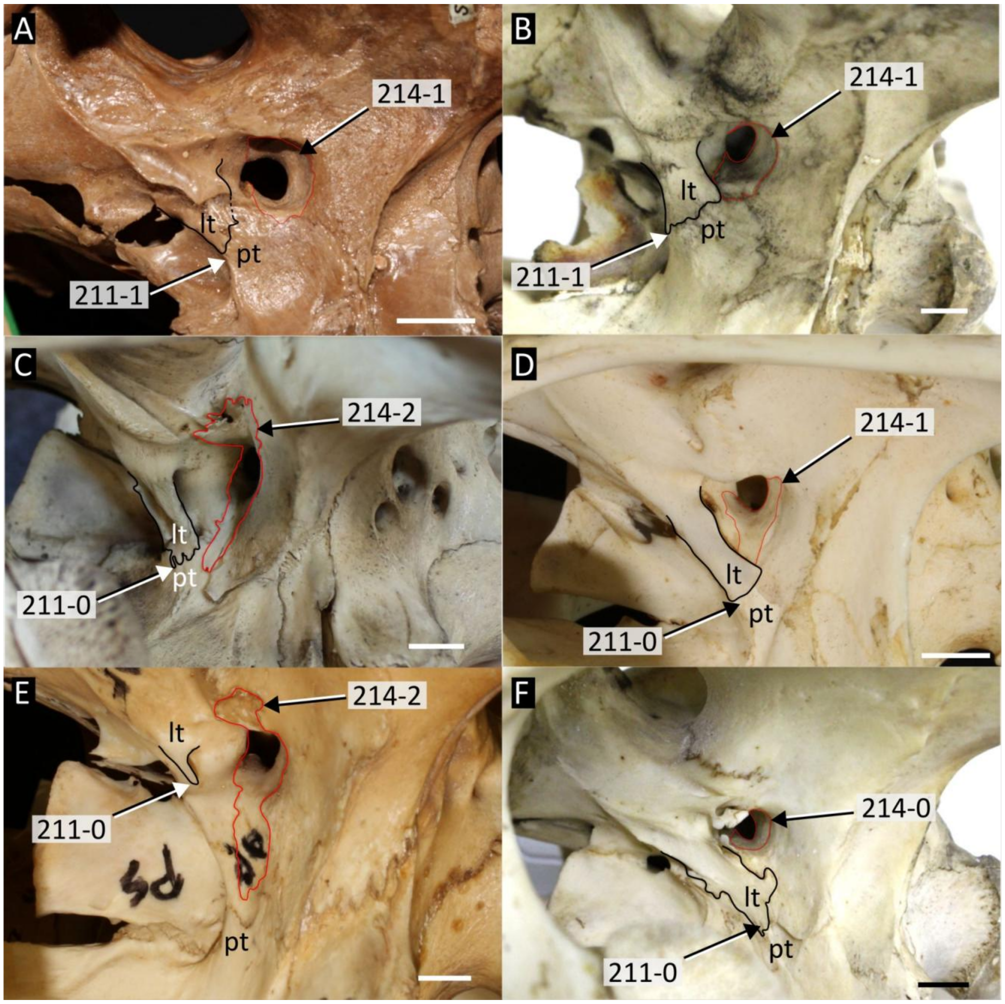

Figure 91: Left lateral view of the braincase showing variation in prootic exposure (red outline). **A**, *Diplocynodon ratelii* (MNHN SG 557); **B**, *Alligator mississippiensis* (NHMUK 1873.2.21.1); **C**, *Gavialis gangeticus* (NHMUK uncatalogued); **D**, *Crocodylus intermedius* (FMNH 75659); **E**, *Tomistoma schlegelii* (USNM 211323); **F**, *Crocodylus niloticus* (NHMUK 1934.6.3.1). Abbreviations: **lt**, laterosphenoid, **pt**, pterygoid. All scale bars = 1 cm.

Strongly procumbent anterior dentary teeth occur in the outgroup *Bernissartia fagesii* (IRScNB 1538) and some paralligatorids, e.g. the 'Glen Rose Form' (Fig. 93A) and *Wannchampsus kirkpachi* (Adams, 2014). Among crocodylians, procumbent dentary teeth also characterise *Arambourgia gaudryi* (MNHN QU17155), and *Mekosuchus*, e.g. *M. inexpectatus* (MNHN NCP 06). All other eusuchians exhibit dorsally or steeply anterodorsally-projecting anterior dentary teeth (Fig. 93B).

217. Dentary, alveoli 3 and 4: confluent (0); separate (1) (after Brochu, 1997a [52]).

Most eusuchians have an enlarged 4th dentary caniniform tooth. Equal enlargement of the 3rd and 4th dentary alveoli, such that they are weakly separated and share the same interalveolar wall, occurs in *Bernissartia fagesii* (IRScNB 1538), *Diplocynodon* (e.g. *D. ratelii*, Fig. 93C), *Leidyosuchus*

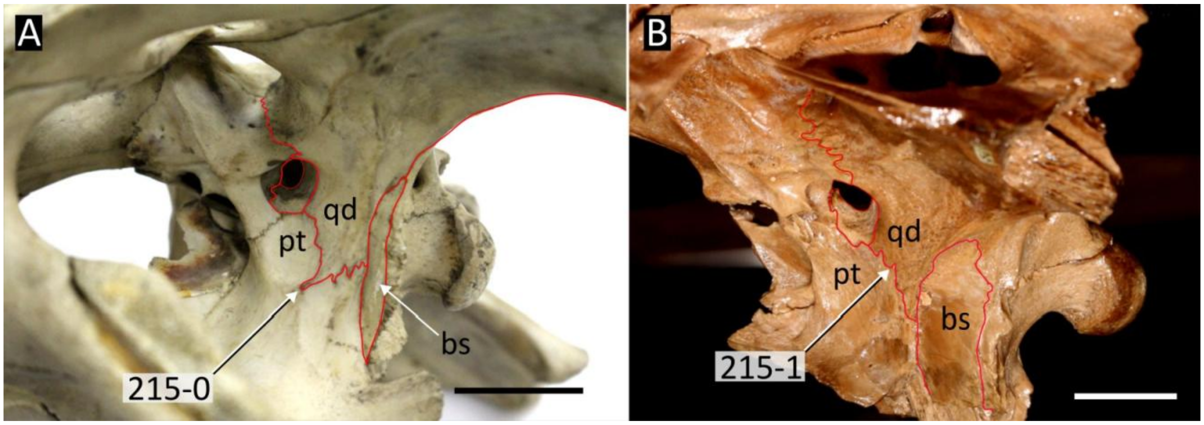

Figure 92: Lateral view of the braincase showing variation in the quadrate-pterygoid suture. **A**, *Alligator mississippiensis* (NHMUK 1873.2.21.1); and **B**, *Diplocynodon ratelii* (MNHN SG 557). Abbreviations: **bs**, basisphenoid; **pt**, pterygoid; **qd**, quadrate. All scale bars = 2 cm.

canadensis (YPM 284), *Borealosuchus* (e.g. *B. sternbergii*, USNM 6533), and *Eothoracosaurus mississippiensis* (Brochu, 2004a). In all other crocodylians, the 3rd and 4th dentary alveoli are separated (Fig. 93D), with the 4th alveolus being notably larger than the third.

218. Dentary, dorsoventral height at the level of alveoli 1–4 relative to alveoli 11–12: at the same level or higher (0); lower (1) (adapted from Bona, 2007; Pinheiro et al., 2013 [124]; Cidade et al., 2017 [183]).

In most crocodylians, the dorsal margin of the alveolar walls of dentary alveoli 1–4 are approximately in line with alveoli 11–12 (Fig. 93E). Bona (2007) recognised an alternative condition in *Eocaiman*, in which the anteriormost dentary alveoli are more ventrally positioned than the posterior dentary alveoli (Fig. 93F). Whereas this condition has only been recognised in *Eocaiman* in previous studies (e.g. Cidade et al., 2017). It also occurs in several non-caimanine alligatoroids, e.g. *Alligator mcgrewi* (AMNH FAM 8700), *Navajosuchus mooki* (AMNH 6780), and *Allognathosuchus wartheni* (YPM PU 16989).

219. Dentary, numerical position of largest alveolus posterior to 4th dentary alveolus: 13 and/or 14 (0); 13 and/or 14 and a posterior series (1); 10, 11 and/or 12 (2); no differentiation posterior to 4th alveolus (3); posterior to 14 (4) (after Brochu, 2004a [167]; Brochu, 2010 [37]; Brochu, 2011 [51]).

Most crocodylians have two areas of enlarged alveoli in the dentary (Brochu, 2004b). Whereas the first consistently occurs at the 4th alveolus, the second is variable and can extend over a series of alveoli. In most eusuchians the second region of enlargement occurs between alveoli 10–12 (219-2), e.g. all extant caimanines (Fig. 94C), extant crocodylids, *Diplocynodon*, and *Borealosuchus*. In many alligatorines (including *Alligator*), this enlargement occurs at the level of alveoli 13 and

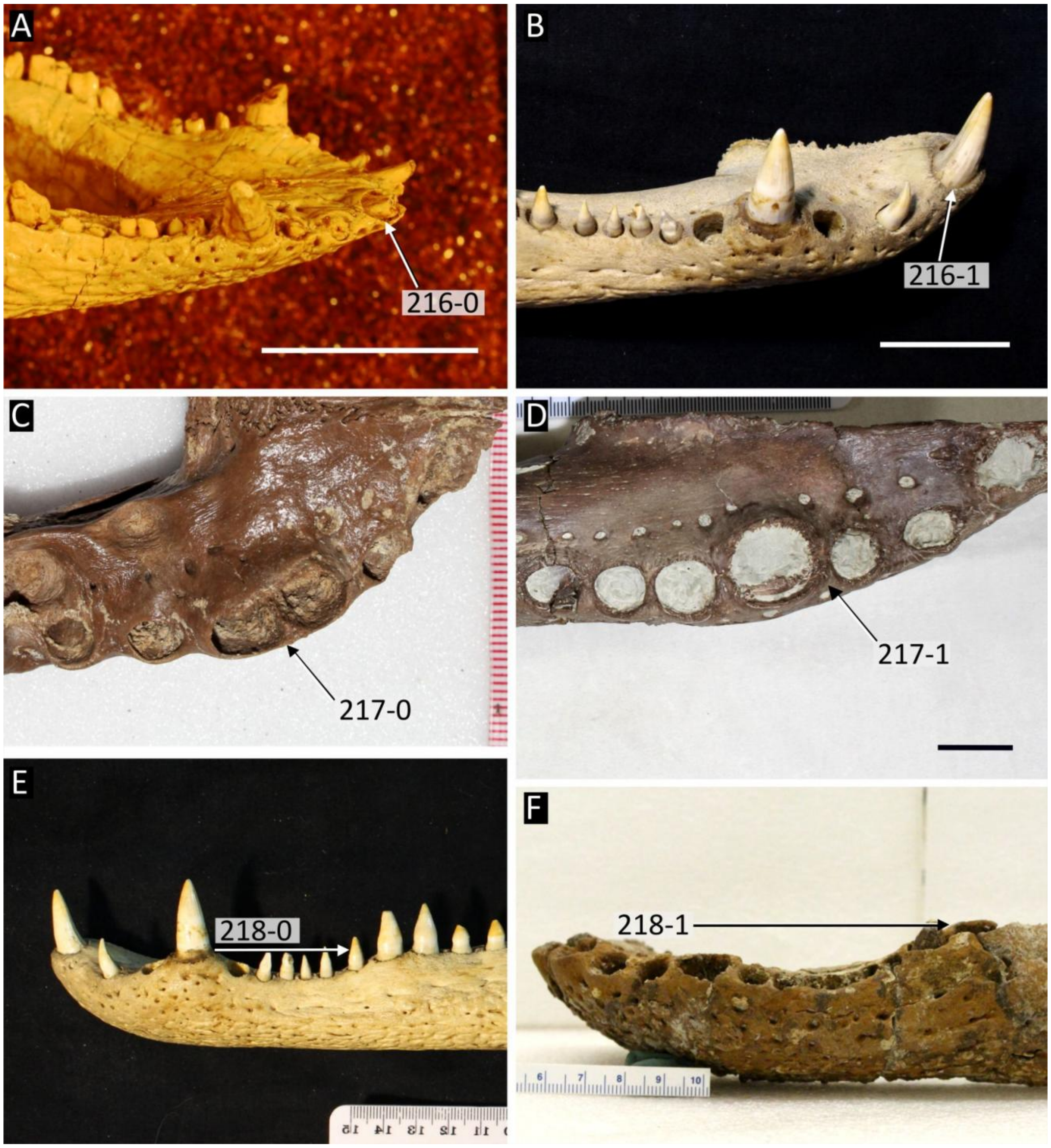

Figure 93: Morphology of the anterior dentary toothrow. **A**, Glen Rose Form (USNM 22039); **B**, *Caiman yacare* (MACN uncatalogued); **C**, *Diplocyndon ratelii* (MNHN G 660); **D**, '*Crocodylus*' *affinis* (UCMP 154341); **E**, *Caiman yacare* (MACN uncatalogued); **F**, *Eocaiman palaeocenicus* (MPEF PV 1933a). Scale bars in A, B, and D = 2 cm; C = mm; E, F = cm.

14 (219-0) (Fig. 94A), but other alligatorines show enlargement of the 13th, 14th, and a posterior series of dentary alveoli (219-1) e.g. *Allognathosuchus wartheni* (Fig. 94B). Several longirostrine crocodylians, e.g. *Gavialis gangeticus* (Fig. 94D) and *Mourasuchus atopus* (UCMP 38012), have homodont dentition. In the case of *Gavialis*, enlargement of the 4th dentary alveolus is not apparent; however, it is dorsally raised compared to all other dentary alveoli (219-3) (Fig. 95A). Character state 4 describes the enlargement of alveoli posterior to the 14th alveolus (Fig. 94E), which occurs in some non-crocodylian eusuchians, e.g. *Iharkutosuchus makadii* (Mateus et al., 2019, fig.S11).

220. Dentary, shape of dorsal profile between 4th and 10th alveoli in lateral view: linear (0); curved (1); deeply curved (2) (after Brochu, 1997a [68]) (ORDERED).

The dentary is broadly curved between alveoli 4 and 10 in *Bernissartia fagesii* (IRScNB 1538) and most eusuchians (Fig. 95B). As noted by Brochu (1999), the depth of this curvature is notably greater in some *Alligator* species (e.g. *A. mcgrewi*, AMNH FAM 8700), as well as some putative alligatorines, e.g. *Allognathosuchus* (Fig. 95C), *Hassicaosuchus haupti* (HLMD Me 4415), and *Navajosuchus mooki* (AMNH 6780). By contrast, the dentary is completely linear in this region in most longirostrine crocodylians, e.g. *Gavialis gangeticus* (Fig. 95A).

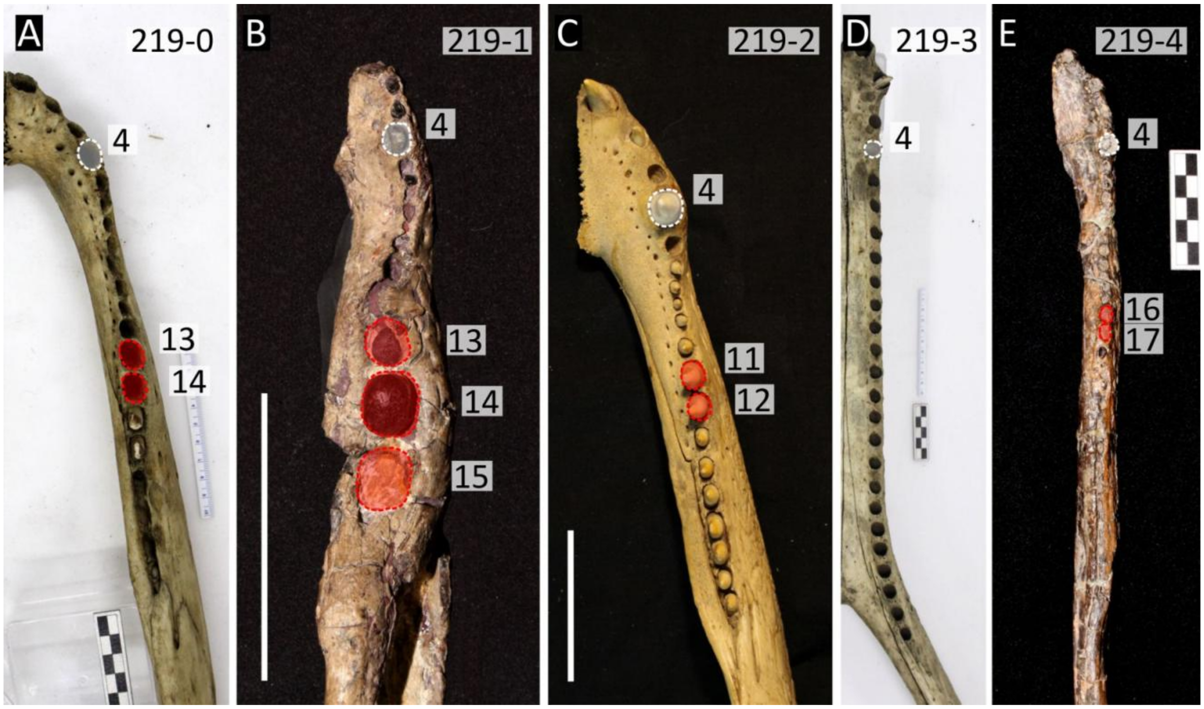

Figure 94: Dorsal view of the right mandibular ramus showing variation in alveolar size in: **A**, *Alligator mississippiensis* (NHMUK 68.2.12.6); **B**, *Allognathosuchus wartheni* (YPM PU 16989); **C**, *Caiman yacare* (MACN uncatalogued); **D**, *Gavialis gangeticus* (NHMUK 1974.3009); **E**, *Listrognathosuchus multidentatus* (AMNH 5179). Largest alveoli posterior to the 4th are shaded in red. Scale bars in B and C = 5 cm, all other scale bars = cm.

221. Mandibular symphysis, posterior extent, adjacent to number of full dentary alveoli: <6 (0); 6–8 (1); 9–12 (2): 13–20 (3); >20 (4) (after Jouve, 2004 [180]; Brochu, 2004a [166]; Salas-Gismondi et al., 2016 [49]) (ORDERED).

This character refers to the full length of the mandibular symphysis, i.e. formed by the dentary and splenial (where present) (Fig. 96). In *Bernissartia fagesii* (IRScNB 1538) and most eusuchians, the dentary symphysis only reaches the level of the 4th dentary tooth (221-0) (Fig. 96A). Most taxa exhibit intraspecific variation by one alveolus at most. For example, in *Alligator mississippiensis* the symphysis might reach five alveoli lengths (Brochu, 2004b), but never six. The plesiomorphic

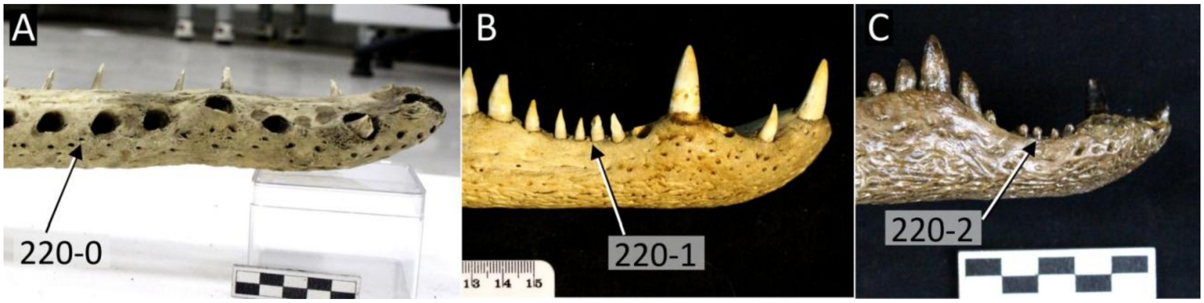

Figure 95: Variation in degree of curvature of the dentary toothrow between alveoli 4 and 10 in selected crocodylians. **A**, *Gavialis gangeticus* (NHMUK 1974.3009); **B**, *Caiman yacare* (MACN uncatalogued); **C**, *Allognathosuchus* sp. (USNM 25807). All scale bars = cm.

character state also occurs in most extant *Crocodylus* species and *Diplocynodon*. The symphysis is adjacent to 6–8 alveoli (221-1) in several alligatorines (e.g. *Navajosuchus mooki* [AMNH 6780] and *Allognathosuchus wartheni* [YPM PU 16989]), 'basal' crocodyloids (e.g. '*Crocodylus*' *affinis*, UCMP 154341), and some extant longirostrine crocodylids, e.g. *Crocodylus johnstoni* (QM J45309) and *Mecistops cataphractus* (Fig. 96B). Fewer taxa are scored for the remaining character states, which are mostly present in longirostrine crocodylians. Whereas the mandibular symphysis reaches 9–12 alveoli (221-2) in some "tomistomines" (e.g. *Thecachampsa antiquus* [Fig. 96C] and *Maroccosuchus zennaroi* [MNHN APH 18]), it is longer (221-3) in all *Gryposuchus* species (e.g. *G. colombianus* [Fig. 96D]), and longer still (221-4) in *Gavialis gangeticus* (Fig. 96E) and *Piscogavialis jugaliperforatus* (SMNK 1282 PAL). Given this continuous spectrum of values, this character is ordered.

222. Splenial, participation in symphysis: full participation, dorsal and ventral to Meckelian fossa (0); partial participation by splenial rostral tip (1); no participation (2) (after Clark, 1994 [77]; Brochu, 1997a [43]; Jouve, 2016 [43]) (ORDERED).

223. Splenial, position of anteriormost tip relative to Meckelian fossa: ventral (0); dorsal (1) (after Clark, 1994 [77]; Brochu, 1997a [43])

224. Splenial, anterior extent in dentary symphysis: adjacent to 1 full alveolus (0); 2 to 3 alveoli (1); 4 to 7 alveoli (2); more than 7 alveoli (3) (after Clark, 1994 [77]; Jouve, 2016 [43]) (ORDERED).

225. Splenial, shape of splenial-dentary suture adjacent to dentary toothrow (in dorsal view): constricted, laterally concave (narrow 'V'-shape) (0); straight (wide 'V'-shape) (1) (after Brochu, 1997a [43]).

Characters 222–225 were derived by reductively coding Character 43 from Jouve (2016), which was adapted from Brochu (1997b, Character 43). As originally formulated, the character combined descriptions of the splenial length and morphology. For example: "… *deep splenial symphysis,*

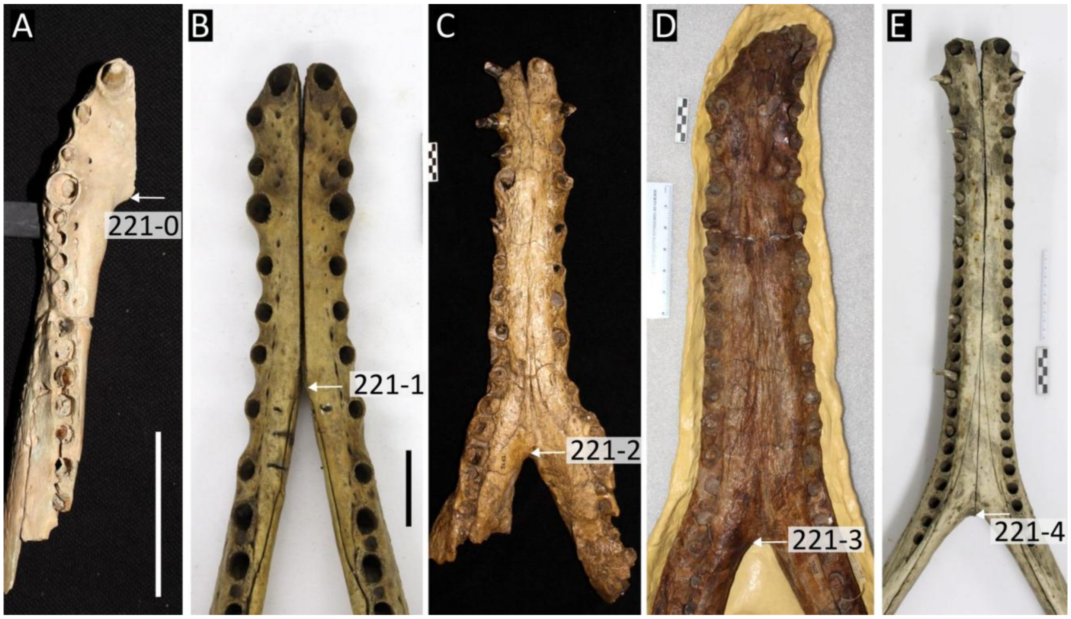

Figure 96: Dorsal view of the dentary symphysis in **A**, *Australosuchus clarkae* (QM F18151); **B**, *Mecistops cataphractus* (NHMUK 1865.4.6.1); **C**, *Thecachampsa antiquus* (AMNH 5662); **D**, *Gryposuchus colombianus* (UCMP 40062); **E**, *Gavialis gangeticus* (NHMUK 1974.3009). Scale bars in A and B = 5 cm, all other scale bars = cm.

participates in the mandibular symphysis over the length of five to seven teeth, and forms wide "V" within symphysis (4); or deep splenial symphysis participates in the mandibular symphysis over the length of five to seven teeth, and splenial constricted within symphysis and forms narrow "V" (5) . . ." (Jouve, 2016). As noted by Harshman et al. (2003), this precludes the grouping of taxa with an elongate symphysis, such as *Tomistoma schlegelii* and *Gavialis gangeticus*. Similarly, character states 1 and 2 in the same character preclude the grouping of taxa that lack a splenial symphysis, based on differences in splenial morphology: "*. . . splenial excluded from mandibular symphysis and anterior tip of splenial passes ventral to Meckelian groove (1); splenial excluded from mandibular symphysis and anterior tip of splenial passes dorsal to Meckelian groove (2) . . .*". Character 222 describes the presence (Fig. 97A–B) or absence (Fig. 97C–D) of a splenial symphysis, but is augmented by an intermediate character state, describing a ventral contribution to the symphysis observed in '*Crocodylus*' *affinis* (Fig. 97E) and *Asiatosuchus depressifrons* (Fig. 97F). This is considered distinct from the condition of some caimanines, in which the splenial approaches the symphysis but does not participate in ti (Fig. 97G–H) (222-2). Character 223 describes the morphology of the anterior splenial tip in taxa that lack a splenial symphysis. As recognised by Brochu (1999), the anterior splenial tip is positioned dorsal to the Meckelian fossa in all extant alligatorids (e.g. *Melanosuchus niger*, Fig. 97D), but ventral in some 'basal' alligatoroids (e.g. *Diplocynodon*) and all extant crocodylids, e.g. *Crocodylus moreletii* (Fig. 97C). Taxa with a splenial symphysis must be scored as a "?". Where present, the splenial symphysis length

is variable. For example, it is adjacent to only one alveolus (224-0) in *Asiatosuchus germanicus* (Fig. 98A), and *Boverisuchus vorax* (UCMP 170767). The splenial symphysis reaches 2–3 alveoli (224-1) in *Borealosuchus sternbergii* (Fig. 98B), *Borealosuchus formidabilis* (YPM PU 16241), and *Maroccosuchus zennaroi* (Jouve et al., 2015, fig.4B). In most longirostrine crocodylians, the symphysis is adjacent to 4–7 alveoli (224-2), e.g. *Thecachampsa* (Fig. 98C), *Eosuchus* (Fig. 98D), and *Tomistoma schlegelii* (Fig. 98E). Some "gavialoids" exhibit a highly elongated splenial symphysis that extends beyond seven alveoli (224-3), e.g. *Gavialis gangeticus* (Fig. 98F), *Eogavialis africanum* (YPM 6263), and *Ikanogavialis gameroi* (Sill, 1970). Character 225 describes the morphology of the splenial symphysis strictly in taxa with a long splenial symphysis, i.e. taxa scored for character state 224-2 or 224-3. The constricted condition has traditionally been recognised only in "tomistomines", e.g. *Thecachampsa* (Fig. 98C) and *Tomistoma schlegelii* (Fig. 98E). This contrasts with the unconstricted splenials of most "gavialoids", e.g. *Eosuchus* (Fig. 98D) and *Gavialis gangeticus* (Fig. 98F), but not *Gryposuchus colombianus* (UCMP 40293), which exhibits the constricted condition (Fig. 98G).

226. Dentary symphysis, shape of posterior margin of symphyseal surface in medial view: dorsal lobe extends further posterior than ventral lobe (0); dorsal and ventral lobes subequal in extent, or ventral lobe projects further posterior than dorsal lobe (1) (after Lee and Yates, 2018 [176]).

In taxa that lack a splenial symphysis, the posterior margin of the symphyseal surface of the dentary exhibits a dorsal and ventral lobe that are separated by the Meckelian fossa. In taxa with a full splenial symphysis (222-0), these lobes are poorly delimited, such that this character is considered inapplicable (Fig. 99C). In extant species of *Alligator*, *Crocodylus*, *Caiman*, and *Melanosuchus* the ventral lobe is anteriorly recessed such that the dorsal lobe clearly extends further posteriorly (Fig. 99A). By contrast, the lobes are subequally developed in some mekosuchines (e.g. *Baru wickeni* [Fig. 99B] and *Australosuchus clarkae* [QM F18151]), *Asiatosuchus depressifrons* (IRScNB R253), and some *Diplocynodon* species, e.g. *D. hantoniensis* (NHMUK OR 30394).

227. Dentary, orientation of posteriormost alveoli: in a straight line (0); in a laterally curved line (1) (new character, based on personal observations).

In *Bernissartia fagesii* (IRScNB 1538) and most eusuchians, the posteriormost dentary alveoli are arranged in a straight line, e.g. *Alligator mississippiensis* (Fig. 100A). By contrast, the posteriormost dentary alveoli of some crocodylians are arranged in a laterally curved line. This latter condition is most prominent in the putative basal alligatorines, *Allognathosuchus* (Fig. 100F) and *Navajosuchus mooki* (Fig. 100E). Indeed, in *Allognathosuchus*, the whole posterior ramus of the mandible appears to be laterally deflected (Fig. 100F). A posteriorly curved toothrow also occurs in some species of *Alligator*, e.g. *A. mcgrewi* (Fig. 100D) and *A. prenasalis* (Fig. 100C), but not

A. mefferdi (Fig. 100B).

228. Dentary, posterior process between angular and splenial on ventral side of the mandible: absent (0); present (1) (after Jouve, 2004 [187]; in Jouve, 2016 [182]).

A posterior process of the dentary between the angular and splenial, on the ventral side of the mandible was scored in *Gavialis gangeticus* by Jouve (2016) (Fig. 101B). A comparable process is present in some fossil specimens (e.g. NHMUK R36727) of this species (Martin, 2019), as well as in *Gavialis lewisi* (YPM 3226). Given its apparent absence in all other taxa considered, it might be diagnostic of *Gavialis*. *Gavialis browni* (AMNH 6279) does not preserve a mandible (Mook, 1932) and, although the mandible is preserved in *Gaviali benjawanicus* (not studied here), the presence or absence of this feature was not described, nor can it clearly be ascertained from the figures (Martin et al., 2012).

229. Splenial, anterior perforation for mandibular ramus of cranial nerve V (i.e. foramen intermandibularis oralis): present (0); absent (1) (after Norell, 1988 [15]; Norell, 1989 [8]; Brochu, 1997a [41]).

The mandibular branch of cranial nerve V exists the splenial anteriorly through the opening of the Meckelian fossa in crocodylians (Schumacher, 1973, fig.30). In some crocodylians, cranial nerve V also exits through the anteriorly positioned foramen intermandibularis oralis (229-0) (Norell, 1989) (Fig. 102B). Among extant crocodylians, this foramen only occurs in *Alligator sinensis* (Brochu, 1999) and *Gavialis gangeticus*, where it is obscured from view by the mandibular symphysis (Norell, 1989, fig.5). Among fossil taxa, the foramen is present in *Bernissartia fagesii* (IRScNB 1538), *Borealosuchus*, alligatorines (e.g. *Allognathosuchus wartheni*, YPM PU 16989), and all species of *Alligator*, except *A. mississippiensis*.

230. Splenial, posterior perforation(s) for mandibular ramus of cranial nerve V: absent (0); present (1) (after Norell, 1988 [15]; Norell, 1989 [8]; Brochu, 1997a [42]).

231. Splenial, number of posterior perforations for mandibular ramus of cranial nerve V: one (0); two (1) (after Norell, 1988 [15]; Norell 1989 [8]; Brochu, 1997a [42]).

Characters 230 and 231 were derived by reductively coding character 42 in Brochu (1997a). Cranial nerve V always exits the splenial anteriorly through the Meckelian fossa and/or the foramen intermandibularis oralis. Some taxa also have a posterior perforation (Fig. 102A), or two posterior perforations (Fig. 102D). The latter condition has traditionally only been recognised in *Paleosuchus* (e.g. Brochu, 1999), but some *Caiman* species (e.g. *Caiman yacare*, Fig. 102D) are polymorphic in terms of the number of posterior perforations.

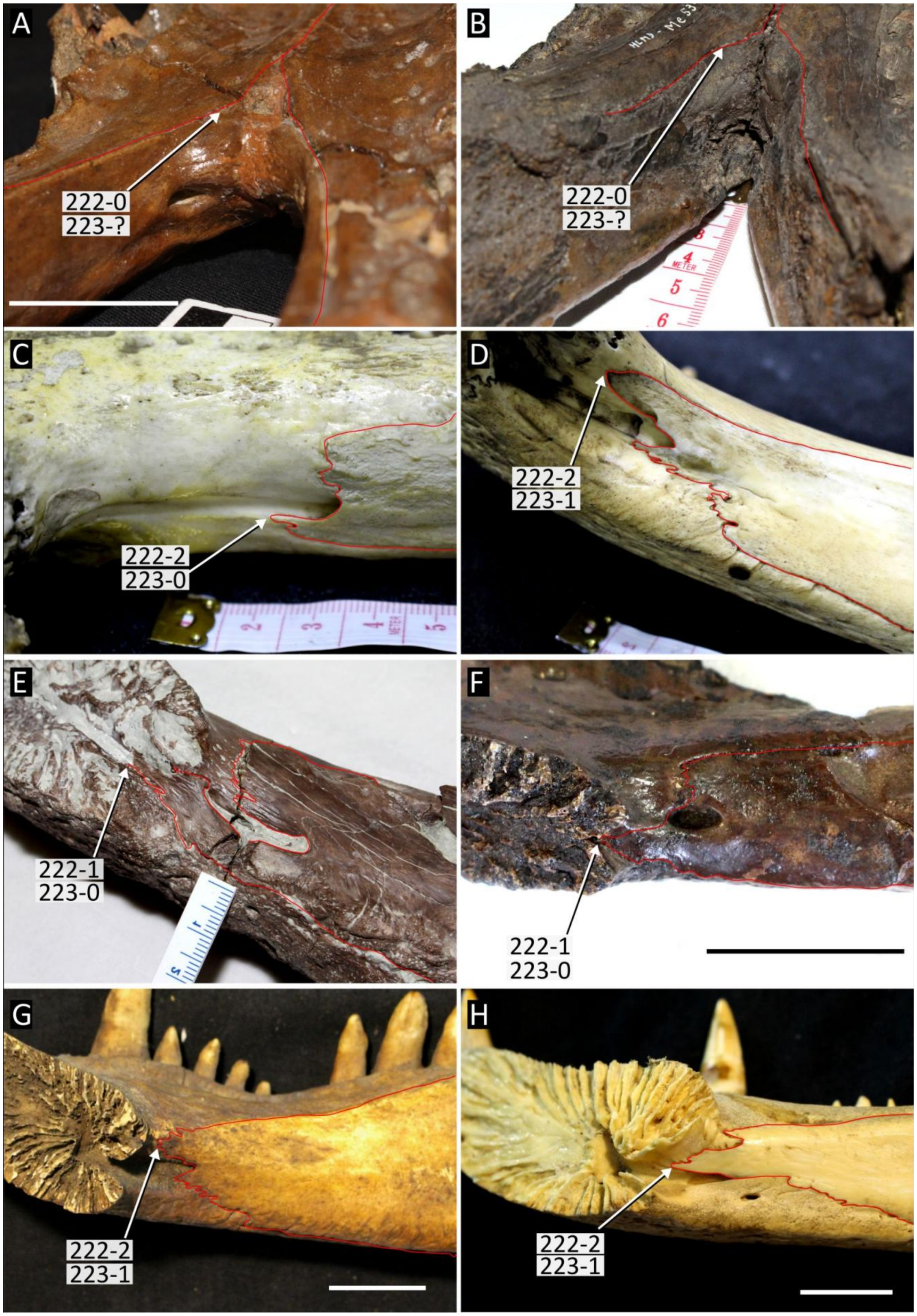

Figure 97: Medial view of the dentary symphysis in selected crocodylians showing variation in participation of the splenial (outlined in red). **A**, *Crocodylus moreletti* (NHMUK); **B**, *Melanosuchus niger* (NHMUK 45.8.25.125); **C**, *'Crocodylus' affinis* (UCMP 154341); **D**, *Asiatosuchus depressifrons* (IRSNB R 253); **E**, *Caiman latirostris* (MACN V 1420); **F**, *Caiman yacare* (MACN uncatalogued). Scale bars in A, F–H = 2 cm, all other scale bars = cm.

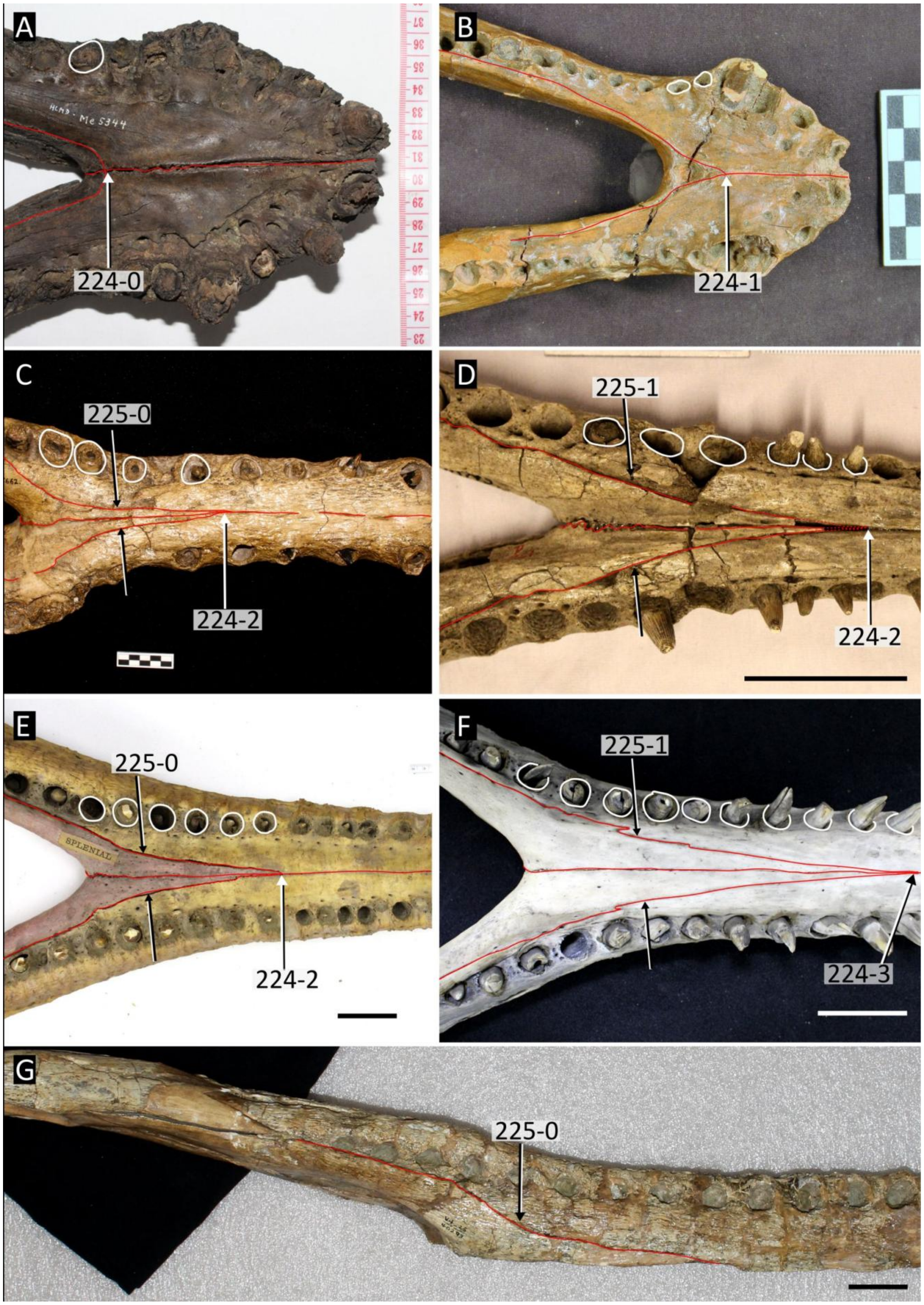

Figure 98: Dorsal view of the mandible showing variation in the splenial symphysis (outlined in red). **A**, *Asiatosuchus germanicus* (HLMD Me 5344); **B**, *Borealosuchus sternbergii* (USNM V 6533); **C**, *Thecachampsa antiquus* (AMNH 5662); **D**, *Eosuchus lerichei* (IRSNB R 49); **E**, *Tomistoma schlegelii* (NHMUK 1894.2.21.1); **F**, *Gavialis gangeticus* (NHMUK uncatalogued); **G**, *Gryposuchus colombianus* (UCMP 40293). All scale bars = 5 cm.

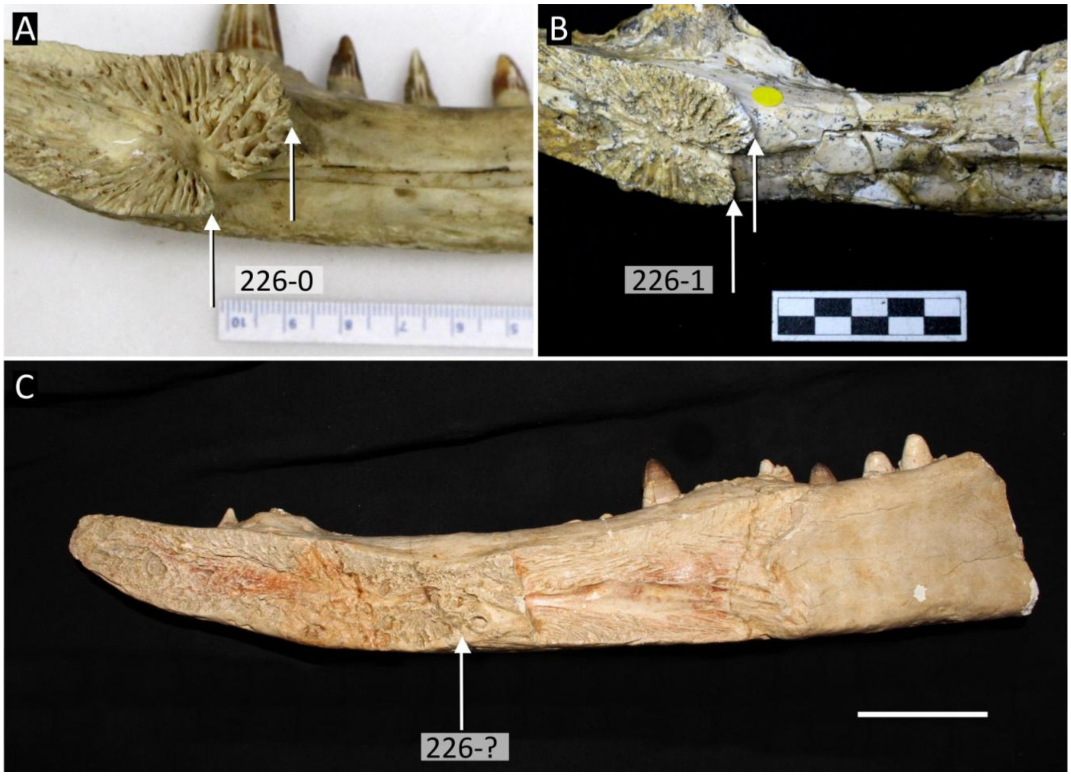

Figure 99: Medial view of the dentary symphysis in: **A**, *Crocodylus siamensis* (NHMUK 1921.4.1.171); **B**, *Baru wickeni* (QM 31070); **C**, *Maroccosuchus zennaroi* (MNHN APH 18). Scale bar in C = 5 cm, all other scale bars = cm.

232. Splenial, shape of dorsal profile: straight (anterodorsally inclined) (0); concave (abruptly dorsally inclined at posterior end) (1) (after Lee and Yates, 2018 [191]).

This character was adapted from Lee and Yates (2018); however, different taxa are scored for the derived character state here, suggesting the anatomical meaning is different between our studies. Character state 1 here captures the distinctive morphology of the mandible in *Mekosuchus* (Balouet & Buffetaut, 1987) and *Iharkutosuchus makadii* (Ösi et al., 2007), in which the splenial is abruptly dorsally inclined at its posterior end (Fig. 102E–F). This contrasts with all other eusuchians where known, in which the dorsal margin of the splenial is largely straight and only modestly inclined, with no distinct change of slope posteriorly (Fig. 102C–D).

233. Splenial, anterior process within the dentary, medial to the posterior toothrow: absent (0); present (1) (new character, based on personal observations).

The derived character state describes an acute inflection of the splenial-dentary suture, lingual to the posteriormost dentary alveoli. Where preserved, this process occurs in all *Gavialis* species. This comprises *G. gangeticus* (Fig. 103A), *G. lewisi* (YPM 3226), and *G. benjawanicus* (Delfino & De Vos, 2010, fig.3; Martin et al., 2012, fig.4), as well as some indeterminate fossil *Gavialis*

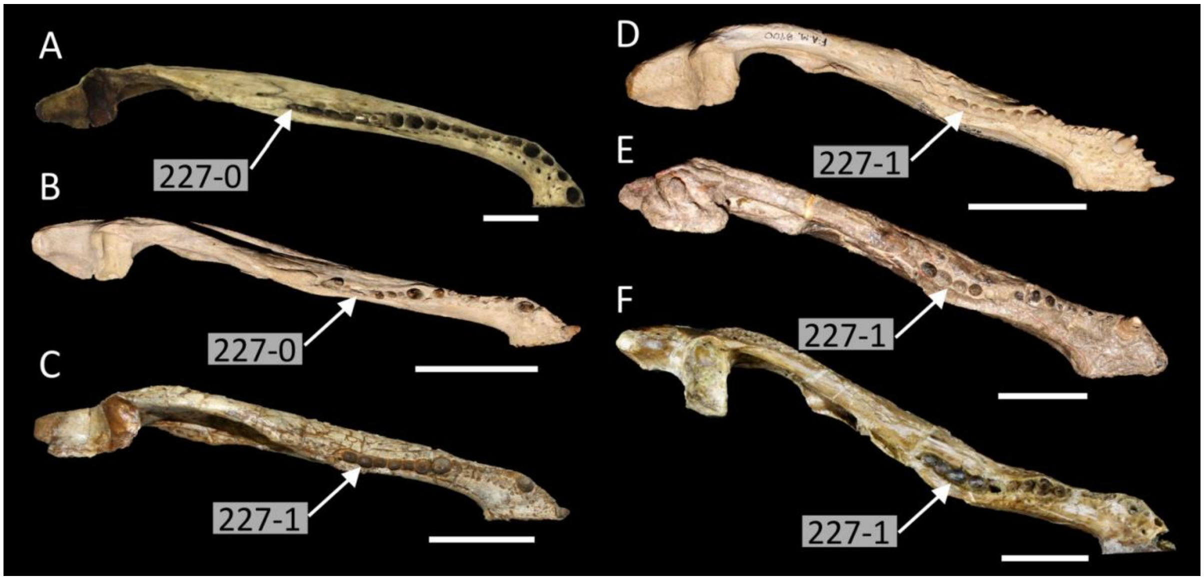

Figure 100: Dorsal view of the mandible in selected alligatorid taxa. **A**, *Alligator mississippiensis* (NHMUK 68.2.12.6); **B**, *Alligator mefferdi* (AMNH 7016); **C**, *Alligator prenasalis* (YPM-PV-14063); **D**, *Alligator mcgrewi* (AMNH FAM 8700); **E**, *Navajosuchus mooki* (AMNH 6780); **F**, *Allognathosuchus* sp. (USNM 25807). All scale bars = 5 cm.

specimens (Fig. 103C) . The condition is unknown in *G. browni* (AMNH 6279), for which the mandible is not preserved.

External mandibular fenestra

234. External mandibular fenestra: absent (0); present (1) (Clark, 1994 [75]; Brochu, 1997a [62]).

235. External mandibular fenestra, size: narrow slit, no discrete fenestral concavity on angular dorsal margin, foramen intermandibularis caudalis not visible (0); moderate discrete concavity on angular dorsal margin, foramen intermandibularis caudalis not visible (1); large, most of foramen intermandibularis caudalis visible (2) (after Norell, 1988 [14]; Brochu, 1999 [62]; Brochu, 2011 [63]; Brochu and Storrs, 2012 [63]) (ORDERED).

Characters 234 and 235 were derived by reductively coding Character 63 in Brochu and Storrs (2012). The external mandibular fenestra is absent in *Bernissartia fagesii* (Fig. 104A), and several non-crocodylian eusuchians, e.g. *Theriosuchus pusillus* (NHMUK 48304), *Iharkutosuchus makadii* (Ösi et al., 2007), and *Lohuecosuchus megadontos* (Narváez et al., 2015). Where present, variation occurs in the size of the fenestra. A small, slit-like fenestra (235-0) occurs in *Mekosuchus* (Fig. 104B), some *Borealosuchus* species (e.g. *B. threeensis* and *B. wilsoni* [Brochu et al., 2012]), and *Portugalosuchus azenhae* (Mateus et al., 2019). All other crocodylians exhibit notably larger fenestra, which can be divided into those in which the foramen intermandibularis caudalis (FIC) is

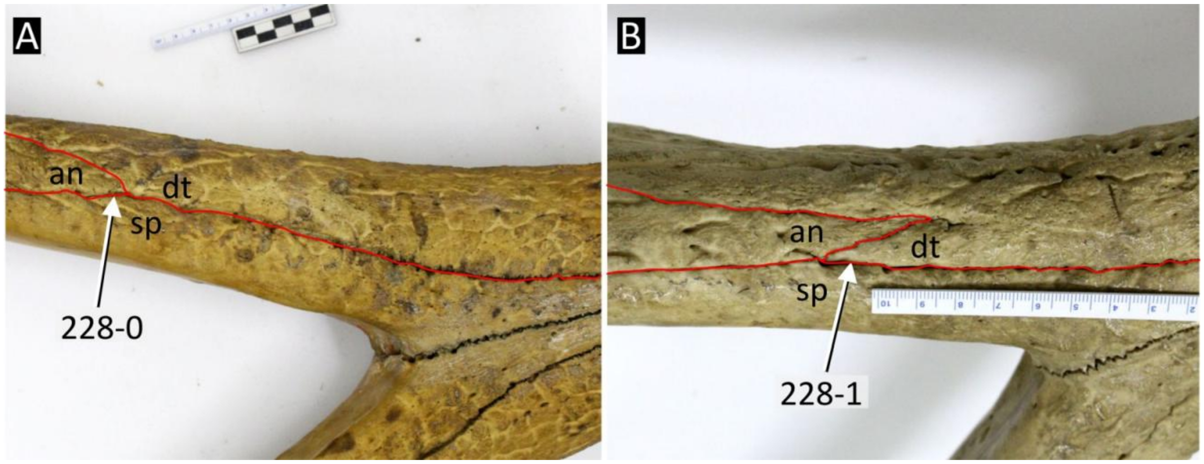

Figure 101: Ventral view of the mandible showing the suture between the dentary, angular and splenial. **A**, *Tomistoma schlegelii* (NHMUK 1894.2.21.1); **B**, *Gavialis gangeticus* (NHMUK 1974.3009). Abbreviations: **an**, angular; **dt**, dentary; **sp**, splenial. All scale bars = cm.

poorly visible through the fenestra (235-1) (Fig. 104G–I), and those with a largely exposed FIC (235-2) (Fig. 104D–E).

236. Surangular-dentary suture, intersection with external mandibular fenestra: anterior to posterodorsal corner (0); at posterodorsal corner (1) (Brochu, 1997a [65]).

This character is inapplicable to taxa without an external mandibular fenestra (234-0). In most crocodylians the surangular-dentary suture intersects the external mandibular fenestra at a shallow angle, anterior to the posterodorsal corner, e.g. *Alligator mississippiensis* (Fig. 104D), *Tomistoma schlegelii* (Fig. 104G), *Gavialis gangeticus* (Fig. 104H) and *Crocodylus* (Fig. 104J–K). By contrast, the surangular-dentary suture is posterodorsally shifted in some taxa, e.g. *Mekosuchus* (Fig. 104B), *Alligator mcgrewi* (Fig. 104C), *Caiman latirostris* (Fig. 104E), and *Procaimanoidea utahensis* (USNM 15996).

237. Surangular-angular suture, intersection with external mandibular fenestra (at maturity): at posterodorsal angle (0); at posterior margin (1); passes broadly along ventral margin (2) (after Norell, 1988 [40]; Brochu, 1997a [47]).

In most eusuchians with an external mandibular fenestra (EMF), the surangular-angular suture is horizontal up to the point where it intersects the posterior margin of the fenestra (237-1), e.g. *Alligator mississippiensis* (Fig. 104D), most *Crocodylus* species (Fig. 104J), and '*Crocodylus*' *affinis* (Fig. 104L). Less commonly, the suture intersects the EMF at a shallow angle, running down the posterior edge of the fenestra (237-2), e.g. *Caiman* (Fig. 104E–F), *Tomistoma schlegelii* (Fig. 104G), and *Gavialis gangeticus* (Fig. 104H). The plesiomorphic state is newly included to capture an uncommon condition wherein the suture intersects the EMF at its posterodorsal corner.

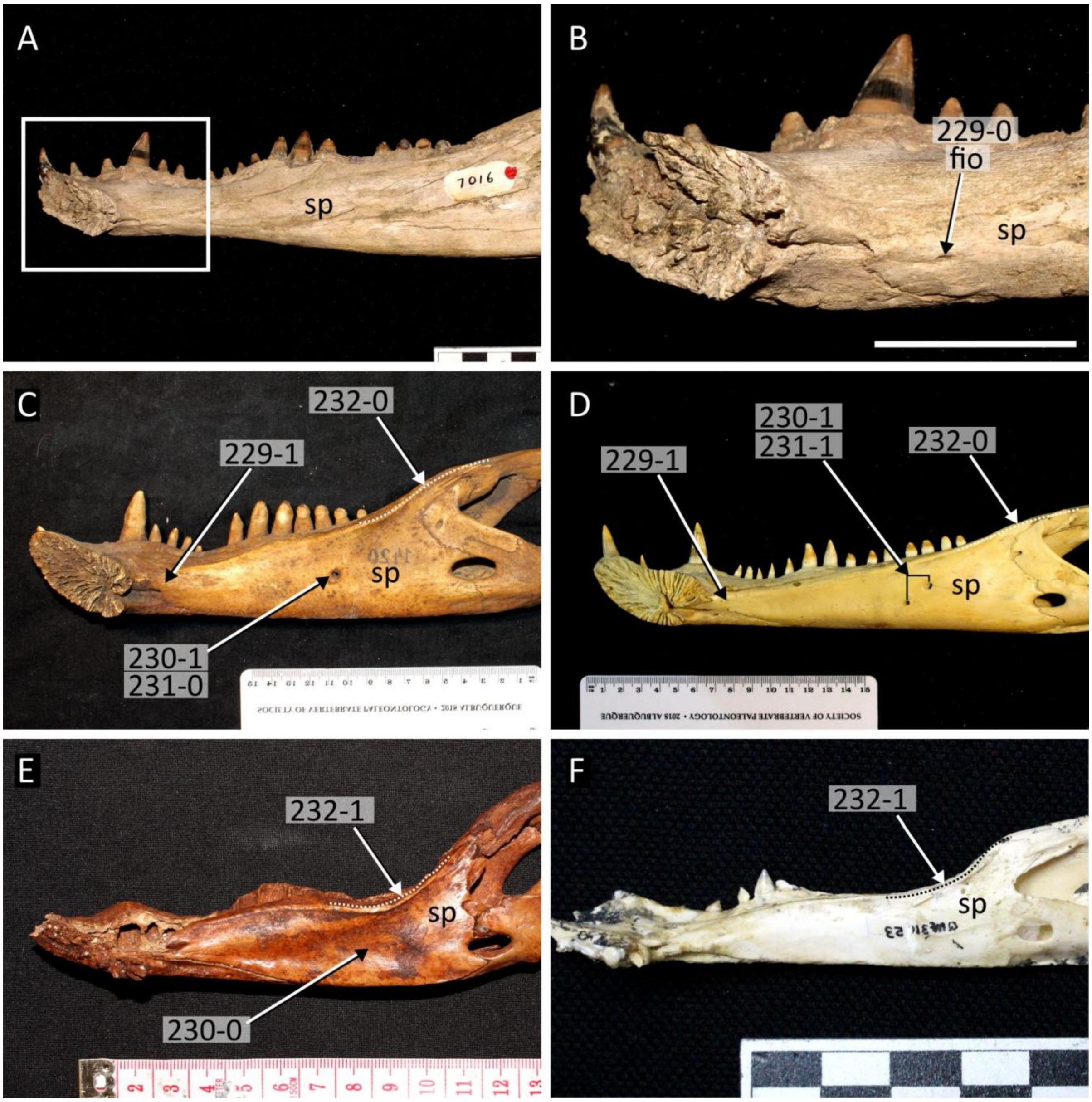

Figure 102: Medial view of the splenial. **A-B**, *Alligator mefferdi* (AMNH 7016); **C**, *Caiman latirostris* (MACN V 1420); **D**, *Caiman yacare* (MACN uncatalogued); **E**, *Mekosuchus inexpectatus* (MNHN NCP 06) (digitally reversed); **F**, *Mekosuchus whitehunterensis* (QM 31053). Abbreviations: **fio**, foramen intermandibularis oralis; **sp**, splenial. Scale bar in **B** = 4 cm.

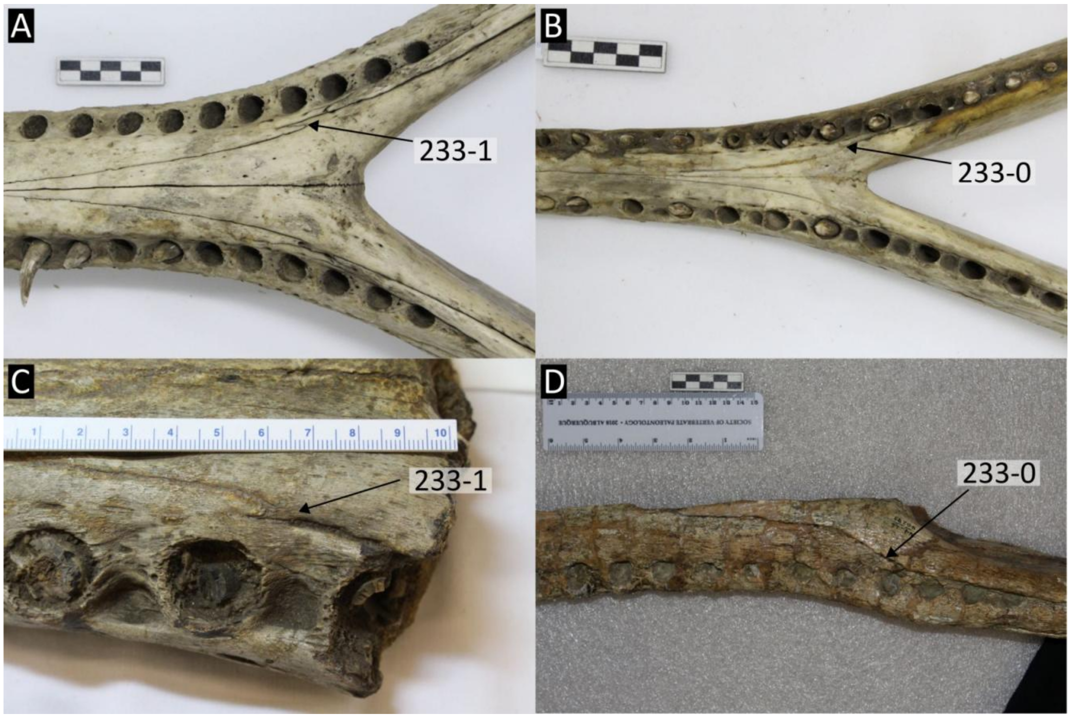

Figure 103: Dorsal view of the splenial symphysis in **A**, *Gavialis gangeticus* (NHMUK 1974.3009); **B**, *Tomistoma schlegelii* (NHMUK 1848.10.31.19); **C**, *Gavialis gangeticus* (NHMUK R 3095); **D**, *Gryposuchus colombianus* (UCMP 40293). All scale bars = cm.

Among taxa included in this study, this condition is only observed in *Alligator mcgrewi* (Fig. 104C), *Navajosuchus mooki* (AMNH 6780), *Mekosuchus* (Fig. 104B), and *Penghusuchus pani* (Shan et al., 2009, fig.4D). As the states do not capture a clearly continuous series, this character is not ordered.

238. Dentary, acute posterior process in the angular ventral to the external mandibular fenestra: present (0); absent (1) (after Jouve 2016 [240]).

In most eusuchians with an external mandibular fenestra, the dentary-angular suture approaches the ventral margin of the fenestra in a posterodorsal direction, before recurving sharply anteriorly to form an acute process (238-0) (Fig. 104C–G). By contrast, the suture simply intersects the ventral margin of the fenestra (238-1) in *Gavialis gangeticus* (Fig. 104H), *Gavialis lewisi* (YPM 3226), *Mekosuchus* (Fig. 104B), and *Ultrastenos willisi* (Stein et al., 2016, fig.4C). Based on character scores therein, Jouve (2016) considered *Gryposuchus colombianus* and *Toyotamaphimeia machikanensis* to also share this latter condition. However, this portion of the mandible is obscured in all specimens of *Gryposuchus colombianus* examined here (UCMP 40062, UCMP 40293), and albeit small, *Toyotamaphimeia* appears to possess a ventral process (238-0) (Kobayashi et al., 2006, fig.11B).

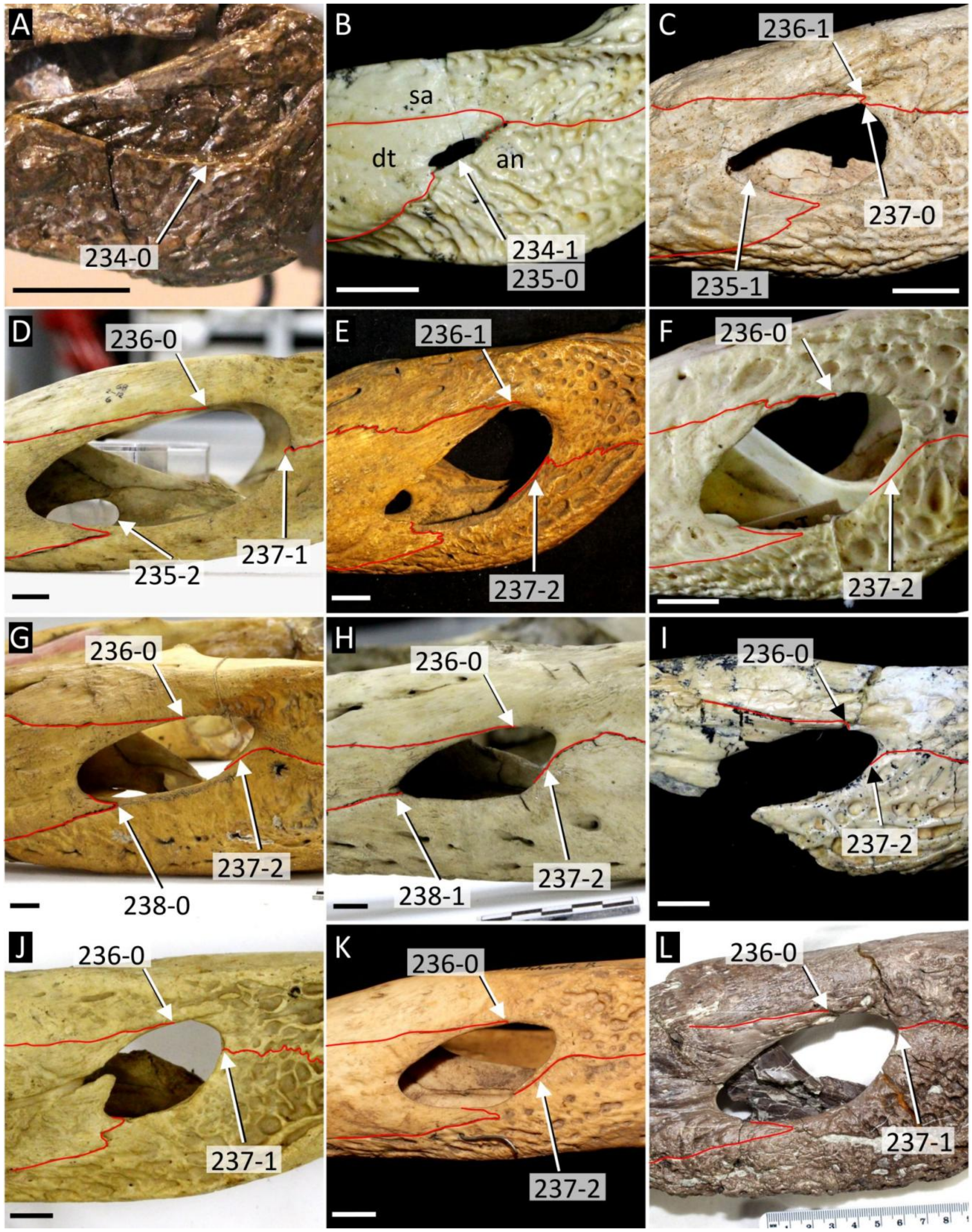

Figure 104: Left lateral view of the external mandibular fenestra in selected crocodylians, showing variation in size and sutural relationships. **A**, *Bernissartia fagesii* (IRSNB 1538); **B**, *Mekosuchus whitehunterensis* (QM 31053); **C**, *Alligator mcgrewi* (AMNH FAM 8700); **D**, *Alligator mississippiensis* (NHMUK 68.2.12.6); **E**, *Caiman latirostris* (MACN V 1420); **F**, *Caiman crocodilus chiapasius* (FMNH 73701); **G**, *Tomistoma schlegelii* (NHMUK 1894.2.21.1); **H**, *Gavialis gangeticus* (NHMUK uncatalogued); **I**, *Baru wickeni* (QM 31072); **J**, *Crocodylus palustris* (NHMUK 1897.12.31.1); **K**, *Crocodylus johnstoni* (QM J39230); **L**, *'Crocodylus' affinis* (UCMP 154341). Abbreviations: **an**, angular; **dt**, dentary; **sa**, surangular. All scale bars = 1 cm.

239. Angular and surangular, margins flush with lateral surface of mandible (0); margins everted forming flange (1) (after Lee and Yates, 2018 [199]).

The surangular and angular form the dorsal and ventral margins of the posterior mandibular ramus, respectively, and are flush with the remainder of the lateral mandibular surface in *Bernissartia fagesii* (IRScNB 1538) and most eusuchians (Fig. 105A). By contrast, some eusuchians exhibit prominent ridges in this region, notably *Mekosuchus inexpectatus* (Fig. 105B), *Mekosuchus whitehunterensis* (Fig. 105C), and *Voay robustus* (Fig. 105D). Although less prominent, homologous ridges are considered present in *Paleosuchus* (AMNH 66391, AMNH 93812), *Boverisuchus vorax* (USNM 12957), and some paralligatorids, e.g. *Theriosuchus pusillus* (NHMUK 48304) and *Shamosuchus djadochtaensis* (Pol et al., 2009). These ridges might serve as sites for muscle attachment on the mandible, and could potentially vary ontogenetically; however, they appear to occur at an early stage of ontogeny where known. For example, the material known for *Mekosuchus whitehunterensis* probabaly represents a juvenile based on its size, and yet this exhibits the dervied state (Fig. 105C). Similar ridges are also present at an early ontogenetic stage in *Paleosuchus* specimens studied here (e.g. AMNH 66391, AMNH 93812). Finally, these ridges were not observed in the largest extant crocodylian specimens studied here, e.g. *Tomistoma schlegelii* (NHMUK 1894.2.21.1), *Crocodylus palustris* (NHMUK 97.12.31.1), and *Crocodylus porosus* (NHMUK 1864.9.11.1).

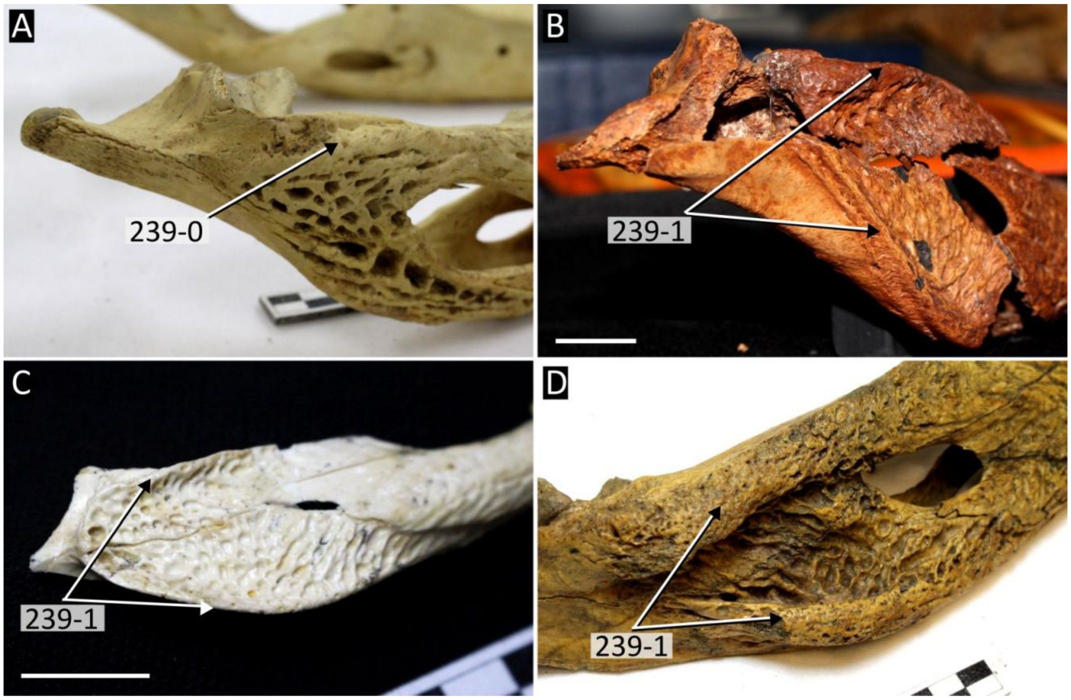

Figure 105: Posterolateral view of the mandible showing variation in development of a flange on the surangular. **A**, *Caiman latirostris* (NHMUK 1897.12.31.1); **B**, *Mekosuchus inexpectatus* (MNHN NCP 06); **C**, *Mekosuchus whitehunterensis* (QM 31053); **D**, *Voay robustus* (NHMUK R 36686). All scale bars = 2 cm.

240. Angular, fossa for *M. pterygoideus ventralis* visible on posterolateral surface of the mandible (0); not visible on posterolateral surface (1) (new character, based on personal observations).

In most eusuchians, the angular is broadly exposed ventral to the retroarticular process, as a smooth and un-pitted surface (240-0). This surface is separated from the remainder of the sculpted lateral mandibular surface by a shallow 'step'. This boundary might mark the anterior extent of insertion for *M. pterygoideus ventralis* (Bona & Desojo, 2011), and occurs in all extant *Crocodylus* species (Fig. 106A), *Alligator* (Fig. 106C), *Allognathosuchus* (Fig. 106E), *Eocaiman palaeocenicus* (Fig. 106G), and *Brachychampsa montana* (Fig. 106H). By contrast, the unornamented angular is minimally exposed ventral to the retroarticular process in all extant caimanines (Fig. 106D, F), *Diplocynodon hantoniensis* (Fig. 106B), and several non-crocodylian taxa including *Bernissartia fagesii* (IRScNB 1538), *Theriosuchus pusillus* (NHMUK 48304) and *Agaresuchus fontisensis* (Narváez et al., 2016, fig.4A).

Surangular

241. Surangular, relative length of the anterior processes: unequal, ventral process $<75\%$ anteroposterior length of dorsal process (measured from surangular foramen) (0); sub-equal, ventral process $\geq 75\%$ length of dorsal process (1) (after Brochu, 1997a [48]).

The presence of sub-equal anterior processes of the surangular (241-1) (Fig. 107C–D) is considered to be an unambiguous synapomorphy of Alligatoroidea (Brochu, 1999), contrasting with the unequal processes of *Tomistoma schlegelii* (Fig. 107A), *Gavialis gangeticus*, and all extant crocodylids (Fig. 107B). The qualifier 'sub-equal' is necessary, since the surangular processes are seldom equal in length, with the dorsal process extending further anteriorly than the ventral process in most eusuchians. For example, in *Alligator mississippiensis* (Fig. 107C), the dorsal process is slightly longer than the ventral process; however, the ventral process is consistently greater than 75% of the length of the dorsal process (measured from the surangular foramen) in all specimens. This contrasts with most other eusuchians, in which the ventral process is usually less than 50% the anteroposterior length of its dorsal counterpart. However, sub-equal surangular processes are not restricted to Alligatoroidea, occuring in a small number of other taxa, including *Eothoracosaurus mississippiensis* (Brochu, 2004a) and *Borealosuchus formidabilis* (Fig. 107D).

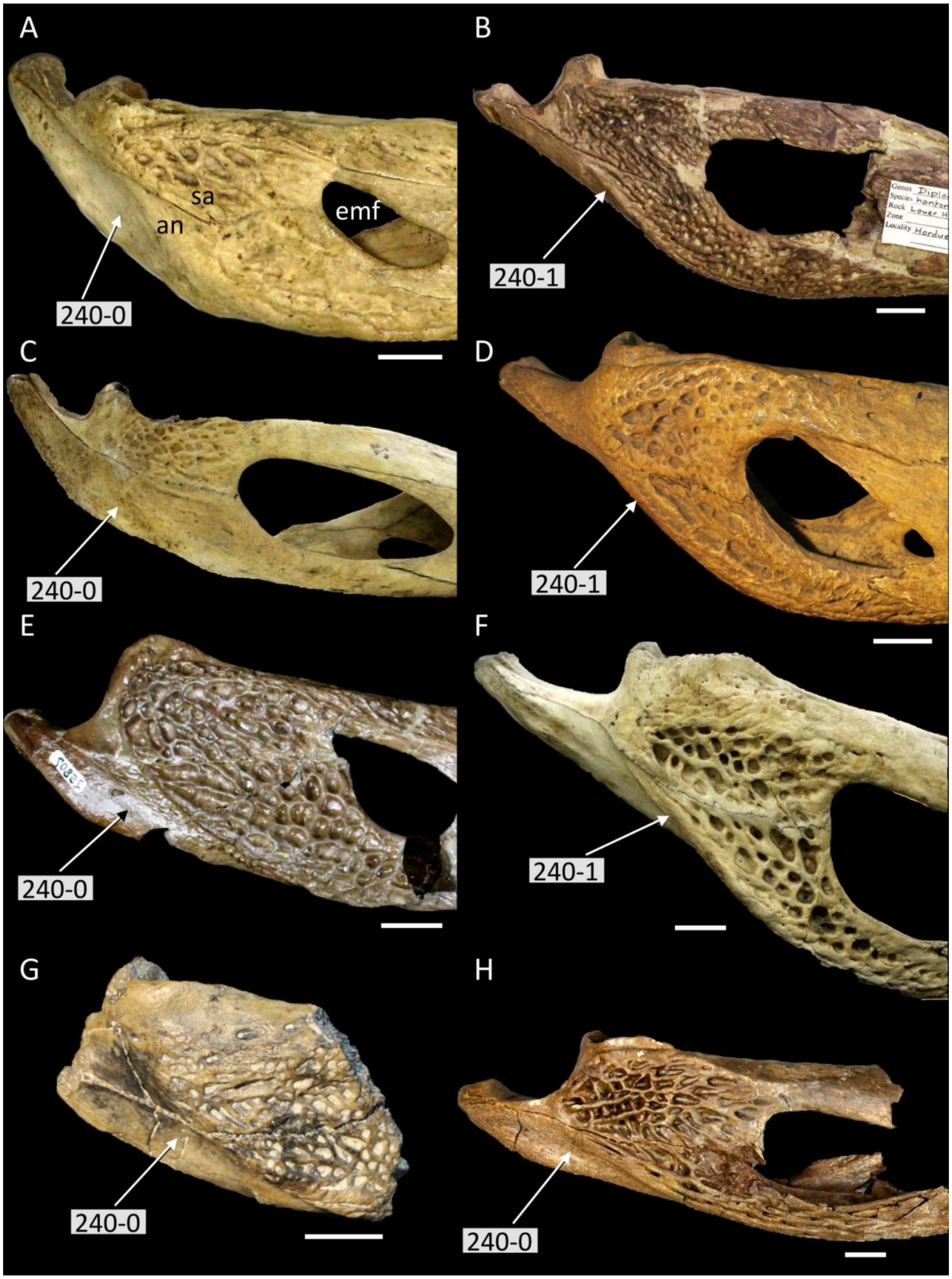

Figure 106: Lateral view of the posterior right mandible in selected crocodylians. **A**, *Crocodylus siamensis* (NHMUK 1921.4.1.168); **B**, *Diplocynodon hantoniensis* (CAMSM TN 904); **C**, *Alligator mississippiensis* (NHMUK 68.2.12.6, digitally reversed); **D**, *Caiman latirostris* (MACN V 1420); **E**, *Allognathosuchus* sp. (USNM 25807, digitally reversed); **F**, *Melanosuchus niger* (NHMUK 45.8.25.125); **G**, *Eocaiman palaeocenicus* (MACN 1914, digitally reversed); **H**, *Brachychampsa montana* (UCMP 133901, digitally reversed). Abbreviations: **an**, angular; **emf**, external mandibular fenestra; **sa**, surangular. All scale bars = 2 cm.

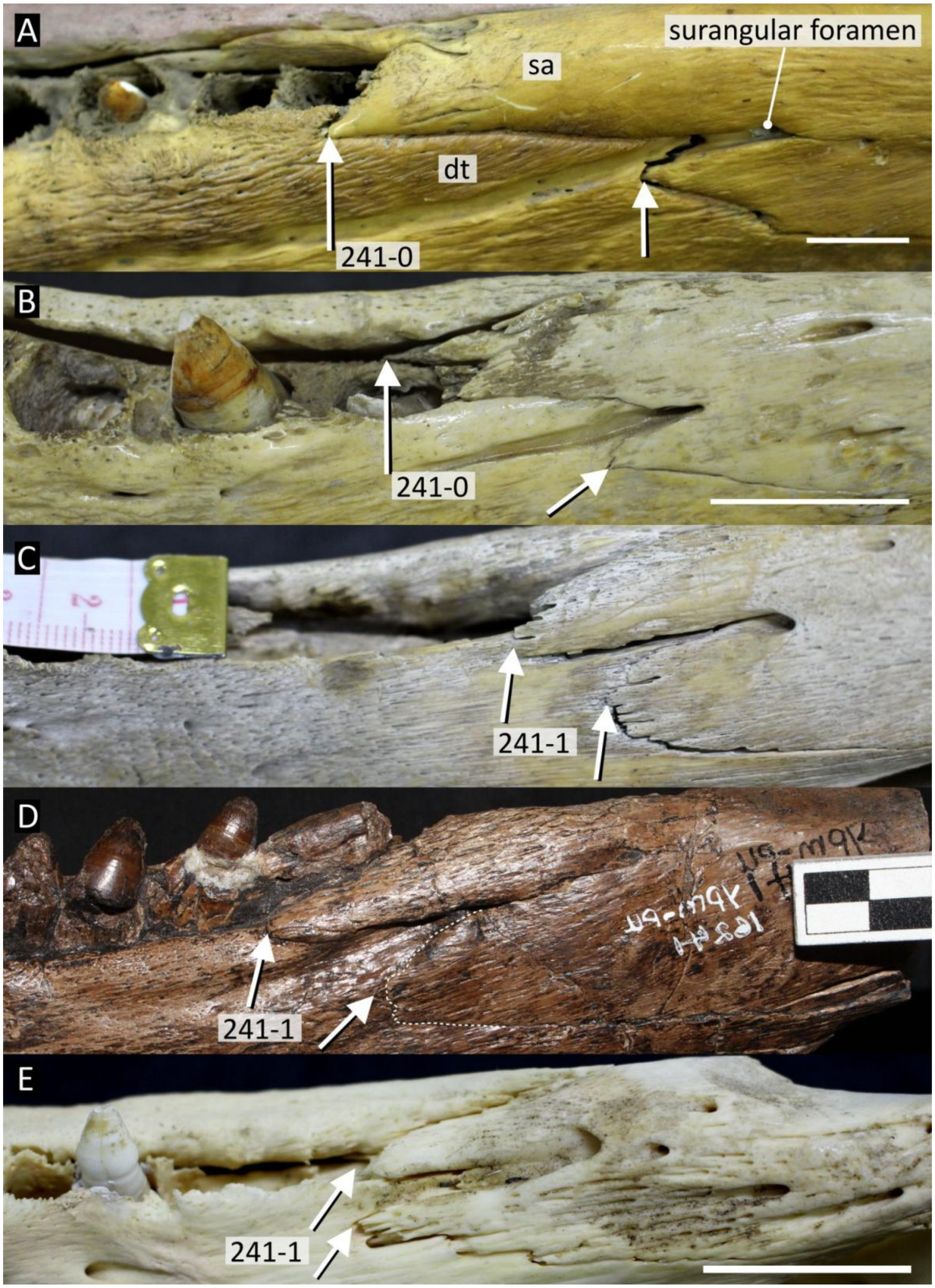

Figure 107: Dorsolateral view of the surangular showing relative lengths of the anterior processes in **A**, *Tomistoma schlegelii* (NHMUK 1894.2.21.1); **B**, *Crocodylus moreletii* (NHMUK 1861.4.1.4); **C**, *Alligator sinensis* (NHMUK X184); **D**, *Borealosuchus formidabilis* (YPM PU 16241, digitally reversed) **E**, *Caiman latirostris* (NHMUK 1897.12.31.1). Abbreviations: **dt**, dentary, **sa**, surangular. Scale bars in A, B and E = 2 cm, all other scale bars = cm.

242. Surangular, anterodorsal process (spur) lingual to posterior most dentary alveoli, between splenial and dentary: present (0); absent (1) (after Brochu, 1997a [61]).

243. Surangular, anterodorsal process (spur), anterior extent: not reaching 1 full alveolus (0); reaching 1–2 alveoli (1); reaching 3 or more alveoli (2) (new character, adapted from Brochu, 1997a [61]) (ORDERED).

The surangular 'spur' is an anterodorsal process of the surangular, which projects between the dentary and the splenial, lingual to the posteriormost dentary alveoli (Fig. 108). The original character was binary, describing the presence or absence of a spur adjacent to one alveolus length, as originally formulated by (Brochu, 1997b). In most previous datasets, this spur has been recognised in *Bernissartia fagesii*, "tomistomines", and "gavialoids" (e.g. Brochu et al., 2012; Iijima & Kobayashi, 2019; Jouve, 2016; Salas-Gismondi et al., 2016); indeed Brochu (1999) noted that very few non-longirostrine crocodylians possess it. Here, this character has been reductively coded, with a new character capturing variation in spur length (Character 243). The surangular spur is recognised much more widely than previous studies, including in many non-longirostrine crocodylians. For example, all extant *Crocodylus* species exhibit a spur, which can extend either less than one alveolus length (243-0) (e.g. *C. siamensis* [Fig. 108C]), or between 1–2 alveoli (243-1) (e.g. *C. palustris* [Fig. 108D]). An elongated spur extending the length of 3 alveoli (243-2) is restricted to a few longirostrine crocodylians, including *Tomistoma schlegelii* (Fig. 108E) and *Gavialis gangeticus* (Fig. 108F). Taxa which lack a spur altogether include all extant alligatorids (Fig. 108A–B), for which Character 243 is inapplicable. The latter character is ordered, given the continuous nature of an increasingly.

244. Surangular, ascending process on lateral wall of glenoid fossa: present (0); absent (1) (Brochu, 1997a [106]).

As originally formulated, the plesiomorphic character state described an ascending process of the surangular that reaches the "*dorsal tip of [the] lateral wall of [the] glenoid fossa*" (Brochu, 1997b). The character wording has been modified here as, even in taxa with an ascending process, it never fully reaches the tip of the glenoid fossa lateral wall, and a small portion of the articular is always exposed. An ascending process occurs in *Bernissartia fagesii* (IRScNB 1538), several non-crocodylian eusuchians (e.g. *Iharkutosuchus makadii* [Ősi et al., 2007] and *Theriosuchus pusillus* [NHMUK 48304]), and many crocodylians, e.g. *Gavialis gangeticus* (Fig. 109A), all extant caimanines (Fig. 109C), *Diplocynodon* (Fig. 109D), and *Borealosuchus*, e.g. *B. sternbergii* (USNM 6533).

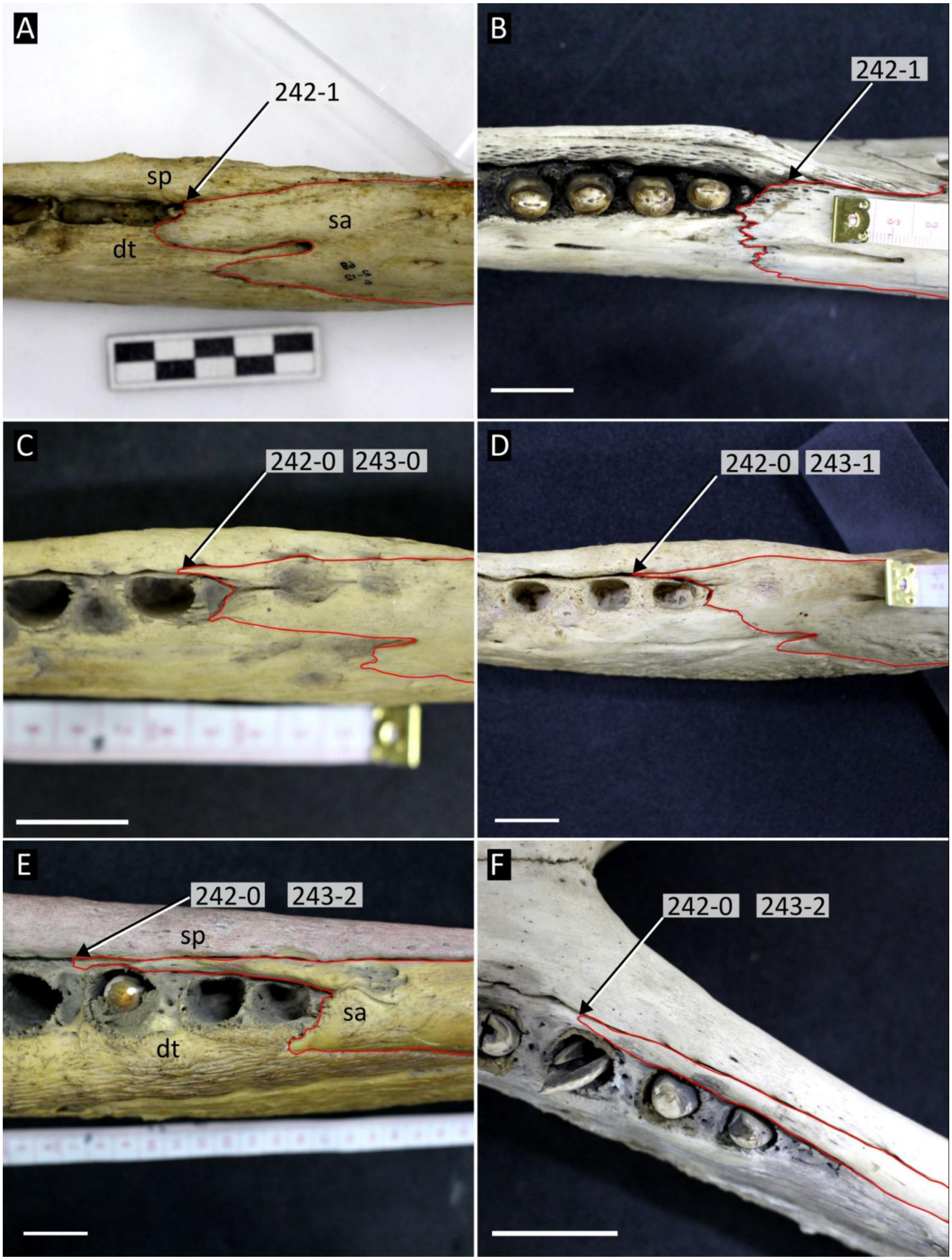

Figure 108: Dorsal view of the posterior mandibular toothrow showing variation in development of the surangular spur. **A**, *Alligator mississippiensis* (NHMUK 68.2.12.6); **B**, *Melanosuchus niger* (NHMUK 45.8.25.125); **C**, *Crocodylus siamensis* (NHMUK 1921.4.1.171); **D**, *Crocodylus palustris* (NHMUK 1897.12.31.1); **E**, *Tomistoma schlegelii* (NHMUK 1894.2.21.1); **F**, *Gavialis gangeticus* (NHMUK uncatalogued). Abbreviations: **dt**, dentary; **sa**, surangular; **sp**, splenial. All scale bars = 2 cm.

245. Surangular, posterior extent on lateral margin of retroarticular process: reaches posterior tip (0); pinches out anterior to posterior tip (1) (after Norell, 1988 [42]; Brochu, 1997a [51]).

The anatomical meaning of this character is identical to Brochu (1997b). The surangular extends to the posterior tip of the retroarticular process in *Borealosuchus* (e.g. *B. sternbergii*, USNM 6533), *Tomistoma schlegelii* (NHMUK 1894.2.21.1), all extant caimanines (Fig. 109C), and all extant species of *Crocodylus*. By contrast, *Bernissartia fagesii* (IRScNB 1538), *Gavialis gangeticus* (Fig. 109A), *Alligator* (Fig. 109B), and some *Diplocynodon* species (Fig. 109D) exhibit a posteriorly truncated surangular.

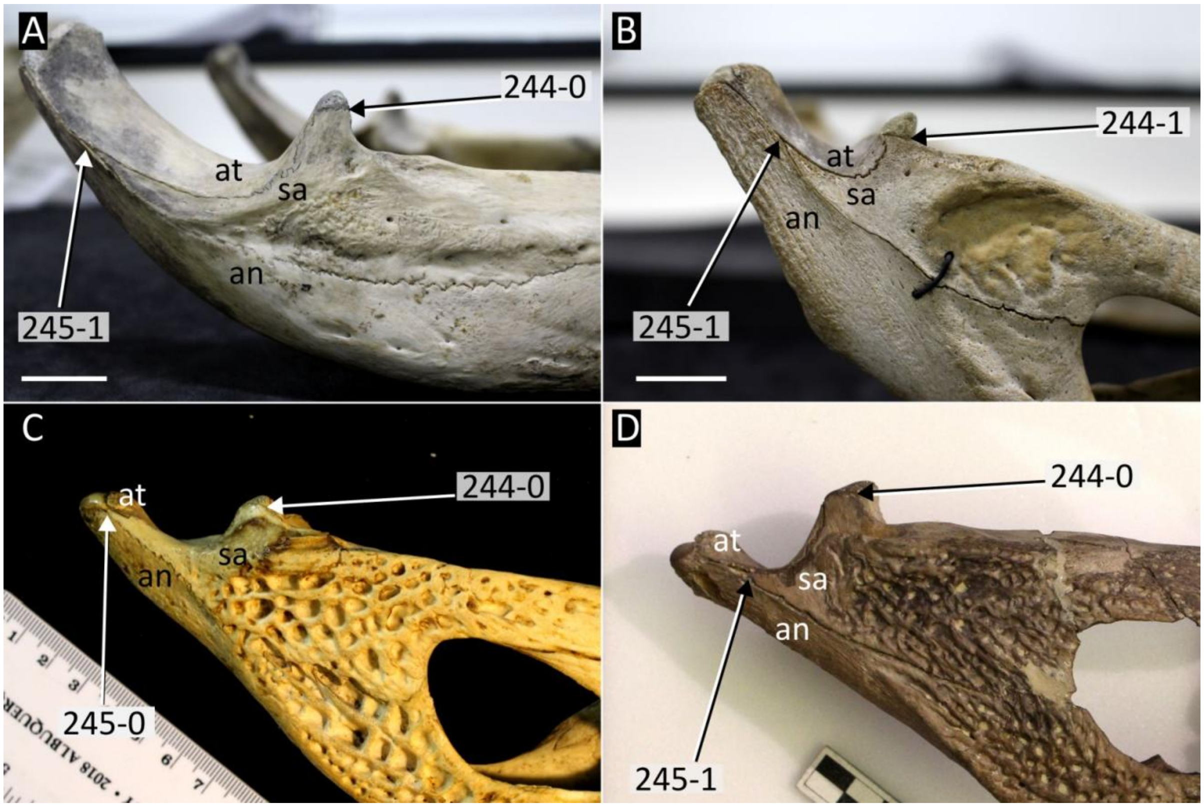

Figure 109: Variation in dorsal and posterior extent of the surangular. **A**, *Gavialis gangeticus* (NHMUK uncatalogued specimen); **B**, *Alligator sinensis* (NHMUK X 184); **C**, *Caiman yacare* (MACN uncatalogued specimen); **D**, *Diplocynodon hantoniensis* (CAMSM TN 904). Abbreviations: **an**, angular; **at**, articular; **sa**, surangular. All scale bars = 2 cm.

246. Surangular, sulcus on dorsal margin lateral to glenoid fossa: absent (0); present (1) (after Wang et al. 2016; Lee and Yates, 2018 [204]).

Wang et al. (2016) described a pit on the dorsolateral margin of the surangular, adjacent to the glenoid fossa, which they considered diagnostic of *Asiatosuchus nanlingensis*. Lee and Yates (2018 [character scores therein]), recognised that this fossa is more common within Crocodylia, occurring in some mekosuchines e.g. *Kambara* (Fig. 110B). Here, a pit is also recognised in *Bernissartia*

fagesii (IRScNB 1538), *Kentisuchus spenceri* (Fig. 110C), some *Borealosuchus* species (e.g. *B. sternbergii* [USNM 6533] and *B. formidabilis* [YPM PU 16241]) and some 'basal' crocodyloids, e.g. *Asiatosuchus depressifrons* (Fig. 110D) and '*Crocodylus*' *affinis* (UCMP 154341).

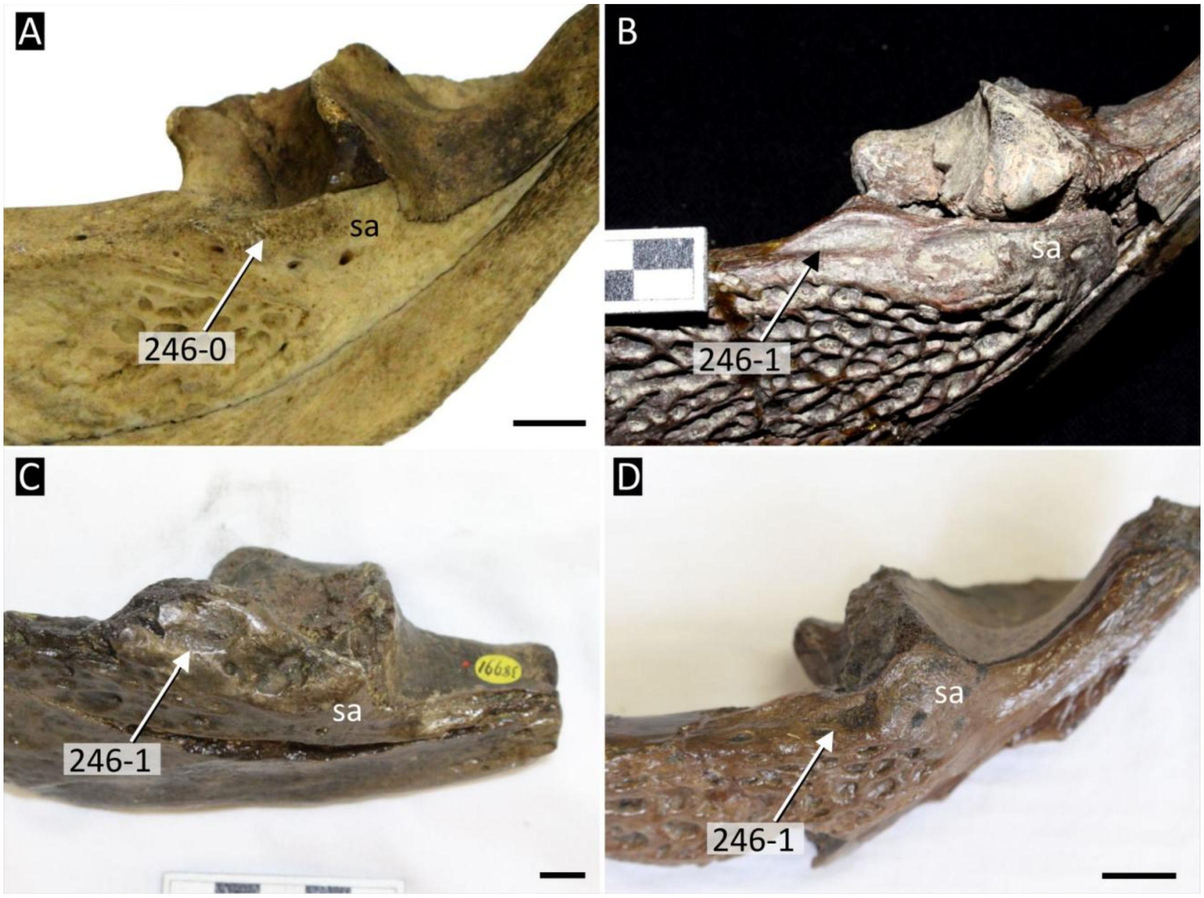

Figure 110: Lateral view of the surangular showing variation in development of a pit on the dorsolateral margin. **A**, *Alligator mississippiensis* (NHMUK); **B**, *Kambara molnari* (QM F12364); **C**, *Kentisuchus spenceri* (NHMUK 38991); **D**, *Asiatosuchus depressifrons* (IRScNB IG 9912). Abbreviations: **sa**, surangular. All scale bars = 1 cm, scale bar C = cm.

247. Surangular-articular suture, shape in glenoid fossa: straight, oriented anteroposteriorly (0); bowed laterally (1) (after Brochu, 1997a [162]).

In *Bernissartia fagesii* (IRScNB 1538) and most eusuchians, the surangular-articular suture is orientated in a straight, anteroposterior line in the floor of the glenoid fossa. This condition occurs in all extant alligatorids (e.g. *Alligator mississippiensis* [Fig. 111A]), most "gavialoids" (e.g. *Gavialis gangeticus*), *Diplocynodon* (e.g. *D. hantoniensis* [NHMUK OR 25188]), and *Borealosuchus* (e.g. *B. sternbergii* [USNM 6533]). By contrast, all extant crocodylids as well as "tomistomines", exhibit an acute 'kink' in the suture (Fig. 111B–C). This condition also occurs in the 'basal' crocodyloids, *Asiatosuchus depressifrons* (IRScNB R253) and '*Crocodylus*' *affinis* (UCMP 154341).

Articular

248. Articular, position of foramen aerum: at medial margin of retroarticular process (0); inset from medial margin of retroarticular process (1) (after Norell, 1988 [16]; Brochu, 1997a [49]).

The foramen aerum, which is positioned on the transverse ridge of the articular, is inset from the medial edge (248-1) in all extant alligatorids (Fig. 111A), as well as *Diplocynodon* (Fig. 111B) and *Leidyosuchus canadensis*. Brochu (1999) noted the potential linkage of this character with that describing the position of the foramen aerum on the quadrate (Character 117 here). Indeed, most taxa with a dorsally positioned quadratic foramen aerum (117-1) also possess a medially inset articular foramen aerum (248-1). Nevertheless, several taxa exhibit different combinations of these characters. For example, in *Mekosuchus inexpectatus* (MNHN NCP 06), *Borealosuchus sternbergii* (USNM 6533), and *Borealosuchus formidabilis* (Erickson, 1976), the quadratic foramen aerum is dorsally positioned (117-1), but the articular foramen aerum is medially positioned (248-0).

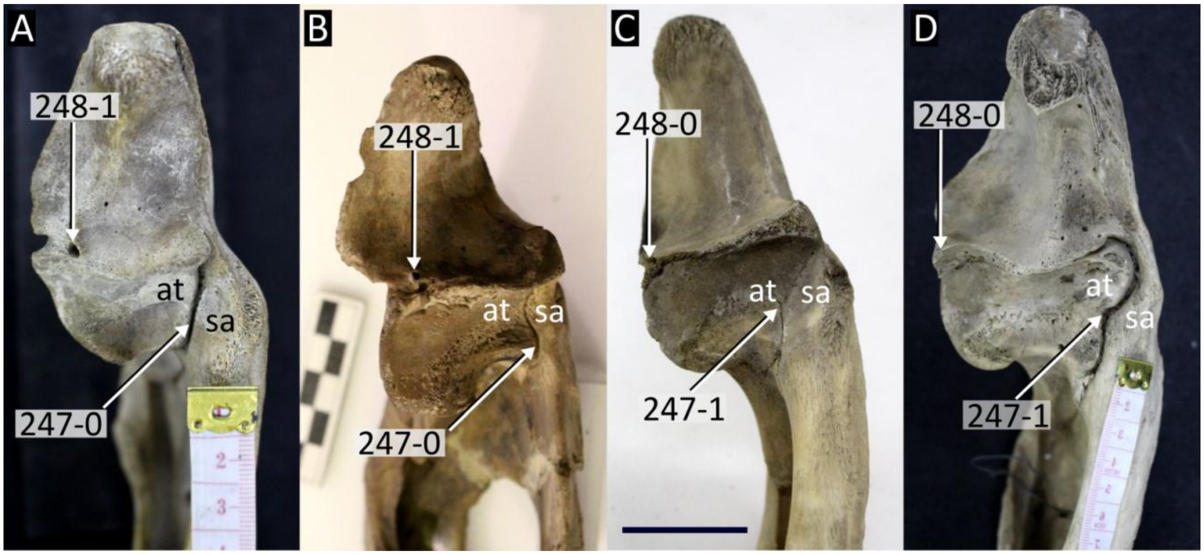

Figure 111: Dorsal view of the glenoid fossa in **A**, *Alligator sinensis* (NHMUK X184); **B**, *Diplocynodon hantoniensis* (CAMSM TN 904, digitally rversed); **C**, *Crocodylus siamensis* (NHMUK 1921.4.1.171); **D**, *Crocodylus porosus* (NHMUK 1864.9.11.1). Abbreviations: **at**, articular; **sa**, surangular. Scale bar C = 2 cm, all other scale bars = cm.

249. Articular, lamina extending from posterior edge of foramen aerum: absent (0); present (1) (new character, based on personal observations).

In *Bernissartia fagesii* (IRScNB 1538) and most eusuchians, the articular foramen aerum is a simple perforation, the margins of which are flush with the surface of the articular (Fig. 112A). By contrast, the foramen aerum of some crocodylians is posteriorly bound by a large, anteroposteriorly orientated lamina, which in some cases overhangs the foramen. This condition is mainly

observed in caimanines, such as *Caiman latirostris* (Fig. 112B), *Mourasuchus atopus* (Fig. 112C), and *Eocaiman palaeocenicus* (Fig XE). A similar condition occurs in *Diplocynodon hantoniensis* (Fig. 112D), *Brachychampsa montana* (Fig. 112F), and the “gavialoid” *Eosuchus minor* (Brochu, 2006a, fig.18).

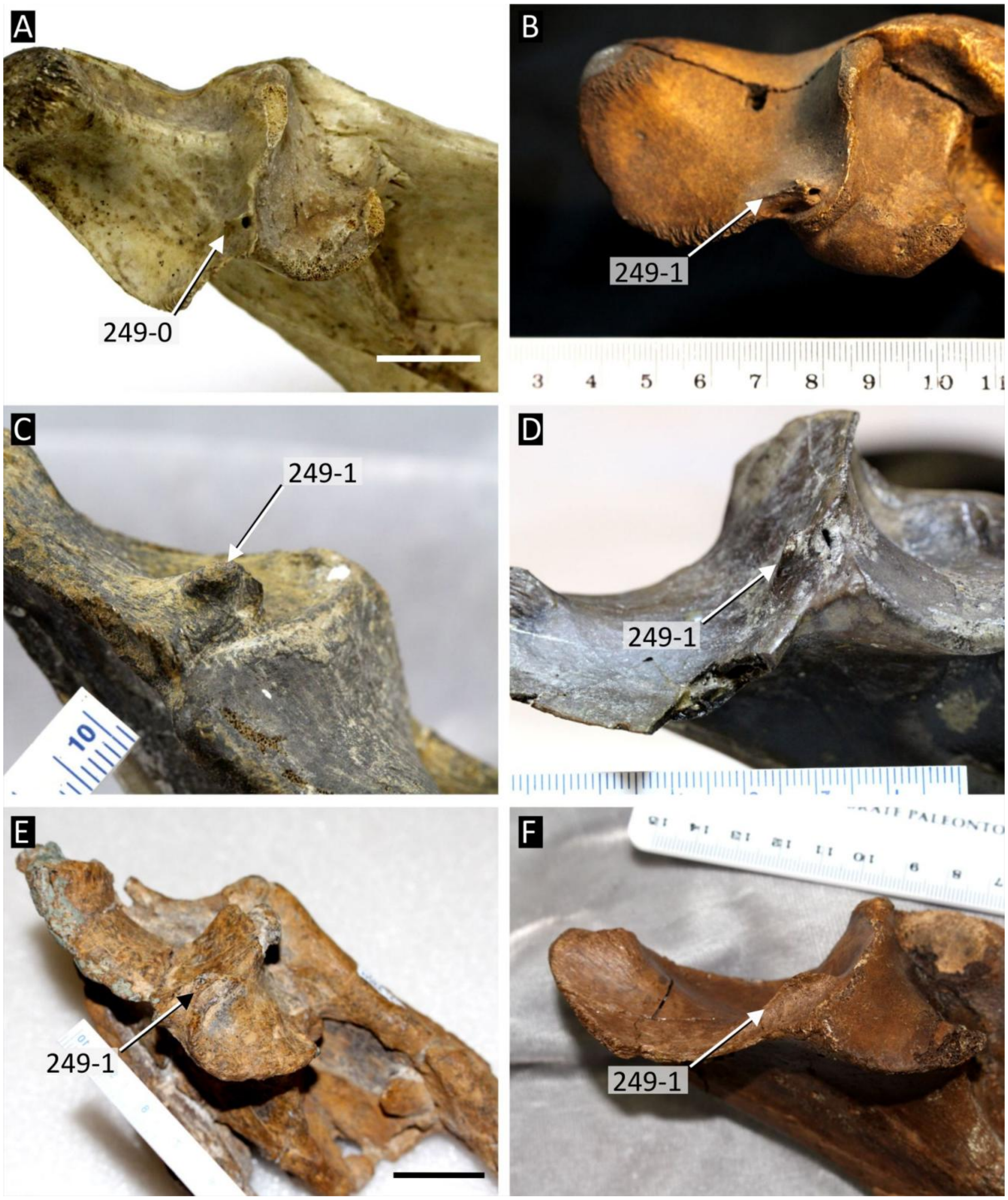

Figure 112: Dorsomedial view of the articular showing variation in development of a lamina trailing from the posterior margin of the foramen aerum. **A**, *Crocodylus siamensis* (NHMUK 1921.4.1.171); **B**, *Caiman latirostris* (MACN V 1420); **C**, *Mourasuchus atopus* (UCMP 38012); **D**, *Diplocynodon hantoniensis* (NHMUK 30397); **E**, *Eocaiman palaeocenicus* (MPEF 1933a); **F**, *Brachychampsa montana* (UCMP 133901). Scale bars in A and E = 2 cm, all other scale bars = cm.

250. Articular, orientation of retroarticular process: projects posteriorly (0); projects posterodorsally (1) (after Benton and Clark, 1988; Norell and Clark, 1990 [7]; Clark, 1994 [71]; Brochu, 1997a [50]).

251. Articular, dorsal extent of retroarticular process: at the same level or ventral to posterior edge of articular fossa (0); dorsal to posterior edge of articular fossa (1) (after Jouve, 2004 [190]; Jouve et al., 2008 [190]; Salas-Gismondi et al., 2015 [71]).

The retroarticular process is directed posteriorly (250-0) in *Bernissartia fagesii* (IRScNB 1538) and several non-crocodylian eusuchians, including *Theriosuchus pusillus* (Fig. 113A), and *Shamosuchus djadochtaensis* (Pol et al., 2009). The only crocodylian found to exhibit this condition is *Mekosuchus inexpectatus* (Fig. 113B). All other crocodylians exhibit a posterodorsally directed retroarticular process (250-1) (Fig. 113C–F). The retroarticular process also varies in its dorsal extent relative to the glenoid fossa of the articular. This would appear to be linked to the orientation of the retroarticular process; indeed, in all taxa with a posteriorly directed retroarticular process (250-0), it does not surpass the glenoid fossa dorsally (251-0) (Fig. 113A–B). However, taxa scored for character state 250-1 can exhibit either a low retroarticular process (251-0) (e.g. *Diplocynodon hantoniensis* and *Caiman latirostris* [Fig. 113C–D]) or a dorsally positioned process (251-1) (e.g. *Alligator mississippiensis* and *Gavialis gangeticus* [Fig. 113E–F]).

252. Articular, sharp longitudinal crest on dorsal surface of retroarticular process: absent (0); present (1) (after Salas-Gismondi et al. 2016 [203]).

The dorsal surface of the retroarticular process is slightly convex in all crocodylians, with a low anteroposterior ridge running along the midline (Fig. 114A). In a few, mostly "gavialoid" crocodylians, a tall crest is present instead, e.g. *Gryposuchus colombianus* (Fig. 114B) and *Argochampsa krebsi* (Fig. 114C). The development of this crest does not appear to be ontogenetic. For example, it does not occur in any specimen of *Gavialis gangeticus* studied here, including the largest individuals (e.g. NHMUK 1974.3009, UMZC R5783). Conversely, it does occur in a very small, and potentially juvenile specimen of *Argochampsa krebsi* (Fig. 114C).

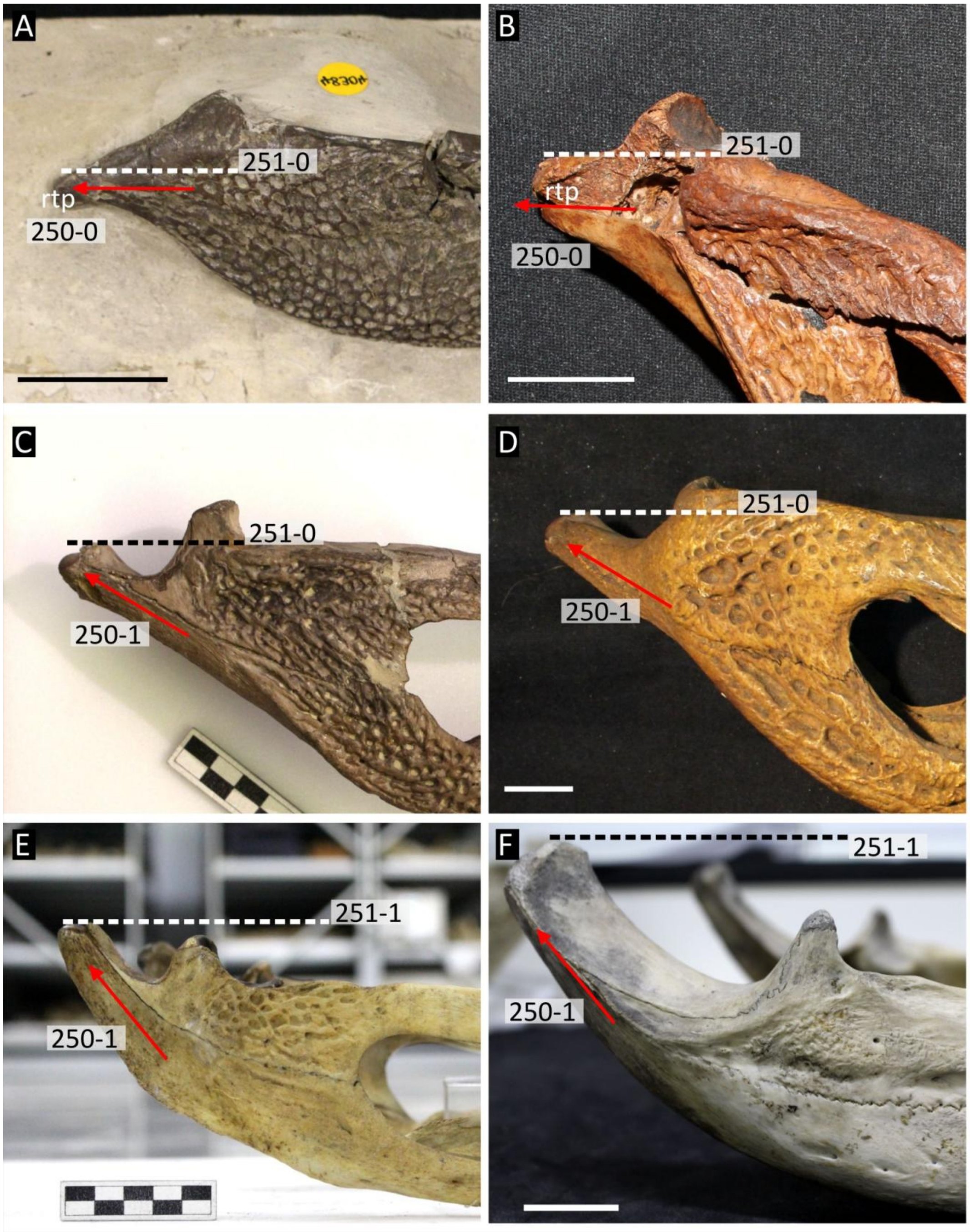

Figure 113: Lateral view of the posterior mandibular ramus showing variation in height and orientation of the retroarticular process. **A**, *Theriosuchus pusillus* (NHMUK 40384) (digitally reversed); **B**, *Mekosuchus inexpectatus* (MNHN NCP 06); **C**, *Diplocynodon hantoniensis* (CAM TN 904); **D**, *Caiman latirostris* (MACN V 1420) (digitally reversed); **E**, *Alligator mississippiensis* (NHMUK 68.2.12.6); **F**, *Gavialis gangeticus* (NHMUK uncatalogued). Abbreviations: **rtp**, retroarticular process. Scale bars C and E = cm, all other scale bars = 2 cm.

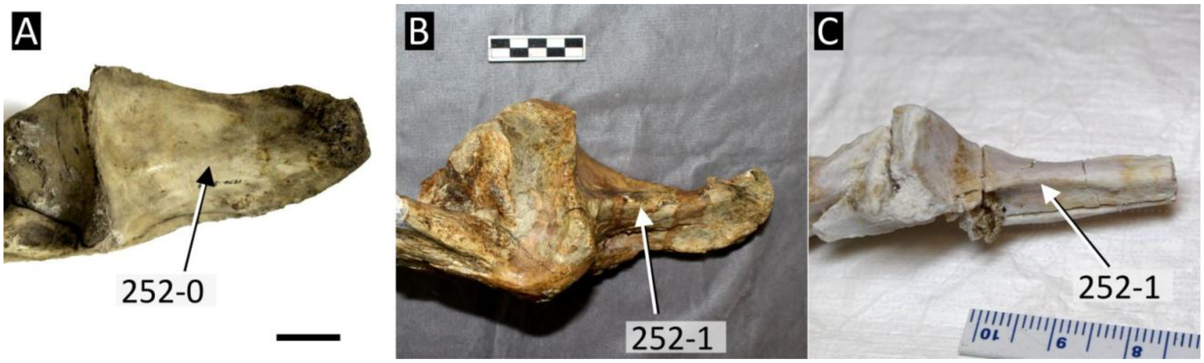

Figure 114: Dorsal view of the mandibular retroarticular process showing development of a crest in **A**, *Gavialis gangeticus* (NHMUK uncatalogued, left articular); **B**, *Gryposuchus colombianus* (UCMP 40293, left articular); **C**, *Argochampsa krebsi* (NHMUK R36872, right articular). Scale bar in A = 2 cm, all other scale bars = cm.

253. Articular, lingual foramen for articular and alveolar nerve perforates surangular only (0); perforates surangular-articular suture (1) (after Brochu, 1997a [45]; Brochu, 2011 [69]).

This character is described and illustrated by Brochu (1999, fig.33). Here it is unmodified, except for the description of the lingual foramen as perforating the surangular-*articular* suture, rather than the surangular-*angular* suture, which appears to be a typographical error. As in earlier studies, a surangular-articular perforation (253-1) is observed in all extant species of *Crocodylus* (Fig. 115G–I) and *Alligator* (Fig. 115B), in addition to *Kambara* (e.g. QM F30077), and *Mekosuchus* (e.g. MNHN NCP 06). The condition also occurs in *Diplocynodon* (all species, where preserved), although it is polymorphic in *D. hantoniensis* (Chapter 2). A surangular-only perforation (253-0) occurs in all extant caimanines (Fig. 115D–F), *Gavialis gangeticus* (Fig. 115C), *Tomistoma schlegelii* (NHMUK 1894.2.21.1), and some *Borealosuchus* species, e.g. *B. sternbergii* (USNM 6533).

254. Articular, anterior process on posterior wall of adductor chamber: absent (0); present (1) (after Brochu, 1997a [44]; Brochu, 2011 [68]).

255. Articular, position of anterior process on posterior wall of adductor chamber: dorsal to lingual foramen (0); ventral to lingual foramen (after Brochu, 1997a [44]; Brochu, 2011 [68]).

Characters 254 and 255 were derived by reductively coding Character 68 in Brochu (2011). In *Bernissartia fagesii* and most eusuchians, the surangular-articular suture forms a straight line in the posterior wall of the mandibular adductor chamber (254-0) (Fig. 115A–C). By contrast, all extant crocodylids, *Mleanosuchus*, and *Caiman* exhibit an anterior process of the articular (254-1) (Fig. 115D–I). Whereas in crocodylids this process is dorsal to the lingual foramen (255-0) (Fig. 115G–I), it occurs ventral to the lingual foramen in *Melanosuchus* and *Caiman* (255-1) (Fig. 115D–F). An additional character state introduced by Brochu (2011) (68-3): "*bears laminae* (=

processes) *above and below foramen*", appears to occur in some species of *Thecachampsa*, based on character scores in Brochu (2011) and Iijima and Kobayashi (2019). However, the relevant portion of the mandible could not be examined in any specimen of *Thecachampsa*, nor has it been clearly figured before, and so this state was excluded.

256. Surangular-angular suture, lingual intersection with articular in the floor of the adductor chamber: at ventral tip (0); dorsal to ventral tip (1) (after Brochu, 1997a [67]).

In postero-medial view of the mandibular adductor chamber, the surangular-angular suture can be seen extending from the external mandibular fenestra to the ventral tip of the articular in most eusuchians (Fig. 115A–C). This suture is more or less straight, but can exhibit a kink (commonly in *Crocodylus* species [Fig. 115G]). By contrast, the suture intersects the articular dorsal to its ventral tip in all extant caimanines (Fig. 115D–F) (Brochu, 1999). Among fossil crocodylians, this condition occurs in *Acresuchus pachytemporalis* (UFAC 2507), *Diplocynodon hantoniensis* (Chapter 2), and *Voay robustus* (NHMUK R36686). Commonly in taxa exhibiting this condition, the surangular forms a narrow, 'finger'-like descending process on the posterior wall of the adductor chamber, e.g. *Melanosuchus niger* (Fig. 115D) and *Caiman yacare* (Fig. 115E); however, this does not occur in *Caiman latirostris* (Fig. 115F), *Caiman crocodilus* (FMNH 69812), *Voay robustus* (NHMUK R36686), or *Diplocynodon hantoniensis* (CAM TN 904).

Angular

257. Angular, anterior extent relative to foramen intermandibularis caudalis (FIC) (in medial view): extends anteriorly beyond half the anteroposterior length of the FIC (0); terminates at, or posterior to the anteroposterior mid-length of the FIC (1) (after Brochu, 1997a [66]).

The anatomical meaning of this character follows the description and figures in Brochu (1999, fig.47), who noted that all extant caimanines exhibit an angular that does not extend far anteriorly relative to the FIC. Here, the anterior extent of the angular is measured relative to the anteroposterior mid-point of the FIC, and the derived condition is recognised in a few additional taxa. Following (Brochu, 1999), all extant caimanines exhibit the derived condition, and it is newly recognised in *Mecistops cataphractus* (Fig. 116) and *Alligator mcgrewi* (Fig. 116C).

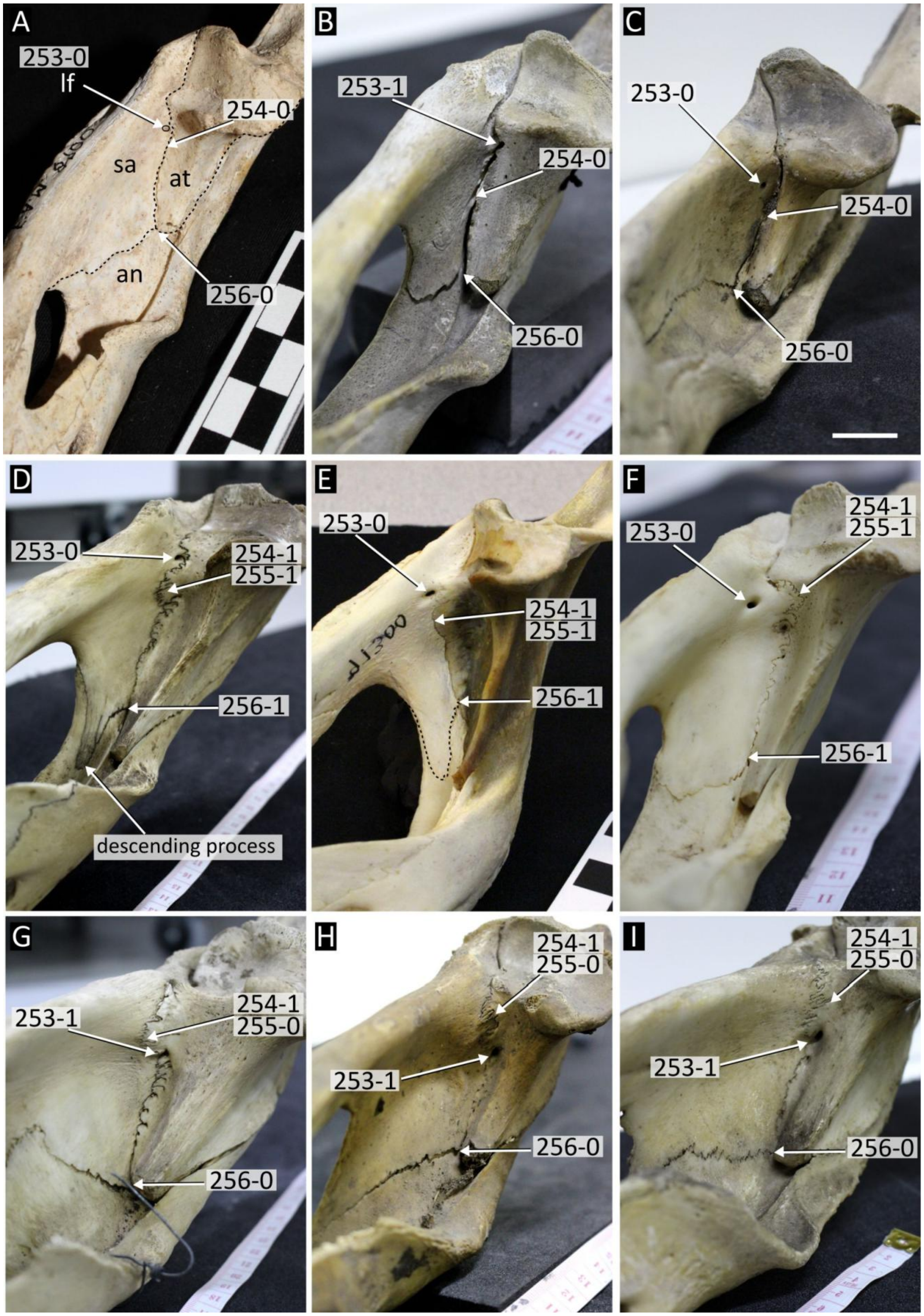

Figure 115: Posteromedial view of the mandibular adductor chamber. **A**, *Alligator mcgrewi* (AMNH FAM 8700) (digitally reversed); **B**, *Alligator sinensis* (NHMUK X184); **C**, *Gavialis gangeticus* (NHMUK uncatalogued); **D**, *Melanosuchus niger* (NHMUK 45.8.25.125); **E**, *Caiman yacare* (AMNH 97300) (digitally reversed); **F**, *Caiman latirostris* (NHMUK 86.10.4.2); **G**, *Crocodylus porosus* (NHMUK 1864.9.11.1); **H**, *Crocodylus siamensis* (NHMUK 1921.4.1.171); **I**, *Crocodylus moreletii* (NHMUK 1861.4.1.4). Abbreviations: **an**, angular; **at**, articular; **lf**, lingual foramen; **sa**, surangular. Scale bar in C = 2 cm, all other scale bars = cm.

Coronoid

258. Splenial, acute posterior process separating angular and coronoid: present (0); absent (1) (after Brochu, 1997a [59]).

(Brochu, 1999) recognised the presence of a 'V' shaped process of the splenial between the angular and coronoid, which is present in extant crocodylids, *Gavialis gangeticus*, and *Tomistoma schlegelii* (Fig. 116A–B), but absent in most alligatorines, (e.g. *Alligator mississippiensis* [Fig. 116F] and *Alligator mcgrewi* [Fig. 116C]), and all extant caimanines, e.g. *Caiman latirostris* (Fig. 116G). Here this process is recognised in a few alligatorines, including *Alligator sinensis* (USNM 292078; Cong et al., 1998: fig.47C) and *Alligator prenasalis* (YPM PU 14063), which were previously scored as absent and unknown for this feature, respectively. The condition is unknown in the outgroup, but at least one non-crocodylian eusuchian, *Agaresuchus fontisensis*, appears to exhibit the process (Narváez et al., 2016, fig.4C), suggesting this is the plesiomorphic condition in Crocodylia.

259. Foramen intermandibularis medius (FIM), anteroposterior length relative to foramen intermandibularis caudalis (FIC): short, less than 25% FIC length (0); long, equal to or greater than 25% FIC length (1) (new character, based on personal observations).

In most eusuchians, the FIM is very small, perforating the coronoid or the splenial-coronoid suture (see Character 260). Uniquely in *Crocodylus acutus* and *Crocodylus intermedius*, this foramen is highly enlarged, such that it is greater than 25% the anteroposterior length of the FIC (Fig. 116D). The same condition is also present (and scored as such) in *Stangerochampsa mccabei*, although in this species this appears to be a result of reduction in size of the FIC, rather than enlargement of the FIM (Wu et al., 1996, fig.2B).

260. Coronoid, position of foramen intermandibularis medius (FIM) (at maturity): on coronoid-splenial suture (0); entirely within coronoid (1) (after Norell, 1988 [12]; Brochu, 1997a [46]).

Where preserved, the FIM is positioned on the anterior sutural contact between the coronoid and splenial in most eusuchians (Fig. 116A–B, D–F). By contrast, this foramen is completely situated within the coronoid in extant *Caiman*, *Melanosuchus* (Fig. 116G–H) and *Purussaurus neivensis* (USNM 10889). Brochu (1999) considered the FIM to be lost at maturity in both *Paleosuchus* species, which was captured in an additional character state (46-2 therein). Nevertheless, he noted that a foramen does occur on the coronoid, but because its position and form were considered different to the FIM, it was tentatively treated as an independent structure. Accordingly, the presence or absence of this foramen was characterised in a separate character (Brochu, 1999: [56]). If the coronoid foramen of *Paleosuchus* is not the FIM, one would expect to see two foramina at some

point in ontogeny. Hatchling *Paleosuchus* specimens were not available for study here, but in two juvenile specimens (AMNH 93812, 66391) only one foramen occurs on the coronoid. Furthermore, the position of the foramen in these specimens (and indeed that figured in a more mature specimen by Brochu [1999: fig.59]) does not appear notably different to the FIM of other caimanines (Fig. 116I). These observations do not disprove the independence of these foramina; however, this could be tested by comparing a series of *Paleosuchus* specimens of different ontogenetic stages. Until then, the simplest explanation is that the perforation of the coronoid in *Paleosuchus* is the FIM at all ontogenetic stages, and it is treated as such here. As a result, we do not include Character 56 of Brochu (1999).

261. Coronoid, anterior extent relative to level of anterior margin of foramen intermandibularis caudalis (FIC): anterior (0); at the same level or posterior (1) (after Jouve et al., 2015 [228]; Lee and Yates, 2018 [194]).

This condition is difficult to assess in most fossil taxa because of poor preservation of the coronoid. The coronoid is positioned posterior to the level of the FIC (261-1) in all extant crocodylids, *Voay* (NHMUK R36686), *Mekosuchus* (QM F31053, MNHN NCP 06), and *Melanosuchus niger* (Fig. 116H). By contrast, the coronoid is at the same level or anterior to the FIC in *Lohuecosuchus* (Narváez et al., 2015, fig.4D), *Agaresuchus* (Narváez et al., 2016, fig.4D), most extant alligatorids (Fig. 116C, F, G), *Gavialis gangeticus* (Fig. 116A), *Maomingosuchus petrolica* (Shan et al., 2017, fig.7C), and *Tomistoma schlegelii* (Fig. 116B).

262. Coronoid, orientation of dorsal profile: inclined anteriorly across entire length (0); horizontal towards posterior end (1) (after Brochu, 1997a [54]).

The coronoid has two posteriorly directed processes, one dorsal and one ventral (Brochu, 1999). In all eusuchians (where known), the anterodorsal edge of the dorsal process is inclined anteriorly (Fig. 116). However, differences occur in the posterior extent of the dorsal process, as well as its orientation. Commonly, the dorsal process has a long posterior extension, which tends to level off to become horizontal (262-1). In this case, the dorsal process almost reaches the level of the posterior extent of the ventral process. This condition occurs in all extant crocodylids and *Alligator* (Fig. 116C–F). By contrast, the dorsal process is anteroposteriorly shorter in some caimanines, and the dorsal profile is inclined across its entire length (262-0) (Fig. 116G). Both *Gavialis gangeticus* and *Tomistoma schlegelii* have distinct morphologies that do not fit easily into either state. In *Gavialis gangeticus*, the dorsal process is strongly truncated posteriorly, such that it can appear absent altogether (Fig. 116A). In *Tomistoma schlegelii* there is some truncation of the dorsal process, but it levels off at its posteriormost extent (Fig. 116B) similar to crocodylids. No other taxa in this dataset share these conditions, rendering a new character or character states uninformative. Since

the condition in these taxa is more reminiscent of caimanines, they are provisionally scored with the plesiomorphic condition, following previous authors (e.g. Brochu, 1999).

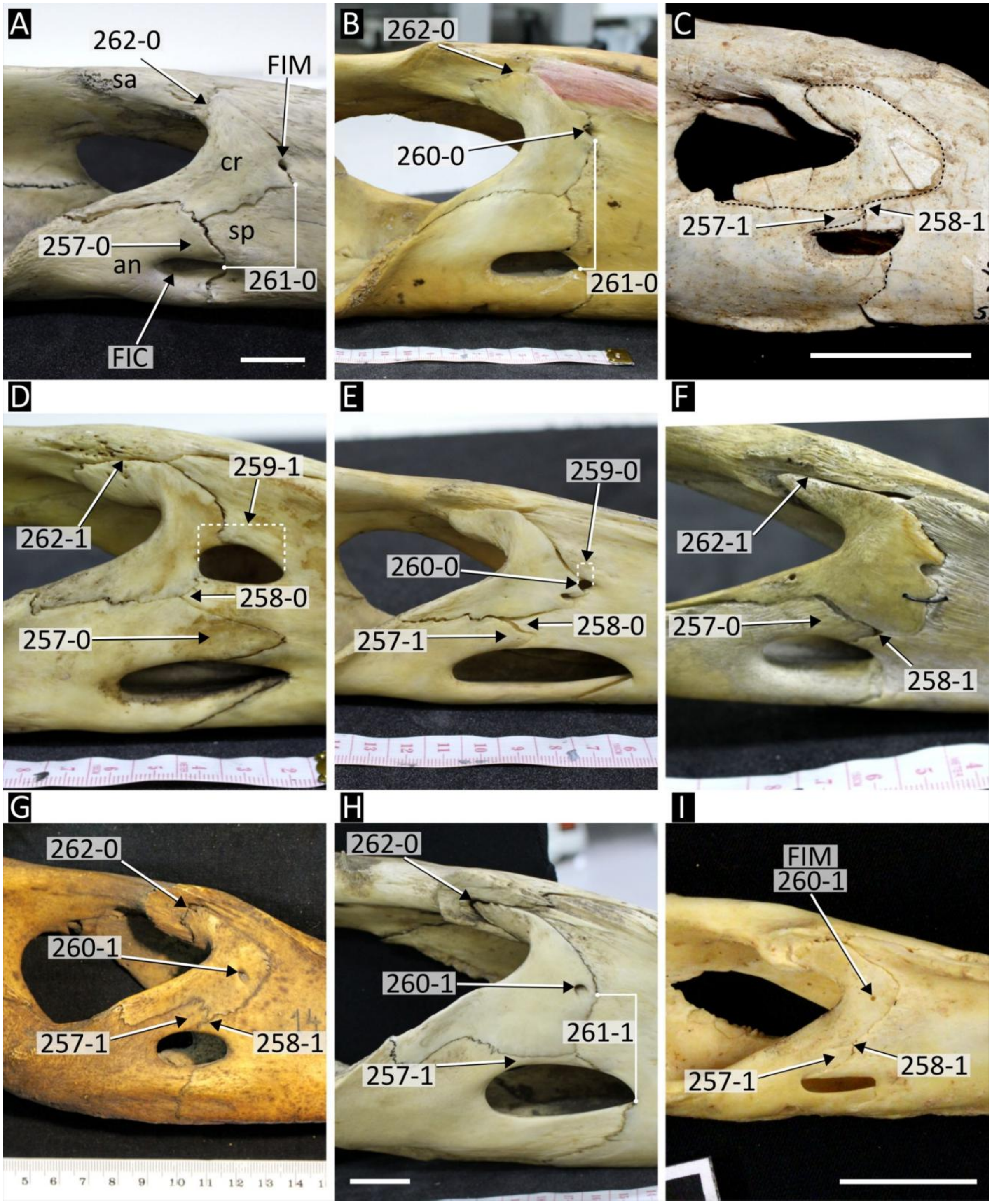

Figure 116: Medial view of the posterior mandibular ramus. **A**, *Gavialis gangeticus* (NHMUK uncatalogued); **B**, *Tomistoma schlegelii* (NHMUK 1894.2.21.1); **C**, *Alligator mcgrewi* (AMNH FAM 8700), **D**, *Crocodylus intermedius* (NHMUK 1851.8.25.29); **E**, *Mecistops cataphractus* (62.6.30.8); **F**, *Alligator sinensis* (NHMUK X184); **G**, *Caiman latirostris* (MACN V 1420); **H**, *Melanosuchus niger* (45.8.25.125); **I**, *Paleosuchus palpebrosus* (AMNH 93812). Abbreviations: **an**, angular; **cr**, coronoid; **FIC**, foramen intermandibularis caudalis; **FIM**, foramen intermandibularis medius; **sp**, splenial; **sa**, surangular. Scale bars A, C, H, I = 2 cm, all other scale bars = cm.

263. Coronoid, prominent medioventral lamina extending over inner (medial) surface of Meckelian fossa: present (0); absent (1) (after Brochu, 1997a [55]).

The anatomical meaning of this character follows that described and illustrated by Brochu (1999: fig.47D–F). In most eusuchians (where known), the coronoid has a ventral process that laps over the inner surface of the Meckelian fossa (63-0). Among extant crocodylians, this occurs in crocodylids and *Alligator*, but not in caimanines (263-1).

Axial column

Cervical vertebrae

264. Proatlas, acute anterior process: present, anterolateral margin of proatlas prominently concave (0); absent, anterior margin of proatlas straight or convex (1) (after Brochu, 1997a [10]).

The proatlas is one of the most poorly preserved elements of the skeleton in Eusuchia; indeed, it was not even possible to examine it in all extant crocodylians for this study. Nevertheless, based on the sample of crocodylians examined, inconsistencies were observed in exisitng characters delimiting the proatlas morphology. Brochu (1999) and all subsequent iterations of this dataset have discretised the morphology of the proatlas into two characters. The first (Brochu, 1997a:[2]) describes the overall morphology as either 'boomerang'-shaped (0), 'strap'-shaped (1), or massive and 'block'-shaped (2). The second (Brochu, 1997a:[10]) described the presence or absence of an anterior process, which was argued as being independent of the first character. The distinction between taxa scored for each of the states of the first of these characters in earlier studies is not always apparent, nor was the proatlas morphology consistent within taxa scored for the same state, as also noted by Sookias (2020). For example, whereas *Crocodylus rhombifer* (Fig. 117G) and *Crocodylus acutus* (Fig. 117H) are scored as having boomerang-shaped proatlases, *Crocodylus porosus* (Fig. 117D) is scored as having a strap-shaped proatlas (Brochu, 2007). However, these taxa do not appear notably different. By contrast, the proatlases of taxa such as *Alligator* (Fig. 117B–C) and *Diplocynodon* (Fig. 117A), which are also scored for the boomerang-shaped condition, appear completely different to those *Crocodylus* species. This is principally due to a prominent anterior process in these taxa. Furthermore, although the proatlases of *Tomistoma schlegelii* (Fig. 117E) and *Gavialis gangeticus* (Fig. 117F) match their description of being "massive and block-shaped" (Brochu, 1997a), the distinction between this and the condition of several *Crocodylus* species is very subtle. Based on these observations, Character 2 of Brochu (1997b) is exlcuded here, and the morphology of the proatlas is characterised only by the presence or absence of a prominent anterior process. The definition of a process can be subjective, as all proatlases taper anteriorly to a degree.

Here, an anterior process is considered present when the anterolateral margins of the proatlas are concave. This is most prominently expressed in *Diplocynodon* (Fig. 117A) and *Paleosuchus*, but it also occurs in *Alligator mississippiensis* (Fig. 117C). By contrast, *Gavialis* (Fig. 118F), *Tomistoma* (Fig. 117E), and all *Crocodylus* species (Fig. 117G–H) examined here, are considered to lack this process. Among fossil crocodylians, *Borealosuchus formidabilis* (Erickson, 1976, fig.14) and *Asiatosuchus germanicus* (HLMD Me 3092) also lack the anterior process.

265. Proatlas, dorsal keel: present (0); absent (1) (after Brochu, 1997a [17]).

(Brochu, 1999) noted that the proatlas of most crocodylians exhibit either a low dorsal midline keel, or lack a keel altogether. By contrast, the keel is very prominent in some crocodylians, e.g. *Gavialis gangeticus* (Fig. 117F), *Diplocynodon* (Fig. 117A), and *Brachychampsa* (UCMP 133901). (Brochu, 1997b) used a binary state character in which only a prominent keel was considered as 'present'. Here, the presence of a midline keel is recognised regardless of size. Consequently, many more taxa are scored for the plesiomorphic state than in the dataset of Brochu (1999), including several *Crocodylus* species. An examination of later iterations of that dataset (e.g. Brochu, 2007a) reveal a similar basis for character state delimitation was used, with most *Crocodylus* species scored as possessing a dorsal keel.

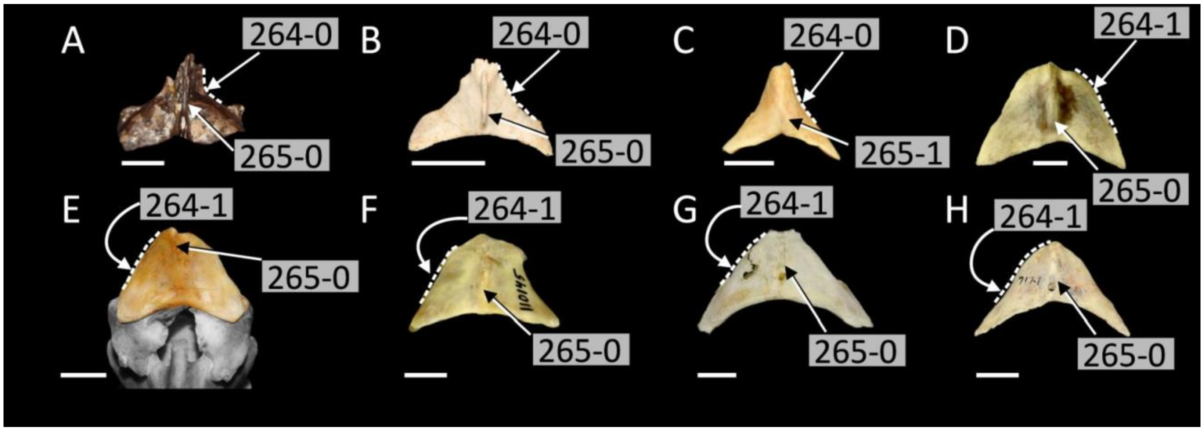

Figure 117: Morphology of the proatlas. **A**, *Diplocynodon hantoniensis* (NHMUK OR 30289); **B**, *Alligator mcgrewi* (AMNH FAM 8700); **C**, *Alligator mississippiensis* (AMNH 71621); **D**, *Crocodylus porosus* (NHMUK uncatalogued); **E**, *Tomistoma schlegelii* (AMNH 113078); **F**, *Gavialis gangeticus* (AMNH 110145); **G**, *Crocodylus rhombifer* (AMNH R154087); **H**, *Crocodylus acutus* (AMNH 7121). All scale bars = 1 cm.

266. Atlas intercentrum, shape in lateral view: wedge-shaped (0); plate-shaped (1) (after Clark, 1994 [89]; Brochu, 1997a [5]).

The presence of a flattened, plate-shaped atlantal intercentrum (Fig. 118A–B) has consistently been recovered as an unambiguous synapomorphy of Globidonta (e.g. Brochu, 1999), i.e. crown group Alligatoridae and a few stemward alligatoroid taxa, e.g. *Brachychampsa montana* (UCMP

133901). By contrast, 'basal' alligatoroids such as *Diplocynodon* (all species, where known) have a distinctive, wedge-shaped atlantal intercentrum (Fig. 118D–E) as is the case in most non-alligatoroid crocodylians. This includes *Gavialis gangeticus* (Fig. 118E), *Tomistoma schlegelii* (AMNH 113078), and all extant crocodylids (Fig. 118A). The same condition has been noted in all members of *Borealosuchus* (Brochu et al. 2012), e.g. *B. formidabilis* (Erickson, 1976, fig.13B) (Erickson, 1976: fig.13B). By contrast, the alligatorid condition is newly recognised in *Borealosuchus sternbergii* (UCMP 134470, Fig. 118C).

267. Atlantal rib, dorsal margin shape: straight, or with modest process (0); with prominent process (1) (Brochu, 1997a [14]).

Brochu (1999) identified a prominent dorsal process on the atlantal ribs of most extant alligatorids, *Brachychampsa*, and *Toyotamaphimeia* (Fig. 118H), contrasting with *Gavialis gangeticus*, *Tomistoma schlegelii*, most extant crocodylids and *Borealosuchus* (Fig. 118F). The anatomical meaning and distribution of this feature is consistent with earlier studies (e.g. Brochu, 1999; Brochu et al., 2012).

268. Atlantal rib, thin medial lamina at proximal end: absent (0); present (1) (after Brochu, 1997a [16]).

269. Atlantal rib, proximal articular facet for opposing atlantal rib: absent (0); present (1) (Brochu, 1997a [15]).

Characters 268 and 269 describe two similar, but independent processes that occur on the anteromedial end of the atlantal rib. Character 268 describes a medial lamina that serves as the attachment point of the atlantodental ligament, which connects the paired atlantal ribs (Fig. 118H) (Brochu, 1999). According to Brochu (1999), and as scored therein, among extant crocodylians this process only occurs in caimanines. Indeed, this process is observed in *Caiman* (e.g. AMNH 97300), *Paleosuchus* (e.g. AMNH 66391) and *Melanosuchus niger* (AMNH 97325). Nevertheless, the only caimanine scored for this condition in all subsequent iterations of the dataset of Brochu (1999) is *Paleosuchus* (e.g. Brochu, 2011; Brochu et al. 2012; Salas-Gismondi et al., 2015; Cidade et al., 2017). This seems likely to be a typographical error that has been carried forward, since the figures in Brochu (1999, fig.28C), clearly show that these medial laminae are present in all extant caimanines. Scores are further modified here, as this process is also recognised on the atlantal ribs of *Alligator mississippiensis* (Fig. 118H), *Brachychampsa montana* (UCMP 133901), and *Borealosuchus sternbergii* (UCMP 134470). Character 269 describes the development of anteroposteriorly long atlantal articular facets, a condition exclusively known in *Paleosuchus* (Brochu, 1999) (Fig. 118I).

270. Odontoid process: mediolateral width across axial rib facets, relative to mediolateral width across

axial tubercula facets: narrower (0); subequal (1) (after Ijima and Kobayashi, 2019 [244]).

Iijima and Kobayashi (2019, fig.S1) illustrated differences in morphology of the odontoid process among crocodylians, noting that the mediolateral width across the ventral facets for the axial rib capitula is notably narrower than that across the dorsal facets for the axial rib tubercula in *Bernissartia fagesii* and all extant alligatorids (Fig. 118J). By contrast, the facets are subequal in width in all extant crocodylids (except *Osteolaemus*), *Gavialis gangeticus*, and *Tomistoma schlegelii* (Fig. 118K). The plesiomorphic condition is recognised in some additional alligatorids including *Alligator mcgrewi* and *Purussaurus neivensis*. By contrast, the 'basal' alligatoroid *Diplocynodon* exhibits the derived condition, e.g. *D. hantoniensis*, and *D. darwini* (Ludwig, 1877, plate 3, 13d).

271. Axial rib, tuberculum shape: short and broad, equal in size to capitulum (0); long and acute, narrower than capitulum (1) (after Brochu, 1997a [20]).

Following Brochu (1997b), two distinctive morphologies of the axial rib tuberculum can be observed in crocodylians (Fig. 118L–R); however, the taxa assigned to each state in this study contrasts with scores in earlier studies (e.g. Brochu, 1999; Brochu et al., 2012; Salas-Gismondi et al., 2015). Whereas the scores herein concur for *Gavialis gangeticus*, in which the axial rib tuberculum is short and broad, approximately equal in dimensions to the capitulum (Fig. 118M), they differ in that we also regard this condition as characterising *Crocodylus* (e.g. *C. acutus* [Fig. 118O], *C. rhombifer* [Fig. 118L], *C. porosus* [Fig. 118Q]), *Tomistoma schlegelii* (AMNH 113078), and *Osteolaemus tetraspis* (AMNH 69057). In the derived character state, the proximal end of the axial rib forms a broad capitulum and a long, narrower tuberculum. This condition is found in all extant alligatorids (e.g. *Alligator mississippiensis* [Fig. 118N] and *Caiman yacare* [Fig. 118P]) as also scored in previous analyses.

272. Axial rib, tuberculum, contact with axial diapophysis: absent, or occurs late in ontogeny (0); present early in ontogeny (1) (Brochu, 1997a [21]).

The definition and scoring of this character is consistent with earlier studies. As described and illustrated by Brochu (1999, fig.30), contact between the axial rib tuberculum and the axial diapophysis can be observed in all extant alligatorids at an early ontogenetic stage (Fig. 119C), but is absent in crocodylids, *Gavialis gangeticus* (AMNH 110145), *Tomistoma schlegelii* (AMNH 113078), *Borealosuchus formidabilis* (Erickson, 1976) and *Brachychampsa montana* (Fig. 119B). For a taxon to be scored for the derived character state, a juvenile specimen is ideally required. However, contact between the axial rib and diapophysis was observed in (probably) mature specimens of two fossil taxa: *Mourasuchus arendsi* (Cidade et al., 2018, fig.8A) and *Purussaurus neivensis* (UCMP 39657). These taxa are scored for the derived condition here pending new data on juvenile individuals that demonstrates such contact was absent early in ontogeny.

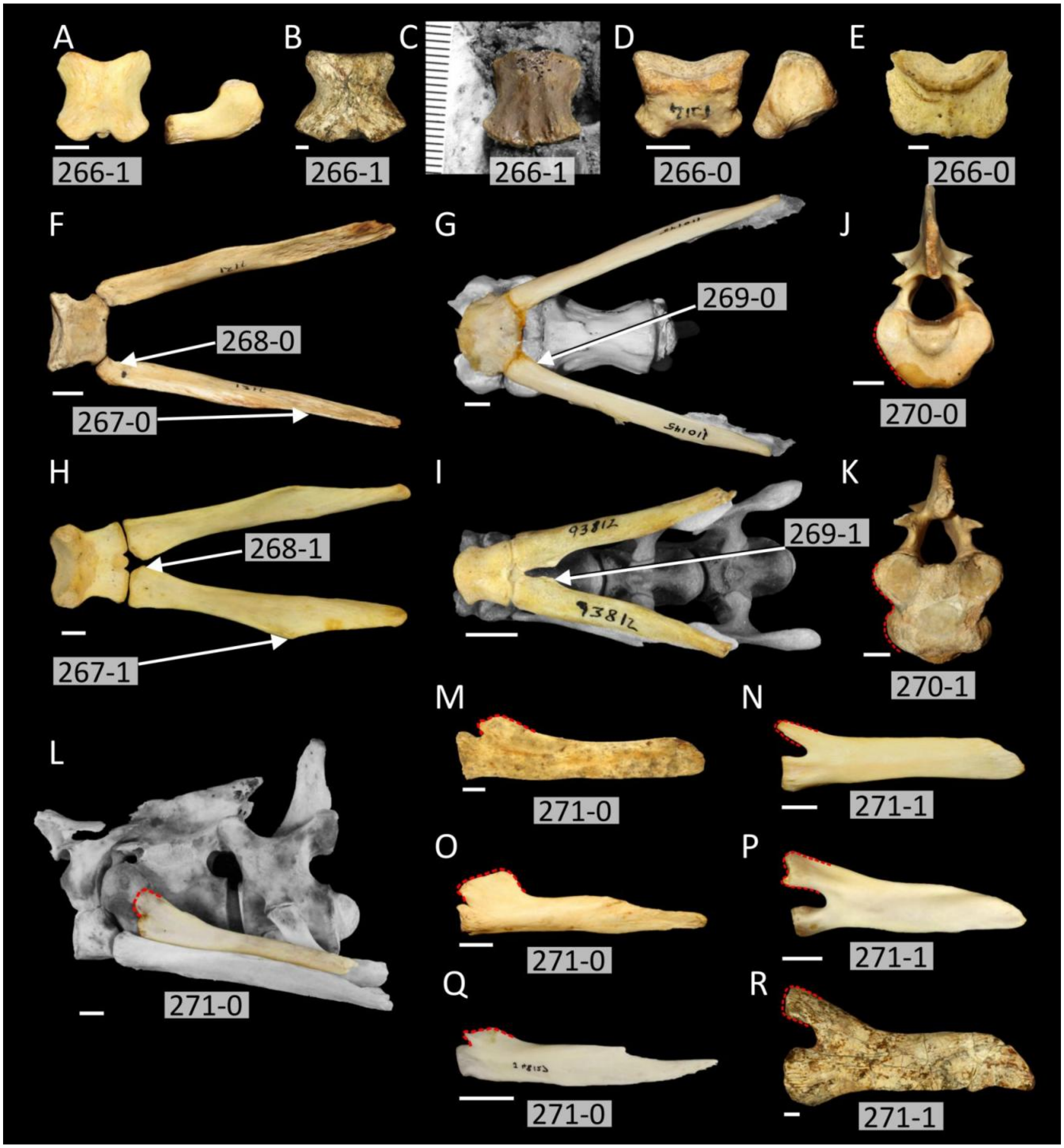

Figure 118: Morphology of the atlas-axis complex. **A**–**E**, atlantal intercentrum (all in ventral view except A and D, which include right lateral views): **A**, *Alligator mississippiensis* (AMNH 71621); **B**, *Purussaurus neivensis* (UCMP 39657); **C**, *Borealosuchus sternbergii* (UCMP 134470); **D**, *Crocodylus acutus* (AMNH 7121); **E**, *Gavialis gangeticus* (UMZC R 5783); **F**–**I**, atlantal ribs of: **G**, *Crocodylus acutus* (AMNH 7121, dorsal view); **G**, *Gavialis gangeticus* (AMNH 110145, ventral view); **H**, *Alligator mississippiensis* (AMNH 71621, dorsal view); **I**, *Paleosuchus palpebrosus* (AMNH 93812, ventral view); **J**–**K**, odontoid process in anterior view: **J**, *Caiman yacare* (AMNH 97300); **K**, *Crocodylus acutus* (AMNH 7121); **L**, atlas-axis complex of *Crocodylus rhombifer*, left lateral view highlighting the axial rib (AMNH R154087); **M**–**R**, lateral view of the left axial rib: **M**, *Gavialis gangeticus* (UMZC R 5783); **N**, *Alligator mississippiensis* (AMNH 71621); **O**, *Crocodylus acutus* (AMNH 7121); **P**, *Caiman yacare* (AMNH 97300); **Q**, *Crocodylus porosus* (QM J48127, digitally reversed); **R**, *Purussaurus neivensis* (UCMP 39657). All scale bars = 1 cm.

273. Axis, neural spine, anterior half of dorsal margin in lateral view (at maturity): horizontal (0); slopes such that it faces anterodorsally (1) (after Brochu, 1997a [11]).

274. Axis, neural spine, posterior half: dorsally inflected to form crest (0); continuous with anterior half, not crested (1) (after Brochu, 1997a [12]).

275. Axis, neural spine, shape of distal end: dorsoventrally thick (0); dorsoventrally thin, rod-like (1) (after Brochu, 1997a [3]).

Characters 273–275 describe subtle differences across regions of the axial neural spine. These characters are respectively based on characters 11, 12, and 3 in Brochu (1997a), all of which were not considered robust by Sookias (2020). Inconsistencies in the character scores of some taxa were similarly observed here; however, these characters are retained, with modifications to wording and character scores. Character 273 describes the orientation of the anterior half of the dorsal margin of the the axial neural spine. According to Brochu (1999), the anterior half is horizontal early in ontogeny in all extant crocodylians, but becomes anteriorly inclined at maturity in some taxa, e.g. *Caiman* and *Melanosuchus* (Fig. 119C). This broadly matches observations made in this study. A sloping anterior half of the neural spine characterises all extant jacareans, *Borealosuchus*, *Boverisuchus vorax*, and *Alligator mcgrewi* (AMNH FAM 8700). This condition contrasts with that exhibited by *Diplocynodon* (Fig. 119A) and *Gavialis gangeticus* (Fig. 119F), in which the anterior half of the spine is horizontal. The distinction between anterior and posterior portions of the axial neural spine appears to be significant, as regardless of the orientation of the anterior half, the posterior half can take on a range of morphologies. These are captured in characters 274 and 275. Character 274 was originally described in terms of the presence or absence of a posterior 'crest'. Based on character scores in Brochu et al. (2012), a crest occurs in all extant *Caiman* species, extant crocodylids, *Tomistoma schlegelii*, and *Gavialis gangeticus*. The wording of the original character is ambiguous since the posterior tip of the axial neural spine could be considered crest-like in most crocodylians. Herein, the crested condition only applies to taxa with a concavity in the dorsal outline of the neural spine, such that spine is dorsally inflected posteriorly. This condition is exemplified by *Purussaurus neivensis* (Fig. 119C), *Brachychampsa montana* (Fig. 119B), contrasting with the uncrested neural spines of *Diplocynodon hantoniensis* (Fig. 119A) and *Caiman* (contra Brochu, 1999) (Fig. 119C). Character 275 originally described the posterior half of the axial neural spine as either "wide" or "narrow" (Brochu, 1997b, character 3). This wording was considered ambiguous, as it is not clear whether this neural spine dimension should be considered as mediolateral or dorsoventral. Here, the derived character state applies to taxa in which the distal end of the neural spine forms a dorsoventrally narrow, rod-like process. This is distinct from the dorsally inflected (crested) condition described in Character 274. The rod-like condition (275-1)

is exemplified by *Gavialis gangeticus* (Fig. 119F), *Eosuchus minor* (Fig. 119G) and most extant crocodylids, e.g. *C. rhombifer* (Fig. 118T).

276. Axis, lateral process (diapophysis) on neural arch lateral margin: absent (0); present (1) (after Norell, 1989 [7]; Brochu, 1997a [4]).

Among extant crocodylians, *Gavialis gangeticus* is the only species with an axial diapophysis (Baur, 1886; Norell, 1989) (Fig. 119F, J). The co-occurrence of this diapophysis in the non-crocodylian neosuchian, *Bernissartia fagesii*, was considered as evidence of the sister relationship of *Gavialis gangeticus* to all other crocodylians (Norell & Clark, 1990; Norell, 1989). Where observed in *Gavialis gangeticus*, this process tends to be a very low, anteroposteriorly orientated crest, positioned dorsal to the neurocentral suture (Fig. 119J). Among fossil crocodylians, an axial diapophysis has traditionally only been recognised in "gavialoids", e.g. *Eosuchus* (Fig. 119K–L) and *Thoracosaurus* (Brochu et al., 2012, character scores therein). However, the diapophysis is also present in at least two "tomistomines": *Toyotamaphimeia* and *Penghusuchus* (Iijima & Kobayashi, 2019).

277. Axis, hypapophysis position: located towards centre of centrum (0); toward anterior end of centrum (1) (Brochu, 1997a [6]).

In most crocodylians, the axial hypapophysis is located towards the anterior end of the centrum (Fig. 119E). Brochu (1999) recognised a posteriorly shifted hypapophysis exclusively in *Diplocynodon*, such that it occurs around the anteroposterior mid-length of the centrum (Fig. 119A). This is similarly recognised here, but several additional crocodylians exhibit the derived condition, e.g. *Caiman yacare* (AMNH 97300), *Alligator sinensis* (USNM 292078), *Alligator mcgrewi* (AMNH FAM 8700), and *Borealosuchus formidabilis* (Erickson, 1976, fig.13B).

278. Axis, hypapophysis shape: un-forked (0); forked (1) (after Brochu, 1997a [19]).

Uniquely among extant crocodylians, *Gavialis gangeticus* exhibits a forked axial hypapophysis (Fig. 119N), a condition shared by several fossil "gavialoids", e.g. *Thoracosaurus* (Brochu, 2004a) and *Eosochus minor* (USNM 181577) (but not *Eosuchus lerichei* [IRScNB R49]) and some "tomistomines", e.g. *Toyotamaphimeia* and *Penghusuchus* (Iijima & Kobayashi, 2019). By contrast, *Bernissartia fagesii* and all other crocodylians exhibit a single, un-forked hypapophysis (Fig. 119M).

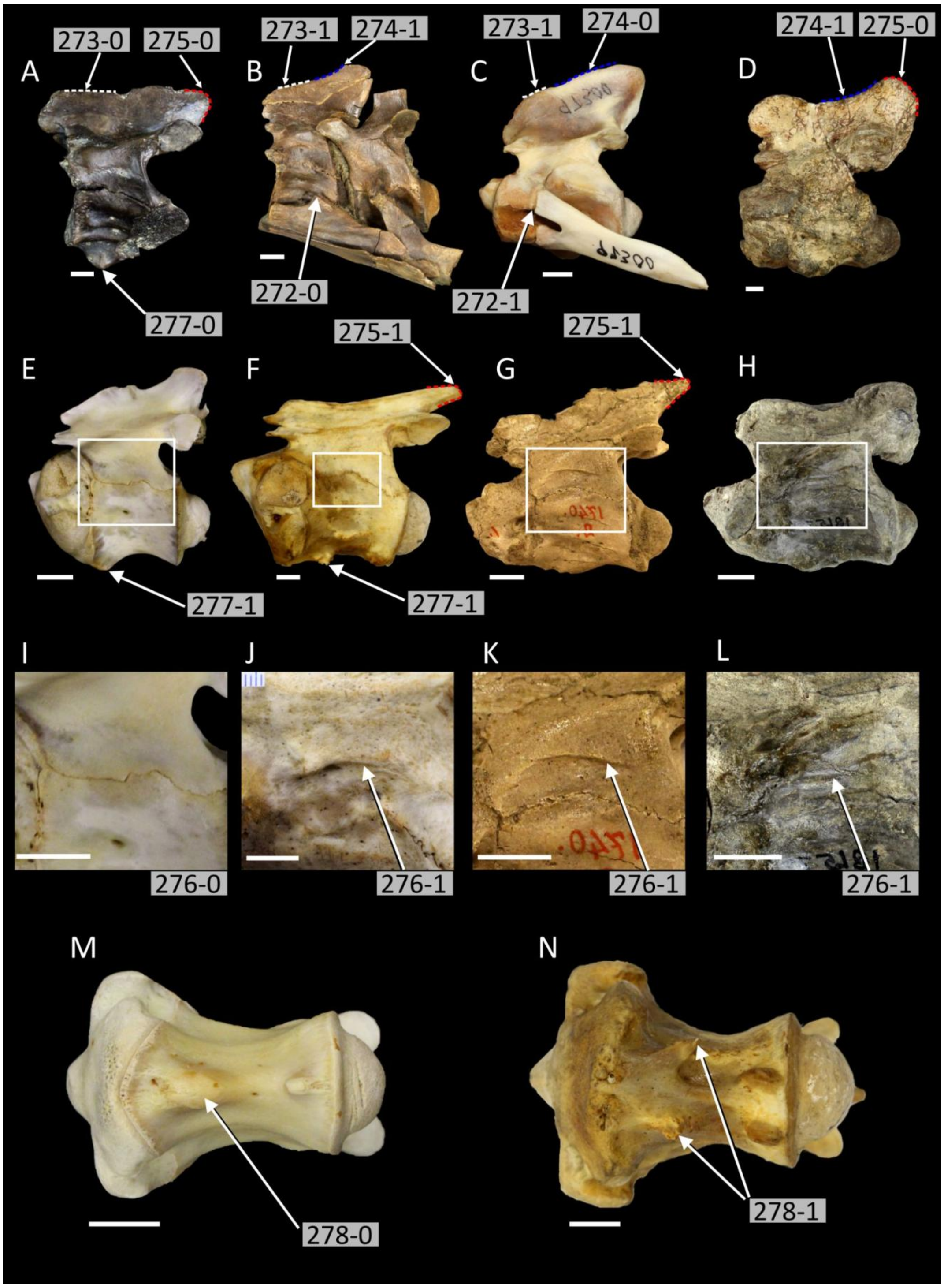

Figure 119: Morphology of the axis in selected crocodylian taxa. **A**–**H**, left lateral view of the axis in: **A**, *Diplocynodon hantoniensis* (NHMUK uncatalogued, digitally reversed); **B**, *Brachychampsa montana* (UCMP 133901); **C**, *Caiman yacare* (AMNH 97300, digitally reversed); **D**, *Purussaurus neivensis* (UCMP 39657, digitally reversed); **E**, *Crocodylus porosus* (QM J48127); **F**, *Gavialis gangeticus* (UMZC R 5783); **G**, *Eosuchus lerichei* (IRScNB R 1740, digitally reversed); **H**, *Eosuchus minor* (USNM 181577, digitally reversed); **I**–**L** enlargement of the regions highlighted in **E**–**H** respectively; **M**, *Crocodylus johnstoni*, ventral view (QM J58446); **N**, *Gavialis gangeticus*, ventral view (UMZC R 5783). All scale bars = 1 cm.

279. Prominent cervical hypapophyses: present (0); absent (1) (after Norell, 1989 [12]; Norell and Clark, 1990 [11]; Clark, 1994 [92]; Brochu, 1997a [8]).

Norell (1989) noted that *Gavialis gangeticus* lacks hypapophyses on its cervical vertebrae, unlike all other extant crocodylians, which have prominent cervical hypapophyses. The absence of prominent cervical hypapophyses is recognised in *Bernissartia fagesii* (Norell & Clark, 1990), *Borealosuchus* (Brochu et al., 2012), some "gavialoids" e.g. (*Eosuchus minor* [Fig. 120B] and *Thoracosaurus*), and also in the "tomistomine" *Toyotamaphimeia machikanensis* (Iijima & Kobayashi, 2019).

280. First postaxial vertebra (Cv3), anteroposterior length at the distal end of the neural spine: long, greater than or equal to half the length of the non-condylar centrum (0); short, dorsal tip acute and less than half the length of the non-condylar centrum (1) (after Brochu, 1997a [9]).

The neural spine of the first postaxial vertebra (third cervical vertebrae) in *Bernissartia fagesii* (IRScNB 1538), *Borealosuchus*, and some caimanines is anteroposteriorly long and often square shaped (280-0) (Fig. 120C). By contrast the neural spine is anteroposteriorly short (280-1) and often acute in most extant crocodylids (Fig. 120A), *Gavialis gangeticus* (Fig. 120G), and "tomistomines" (e.g. *Tomistoma schlegelii* [AMNH 113078], *Toyotamaphimeia*, and *Penghusuchus* [Iijima and Kobayashi, 2019]).

281. Cervical centra: amphicoelous (both articular surfaces concave) (0); procoelous (anterior articular surface concave, posterior articular surface convex) (1) (after Norell and Clark, 1990 [8]; Clark, 1994 [92]; Brochu, 1997 [18]; Brochu et al., 2012 [21]).

As noted by Norell and Clark (1990), the cervical centra are plesiomorphically amphicoelous in neosuchians (Fig. 120D), e.g. *Bernissartia fagesii* (IRScNB 1538), contrasting with the procoelous cervical centra of eusuchians (Fig. 120C). The morphology of the dorsal and caudal centra is discretised separately (Characters 285 and 292) as both can vary independently of that of the cervical vertebrae.

282. Posterior cervical vertebrae (C7–C9), anterior extent of hypapophyses (C7–C9): level with, or anterior to the level of anterior margin of the prezygapophyses (0); posterior to the level of anterior margin of prezygapophyses (1) (after Iijima and Kobayashi, 2019 [246]).

Iijima and Kobayashi (2019, character 246) note that the posterior cervical hypapophyses of all extant alligatorids project anteroventrally "well beyond" the level of the centrum (Fig. 120F). In all eusuchians examined here, the posterior cervical hypapophyses are anteriorly hooked to a degree, and can still extend towards and slightly beyond the centrum margin, e.g. *Crocodylus acutus* (Fig. 120E), *Gavialis gangeticus* (Fig. 120G); nevertheless, they never reach the same anterior extent

as in alligatorids. To capture this difference, here the anterior hypapophyseal extent is measured relative to the anterior extent of the prezygapophyseal facets. These provide a more anteriorly positioned landmark for measuring hypapophyseal extent, removing the need for subjective terminology such as “well-beyond”. In addition to extant alligatorids, strongly anteroventrally directed hypapophyses are newly recognised in *Alligator mcgrewi* (Fig. 120H) and *Purussaurus neivensis* (UCMP 39657).

283. Cervical rib 8, length in proportion to cervical rib 9: long, greater than half the length of cervical rib 9 (0); short, equal to or less than half the length of cervical rib 9 (1) (after Iijima and Kobayashi, 2019 [247]).

Mook (1921) noted that the 8th cervical rib of extant *Crocodylus* and *Tomistoma schlegelii* is elongate, being similar in morphology to the 9th cervical rib (described as the first dorsal rib therein) (Fig. 121A–B). By contrast, he noted that the 8th cervical rib is shorter in *Alligator* (Fig. 121E) and *Caiman* (Fig. 121F). As later demonstrated by Iijima and Kobayashi (2019, fig.S2), *Gavialis gangeticus* also exhibits an elongated 8th cervical rib, like crocodylids (Fig. 121C), whereas all extant alligatorids exhibit a shortened rib. This character does not necessarily require the preservation of the 9th rib as, in both plesiomorphic and derived conditions, the morphology of the 8th rib is distinct from any other rib. The shortened 8th cervical rib of alligatorids can be distinguished from any of the preceding cervical ribs, in that the capitular and tubercular processes are parallel to the shaft of the rib (rather than perpendicular). Furthermore, the shaft is only slightly longer than the proximal articular processes, unlike any subsequent presacral ribs. Isolated 8th cervical ribs in crocodylids, *Tomistoma* and *Gavialis* are very similar in appearance to the 9th and subsequent dorsal ribs, yet they lack the prominent medial curvature of the shaft. The morphology of the 8th cervical rib is poorly known in fossil crocodylians. For example Iijima and Kobayashi (2019) could only score this character in *Borealosuchus formidabilis* and *Toyotamaphimeia* (283-0 in both). The derived, short condition is additionally recognised in several fossil alligatoroids including *Alligator mcgrewi* (Fig. 121H), *Purussaurus neivensis* (Fig. 121I), and *Diplocynodon darwini* (Fig. 121J). By contrast, *Borealosuchus sternbergii* (UCMP 134470) and ‘*Crocodylus*’ *affinis* (Fig. 121D) exhibit an elongate rib.

284. Hypapophyseal keels, posterior retention: until tenth postatlantal vertebra (Dv2) (0); eleventh postatlantal vertebra (Dv3) (1); twelfth postatlantal vertebra (Dv4) (2) (after Brochu, 1997a [7]) (ORDERED).

This character requires the consecutive preservation of the anteriormost dorsal vertebrae, and so is poorly known in fossil crocodylians. In *Bernissartia fagesii* (IRScNB 1538) (and no other taxon in this dataset), hypapophyses occur up to and including the 10th postatlantal vertebra (dorsal verte-

bra 2) (284-0). In most extant crocodylians, hypapophyses occur up to the 11th postatlantal vertebra (dorsal vertebra 3) (284-1), e.g. *Alligator mississippiensis* (AMNH 71621), Jacarea, *Gavialis gangeticus* (UCMZ R5783), and *Tomistoma schlegelii* (AMNH 113078). In fewer taxa, the hypapophyses occur further posteriorly, up to the 12th postatlantal vertebra (284-2), e.g. *Paleosuchus* (AMNH 97326), *Alligator sinensis* (USNM 292078) and *Diplocynodon hantoniensis* (Rio et al., 2020). Character states are re-organised relative to earlier studies as the character is newly ordered.

Dorsal vertebrae

285. Dorsal centra: amphicoelous (0); procoelous (1) (after Norell and Clark, 1990 [10]; Clark, 1994 [93]).

Where preserved procoelous dorsal centra occur in all taxa in this dataset, except *Bernissartia fagesii* (IRScNB 1538) and *Theriosuchus pusillus* (e.g. NHMUK 48216; see also Tennant et al., 2016:p.914), which exhibit amphicoelous dorsal centra.

286. Dorsal vertebrae, maximum mediolateral width across both transverse processes at vertebrae 7––10: equal to or greater than twice the equivalent width on DV1 (0); less than twice the equivalent width on DV1 (1) (after Iijima and Kobayashi, 2019 [248]).

287. Dorsal vertebrae, fusion of the diapophysis and parapophysis, occurrence: anterior to or on the 12th dorsal vertebra (0); on the 13th dorsal vertebra (1) (after Iijima and Kobayashi, 2019 [249]).

Characters 286–287 were based on the observations of Iijima and Kubo (2019b) who recognised two currently autapomorphic features of *Gavialis gangeticus*. The first is that the maximum width across the transverse processes (usually around dorsal vertebrae 7–10) is approximately twice the width of that of the first dorsal vertebrae, contrasting with all other extant crocodylians (286-0). This requires serial measurements of dorsal vertebrae 1–10. Secondly, whereas the fusion of the parapophysis and diapophysis occurs anterior to or on the 12th dorsal vertebra in most crocodylians, it occurs at the 13th dorsal vertebra in *Gavialis gangeticus*. Given ongoing work revising *Gavialis* and closely related forms (e.g. Martin, 2019), these characters have been included here to aid future studies that might be able to incorporate more *Gavialis* OTUs, and that might demonstrate their wider distribution within the genus.

288. Presacral vertebrae, maximum mediolateral width across prezygapophyses: sub-equal throughout (0); increases posteriorly throughout presacral vertebrae (1) (after Iijima and Kobayashi, 2019 [250]).

This character requires measurements of width across the prezygapophyses in all presacral vertebrae, and character scores were based on the observations of Iijima and Kobayashi (2019, fig.3b)

and Iijima and Kubo (2019b). The width across the prezygapophyses appears very consistent across presacral vertebrae in *Gavialis gangeticus* and *Toyotamaphimeia*. By contrast, in all other extant crocodylians, as well as *Penghusuchus pani*, the width across the prezygapophyses increases posteriorly along the presacral vertebrae.

Sacral vertebrae

289. Sacral vertebra 1, anterior extent of sacral rib capitulum: anterior to tuberculum (visible in dorsal view) (0); at the same level as tuberculum (obscured in dorsal view) (after Brochu, 1997a [13]).

In dorsal view, the anterior surface of the first sacral rib is visible in *Gavialis gangeticus* (Fig. 122C) and all extant alligatorids, e.g. *Alligator mississippiensis* (Fig. 122A) (289-0). This results from the more anterior position of the capitulum relative to the tuberculum. By contrast, the capitulum is obscured in dorsal view by the tuberculum in all extant crocodylids (289-1) (Fig. 122B). *Tomistoma schlegelii* was scored with the 'crocodylid' condition in most earlier datasets (e.g. Brochu et al., 2012; Narváez et al., 2016) but, as noted by Sookias (2020), it exhibits a dorsally exposed capitulum (Fig. 122D). Other "tomistomines" show variation in this feature. For example, whereas the sacral rib capitulum is dorsally exposed in *Penghusuchus* (Shan et al., 2009, fig.10a), it is concealed in *Toyotamaphimeia* (Kobayashi et al., 2006, fig.43B).

290. Sacral vertebra 2, posterior extent of ribs: extend beyond level of posterior extent of postzygapophyses (0); terminate level with or anterior to level of postzygapophyses (after Iijima and Kobayashi, 2019 [251]).

Where known, in most eusuchians, the posterolateral tip of the second sacral rib extends posteriorly beyond the level of the postzygapophyses (Fig. 122A, B, D). By contrast, in *Gavialis gangeticus* (Fig. 122C) and *Bernissartia fagesii* (IRScNB 1538), the second sacral rib terminates notably further anteriorly, such that it does not exceed the posterior extent of the postzygapophyses. This character was modified from Iijima and Kobayashi (2019, character 251), who used the posterior end of the centrum as a marker point for the posterior extent of the sacral ribs; however, the sacral ribs extend beyond the posterior end of the centrum in all taxa in this dataset.

Caudal vertebrae

291. Caudal vertebra 1, centrum: opisthocoelous or procoelous (0); biconvex (1) (after Salisbury et al., 2006 [171]; Norell and Clark, 1990 [9]; Clark, 1994 [94]).

In *Bernissartia fagesii* (IRScNB 1538) and most eusuchians, the centrum of the first caudal vertebra is biconvex. This contrasts with *Isisfordia duncanii*, which exhibits a procoelous first caudal

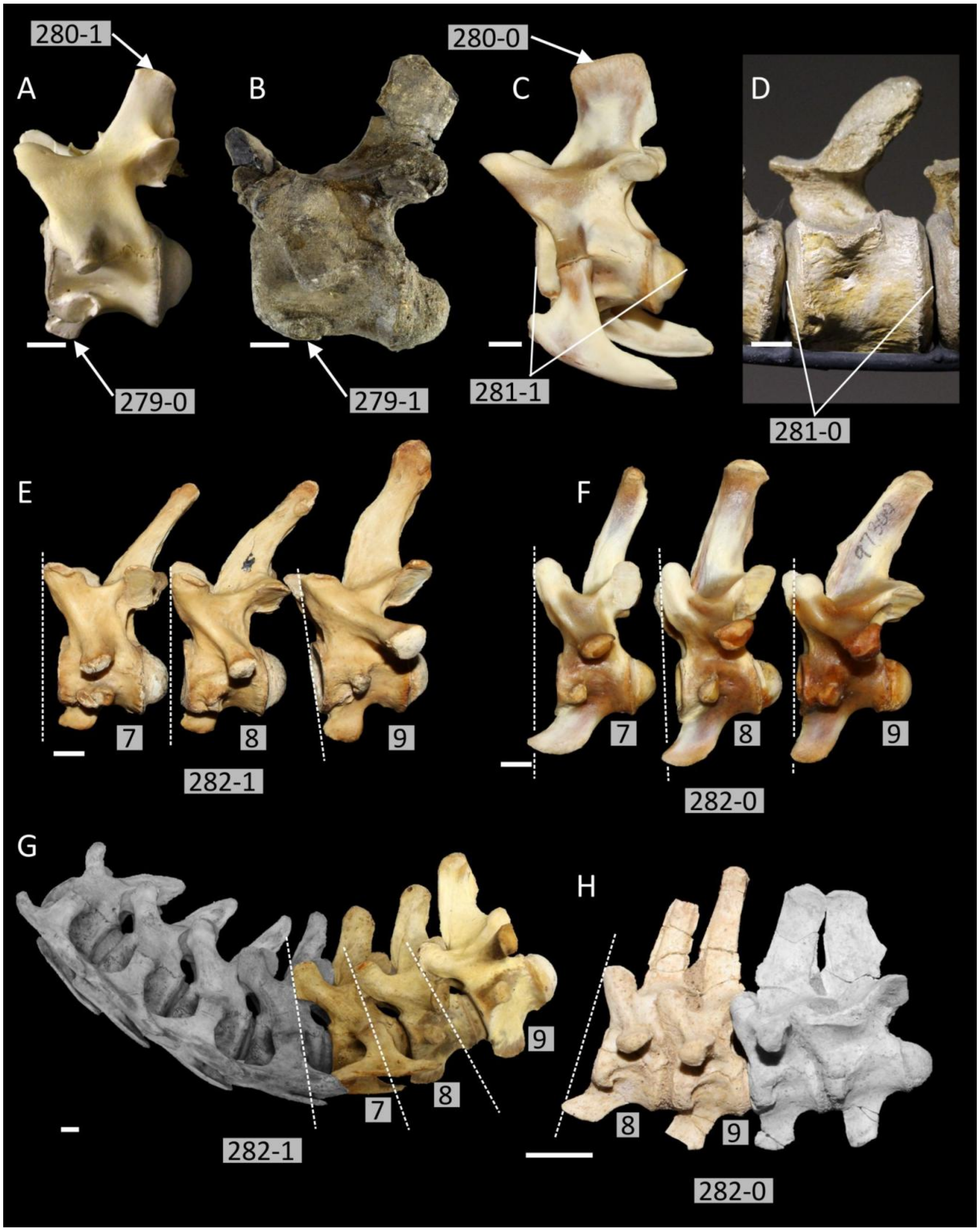

Figure 120: Morphology of the cervical vertebrae. **A**–**C**, 3rd cervical vertebra (i.e. 1st postaxial vertebra) in: **A**, *Crocodylus porosus* (QM J48127); **B**, *Eosuchus minor* (USNM 181577); **C**, *Caiman yacare* (AMNH 97300); **D**, *Champsosaurus lemoinei* (IRScNB 1582); **E**–**H**, posteriormost cervical vertebrae (7–9) of **E**, *Crocodylus acutus* (AMNH 7121); **F**, *Caiman yacare* (AMNH 97300); **G**, *Gavialis gangeticus* (UMCZ R5783); **H**, *Alligator mcgrewi* (AMNH FAM 8700). Numbers indicate position in cervical series. All scale bars = 1 cm.

centrum (Salisbury et al., 2006), and *Theriosuchus pusillus* (NHMUK 48216), in which the anteriormost caudal centra are opisthocoelous.

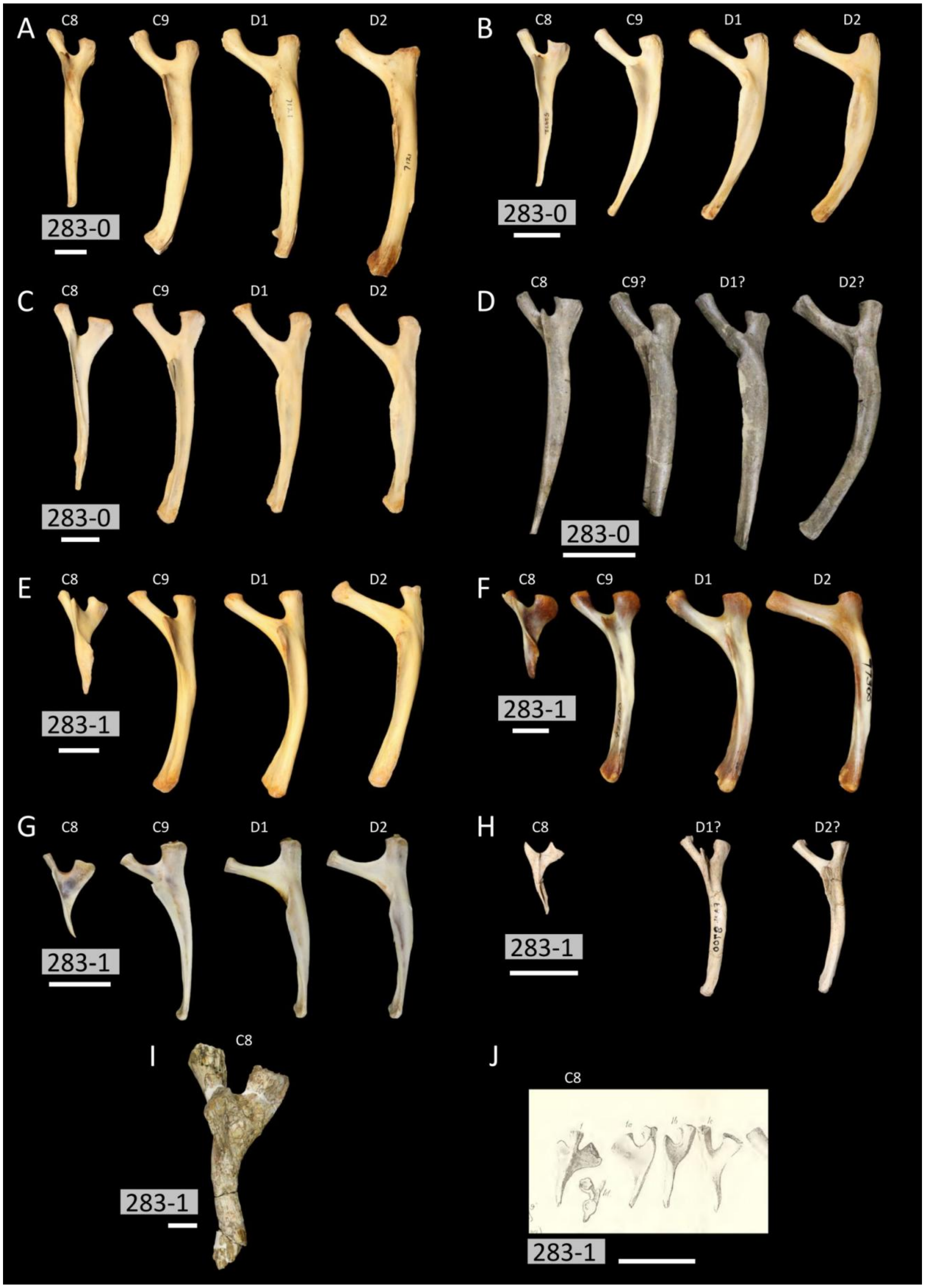

Figure 121: Morphology of the cervical and dorsal ribs in selected crocodylians. All left ribs in anterior view except J–right rib. **A**, *Crocodylus acutus* (AMNH 7121); **B**, *Tomistoma schlegelii* (USNM 52972); **C**, *Gavialis gangeticus* (AMNH 110145); **D**, *'Crocodylus' affinis* (USNM 18171); **E**, *Alligator mississippiensis* (AMNH 71621); **F**, *Caiman yacare* (AMNH 97300); **G**, *Melanosuchus niger* (AMNH 97325); **H**, *Alligator mcgrewi* (AMNH FAM 8700, D1 and D2 digitally reversed); **I**, *Purussaurus neivensis* (UCMP 39657); J, *Diplocynodon darwini* (Ludwig, 1877: Plate12, fig.1). All scale bars = 2 cm.

292. Caudal centra, posterior to first caudal vertebra: procoelous (0); amphicoelous or opisthocoelous (1) (after Norell and Clark, 1990 [9]; Clark, 1994 [94]; Salisbury et al., 2006 [171]; Pol et al., 2009 [94]).

In *Bernissartia fagesii* (IRScNB 1538) and most eusuchians, the caudal centra posterior to the first caudal vertebra are procoelous. By contrast, in *Shamosuchus djadochtaensis* (Pol et al., 2009) and *Theriosuchus pusillus* (NHMUK 48216), they are amphicoelous.

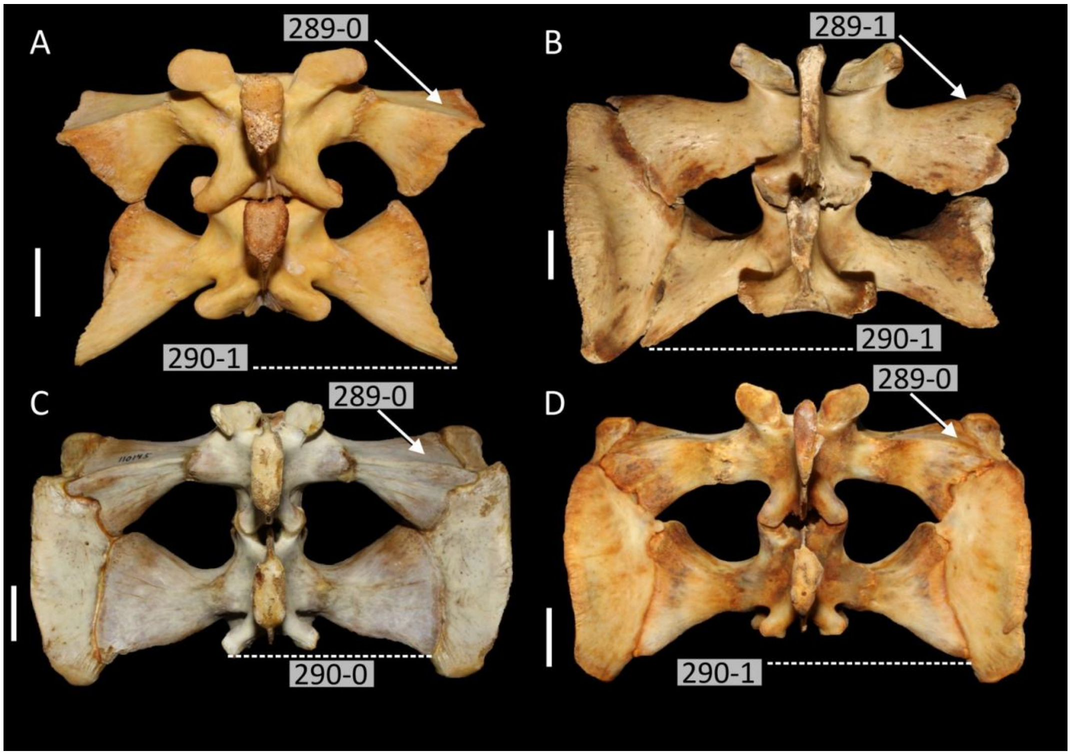

Figure 122: Dorsal view of the sacral vertebrae 1 (top) and 2 (bottom). **A**, *Alligator mississippiensis* (AMNH 71621); **B**, *Crocodylus acutus* (AMNH 7121); **C**, *Gavialis gangeticus* (AMNH 110145); **D**, *Tomistoma schlegelii* (AMNH 113078). All scale bars = 2 cm.

293. Caudal vertebrae, number with transverse processes: first 15 or fewer (0); 16 to 20 (1); 21 or more (2) (after Iijima and Kobayashi, 2019 [253]) (ORDERED).

In all extant crocodylians, the width across the vertebral transverse processes increases posteriorly through the vertebral column, reaching a maximum around dorsal vertebrae 7–9 (Iijima & Kubo, 2019b, fig.4). The width across the transverse processes then decreases through the remaining dorsal vertebrae, continuing through the anteriormost caudal vertebrae, until becoming lost in the posteriormost caudal vertebrae. Iijima and Kobayashi (2019) noted differences in the number of caudal vertebrae with transverse processes in extant crocodylians. In general, the transverse processes extend further posteriorly in extant alligatorids (293-1, 293-2) than in crocodylids, *Gavialis gangeticus*, and *Tomistoma schlegelii* (293-0). Three exceptionally preserved fossil taxa are newly

scored in this dataset: *Bernissartia fagesii* (IRScNB 1538), *Diplocynodon darwini* (HLMD Me 10262), and *Asiatosuchus germanicus* (SMNK 1801), all of which exhibit transverse processes on 15 or fewer caudal vertebrae (293-0). The character is also ordered.

294. Caudal vertebrae, articular surfaces of chevrons posterior to the first: open, or partially fused (0); completely fused (1) (after Iijima and Kobayashi, 2019 [254]).

This character was illustrated by Iijima and Kobayashi (2019, fig.S5), which shows that the articular surfaces of the haemal arches (connecting the chevrons with the ventral surface of the caudal vertebrae) are fused (294-1) in all extant alligatorids, with the exception of *Alligator mississippiensis*. By contrast, in all extant crocodylids, *Gavialis gangeticus*, and *Tomistoma schlegelii* the articular surfaces are incipiently or completely fused (294-1). Among fossil crocodylians, *Borealosuchus* (Erickson, 1976, fig.20) and *Piscogavialis jugaliperforatus* (SMNK 1282 PAL) exhibit incipiently or unfused chevrons, whereas *Diplocynodon darwini* (SMF Me 1137) exhibits the fully fused condition, like most extant alligatorids. The first chevron is excluded from the character, as its articular surfaces are always fused.

Hyoid and interclavicle

295. Hyoid, shape of dorsal projection (cornu): plate-shaped (0); rod-shaped (1) (after Brochu, 1997a [57]).

296. Hyoid, flare of dorsal projection (cornu): absent (0); present (1) (after Brochu, 1997a [58]).

Characters 295 and 296 describe two independent features in the morphology of the proximal end of the hyoid (cornu), as outlined by Brochu (1999, fig.56). The hyoids flare anteriorly in all extant caimanines (Wermuth, 1953, fig.11a), as well as *Gavialis gangeticus* (Fig. 123A) and *Borealosuchus formidabilis* (Erickson, 1976, fig.12). This contrasts with the parallel-sided proximal end of the hyoid in all extant crocodylids, *Tomistoma schlegelii*, and*Alligator*. The hyoid of extant crocodylids can be distinguished further from all other crocodylians by the presence of a cylindrical, rod-shaped proximal end (295-1). This contrasts with the mediolaterally narrow, plate-shaped proximal end of the hyoid in all other crocodylians (where known). Sookias (2020) did not consider the character describing the presence or absence of hyoid flare to be robust, stating that *Tomistoma* and *Gavialis* share the same flared condition. However, the observations in this study re-affirm earlier character scores (Fig. 123).

297. Interclavicle flexure: minimal dorsoventral flexure, minimum angle between anterior and posterior ends $<15°$ (0); moderate dorsoventral flexure, minimum angle 15–25° (1); severe dorsoventral flexure, minimum angle $> 25°$ (2) (after Brochu, 1997a [30]) (ORDERED).

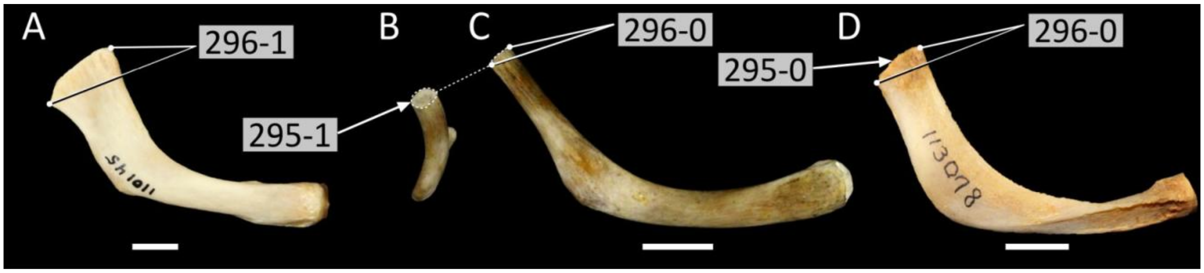

Figure 123: Variation in hyoid morphology (all left hyoids in lateral view except B, which is in anterior view). **A**, *Gavialis gangeticus* (AMNH 110145), **B**–**C**, *Crocodylus porosus* (AMNH 7115); **D**, *Tomistoma schlegelii* (AMNH 113078). All scale bars = 1 cm.

The interclavicle is an ossified median process that projects from the anterior end of the sternum. Brochu (1999) described variation in the dorsoventral curvature (flexure) of the interclavicle, which was discretised into three, subjectively-defined character states describing increasing degrees of flexure. Sookias (2020) recognised inconsistencies between character scores in earlier datasets and observations of actual specimens, as is also found here. As described by Brochu (1999), and later scored by Brochu et al. (2012), *Paleosuchus* exhibits the most extreme dorsoventral flexure (297-2), which is also shared by *Osteolaemus* (AMNH 69057). This condition is newly recognised in *Alligator mcgrewi* (Fig. 124D). By contrast with Brochu et al. (2012), severe flexure of the interclavicle was not observed in any *Crocodylus* species, which instead show little to no flexure, e.g. *Crocodylus johnstoni* (Fig. 124B). Minimal dorsoventral flexure (297-0) was also observed in extant *Caiman* and *Melanosuchus*, as well as extant *Alligator* and *Brachychampsa montana* (Fig. 124A). An intermediate degree of flexure (297-1) is exemplified by *Gavialis gangeticus* (Fig. 124C) and *Tomistoma schlegelii* (AMNH 113078). This character is ordered given the clear, continuous nature of increasing flexure.

298. Interclavicle, shape of anterior end (at maturity): plate-shaped (0); rod-shaped (1) (after Brochu, 1997a [31]).

A rod-like anterior tip of the interclavicle (298-1) has been recovered as an unambiguous autapomorphy of *Paleosuchus* (Brochu, 1999). Furthermore, in all datasets examined here, *Paleosuchus* is the only taxon to exhibit this condition (e.g. Brochu et al., 2012; Cidade et al., 2017; Narváez et al., 2016). Sookias (2020) noted that there was no marked difference between the condition of the interclavicle in *Paleosuchus* and other crocodylians. However, based on specimens photographed in his supplementary material, this could be a result of the study of juvenile specimens. Indeed, Brochu (1999) stated that 'mature' *Paleosuchus* specimens exhibit this condition, implying that it is absent in juveniles. Accordingly, larger *Paleosuchus* interclavicles studied here do exhibit a rod-like condition (Fig. 124F), which is distinct from the plate-shaped condition of all other crocodylians (Fig. 124E).

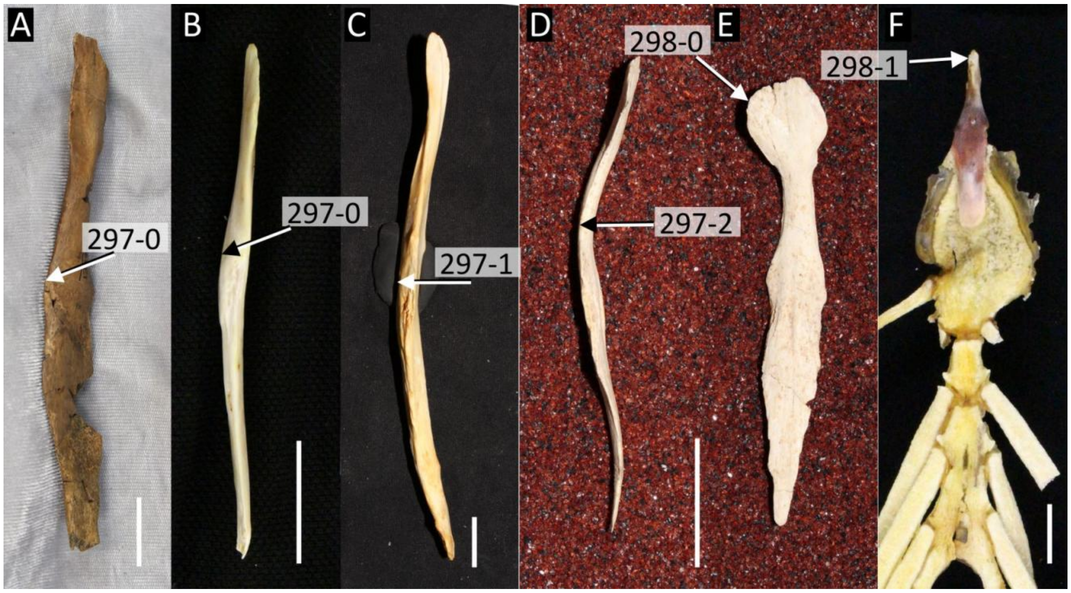

Figure 124: Morphology of the interclavicle. **A**–**C**, left lateral view of the interclavicle in **A**, *Brachychampsa montana* (UCMP 133901); **B**, *Crocodylus johnstoni* (QM J58446) **C**, *Gavialis gangeticus* (USNM 576261, digitally reversed); **D**–**E**, *Alligator mcgrewi* (AMNH FAM 8700) in left lateral (**D**) and dorsal views (**E**); **F**, *Paleosuchus palpebrosus* (AMNH 97326, dorsal view attached to sternum). All scale bars = 2 cm.

Appendicular skeleton

Scapulo-coracoid

299. Scapula, deltoid crest shape: thin, with sharp margin (0); wide, with broad margin (1) (after Brochu, 1997a [23]).

The deltoid crest (acromion process) of the scapula serves as the point of origin of *M. coracobrachialis brevis dorsalis* and *M. deltoideus clavicularis* (Meers, 2003). The derived character state applies principally to species of *Alligator*, e.g. *A. mississippiensis*, and *A. sinensis*, which at maturity exhibit a broad crest (Brochu, 1999, fig.51) (Fig. 125A). One might expect that this character will be influenced by ontogeny, i.e. juvenile individuals of a species will exhibit a narrow crest (299-0), which broadens with maturity (299-1). However, at maturity, the scapulae of taxa with the derived character state, e.g. *A. mississippiensis*, consistently exhibit a broader deltoid crest than those of equally sized or even large crocodylians (Fig. 125B, D, F).

300. Scapulocoracoid synchondrosis: closes very late in ontogeny (0); closes early in ontogeny (1) (after Brochu, 1997a [24]).

In all extant caimanines, the scapulocoracoid synchondrosis closes relatively early in ontogeny (300-0), before that of the neurocentral sutures (Brochu, 1995) (Fig. 125H). By contrast, the suture remains open in all but the most mature individuals of other extant crocodylians (300-1) (Brochu,

1999) (Fig. 125G). In this study, all crocodylians known from mature individuals, with disarticulated scapulocoracoids, are scored with the plesiomorphic character state, given that had the scapulocoracoid been fused early in ontogeny, these elements should presumably still be in articulation. Very few fossil crocodylians are scored for character state (1). The material known for *Necrosuchus* represents an immature individual, since the dorsal and sacral neurocentral sutures are still visible (Brochu, 1996). Based on this, and the incipient closure of the scapulocoracoid synchondrosis, it can be scored for the derived character state. A specimen of *Mourasuchus arendsi* preserving the scapulocoracoid (Cidade et al., 2018) was close to maturity, based on partial closure of the cervical neruocentral sutures. Since it exhibits incipient closure of the scapulocoracoid synchondrosis, it too can be scored for the derived condition.

301. Scapulocoracoid facet, shape anterior to glenoid fossa (at maturity): uniformly narrow (0); broad immediately anterior to glenoid fossa, tapering anteriorly (1) (after Brochu, 1997a [25]).

The morphology of the scapulocoracoid facet can be determined by viewing either the scapula or coracoid in proximal view (Fig. 125I–M). Brochu (1999) noted that the facet is tear-drop-shaped in all species, but some (mostly 'brevirsotrine') crocodylians exhibit a prominent difference in facet width between the anterior and posterior ends, which is accentuated at maturity (301-1). Sookias (2020) rejected this character as it was unobservable in the sample of crocodylians in his study. However, this sample appears to be entirely juvenile, and all the relevant images are in lateral view, from which point the morphology cannot be determined. The observations of Brochu (1999) are supported here, albeit with some character score changes. A uniformly narrow facet is exhibited in *Bernissartia fagesii* (IRScNB 1538), some *Borealosuchus* species (e.g. *B. sternbergii*), *Brachychampsa montana* (Fig. 125M), and some "gavialoids" e.g. *Eogavialis africanum* (Fig. 125K) and *Eosuchus minor* (USNM 355967). However, the condition in *Gavialis gangeticus* (Fig. 125L) is considered closer to that of extant crocodylids (Fig. 125I), *Tomistoma schlegelii*, alligatorids, and the gavialoid *Piscogavialis jugaliperforatus* (Fig. 125J).

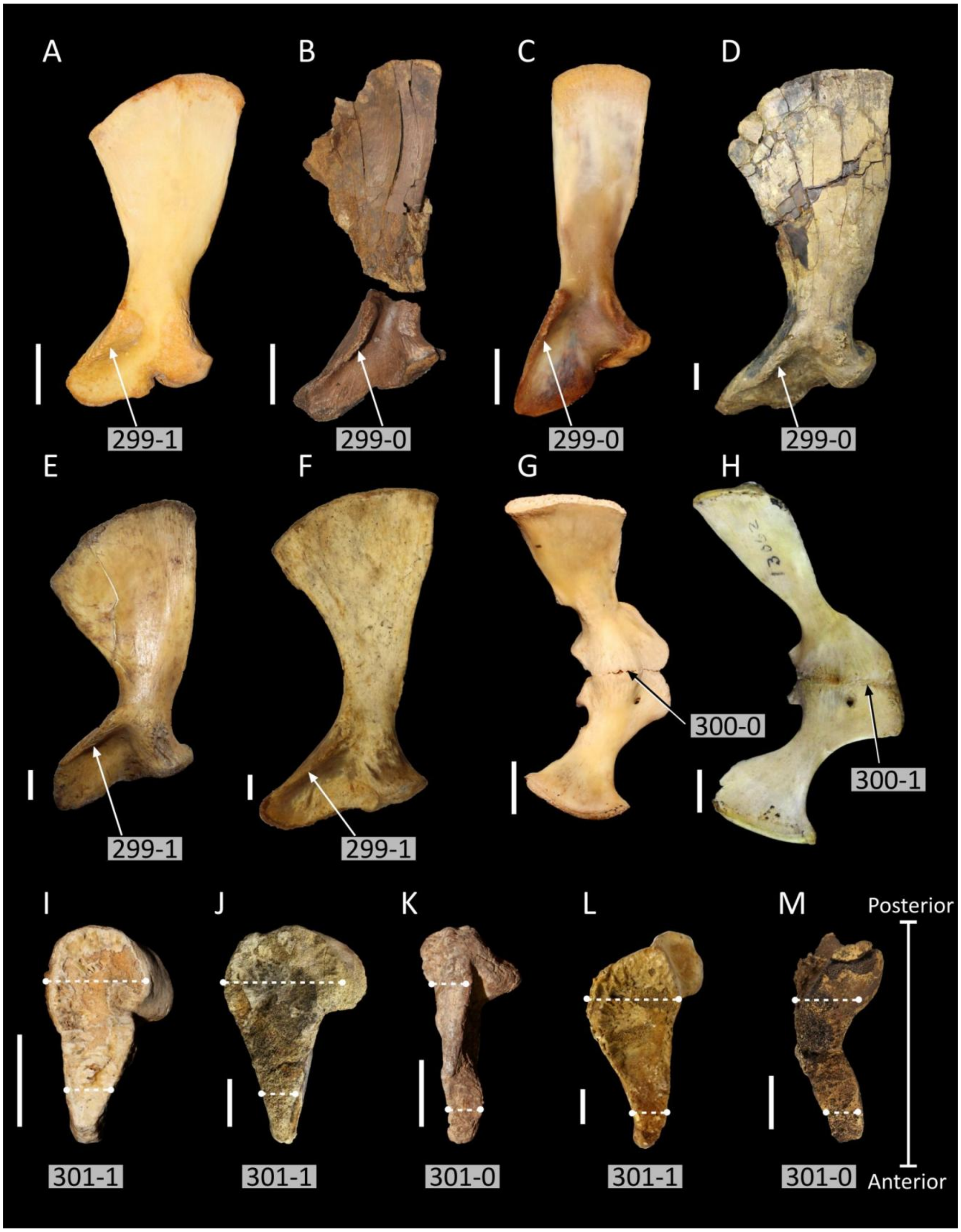

Figure 125: Morphology of the scapulacoracoid. A–F, lateral view of the left scapula in: **A**, *Alligator mississippiensis* (AMNH 71621); **B**, *Brachychampsa montana* (UCMP 133901); **C**, *Caiman yacare* (AMNH 97300); **D**, *Asiatosuchus germanicus* (SMF Me 1801); **E**, *Voay robustus* (NHMUK R 36661); **F**, *Gavialis gangeticus* (UMZC R 5783); G–H, medial view of the left scapula coracoid in: **G**, *Alligator sinensis* (USNM 292078, digitally reversed) and **H**, *Caiman crocodilus* (FMNH 13062); I–K, proximal view of the articular facet of the left coracoid in: **I**, *Crocodylus acutus* (AMNH 7121); **J**, *Piscogavialis jugaliperforatus* (SMNK 1282 PAL); **K**, *Eogavialis africanum* (SMNS 11225, digitally reversed); L–M, proximal view of the articular facet of the right scapula in **L**, *Gavialis gangeticus* (UMZC R 5783); **M**, *Brachychampsa montana* (UCMP 133901, digitally reversed). All scale bars = 2 cm.

Forelimb

302. Humerus, proximal margin of deltopectoral crest: straight, emerging smoothly from proximal end of the humerus (0); concave, emerging abruptly from proximal end of humerus (1) (after Brochu, 1997a [26]).

The morphology of the deltopectoral crest of the humerus appears to vary continuously in eusuchians. For example, in *Gavialis gangeticus* (Fig. 126A), the crest is very low, lacking a prominent apex. This condition is broadly similar to several *Borealosuchus* species (Fig. 126B), *Leidyosuchus canadensis* (Fig. 126C), and the outgroup, *Bernissartia fagesii* (IRScNB 1538). By contrast, the deltopectoral crest is more prominent in extant crocodylids, *Alligator* (Fig. 126F) and *Diplocynodon* (Fig. 126D). In extant caimanines and some fossil representatives, the apex of the crest appears even sharper (Fig. 126G–H). It was not considered practical to characterise this morphology as a continuous character. As such, following earlier studies, the morphology is discretised as a binary character describing either a low, smoothly emerging crest (302-0), or an abruptly emerging crest (302-1).

303. Humerus, axial rotation of the proximal epiphysis relative to the distal epiphysis: small, ventral surface of the proximal epiphysis not visible in medial view (0); large, ventral surface exposed in medial view (1) (new character, after Stein et al., 2012).

This character describes the degree of torsion between the proximal and distal ends of the humerus. Uniquely among extant crocodylians, the proximal epiphysis of the humerus is highly rotated relative to the distal epiphysis in *Gavialis gangeticus* (Fig. 126A). Consequently, when viewed medially, the ventral surface of the proximal epiphysis is visible. This can also be described in terms of the orientation of the long axes of the distal and proximal epiphyses, which are offset in *Gavialis*. By contrast, other extant crocodylians exhibit a minimal degree of torsion, such that the long axes of the epiphyses are aligned, and the ventral surface of the proximal epiphysis is not visible e.g. *Alligator mississippiensis* (Fig. 126F). Other than *Gavialis gangeticus*, the derived condition is only tentatively found in the "tomistomine" *Penghusuchus pani* (Shan et al., 2009, fig.14).

304. Humerus, scarring on proximodorsal surface for *M. teres major* and *M. dorsalis scapulae* (at maturity): two muscle scars (0); single muscle scar (*M. teres major* and *M. dorsalis scapulae* insert on common tendon) (1) (after Brochu, 1997a [29]).

This character was introduced by Brochu (1997b), and has never been illustrated and only briefly described (Brochu, 1997a). According to earlier datasets (Brochu et al., 2012), *Bernissartia fagesii*, and some "gavialoids", (e.g. *Gavialis gangeticus*, *Eosuchus minor*, and *Thoracosaurus*) exhibit two muscle scars on the dorsal surface of the humerus (304-0), approximately level with the loca-

tion of the deltopectoral crest on the ventral surface. By contrast, all other eusuchians (e.g. Fig. 126J) exhibit one prominent scar (304-1), which serves as the insertion point for *M. teres major* and *M. latissimus dorsi* (Meers, 2003). The latter was commonly found in most crocodylians in this dataset (Fig. 126J). Two scars matching the description of the plesomorphic character state were observed in a mature specimen of *G. gangeticus* (UMZC R 5783) (Fig. 126K); however, these are not apparent in slightly smaller specimens, which appeared to have only one prominent scar (AMNH 110145, USNM 576261). As such the character must be scored in mature specimens. Two scars are also present in *Eosuchus minor* (USNM 355967 Brochu, 2006b), and *Thoracosaurus*. The condition in the latter is based on character scores in a previous dataset (Brochu et al., 2012), since appendicular remains of *Thoracosaurus* were not studied herein.

305. Ulna, shape of olecranon process: narrow and sub-angular (0); wide and rounded (1) (after Brochu, 1997a [27]).

The olecranon process is narrow (mediolaterally compressed) in *Bernissartia fagesii* and all species of *Borealosuchus* (Fig. 127A). This contrasts with the broadly rounded olecranon process of all other crocodylians (Fig. 127B), were known.

306. Ulna, proximal diaphysis curved (0); straight (1) (new character, based on personal observations).

In most eusuchians, the ulna is almost entirely straight and robust, with a slight dorsal curvature at its proximal end (Fig. 127B). By contrast, in *Bernissartia fagesii* (IRScNB 1538), *Isisfordia duncani* (QM F36211), and at least two *Borealosuchus* species (*B. sternbergii* [Fig. 127A] and *B. formidabilis* [Erickson, 1976, fig.26]), the ulna is slender, and prominently curved at its proximal end.

Pelvic girdle

307. Ilium, preacetabular process shape: acute, pointed anteriorly (0); broad, rounded anteriorly (1) (after Benton and Clark, 1988; Clark, 1994 [84]; Brochu, 1997a [34]).

All earlier studies characterised the preacetabular process of the ilium as either "prominent" or "virtually absent" (Brochu, 1997b; Brochu et al., 2012). However, examination of a large sample of crocodylian ilia reveals a significant amount of variation in the development of this process (Fig. 128). A convexity on the anterodorsal margin of the ilium, which could be interpreted as a preacetabular process, occurs in most eusuchians, but differs in its size. Although, this justifies the use of the terms "prominent" or "virtually absent", it introduces subjectivity. Here, a distinction is made between the morphology of the preacetabular process, from those with an acute tip (307-0) to those with a rounded tip (307-1). As a result, the plesiomorphic condition is more widespread.

Whereas *Bernissartia fagesii* (IRScNB 1538), *Borealosuchus* (Fig. 128A), and *Gavialis gangeticus* (Fig. 128D) exhibit a prominent, acute preacetabular process (307-0), all extant alligatorids and most crocodylids exhibit a smaller, rounded process (307-1). By contrast to earlier studies (e.g. Brochu et al., 2012), the morphology of the preacetabular process in *Diplocynodon darwini* (Fig. 128C), *Crocodylus acutus* (Fig. 128H), and *Alligator prenasalis* (Fig. 128Q), is scored with the plesiomorphic condition, whereas that of *Eogavialis africanum* (Fig. 128E) more closely resembles the derived state.

308. Ilium, dorsal outline of postacetabular process: convex, no dorsal indentation (0); broadly concave with a small indentation (1);strongly concave, with an acute indentation (“wasp-waisted”) (2) (after Brochu, 1997a [28]) (ORDERED).

309. Ilium, posterior margin of the postacetabular process: deep, anteroposterior length to dorsoventral height ratio <1 (0); shallow, length to height ≥ 1 (1) (after Brochu, 1997a [28]; Groh et al., 2019 [523]).

Characters 308 and 309 were derived by reductively coding character 28 in Brochu (1997b), which originally combined descriptions of the dorsoventral height (Character 309) and dorsal outline (Character 308) of the postacetabular process. Under the original format, some combinations of morphological features could not be accounted for in several crocodylians. For example, all *Diplocynodon* species were described as exhibiting a postacetabular process that was deep, lacking a dorsal indentation (Brochu, 1997b, character 28-4). Although this applies to *D. hantoniensis* (NHMUK OR 30362) and *D. darwini* (Fig. 128C), *D. ratelii* exhibits a deep postacetabular process (309-0) with an acute indentation (308-2) (Fig. 128B).

310. Ilium, postacetabular process: projects posteriorly (0); posterodorsally (1) (after Wu and Suez, 1996 [41]; Pol and Norell, 2004 [110]; Groh et al., 2020 [530]).

This character is not usually applied to datasets consisting primarily of crocodylian taxa (e.g. Brochu, 1999; Brochu et al., 2012; Jouve et al., 2015; Lee & Yates, 2018); however, a posterodorsally directed postacetabular process is present in *Borealosuchus formidabilis* (Fig. 128A) and *Borealosuchus wilsoni* (FMNH PR 1674) in this dataset. In *Bernissartia*, and all other eusuchians (Fig. 128B–R), the process is primarily posteriorly directed.

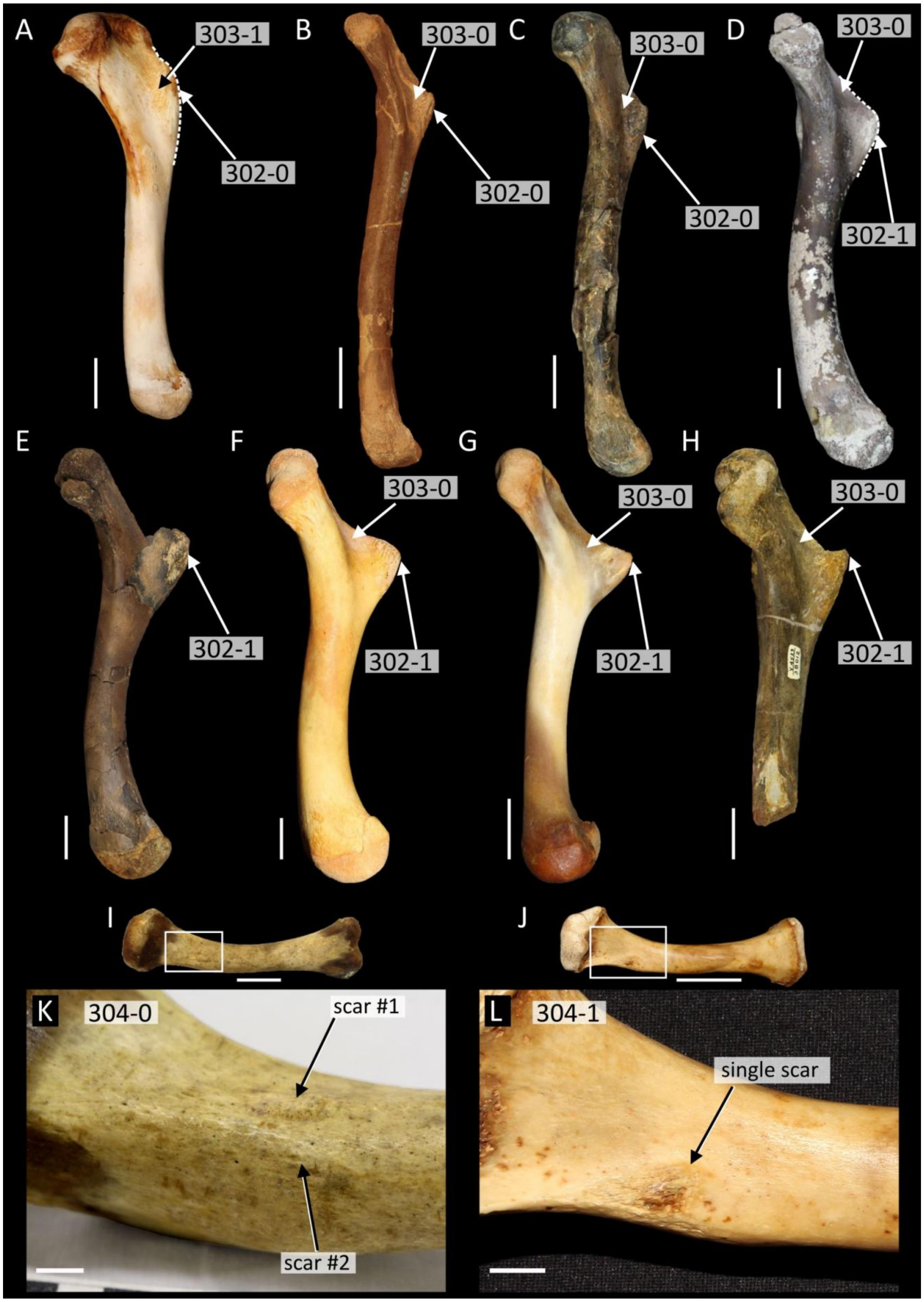

Figure 126: Variation in humeral morphology in Crocodylia. **A**–**H**, medial view of the left humerus in: **A**, *Gavialis gangeticus* (AMNH 110145); **B**, *Borealosuchus sternbergii* (USNM 6533); **C**, *Leidyosuchus canadensis* (UCMP 131696, digitally reversed); **D**, *Diplocynodon hantoniensis* (NHMUK OR 30206, digitally reversed); **E**, *Brachychampsa montana* (UCMP 133901); **F**, *Alligator mississippiensis* (AMNH 71621); **G**, *Caiman yacare* (AMNH 97300); **H**, *Mourasuchus atopus* (UCMP 38012). **I**–**J**, dorsal view of the humerus in **I**, *Gavialis gangeticus* (UMZC R 5783); **J** *Crocodylus acutus* (AMNH 7121); **K**–**L**, enlargement of muscle attachments highlighted in I and J respectively. Scale bars in A–J = 2 cm, scale bars in K–L = 1 cm.

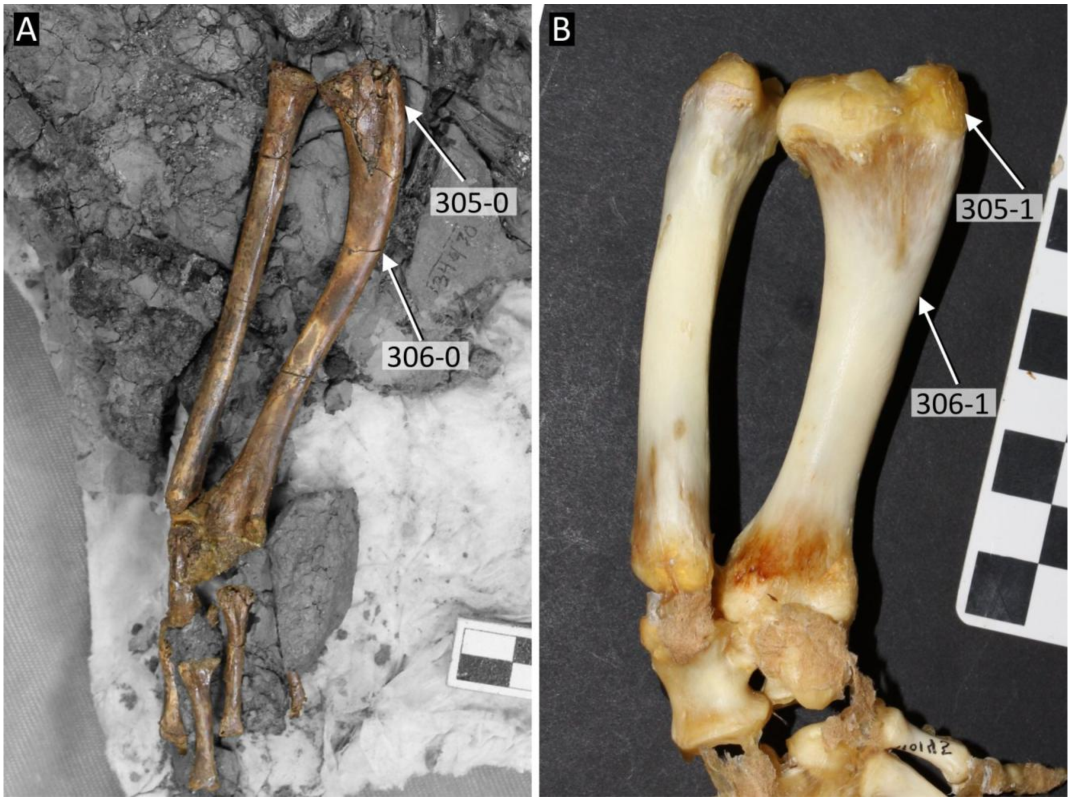

Figure 127: Medial view of the right ulna and radius of **A**, *Borealosuchus sternbergii* (UCMP 134430); **B**, *Gavialis gangeticus* (AMNH 110145, digitally reversed). All scale bars = cm.

Soft tissue

311. Cerebrum, posterodorsal outline anterior to optic lobe: flat (0); with sharp step (1) (new character, after Serrano-Martinez et al., 2019b).

In extant alligatorids (where known), there is a pronounced step on the dorsal margin of the cerebrum, anterior to the region of the optic lobes (311-1) (Serrano-Martínez et al., 2019b). This condition also appears in some extant crocodylids, e.g. *Osteolaemus tetraspis* and *Crocodylus niloticus*, but not *Crocodylus johnstoni*, which has a broadly rounded posterodorsal outline (311-0) (Serrano-Martínez et al., 2019b). The broadly rounded condition also characterises the non-crocodylian eusuchian *Lohuecosuchus megadontos* (Serrano-Martínez et al., 2019a), 'basal' alligatoroids (e.g. *Diplocynodon tormis* and *Leidyosuchus canadensis*), *Tomistoma schlegelii*, *Gavialis gangeticus*, and *Gryposuchus neogaeus*) (Bona et al., 2017; Serrano-Martínez et al., 2019b; Storrs et al., 1983).

312. Medial pharyngeal sinus, ratio of ventral length (measured from ventral tip to junction with basisphenoid diverticulum) to dorsal length (measured from basisphenoid diverticulum to dorsal tip): ≥ 2 (0); < 2 (1) (new character, after Serrano-Martinez et al. 2019b).

Serrano-Martínez et al. (2019b) described differences in length of the medial pharyngeal sinus in

Crocodylia. In their sample of extant crocodylids (*Osteolaemus tetraspis*, *Crocodylus johnstoni*, and *Crocodylus niloticus*), the length of the ventral portion of the medial pharyngeal sinus (i.e. below the intersection with the basisphenoid diverticulum) is twice the length of the dorsal portion (312-0) (Serrano-Martínez et al., 2019b, fig.5). This condition is otherwise only known in *Lohuecosuchus megadontos* (Serrano-Martínez et al., 2019a). By contrast, the ventral portion of the medial pharyngeal sinus is shorter (312-1) in alligatoroids (*Alligator mississippiensis*, *Caiman crocodilus*, *Diplocynodon tormis*, and *Mourasuchus arendsi*), *Tomistoma schlegelii*, and *Gavialis gangeticus*) (Bona et al., 2013a; Serrano-Martínez et al., 2019b).

313. Keratinised buccal cavity: present (0); absent (1) (after Brochu, 1997a [159], adapted from Taplin and Grigg, 1989).

Taplin and Grigg (1989) noted that all extant crocodylids, as well as *Tomistoma schlegelii* and *Gavialis gangeticus*, share a similar morphology of the tongue and buccal cavity, which has a yellow/ orange tint, and is keratinised (Grigg & Kirshner, 2015, fig.11.23). This keratinisation reduces the permeability of the buccal cavity, which is interpreted as an adaptation for inhabiting saltwater environments (Taplin & Grigg, 1989). By contrast, all extant alligatorids lack keratinisation of the tongue and buccal cavity, in which it is smooth, with a glutinous sheen (Grigg & Kirshner, 2015, fig.11.30).

314. Integumentary sensory organs, distribution on body: cranial only (0); cranial and postcranial (1) (after Lee and Yates, 2018 [275], adapted from Grigg and Kirshner, 2015).

Integumentary sense organs (ISOs) (Brazaitis, 1987) are small, millimetre-sized black pits that occur on the cranial scales of all extant crocodylians (Fig. 129A). These serve a diverse number functions, including the detection of water-borne disturbances (Grigg & Kirshner, 2015). Whereas in extant alligatorids ISOs are restricted to the skull and mandible, they occur all over the body and limbs of extant crocodylids, *Tomistoma schlegelii*, and *Gavialis gangeticus* (Fig. 129C) (Grigg and Kirshner, 2015).

315. Ventral scales, follicle gland pore: present (0); absent (1) (after Poe, 1996 [113]; Brochu, 1997a [155]; adapted from Brazaitis, 1973).

Brazaitis (1973) described the presence of 'follicle gland pores' on the ventral scales of all extant crocodylids, *Tomistoma schlegelii* and *Gavialis gangeticus*, whereas these are absent in extant alligatorids (Brazaitis, 1973, fig.2C). The difference in structure between these pores and ISOs has not been explored, but they are tentatively treated independently pending examination of additional crocodylian skins.

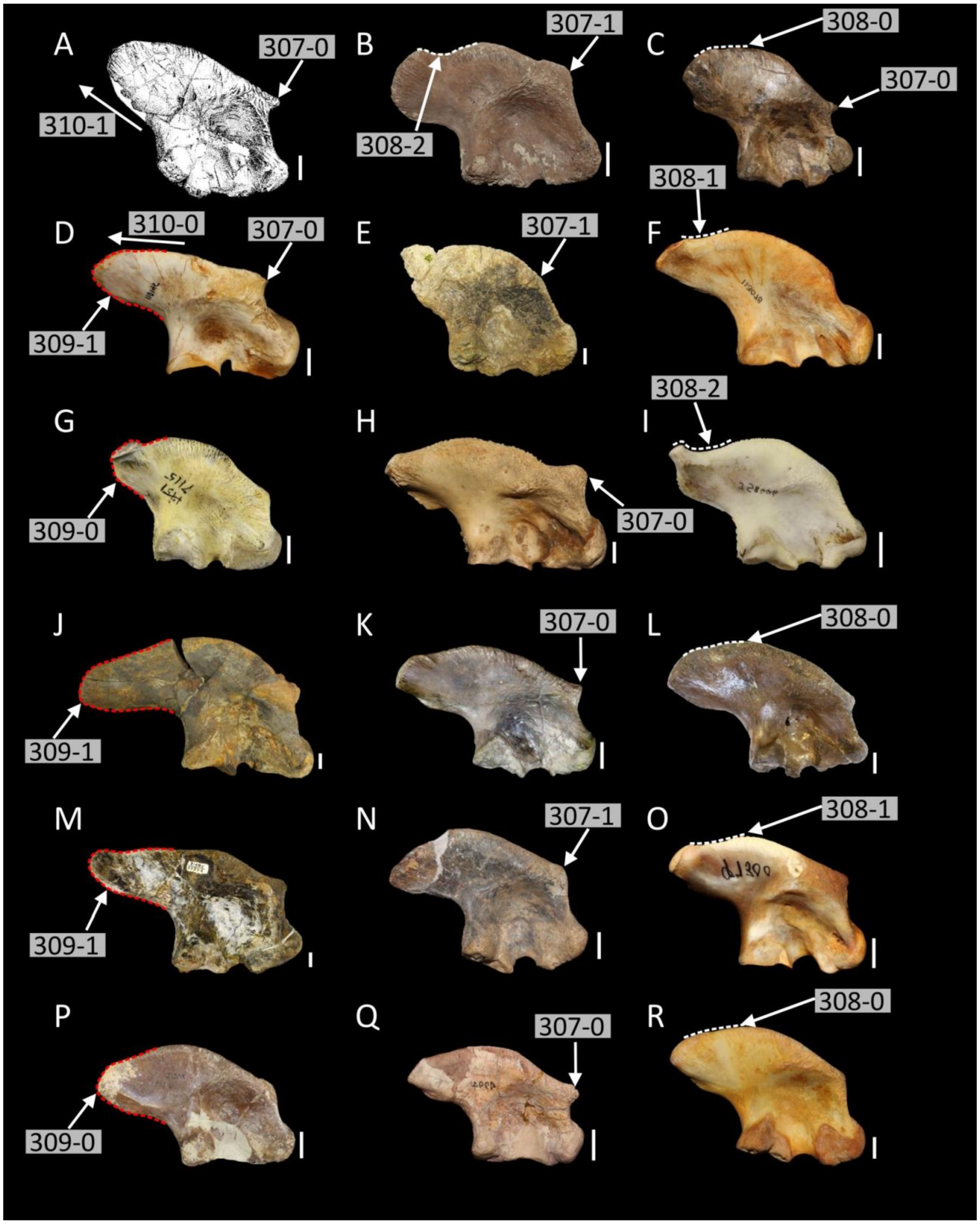

Figure 128: Variation in morphology of the ilium in Crocodylia (right ilium, lateral view). **A**, *Borealosuchus formidabilis* (Erickson, 1976: fig.27A, digitally reversed); **B**, *Diplocynodon ratelii* (MNHN uncatalogued, digitally reversed); **C**, *Diplocynodon darwini* (SMF Me-3784); **D**, *Gavialis gangeticus* (AMNH 110145, digitally reversed); **E**, *Eogavialis africanum* (NHMUK R 6199); **F**, *Tomistoma schlegelii* (AMNH 113078, digitally reversed); **G**, *Crocodylus porosus* (AMNH 7115); **H**, *Crocodylus acutus* (AMNH 7121); **I**, *Crocodylus johnstoni* (QM J 58446, digitally reversed); **J**, *Asiatosuchus germanicus* (SMF Me-1801); **K**, '*Crocodylus*' *affinis* (USNM 18171); **L**, *Asiatosuchus depressifrons* (IRSNB 9912); **M**, *Mourasuchus atopus* (UCMP 38012); **N**, *Necrosuchus ionensis* (AMNH 3219); **O**, *Caiman yacare* (AMNH 97300); **P**, *Alligator olseni* (MCZ 4719, digitally reversed); **Q**, *Alligator prenasalis* (AMNH 4994, digitally reversed); **R**, *Alligator mississippiensis* (AMNH 71621). All scale bars = 1 cm.

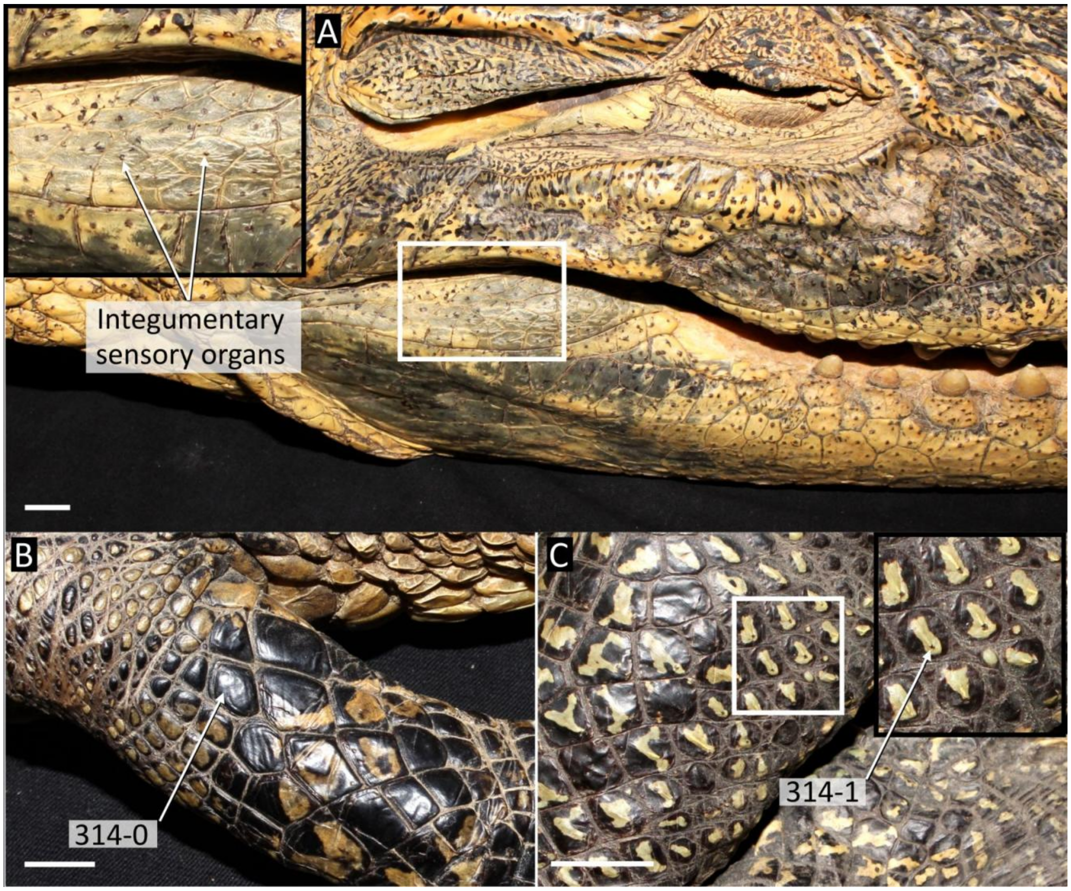

Figure 129: Variation in distribution of integumentary sensory organs in crocodylian skin. **A**, lateral view of the skull in *Crocodylus niloticus* (USNM 63592); **B**, *Alligator mississippiensis* (USNM 25148); **C**, *Crocodylus porosus* (USNM 72730). All scale bars = 2 cm.

316. Ventral collar scales: not enlarged relative to other ventral scales (0); in 1–2 enlarged rows (1) (after Poe, 1996 [115]; Brochu, 1997a [156]; adapted from Brazaitis, 1973 and Fuchs, 2006 [13]).

‘Ventral collar scales’ refer to ventral scales at the level of the forelimbs (Fuchs, 2006, fig.4). Brazaitis (1973) described different degrees of enlargement of the ventral collar scales in extant crocodylians, which were later discretised into three character states by Poe (1997, character 115): not enlarged (0); one enlarged row (1); or two enlarged rows (2). According to earlier datasets (e.g. Brochu, 1997b; Brochu et al., 2012), all extant crocodylids, *Tomistoma schlegelii*, and *Gavialis gangeticus* lack enlargement of the ventral collar scales, most alligatorids exhibit two enlarged rows, and *Paleosuchus* exhibits one enlarged row. However, these scores do not match the descriptions and illustrations in Fuchs (2006), which provides the most comprehensive account of crocodylian skins. For example, some *Crocodylus* species e.g. (*C. palustris*, *C. rhombifer*, and *C. novaeguineae*), *Osteolaemus tetraspis*, and *Tomistoma schlegelli* exhibit 1–2 enlarged rows (Fuchs, 2006). Additionally, although *Paleosuchus* exhibits one single enlarged row, so does *Caiman crocodilus* (Fuchs, 2006, fig.37). Furthermore, the distinction between 1 or 2 enlarged rows is

challenging, as scale size varies gradationally in this region. Based on these observations, the character has been simplified to distinguish between taxa that lack enlargement of the ventral collar scales (316-0) (*Gavialis gangeticus* and some *Crocodylus* species), from those with any degree of enlargement in this region (316-1) (all alligatorids, *Tomistoma schlegelii*, and most crocodylids).

317. Tail dorsal scalation, number of transverse scale rows from the level of the cloacal vent to the point of convergence of paired mid-dorsal crests into a single longitudinal crest: >13 (0); ≤13 (1) (after Wermuth, 1953; Poe, 1996 [116]; Brochu, 1997a [157]; Fuchs, 2006 [17]).

In all extant crocodylians, the dorsal scutes of the tail form paired longitudinal crests, which become more prominent posteriorly. These occur as two parallel rows on the lateral edges of the tail and converge posteriorly (Fig. 130). The point at which they converge into a single, midline longitudinal crest varies between species (Wermuth, 1953, fig.4). The number of transverse rows between the level of the cloacal vent and the point of convergence of the paired midline osteoderms, was counted in all extant crocodylians based on data in Fuchs (2006). There is a discontinuity in the data at 13 transverse rows, and thus this was used to define the character states. In general, the crests converge further anteriorly in caimanines (Fig. 130B) and *Osteolaemus* (317-1) than in all other extant crocodylians (317-0) (Fig. 130A).

Osteoderms

318. Palpebral, number of ossifications: one (0); two or more (1) (after Norell, 1988 [8]; Clark, 1994 [65]; Brochu, 1997a [96]).

319. Palpebral, size in relation to orbit: small, covering no more than half the area of the orbit (0); large, covering more than half the orbit (usually completely concealing it) (1) (new character, based on personal observations).

The crocodylian palpebral is essentially an orbital osteoderm (Vickaryous & Hall, 2008). In most extant crocodylians, this is a small, anteromedially positioned element comprising one ossification (318-0, 319-0) (Nesbitt et al., 2012). This is the case in *Alligator* (Fig. 131A), Jacarea (Fig. 131B), *Crocodylus*, *Mecistops*, *Tomistoma schlegelii*, and *Gavialis gangeticus* (Nesbitt et al., 2012). By contrast, the palpebral is composed of multiple ossifications in *Osteolaemus* (two ossifications) and *Paleosuchus* (three ossifications) (Fig. 131E) (318-1) (Brochu, 1999). Among extant crocodylians, *Paleosuchus* exhibits the largest palpebral, which almost entirely conceals the orbit (319-1), a condition also found in the 'Glen Rose Form' (MCZ 4384) and *Theriosuchus pusil-*

lus (NHMUK 48270). Although enlarged in *Osteolaemus*, the palpebral does not conceal the orbit (319-0).

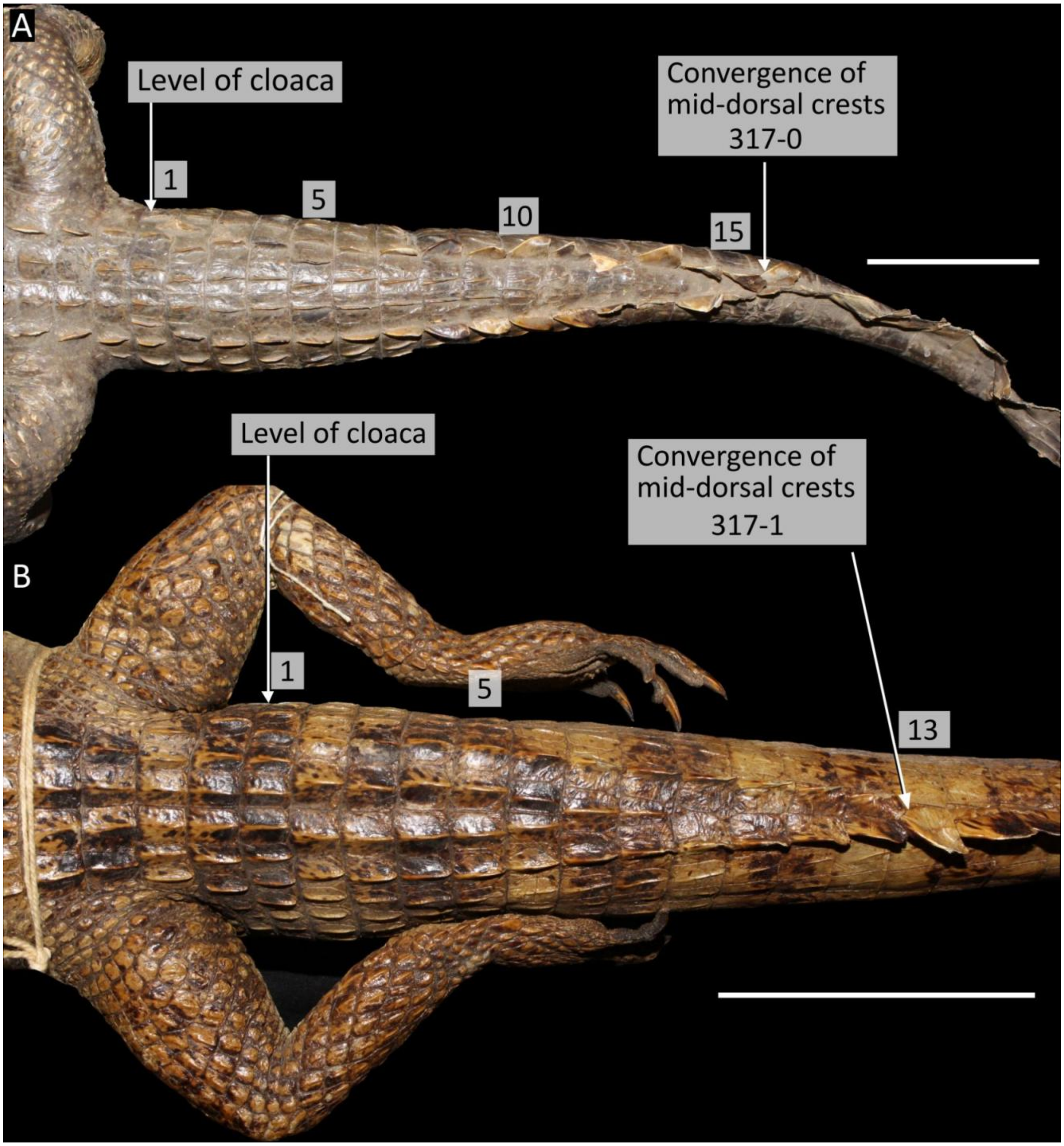

Figure 130: Dorsal view of the tail showing variation in scale crests between: **A**, *Crocodylus acutus* (USNM 52491); **B**, *Caiman crocodilus* (USNM 54094). Numbers refer to counts of the transverse scale row beginning from the level of the cloaca. All scale bars = 5 cm.

320. Tongue, lingual osmoregulatory pores: large, 1–2 mm (0); small, < 1 mm (1) (after Brochu, 1997a [158]; adapted from Taplin and Grigg, 1989).

As is the case in extant crocodylids and *Tomistoma schlegelii*, *Gavialis gangeticus* exhibits some specialisations for saltwater tolerance, e.g. a keritanised buccal cavity. *Tomistoma schlegelii* and all extant crocodylids also exhibit large osmoregulatory pores on the tongue, which secrete excess sodium chloride (Grigg & Kirshner, 2015, fig.11.23). While *Gavialis gangeticus* exhibits these pores, they are highly reduced, similar to the condition in all extant alligatorids that principally inhabit freshwater environments (320-0) (Taplin & Grigg, 1989).

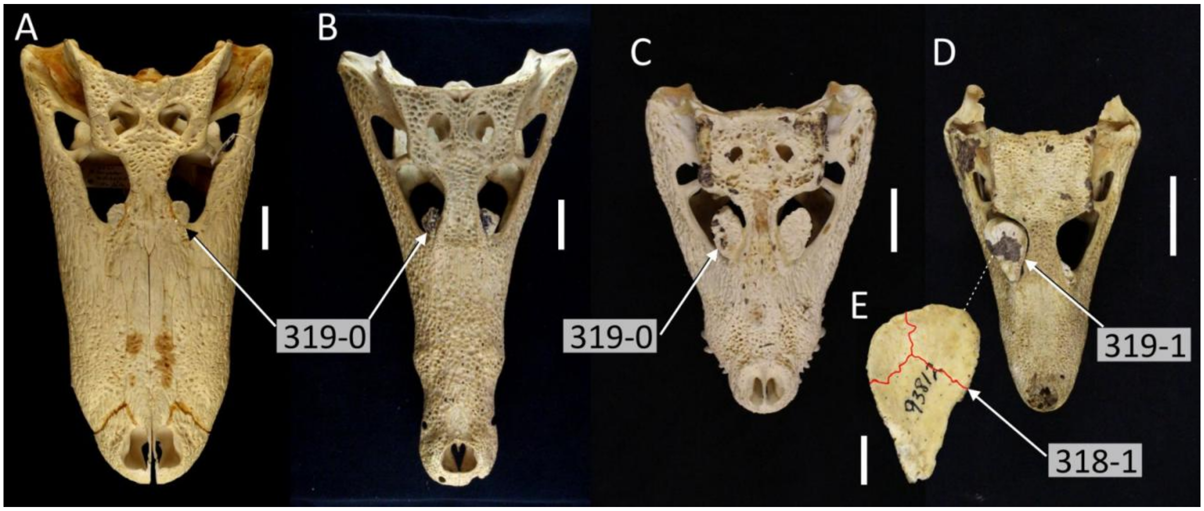

Figure 131: Variation in the morphology of the palpebral in **A**, *Alligator mississippiensis* (AMNH 71621); **B**, *Caiman crocodilus apaporiensis* (FMNH 69812); **C**, *Osteolaemus tetraspis* (AMNH 117801); **D**, *Paleosuchus palpebrosus* (AMNH 93812), **E**, enlargement of the palpebral in D in ventral view showing multiple ossifications. Scale bars A–D = 5 cm, E = 1 cm.

321. Postoccipital osteoderms, number of rows: two or more (0); one (1) (after Brochu and Storrs, 2012 [183]).

Postoccipital osteoderms are the small, anteriormost osteoderms of the nape (Fig. 132). As with the arrangement of the nuchal osteoderms (discussed below), their precise arrangement, number, and size is considerably variable in extant crocodylians (Ross & Mayer, 1983). Nevertheless, broad differences are discernible. In extant *Crocodylus*, *Gavialis gangeticus*, and *Paleosuchus palpebrosus*, there is one enlarged, transverse row of postoccipital osteoderms (321-1) (Fig. 132D–E). This differs from most caimanines, *Alligator*, *Tomistoma schlegelii* (contra Brochu & Storrs, 2012) and osteolaemines, which exhibit multiple rows of tightly packed osteoderms in this region (Fig. 132F–H). This character cannot be scored for any fossil crocodylian in this dataset.

322. Nuchal osteoderms, grade continuously into dorsal shield (0); differentiated from dorsal shield (1) (after Brochu, 1997a [38]).

323. Number of nuchal osteoderms: four (0); six (1); eight or more (2) (after Brochu, 1997a [38]) (ORDERED).

Characters 322 and 323 were derived by reductively coding character 38 in Brochu (1997b). As originally formatted, the character does not allow the consideration of the number of nuchal osteoderms in taxa with a continuous nuchal-dorsal transition (322-0), e.g. *Mecistops cataphractus* (USNM 60578), *Gavialis gangeticus* (Fig. 132I), and *Tomistoma schlegelii* (Fig. 132F). This might be considered a reasonable distinction, as determining at which point the nuchal osteoderms 'end' and the dorsal osteoderms 'start' is challenging in taxa with a continuous nuchal-dorsal transition. However, this can be achieved given that there is a one-to-one relationship between transverse osteoderm rows and vertebrae (Ross & Mayer, 1983). Following the illustrations in Ross and Mayer (1983), nuchal osteoderms were considered to be those in between the postoccipital osteoderms, and the 18th precaudal row of osteoderms (measured from the sacro-caudal junction). Accordingly, *Tomistoma schlegelii* and *Gavialis gangeticus* share the same number of osteoderms (323-2), in common with all extant caimanines (Fig. 132G–H) and *Brachychampsa montana* (UCMP 133901). All extant *Crocodylus* species, *Mecistops*, and *Alligator*, exhibit 6 nuchal osteoderms (323-1) (Fig. 132B–E), whereas *Osteolaemus* exhibits only 4 (323-0) (Fig. 132A).

324. Dorsal osteoderms, maximum number in the middle transverse row (at maturity): two (0); four (1); six (2); eight (3); ten (4) (after Norell and Clark, 1990 [12]; Clark, 1994 [97]; Brochu, 1997a [37] (ORDERED).

This character has been modified by the addition of a character state (324-0), which is observed in *Theriosuchus pusillus* (Fig. 133A), and by ordering the character. The wording is also modified such that the number of osteoderms is counted in the transverse row with the most osteoderms (usually at the anteroposterior mid-point). This difference accounts for the fact that the number of osteoderms per row decreases towards the anterior and posterior ends of the dorsal shield. For example, whereas there are ten osteoderms in the middle transverse row in *Brachychampsa montana* (324-4) (Fig. 133E), there are only four in the anteriormost row. A maximum of four dorsal osteoderms per row (324-1) occurs in *Bernissartia fagesii* (Fig. 133D), and some "gavialoids" (e.g. *Gavialis gangeticus* [Fig. 132I] and *Eosuchus minor* [USNM 321933]), as well as the "tomistomine" *Maomingosuchus petrolica* (ZMNH uncatalogued specimens on display). Most commonly there are six (324-2), as found in most extant *Crocodylus*, osteolaemines, *Alligator*, *Paleosuchus*, *Tomistoma schlegelii* (Ross & Mayer, 1983) and the 'basal' alligatoroid *Diplocynodon darwini* (Fig. 133B). Extant *Caiman*, *Melanosuchus*, and *Hassiacosuchus haupti* exhibit eight osteoderms (324-3) (Fig. 133C).

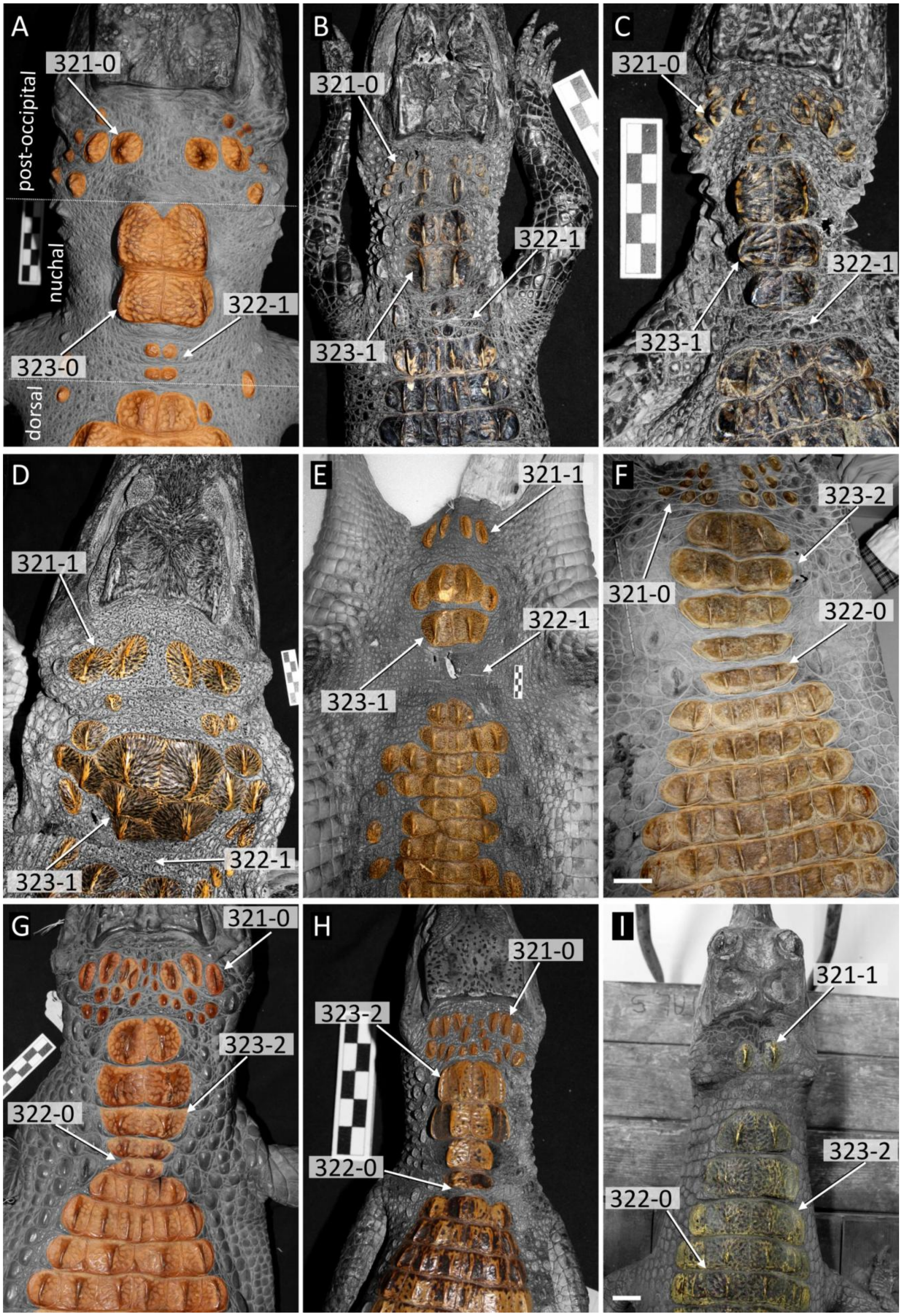

Figure 132: Morphological variation of the postoccipital and nuchal osteoderms in extant crocodylians. **A**, *Osteolaemus tetraspis* (USNM 233978); **B**, *Alligator mississippiensis* (USNM 25148); **C**, *Alligator sinensis* (USNM 67712); **D**, *Crocodylus niloticus* (USNM 63592); **E**, *Crocodylus acutus* (USNM 243433); **F**, *Tomistoma schlegelii* (FMNH uncatalogued); **G**, *Caiman latirostris* (USNM 98780); **H**, *Caiman crocodilus* (USNM 142089); **I**, *Gavialis gangeticus* (NHMUK). Scale bars in F and I = 5 cm, all other scale bars = cm.

325. Dorsal osteoderms, longitudinal midline keel: absent (0); present (1) (after Buscalioni et al., 1992 [22]; Brochu, 1997a [35]).

The anatomical meaning of this character follows that outlined by Buscalioni et al. (1992). A sagittal keel occurs on the dorsal osteoderms of *Bernissartia fagesii* (IRScNB 1538) and most eusuchians in this dataset (325-1) (Fig. 134I). By contrast, the keel is absent entirely in *Borealosuchus* and most longirostrine crocodylians, including “gavialoids” (e.g. *Eogavialis africanum* [Fig. 134], *Eosuchus lerichei* [IRScNB R 49]) and “tomistomines”, e.g. *Toyotamaphimeia* (Iijima & Kobayashi, 2019).

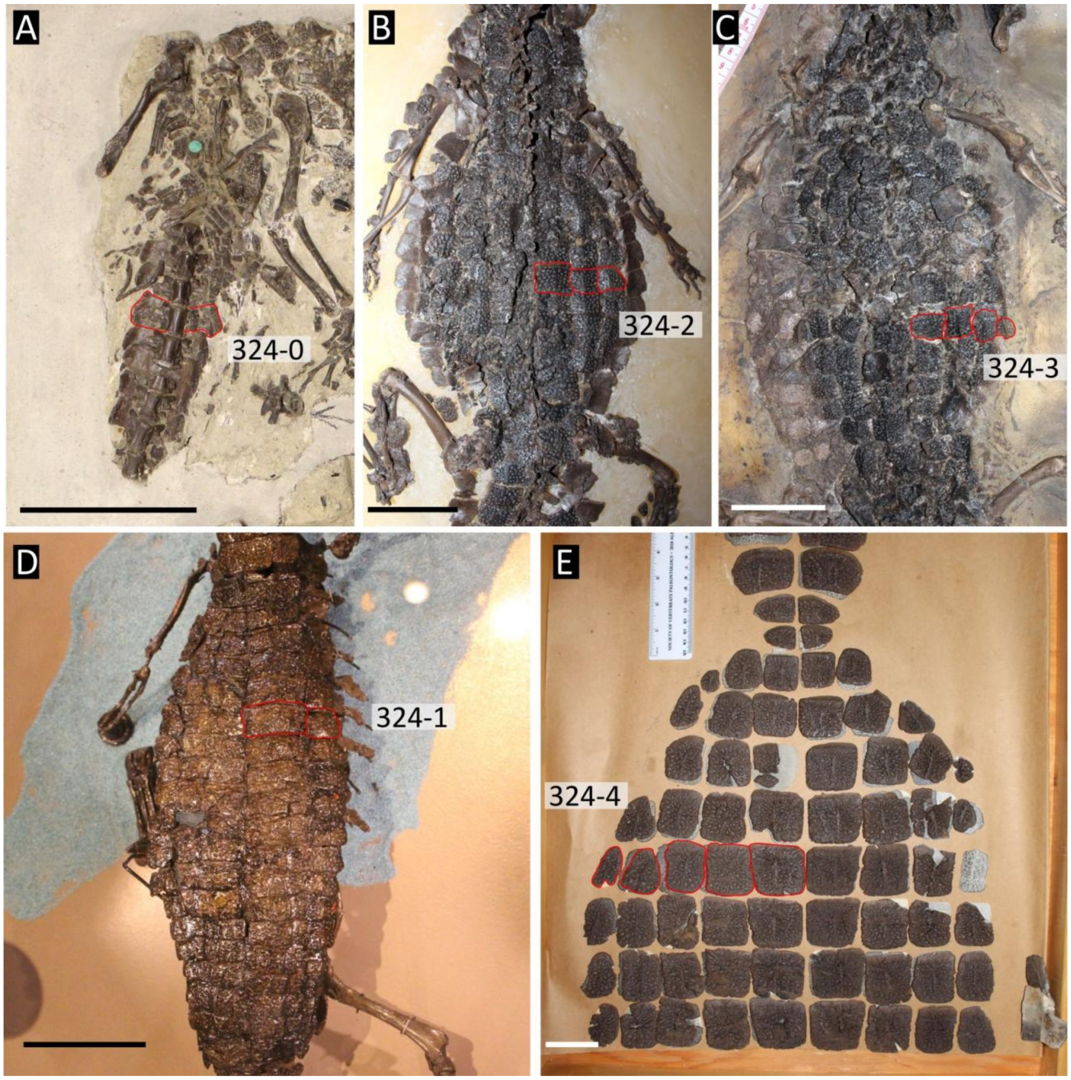

Figure 133: Variation in number of contiguous osteoderm rows. (All specimens in dorsal view except A, which is in ventral view). **A**, *Theriosuchus pusillus* (NHMUK 48216); **B**, *Diplocynodon darwini* (HLMD Me-10262); **C**, *Hassiacosuchus haupti* (HLMD Be-137); **D**, *Bernissartia fagesii* (IRSNB 1538); **E**, *Brachychampsa montana* (UCMP 133901). All scale bars = 5 cm.

326. Dorsal midline osteoderm shape: rectangular, width to length ratio $>$ 1 (0); approximately square,

width to length ratio $\leq$ 1 (1) (after Norell and Clark, 1990 [16]; Clark, 1994 [95]; Brochu, 1997a [36]).

327. Dorsal midline osteoderms, anterolateral process: present (0); absent (1) (after Norell and Clark, 1990 [31]; Clark, 1994 [96]; Brochu, 1997a [40]).

Characters 326 and 327 refer to the morphology of dorsal osteoderms in the first paravertebral (longitudinal) row, i.e. either side of the sagittal plane. This is a salient point as the shape of the osteoderms can vary along a single transverse row. These osteoderms are notably wider than long in *Bernissartia* (IRScNB 1538), *Borealosuchus* (Erickson, 1976, fig.30), and most longirostrine crocodylians, including *Gavialis gangeticus* and *Tomistoma schlegelii*. The decision was made not to treat this character continuously since osteoderm shape varies across transverse rows of the same individual, and there is uncertainty in the position of isolated fossil osteoderms.

Where present the anterolateral process of the dorsal midline osteoderms varies in morphology between taxa. In *Theriosuchus pusillus* (NHMUK 48216), this process is extremely acute, forming a peg-like process. A homologous rounded swelling at the anterolateral margin of the osteoderm also occurs in *Bernissartia* (IRScNB 1538), *Borealosuchus* (e.g. *B. sternbergii* [UCMP 134470]), "gavialoids" (e.g. *Eogavialis africanum* [NHMUK R 3343]), and some *Diplocynodon* species (Fig. 134D). This condition differs to the approximately straight anterior margin of the osteoderm in all other eusuchians where known (Fig. 134F–H).

328. Ventral osteoderms: absent (or poorly developed) (0); present, single ossification (1); present, paired ossification (2) (after Buscalioni et al., 1992 [21]; Brochu, 1997a [39]).

Uniquely among extant crocodylians, caimanines exhibit paired (bipartite) ventral osteoderms. These typically comprise a short anterior element, with an unornamented gliding surface at its anterior end, and a sutural margin on it posterior end for a larger posterior element (Fig. 134C, J). Paired ventral osteoderms occur in a few fossil crocodylians, including all *Diplocynodon* (Fig. 134E) and *Borealosuchus* species where known (Brochu, 1997a; Brochu et al., 2012). By contrast, *Bernissartia fagesii* (IRScNB 1538) exhibits single, well-formed osteoderms, as is the case in the 'basal' alligatoroids *Leidyosuchus* (Brochu, 1997a) and *Brachychampsa montana* (UCMP 133901), as well as *Alligator sinensis* (Fig. 134B) and *Crocodylus johnstoni* (Grigg & Kirshner, 2015, fig.3.15) among extant crocodylians. The remaining extant crocodylids (all further species of *Crocodylus* and *Mecistops*), *Tomistoma schlegelii*, and *Gavialis gangeticus*, exhibit poorly formed ventral osteoderms, or they are absent altogether. The poorly formed condition (328-0) is distinguished from 328-1 by the small, irregular shape of the osteoderms, e.g. (Fig. 134A).

329. Tail armour: partial covering (usually $\leq$ 10 transverse rows of contiguous osteoderms posteriorly

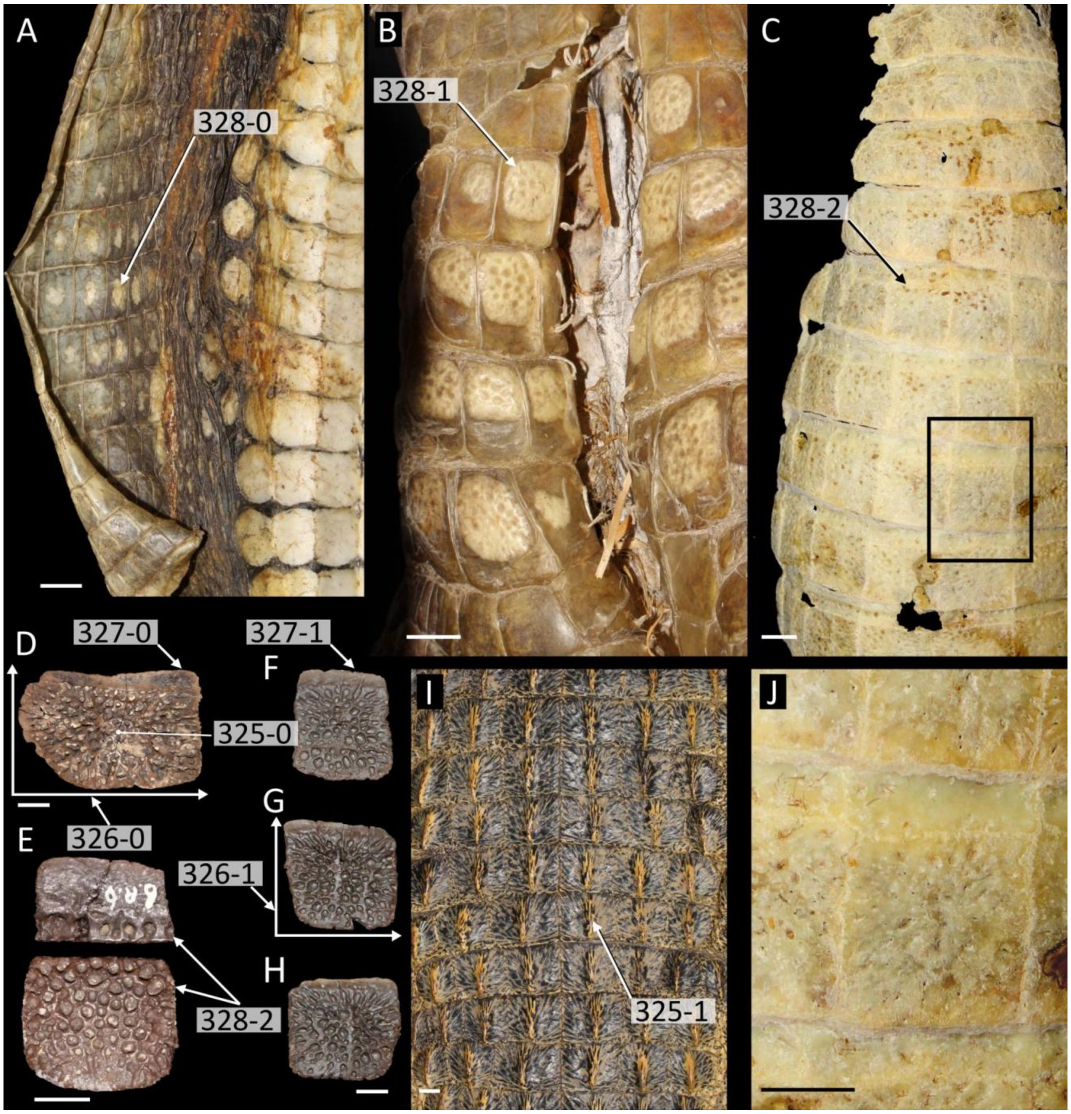

Figure 134: Morphology of the dorsal and ventral osteoderms. **A**–**B**, ventral view of the skin showing ventral osteoderms in **A**, *Mecistops cataphractus* (USNM 60578); **B**, *Alligator sinensis* (USNM 52557); **C**, ventral view of the ventral osteoderms in *Paleosuchus trigonatus* (USNM 302052); **D**, dorsal midline osteoderm of *Diplocynodon hantoniensis* (NHMUK uncatalogued); **E**, paired ventral osteoderm in *Diplocynodon hantoniensis* (NHMUK uncatalogued); **F**–**H**, dorsal midline osteoderms of *Brachychampsa montana* (UCMP 133901); **I**, dorsal osteoderms of *Crocodylus niloticus* (USNM 63592); **J**, enlargement of paired ventral osteoderms in **C**. All scale bars = 1 cm.

from the level of caudal vertebra one) (0); tail completely encased in osteoderms (1) (new character, adapted from Frey et al. 1987).

330. Limb armour: forelimbs and hindlimbs lack osteoderms, or weakly armoured with patches of poorly developed osteoderms (0); densely covered in well-formed osteoderms (1) (new character, adapted from Frey et al. 1987).

Frey et al. (1987) compared the distribution of osteoderms in *Diplocynodon* (‘*Baryphracta*’) *deponiae* with *Diplocynodon darwini*, noting differences in osteoderm extent on the tail (Character

329) and limbs (Character 330). In particular, *Diplocynodon deponiae* exhibits a tail that is completely encased in osteoderms (329-1) (Fig. 135C). This condition is found in a few other taxa in this dataset, including extant caimanines (e.g. *Paleosuchus* [Fig. 135B]), *Hassiacosuchus haupti* (Fig. 135D), *Tsoabichi greenriverensis* (FMNH PR 1793), and *Theriosuchus pusillus* (NHMUK 48216). By contrast, multiple specimens of the exceptionally preserved species *Diplocynodon darwini*, consistently lack an osteoderm-encased tail. Indeed, most specimens of this species exhibit less than ten transverse rows of contiguous osteoderms on the tail (beginning from the first caudal vertebra). Osteoderms on the remainder of the tail of this species comprise two linear arrays of pin-shaped ossifications on the dorsolateral edges (329-0) (Fig. 135A). All other extant crocodylians similarly lack a tail encased in osteoderms, along with *Bernissartia* fagesii (IRScNB 1538), *Alligator prenasalis* (YPM 13799), and *Borealosuchus wilsoni* (FMNH PR 1674).

The distribution of osteoderms on the limbs follows a very similar pattern to that described for the tail. Indeed, almost all taxa with an osteoderm-encased tail (329-1) also exhibit a dense covering of osteoderms on the limbs (330-1). *Diplocynodon darwini* proves the exception to this rule, since it lacks an osteoderm encased tail (329-0) (Fig. 135A), but exhibits a dense covering of osteoderms on the fore- and hindlimbs (330-1) (Fig. 136C–D), as in *Diplocynodon deponiae* (Fig. 136E–G).

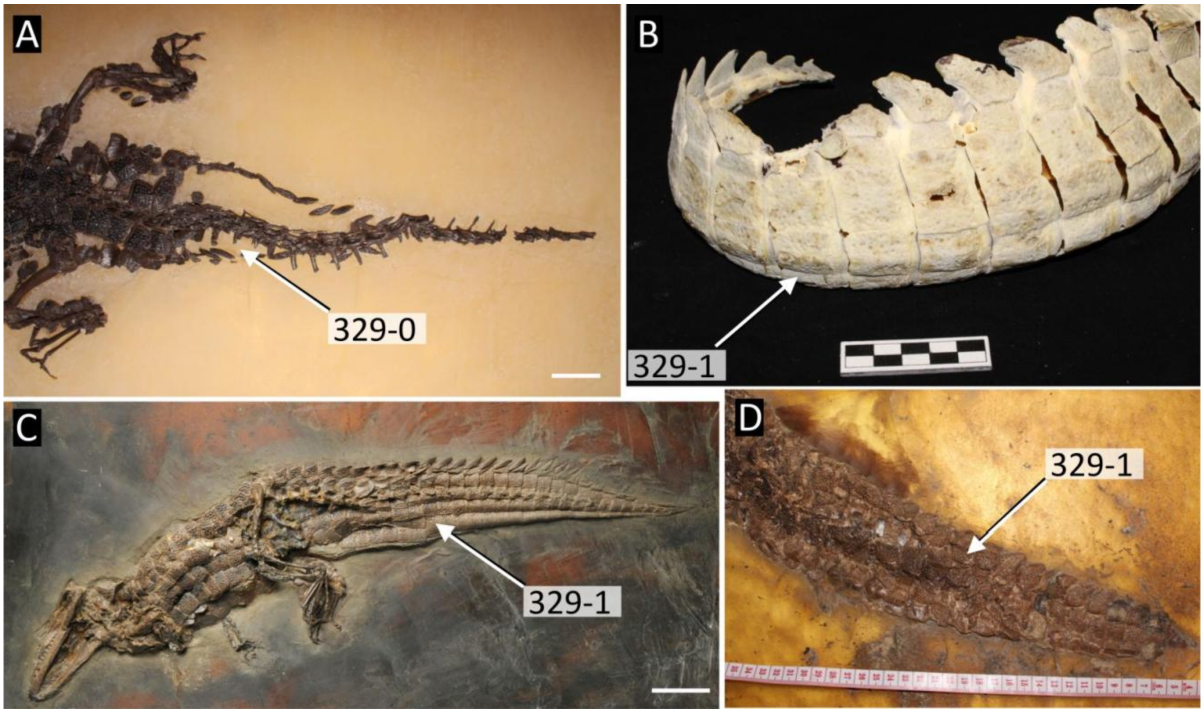

Figure 135: Variation in osteoderm arrangement in the tail of selected crocodylians: **A**, *Diplocynodon darwini* (HLMD Me-10262); **B**, *Paleosuchus trigonatus* (USNM 302052); **C**, *Diplocynodon deponiae* (SMF Me-899); **D**, *Hassiacosuchus haupti* (HLMD-Me-9119). Scale bars in A and C = 5 cm.

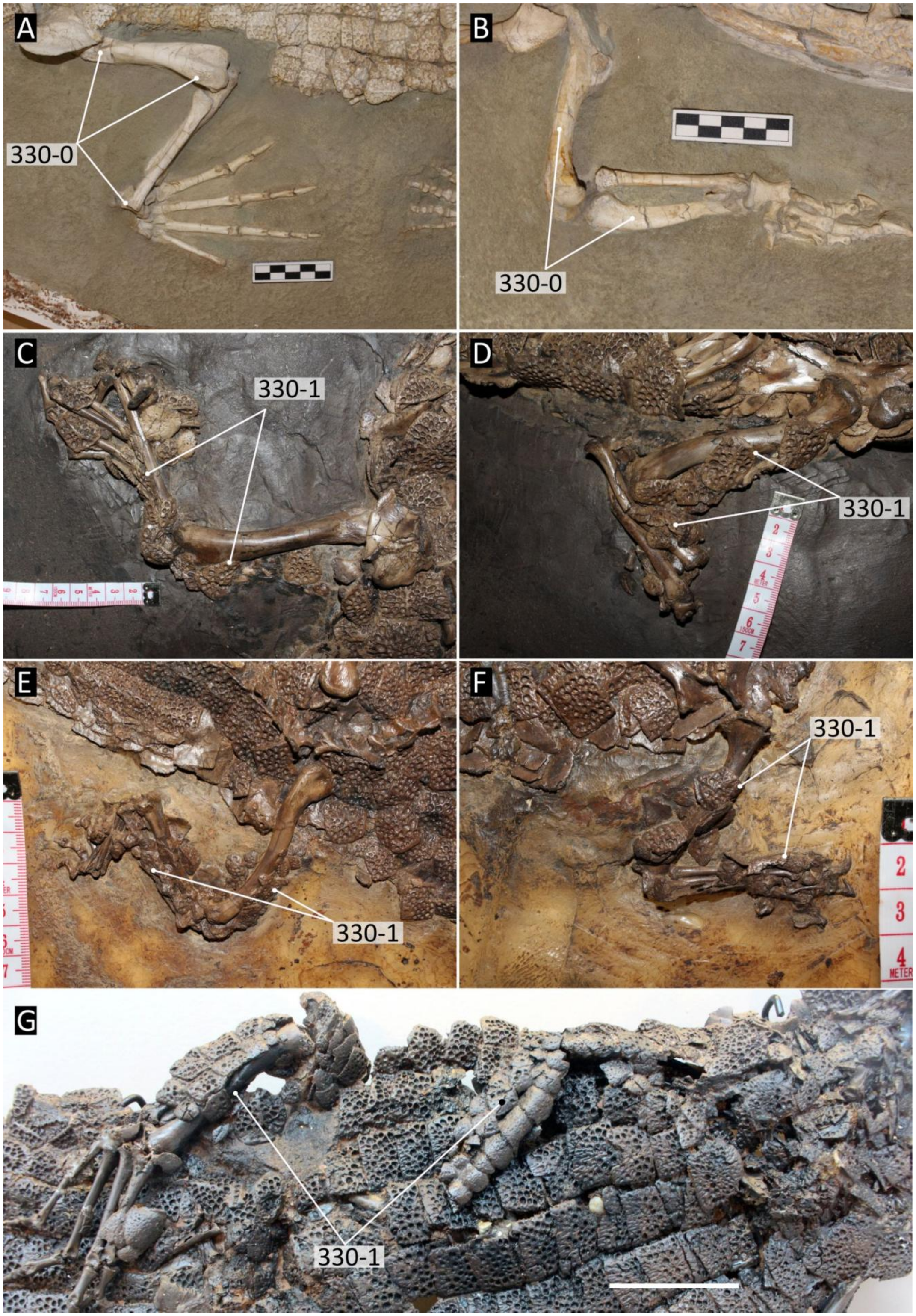

Figure 136: Variation in osteoderm cover on the hindlimb (left) and forelimb (right) (all right hand side): **A**–**B**, *Alligator prenasalis*, dorsal view (YPM 13799, digitally reversed in B); **C**–**D**, *Diplocynodon darwini*, dorsal view (HLMD Me-236); **E**–**F**, *Diplocynodon deponiae*, dorsal view (HLMD-Be-147); **G**, *Diplocynodon deponiae*, ventral view (IRScNB R261). Scale bars A–F = cm, G = 5cm.

Bibliography

Adams, T. L. (2014). Small crocodyliform from the Lower Cretaceous (Late Aptian) of Central Texas and its systematic relationship to the evolution of Eusuchia. *Journal of Paleontology*, *88*(5), 1031–1049.

Aguilera, O. A., Riff, D., & Bocquentin-Villanueva, J. (2006). A new giant *Purussaurus* (Crocodyliformes, Alligatoridae) from the upper Miocene Urumaco formation, Venezuela. *Journal of Systematic Palaeontology*, *4*(3), 221–232.

Andrade, M. B., Edmonds, R., Benton, M. J., & Schouten, R. (2011). A new Berriasian species of Goniopholis (Mesoeucrocodylia, Neosuchia) from England, and a review of the genus. *Zoological Journal of the Linnean Society*, *163*(suppl 1), S66–S108.

Antunes, M. T. (1961). *Tomistoma lusitanica, crocodilien du Miocéne du Portugal*. Revista da Faculdade de Ciencias de Lisboa.

Aoki, R. (1976). On the generic status of *Mecistops* (Crocodylidae), and the origin of *Tomistoma* and *Gavialis*. *Bulletin Atagawa Institute*, *6*(7), 23–30.

Balouet, J.-C., & Buffetaut, E. (1987). *Mekosuchus inexpectatus*, ng, n. sp., crocodilien nouveau de l'Holocene de Nouvelle Calédonie. *Comptes rendus de l'Académie des sciences. Série 2, Mécanique, Physique, Chimie, Sciences de l'univers, Sciences de la Terre*, *304*(14), 853–856.

Barrios, F. (2011). *Nuevos restos de Alligatoridae Cuvier, 1807 (Eusuchia, Crocodylia) del Neógeno del Sur de la província de Salta. Implicancias sistemáticas* (Thesis).

Baur, G. (1886). The proatlas, atlas, and axis of the Crocodilia. *American Naturalist*, *20*(3), 288–293.

Benton, M. J., & Clark, J. M. (1988). Archosaur phylogeny and the relationships of the Crocodylia. In M. J. Benton (Ed.), *The phylogeny and classification of the tetrapods* (pp. 295–338). Clarendon Press, Oxford.

Blanco, M. V., Bona, P., Olivares, A. I., & Desojo, J. B. (2015). Ontogenetic variation in the skulls of *Caiman*: the case of *Caiman latirostris* and *Caiman yacare* (Alligatoridae, Caimaninae). *The Herpetological Journal*, *25*(2), 65–73.

Bona, P. (2007). Una nueva especie de *Eocaiman* Simpson (Crocodylia, Alligatoridae) del Paleoceno Inferior de Patagonia. *Ameghiniana*, *44*(2), 435–445.

Bona, P., Carabajal, A. P., & Gasparini, Z. (2017). Neuroanatomy of *Gryposuchus neogaeus* (Crocodylia, Gavialoidea): a first integral description of the braincase and endocranial morphological variation in extinct and extant gavialoids. *Earth and Environmental Science Transactions of the Royal Society of Edinburgh*, *106*(4), 235–246.

Bona, P., Degrange, F. J., & Fernández, M. S. (2013a). Skull anatomy of the bizarre crocodylian *Mourasuchus nativus* (Alligatoridae, Caimaninae). *The Anatomical Record*, *296*(2), 227–239.

Bona, P., & Desojo, J. B. (2011). Osteology and cranial musculature of *Caiman latirostris* (Crocodylia: Alligatoridae). *Journal of Morphology*, *272*(7), 780–795.

Bona, P., Ezcurra, M. D., Barrios, F., & Fernandez Blanco, M. V. (2018). A new Palaeocene crocodylian from southern Argentina sheds light on the early history of caimanines. *Proceedings of the Royal Society B: Biological Sciences*, *285*(1885), 20180843.

Bona, P., Riff, D., & de Gasparini, Z. B. (2013b). Late Miocene crocodylians from northeast Argentina: new approaches about the austral components of the Neogene South American crocodylian fauna. *Earth and Environmental Science Transactions of the Royal Society of Edinburgh*, *103*(3-4), 551–570.

Brazaitis, P. (1973). The identification of living crocodilians. *Zoologica*, *58*(4), 59–101.

Brazaitis, P. (1987). Identification of crocodilian skins and products. In G. J. Webb, S. C. Manolis, & P. J. Whitehead (Eds.), *Wildlife management: Crocodiles and alligators* (pp. 373–386). Surrey Beatty; Sons, Sydney.

Brochu, C. A. (1995). Heterochrony in the crocodylian scapulocoracoid. *Journal of Herpetology*, *29*(3), 464–468.

Brochu, C. A. (1996). Closure of neurocentral sutures during crocodilian ontogeny: implications for maturity assessment in fossil archosaurs. *Journal of Vertebrate Paleontology*, *16*(1), 49–62.

Brochu, C. A. (1997a). A review of "*Leidyosuchus*" (Crocodyliformes, Eusuchia) from the Cretaceous through Eocene of North America. *Journal of Vertebrate Paleontology*, *17*(4), 679–697.

Brochu, C. A. (1997b). Morphology, fossils, divergence timing, and the phylogenetic relationships of *Gavialis*. *Systematic Biology*, *46*(3), 479–522.

Brochu, C. A. (1999). Phylogenetics, taxonomy, and historical biogeography of Alligatoroidea. *Journal of Vertebrate Paleontology*, *19 (Supplement to No. 2)*, 9–100.

Brochu, C. A. (2000). Phylogenetic relationships and divergence timing of *Crocodylus* based on morphology and the fossil record. *Copeia*, *2000*(3), 657–673.

Brochu, C. A. (2003). Phylogenetic approaches toward crocodylian history. *Annual Review of Earth and Planetary Sciences*, *31*(1), 357–397.

Brochu, C. A. (2004a). A new Late Cretaceous gavialoid crocodylian from eastern North America and the phylogenetic relationships of thoracosaurs. *Journal of Vertebrate Paleontology*, *24*(3), 610–633.

Brochu, C. A. (2004b). Alligatorine phylogeny and the status of *Allognathosuchus* Mook, 1921. *Journal of Vertebrate Paleontology*, *24*(4), 857–873.

Brochu, C. A. (2006a). *Eosuchus* (Crocodylia, Gavialoidea) from the Lower Eocene of the Isle of Sheppey, England. *Journal of Vertebrate Paleontology*, *26*(2), 466–470.

Brochu, C. A. (2006b). Osteology and phylogenetic significance of *Eosuchus minor* (Marsh, 1870) new combination, a longirostrine crocodylian from the late Paleocene of North America. *Journal of Paleontology*, *80*(1), 162–186.

Brochu, C. A. (2007a). Morphology, relationships, and biogeographical significance of an extinct horned crocodile (Crocodylia, Crocodylidae) from the Quaternary of Madagascar. *Zoological Journal of the Linnean Society*, *150*(4), 835–863.

Brochu, C. A. (2007b). Systematics and taxonomy of Eocene tomistomine crocodylians from Britain and Northern Europe. *Palaeontology*, *50*(4), 917–928.

Brochu, C. A. (2010). A new alligatorid from the lower Eocene Green River Formation of Wyoming and the origin of caimans. *Journal of Vertebrate Paleontology*, *30*(4), 1109–1126.

Brochu, C. A. (2011). Phylogenetic relationships of *Necrosuchus ionensis* Simpson, 1937 and the early history of caimanines. *Zoological Journal of the Linnean Society*, *163*, S228–S256.

Brochu, C. A. (2012). Phylogenetic relationships of Palaeogene ziphodont eusuchians and the status of *Pristichampsus* Gervais, 1853. *Earth and Environmental Science Transactions of the Royal Society of Edinburgh*, *103*(3-4), 521–550.

Brochu, C. A., & Gingerich, P. D. (2000). New tomistomine crocodylian from the middle Eocene (Bartonian) of Wadi Hitan, Fayum Province, Egypt. *Contributions from the Mueum of Paleontology, The University of Michigan*, *30*(10), 251–268.

Brochu, C. A., Njau, J., Blumenschine, R. J., & Densmore, L. D. (2010). A new horned crocodile from the Plio-Pleistocene hominid sites at Olduvai Gorge, Tanzania. *PLoS One*, *5*(2), e9333.

Brochu, C. A., Parris, D. C., Grandstaff, B. S., Denton Jr, R. K., & Gallagher, W. B. (2012). A new species of *Borealosuchus* (Crocodyliformes, Eusuchia) from the Late Cretaceous–early Paleogene of New Jersey. *Journal of Vertebrate Paleontology*, *32*(1), 105–116.

Brochu, C. A., & Storrs, G. W. (2012). A giant crocodile from the Plio-Pleistocene of Kenya, the phylogenetic relationships of Neogene African crocodylines, and the antiquity of *Crocodylus* in Africa. *Journal of Vertebrate Paleontology*, *32*(3), 587–602.

Buchanan, L. A. (2009). *Kambara taraina* sp. nov. (Crocodylia, Crocodyloidea), a new Eocene mekosuchine from Queensland, Australia, and a revision of the genus. *Journal of Vertebrate Paleontology*, *29*(2), 473–486.

Buffetaut, E. (1985). The place of *Gavialis* and *Tomistoma* in Eusuchian evolution: a reconciliation of palaeontological and biochemical data. *Neues Jahrbuch für Geologie und Paläontologie. Monatshefte*, (12), 707–716.

Buscalioni, A., Ortega, F., Weishampel, D., & Jianu, C. (2001). A revision of the crocodyliform *Allodaposuchus precedens* from the Upper Cretaceous of the Hateg Basin, Romania. Its relevance in the phylogeny of Eusuchia. *Journal of Vertebrate Paleontology*, *21*(1), 74–86.

Buscalioni, A. D., Ortega, F., & Vasse, D. (1997). New crocodiles (Eusuchia: Alligatoroidea) from the Upper Cretaceous of southern Europe. *Comptes Rendus de l'Académie des Sciences. Series IIA, Earth and Planetary Science*, *325*(7), 525–530.

Buscalioni, A. D., Piras, P., Vullo, R., Signore, M., & Barbera, C. (2011). Early Eusuchia rocodylomorpha from the vertebrate-rich Plattenkalk of Pietraroia (Lower Albian, southern Apennines, Italy). *Zoological Journal of the Linnean Society*, *163*, S199–S227.

Buscalioni, A. D., Sanz, J. L., & Casanovas, M. L. (1992). A new species of the eusuchian crocodile *Diplocynodon* from the Eocene of Spain. *Neues Jahrbuch für Geologie und Paläontologie Abhandlungen*, *187*, 1–29.

Cautley, P. T., & Falconer, H. (1840). XXXIV.–Notice on the Remains of a Fossil Monkey from the Tertiary Strata of the Sewalik Hills in the North of Hindoostan. *Transactions of the Geological Society of London*, *2*(3), 499–504.

Cidade, G. M., Fortier, D., Rincon, A. D., & Hsiou, A. S. (2019a). Taxonomic review of two fossil crocodylians from the Cenozoic of South America and its implications for the crocodylian fauna of the continent. *Zootaxa*, *4656*(3), 475–486.

Cidade, G. M., Solórzano, A., Rincón, A. D., Riff, D., & Hsiou, A. S. (2017). A new *Mourasuchus* (Alligatoroidea, Caimaninae) from the late Miocene of Venezuela, the phylogeny of Caimaninae and considerations on the feeding habits of *Mourasuchus*. *PeerJ*, *5*, e3056.

Cidade, G. M., Solórzano, A., Rincón, A. D., Riff, D., & Hsiou, A. S. (2018). Redescription of the holotype of the Miocene crocodylian *Mourasuchus arendsi* (Alligatoroidea, Caimaninae) and perspectives on the taxonomy of the species. *Historical Biology*, 1–17. https://doi.org/10.1080/08912963.2018.1528246

Cidade, G. M., Souza-Filho, J. P., Hsiou, A. S., Brochu, C. A., & Riff, D. (2019b). New specimens of *Mourasuchus* (Alligatoroidea, Caimaninae) from the Miocene of Brazil and Bolivia and their taxonomic and morphological implications. *Alcheringa: An Australasian Journal of Palaeontology*, *43*(2), 261–278.

Clark, J. M. (1994). Patterns of evolution in Mesozoic Crocodyliformes. In N. Fraser & H.-D. Sues (Eds.), *In the shadow of the dinosaurs: Early mesozoic tetrapods* (pp. 84–97). New York, Cambridge University Press.

Clark, J. M., & Norell, M. (1992). The Early Cretaceous crocodylomorph *Hylaeochampsa vectiana* from the wealden of the Isle of Wight. *American Museum Novitates*, *3032*, 1–19.

Cossette, A. P., & Brochu, C. A. (2018). A new specimen of the alligatoroid *Bottosaurus harlani* and the early history of character evolution in alligatorids. *Journal of Vertebrate Paleontology*, *38*(4), (1)–(22).

Delfino, M., Codrea, V., Folie, A., Dica, P., Godefroit, P., & Smith, T. (2008a). A complete skull of *Allodaposuchus precedens* Nopcsa, 1928 (Eusuchia) and a reassessment of the morphology of the taxon based on the Romanian remains. *Journal of Vertebrate Paleontology*, *28*(1), 111–122.

Delfino, M., & De Vos, J. (2010). A revision of the Dubois crocodylians, *Gavialis bengawanicus* and *Crocodylus ossifragus*, from the Pleistocene *Homo erectus* beds of Java. *Journal of Vertebrate Paleontology*, *30*(2), 427–441.

Delfino, M., Martin, J. E., & Buffetaut, E. (2008b). A new species of *Acynodon* (Crocodylia) from the upper cretaceous (Santonian–Campanian) of Villaggio del Pescatore, Italy. *Palaeontology*, *51*(5), 1091–1106.

Delfino, M., Martin, J. E., de Lapparent de Broin, F., & Smith, T. (2019). Evidence for a pre-PETM dispersal of the earliest European crocodyloids. *Historical Biology*, *31*(7), 845–852.

Delfino, M., Piras, P., & Smith, T. (2005). Anatomy and phylogeny of the gavialoid crocodylian *Eosuchus lerichei* from the Paleogene of Europe. *Acta Palaeontologica Polonica*, *50*(3), 565–580.

Delfino, M., & Smith, T. (2009). A reassessment of the morphology and taxonomic status of '*Crocodylus*' *depressifrons* (Crocodylia, Crocodyloidea) based on the Early Eocene remains from Belgium. *Zoological Journal of the Linnean Society*, *156*(1), 140–167.

Delfino, M., & Smith, T. (2012). Reappraisal of the morphology and phylogenetic relationships of the middle Eocene alligatoroid *Diplocynodon deponiae* (Frey, Laemmert, and Riess, 1987) based on a three-dimensional specimen. *Journal of Vertebrate Paleontology*, *32*(6), 1358–1369.

Duméril, C. (1806). *Zoologie analytique, ou Méthode naturelle de classification des animaux: rendue plus facile à l'aide de tableaux synoptiques*. Allais.

Erickson, B. R. (1976). *Osteology of the early eusuchian crocodile Leidyosuchus formidabilis, sp. nov* (Vol. 2). Monographs of the Science Museum of Minnesota (Paleontology).

Erickson, B. R. (1982). *Wannaganosuchus*, a new alligator from the Paleocene of North America. *Journal of Paleontology*, 492–506.

Frey, E., Laemmert, A., & Riess, J. (1987). *Baryphracta deponiae* n. g. n. sp. (Reptilia, Crocodylia), ein neues Krokodil aus der Grube Messel bey Darmstadt (Hessen, Bundesrepublik Deutschland). *Neues Jahrbuch für Geologie und Paläontologie, Monatschefte*, *1987*, 15–26.

Fuchs, K. (2006). *The crocodile skin: important characteristics in identifying crocodilian species*. Frankfurt, Andreas S. Brahm.

Gilmore, C. W. (1911). A new fossil alligator from the Hell Creek beds of Montana. *Proceedings of the United States National Museum*, *41*(1860), 297–302.

Gomani, E. M. (1997). A crocodyliform from the Early Cretaceous dinosaur beds, northern Malawi. *Journal of Vertebrate Paleontology*, *17*(2), 280–294.

Grigg, G., & Kirshner, D. (2015). *Biology and evolution of crocodylians*. Cornell University Press.

Groh, S. S., Upchurch, P., Barrett, P. M., & Day, J. J. (2020). The phylogenetic relationships of neosuchian crocodiles and their implications for the convergent evolution of the longirostrine condition. *Zoological Journal of the Linnean Society*, *188*(2), 473–506.

Harshman, J., Huddleston, C. J., Bollback, J. P., Parsons, T. J., & Braun, M. J. (2003). True and false gharials: a nuclear gene phylogeny of Crocodylia. *Systematic Biology*, *52*(3), 386–402.

Hastings, A. K., Bloch, J. I., Cadena, E. A., & Jaramillo, C. A. (2010). A new small short-snouted dyrosaurid (Crocodylomorpha, Mesoeucrocodylia) from the Paleocene of Northeastern Colombia. *Journal of Vertebrate Paleontology*, *30*(1), 139–162.

Holliday, C. M., Tsai, H. P., Skiljan, R. J., George, I. D., & Pathan, S. (2013). A 3D interactive model and atlas of the jaw musculature of *Alligator mississippiensis*. *PLoS One*, *8*(6), e62806.

Holliday, C. M., & Witmer, L. M. (2009). The epipterygoid of crocodyliforms and its significance for the evolution of the orbitotemporal region of eusuchians. *Journal of Vertebrate Paleontology*, *29*(3), 715–733.

Hua, S., & Jouve, S. (2004). A primitive marine gavialoid from the Paleocene of Morocco. *Journal of Vertebrate Paleontology*, *24*(2), 341–350.

Huxley, T. H. (1875). On *Stagonolepis robertsoni*, and on the Evolution of the Crocodilia. *Quarterly Journal of the Geological Society*, *31*(1-4), 423–438.

Iijima, M., & Kubo, T. (2019a). Allometric growth of limb and body proportions in crocodylians. *Journal of Zoology*, *309*(3), 200–211.

Iijima, M., & Kobayashi, Y. (2019). Mosaic nature in the skeleton of East Asian crocodylians fills the morphological gap between "Tomistominae" and Gavialinae. *Cladistics*, *35*(6), 623–632.

Iijima, M., & Kubo, T. (2019b). Comparative morphology of presacral vertebrae in extant crocodylians: taxonomic, functional and ecological implications. *Zoological Journal of the Linnean Society*, *186*(4), 1006–1025.

Iijima, M., Kubo, T., & Kobayashi, Y. (2018). Comparative limb proportions reveal differential locomotor morphofunctions of alligatoroids and crocodyloids. *Royal Society Open Science*, *5*(3), 171774.

Iordansky, N. N. (1973). The skull of the Crocodilia. In C. Gans & T. S. Parsons (Eds.), *Biology of the Reptilia* (pp. 201–262). Academic Press, London.

Jouve, S. (2004). *Etude des Crocodyliformes fini Crétacé-Paléogène du Bassin des Oulad Abdoun (Maroc) et comparaison avec les faunes africaines contemporaines: systématique, phylogénie et paléobiogéographie* (Thesis).

Jouve, S. (2009). The skull of *Teleosaurus cadomensis* (Crocodylomorpha; Thalattosuchia), and phylogenetic analysis of Thalattosuchia. *Journal of Vertebrate Paleontology*, *29*(1), 88–102.

Jouve, S. (2016). A new basal tomistomine (Crocodylia, Crocodyloidea) from Issel (Middle Eocene; France): palaeobiogeography of basal tomistomines and palaeogeographic consequences. *Zoological Journal of the Linnean Society*, *177*(1), 165–182.

Jouve, S., Bardet, N., Jalil, N.-E., Suberbiola, X. P., Bouya, B., & Amaghzaz, M. (2008). The oldest African crocodylian: phylogeny, paleobiogeography, and differential survivorship of marine reptiles through the Cretaceous-Tertiary boundary. *Journal of Vertebrate Paleontology*, *28*(2), 409–421.

Jouve, S., Bouya, B., Amaghzaz, M., & Meslouh, S. (2015). *Maroccosuchus zennaroi* (Crocodylia: Tomistominae) from the Eocene of Morocco: Phylogenetic and palaeobiogeographical implications of the basalmost tomistomine. *Journal of Systematic Palaeontology*, *13*(5), 421–445.

Jouve, S., Iarochene, M., Bouya, B., & Amaghzaz, M. (2006a). A new species of *Dyrosaurus* (Crocodylomorpha, Dyrosauridae) from the early Eocene of Morocco: phylogenetic implications. *Zoological Journal of the Linnean Society*, *148*(4), 603–656.

Jouve, S., Iarochene, M., Bouya, B., & Amaghzaz, M. (2006b). New material of *Argochampsa krebsi* (Crocodylia: Gavialoidea) from the Lower Paleocene of the Oulad Abdoun Basin (Morocco): phylogenetic implications. *Geobios*, *39*(6), 817–832.

Kälin, J. A. (1933). Beiträge zur vergleichenden Osteologie des Crocodilidenschädels. *Zoologische Jahrbücher*, *57*, 535–714.

Kobayashi, Y., Tomida, Y., Kamei, T., & Eguchi, T. (2006). Anatomy of a Japanese tomistomine crocodylian, *Toyotamaphimeia machikanensis* (Kamei et Matsumoto, 1965), from the middle Pleistocene of Osaka Prefecture: the reassessment of its phylogenetic status within Crocodylia. *National Science Museum Monographs*, *35*, 1–121.

Kraus, R. (1998). The cranium of *Piscogavialis jugaliperforatus* n. gen., n. sp. (Gavialidae, Crocodylia) from the Miocene of Peru. *Paläontologische Zeitschrift*, *72*(3-4), 389–405.

Lee, M. S. Y., & Yates, A. M. (2018). Tip-dating and homoplasy: reconciling the shallow molecular divergences of modern gharials with their long fossil record. *Proceedings of the Royal Society B: Biological Sciences*, *285*(1881), 20181071.

Li, C., Wu, X.-C., & Rufolo, S. J. (2019). A new crocodyloid (Eusuchia: Crocodylia) from the Upper Cretaceous of China. *Cretaceous Research*, *94*, 25–39.

Ludwig, R. (1877). *Fossile Crocodiliden aus der Tertiärformation des mainzer Beckens* (Vol. 3). Paleontographica Supplement.

Martin, B. G. H., & Bellairs, A. d. (1977). The narial excrescence and pterygoid bulla of the gharial, *Gavialis gangeticus* (Crocodilia). *Journal of Zoology*, *182*(4), 541–558.

Martin, J. E. (2007). New material of the Late Cretaceous globidontan *Acynodon iberoccitanus* (Crocodylia) from southern France. *Journal of Vertebrate Paleontology*, *27*(2), 362–372.

Martin, J. E. (2019). The taxonomic content of the genus *Gavialis* from the Siwalik Hills of India and Pakistan. *Papers in Palaeontology*, *5*(3), 483–497.

Martin, J. E., Buffetaut, E., Naksri, W., Lauprasert, K., & Claude, J. (2012). *Gavialis* from the Pleistocene of Thailand and its relevance for drainage connections from India to Java. *PloS one*, *7*(9), e44541.

Martin, J. E., Delfino, M., Garcia, G., Godefroit, P., Berton, S., & Valentin, X. (2016). New specimens of *Allodaposuchus precedens* from France: intraspecific variability and the diversity of European Late Cretaceous eusuchians. *Zoological Journal of the Linnean Society*, *176*(3), 607–631.

Martin, J. E., Smith, T., de Lapparent de Broin, F., Escuillié, F., & Delfino, M. (2014). Late Palaeocene eusuchian remains from Mont de Berru, France, and the origin of the alligatoroid *Diplocynodon*. *Zoological Journal of the Linnean Society*, *172*(4), 867–891.

Martin, J. E., Smith, T., Salaviale, C., Adrien, J., & Delfino, M. (2020). Virtual reconstruction of the skull of *Bernissartia fagesii* and current understanding of the neosuchian–eusuchian transition. *Journal of Systematic Palaeontology*, 1–23.

Mateus, O., Puértolas-Pascual, E., & Callapez, P. M. (2019). A new eusuchian crocodylomorph from the Cenomanian (Late Cretaceous) of Portugal reveals novel implications on the origin of Crocodylia. *Zoological Journal of the Linnean Society*, *186*(2), 501–528.

Medem, F. (1958). The crocodilian genus *Paleosuchus*. *Fieldiana Zoology*, *39*.

Meers, M. B. (2003). Crocodylian forelimb musculature and its relevance to Archosauria. *The Anatomical Record Part A: Discoveries in Molecular, Cellular, and Evolutionary Biology: An Official Publication of the American Association of Anatomists*, *274*(2), 891–916.

Megirian, D. (1994). A new species of *Quinkana* (Molnar) (Eusuchia: Crocodylidae) from the Miocene Camfield beds of Northern Australia. *The Beagle: Records of the Museums and Art Galleries of the Northern Territory*, *11*, 145–166.

Molnar, R. E. (1981). Pleistocene ziphodont crocodilians of Queensland. *Records of the Australian Museum*, *33*(19), 803–834.

Molnar, R. E. (1982). Cenozoic fossil reptiles in Australia. *The Fossil Vertebrate Record of Australia'.(Eds P V Rich and E M Thompson.) pp*, 228–33.

Montefeltro, F. C., Larsson, H. C. E., de França, M. A. G., & Langer, M. C. (2013). A new neosuchian with Asian affinities from the Jurassic of northeastern Brazil. *Naturwissenschaften*, *100*(9), 835–841.

Mook, C. C. (1921). Notes on the postcranial skeleton in the Crocodilia. *Bulletin of American Museum of Natural History*, *44*, 67–100.

Mook, C. C. (1932). A new species of fossil gavial from the Siwalik beds. *American Museum Novitates*, *514*, 1–5.

Mook, C. C. (1959). A new species of fossil crocodile of the genus *Leidyosuchus* from the Green River beds. *American Museum Novitates*, *1933*, 1–6.

Narváez, I., Brochu, C. A., Escaso, F., Pérez-García, A., & Ortega, F. (2016). New Spanish Late Cretaceous eusuchian reveals the synchronic and sympatric presence of two allodaposuchids. *Cretaceous Research*, *65*, 112–125.

Narváez, I., Brochu, C. A., De Celis, A., Codrea, V., Escaso, F., Pérez-García, A., & Ortega, F. (2019). New diagnosis for *Allodaposuchus precedens*, the type species of the European Upper Cretaceous clade Allodaposuchidae. *Zoological Journal of the Linnean Society*, *189*(2), 618–634.

Narváez, I., Brochu, C. A., Escaso, F., Pérez-García, A., & Ortega, F. (2015). New crocodyliforms from southwestern Europe and definition of a diverse clade of European Late Cretaceous basal eusuchians. *PLoS One*, *10*(11), e0140679.

Nesbitt, S. J., Turner, A. H., & Weinbaum, J. C. (2012). A survey of skeletal elements in the orbit of Pseudosuchia and the origin of the crocodylian palpebral. *Earth and Environmental Science Transactions of the Royal Society of Edinburgh*, *103*(3–4), 365–381.

Norell, M. A., & Clark, J. M. (1990). A reanalysis of *Bernissartia fagesii*, with comments on its phylogenetic position and its bearing on the origin and diagnosis of the Eusuchia. *Bulletin de l'Institut Royal des Sciences Naturelles de Belgique*, *60*, 115–128.

Norell, M. A. (1988). *Cladistic approaches to paleobiology as applied to the phylogeny of alligatorids* (Thesis).

Norell, M. A. (1989). The higher level relationships of the extant Crocodylia. *Journal of Herpetology*, *23*(4), 325–335.

Norell, M., Clark, J. M., & Hutchison, J. H. (1994). The Late Cretaceous alligatoroid *Brachychampsa montana* (Crocodylia): new material and putative relationships. *American Museum Novitates*, *3116*, 1–26.

Ortega, F., Gasparini, Z., Buscalioni, A. D., & Calvo, J. O. (2000). A new species of *Araripesuchus* (Crocodylomorpha, Mesoeucrocodylia) from the lower Cretaceous of Patagonia (Argentina). *Journal of Vertebrate Paleontology*, *20*(1), 57–76.

Ösi, A. (2008). Cranial osteology of *Iharkutosuchus makadii*, a Late Cretaceous basal eusuchian crocodyliform from Hungary. *Neues Jahrbuch für Geologie und Paläontologie-Abhandlungen*, *248*(3), 279–299.

Ösi, A., Clark, J. M., & Weishampel, D. B. (2007). First report on a new basal eusuchian crocodyliform with multicusped teeth from the Upper Cretaceous (Santonian) of Hungary. *Neues Jahrbuch für Geologie und Paläontologie-Abhandlungen*, *243*(2), 169–177.

Pinheiro, A. E. P., Fortier, D. C., Pol, D., Campos, D. A., & Bergqvist, L. P. (2013). A new *Eocaiman* (Alligatoridae, Crocodylia) from the Itaboraí Basin, Paleogene of Rio de Janeiro, Brazil. *Historical Biology*, *25*(3), 327–337.

Piras, P., & Buscalioni, A. D. (2006). *Diplocynodon muelleri* comb. nov., an Oligocene diplocynodontine alligatoroid from Catalonia (Ebro Basin, Lleida province, Spain). *Journal of Vertebrate Paleontology*, *26*(3), 608–620.

Poe, S. (1997). Data set incongruence and the phylogeny of crocodilians. *Systematic Biology*, *45*(4), 393–414.

Pol, D., & Norell, M. (2004). A new crocodyliform from Zos Canyon, Mongolia. *American Museum Novitates*, *3445*, 1–36.

Pol, D., Turner, A. H., & Norell, M. A. (2009). Morphology of the Late Cretaceous crocodylomorph *Shamosuchus djadochtaensis* and a discussion of neosuchian phylogeny as related to the origin of Eusuchia. *Bulletin of the American Museum of Natural History*, *324*, 1–104.

Price, L. I. (1964). Sôbre o crânio de um grande crocodilídeo extinto do Alto Rio Juruá, Estado do Acre. *Anais da Academia Brasileira de Ciências*, *56*, 59–66.

Riff, D., & Aguilera, O. A. (2008). The world's largest gharials *Gryposuchus*: description of *G. croizati* n. sp. (Crocodylia, Gavialidae) from the Upper Miocene Urumaco Formation, Venezuela. *Paläontologische Zeitschrift*, *82*(2), 178–195.

Rio, J. P., Mannion, P. D., Tschopp, E., Martin, J. E., & Delfino, M. (2020). Reappraisal of the morphology and phylogenetic relationships of the alligatoroid crocodylian *Diplocynodon hantoniensis* from the late Eocene of the United Kingdom. *Zoological Journal of the Linnean Society*, *188*(2), 579–629.

Ross, F. D., & Mayer, G. C. (1983). On the dorsal armor of the Crocodilia. In A. G. Rhodin & K. Miyata (Eds.), *Advances in herpetology and evolutionary biology* (pp. 305–331). Cambridge, Massachusetts, Harvard University Press.

Salas-Gismondi, R., Flynn, J. J., Baby, P., Tejada-Lara, J. V., Claude, J., & Antoine, P.-O. (2016). A new 13 million year old gavialoid crocodylian from proto-Amazonian mega-wetlands reveals parallel evolutionary trends in skull shape linked to longirostry. *PloS one*, *11*(4), e0152453.

Salas-Gismondi, R., Flynn, J. J., Baby, P., Tejada-Lara, J. V., Wesselingh, F. P., & Antoine, P.-O. (2015). A Miocene hyperdiverse crocodylian community reveals peculiar trophic dynamics in proto-Amazonian mega-wetlands. *Proceedings of the Royal Society B: Biological Sciences*, *282*(1804), 20142490.

Salas-Gismondi, R., Moreno-Bernal, J. W., Scheyer, T. M., Sánchez-Villagra, M. R., & Jaramillo, C. (2019). New Miocene Caribbean gavialoids and patterns of longirostry in crocodylians. *Journal of Systematic Palaeontology*, *17*(12), 1049–1075.

Salisbury, S. W., & Willis, P. M. A. (1996). A new crocodylian from the early Eocene of south-eastern Queensland and a preliminary investigation of the phylogenetic relationships of crocodyloids. *Alcheringa*, *20*(3), 179–226.

Salisbury, S. W., Molnar, R. E., Frey, E., & Willis, P. M. A. (2006). The origin of modern crocodyliforms: new evidence from the Cretaceous of Australia. *Proceedings of the Royal Society B: Biological Sciences*, *273*(1600), 2439–2448.

Scheyer, T. M., & Delfino, M. (2016). The late Miocene caimanine fauna (Crocodylia: Alligatoroidea) of the Urumaco Formation, Venezuela. *Palaeontologia Electronica*, *19*(3), 1–57.

Schumacher, G.-H. (1973). The head muscles and hyolaryngeal skeleton of turtles and crocodilians. In C. Gans & T. S. Parsons (Eds.), *Biology of the reptilia* (pp. 101–199).

Serrano-Martínez, A., Knoll, F., Narváez, I., Lautenschlager, S., & Ortega, F. (2019a). Inner skull cavities of the basal eusuchian *Lohuecosuchus megadontos* (Upper Cretaceous, Spain) and neurosensorial implications. *Cretaceous Research*, *93*, 66–77.

Serrano-Martínez, A., Knoll, F., Narváez, I., & Ortega, F. (2019b). Brain and pneumatic cavities of the braincase of the basal alligatoroid *Diplocynodon tormis* (Eocene, Spain). *Journal of Vertebrate Paleontology*, *39*(1), e1572612.

Shan, H.-Y., Wu, X.-C., Cheng, Y.-N., & Sato, T. (2017). *Maomingosuchus petrolica*, a restudy of '*Tomistoma*' *petrolica* Yeh, 1958. *Palaeoworld*, *26*(4), 672–690.

Shan, H.-y., Wu, X.-c., Cheng, Y.-n., & Sato, T. (2009). A new tomistomine (Crocodylia) from the Miocene of Taiwan. *Canadian Journal of Earth Sciences*, *46*(7), 529–555.

Sill, W. D. (1970). Nota preliminar sobre un nuevo Gavial del Plioceno de Venezuela y una discusion de los gaviales Sudamericanos. *Ameghiniana*, *7*(2), 151–159.

Sookias, R. B. (2020). Exploring the effects of character construction and choice, outgroups and analytical method on phylogenetic inference from discrete characters in extant crocodilians. *Zoological Journal of the Linnean Society*, *189*(2), 670–699.

Souza-Filho, J. P., Souza, R. G., Hsiou, A. S., Riff, D., Guilherme, E., Negri, F. R., & Cidade, G. M. (2019). A new caimanine (Crocodylia, Alligatoroidea) species from the Solimões Formation of Brazil and the phylogeny of Caimaninae. *Journal of Vertebrate Paleontology*, *38*(5), e1528450.

Stein, M., Hand, S. J., & Archer, M. (2016). A new crocodile displaying extreme constriction of the mandible, from the late Oligocene of Riversleigh, Australia. *Journal of Vertebrate Paleontology*, *36*(5), e1179041.

Sternberg, C. M. (1932). A new fossil crocodile from Saskatchewan. *The Canadian Field-Naturalist*, *44*, 128–133.

Storrs, G. W., Lucas, S. G., & Schoch, R. M. (1983). Endocranial cast of an early Paleocene crocodilian from the San Juan basin, New Mexico. *Copeia*, *1983*(3), 842–845.

Taplin, L. E., & Grigg, G. C. (1989). Historical zoogeography of the eusuchian crocodilians: a physiological perspective. *American Zoologist*, *29*(3), 885–901.

Tarsitano, S. F., Frey, E., & Riess, J. (1989). The evolution of the Crocodilia: a conflict between morphological and biochemical data. *American Zoologist*, *29*(3), 843–856.

Tennant, J. P., Mannion, P. D., & Upchurch, P. (2016). Evolutionary relationships and systematics of Atoposauridae (Crocodylomorpha: Neosuchia): implications for the rise of Eusuchia. *Zoological Journal of the Linnean Society*, *177*(4), 854–936.

Turner, A. H. (2015). A review of *Shamosuchus* and *Paralligator* (Crocodyliformes, Neosuchia) from the Cretaceous of Asia. *PLoS One*, *10*(2), e0118116.

Turner, A. H., & Pritchard, A. C. (2015). The monophyly of Susisuchidae (Crocodyliformes) and its phylogenetic placement in Neosuchia. *PeerJ*, *3*, e759.

Vickaryous, M. K., & Hall, B. K. (2008). Development of the dermal skeleton in *Alligator mississippiensis* (Archosauria, Crocodylia) with comments on the homology of osteoderms. *Journal of morphology*, *269*(4), 398–422.

Wang, Y.-y., Sullivan, C., & Liu, J. (2016). Taxonomic revision of *Eoalligator* (Crocodylia, Brevirostres) and the paleogeographic origins of the Chinese alligatoroids. *PeerJ*, *4*, e2356.

Wermuth, H. (1953). Systematik der rezenten Krokodile. *Mitteilungen der Zoologisches Museum Berlin*, *29*, 375–511.

Willis, P. M. A. (1997). New crocodilians from the Late Oligocene White Hunter Site, Riversleigh, northwestern Queensland. *Memoirs of the Queensland Museum*, *41*, 423–438.

Willis, P. M. A., Murray, P. F., & Megirian, D. (1990). *Baru darrowi* gen. et sp. nov., a large broad-snouted crocodyline (Eusuchia: Crocodylidae) from mid-Tertiary freshwater limestones in northern Australia. *Memoirs of the Queensland Museum*, *29*(2), 521–540.

Willis, P. M. A., Molnar, R. E., & Scanlon, J. D. (1993). An early Eocene crocodilian from Murgon, southeastern Queensland. *Kaupia*, *3*, 27–33.

Willis, P. M. (1993). *Trilophosuchus rackhami* gen. et sp. nov., a new crocodilian from the early Miocene limestones of Riversleigh, northwestern Queensland. *Journal of Vertebrate Paleontology*, *13*(1), 90–98.

Willis, P. (2001). New crocodilian material from the Miocene of Riversleigh (northwestern Queensland, Australia). *Crocodilian biology and evolution*, 64–74.

Witmer, L. M. (1997). The evolution of the antorbital cavity of archosaurs: a study in soft-tissue reconstruction in the fossil record with an analysis of the function of pneumaticity. *Journal of Vertebrate Paleontology*, *17*(S1), 1–76.

Wu, X.-C., & Brinkman, D. B. (1993). A new crocodylomorph of ”mesosuchian” grade from the Upper Cretaceous Upper Milk River Formation, southern Alberta. *Journal of Vertebrate Paleontology*, *13*(2), 153–160.

Wu, X.-C., Brinkman, D. B., & Russell, A. P. (1996). A new alligator from the Upper Cretaceaous of Canada and the relationship of early eusuchians. *Palaeontology*, *39*, 351–376.

Wu, X.-C., Russell, A. P., & Brinkman, D. B. (2001a). A review of *Leidyosuchus canadensis* Lambe, 1907 (Archosauria: Crocodylia) and an assessment of cranial variation based upon new material. *Canadian Journal of Earth Sciences*, *38*(12), 1665–1687.

Wu, X.-C., Russell, A. P., & Cumbaa, S. L. (2001b). *Terminonaris* (Archosauria: Crocodyliformes): New material from Saskatchewan, Canada, and comments on its phylogenetic relationships. *Journal of Vertebrate Paleontology*, *21*(3), 492–514.

Wu, X.-C., & Sues, H.-D. (1996). Anatomy and phylogenetic relationships of *Chimaerasuchus paradoxus*, an unusual crocodyliform reptile from the Lower Cretaceous of Hubei, China. *Journal of Vertebrate Paleontology*, *16*(4), 688–702.

Wu, X.-C., Sues, H.-D., & Dong, Z.-M. (1997). *Sichuanosuchus shuhanensis*, a new? Early Cretaceous protosuchian (Archosauria: Crocodyliformes) from Sichuan (China), and the monophyly of Protosuchia. *Journal of Vertebrate Paleontology*, *17*(1), 89–103.

Yates, A. M. (2017). The biochronology and palaeobiogeography of *Baru* (Crocodylia: Mekosuchinae) based on new specimens from the Northern Territory and Queensland, Australia. *PeerJ*, *5*, e3458.

Zangerl, R. (1944). *Brachyuranochampsa eversolei*, gen. et sp. nov., a new crocodilian from the Washakie Eocene of Wyoming. *Annals of the Carnegie Museum*, *30*, 77–84.
