# Supplementary figures and images for: Phylogenetic analysis of a new morphological dataset elucidates the evolutionary history of Crocodylia and resolves the long-standing gharial problem
Source: PeerJ. 2021 Sep 6;9:e12094. doi: 10.7717/peerj.12094 (PMC8428266; doi:10.7717/peerj.12094)

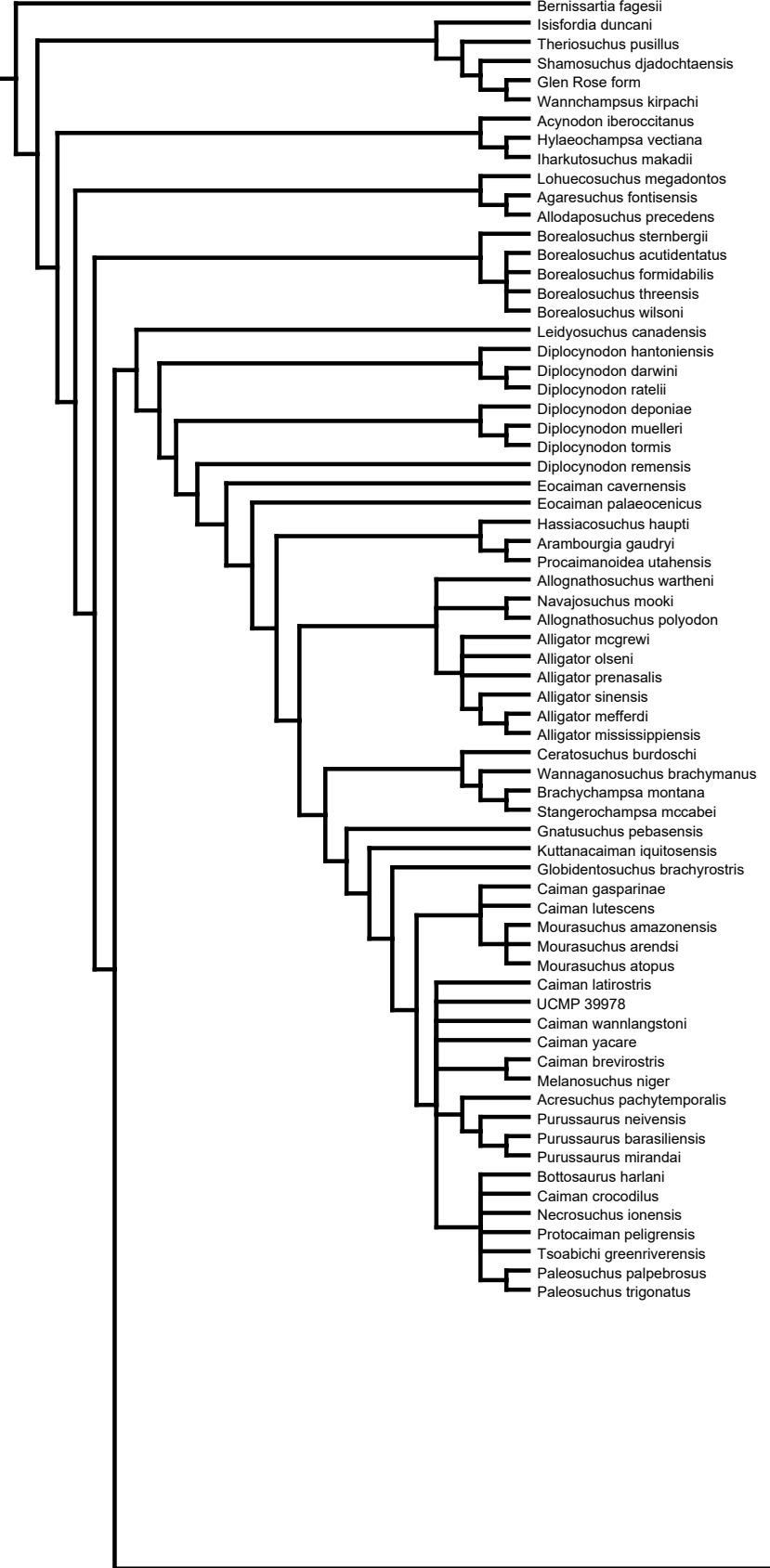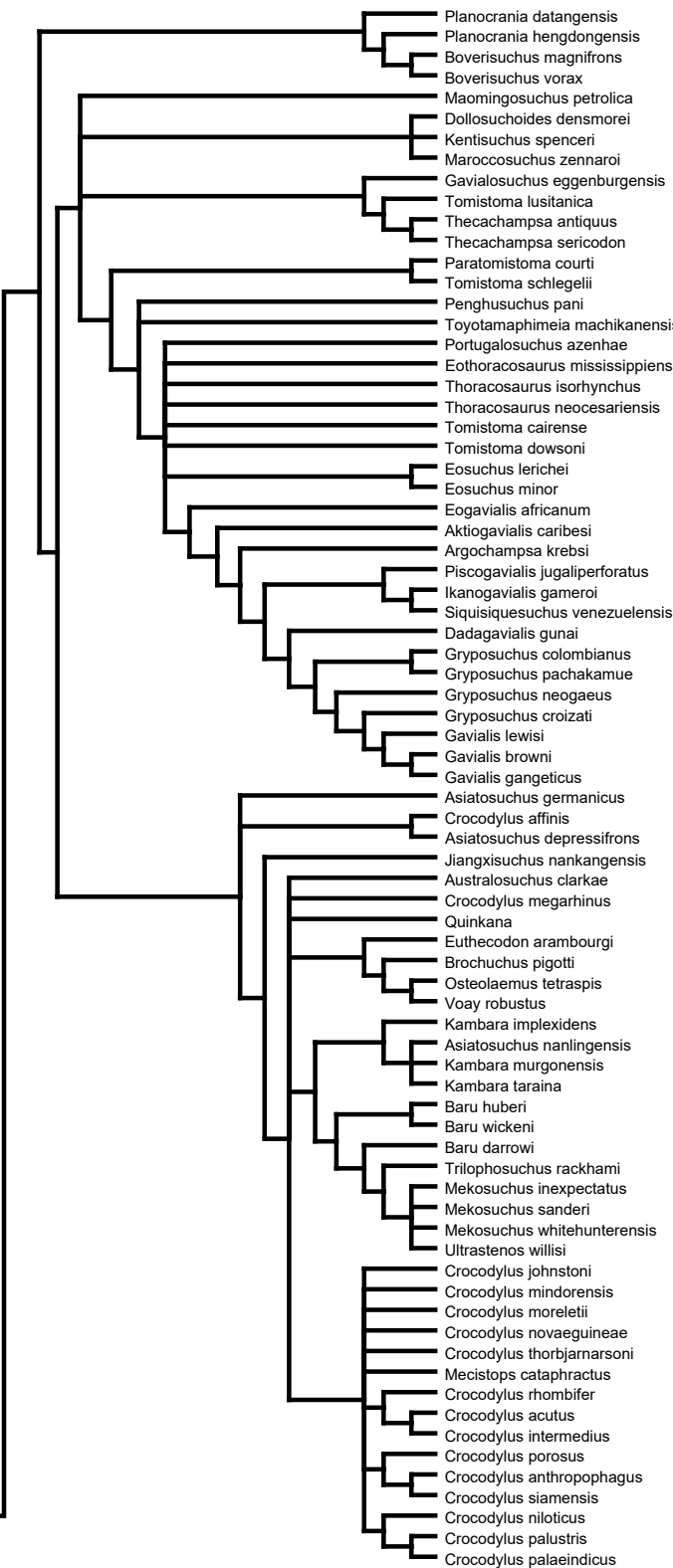

Supplement: Supplemental Information 3 [file peerj-09-12094-s003.pdf]

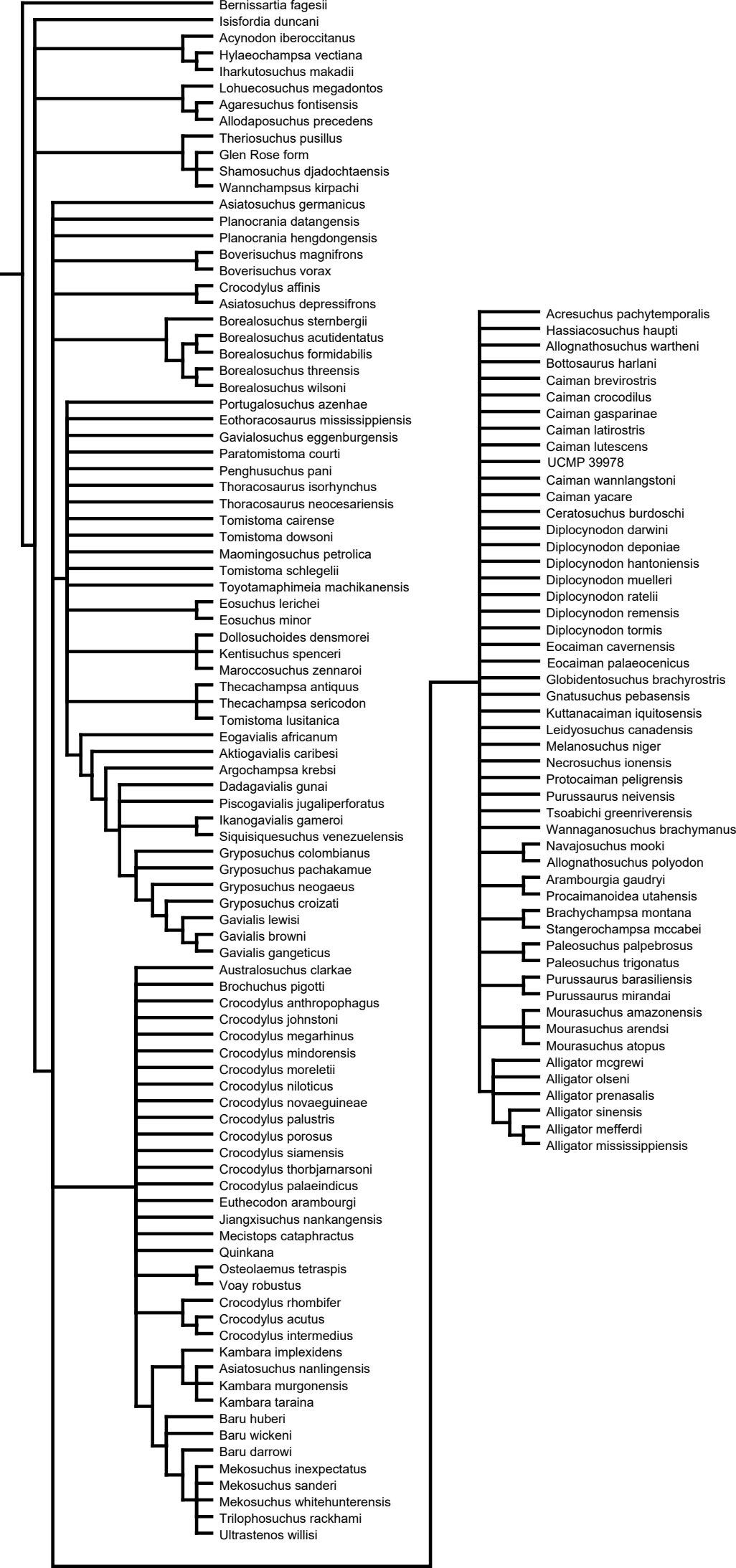

Supplement: Supplemental Information 4 [file peerj-09-12094-s004.pdf]

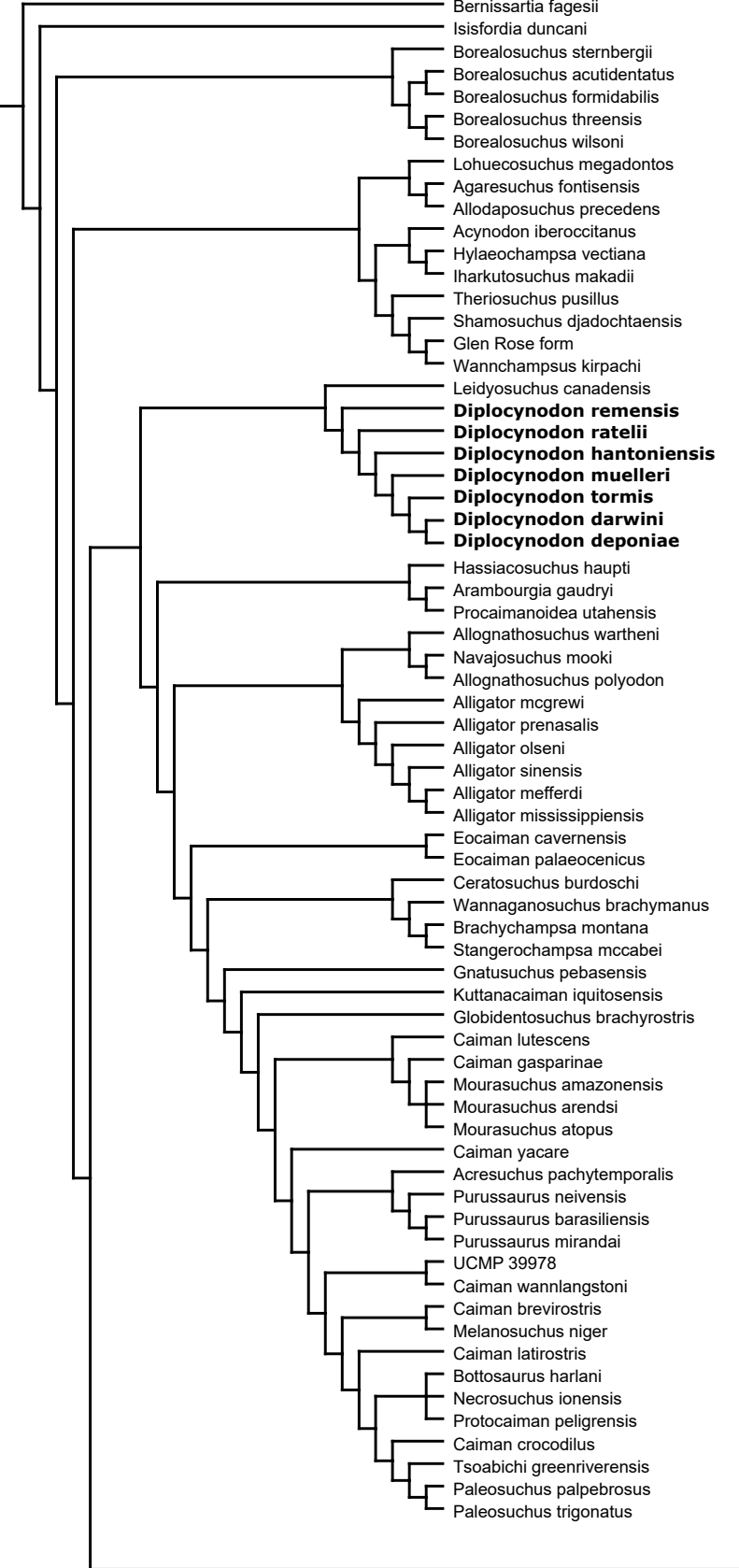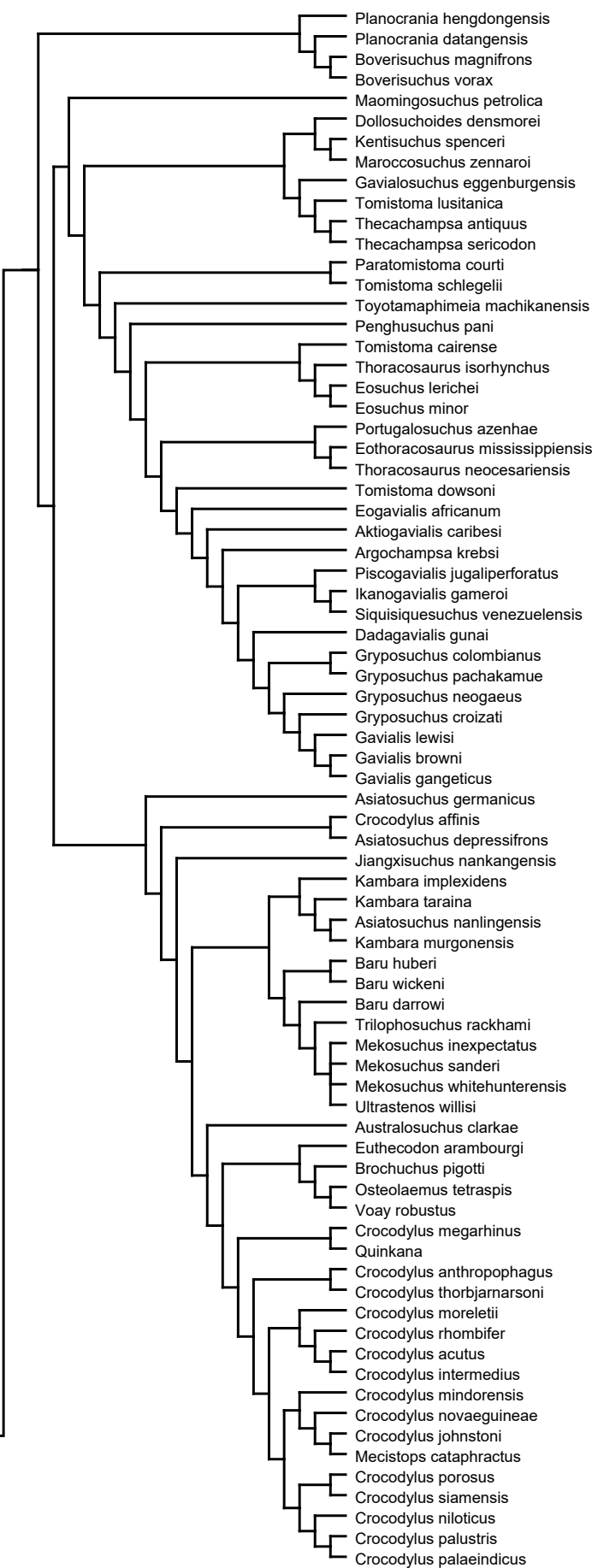

Supplement: Supplemental Information 5 [file peerj-09-12094-s005.pdf]

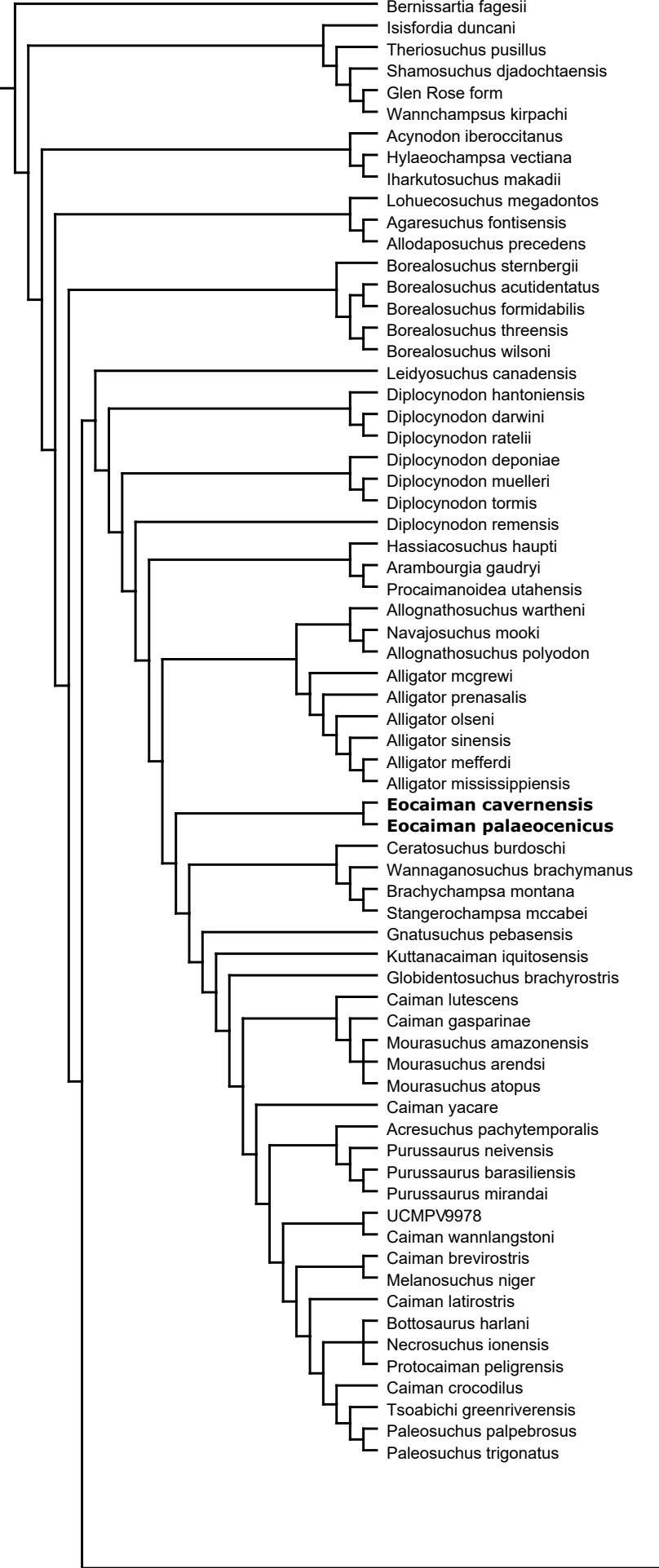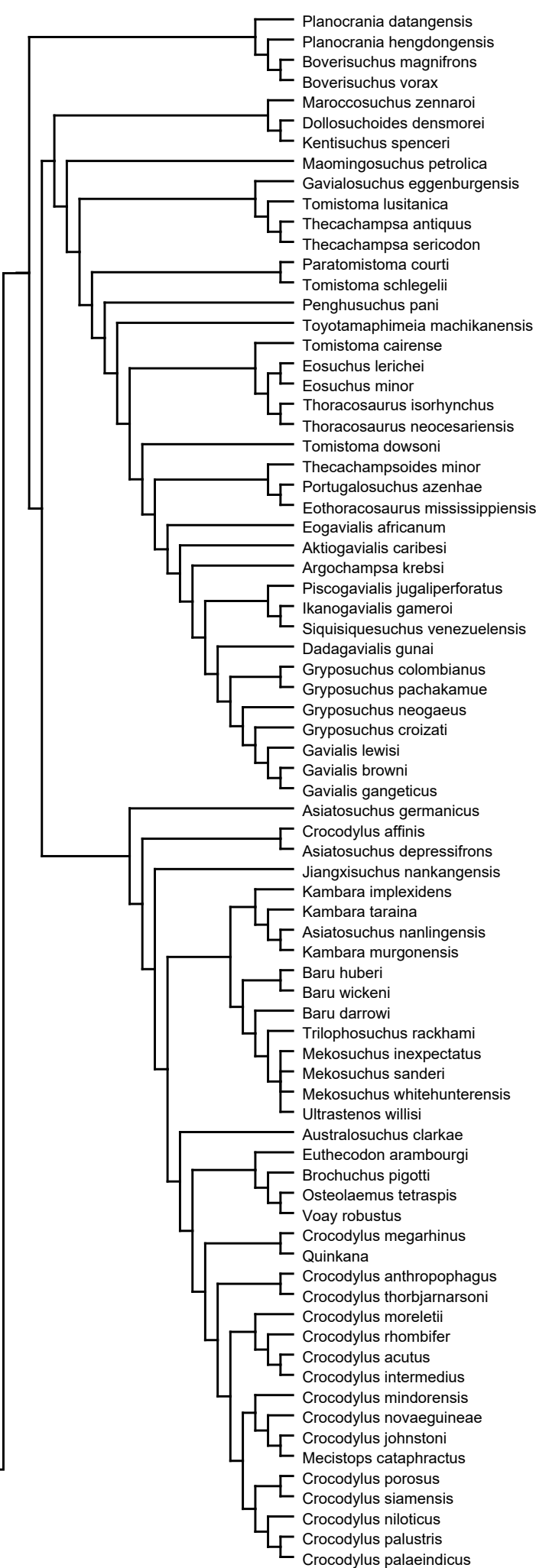

Supplement: Supplemental Information 6 [file peerj-09-12094-s006.pdf]

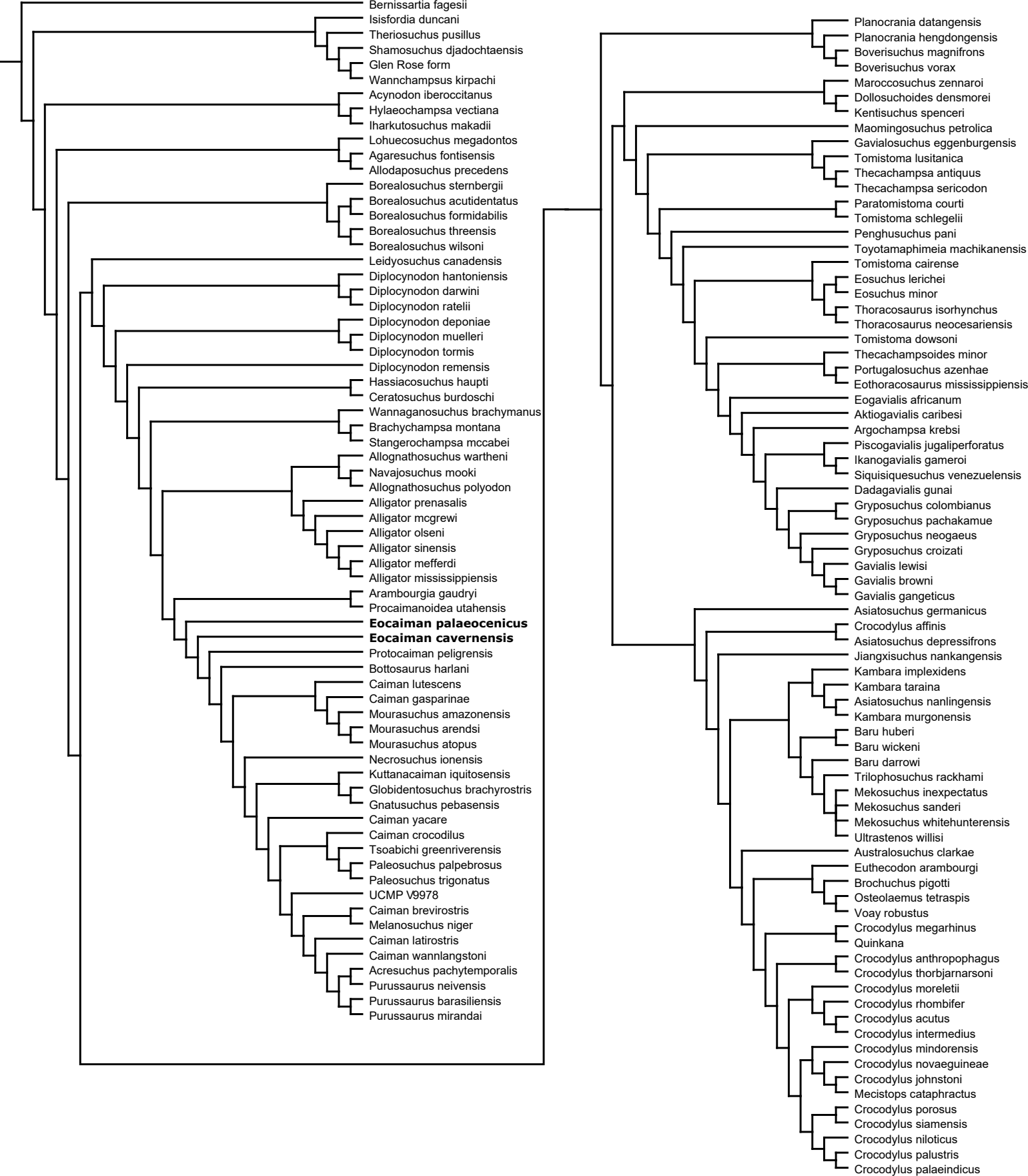

Supplement: Supplemental Information 7 [file peerj-09-12094-s007.pdf]

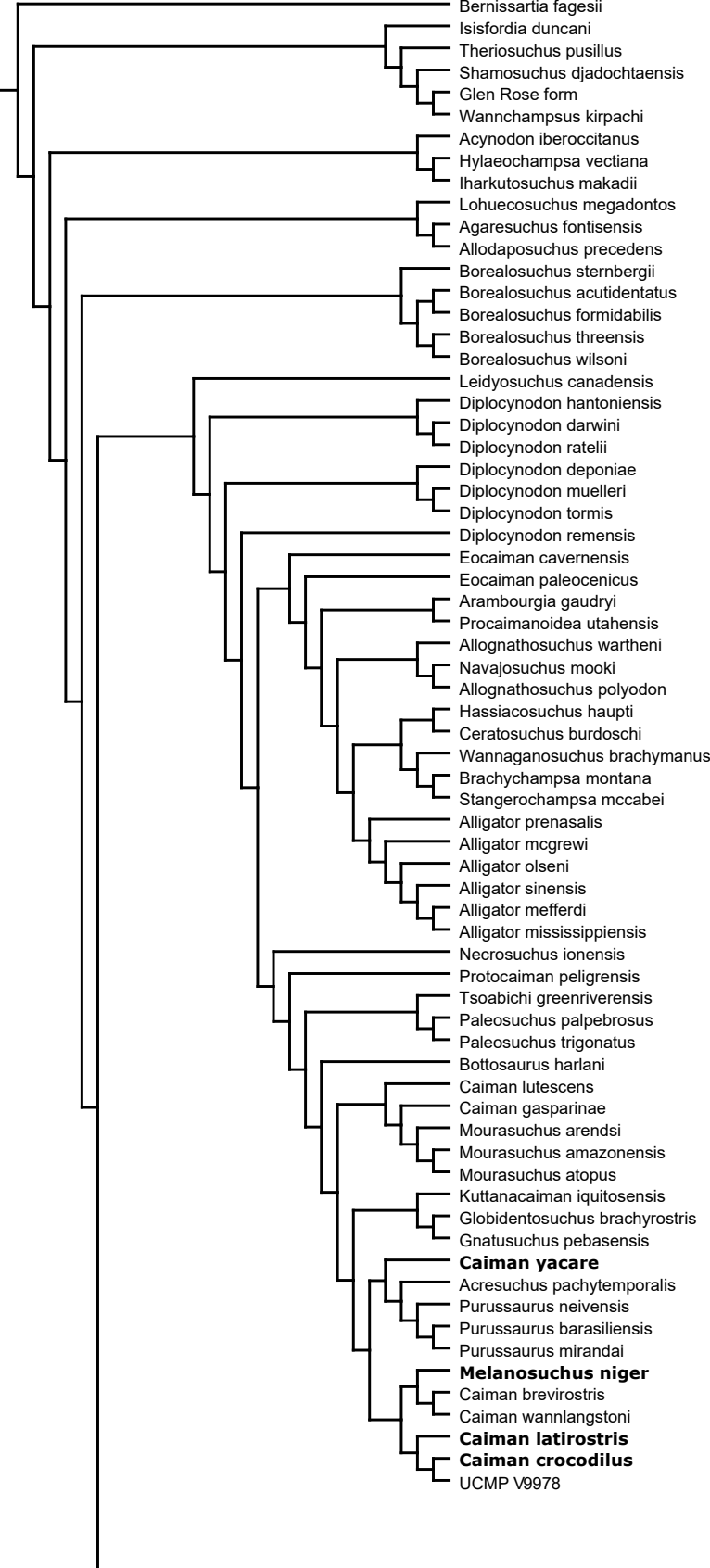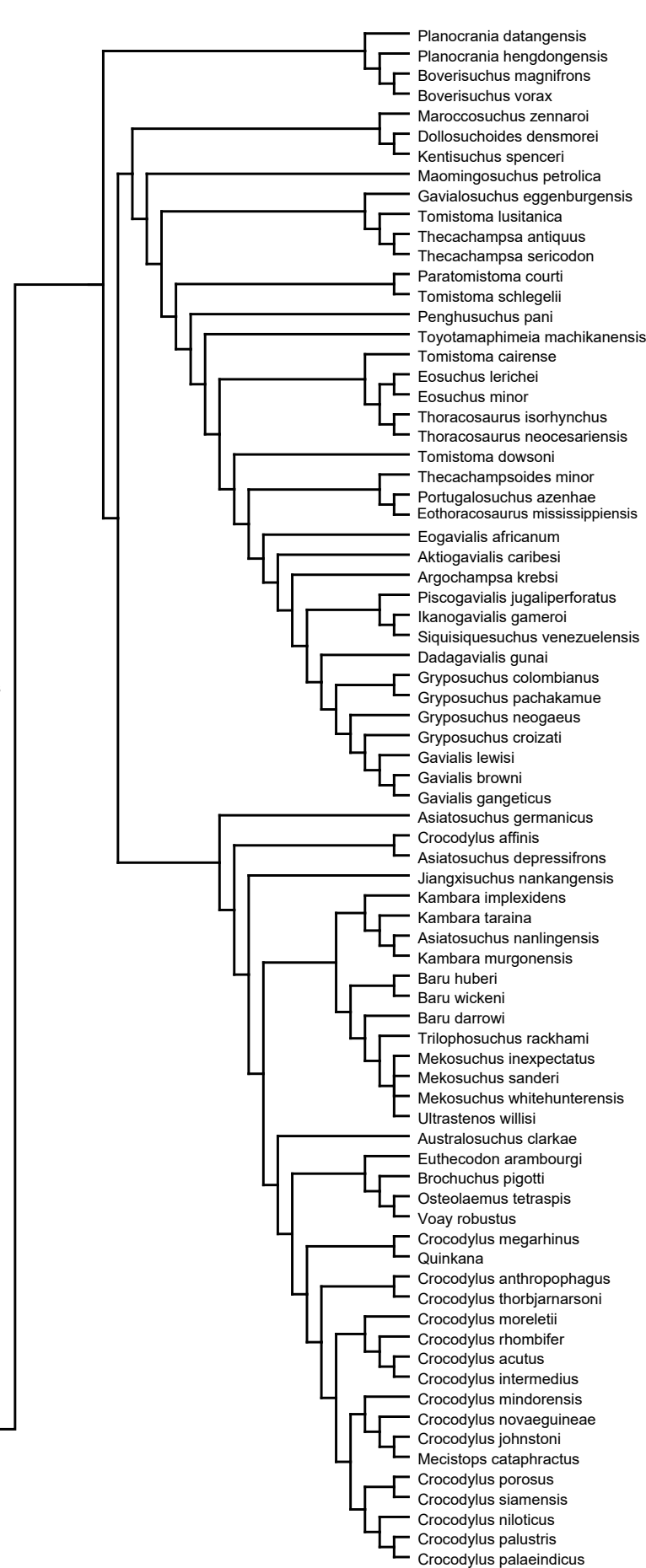

Supplement: Supplemental Information 8 [file peerj-09-12094-s008.pdf]

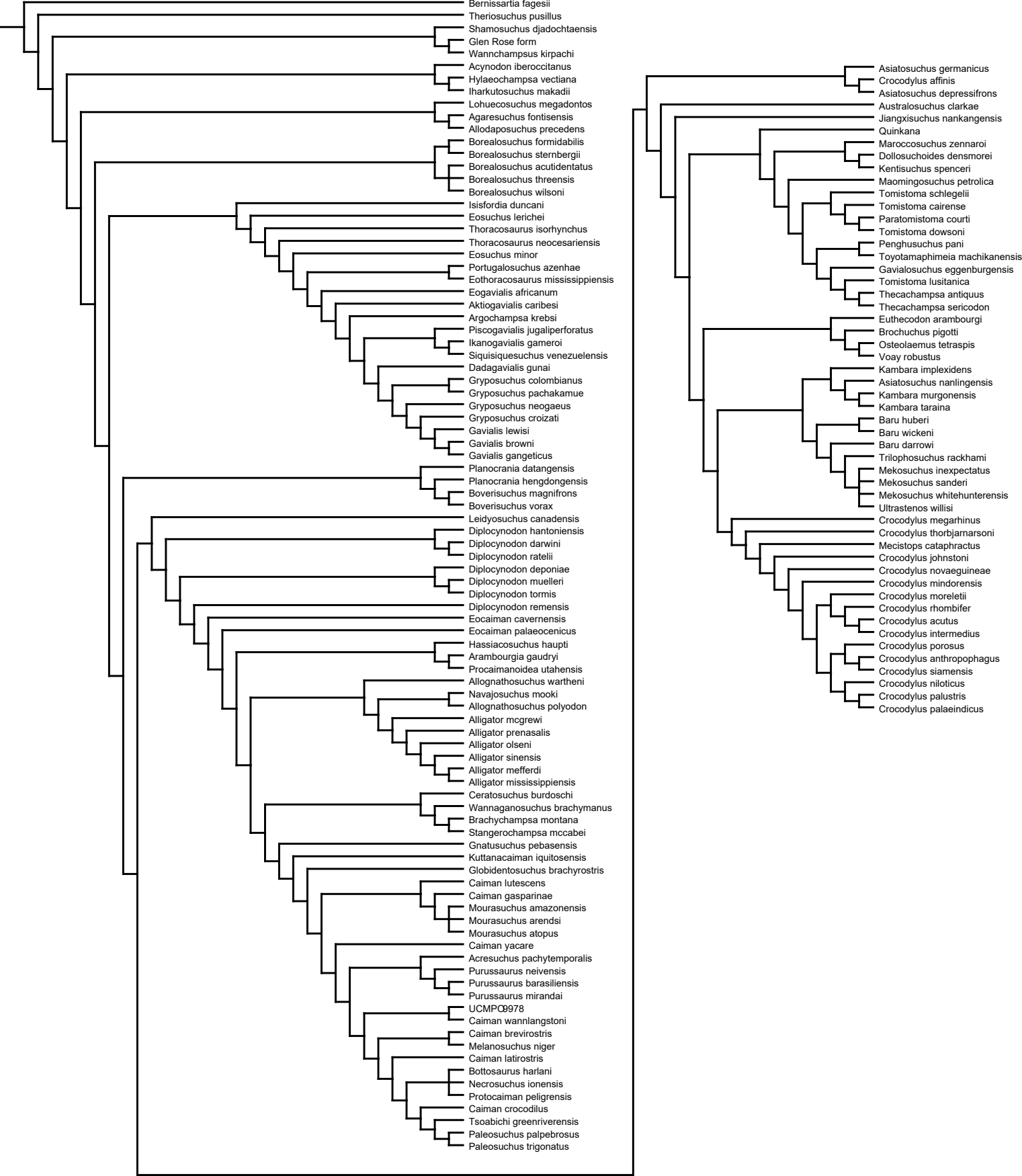

Supplement: Supplemental Information 9 [file peerj-09-12094-s009.pdf]

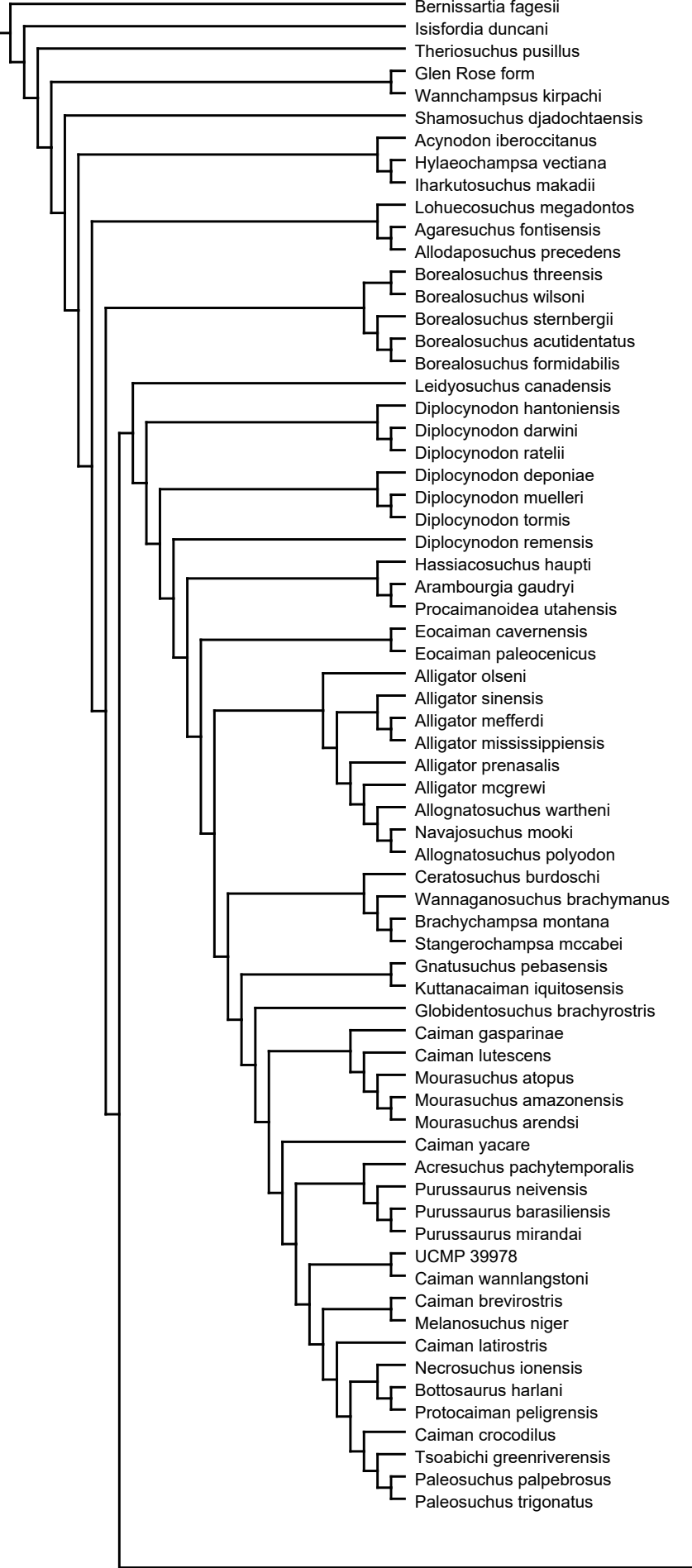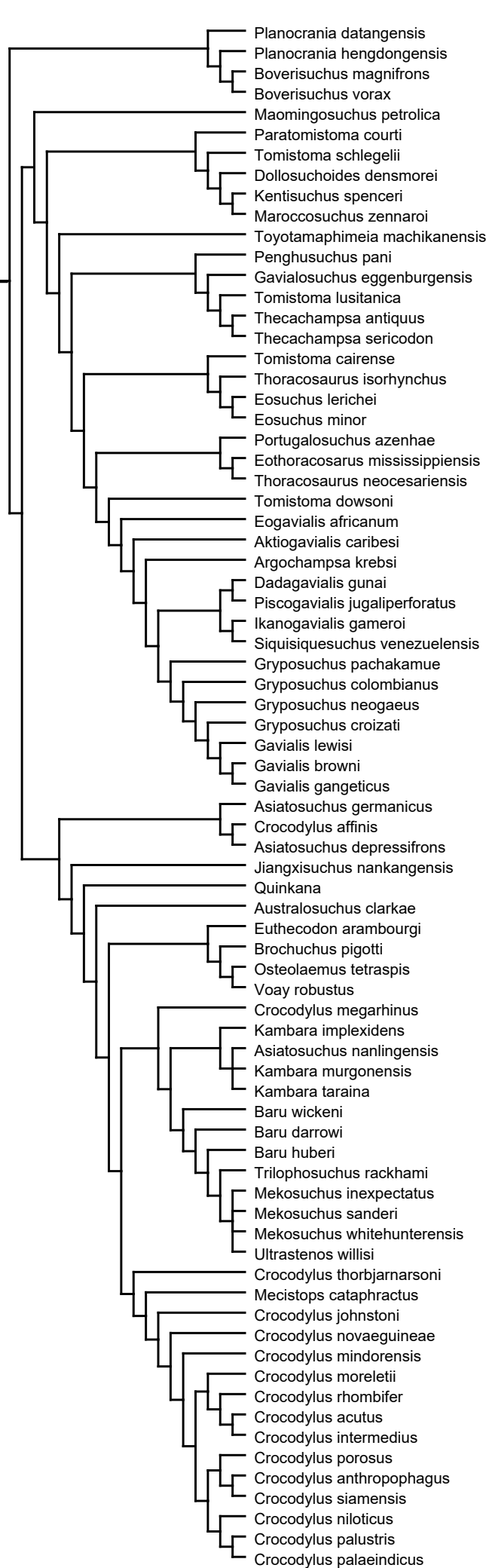

Supplement: Supplemental Information 10 [file peerj-09-12094-s010.pdf]

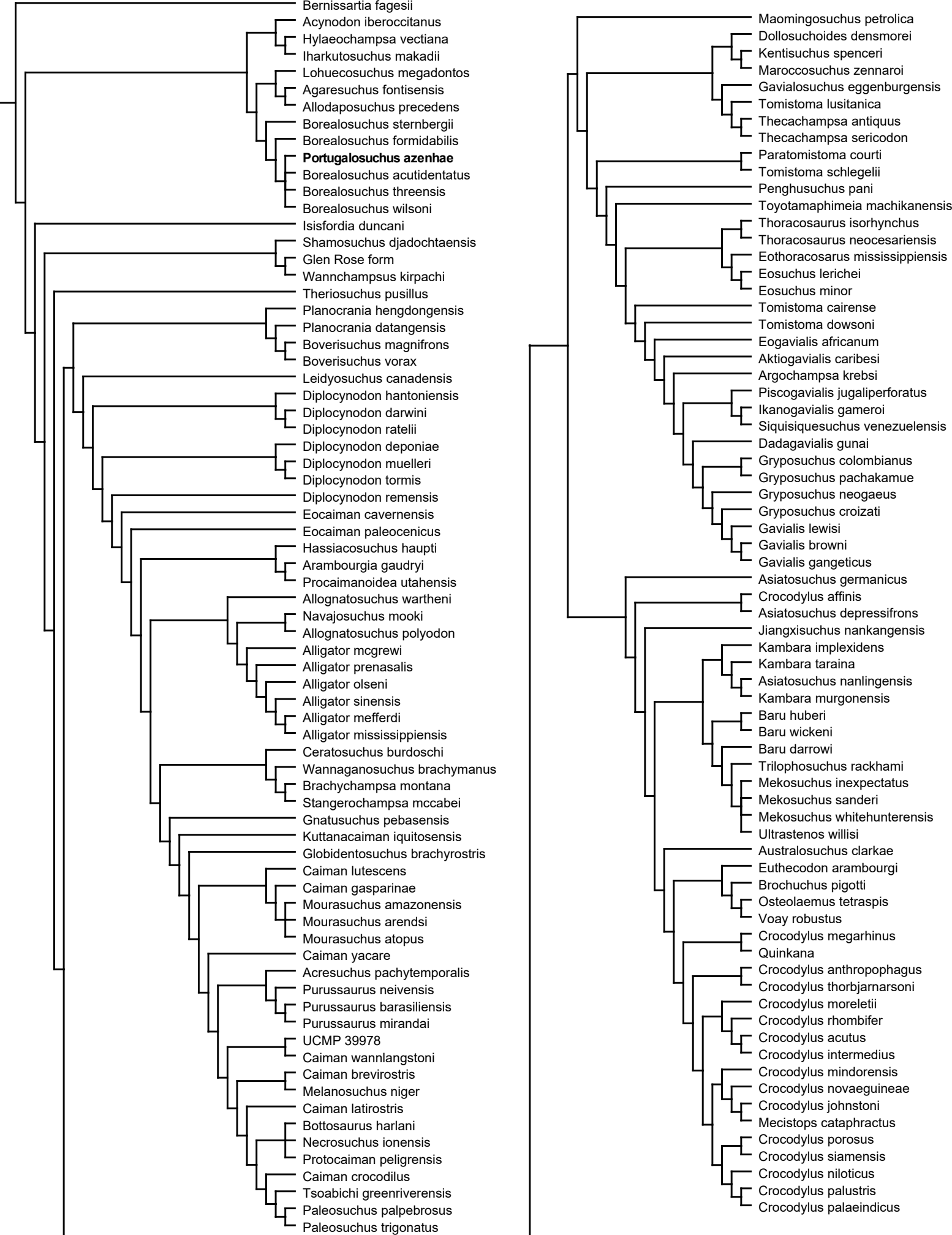

Supplement: Supplemental Information 11 [file peerj-09-12094-s011.pdf]

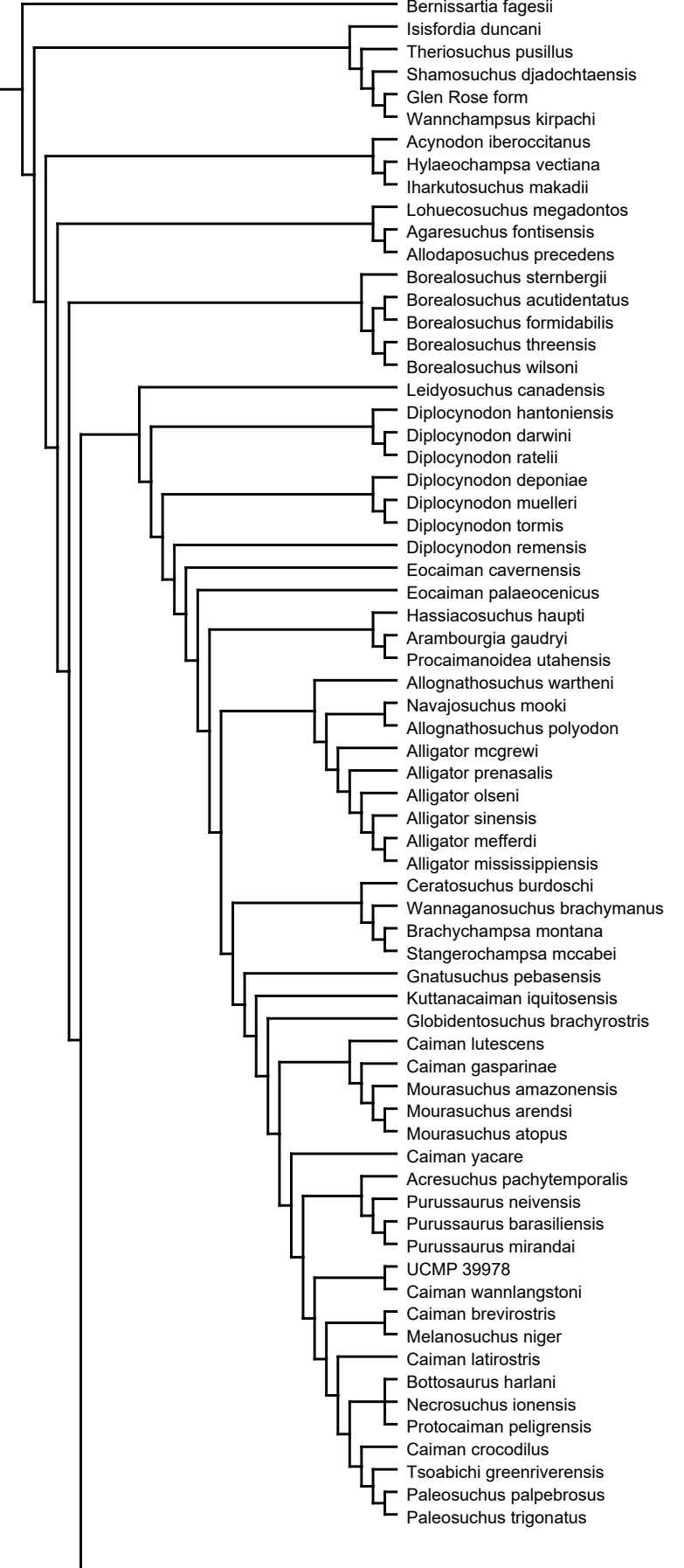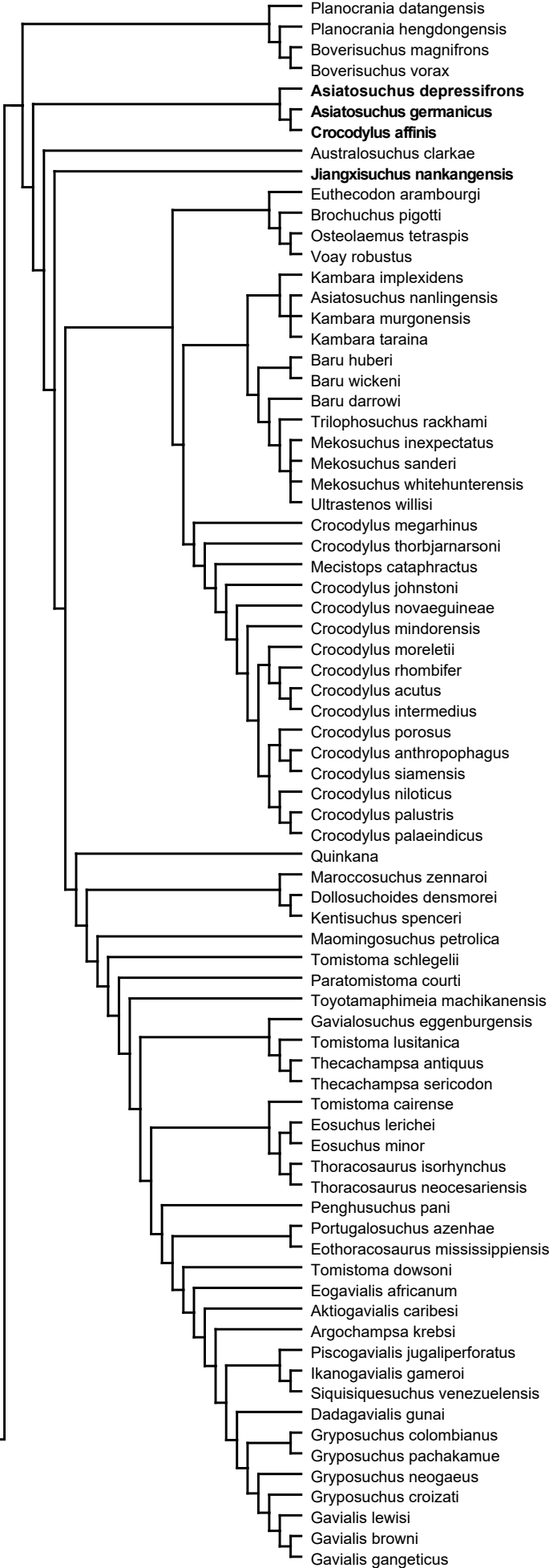

Supplement: Supplemental Information 12 [file peerj-09-12094-s012.pdf]

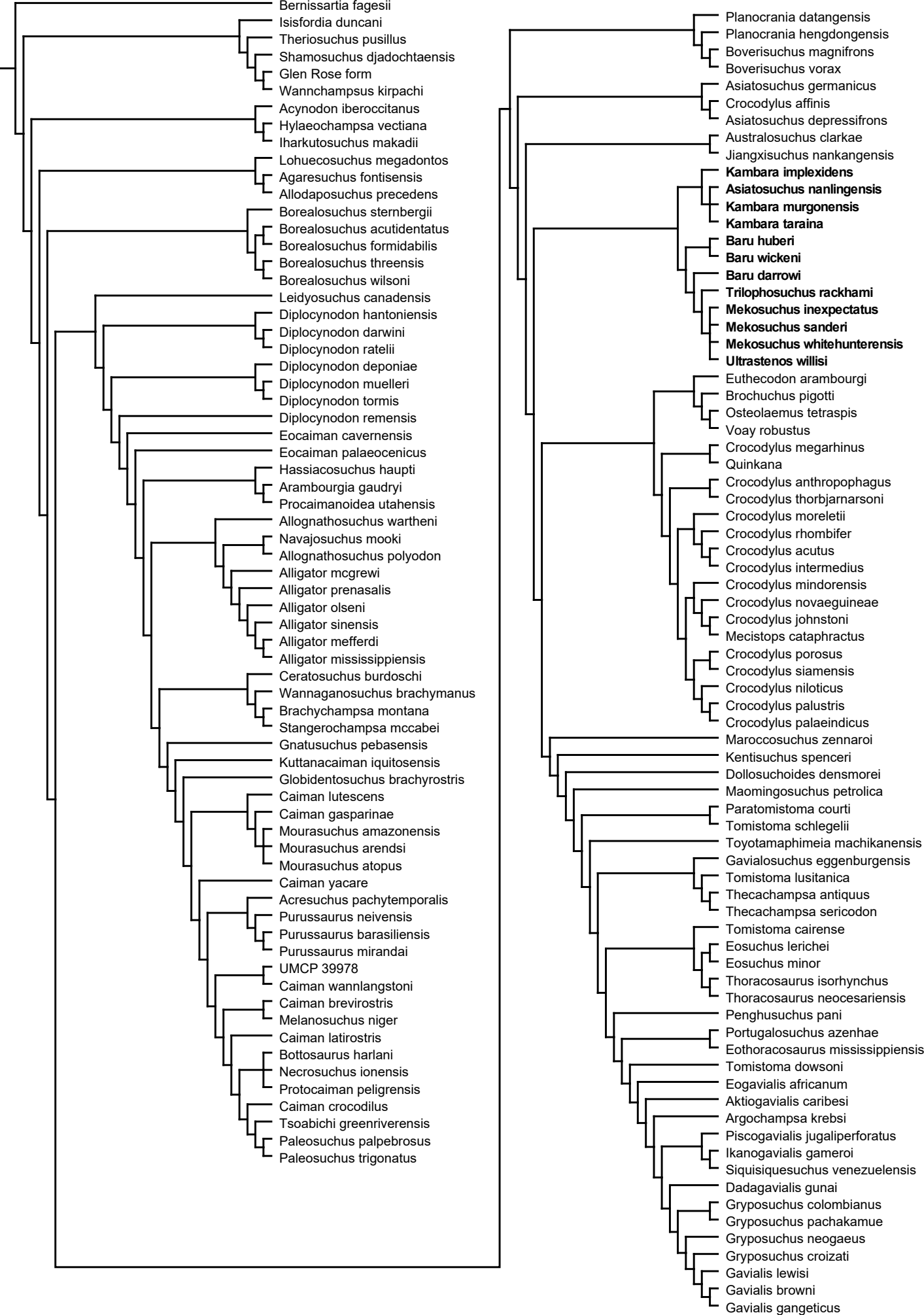

Supplement: Supplemental Information 13 [file peerj-09-12094-s013.pdf]

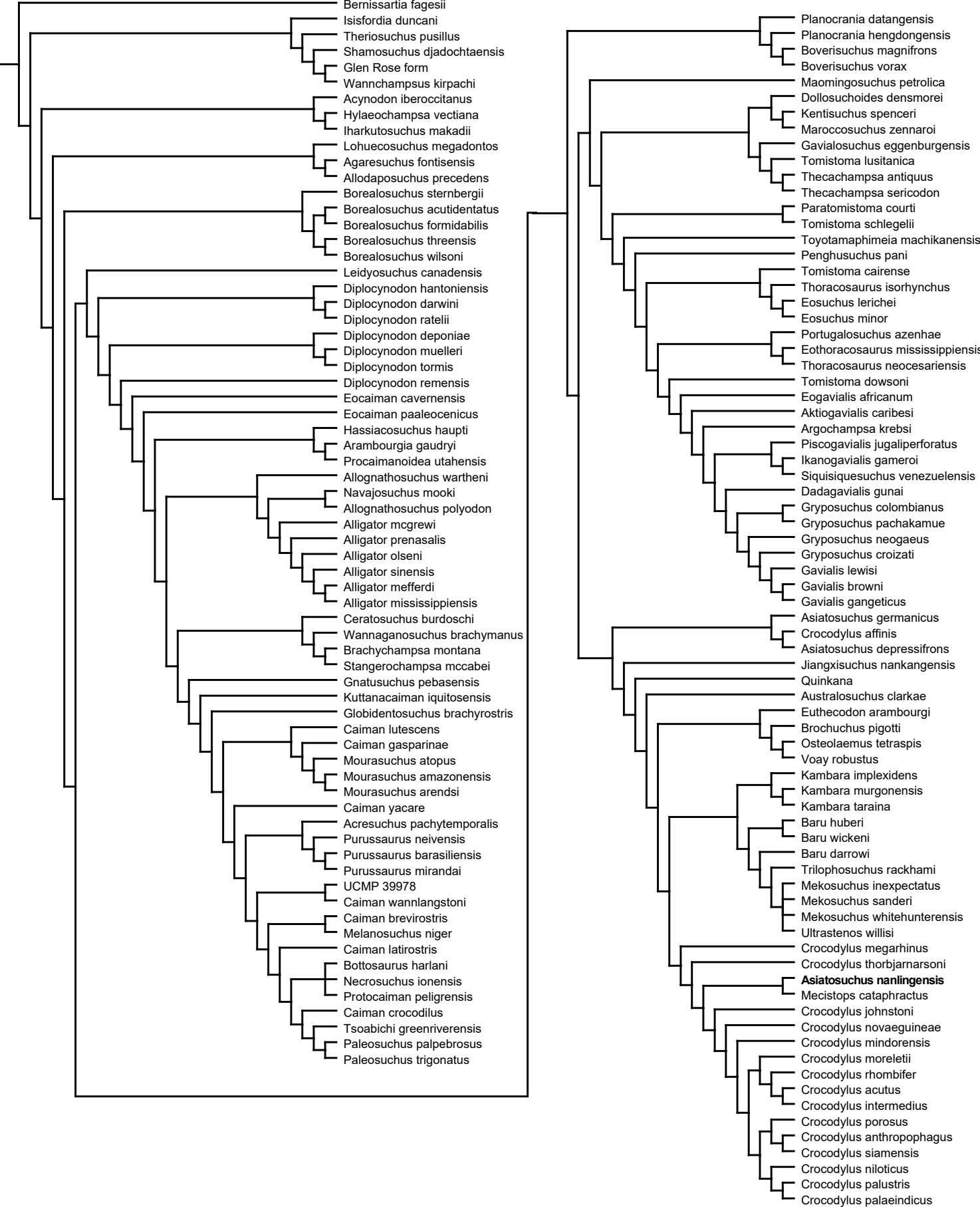

Supplement: Supplemental Information 14 [file peerj-09-12094-s014.pdf]

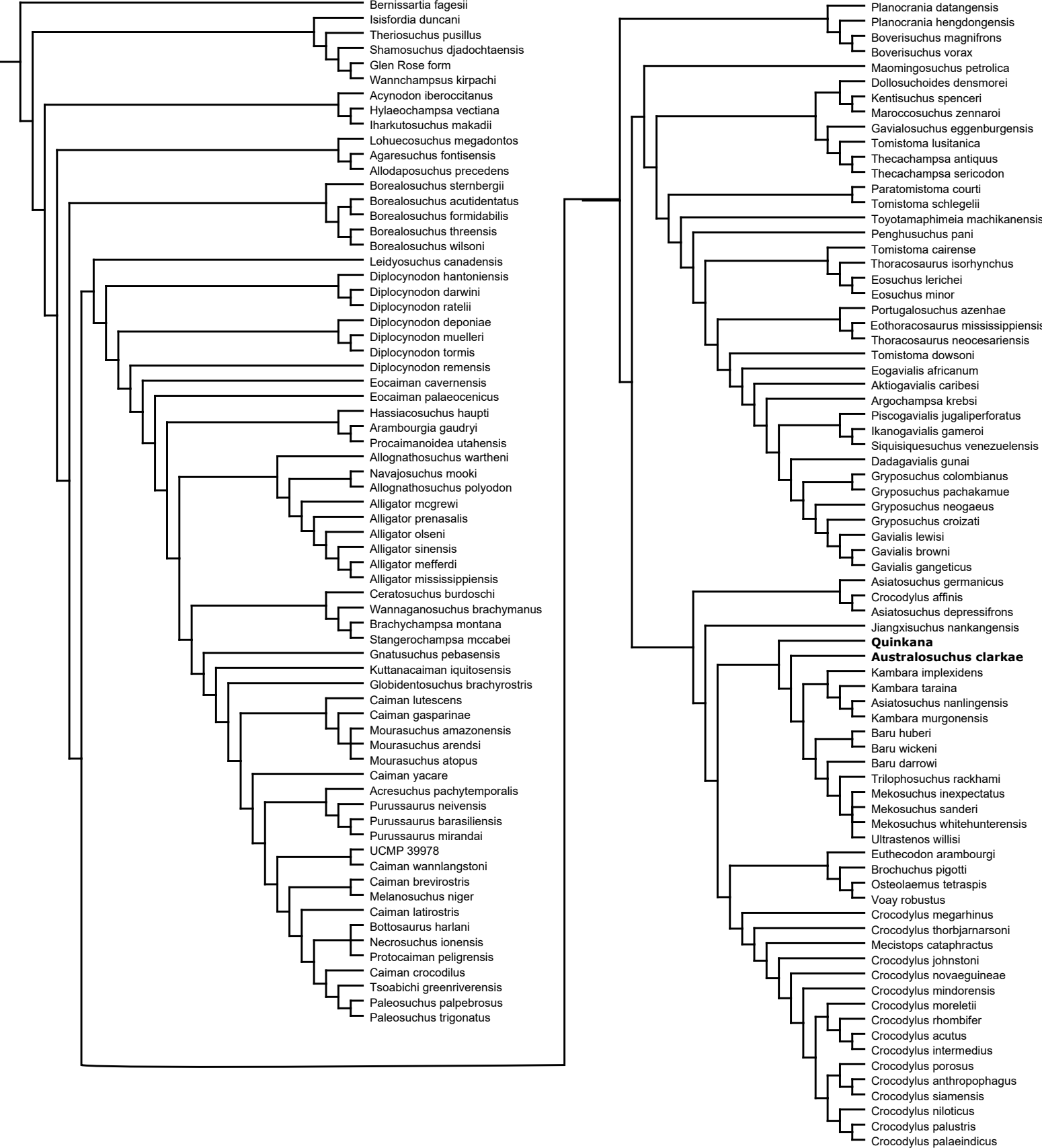

Supplement: Supplemental Information 15 [file peerj-09-12094-s015.pdf]

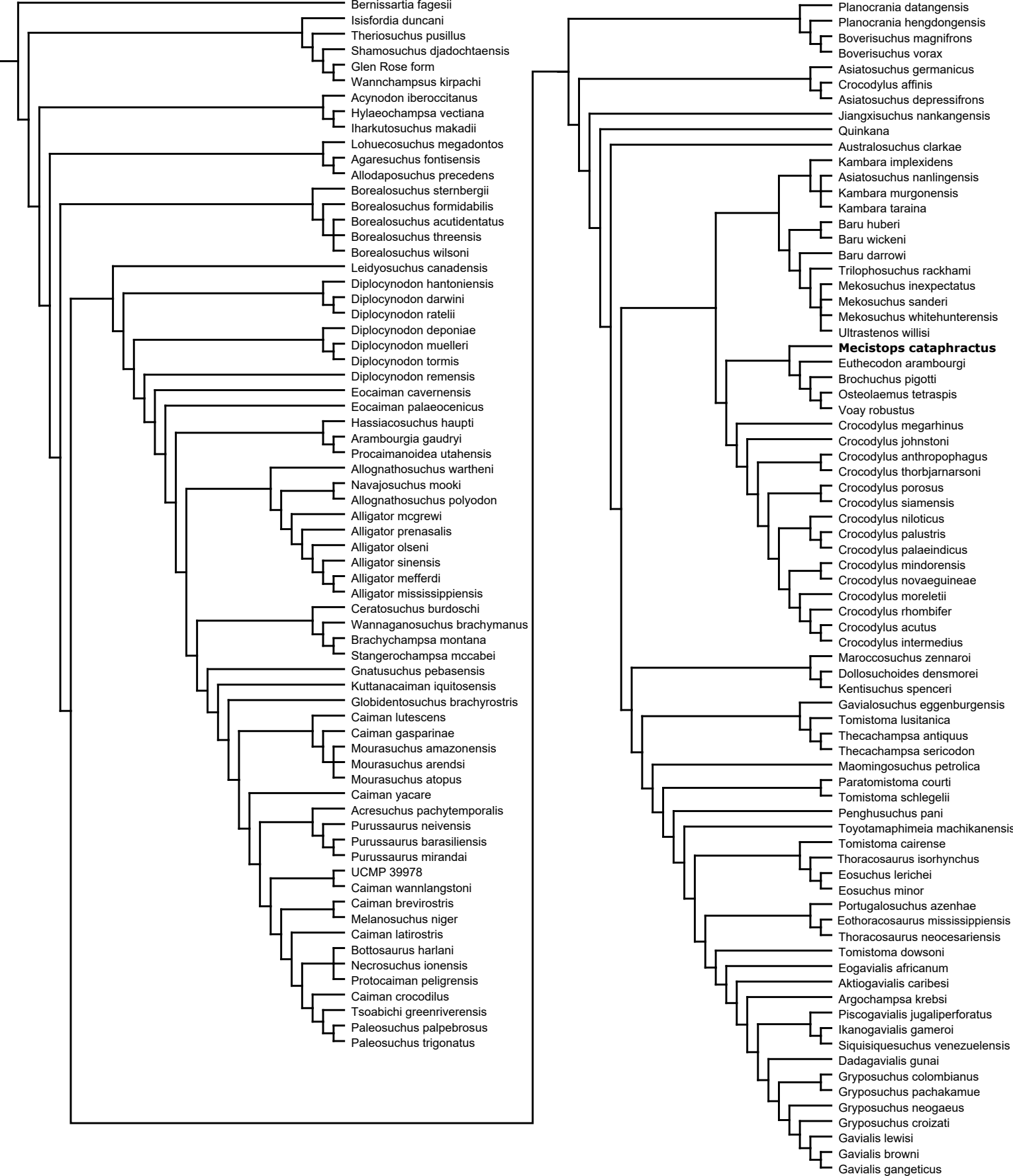

Supplement: Supplemental Information 16 [file peerj-09-12094-s016.pdf]
