# Supplementary material for: Phylogenetic analysis of a new morphological dataset elucidates the evolutionary history of Crocodylia and resolves the long-standing gharial problem
Source: PeerJ. 2021 Sep 6;9:e12094. doi: 10.7717/peerj.12094 (PMC8428266; doi:10.7717/peerj.12094)
Supplement: Supplemental Information 17 [file peerj-09-12094-s017.pdf]

Table 1: Rejected characters

Study	Character	Reason for rejection
Groh et al. (2020)	2	not applicable to this dataset
Groh et al. (2020)	6	not applicable to this dataset
Groh et al. (2020)	7	not applicable to this dataset
Groh et al. (2020)	8	similar to character 5 in this dataset
Groh et al. (2020)	9	not applicable to this dataset
Groh et al. (2020)	10	similar to character 141 in this dataset
Groh et al. (2020)	11	not applicable to this dataset
Groh et al. (2020)	14	continuous character not measured
Groh et al. (2020)	15	not applicable to this dataset
Groh et al. (2020)	17	similar to character 53 in this dataset
Groh et al. (2020)	18	similar to character 58 in this dataset
Groh et al. (2020)	19	not applicable to this dataset
Groh et al. (2020)	20	not applicable to this dataset
Groh et al. (2020)	21	similar to character 6 in this dataset
Groh et al. (2020)	22	similar to character 61 in this dataset
Groh et al. (2020)	23	similar to character 62 in this dataset
Groh et al. (2020)	24	similar to character 6 in this dataset
Groh et al. (2020)	25	similar to character 6 in this dataset
Groh et al. (2020)	29	not applicable to this dataset
Groh et al. (2020)	32	similar to character 6 in this dataset
Groh et al. (2020)	36	similar to character 183 in this dataset
Groh et al. (2020)	37	similar to character 1 in this dataset
Groh et al. (2020)	40	similar to character 10 in this dataset
Groh et al. (2020)	43	similar to character 2 in this dataset
Groh et al. (2020)	44	similar to character 8 in this dataset
Groh et al. (2020)	45	similar to character 8 in this dataset
Groh et al. (2020)	48	similar to character 14 in this dataset
Groh et al. (2020)	49	not applicable to this dataset
Groh et al. (2020)	50	not applicable to this dataset
Groh et al. (2020)	51	not applicable to this dataset
Groh et al. (2020)	53	similar to character 218 in this dataset
Groh et al. (2020)	54	similar to character 220 in this dataset
Groh et al. (2020)	57	similar to character 262 in this dataset
Groh et al. (2020)	58	not applicable to this dataset
Groh et al. (2020)	59	similar to character 266 in this dataset
Groh et al. (2020)	60	similar to character 277 in this dataset
Groh et al. (2020)	62	similar to character 275 in this dataset
Groh et al. (2020)	63	not applicable to this dataset
Groh et al. (2020)	64	similar to character 280 in this dataset
Groh et al. (2020)	65	not applicable to this dataset
Groh et al. (2020)	66	not applicable to this dataset
Groh et al. (2020)	67	similar to character 301 in this dataset

Table 1: Rejected characters

Study	Character	Reason for rejection
Groh et al. (2020)	70	not applicable to this dataset
Groh et al. (2020)	71	continuous character not measured
Groh et al. (2020)	72	continuous character not measured
Groh et al. (2020)	73	continuous character not measured
Groh et al. (2020)	74	continuous character not measured
Groh et al. (2020)	75	continuous character not measured
Groh et al. (2020)	76	considered impractical to measure
Groh et al. (2020)	77	not applicable to this dataset
Groh et al. (2020)	78	considered impractical to measure
Groh et al. (2020)	79	continuous character not measured
Groh et al. (2020)	81	continuous character not measured
Groh et al. (2020)	107	invalid character considered artefact of preservation
Groh et al. (2020)	116	not applicable to this dataset
Groh et al. (2020)	123	considerable ontogenetic and intraspecific variation in character
Groh et al. (2020)	130	not applicable to this dataset
Groh et al. (2020)	138	similar to character 159 in this dataset
Groh et al. (2020)	143	similar to character 179 in this dataset
Groh et al. (2020)	158	similar to character 60 in this dataset
Groh et al. (2020)	163	anatomically invalid character
Groh et al. (2020)	261	anatomically invalid character
Groh et al. (2020)	291	not applicable to this dataset
Groh et al. (2020)	295	not applicable to this dataset
Groh et al. (2020)	362	not applicable to this dataset
Groh et al. (2020)	381	ontogenetically variable
Groh et al. (2020)	384	all taxa scored the same in this dataset
Groh et al. (2020)	414	character 155 in this dataset
Groh et al. (2020)	423	not applicable to this dataset
Iijima and Kobayashi (2019)	245	no variation in taxa
Salas-Gismondi et al. (2019)	207	similar to character 14 in this dataset
Salas-Gismondi et al. (2019)	208	similar to character 11 in this dataset
Salas-Gismondi et al. (2019)	209	similar to character 9 in this dataset
Lee and Yates (2018)	1	anatomically invalid character
Lee and Yates (2018)	6	no variation in taxa
Lee and Yates (2018)	13	redundant with character 143 in this dataset
Lee and Yates (2018)	15	scores do not match observations
Lee and Yates (2018)	17	scores do not match observations
Lee and Yates (2018)	32	no variation in taxa
Lee and Yates (2018)	35	anatomically invalid character
Lee and Yates (2018)	47	anatomically invalid character

Table 1: Rejected characters

Study	Character	Reason for rejection
Lee and Yates (2018)	54	no variation in taxa
Lee and Yates (2018)	62	no variation in taxa
Lee and Yates (2018)	73	redundant with character 84 in this dataset
Lee and Yates (2018)	82	varies intraspecifically
Lee and Yates (2018)	83	similar to character 84 in this dataset
Lee and Yates (2018)	84	no variation in taxa
Lee and Yates (2018)	85	no variation in taxa
Lee and Yates (2018)	86	no variation in taxa
Lee and Yates (2018)	89	no variation in taxa
Lee and Yates (2018)	93	no variation in taxa
Lee and Yates (2018)	94	redundant with character 8 in this dataset
Lee and Yates (2018)	102	no variation in taxa
Lee and Yates (2018)	113	variable within species ontogenetically
Lee and Yates (2018)	114	variable within species ontogenetically
Lee and Yates (2018)	116	no variation in taxa
Lee and Yates (2018)	141	varies intraspecifically
Lee and Yates (2018)	148	similar to character 171 in this dataset
Lee and Yates (2018)	149	similar to character 172 in this dataset
Lee and Yates (2018)	153	no variation in taxa
Lee and Yates (2018)	170	autapomorphy of *Crocodylus johnstoni* in this dataset
Lee and Yates (2018)	172	no variation in taxa
Lee and Yates (2018)	177	no variation in taxa
Lee and Yates (2018)	178	similar to character 151 in this dataset
Lee and Yates (2018)	179	no variation in taxa
Lee and Yates (2018)	181	no variation in taxa
Lee and Yates (2018)	183	varies intraspecifically
Lee and Yates (2018)	189	no variation in taxa
Lee and Yates (2018)	193	no variation in taxa
Lee and Yates (2018)	200	no variation in taxa
Lee and Yates (2018)	218	no variation in taxa
Lee and Yates (2018)	219	not practical to score
Lee and Yates (2018)	237	no variation in taxa
Lee and Yates (2018)	262	character 321 in this dataset
Lee and Yates (2018)	277	character 317 in this dataset
Cidade et al. (2017)	187	no variation in taxa
Jouve (2016)	140	similar to character 9 in this dataset
Jouve (2016)	211	no variation in taxa
Jouve (2016)	241	similar to character 126 in this dataset
Salas-Gismondi et al. (2016)	202	no variation in taxa
Salas-Gismondi et al. (2016)	206	similar to character 72 in this dataset

Table 1: Rejected characters

Study	Character	Reason for rejection
Jouve et al. (2015)	203	similar to character 147 in this dataset
Jouve et al. (2015)	205	similar to character 46 in this dataset
Jouve et al. (2015)	226	varies intraspecifically
Jouve et al. (2015)	227	similar to character 147 in this dataset
Jouve et al. (2015)	238	similar to character 13 in this dataset
Salas-Gismondi et al. (2015)	199	no variation in taxa
Brochu and Storrs (2012)	189	no variation in taxa
Brochu and Storrs (2012)	182	all taxa scored as "?"
Brochu (2011)	106	similar to character 147 here
Brochu (2011)	20	no variation in taxa
Brochu (2011)	179	no variation in taxa
Buscalioni et al. (2011)	181	no variation in taxa
Buscalioni et al. (2011)	182	no variation in taxa
Buscalioni et al. (2011)	183	similar to character 13 in this dataset
Jouve et al. (2008)	170	similar to character 2 in this dataset
Jouve et al. (2008)	185	no variation in taxa
Jouve et al. (2008)	187	similar to character 16 in this dataset
Jouve et al. (2008)	192	similar to character 50 in this dataset
Jouve et al. (2008)	193	similar to character 50 in this dataset
Jouve et al. (2008)	194	considered impractical to score
Ösi et al. (2007)	166	autapomorphy of *Iharkutosuchus* makadii
Ösi et al. (2007)	167	no variation in taxa
Ösi et al. (2007)	169	no variation in taxa
Salisbury et al. (2006)	169	no variation in taxa
Salisbury et al. (2006)	172	similar to characters 188 and 189 here
Salisbury et al. (2006)	173	autapomorphy of *Isisfordia duncani* in this dataset
Ortega et al. (2000)	115	no variation in taxa
Ortega et al. (2000)	161	no variation in taxa
Brochu (1999)	1	no variation in taxa
Brochu (1999)	2	no variation in taxa
Brochu (1999)	56	anatomically invalid character (see character list)
Brochu (1999)	60	no variation in taxa
Brochu (1999)	125	uninformative as state 1 is autapomorphy of *Melanosuchus niger*
Brochu (1999)	160	uninformative state 1 is autapomorphy of *Gavialis gangeticus*
Brochu (1999)	161	similar to character 3 here
Brochu (1999)	163	no variation in taxa
Brochu (1999)	164	no variation in taxa

Table 1: Rejected characters

Study	Character	Reason for rejection
Norell and Clark (1990)	2	no variation in taxa
Norell and Clark (1990)	3	no variation in taxa
