# Supplementary material for: Phylogenetic analysis of a new morphological dataset elucidates the evolutionary history of Crocodylia and resolves the long-standing gharial problem
Source: PeerJ. 2021 Sep 6;9:e12094. doi: 10.7717/peerj.12094 (PMC8428266; doi:10.7717/peerj.12094)
Supplement: Supplemental Information 18 [file peerj-09-12094-s018.pdf]

Table 1: First and last appearance dates used in measures of stratigraphic congruence.

Taxon	FAD	LAD
Acresuchus pachytemporalis	11.62	5.333
Acynodon iberoccitanus	77.9	66
Agaresuchus fontisensis	77.9	69
Aktiogavialis caribesi	11.6	7.2
Alligator mcgrewi	20.43	15.97
Alligator mefferdi	13.8	10.3
Alligator mississippiensis	10.3	0
Alligator olseni	20.4	16
Alligator prenasalis	37.2	33.9
Alligator sinensis	3.6	0
Allodaposuchus precedens	77.9	66
Allognathosuchus polyodon	50.3	47.8
Allognathosuchus wartheni	56	50.3
Arambourgia gaudryi	37.8	33.9
Argochampsa krebsi	66	56
Asiatosuchus depressifrons	56	47.8
Asiatosuchus germanicus	47.8	41.2
Asiatosuchus nanlingensis	61.6	59.2
Australosuchus clarkae	28.1	16
Baru darrowi	16	11.6
Baru huberi	28.1	23
Baru wickeni	28.1	23
Bernissartia fagesii	129.4	125
Borealosuchus acutidentatus	66	63.3
Borealosuchus formidabilis	61.6	59.2
Borealosuchus sternbergii	70.6	63.3
Borealosuchus threensis	70.6	61.7
Borealosuchus wilsoni	56	47.8
Bottosaurus harlani	70.6	66
Boverisuchus magnifrons	47.8	41.2
Boverisuchus vorax	50.3	47.8
Brachychampsa montana	72.1	66
Brochuchus pigotti	20.4	16
Caiman brevirostris	11.6	5.3
Caiman crocodilus	2.58	0
Caiman gasparinae	11.6	7.2
Caiman latirostris	9	0
Caiman lutescens	11.6	7.2
Caiman wannlangstoni	16	5.3
Caiman yacare	11.6	0
Ceratosuchus burdoschi	56.8	56
Crocodylus acutus	2.6	0.1

Taxon	FAD	LAD
'*Crocodylus*' *affinis*	50.3	47.8
Crocodylus anthropophagus	1.8	0.01
Crocodylus intermedius	0.1	0
Crocodylus johnstoni	2.6	0
'*Crocodylus*' *megarhinus*	33.9	28.1
Crocodylus mindorensis	0.1	0
Crocodylus moreletii	0.1	0
Crocodylus niloticus	11.6	0
Crocodylus novaeguineae	0.1	0
Crocodylus palaeindicus	11.6	2.6
Crocodylus palustris	0.1	0.01
Crocodylus porosus	5.3	0
Crocodylus rhombifer	2.6	0
Crocodylus siamensis	2.6	0.8
Crocodylus thorbjarnarsoni	5.3	1.8
Caiman cf. *lutescens*, ('La Venta *Caiman*')	11.6	7.2
Dadagavialis gunai	23.03	15.97
Diplocynodon darwini	47.8	41.2
Diplocynodon deponiae	47.8	41.2
Diplocynodon hantoniensis	37.8	33.9
Diplocynodon muelleri	33.9	28.1
Diplocynodon ratelii	23	13.8
Diplocynodon remensis	59.2	56
Diplocynodon tormis	41.2	37.8
Dollosuchoides densmorei	47.8	41.2
Eocaiman cavernensis	47.8	37.8
Eocaiman palaeocenicus	66	61.6
Eogavialis africanum	38	28.4
Eosuchus lerichei	58.7	55.8
Eosuchus minor	59.2	47.8
Eothoracosaurus mississippiensis	72.6	66
Euthecodon arambourgi	23	16
Gavialis browni	11.6	5.3
Gavialis gangeticus	2.6	0
Gavialis lewisi	5.3	2.6
Gavialosuchus eggenburgensis	20.4	16
Glen Rose form	125	100.5
Globidentosuchus brachyrostris	11.6	5.3
Gnatusuchus pebasensis	16	11.6
Gryposuchus colombianus	16	11.6
Gryposuchus croizati	11.6	5.3
Gryposuchus neogaeus	11.6	7.2
Gryposuchus pachakamue	16	11.6
Hassiacosuchus haupti	47.8	41.2
Hylaeochampsa vectiana	127.2	125

Taxon	FAD	LAD
Iharkutosuchus makadii	86.3	83.6
Ikanogavialis gameroi	11.6	6.8
Isisfordia duncani	106.8	97.2
Jiangxisuchus nankangensis	70.6	66
Kambara implexidens	56	47.8
Kambara murgonensis	56	47.8
Kambara taraina	47.8	33.9
Kentisuchus spenceri	55.8	48.6
Kuttanacaiman iquitosensis	16	11.6
Leidyosuchus canadensis	77.9	72.1
Lohuecosuchus megadontos	77.9	69
Maomingosuchus petrolica	37.8	33.9
Maroccosuchus zennaroi	55.8	48.6
Mecistops cataphractus	11.6	0
Mekosuchus inexpectatus	0.01	0
Mekosuchus sanderi	16	11.6
Mekosuchus whitehunterensis	28.1	23
Melanosuchus niger	0.1	0
Mourasuchus amazonensis	11.6	7.2
Mourasuchus arendsi	11.6	5.3
Mourasuchus atopus	16	5.3
Navajosuchus mooki	66	61.6
Necrosuchus ionensis	61.6	59.2
Osteolaemus tetraspis	11.6	0
Paleosuchus palpebrosus	0.1	0
Paleosuchus trigonatus	0.1	0
Paratomistoma courti	41.3	38
Penghusuchus pani	11.5	5.3
Piscogavialis jugaliperforatus	11.6	5.3
Planocrania datangensis	61.6	59.2
Planocrania hengdongensis	59.2	56
Portugalosuchus azenhae	99.7	94.3
Procaimanoidea utahensis	46.2	41.2
Protocaiman peligrensis	66	61.6
Purussaurus barasiliensis	11.6	7.2
Purussaurus mirandai	11.6	5.3
Purussaurus neivensis	16	5.3
Quinkana	28.1	0.01
Shamosuchus djadochtaensis	83.6	72.1
Siquisiquesuchus venezuelensis	20.4	16
Stangerochampsa mccabei	70.6	66
Thecachampsa antiquus	13.8	5.3
Thecachampsa sericodon	15.97	11.6
Theriosuchus pusillus	145.5	140.2
Thoracosaurus isorhynchus	70.6	61.6

Taxon	FAD	LAD
Thoracosaurus neocesariensis	70.6	61.7
Tomistoma cairense	47.8	41.2
Tomistoma dowsoni	23	16
Tomistoma lusitanica	20.4	7.2
Tomistoma schlegelii	0.1	0
Toyotamaphimeia machikanensis	0.8	0.1
Trilophosuchus rackhami	16	11.6
Tsoabichi greenriverensis	56	47.8
Ultrastenos willisi	28.1	23
Voay robustus	0.1	0.01
Wannaganosuchus brachymanus	61.6	56
Wannchampsus kirpachi	119	113
