# Supplementary material for: Phylogenetic analysis of a new morphological dataset elucidates the evolutionary history of Crocodylia and resolves the long-standing gharial problem
Source: PeerJ. 2021 Sep 6;9:e12094. doi: 10.7717/peerj.12094 (PMC8428266; doi:10.7717/peerj.12094)
Supplement: Supplemental Information 19 [file peerj-09-12094-s019.pdf]

Table 1: Summary of constrained analyses.

Constraint	MPTs	Tree length	Tree length increase	Templeton test
(1) *Diplocynodon* monophyletic	3	8282.499	100.643	insignificant
(2) *Eocaiman* forced into Caimaninae	3	8190.625	8.769	insignificant
(3) *Eocaiman* forced into clade of South American caimanines	3	8254.124	72.269	insignificant
(4) Extant Jacareans (sensu Norell, 1988) monophyletic, with the following floating taxa: *Caiman wannlangstoni*, *Caiman brevirostris*, UCMP 39978, *Acresuchus pachytemporalis*, *Purussaurus*, and *Necrosuchus*	3	8182.617	0.761	insignificant
(5) Force *Portugalosuchus* out of Crocodylia, 'thoracosaurs' float	9	8829.43595	47.58	insignificant
(6) Force "Brevirostres", i.e. Alligatoroidea + "Crocodyloidea"	3	8187.359	5.503	significant
(7) 'Thoracosaurs' forced out of Crocodylia. *Portugalosuchus*, planocraniids, and *Borealosuchus* float	3	8373.965	192.110	significant
(8) 'Thoracosaurs' forced out of Gavialidae (*Gavialis*+*Tomistoma*), all non-thoracosaurine gavialoids float	3	8184.082	2.226	insignificant
(9) 'Thoracosaurs' forced out of Crocodylia (*Argochampsa*, *Eogavialis*, *Portugalosuchus*, planocraniids, *Borealosuchus* float)	9	8286.94	105.085	significant
(10) *Asiatosuchus germanicus*, *Asiatosuchus depressifrons*, and '*Crocodylus*' *affinis* forced out of Longirostres, *Jiangxisuchus* floats	3	8188.548	6.692	insignificant
(11) Monophyletic mekosuchinae forced outside of Longirostres, *Quinkana*, *Australosuchus* and *Asiatosuchus nanlingensis* float in Mekosuchinae and Crocodyloidea	3	8198.052	16.196	insignificant
(12) Force *Asiatosuchus nanlingensis* forced out of Mekosuchinae. *Australosuchus* and *Quinkana* float	3	8202.683	20.827	insignificant
(13) *Quinkana* and *Australosuchus* forced in Mekosuchinae, *Asiatosuchus nanlingensis* floats	3	8204.086	22.230	significant
(14) *Mecistops* forced into monophyletic Osteolaeminae	3	8201.109	19.254	insignificant
