# Supplementary material for: Phylogenetic analysis of a new morphological dataset elucidates the evolutionary history of Crocodylia and resolves the long-standing gharial problem
Source: PeerJ. 2021 Sep 6;9:e12094. doi: 10.7717/peerj.12094 (PMC8428266; doi:10.7717/peerj.12094)
Supplement: Supplemental Information 20 [file peerj-09-12094-s020.pdf]

Character	Node/Clade	Longirostrine character?	Synapomorphy type	*Thoracosaurus isorhynchus*	*Thoracosaurus neocesariensis*	*Eothoracosaurus mississippiensis*	*Eosuchus minor*	*Eosuchus lerichei*	*Portugalosuchus azenhae*	*Eogavialis africanum*	*Argochampsa krebsi*
107-1	Gavialoidea	No	Exclusive	1	1	1	1	0	0	1	?
136-1	Gavialoidea	No	Ambiguous	0	?	0	0	0	0	1	1
144-1	Gavialoidea	Yes	Ambiguous	1	?	0	0	0	?	1	1
151-2	Gavialoidea	Yes	Ambiguous	2	?	2	2	2	?	2	2
154-0	Gavialoidea	No	Ambiguous	0	?	0	0	0	?	0	0
183-1	Gavialoidea	No	Ambiguous	?	?	?	0	0	?	0	?
191-0	Gavialoidea	No	Ambiguous	0	?	?	0	0	?	0	0
206-0	Gavialoidea	No	Exclusive	?	0	0	1	0	?	0	0
41-1	Gavialoidea	No	Ambiguous	1	1	1	1	1	?	1	1
50-1	Gavialoidea	Yes	Ambiguous	1	1	1	1	1	?	1	1
75-2	Gavialoidea	No	Ambiguous	0	0	0	1	0	0	2	1
46-1	*Maomingosuchus* + *Gavialis*	Yes	Shared	1	?	1	1	1	?	1	1
98-1	*Maomingosuchus* + *Gavialis*	No	Ambiguous	1	?	?	?	0	1	1	?
220-0	*Maomingosuchus* + *Gavialis*	Yes	Ambiguous	?	?	0	1	0	?	0	0
224-2	*Maomingosuchus* + *Gavialis*	Yes	Ambiguous	?	?	2	2	2	?	3	?
219-3	Gavialidae	Yes	Ambiguous	?	?	3	3	3	?	3	?
221-3	Gavialidae	Yes	Exclusive	?	?	3	2	?	?	3	?
280-1	Gavialidae	No	Shared	?	?	?	?	?	?	?	?
328-0	Gavialidae	No	Shared	?	?	?	?	?	?	?	?
278-1	*Toyotamaphimeia* + *Gavialis*	No	Exclusive	1	1	?	1	0	?	1	?
193-1	*Toyotamaphimeia* + *Gavialis*	No	Ambiguous	1	1	1	?	0	1	0	1
247-0	*Toyotamaphimeia* + *Gavialis*	No	Ambiguous	?	?	0	0	?	?	0	1
276-1	*Toyotamaphimeia* + *Gavialis*	No	Ambiguous	1	1	?	1	1	?	?	0
288-0	*Toyotamaphimeia* + *Gavialis*	No	Exclusive	?	?	?	?	?	?	?	?
307-0	*Toyotamaphimeia* + *Gavialis*	No	Ambiguous	?	?	?	0	?	?	1	?
309-0	*Toyotamaphimeia* + *Gavialis*	No	Ambiguous	?	?	?	?	?	?	?	?
325-0	*Toyotamaphimeia* + *Gavialis*	No	Shared	0	?	?	0	0	?	0	0
327-0	*Toyotamaphimeia* + *Gavialis*	No	Ambiguous	?	?	0	0	1	?	0	1
89-0	*Toyotamaphimeia* + *Gavialis*	No	Shared	0	?	0	0	0	0	0	0
149-1	Penghusuchus + *Gavialis*	Yes	Shared	1	1	1	1	1	?	1	1
253-1	Penghusuchus + *Gavialis*	No	Ambiguous	?	?	1	1	?	?	?	?
302-0	Penghusuchus + *Gavialis*	No	Shared	0	0	?	1	?	?	?	0
90-0	’*T*’. *cairense* + *Gavialis*	No	Shared	0	?	?	0	0	0	0	1
147-7	’*T*’. *cairense* + *Gavialis*	Yes	Shared	7	?	7	7	7	?	7	7
304-0	’*T*’. *cairense* + *Gavialis*	No	Shared	0	0	?	?	?	?	?	?

Character	Node/Clade	Longirostrine character?	Synapomorphy type	*Thoracosaurus isorhynchus*	*Thoracosaurus neocesariensis*	*Eothoracosaurus mississippiensis*	*Eosuchus minor*	*Eosuchus lerichei*	*Portugalosuchus azenhae*	*Eogavialis africanum*	*Argochampsa krebsi*
63-1	*Portugalosuchus* + *Gavialis*	No	Shared	0	?	1	0	0	1	?	0
91-1	*Portugalosuchus* + *Gavialis*	No	Shared	0	?	?	?	?	1	1	1
94-1	*Portugalosuchus* + *Gavialis*	No	Ambiguous	1	?	1	0	0	1	1	1
115-0	*Portugalosuchus* + *Gavialis*	No	Shared	?	?	0	?	1	?	0	0
158-1	*Portugalosuchus* + *Gavialis*	Yes	Exclusive	0	?	1	1	0	?	1	1

Table 1: List of all synapomorphies for Gavialoidea and less inclusive clades that group 'thoracosaurs' and *Portugalosuchus* with other gavialoids. Red cells highlight character states that contrast with most or all other members of the respective node. Grey cells highlight missing data. Nodes described as *taxon x* + *taxon y*, indicate the least inclusive clade defined by those taxa. Underlined rows are exclusive synapomorphies that are discussed in the main text.
