# Supplementary material for: Phylogenetic analysis of a new morphological dataset elucidates the evolutionary history of Crocodylia and resolves the long-standing gharial problem
Source: PeerJ. 2021 Sep 6;9:e12094. doi: 10.7717/peerj.12094 (PMC8428266; doi:10.7717/peerj.12094)
Supplement: Supplemental Information 21 [file peerj-09-12094-s021.pdf]

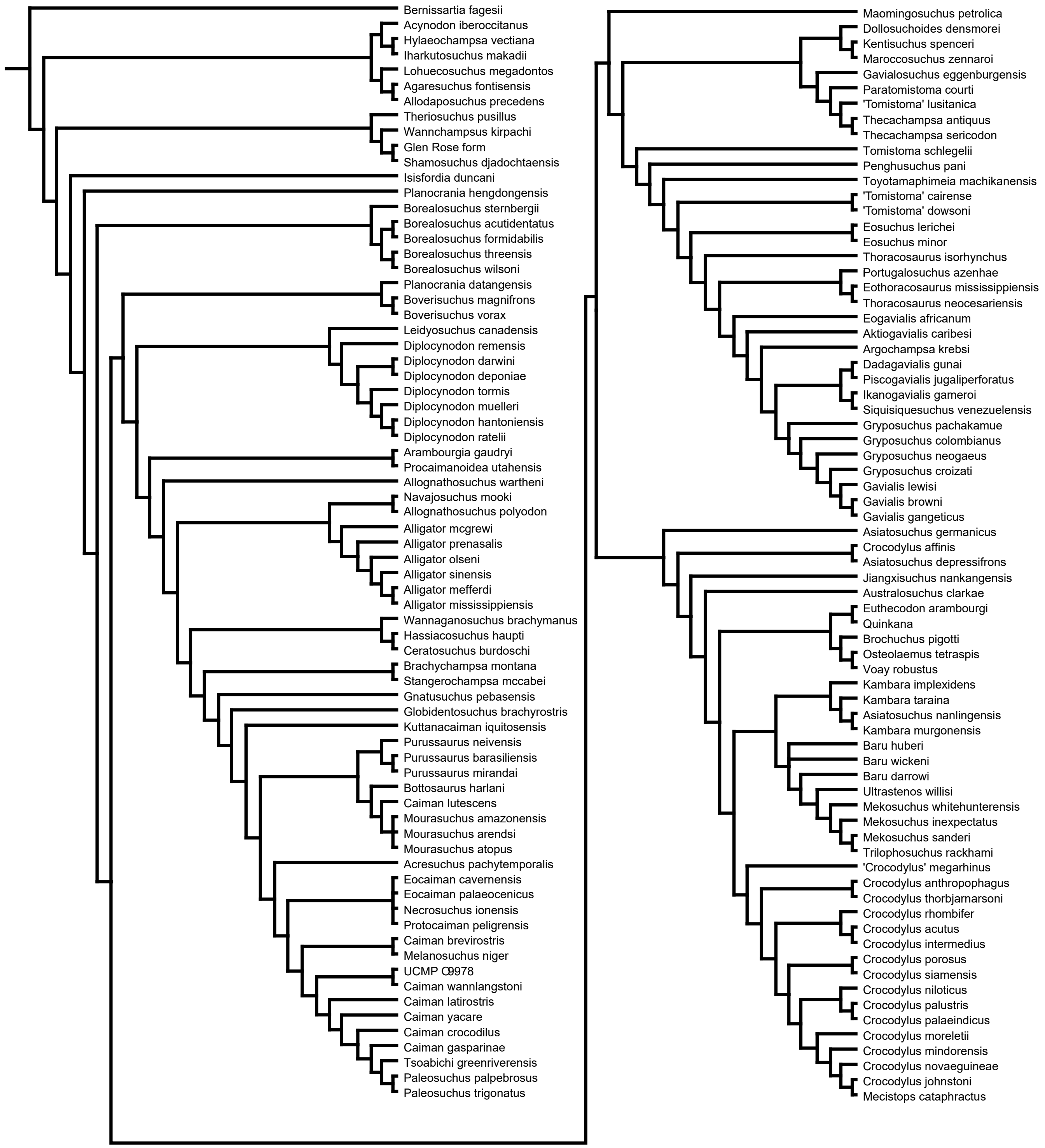

Figure 1: Strict consensus of 6 MPTs from Analysis 1.1

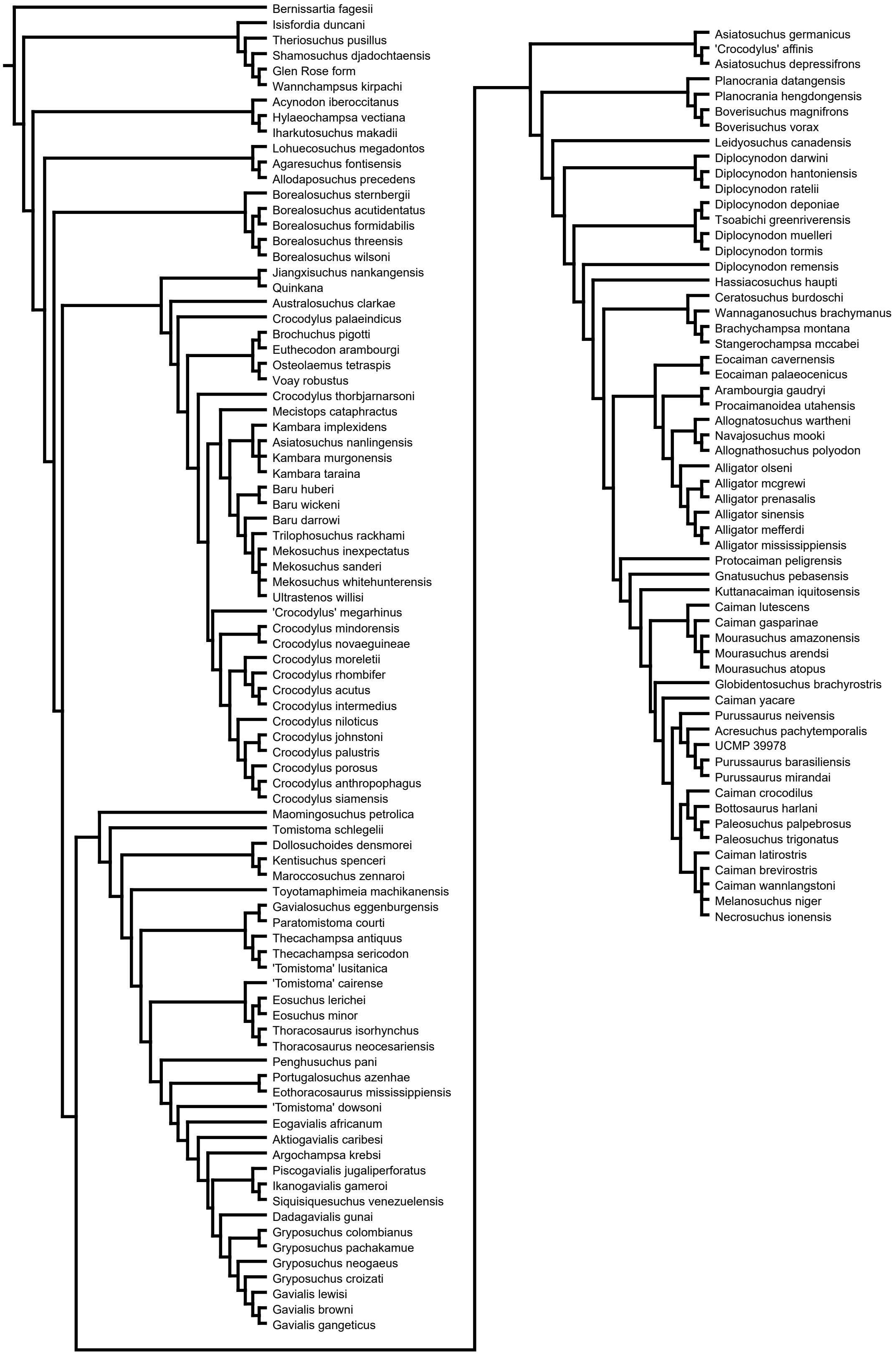

Figure 2: Strict consensus of 9 MPTs from Analysis 1.2

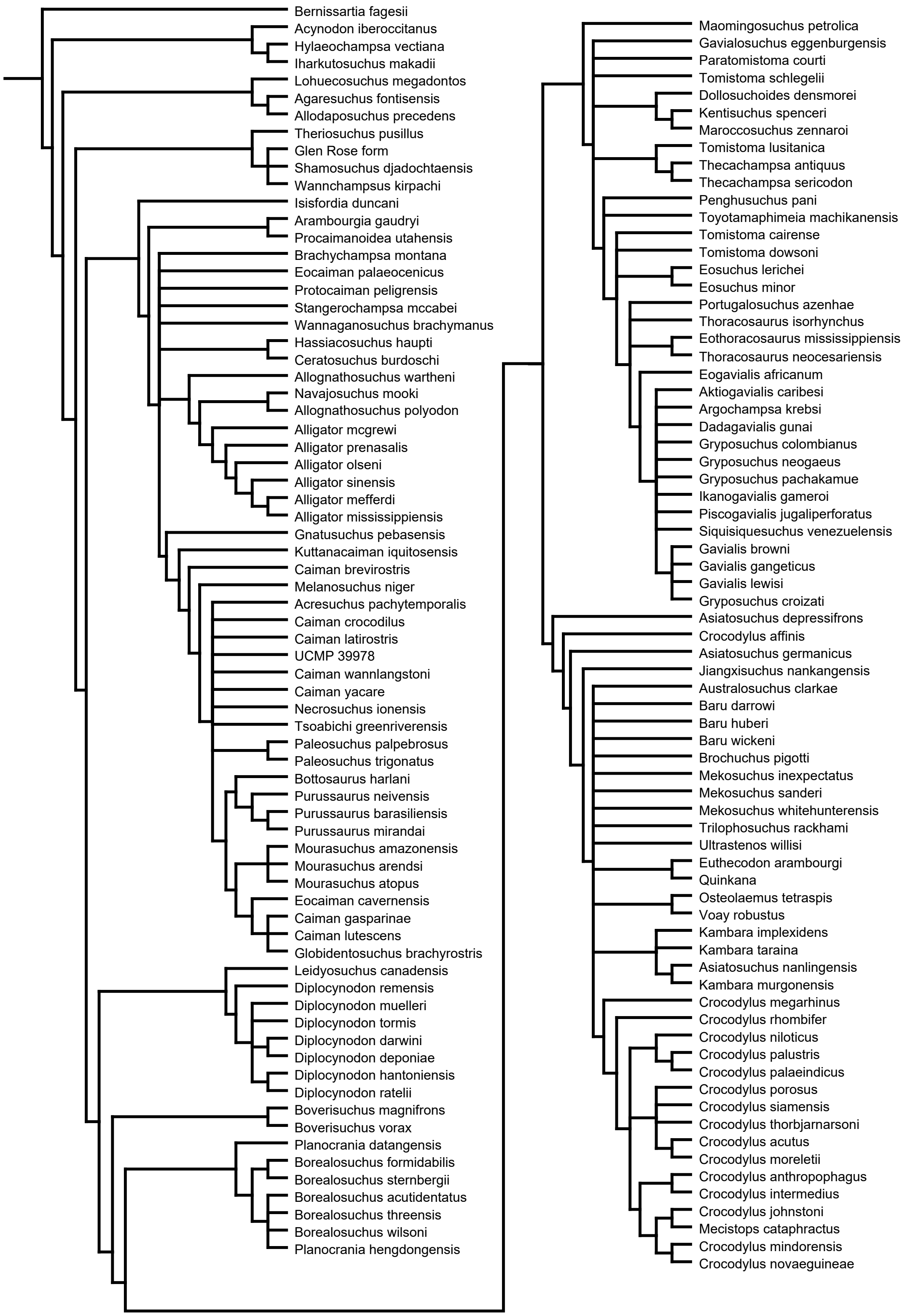

Figure 3: Strict consensus of 400,000 MPTs from Analysis 2.1

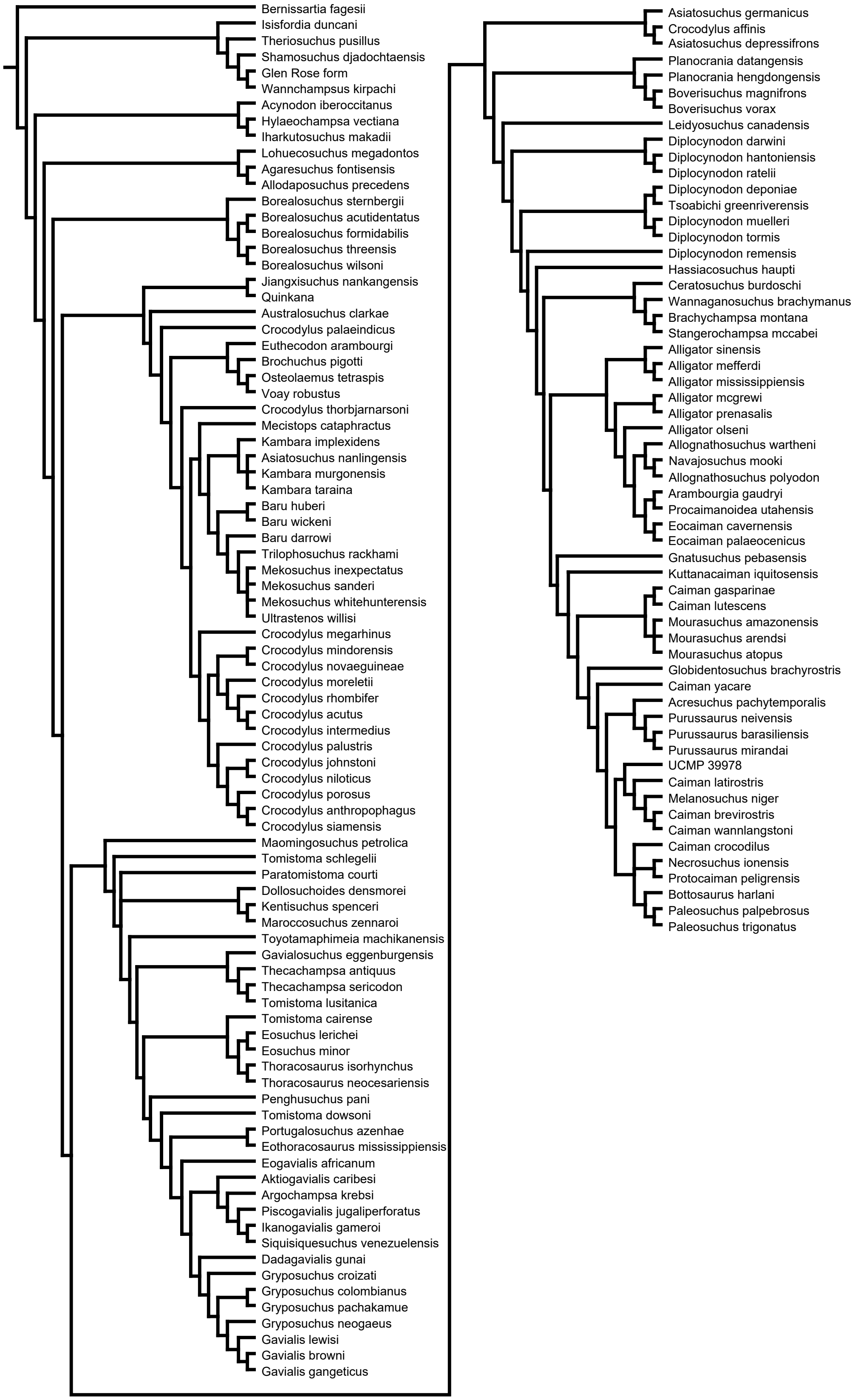

Figure 4: Strict consensus of 3 MPTs from Analysis 2.2

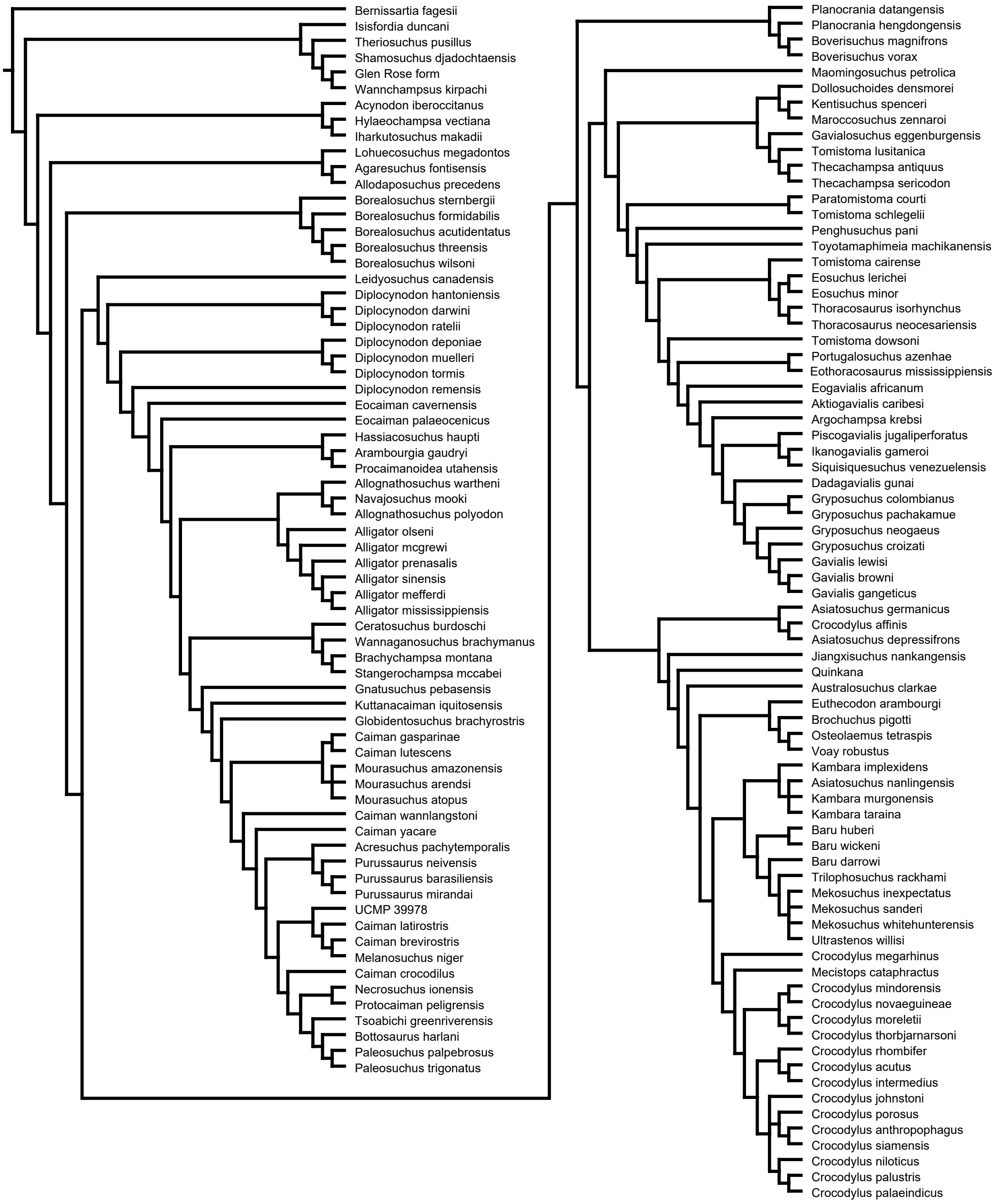

Figure 5: Strict consensus of 3 MPTs from Analysis 2.3

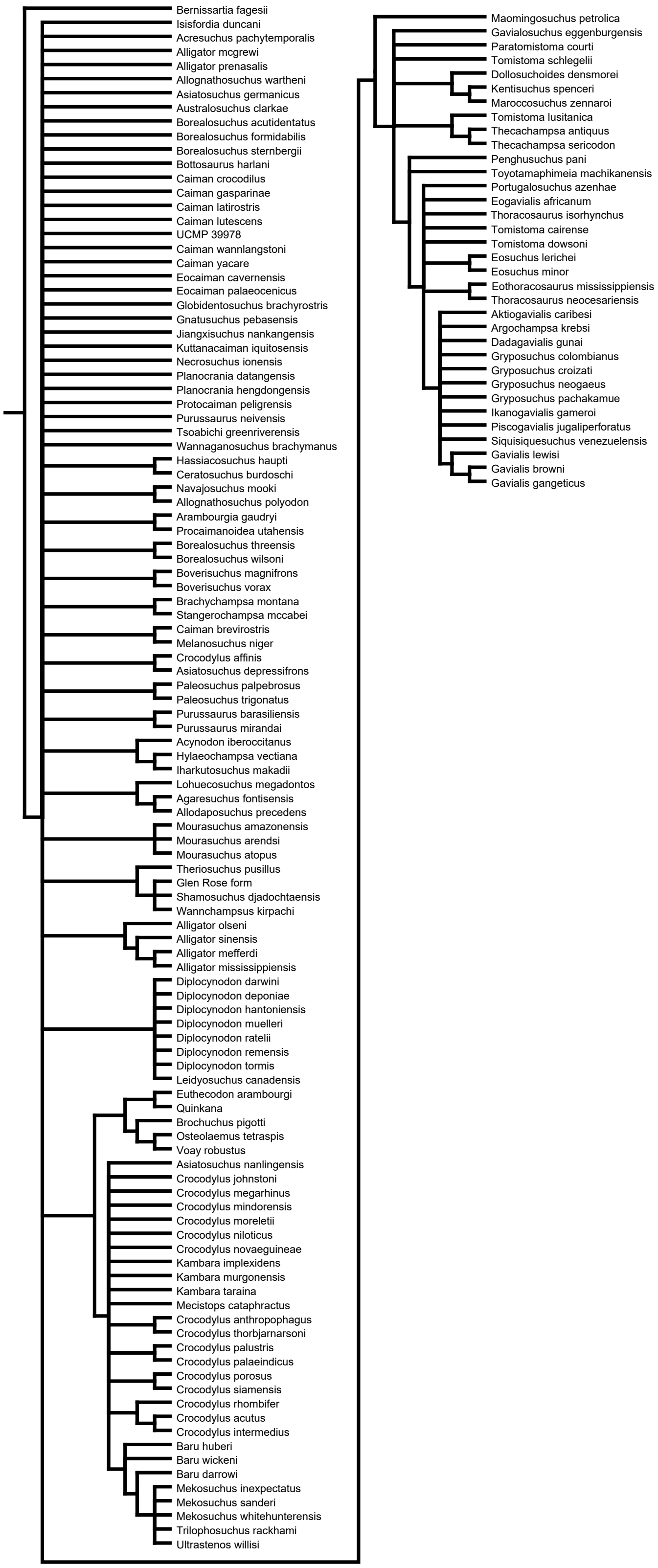

Figure 6: Strict consensus of 400,000 MPTs from Analysis 3.1

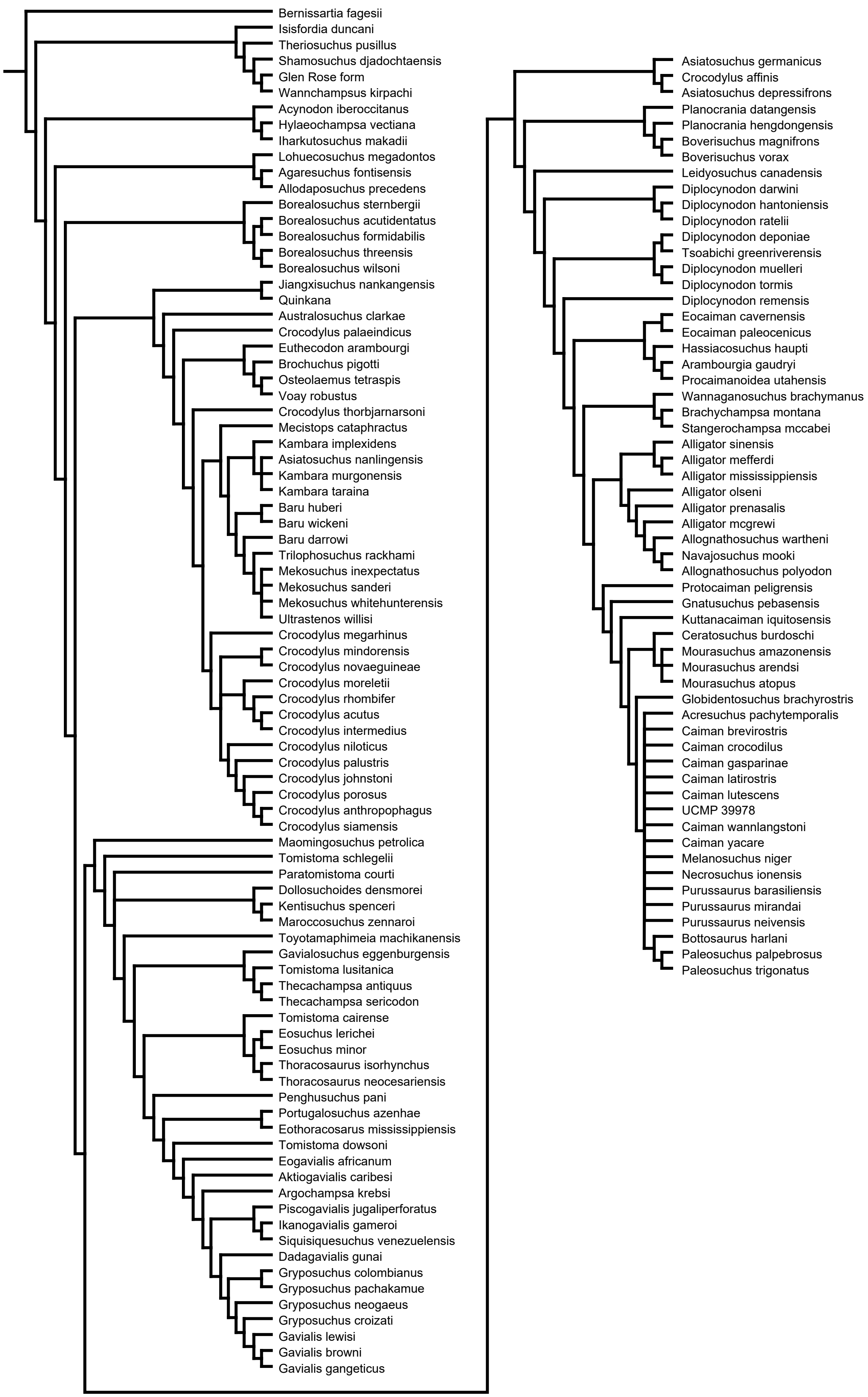

Figure 7: Strict consensus of 69 MPTs from Analysis 3.2

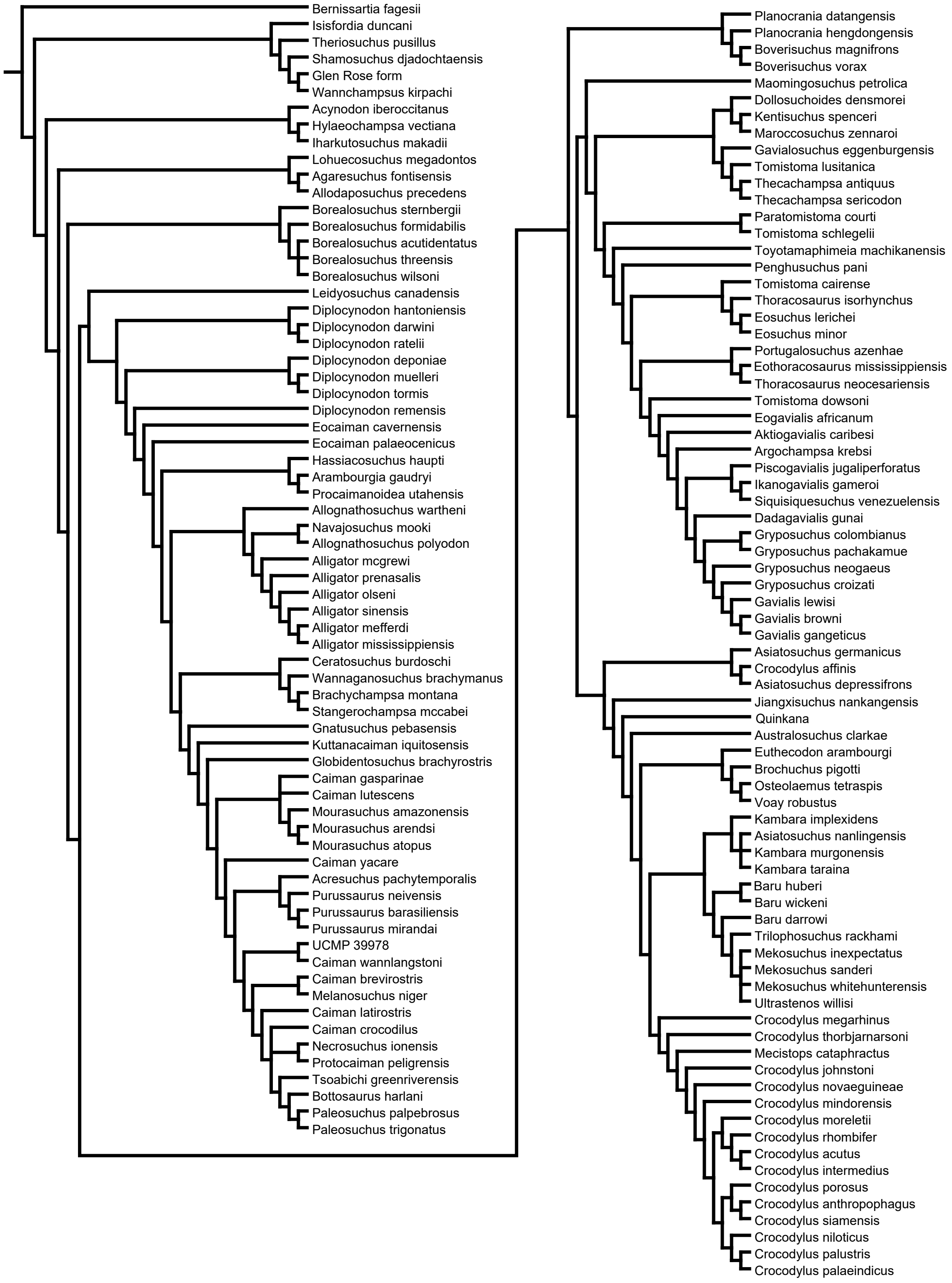

Figure 8: Strict consensus of 3 MPTs from Analysis 3.3
