# Supplementary material for: Phylogenetic analysis of a new morphological dataset elucidates the evolutionary history of Crocodylia and resolves the long-standing gharial problem
Source: PeerJ. 2021 Sep 6;9:e12094. doi: 10.7717/peerj.12094 (PMC8428266; doi:10.7717/peerj.12094)
Supplement: Supplemental Information 22 [file peerj-09-12094-s022.pdf]

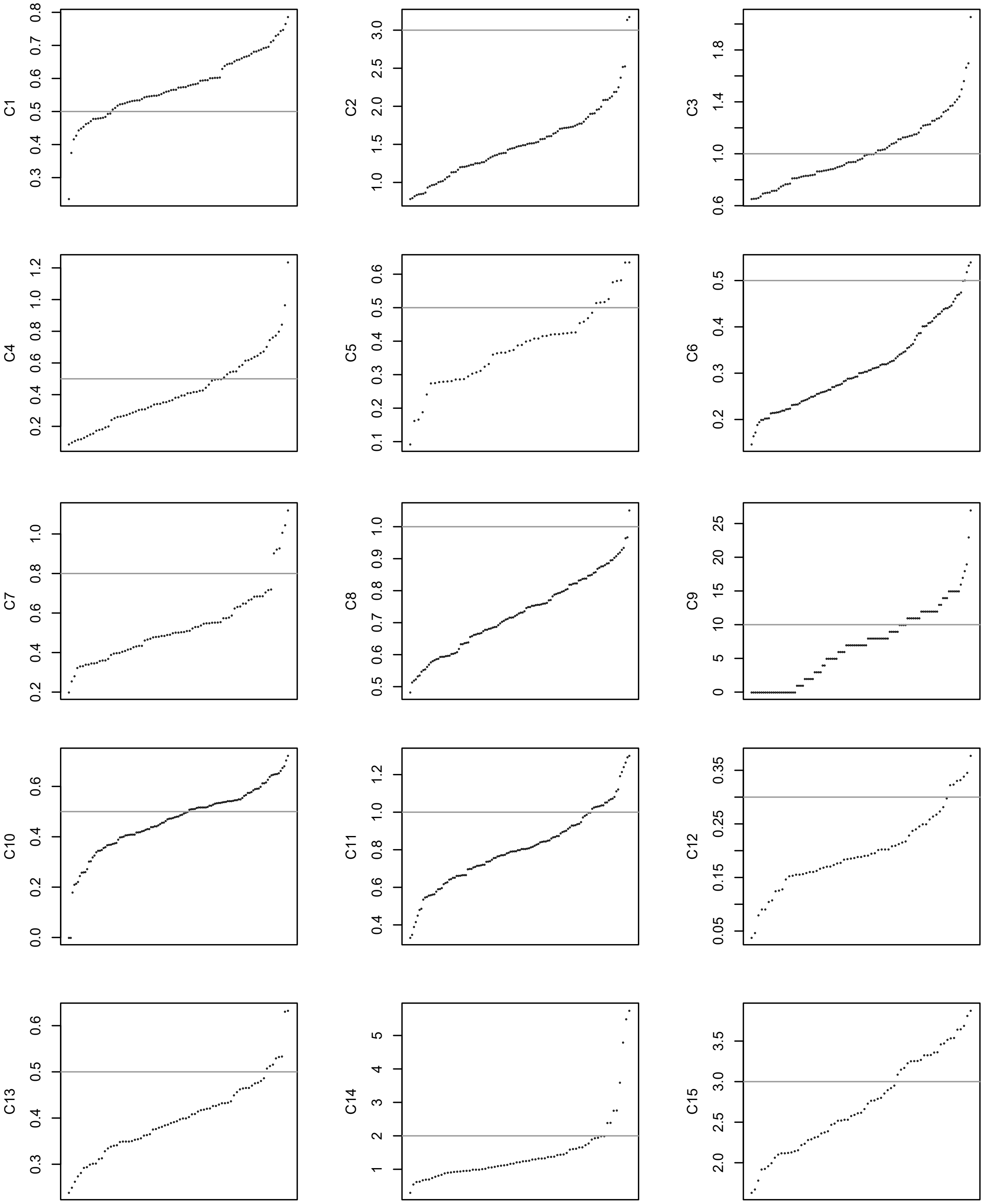

Figure 1: Measured values for continuous characters 1–15. Points represent the ratios of measured values for taxa in this analysis ordered from smallest to largest. Horizontal grey lines indicate the threshold value delimiting character states. See character list for character descriptions.

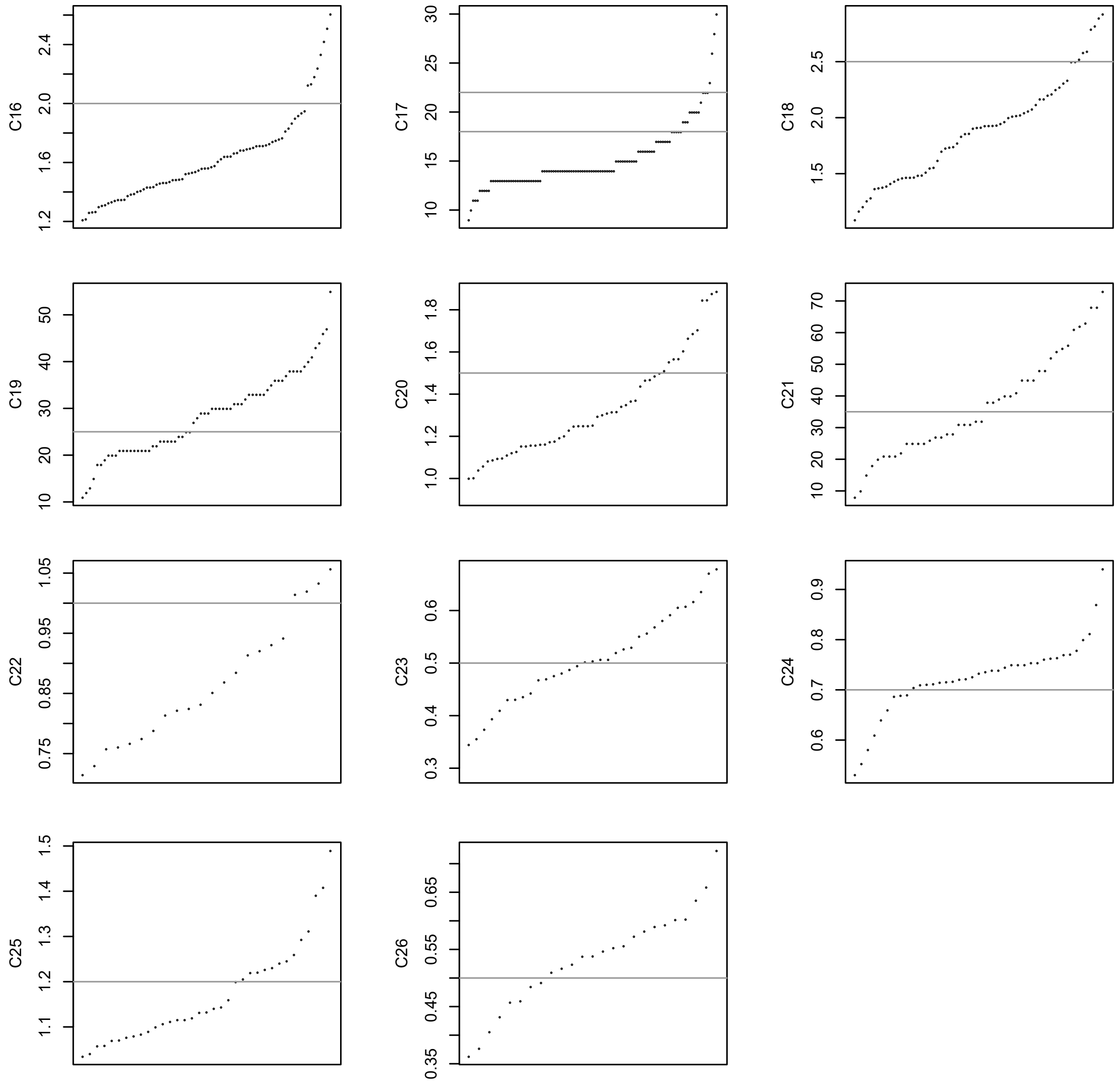

Figure 2: Measured values for for continuous characters 16–26. Points represent the ratios of measured values for taxa in this analysis ordered from smallest to largest. Horizontal grey lines indicate the threshold value delimiting character states. See character list for character descriptions.

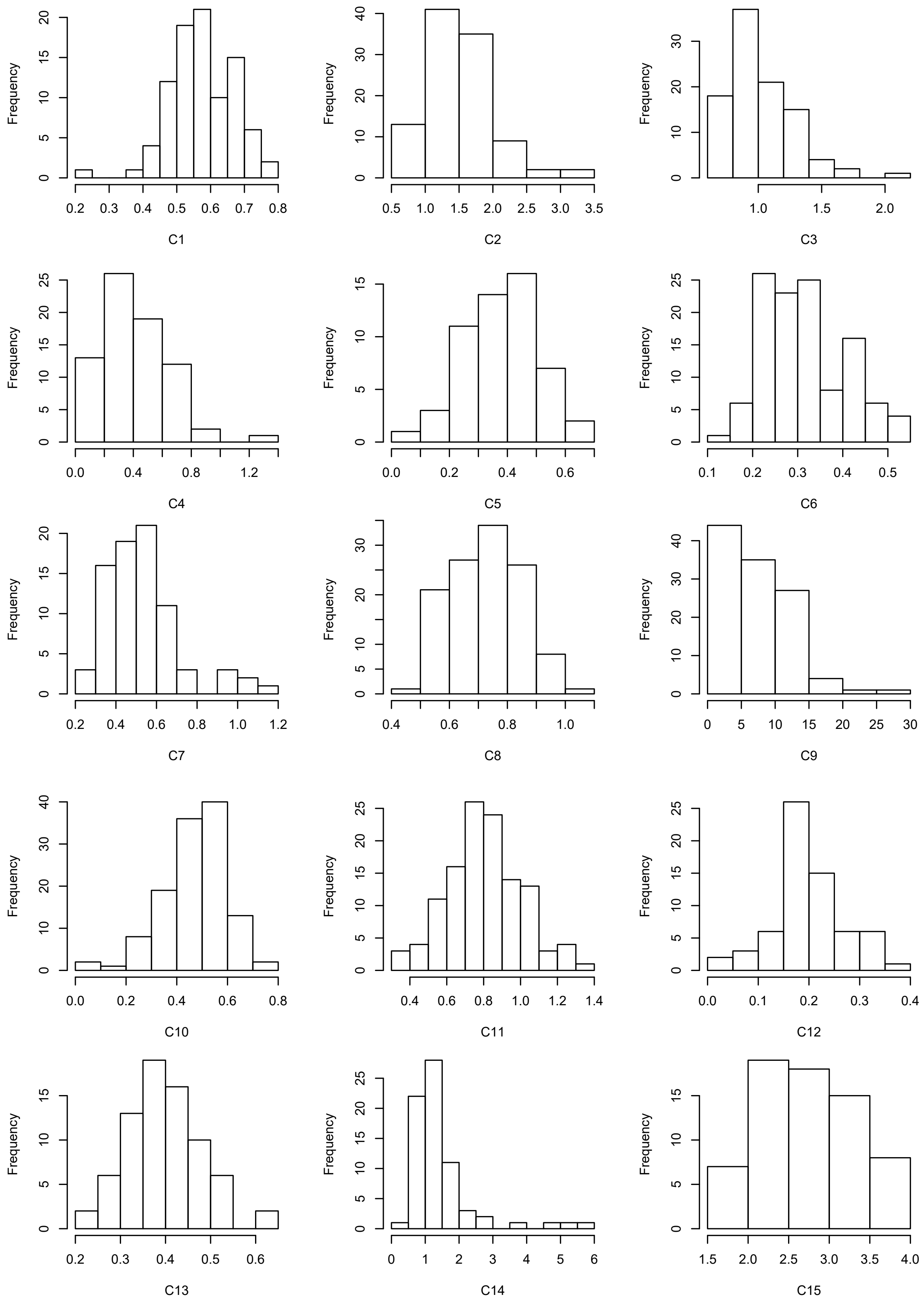

Figure 3: Histograms of measurements for characters 1–15

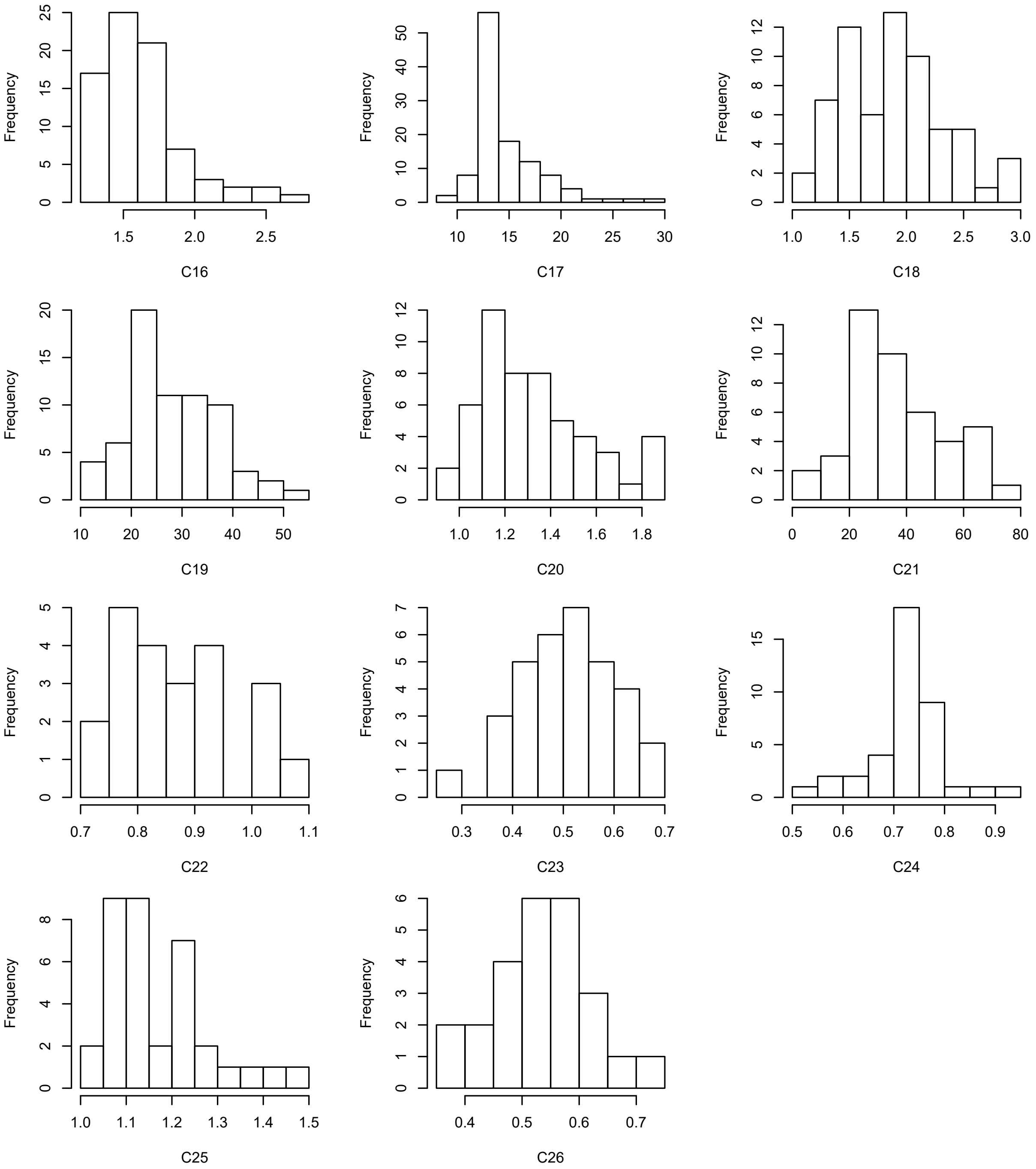

Figure 4: Histograms of measurements for characters 16–26
