# Supplementary material for: Phylogenetic analysis of a new morphological dataset elucidates the evolutionary history of Crocodylia and resolves the long-standing gharial problem
Source: PeerJ. 2021 Sep 6;9:e12094. doi: 10.7717/peerj.12094 (PMC8428266; doi:10.7717/peerj.12094)
Supplement: Supplemental Information 26 [file peerj-09-12094-s026.pdf]

Character CI values

1. 0.0979
2. 0.3422
3. 0.1562
4. 0.1645
5. 0.0874
6. 0.0425
7. 0.1374
8. 0.0438
9. 0.0236
10. 0.0824
11. 0.0909
12. 0.0578
13. 0.0498
14. 0.0116
15. 0.2266
16. 0.1544
17. 0.0404
18. 0.3049
19. 0.0649
20. 0.1693
21. 0.0976
22. 0.0889
23. 0.0947
24. 0.2022
25. 0.1237
26. 0.1212
27. 0.3333
28. 0.3333
29. 1
30. 0.0833
31. 0.0769
32. 0.1667
33. 0.1429
34. 0.5
35. 1
36. 1
37. 0.1429
38. 0.1667
39. 1
40. 1
41. 0.0588
42. 0.5
43. 0.5
44. 0.1429
45. 1
46. 0.0909
47. 0.25
48. 0.3333
49. 0.0909
50. 0.0833
51. 0.5
52. 0.1667
53. 0.1
54. 0.0476
55. 0.1429
56. 0.1667
57. 0.0833
58. 0.0571
59. 0.3333
60. 0.0909
61. 0.1111
62. 0.1429
63. 0.05
64. 0.5
65. 1
66. 1
67. 0.25
68. 1
69. 0.3333
70. 0.1
71. 0.5
72. 0.087
73. 0.0667
74. 0.0455
75. 0.0625
76. 0.0345
77. 0.1111
78. 0.1333
79. 0.0588
80. 0.3333
81. 0.3333
82. 0.1667
83. 0.2
84. 0.3333
85. 0.5
86. 1
87. 0.2
88. 0.4
89. 0.1429
90. 0.1667
91. 0.3333
92. 0.1667
93. 0.1111
94. 0.15
95. 0.25
96. 0.0769
97. 0.25
98. 0.1667
99. 0.1
100. 0.2
101. 1
102. 0.1667
103. 0.25
104. 0.5
105. 0.1429
106. 0.1667
107. 0.1667
108. 0.1
109. 0.1538
110. 0.2222
111. 1
112. 0.3333
113. 0.5
114. 0.125
115. 0.0714
116. 1
117. 0.3333
118. 0.1667
119. 0.1765
120. 1
121. 0.125
122. 1
123. 0.2
124. 0.1
125. 1
126. 0.2
127. 0.25
128. 0.3333
129. 0.25
130. 0.0909
131. 0.25
132. 0.2
133. 0.5
134. 0.1667
135. 0.1429
136. 0.1667
137. 0.3333
138. 0.1111
139. 0.0714
140. 0.5
141. 0.1304
142. 0.1316
143. 0.3333
144. 0.1667
145. 0.05
146. 0.1
147. 0.5833
148. 0.1111
149. 0.2
150. 0.125
151. 0.1667
152. 0.1667
153. 0.5
154. 0.0556
155. 0.3333
156. 0.2
157. 1
158. 0.3333
159. 0.3333
160. 0.1
161. 0.125
162. 0.1
163. 0.0667
164. 0.1667
165. 0.5
166. 0.125
167. 0.0833
168. 0.1429
169. 0.125
170. 1
171. 0.1111
172. 0.2
173. 0.0588
174. 0.0667
175. 0.0769
176. 0.3333

177. 0.3333
178. 1
179. 0.3333
180. 0.2
181. 0.1
182. 1
183. 0.125
184. 0.0833
185. 0.25
186. 0.5
187. 1
188. 0.6667
189. 0.2
190. 0.2727
191. 0.0833
192. 0.1667
193. 0.0833
194. 0.2
195. 0.375
196. 0.2
197. 0.0667
198. 1
199. 1
200. 0.5
201. 0.25
202. 0.25
203. 0.25
204. 0.3333
205. 0.25
206. 0.25
207. 0.3333
208. 0.5
209. 0.3
210. 0.5
211. 0.2
212. 0.25
213. 1
214. 0.1333
215. 0.1429
216. 0.2
217. 0.2
218. 0.3333
219. 0.3077
220. 0.2222
221. 0.1379
222. 0.0909
223. 1
224. 0.3
225. 0.3333
226. 0.0909
227. 0.25
228. 1
229. 0.0769
230. 0.6667
231. 1
232. 1
233. 1
234. 0.5
235. 0.1818
236. 0.1429
237. 0.1
238. 0.3333
239. 0.1429
240. 0.0909
241. 0.1429
242. 0.125
243. 0.125
244. 0.0909
245. 0.1
246. 0.1429
247. 0.1111
248. 0.25
249. 0.125
250. 0.5
251. 0.0769
252. 0.25
253. 0.0833
254. 0.2
255. 0.5
256. 0.3333
257. 0.25
258. 0.1429
259. 0.5
260. 1
261. 0.1667
262. 0.3333
263. 0.5
264. 0.2
265. 0.25
266. 0.5
267. 0.2
268. 0.5
269. 1
270. 0.5
271. 0.5
272. 0.5
273. 0.0714
274. 0.0909
275. 0.1667
276. 0.3333
277. 0.1429
278. 0.5
279. 0.5
280. 0.1667
281. 100
282. 0.3333
283. 1
284. 0.2857
285. 0.5
286. 1
287. 1
288. 1
289. 0.1667
290. 1
291. 1
292. 1
293. 0.6667
294. 0.5
295. 1
296. 0.3333
297. 0.2857
298. 1
299. 0.2
300. 0.5
301. 0.1667
302. 0.1429
303. 0.5
304. 0.5
305. 0.5
306. 0.3333
307. 0.0909
308. 0.1538
309. 0.1
310. 1
311. 0.25
312. 0.5
313. 1
314. 1
315. 1
316. 0.2
317. 1
318. 0.3333
319. 0.5
320. 1
321. 0.2
322. 0.25
323. 0.4
324. 0.3636
325. 0.25
326. 0.1
327. 0.1111
328. 0.25
329. 0.3333
330. 0.5
